# Supplementary material for: Identification of plants’ functional counterpart of the metazoan mediator of DNA Damage checkpoint 1
Source: EMBO Rep. 2024 Mar 4;25(4):19. doi: 10.1038/s44319-024-00107-8 (PMC11014961; doi:10.1038/s44319-024-00107-8)
Supplement: Supplementary file 1 — Figure Source Data for EV [file 44319_2024_107_MOESM1_ESM.zip › ExpandedViewSourceData/Figure EV2/2A/EMBOR-2024-58742V1_SourceDataForExpandedView2A.pdf]

```
Mesculenta_BCP1        TCKVCGSHDRGEVMLIC--GDESGSVGCGIGMHIDCCDP--PLENIPEEDWFCPNCS
Spurpurea_BCP1         ACEVCGSRDRGEVMLIC--SDESGSAGCGVGMHMDCCDP--PLESIPEEDWFCPKCS
Ptrichocarpa_BCP1      ACEVCCSCDRGEDMLIC--GDECGSVGCGAGIHIDCCDP--PLESIPEEDWFCPKCS
Csinensis_BCP1         TCQACGCSDRGEVMLIC--GDESGSVGCGVGMHIDCCDP--PLESVPEEDWFCPTCT
Tcacao_BCP1            TCQVCGSPERGEVMLIC--GDESGSVGCGVGIHIDCCDP--PLEDVPDDDWFCPKCS
Graimondii_BCP1        TCQVCGSGDREDVMLIC--GNESGSVGCGIGIHIDCCDP--PLDNVPEEDWFCPKCN
Cpapaya_BCP1           VCQVCGCHDREEVMLIC--GDESGSTGCGTGAHIDCCDP--PLHEVPEGDWFCPKCN
Mdomestica_BCP1        PCVVCGSGERGEVMLIC--GNESGSVGCGIGTHIECCNP--PLESVPEDDWFCPDC-
Ppersica_BCP1          PCVVCGSVERGEVMLIC--GNESGSVGCGVGTHIDCCNP--PLEVVPEGDWFCPKC-
Fvesca_BCP1            PCVVCGSCDRGEVMLIC--GNESGSVGCGIGTHIDCCSP--PLEDIPEGDWFCPKCC
Mtruncatula_BCP1       ACQVCGSRERGDVMLIC--GDESGSVGCGVGTHIDCCDP--PLAAVPEEDWFCPKCS
Tpratense_BCP1         ACQVCGSQERGDVMLIC--GDESGSVGCGVGTHIDCCNP--PLTVVPEEDWFCPKCS
Carietinum_BCP1        ACRVCRSRERGDVMLIC--GDESGSVGCGMGTHIDCCDP--PLTAVPEEDWFCPKCI
Pvulgaris_BCP1         TCQVCGSRDRGDVMLIC--GDESGSVGCGEGTHIDCCDP--PLTDVPEYDWFCSKCS
Gsoja_BCP1             ICKVCGSRDRGDVMLIC--GDESGSVGCGIGTHIDCCDP--PLTHVPEEDWFCPKCS
Ahypogaea_BCP1         ACEVCGSRDRGDVMLIC--GDESGSVGCGVGTHIDCCDP--PLMDVPEEDWFCSKCI
Lusitatissimum_BCP1    ACQVCGSRDRGEVMLLC--GDESGKVGCGGGRHIDCCDP--PLDSIPEEDWFCPNCC
Carabica_BCP1          TCQVCGSRDRGEVMLIC--GDENGSSGCGIGTHIDCCDP--PLEEIPQEDWFCPNCR
Dcarota_BCP1           PCEVCGSRDRGEDMLIC--GSESGTKGCGTGRHIDCCDP--PLPDVPSEDWFCPKCS
Csativus_BCP1          ACQECGSRDRGEVMLIC--GNEDGSSGCGIGMHTDCCNP--PLLDIPEGDWFCSDCI
Mguttatus_BCP1         ACQVCGSRDRGEEMLIC--GSEHGSVGCGVGVHLNCLDP--PLVDVPKEDWFCRSCS
Cquinoa_BCP1           ACQVCGSTDRGEVMLVC--GDESGSTGCGIGTHIDCCDP--PLESIPEEDWFCPKC-
Slycopersicum_BCP1     ACQVCGSPDRGEVMLIC--GDESGSLGCGIGMHVDCCDP--PLECIPEEDWFCPECS
Lsativa_BCP1           ACEVCGSRDRGEEMLMC--GNESGSIGCGVGMHIDCCDP--PFEDVPEEDWFCPNC-
Ahypochondriacus_BCP1  ACQVCGSTDRGEVMLIC--GNENGSVGCGIGTHIDCCDP--PLEKIPEEDWFCPQCA
Soleracea_BCP1         ACQICGLTDRGEVMLVC--GNESGTTGCGVGSHIDCCDP--PLKDIPEEDWFCPKCY
Hannuus_BCP1           ACEVCGARERGDEMLIC--GDEGGSIGCGVGTHIDCCDP--PLAEVPEEDWFCSKCR
Pamilis_BCP1           ACQVCGSTDRAEVMLIC--GDESGSSGCGLGTHIDCLDP--PLESVPEEDWLCSKCC
Klaxiflora_BCP1        KCCVCGSGDRGHQMLIC--GDENGSVGCGIGTHVECCDP--PLQEIPDDDWFCPKCS
Acoerulea_BCP1         CCEKCGSFDRAEVMLIC--GDETGSLGCGIGTHIDCCDP--PLEAVPDEDWFCPNCT
Ltulipifera_BCP1       SCEVCGSRDRGGVMLIC--GDERGTLGCGVGTHIDCCKP--PLEAVPKEDWFCSKCQ
Ckanehirae_BCP1        ACEVCGSHERGDVMLIC--GDEGGSVGCGKGTHIDCCDP--PLEAVPEGDWFCSKCR
Ncolorata_BCP1         VCSVCKLNDRAEVMLIC--GDDAGKVGCGVAMHIDCCDP--PLDSVPEDDWFCSKCC
Atrichopoda_BCP1       ICDVCHSGERANVMLLC--GDENGA-GCGVGRHIDCCDP--PFEAVPEEDWFCSERC
Sitalica_BCP1          SCSACGCKDRGEVMLIC-GD-EDGETGCGIGMHIDCCDP--PLDAVPDDDWLCPKCA
Sviridis_BCP1          SCSACGCKDRGEVMLIC-GD-EDGETGCGIGMHIDCCDP--PLDAVPDDDWLCPKCA
Pvirgatum_BCP1         SCSACGLKDRGEVMLIC-GD-EDGETGCGIGMHIDCCDP--PLDAVPDDDWLCPKCA
Sbicolor_BCP1          SCSVCGQKDRGDVMLIC-GD-EDGETGCGIGMHIDCCDP--PLDAVPDDDWLCPKCA
Msinensis_BCP1         SCSACFRKDRGDVMLIC-GE-EDGEIGCGIGMHIDCCDP--PLEAVPDDDWLCPKCA
Osativa_BCP1           SCSACGSTDRGEVMLIC-GN-EDGSTGCGVGMHIDCCDP--PLEAVPEHDWLCPQCE
Tintermedium_BCP1      SCAACGSTDRAEVMLIC-GS-EDGTVGCGAGMHIDCCDP--PLDRVPDDDWLCPKCE
Jascendens_BCP1        SCSVCGSNDREDVMLIC-GD-EDGATGCGIGAHIDCCDP--PLEAVPECDWLCPKCD
Platifolius_BCP1       SCSVCGCKDREDVMLIC-GE-EDSTSGCGVGMHIDCCDP--PIEAVPDGEWLCPKCD
Bdistachyon_BCP1       SCSACGSNNREGSQLICSGG-EGNQASCGVAMHVDCWNPHHP-EPVPDGEWLCPKCD
Bstacei_BCP1           SCSACGSNNREGSQLICSGG-DGSRAVCGAAMHVDCWNPHHP-EPVPDGEWLCPKCD
Zmays_BCP1             SCSACGSDNREVPMLVCGGDGRSQSSGCGVRTHADCCNP--PVEGCDGGEWLCGRCD
Othomaeum_BCP1         VCSTCGSSDRDRPMLVCGSD--GNSAGCGIVVHVDCCDPAVEAGA-RAGEWLCTKCD
Macuminata_BCP1        SCAVCGSAGRGEVMLIC--GDEAGAVGCGIGTHIDCCDP--PLDSVPEGDWYCSKCS
Aofficinalis_BCP1      SCSVCGLNDRGEVMLMC--GYEDGSVGCGIGTHIDCCNP--PLEAVPEGDWFCTKCS
Acomosus_BCP1          SCTVCGSRERGEVMLIC--GNEGGTVGCGIGTHIDCCNP--PMEAIPEGDWFCAKCS
Spolyrhiza_BCP1        SCSVCGSLERGDVMLIC--GDEQGLTGCGAAMHTDCCQP--PLSAVPEDDWFCPRCA
Zmarina_BCP1           KCVVCKRHDRGEVMLMC--GDEEGSIGCGIATHIDCCVP--PFESVPKEDWFCEKCS
Tplicata_BCP1          TCTTCGSADRDDVMLLC--GDEQGR-GCGIAMHIDCCQP--PLEEVPEDDWFCFKCK
Dcomplanatum_BCP1      ACFVCAQTDREDVMLLC--GDDQGR-GCGVAIHIDCCQP--SLQSVPEEDWFCIACS
Itaiwanesis_BCP1       ACDACGRIDREEVMLLC--GDGHGK-GCGRAMHIDCCRP--PLKRVPKDDWFCSQCK
Aangustus_BCP1         FCSVCKKGDREDVMMLC--GDDKGR-GCGVAMHIYCCKP--PLSEVPEEDWFCKKCL
Aagrestis_BCP1         FCSVCKKGDREDVMMLC--GDDKGR-GCGVAMHIYCCKP--PLSEVPEEDWFCNKCL
Scucullata_BCP1        RCSACGLSDRDDVMLLC--GDG-GN-GCGIAMHIDCCKP--PLAEVPANDWYCMQCT
Afiliculoides_BCP1     RCSVCGSSDREDVMLLC--GDDIGN-GCGIAMHTDCCKP--PLSEVPVNDWFCVQCT
Mvestita_BCP1          SCAVCGQSDRGDVMLLC--GDGEGS-GCGCAMHIDCCEP--PLDEVPSCDWYCMECT
Cricardii_BCP1         CCVVCGQSDREDVMLLC--GDDKGS-GCGTAMHTDCCNP--PLKEVPPHDWYCTHCT
Acapillus_BCP1         CCAVCGRSDREDVMLLC--GDDKGS-GCGTAMHIDCCKP--PLEKVPANDCFNEFTI
Aspinulosa_BCP1        CCSVCGLSDREDVMLLC--GDDKGS-GCGTAMHIDCCKP--PLDEVPVQDWYCKQCT
Mpolymorpha_BCP1       ACVVCGRTDDEKVMLLC--GDGKGN-GCGLATHIHCCSP--PLFEVPEEDWFCRNCE
Smoellendorffii_BCP1   ACNVCGRKDSEEVMLFC-DGDD-----CEVATHTFCLEP--PLDKVPAGDWYCARCS
Mendlicherianum_BCP1   VCEACRRADMEDVMLLC--GDDEGQ-GCGRALHIQCMEP--PLDRVPDEDWFCEYCS
Cbraunii_BCP1          ACEVCGKADKEDKMVLC--GDDLGKRGCGIGTHIFCMTP--PLKRVPDEDWFCNKCK
Smuscicola_BCP1        ACTKCHRTDDEDIMILC--GDDRGR-GCSLAVHIHCLTP--PLKTVPQEDWFCSSCS
Knitens_BCP1           ACGVCGRVDQEAEMVLC----DGRGGQCDLAVHIFCMAP--PLKQVPEGDWFCSICQ
```

Source data for Figure EV2A. Sequence alignment of the PHD finger from BCP1 with cysteine residues shaded in green and histidine in blue.
