# Supplementary material for: Identification of plants’ functional counterpart of the metazoan mediator of DNA Damage checkpoint 1
Source: EMBO Rep. 2024 Mar 4;25(4):19. doi: 10.1038/s44319-024-00107-8 (PMC11014961; doi:10.1038/s44319-024-00107-8)
Supplement: Supplementary file 1 — Figure Source Data for EV [file 44319_2024_107_MOESM1_ESM.zip › ExpandedViewSourceData/Figure EV5/EV5C/EMBOR-2024-58742V1_SourceDataForExpandedView5C.pdf]

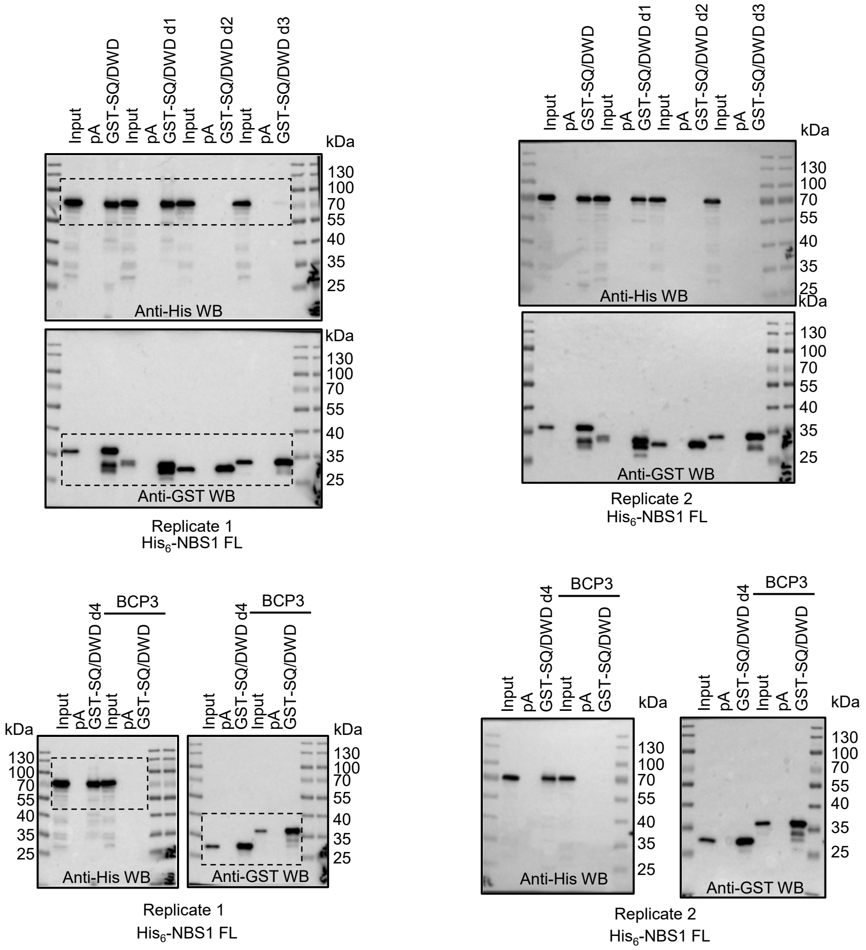

Source data for Figure EV5C. Uncropped images of pull-down western blots demonstrating interaction of NBS1 full length (FL) with SQ/DWD deletion mutants of BCP4. Dashed boxes correspond to images presented in Fig EV5C. Shown are chemiluminescence signals overlayed with membranes.
