# Supplementary figures and images for: Identification of plants’ functional counterpart of the metazoan mediator of DNA Damage checkpoint 1
Source: EMBO Rep. 2024 Mar 4;25(4):19. doi: 10.1038/s44319-024-00107-8 (PMC11014961; doi:10.1038/s44319-024-00107-8)

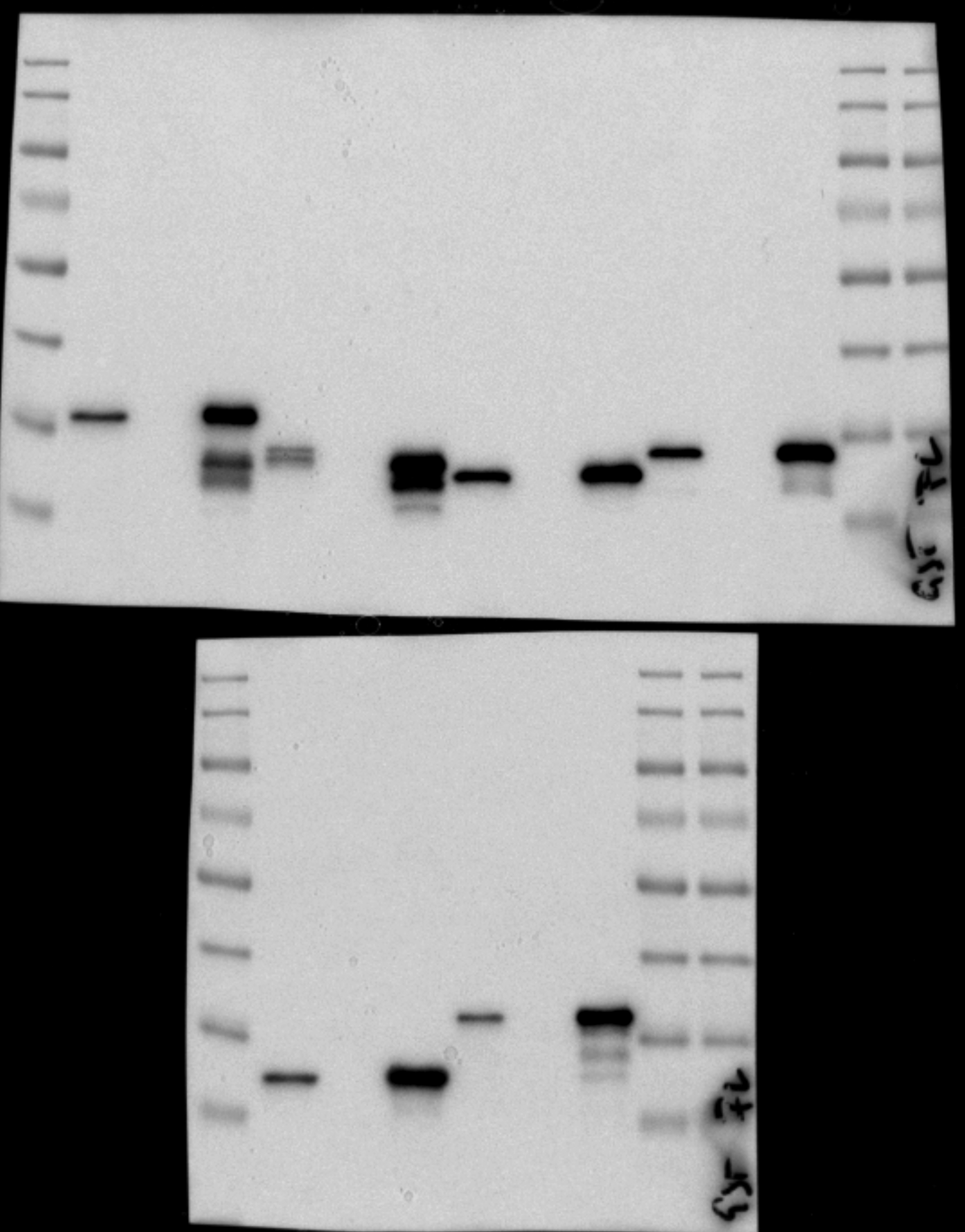

Supplement: Supplementary file 1 — Figure Source Data for EV [file 44319_2024_107_MOESM1_ESM.zip › ExpandedViewSourceData/Figure EV5/EV5C/GST WB NBS1 FL Replicate 2+Membrane.tif]

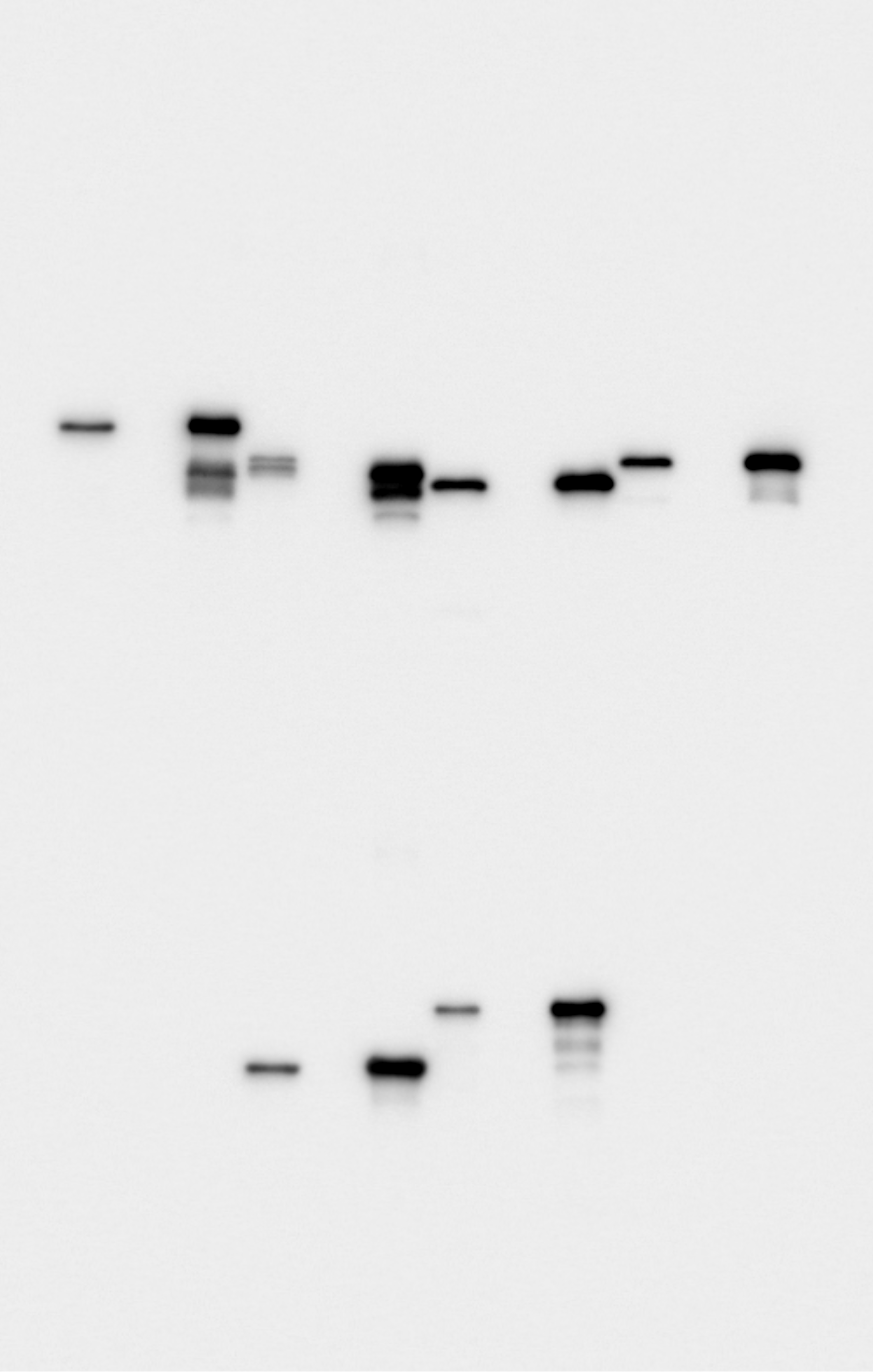

Supplement: Supplementary file 1 — Figure Source Data for EV [file 44319_2024_107_MOESM1_ESM.zip › ExpandedViewSourceData/Figure EV5/EV5C/GST WB NBS1 FL Replicate 2.tif]

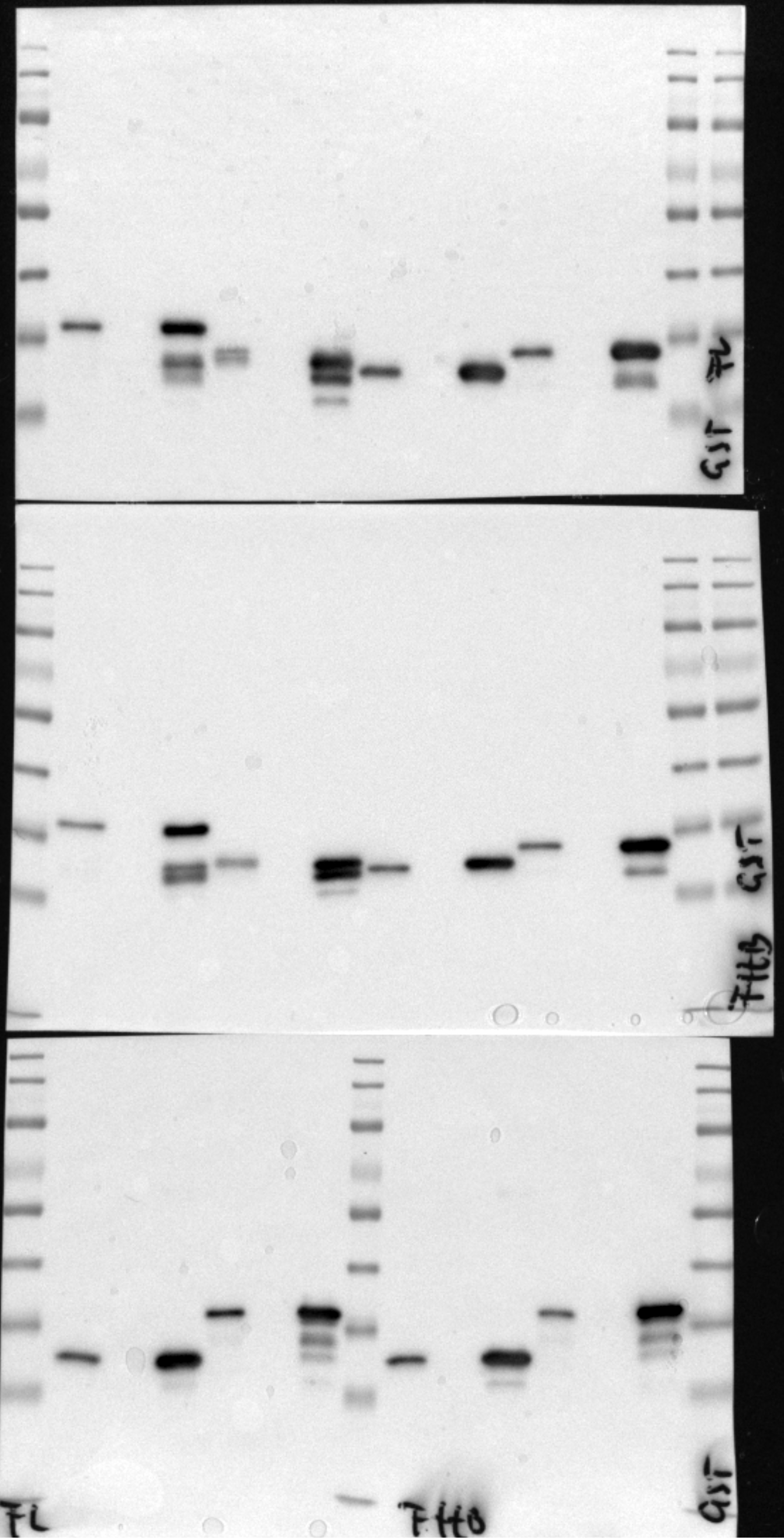

Supplement: Supplementary file 1 — Figure Source Data for EV [file 44319_2024_107_MOESM1_ESM.zip › ExpandedViewSourceData/Figure EV5/EV5C/GST WB NBS1 FL_NBS1 FHA+tBRCT Replicate 1+Membrane.tif]

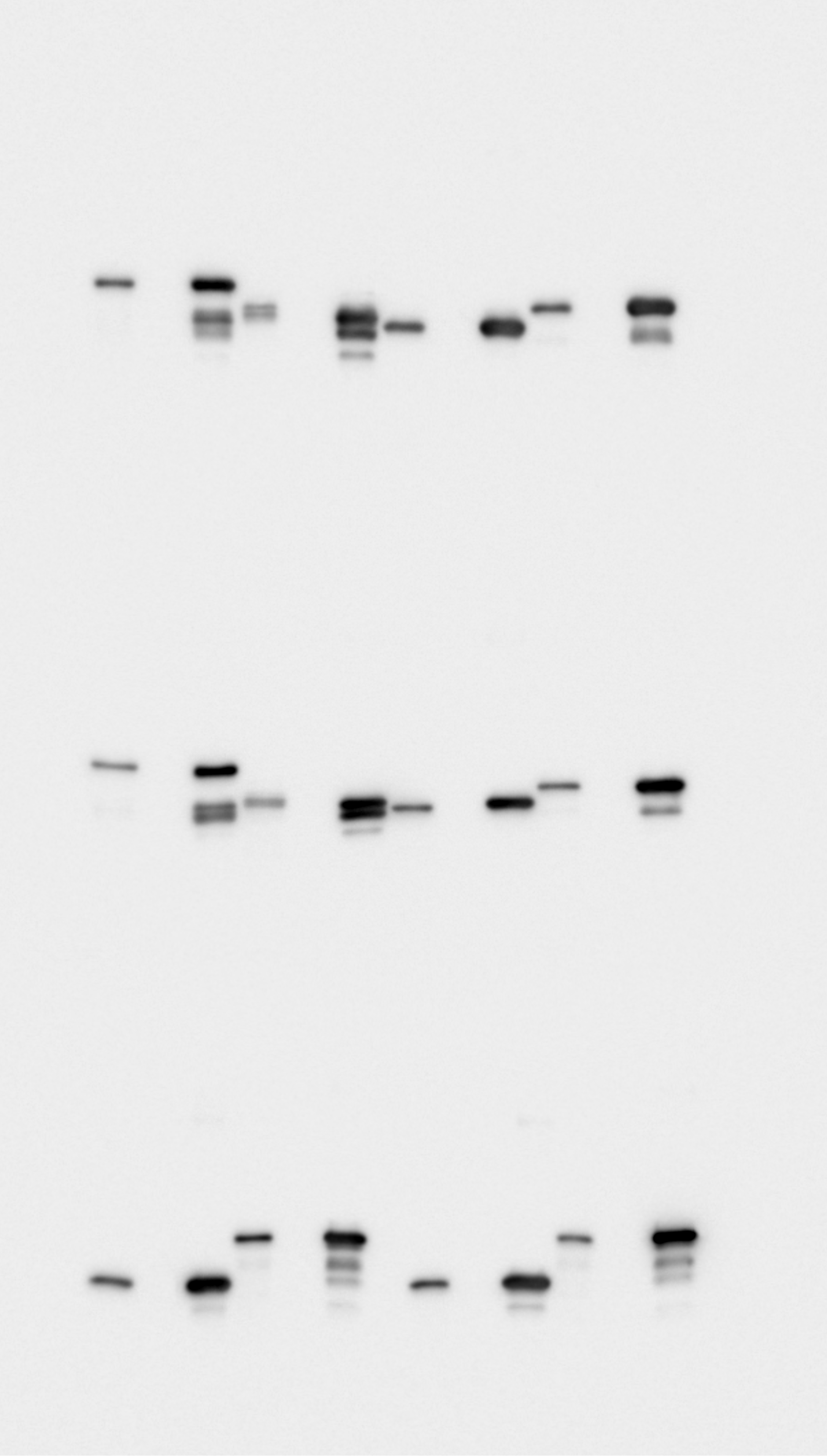

Supplement: Supplementary file 1 — Figure Source Data for EV [file 44319_2024_107_MOESM1_ESM.zip › ExpandedViewSourceData/Figure EV5/EV5C/GST WB NBS1 FL_NBS1 FHA+tBRCT Replicate 1.tif]

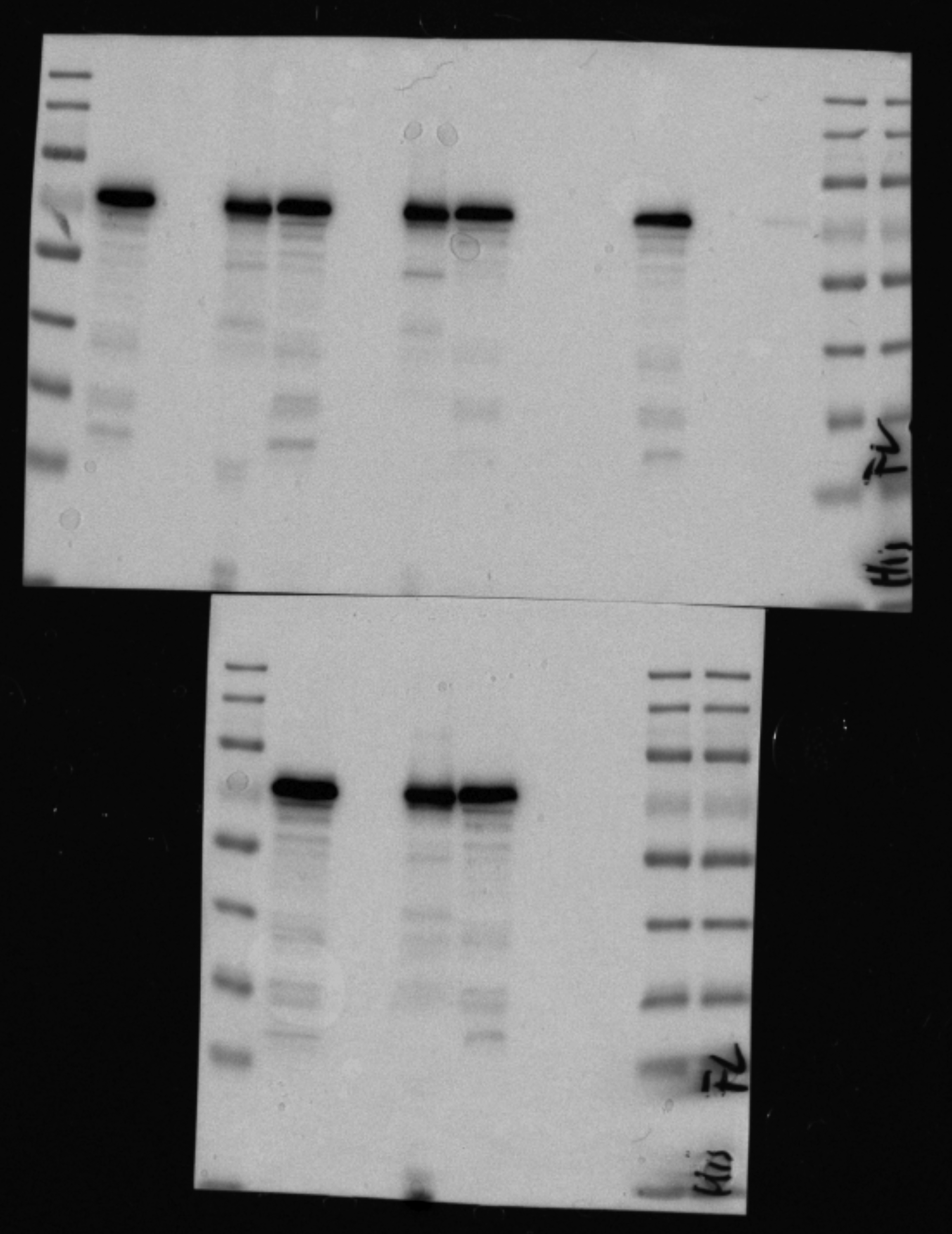

Supplement: Supplementary file 1 — Figure Source Data for EV [file 44319_2024_107_MOESM1_ESM.zip › ExpandedViewSourceData/Figure EV5/EV5C/His WB NBS1 FL_ Replicate 2+Membrane.tif]

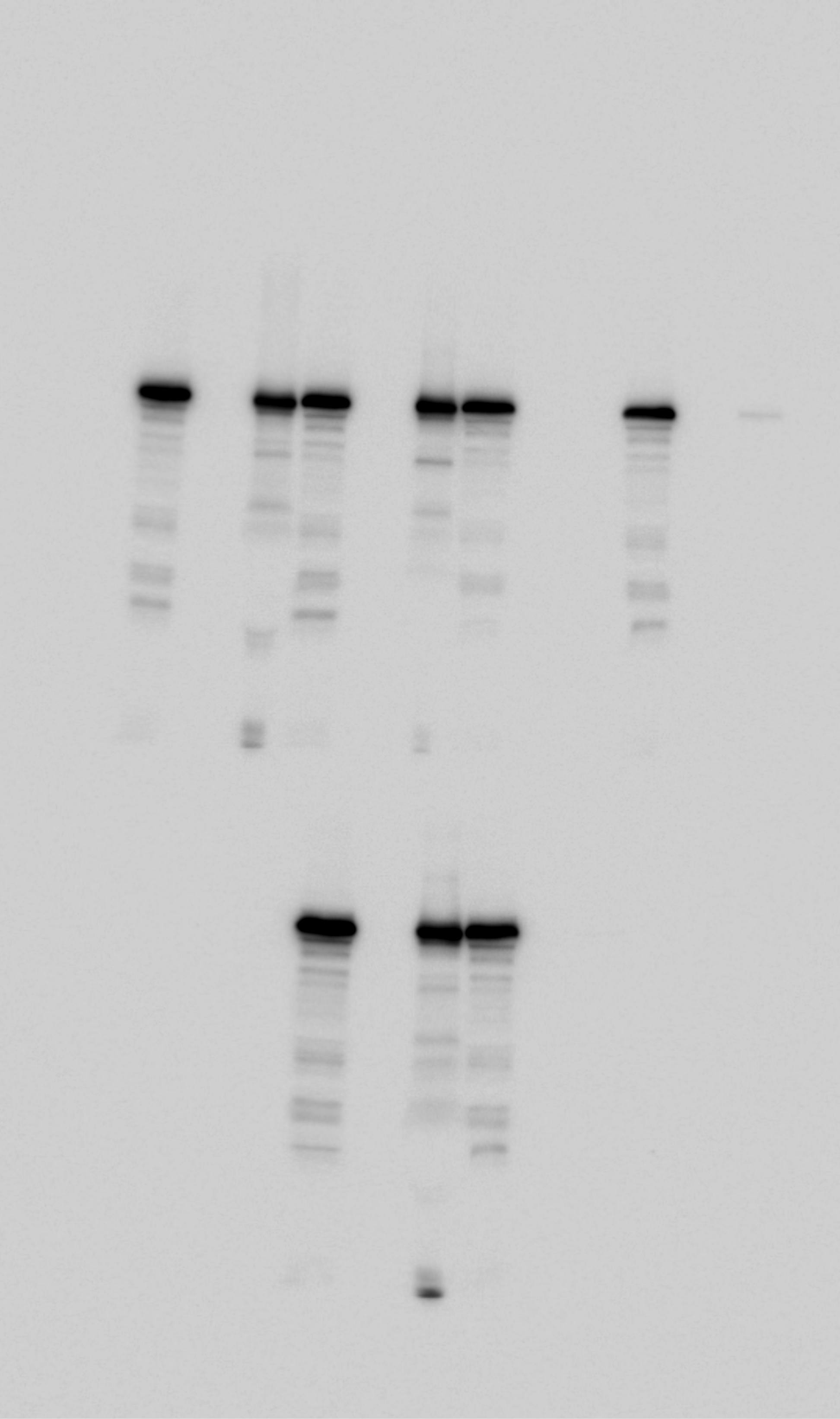

Supplement: Supplementary file 1 — Figure Source Data for EV [file 44319_2024_107_MOESM1_ESM.zip › ExpandedViewSourceData/Figure EV5/EV5C/His WB NBS1 FL_ Replicate 2.tif]

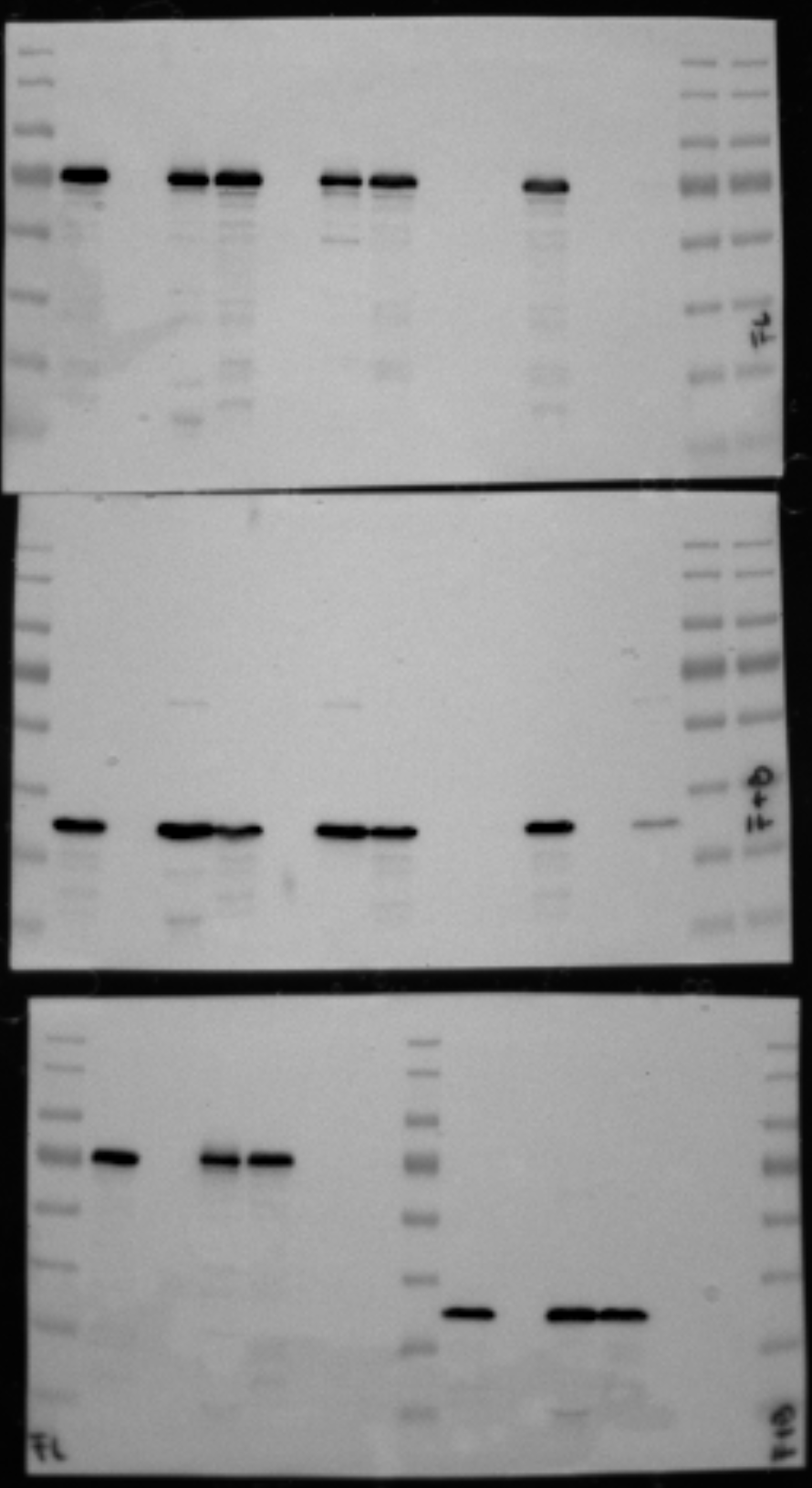

Supplement: Supplementary file 1 — Figure Source Data for EV [file 44319_2024_107_MOESM1_ESM.zip › ExpandedViewSourceData/Figure EV5/EV5C/His WB NBS1 FL_NBS1 FHA+tBRCT Replicate 1+Membrane.tif]

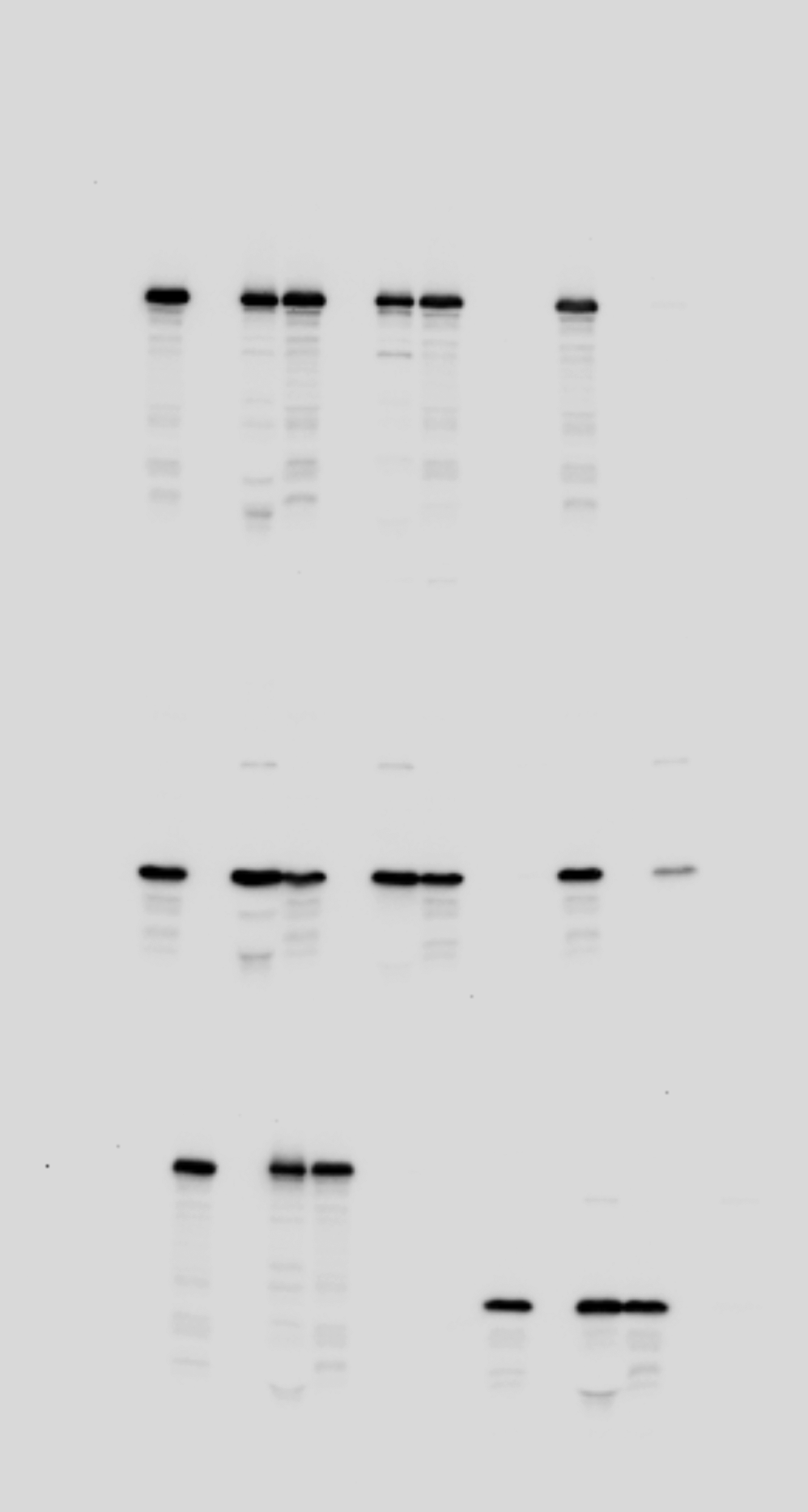

Supplement: Supplementary file 1 — Figure Source Data for EV [file 44319_2024_107_MOESM1_ESM.zip › ExpandedViewSourceData/Figure EV5/EV5C/His WB NBS1 FL_NBS1 FHA+tBRCT Replicate 1.tif]

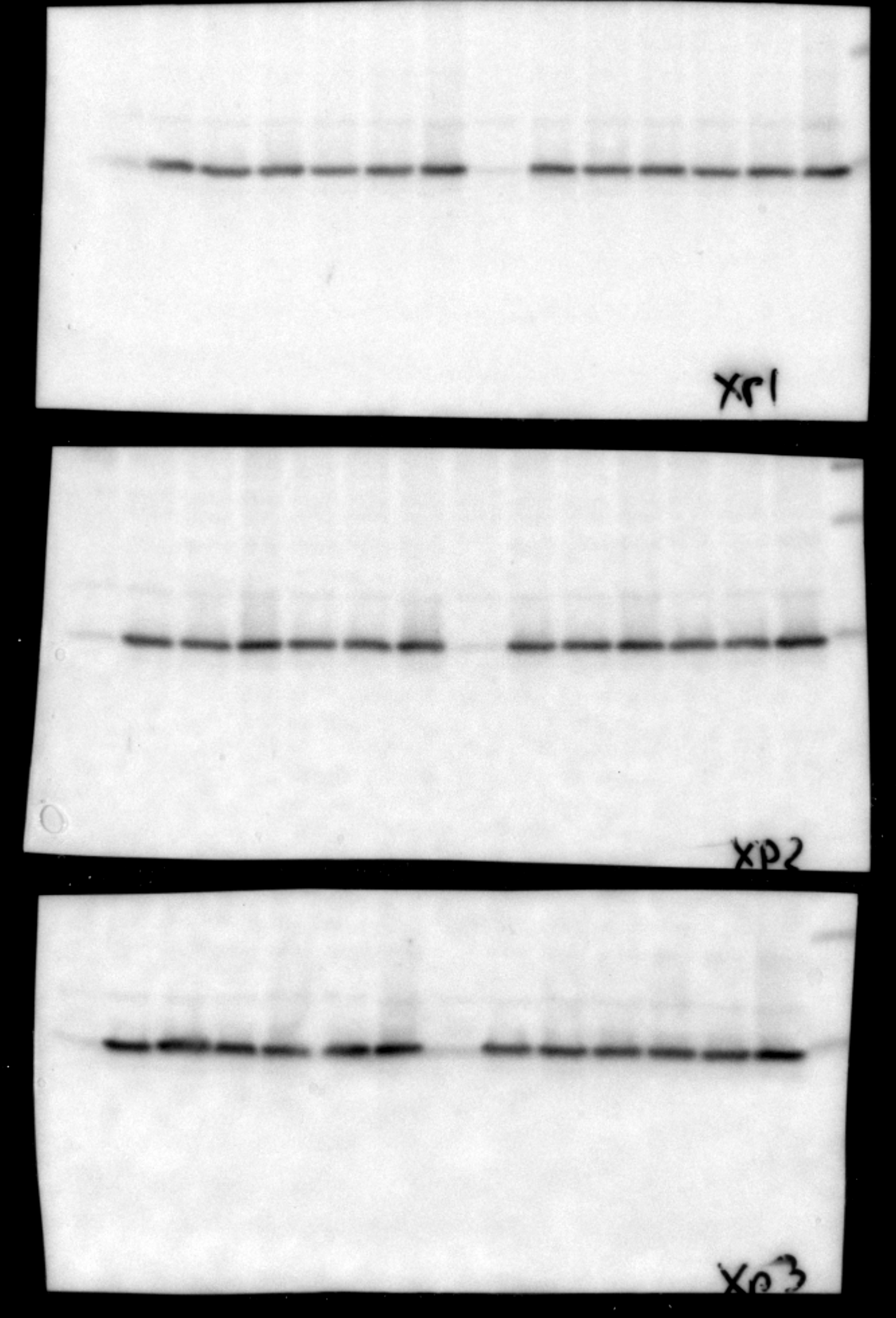

Supplement: Supplementary file 2 — Source Data Fig. 2 [file 44319_2024_107_MOESM2_ESM.zip › Figure 2/2B/gammaH2A.X WB+membrane.tif]

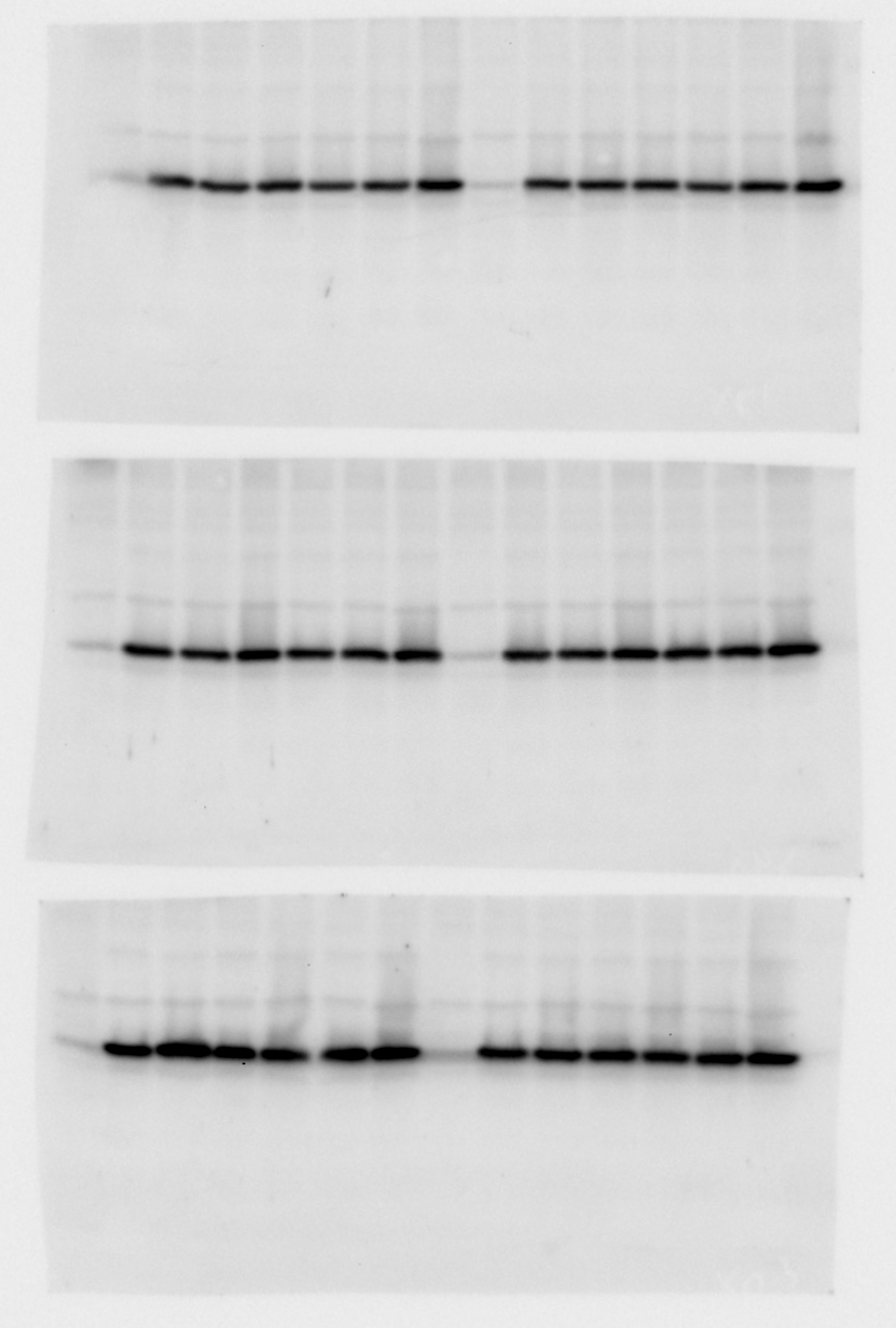

Supplement: Supplementary file 2 — Source Data Fig. 2 [file 44319_2024_107_MOESM2_ESM.zip › Figure 2/2B/gammaH2A.X WB.tif]

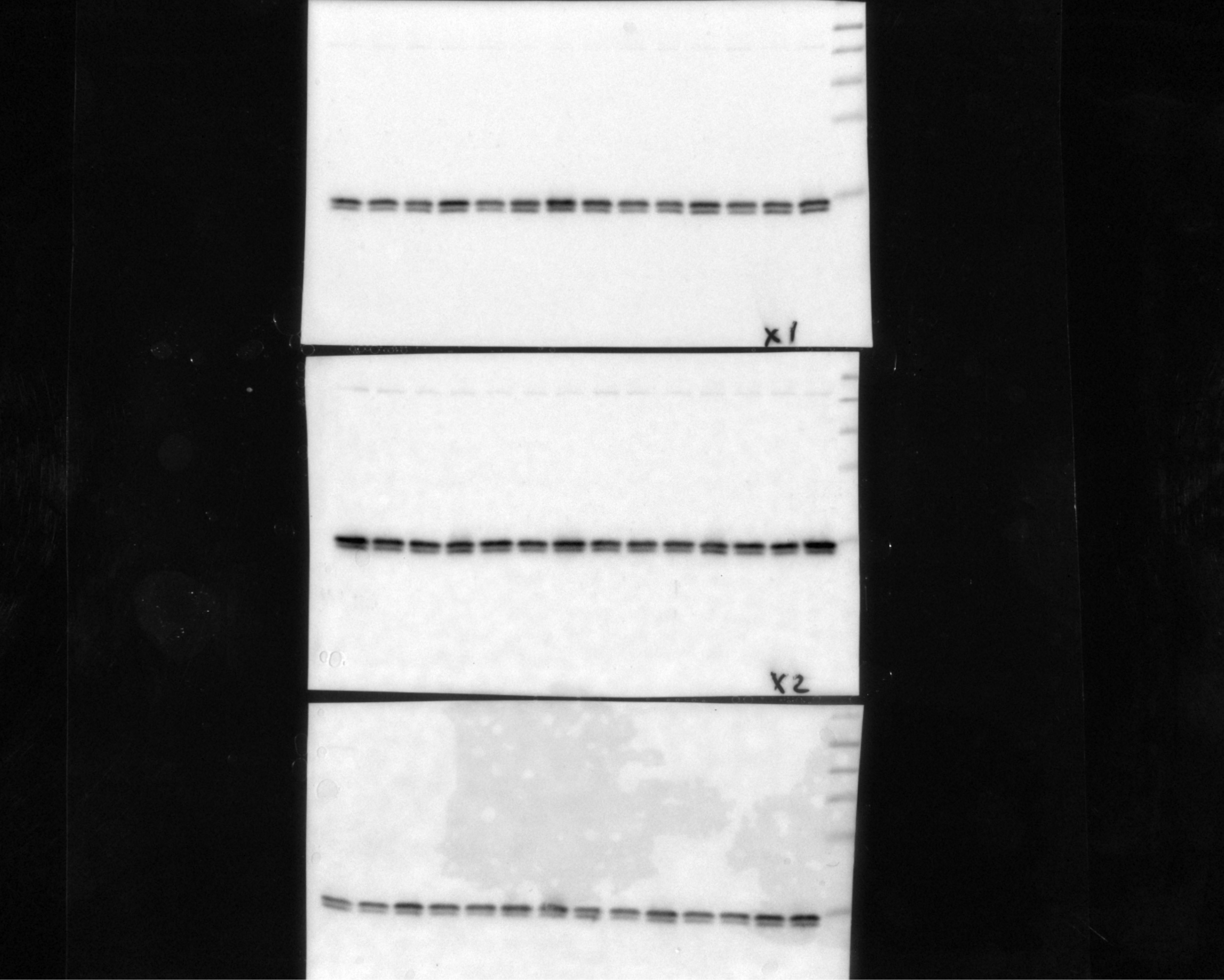

Supplement: Supplementary file 2 — Source Data Fig. 2 [file 44319_2024_107_MOESM2_ESM.zip › Figure 2/2B/H2A.X WB+membrane.tif]

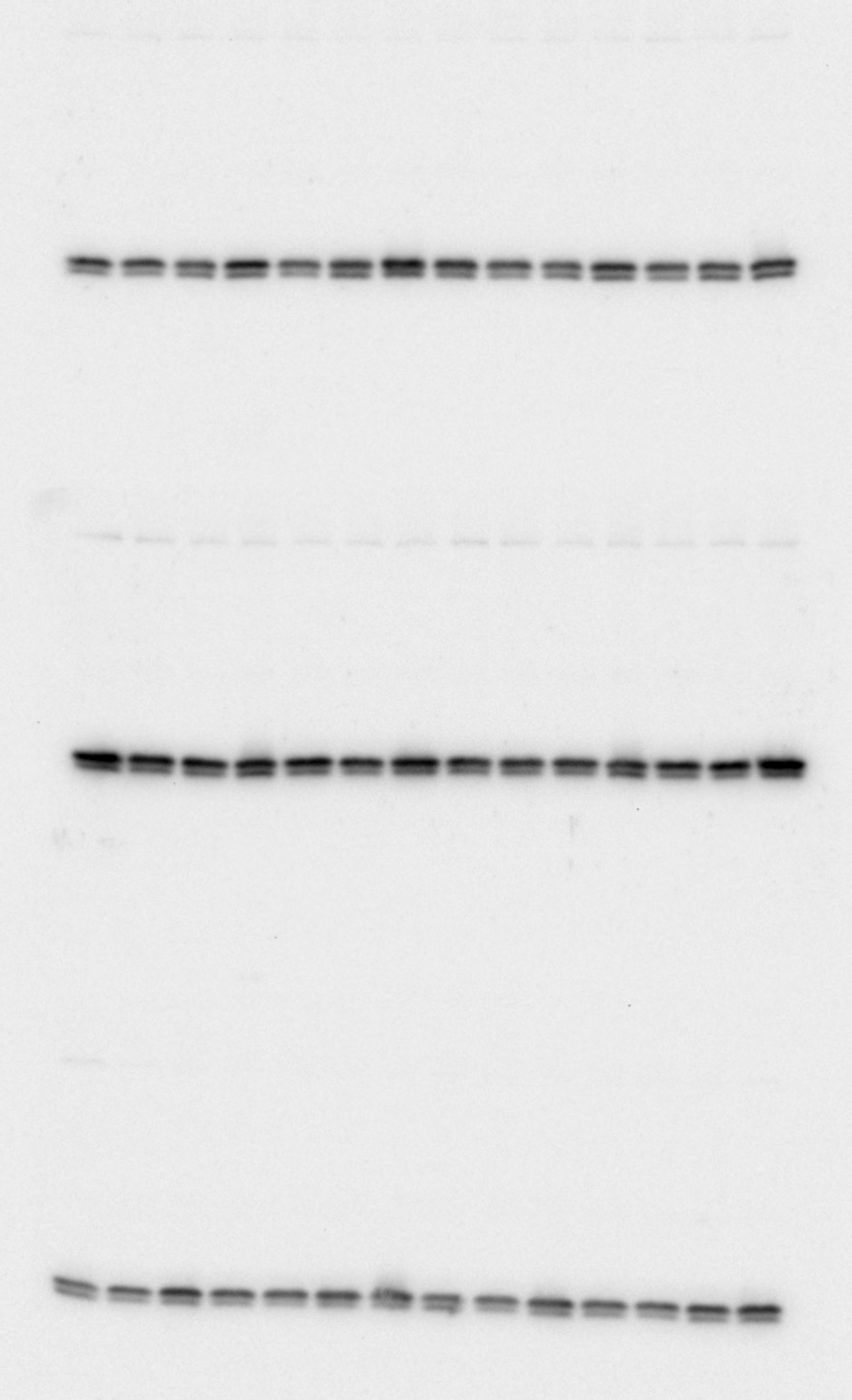

Supplement: Supplementary file 2 — Source Data Fig. 2 [file 44319_2024_107_MOESM2_ESM.zip › Figure 2/2B/H2A.X WB.tif]

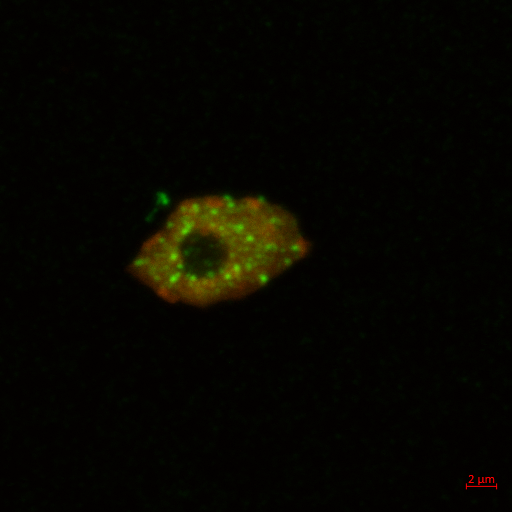

Supplement: Supplementary file 2 — Source Data Fig. 2 [file 44319_2024_107_MOESM2_ESM.zip › Figure 2/2C/bcp1-1_DAPI+gammaH2A.tif]

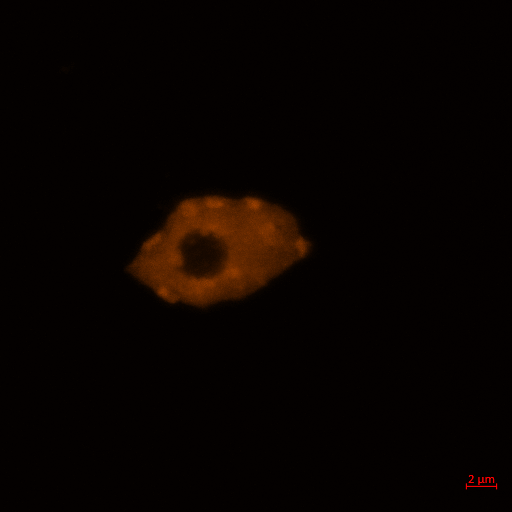

Supplement: Supplementary file 2 — Source Data Fig. 2 [file 44319_2024_107_MOESM2_ESM.zip › Figure 2/2C/bcp1-1_DAPI.tif]

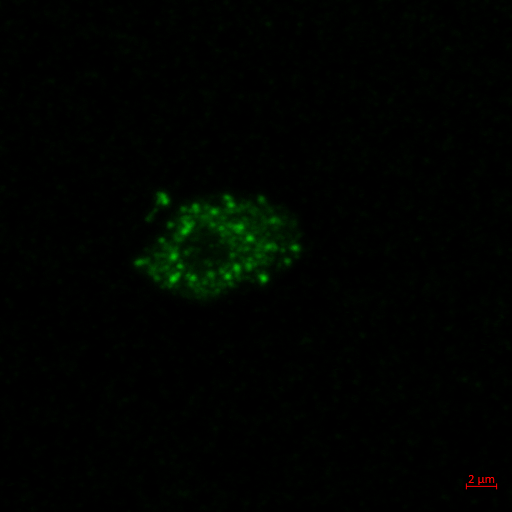

Supplement: Supplementary file 2 — Source Data Fig. 2 [file 44319_2024_107_MOESM2_ESM.zip › Figure 2/2C/bcp1-1_gammaH2A.tif]

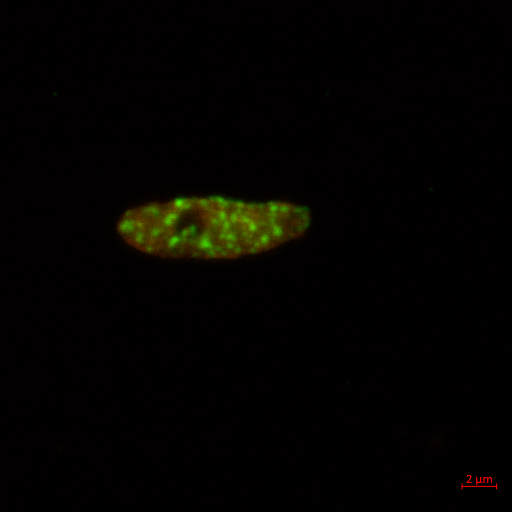

Supplement: Supplementary file 2 — Source Data Fig. 2 [file 44319_2024_107_MOESM2_ESM.zip › Figure 2/2C/bcp2-1_DAPI+gammaH2A.tif]

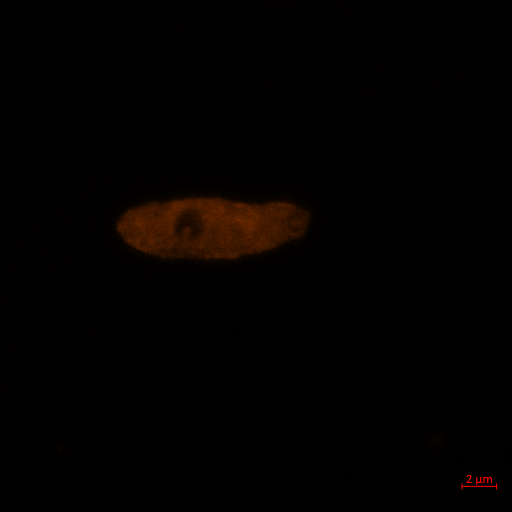

Supplement: Supplementary file 2 — Source Data Fig. 2 [file 44319_2024_107_MOESM2_ESM.zip › Figure 2/2C/bcp2-1_DAPI.tif]

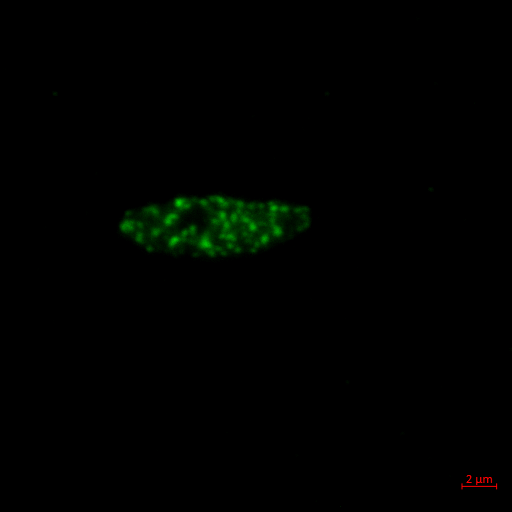

Supplement: Supplementary file 2 — Source Data Fig. 2 [file 44319_2024_107_MOESM2_ESM.zip › Figure 2/2C/bcp2-1_gammaH2A.tif]

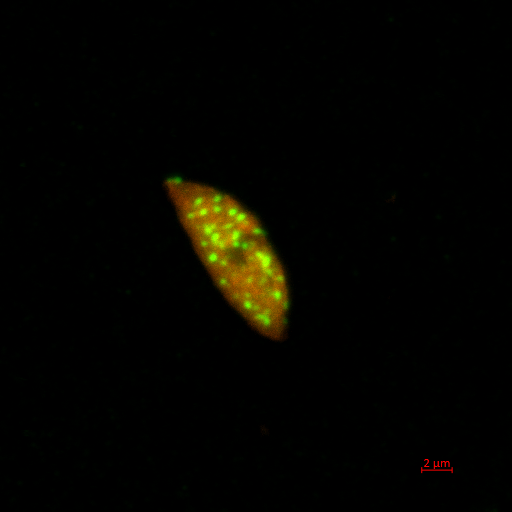

Supplement: Supplementary file 2 — Source Data Fig. 2 [file 44319_2024_107_MOESM2_ESM.zip › Figure 2/2C/bcp3-1_DAPI+gammaX.tif]

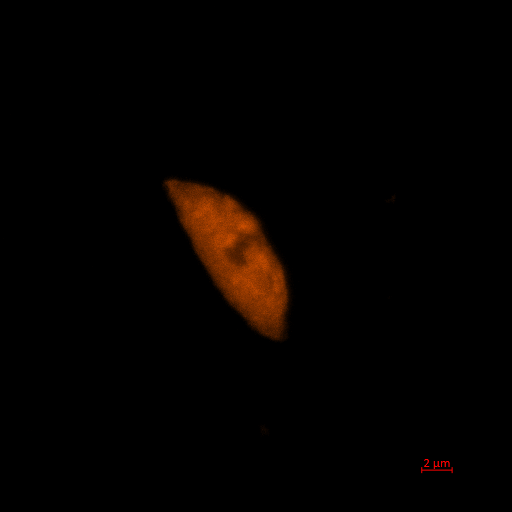

Supplement: Supplementary file 2 — Source Data Fig. 2 [file 44319_2024_107_MOESM2_ESM.zip › Figure 2/2C/bcp3-1_DAPI.tif]

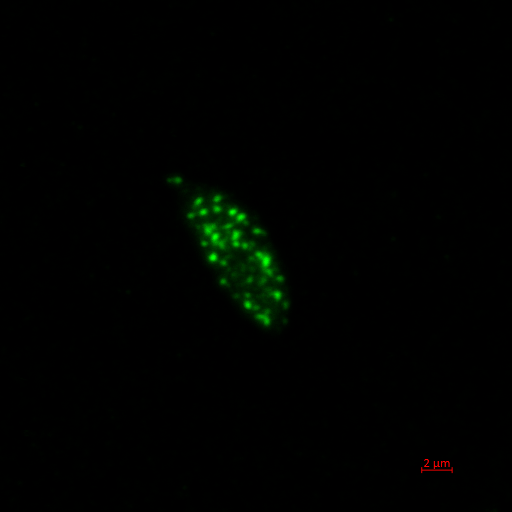

Supplement: Supplementary file 2 — Source Data Fig. 2 [file 44319_2024_107_MOESM2_ESM.zip › Figure 2/2C/bcp3-1_gammaH2A.tif]

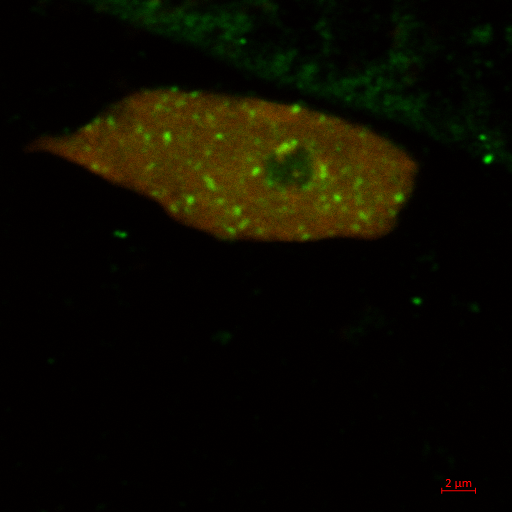

Supplement: Supplementary file 2 — Source Data Fig. 2 [file 44319_2024_107_MOESM2_ESM.zip › Figure 2/2C/bcp4-2_DAPI+gammaX.tif]

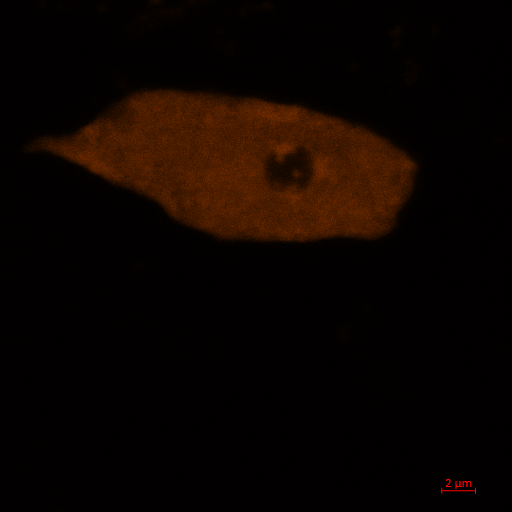

Supplement: Supplementary file 2 — Source Data Fig. 2 [file 44319_2024_107_MOESM2_ESM.zip › Figure 2/2C/bcp4-2_DAPI.tif]

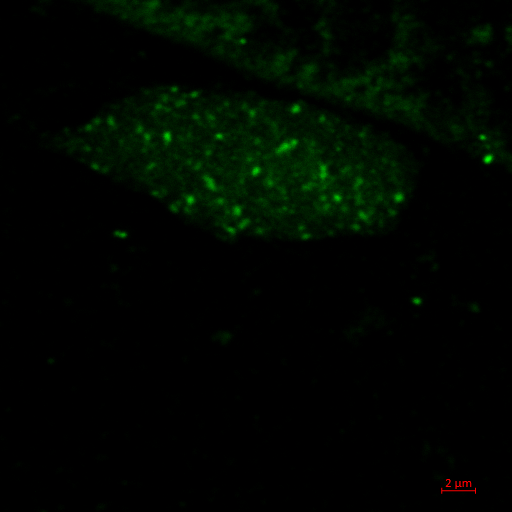

Supplement: Supplementary file 2 — Source Data Fig. 2 [file 44319_2024_107_MOESM2_ESM.zip › Figure 2/2C/bcp4-2_gammaX.tif]

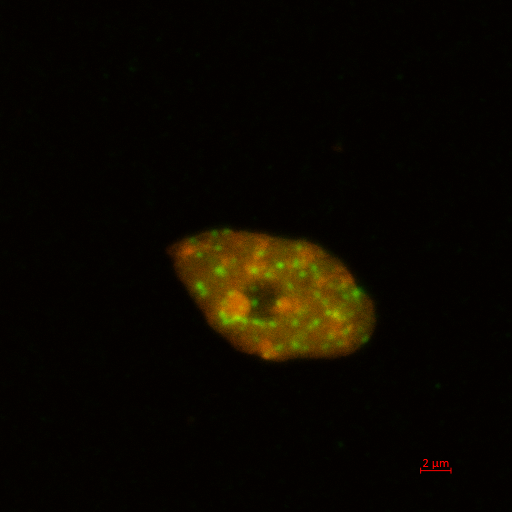

Supplement: Supplementary file 2 — Source Data Fig. 2 [file 44319_2024_107_MOESM2_ESM.zip › Figure 2/2C/WT_DAPI+gammaH2A.tif]

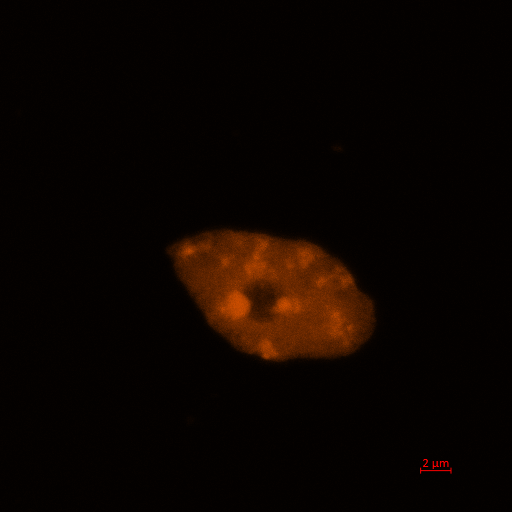

Supplement: Supplementary file 2 — Source Data Fig. 2 [file 44319_2024_107_MOESM2_ESM.zip › Figure 2/2C/WT_DAPI.tif]

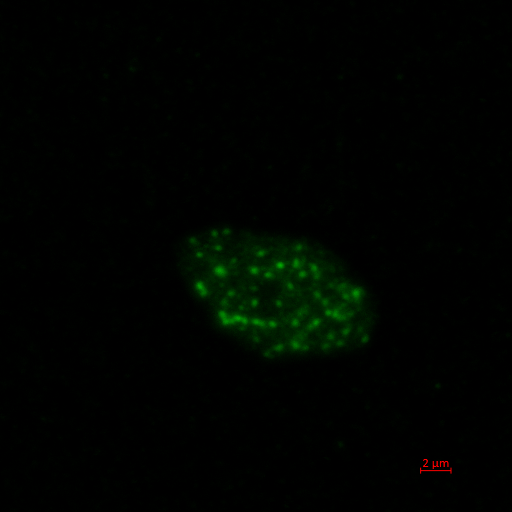

Supplement: Supplementary file 2 — Source Data Fig. 2 [file 44319_2024_107_MOESM2_ESM.zip › Figure 2/2C/WT_gammaH2A.tif]

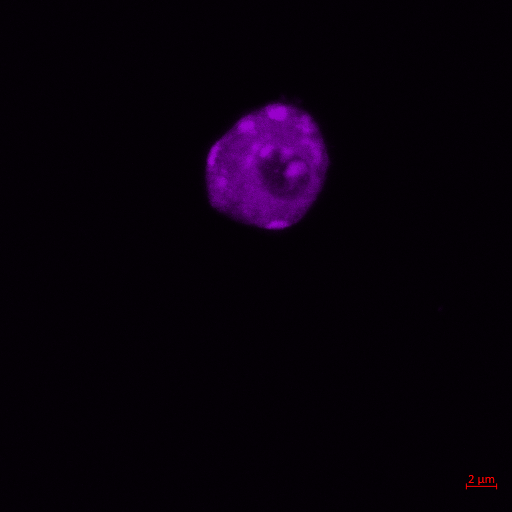

Supplement: Supplementary file 3 — Source Data Fig. 3 [file 44319_2024_107_MOESM3_ESM.zip › Figure 3/3A/BCP1-DAPI.tif]

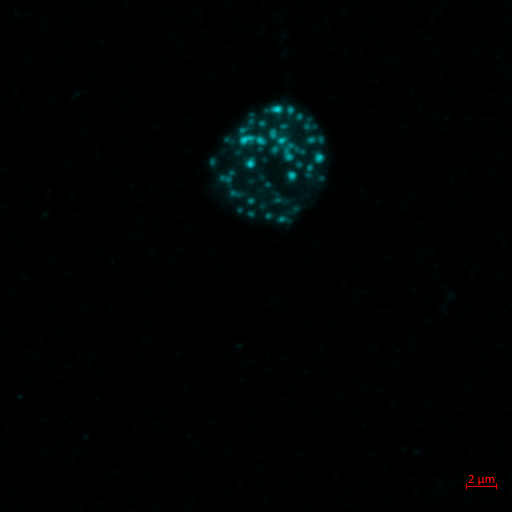

Supplement: Supplementary file 3 — Source Data Fig. 3 [file 44319_2024_107_MOESM3_ESM.zip › Figure 3/3A/BCP1-gammaH2A.X.tif]

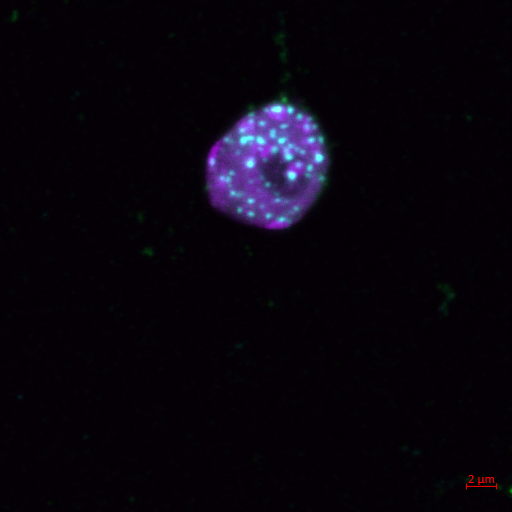

Supplement: Supplementary file 3 — Source Data Fig. 3 [file 44319_2024_107_MOESM3_ESM.zip › Figure 3/3A/BCP1-mClover-gammaH2A.X-DAPI.tif]

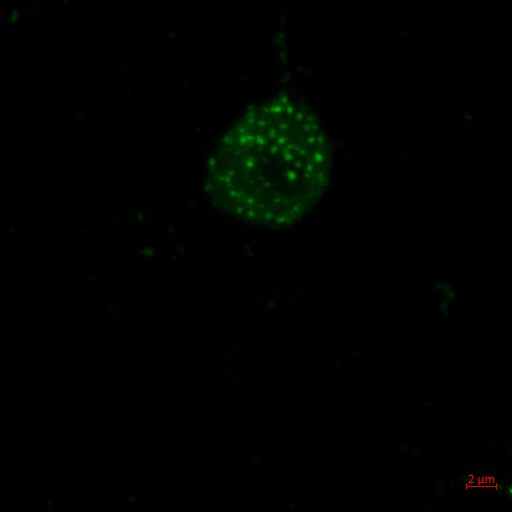

Supplement: Supplementary file 3 — Source Data Fig. 3 [file 44319_2024_107_MOESM3_ESM.zip › Figure 3/3A/BCP1-mClover.tif]

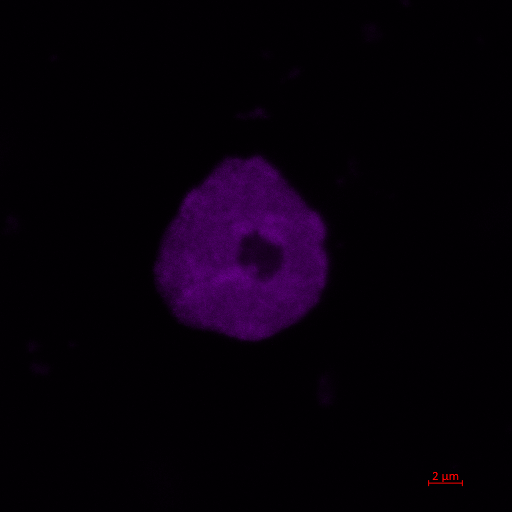

Supplement: Supplementary file 3 — Source Data Fig. 3 [file 44319_2024_107_MOESM3_ESM.zip › Figure 3/3A/BCP4-DAPI.tif]

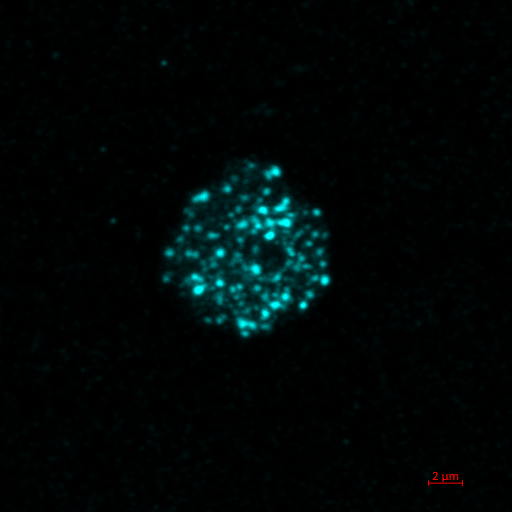

Supplement: Supplementary file 3 — Source Data Fig. 3 [file 44319_2024_107_MOESM3_ESM.zip › Figure 3/3A/BCP4-gammaH2A.X.tif]

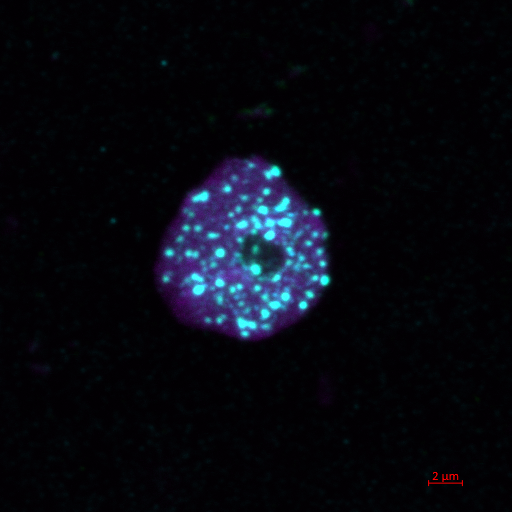

Supplement: Supplementary file 3 — Source Data Fig. 3 [file 44319_2024_107_MOESM3_ESM.zip › Figure 3/3A/BCP4-mClover-gammaH2A.X-DAPI.tif]

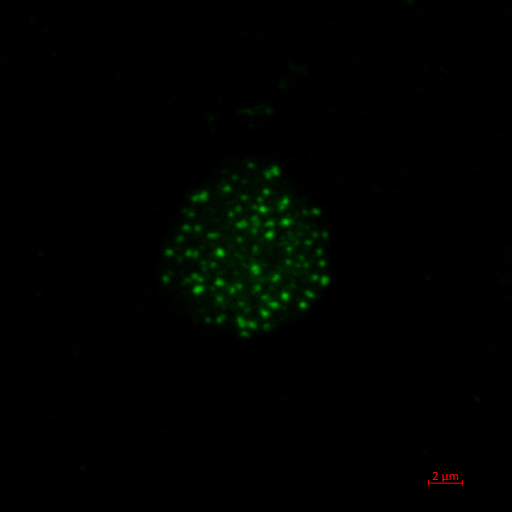

Supplement: Supplementary file 3 — Source Data Fig. 3 [file 44319_2024_107_MOESM3_ESM.zip › Figure 3/3A/BCP4-mClover.tif]

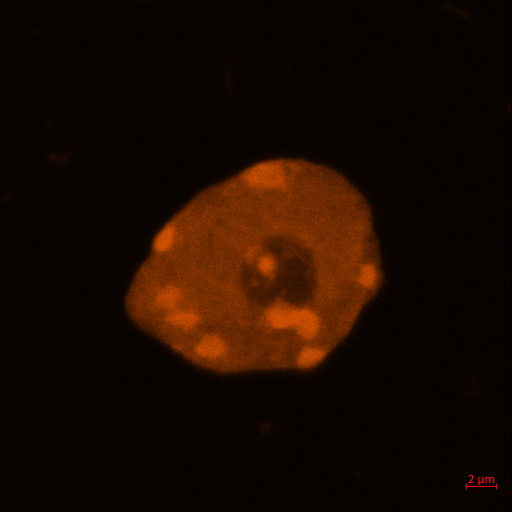

Supplement: Supplementary file 3 — Source Data Fig. 3 [file 44319_2024_107_MOESM3_ESM.zip › Figure 3/3B/BCP1-DAPI1.tif]

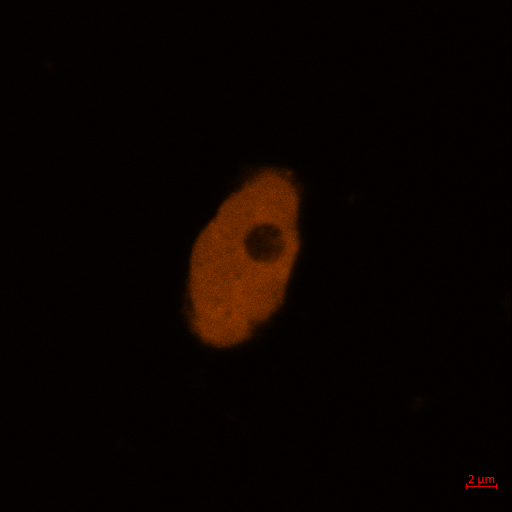

Supplement: Supplementary file 3 — Source Data Fig. 3 [file 44319_2024_107_MOESM3_ESM.zip › Figure 3/3B/BCP1-DAPI2.tif]

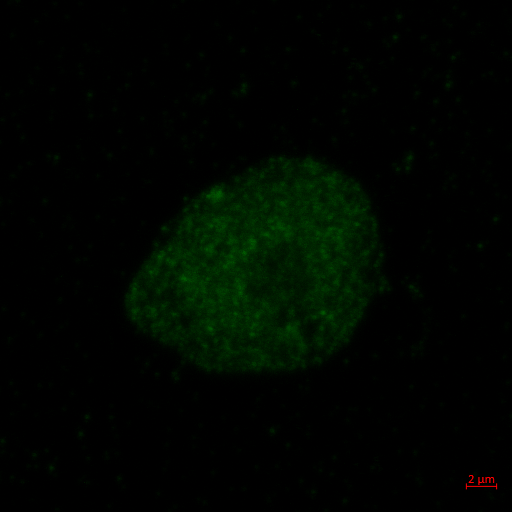

Supplement: Supplementary file 3 — Source Data Fig. 3 [file 44319_2024_107_MOESM3_ESM.zip › Figure 3/3B/BCP1-mClover1.tif]

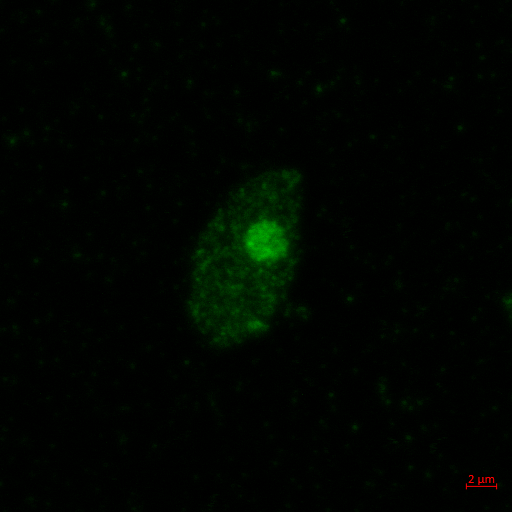

Supplement: Supplementary file 3 — Source Data Fig. 3 [file 44319_2024_107_MOESM3_ESM.zip › Figure 3/3B/BCP1-mClover2.tif]

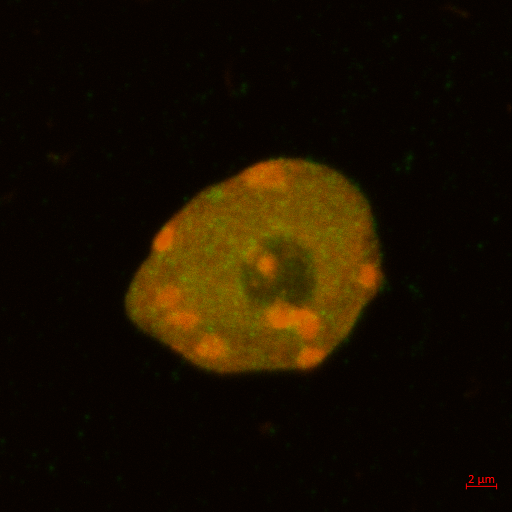

Supplement: Supplementary file 3 — Source Data Fig. 3 [file 44319_2024_107_MOESM3_ESM.zip › Figure 3/3B/DAPI+BCP1-mClover1.tif]

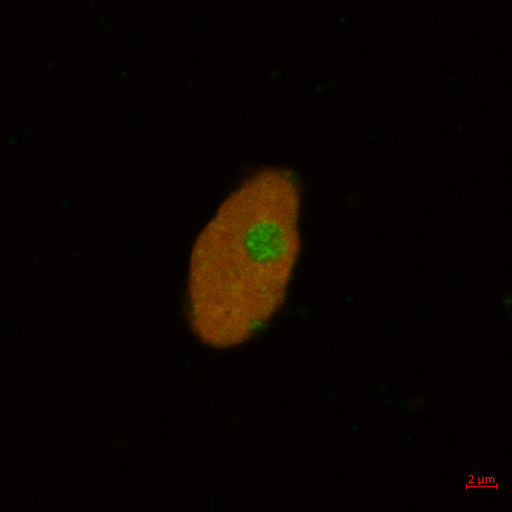

Supplement: Supplementary file 3 — Source Data Fig. 3 [file 44319_2024_107_MOESM3_ESM.zip › Figure 3/3B/DAPI+BCP1-mClover2.tif]

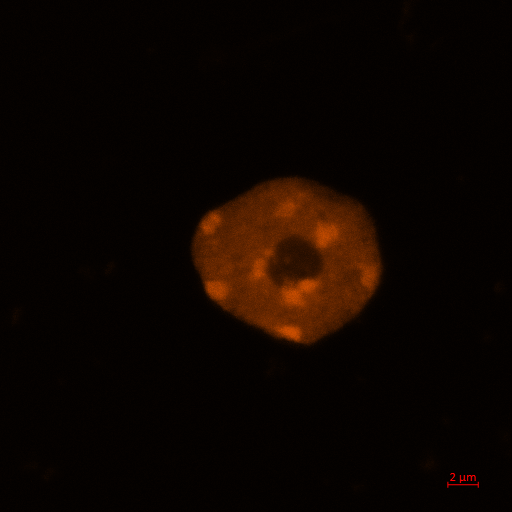

Supplement: Supplementary file 3 — Source Data Fig. 3 [file 44319_2024_107_MOESM3_ESM.zip › Figure 3/3C/BCP4-DAPI1.tif]

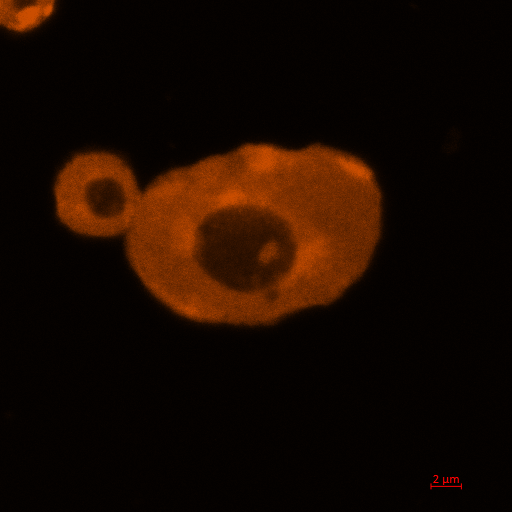

Supplement: Supplementary file 3 — Source Data Fig. 3 [file 44319_2024_107_MOESM3_ESM.zip › Figure 3/3C/BCP4-DAPI2.tif]

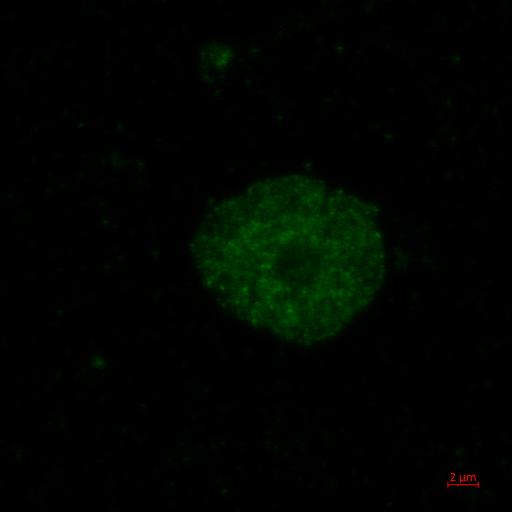

Supplement: Supplementary file 3 — Source Data Fig. 3 [file 44319_2024_107_MOESM3_ESM.zip › Figure 3/3C/BCP4-mClover1.tif]

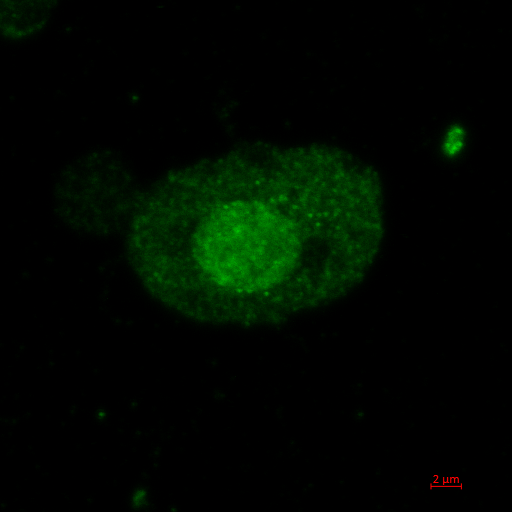

Supplement: Supplementary file 3 — Source Data Fig. 3 [file 44319_2024_107_MOESM3_ESM.zip › Figure 3/3C/BCP4-mClover2.tif]

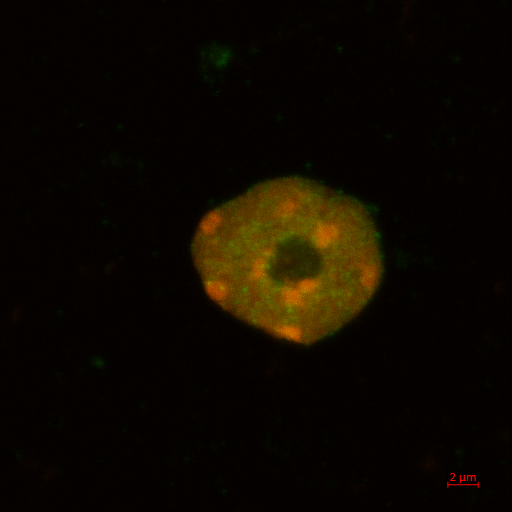

Supplement: Supplementary file 3 — Source Data Fig. 3 [file 44319_2024_107_MOESM3_ESM.zip › Figure 3/3C/DAPI+BCP4-mClover1.tif]

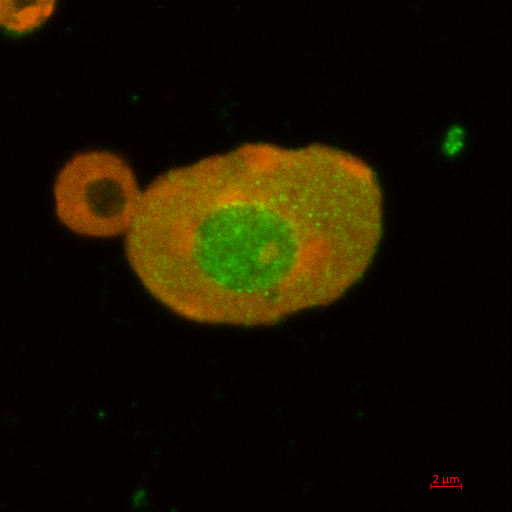

Supplement: Supplementary file 3 — Source Data Fig. 3 [file 44319_2024_107_MOESM3_ESM.zip › Figure 3/3C/DAPI+BCP4-mClover2.tif]

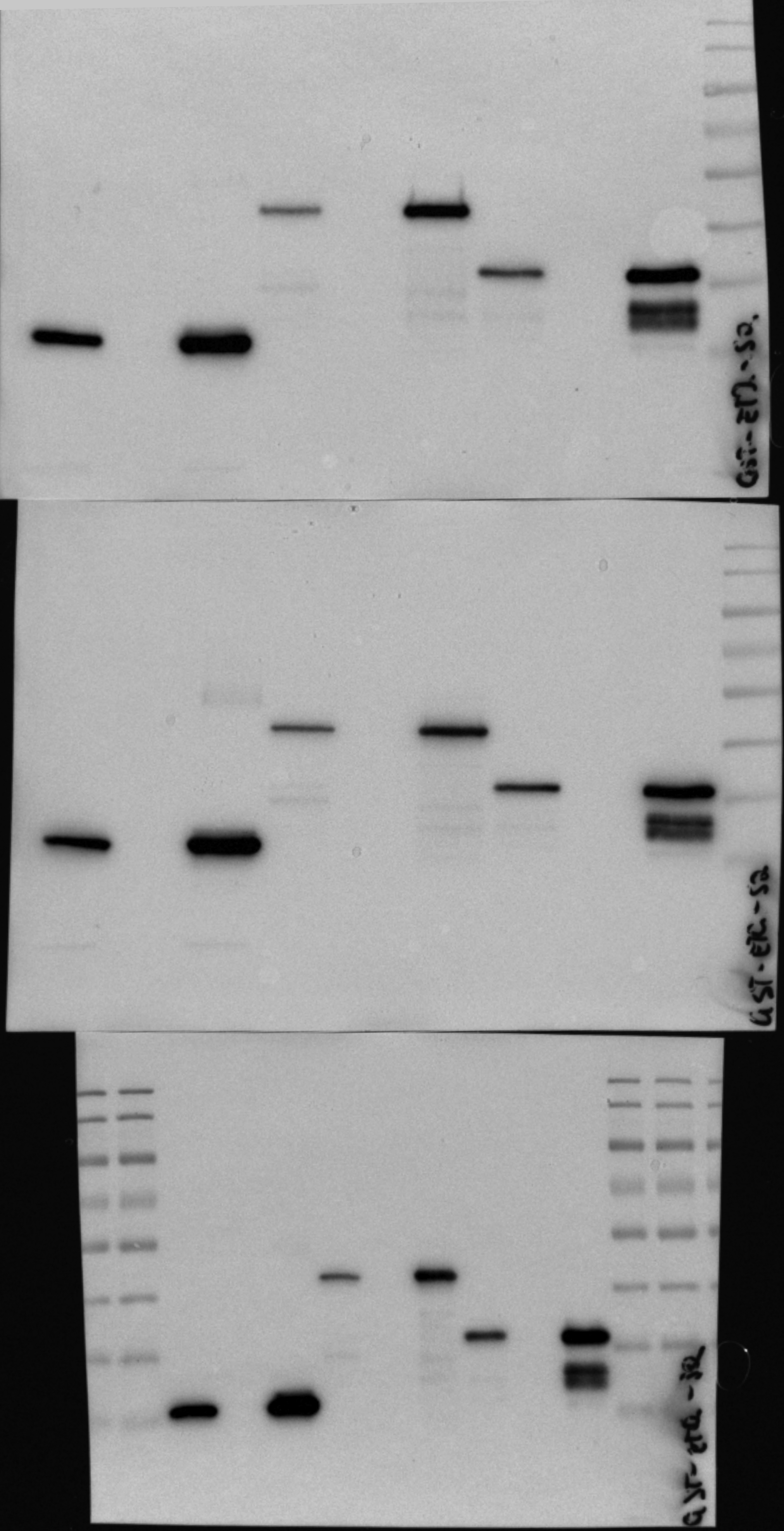

Supplement: Supplementary file 6 — Source Data Fig. 6 [file 44319_2024_107_MOESM6_ESM.zip › Figure 6/6B/GST WB NBS1 FL Replicate 1_NBS1 FHA+tBRCT Replicate 1 and 2+Membrane.tif]

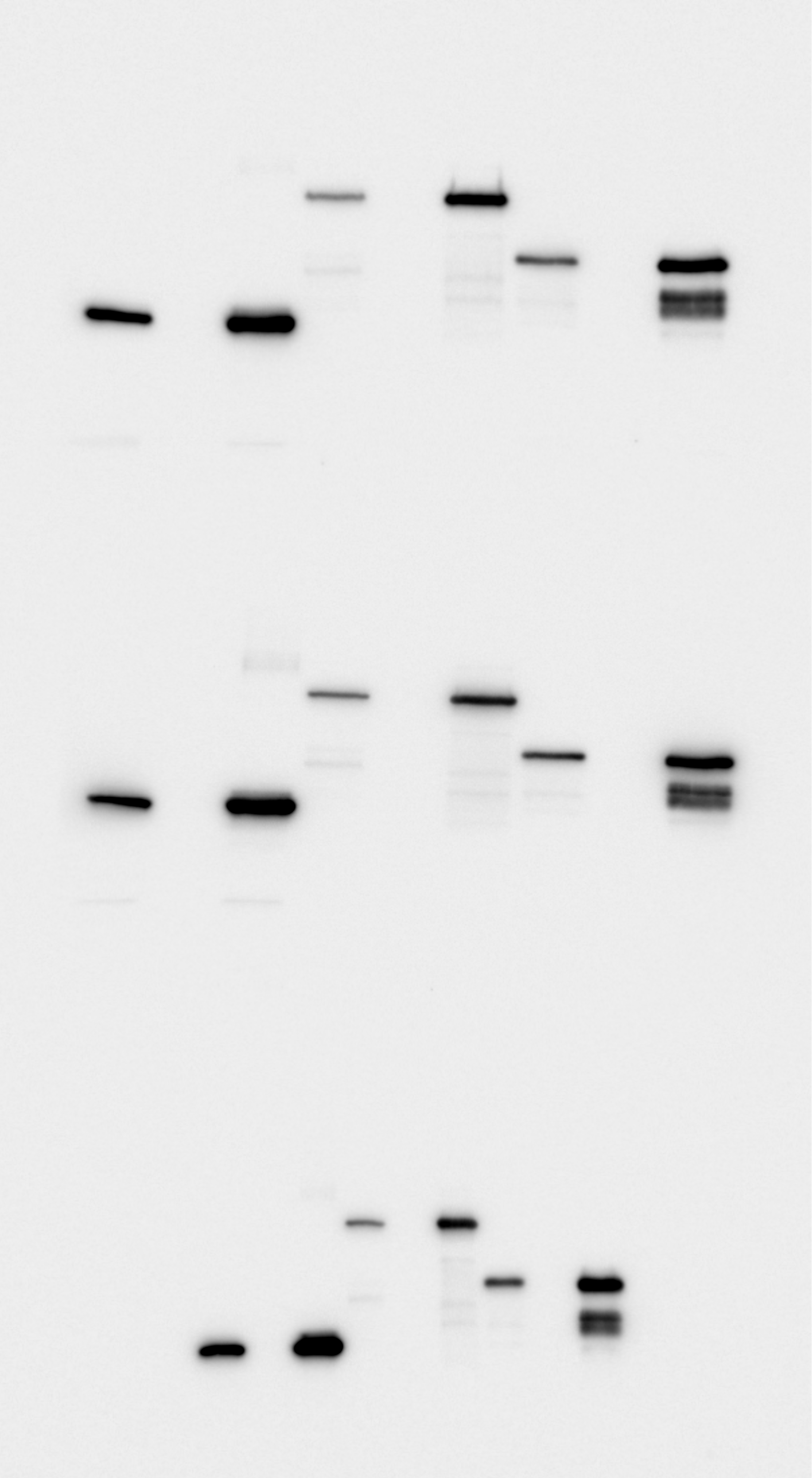

Supplement: Supplementary file 6 — Source Data Fig. 6 [file 44319_2024_107_MOESM6_ESM.zip › Figure 6/6B/GST WB NBS1 FL Replicate 1_NBS1 FHA+tBRCT Replicate 1 and 2.tif]

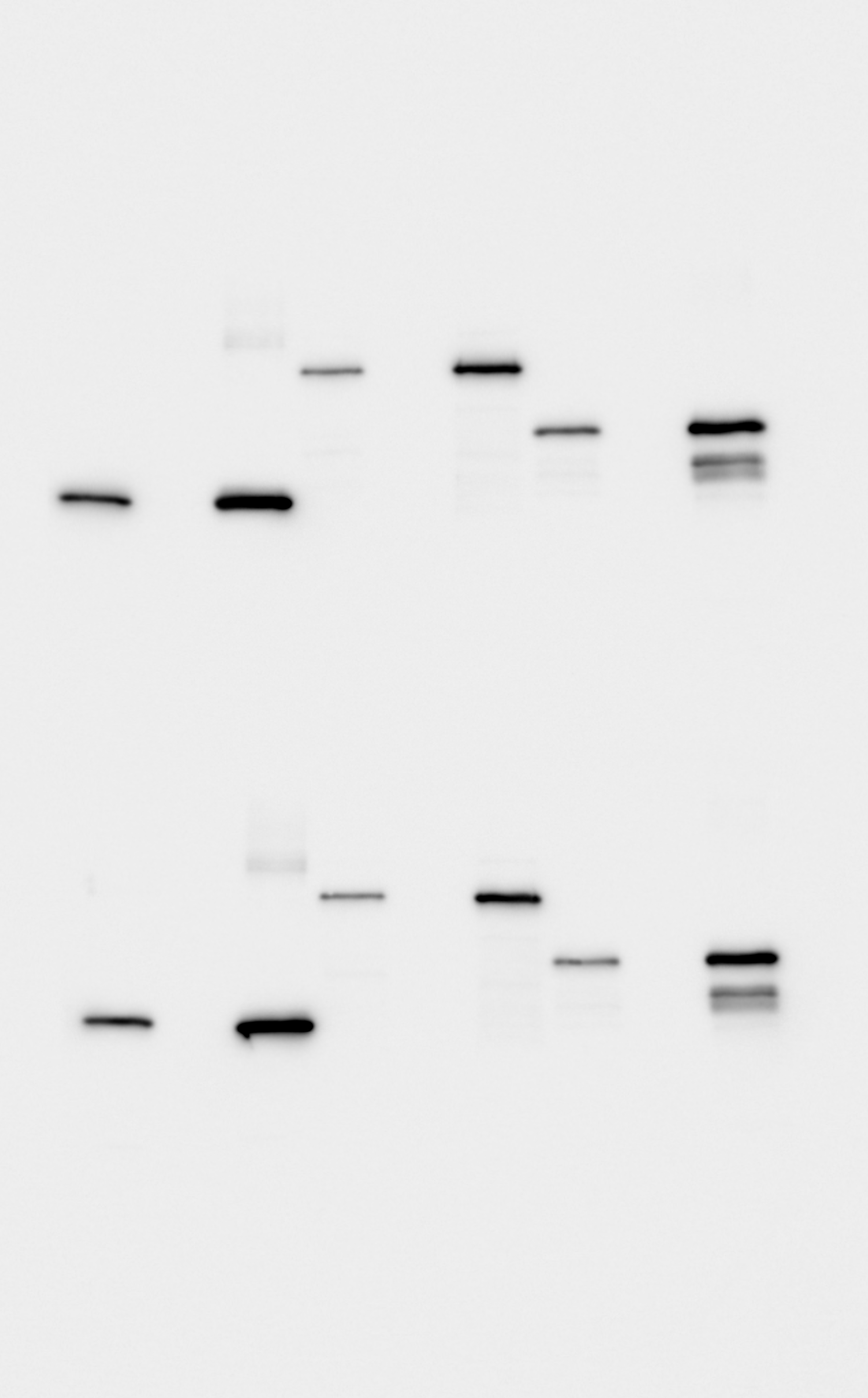

Supplement: Supplementary file 6 — Source Data Fig. 6 [file 44319_2024_107_MOESM6_ESM.zip › Figure 6/6B/GST WB NBS1 FL Replicate 2 and 3.tif]

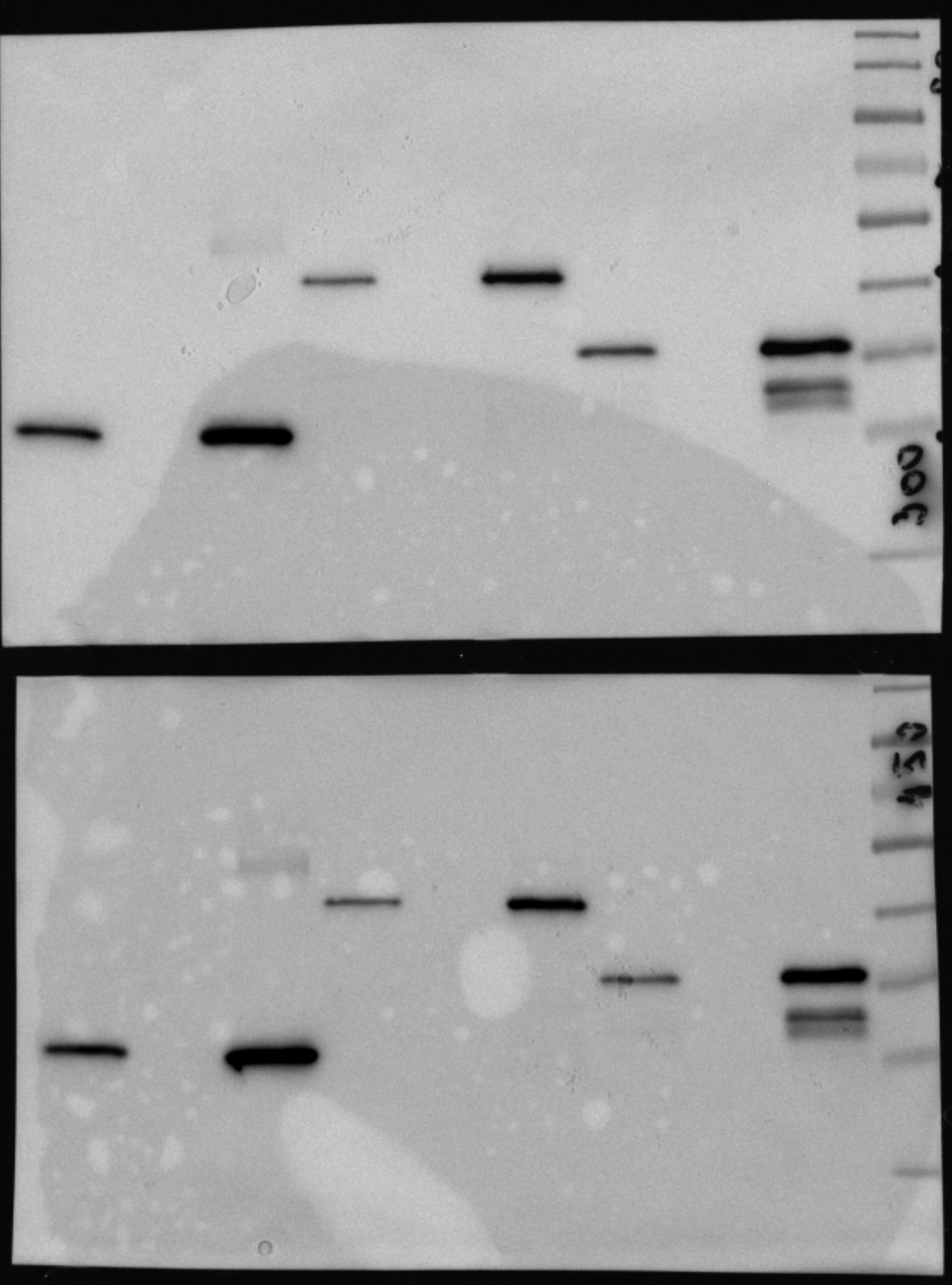

Supplement: Supplementary file 6 — Source Data Fig. 6 [file 44319_2024_107_MOESM6_ESM.zip › Figure 6/6B/GST WB NBS1 FL Replicate 3 and 2+Membrane.tif]

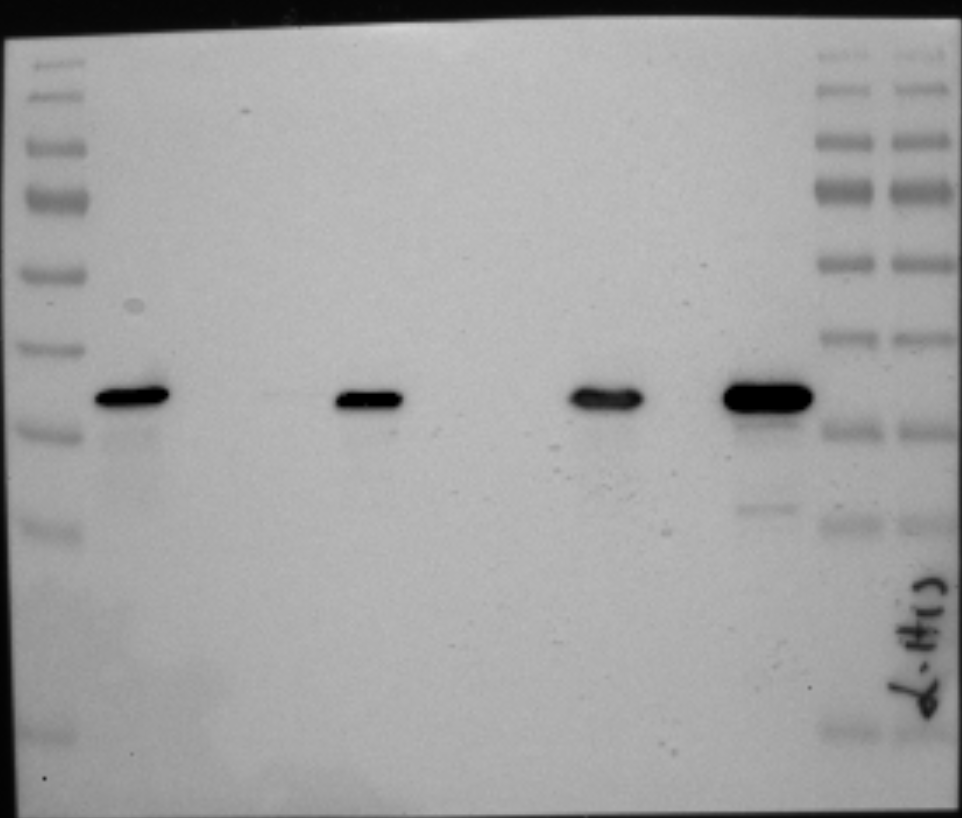

Supplement: Supplementary file 6 — Source Data Fig. 6 [file 44319_2024_107_MOESM6_ESM.zip › Figure 6/6B/His WB NBS1 FHA+tBRCT Replicate 1+Membrane.tif]

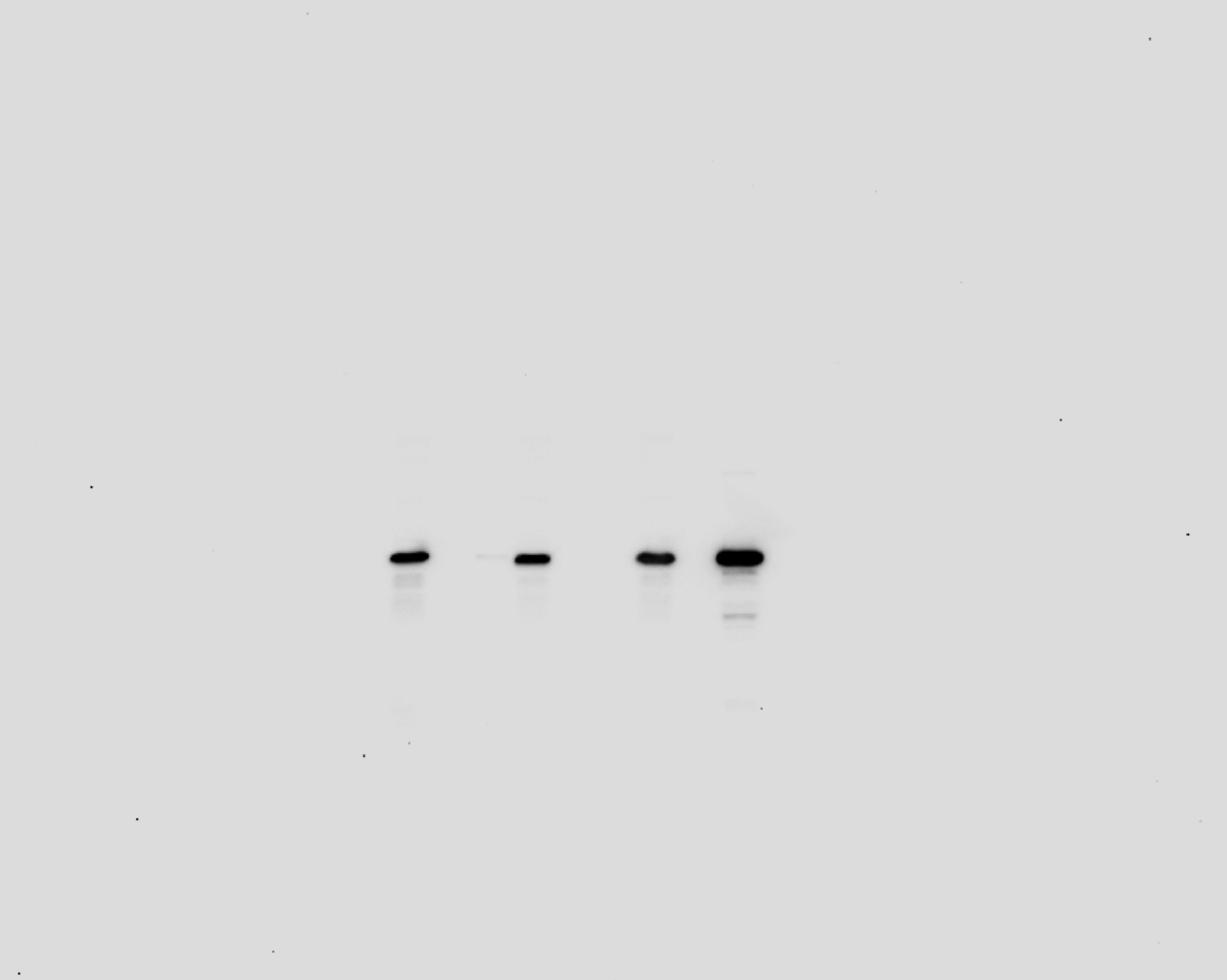

Supplement: Supplementary file 6 — Source Data Fig. 6 [file 44319_2024_107_MOESM6_ESM.zip › Figure 6/6B/His WB NBS1 FHA+tBRCT replicate 1.tif]

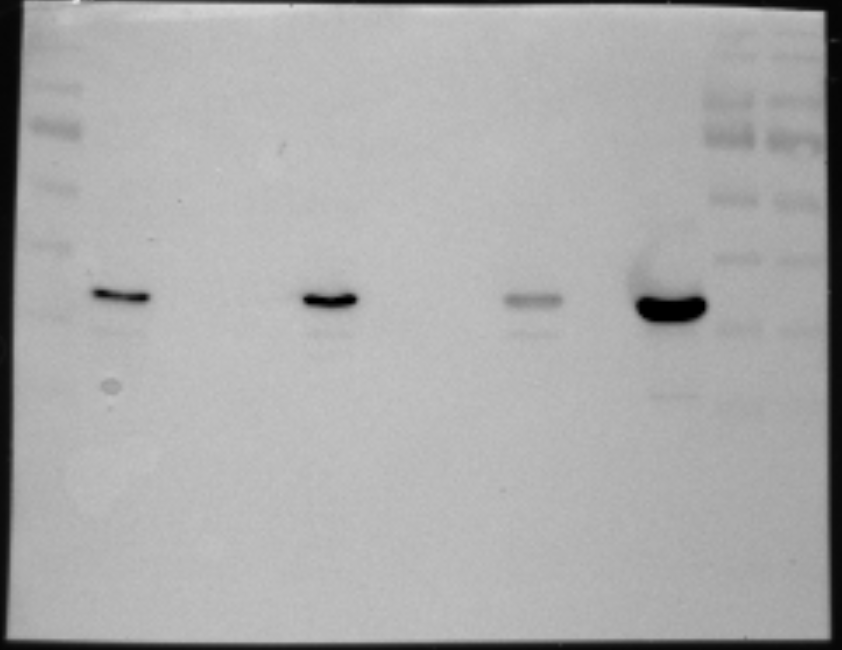

Supplement: Supplementary file 6 — Source Data Fig. 6 [file 44319_2024_107_MOESM6_ESM.zip › Figure 6/6B/His WB NBS1 FHA+tBRCT Replicate 2+Membrane.tif]

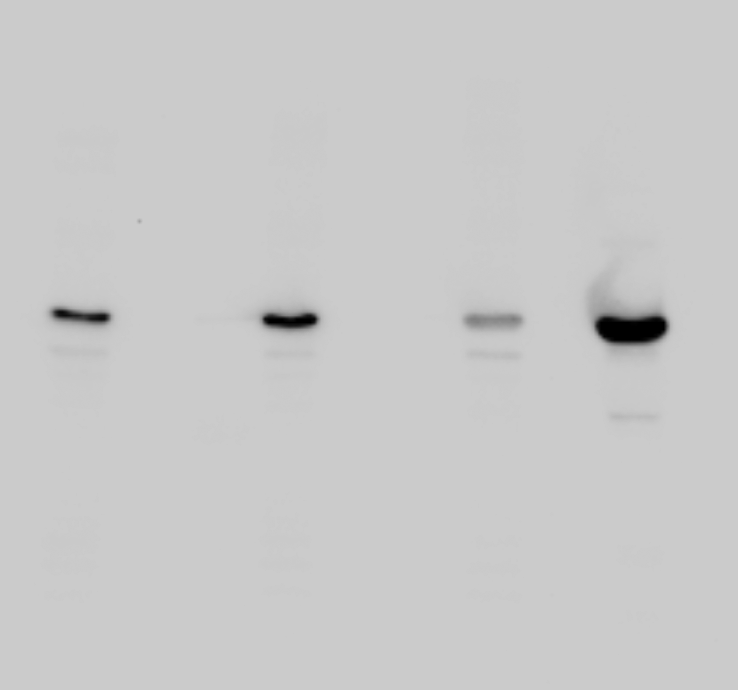

Supplement: Supplementary file 6 — Source Data Fig. 6 [file 44319_2024_107_MOESM6_ESM.zip › Figure 6/6B/His WB NBS1 FHA+tBRCT Replicate 2.tif]

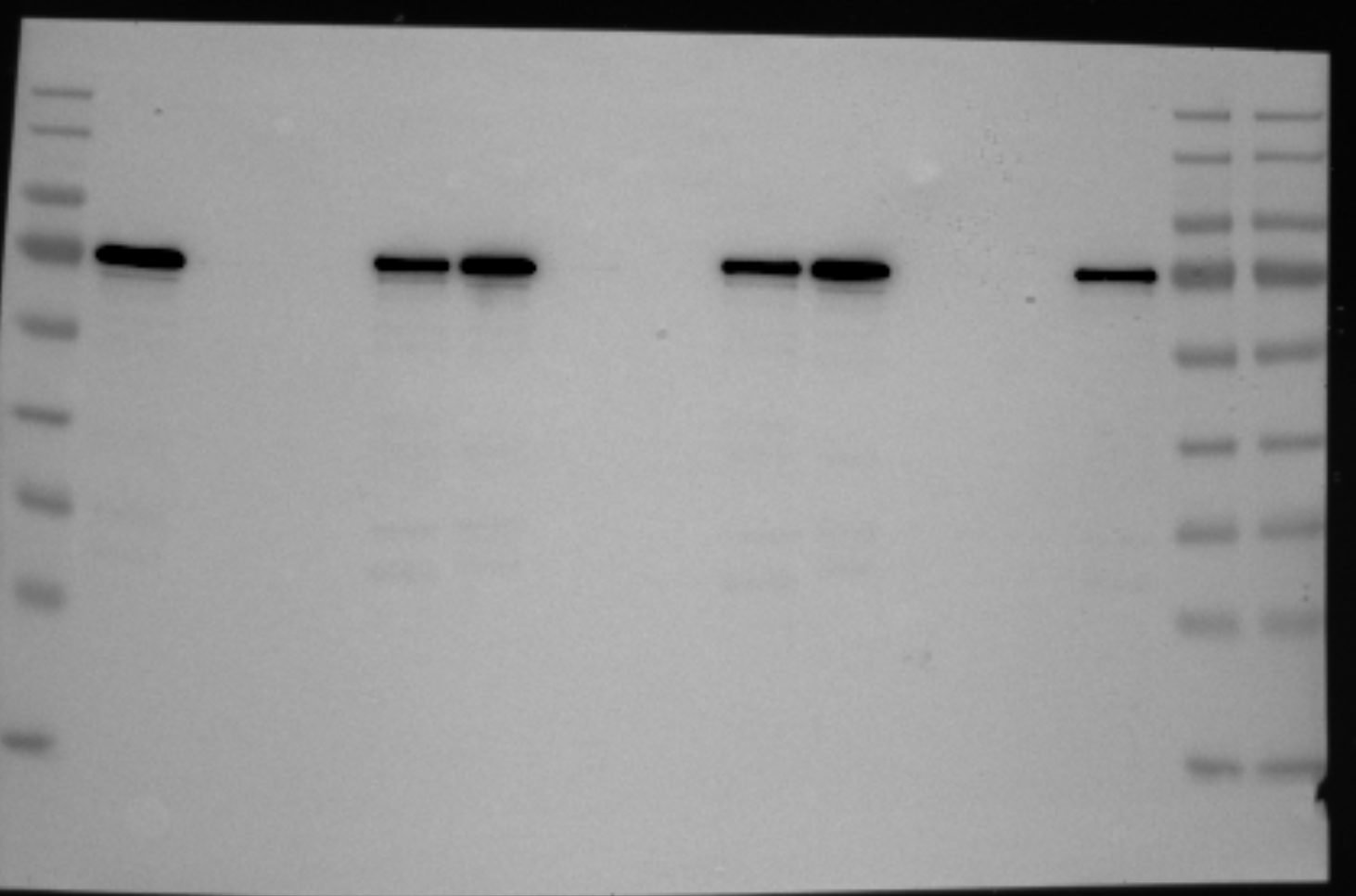

Supplement: Supplementary file 6 — Source Data Fig. 6 [file 44319_2024_107_MOESM6_ESM.zip › Figure 6/6C/His WB+membrane.tif]

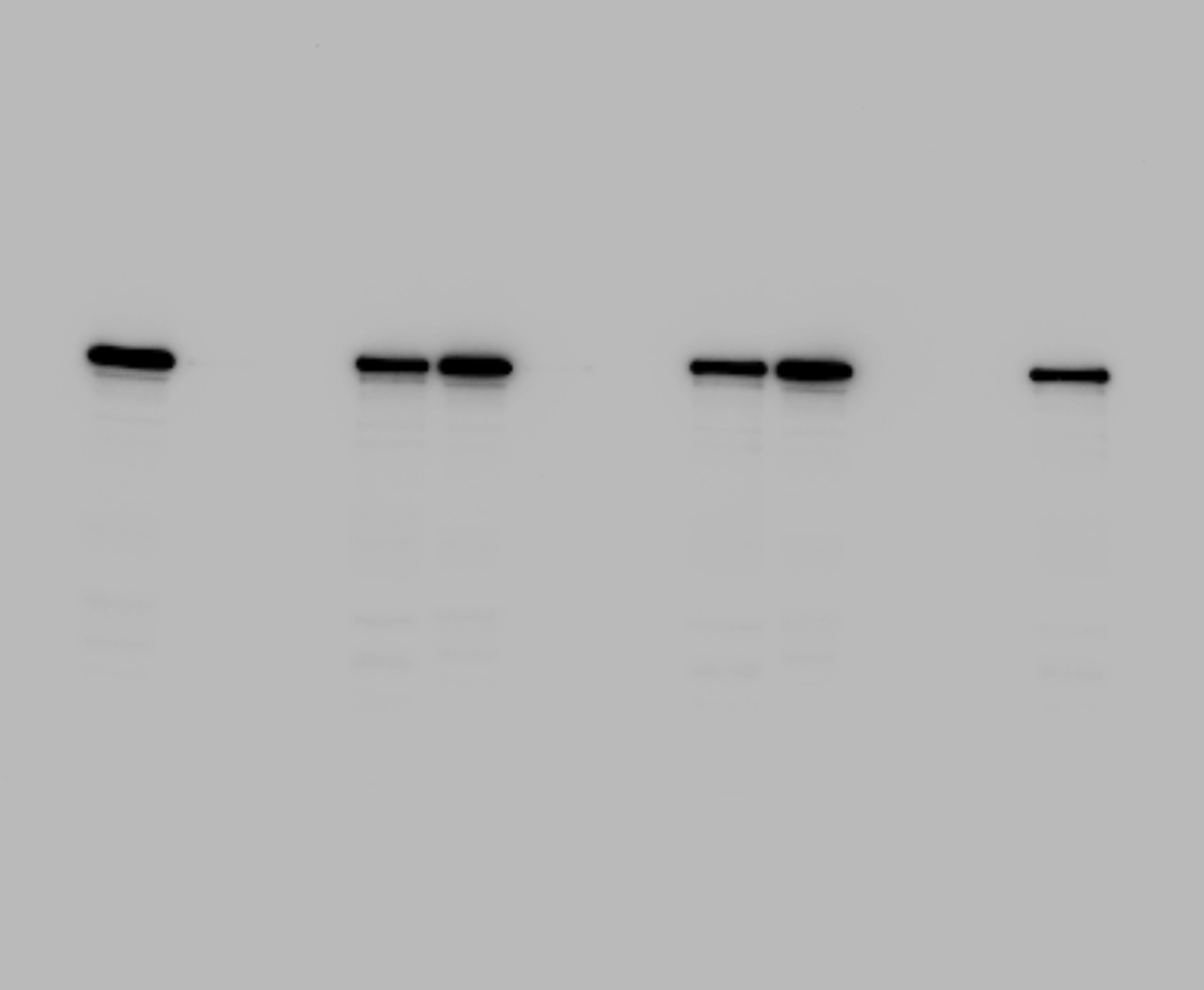

Supplement: Supplementary file 6 — Source Data Fig. 6 [file 44319_2024_107_MOESM6_ESM.zip › Figure 6/6C/His WB.tif]

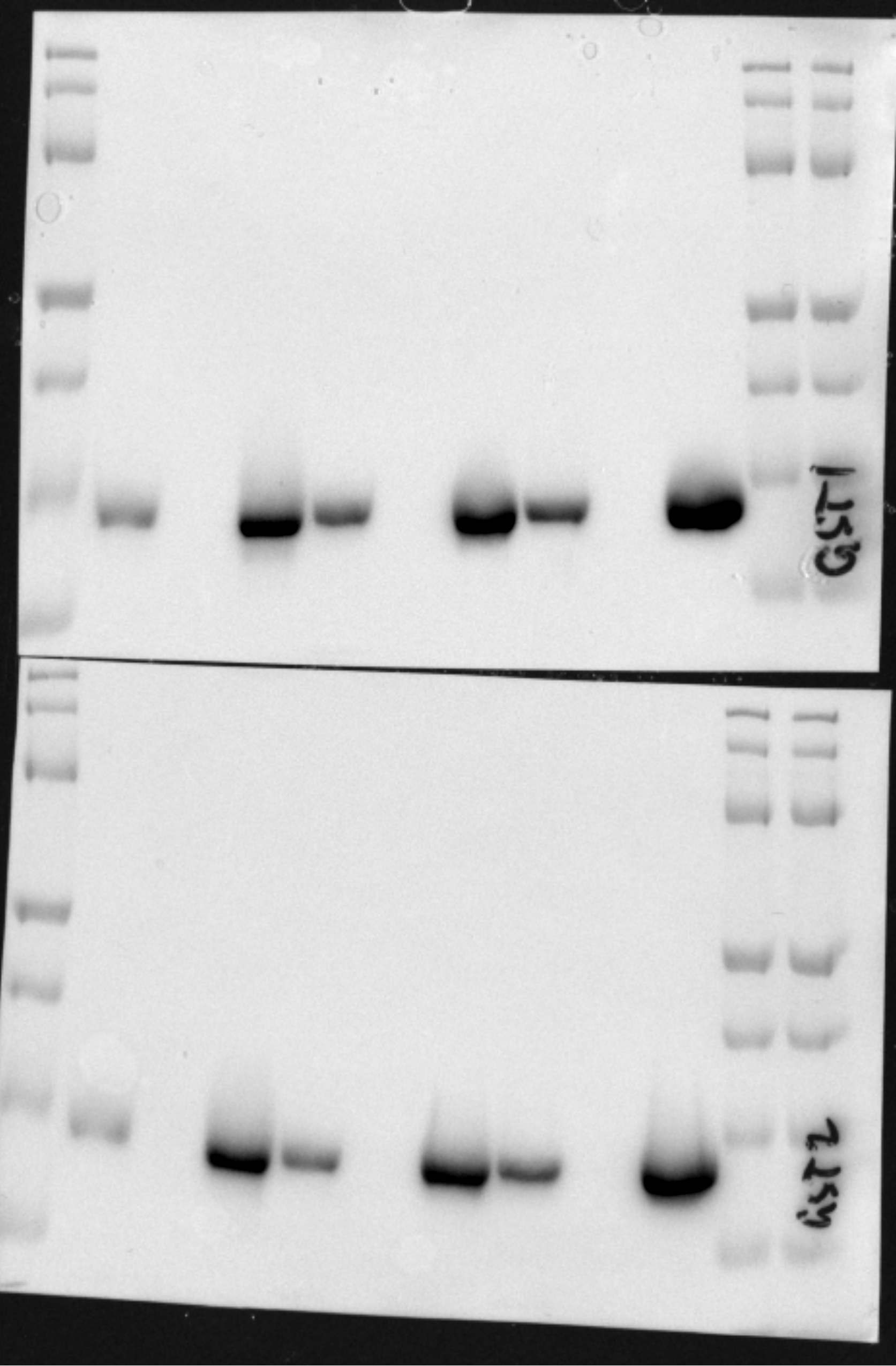

Supplement: Supplementary file 6 — Source Data Fig. 6 [file 44319_2024_107_MOESM6_ESM.zip › Figure 6/6H/GST WB NBS1 (FHA+tBRCT)+SQpoint mutants Replicate 1 and 2+Membrane.tif]

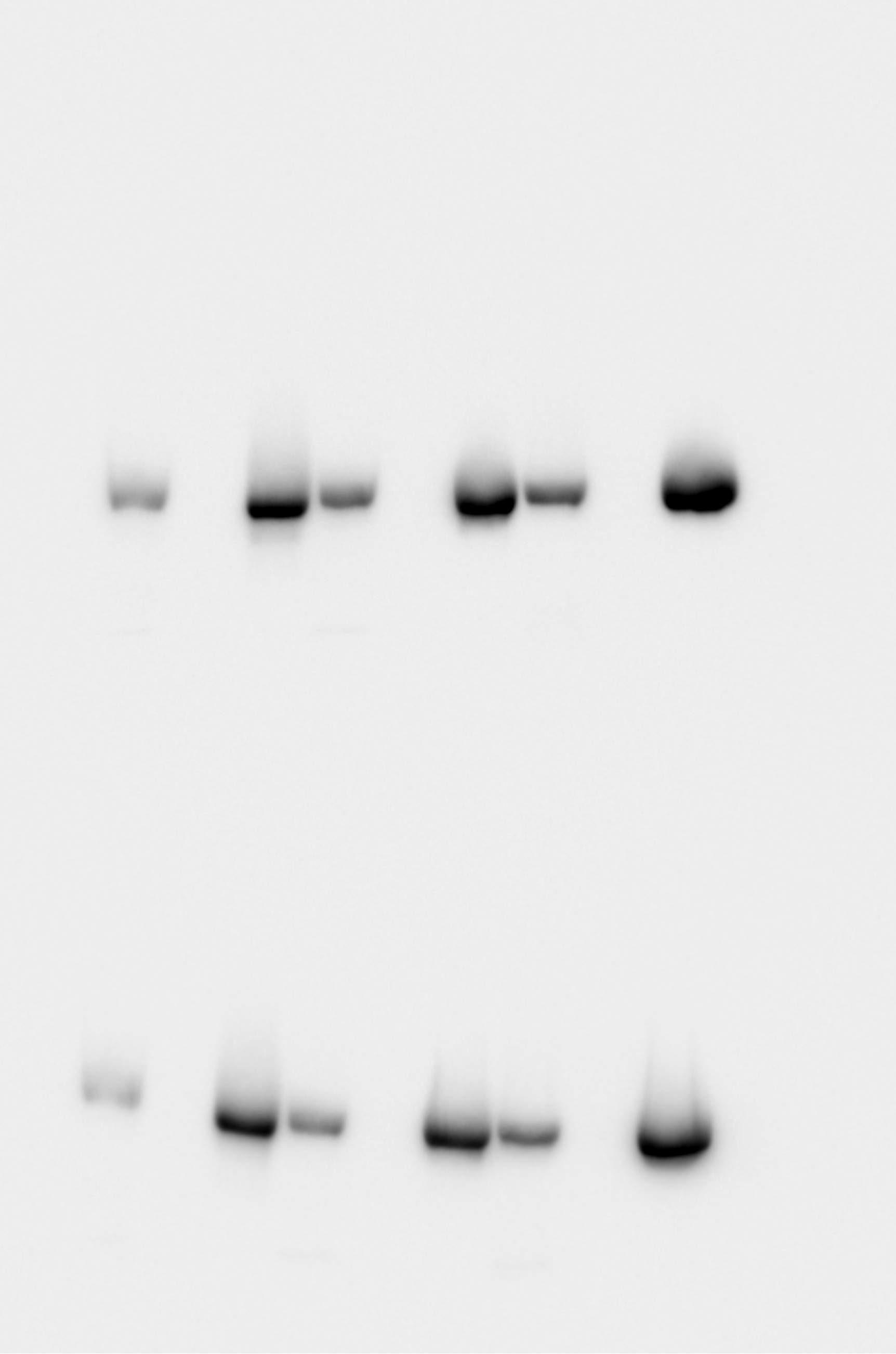

Supplement: Supplementary file 6 — Source Data Fig. 6 [file 44319_2024_107_MOESM6_ESM.zip › Figure 6/6H/GST WB NBS1 (FHA+tBRCT)+SQpoint mutants Replicate 1 and 2.tif]

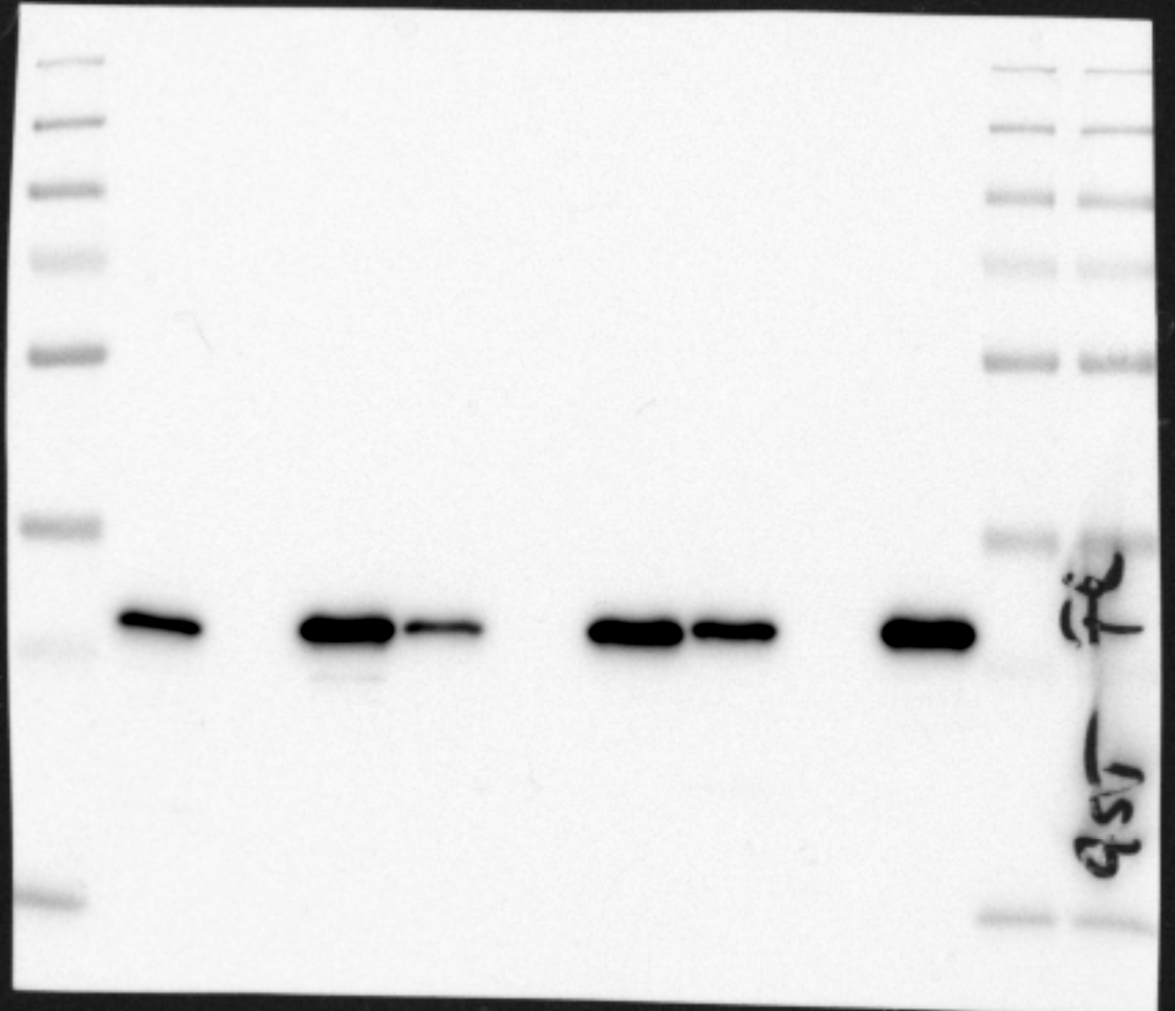

Supplement: Supplementary file 6 — Source Data Fig. 6 [file 44319_2024_107_MOESM6_ESM.zip › Figure 6/6H/GST WB NBS1 FL+SQpoint mutants Replicate 2+membrane.tif]

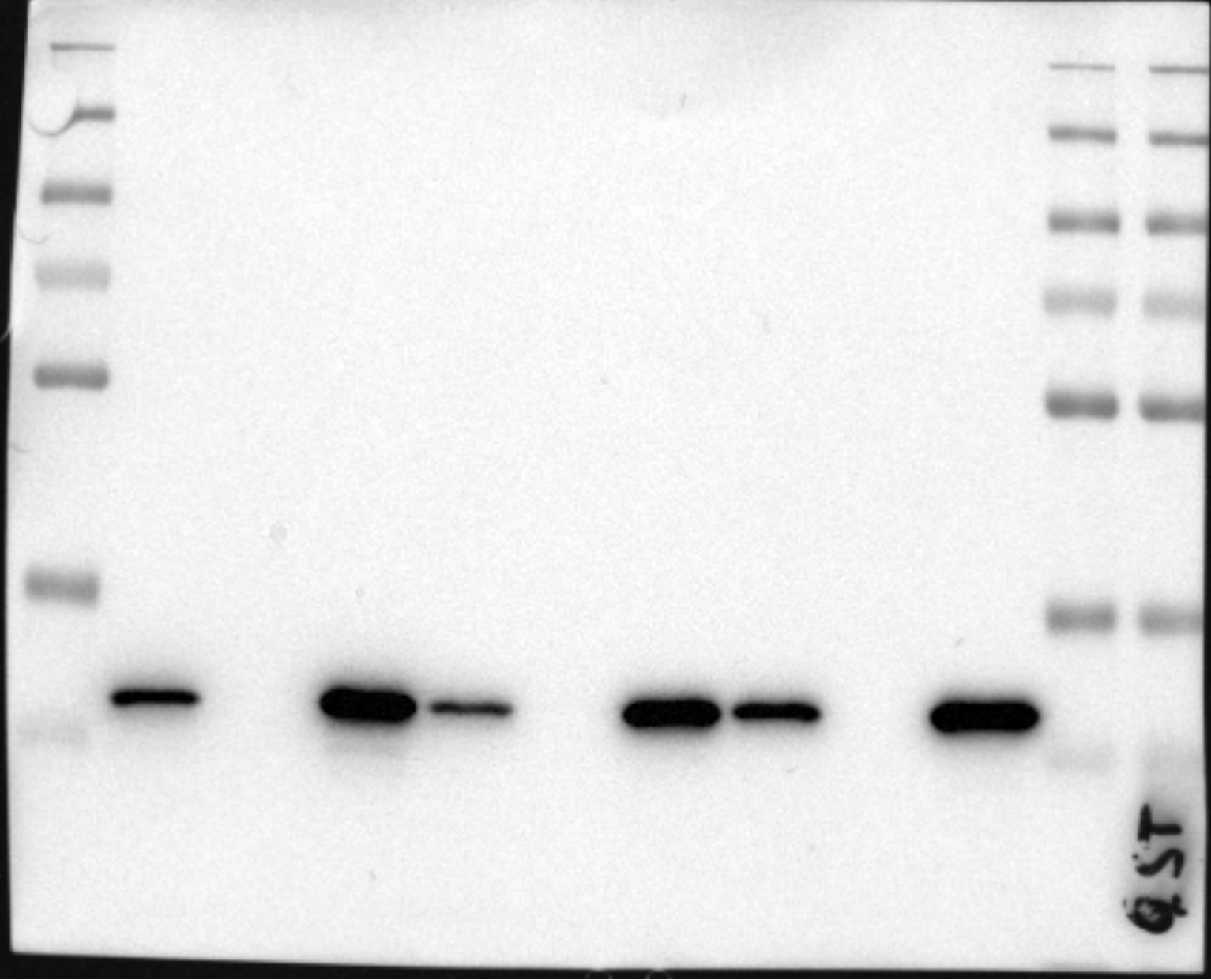

Supplement: Supplementary file 6 — Source Data Fig. 6 [file 44319_2024_107_MOESM6_ESM.zip › Figure 6/6H/GST WB NBS1 FL+SQpoint mutants Replicate 1+membrane.tif]

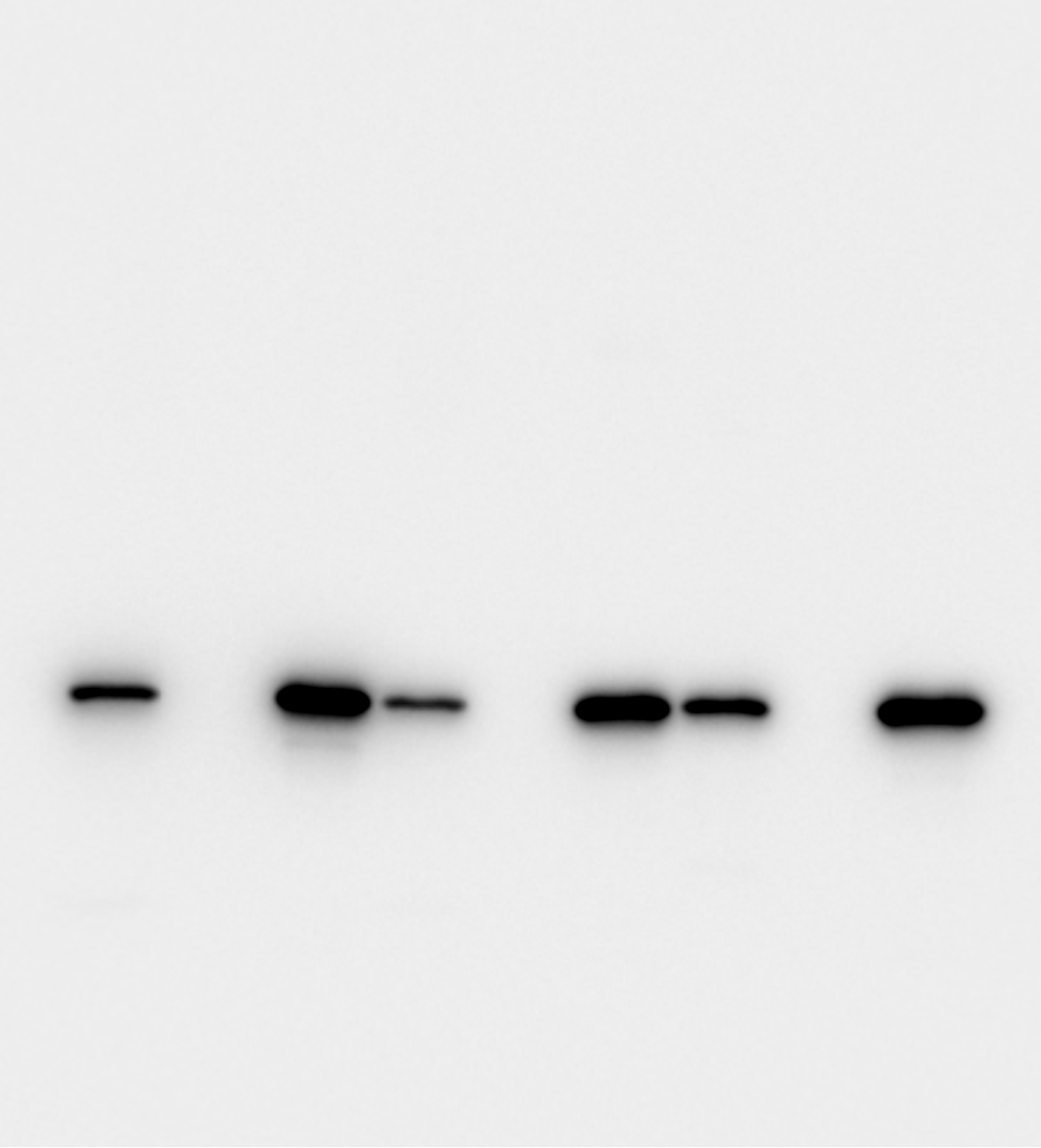

Supplement: Supplementary file 6 — Source Data Fig. 6 [file 44319_2024_107_MOESM6_ESM.zip › Figure 6/6H/GST WB NBS1 FL+SQpoint mutants Replicate 1.tif]

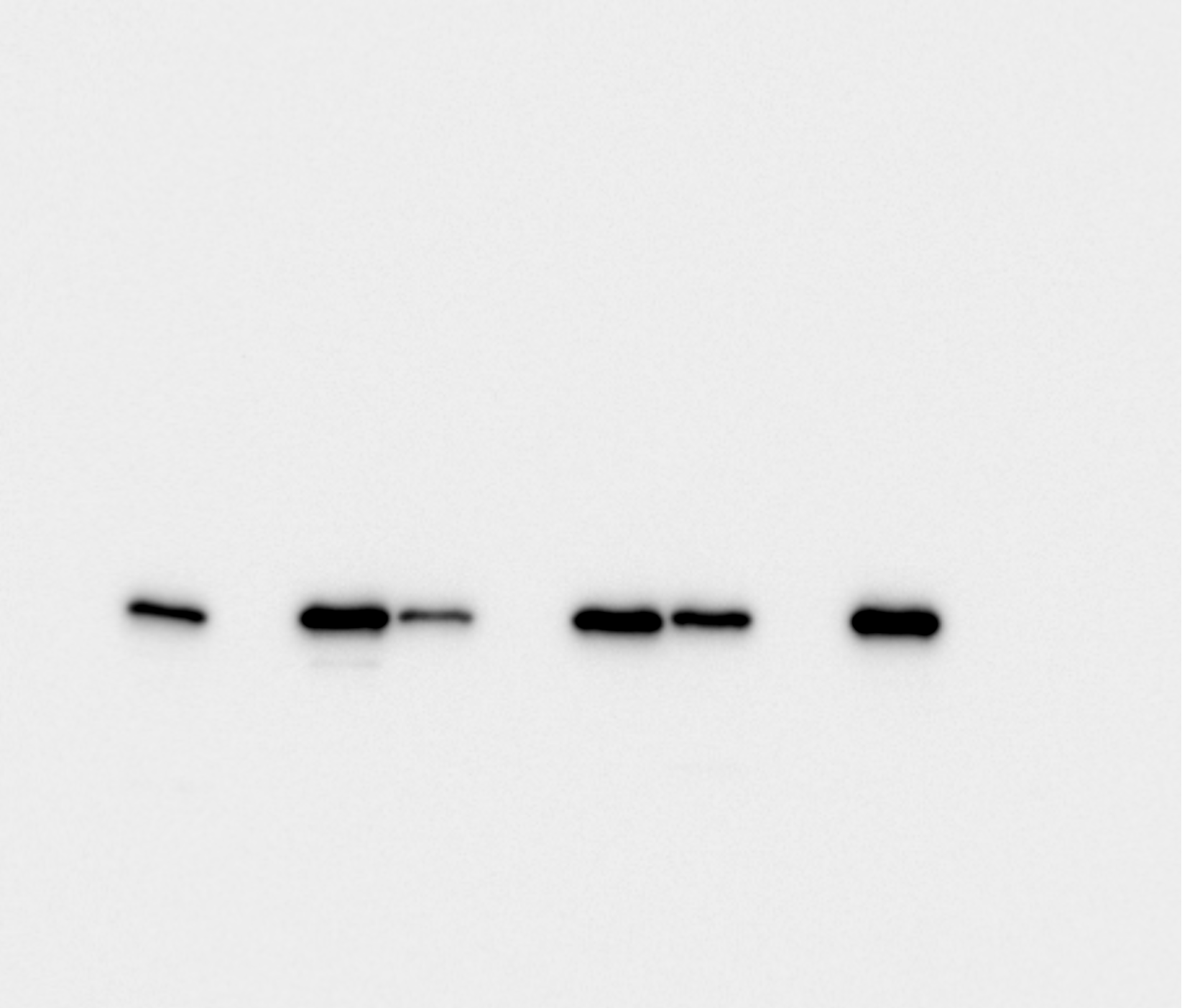

Supplement: Supplementary file 6 — Source Data Fig. 6 [file 44319_2024_107_MOESM6_ESM.zip › Figure 6/6H/GST WB NBS1 FL+SQpoint mutants Replicate 2.tif]

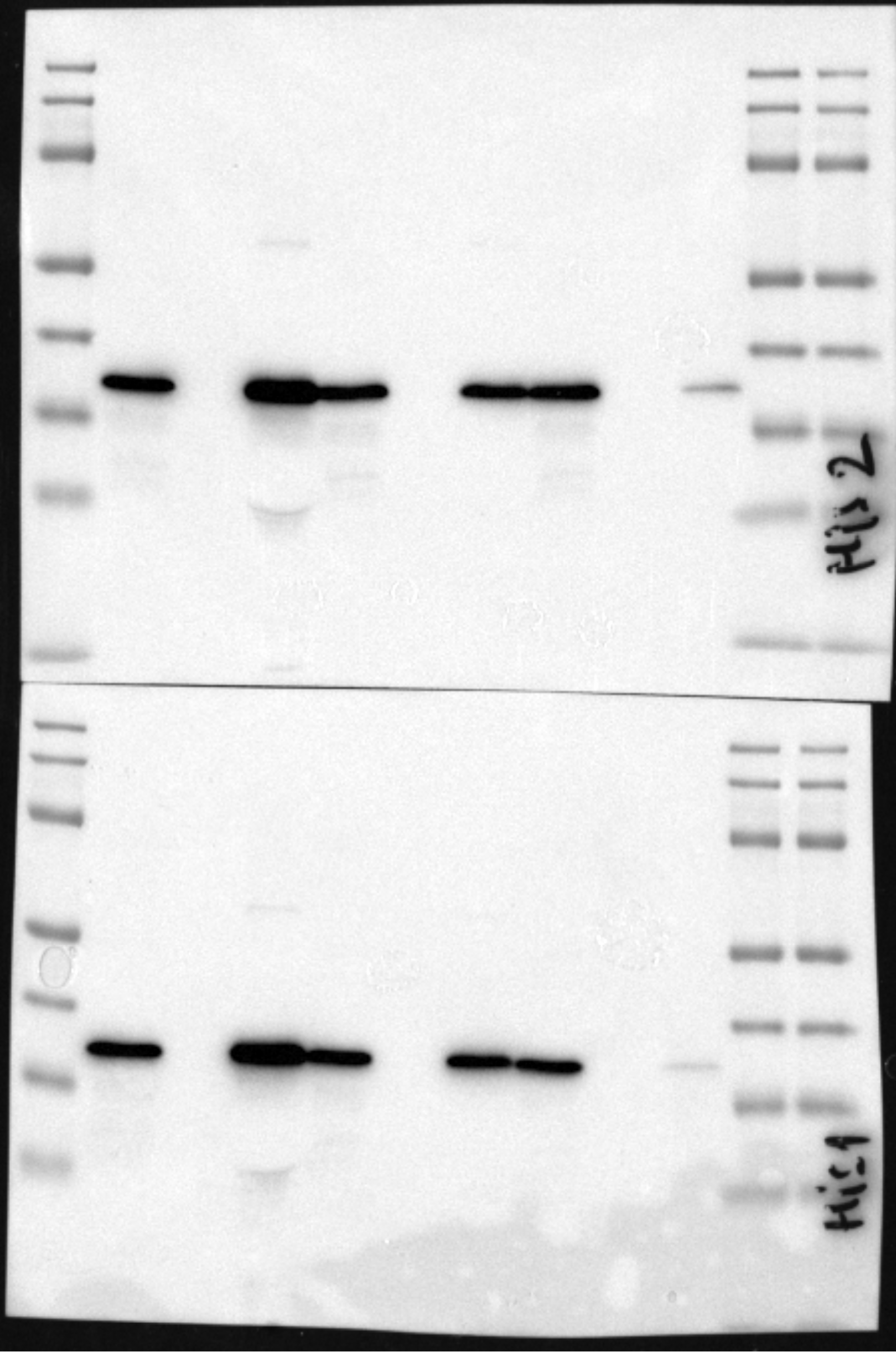

Supplement: Supplementary file 6 — Source Data Fig. 6 [file 44319_2024_107_MOESM6_ESM.zip › Figure 6/6H/His WB NBS1 (FHA+tBRCT)+SQpoint mutants Replicate 2 and 1+Membrane.tif]

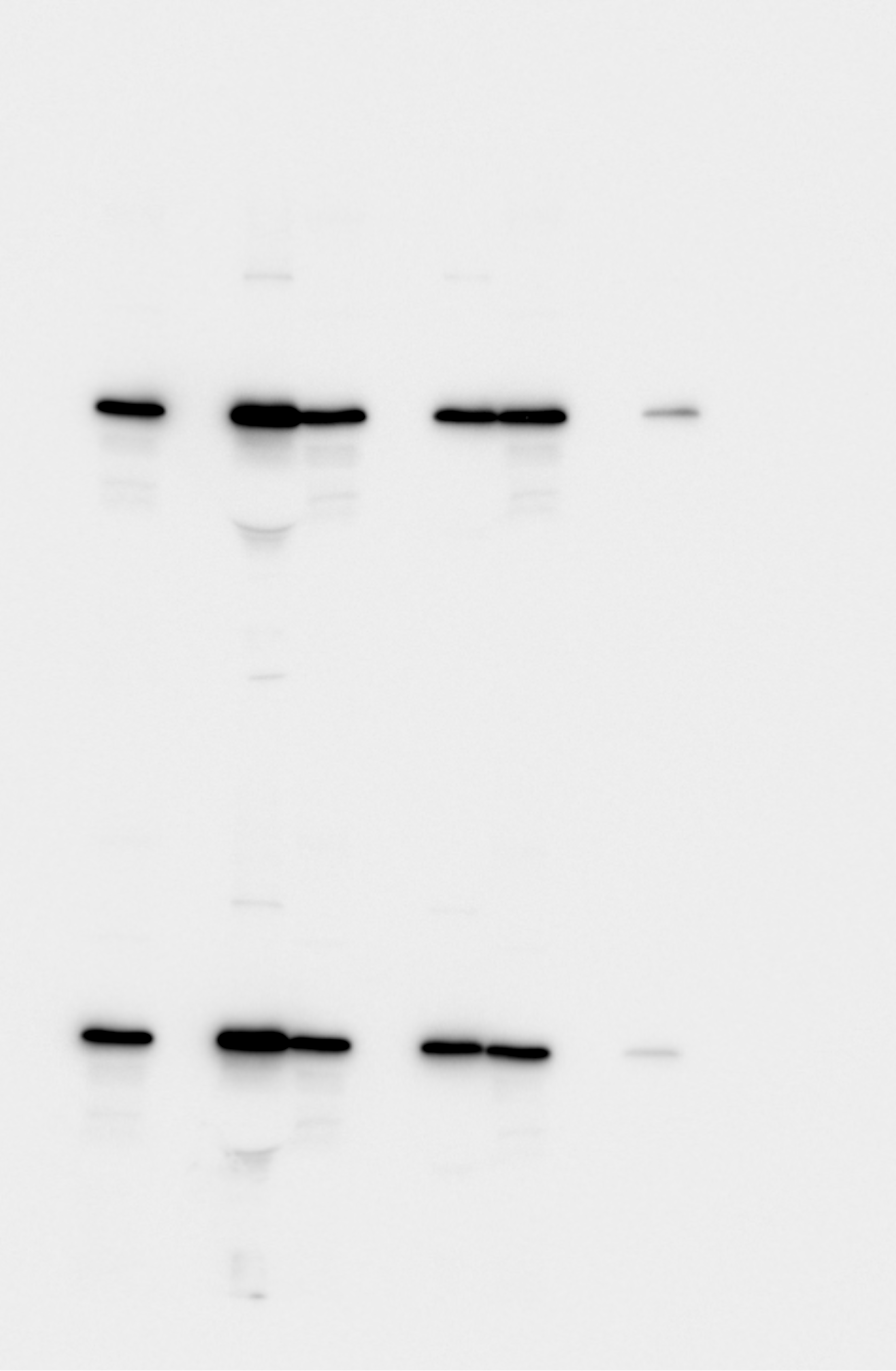

Supplement: Supplementary file 6 — Source Data Fig. 6 [file 44319_2024_107_MOESM6_ESM.zip › Figure 6/6H/His WB NBS1 (FHA+tBRCT)+SQpoint mutants Replicate 2 and 1.tif]

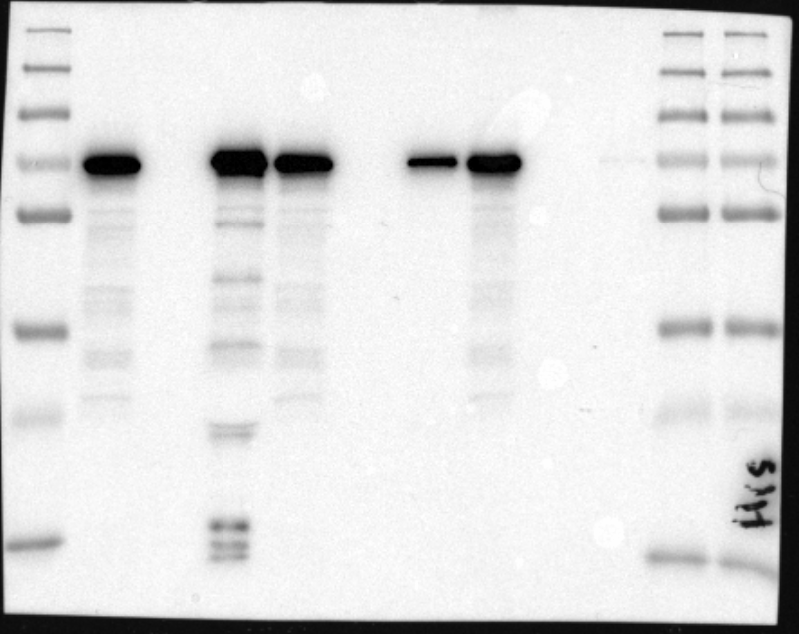

Supplement: Supplementary file 6 — Source Data Fig. 6 [file 44319_2024_107_MOESM6_ESM.zip › Figure 6/6H/His WB NBS1 FL+SQpoint mutants Replicate 1+membrane.tif]

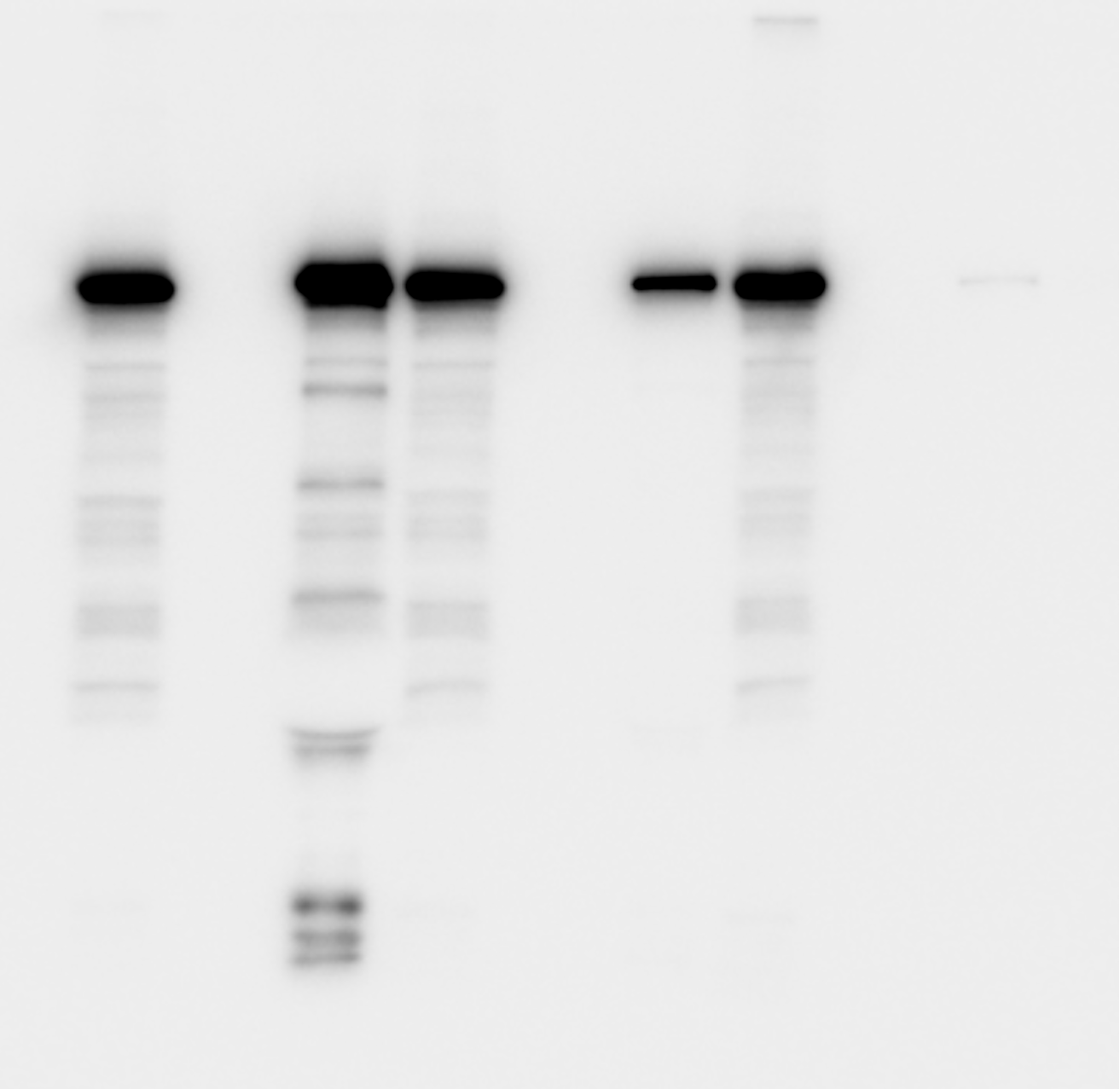

Supplement: Supplementary file 6 — Source Data Fig. 6 [file 44319_2024_107_MOESM6_ESM.zip › Figure 6/6H/His WB NBS1 FL+SQpoint mutants Replicate 1.tif]

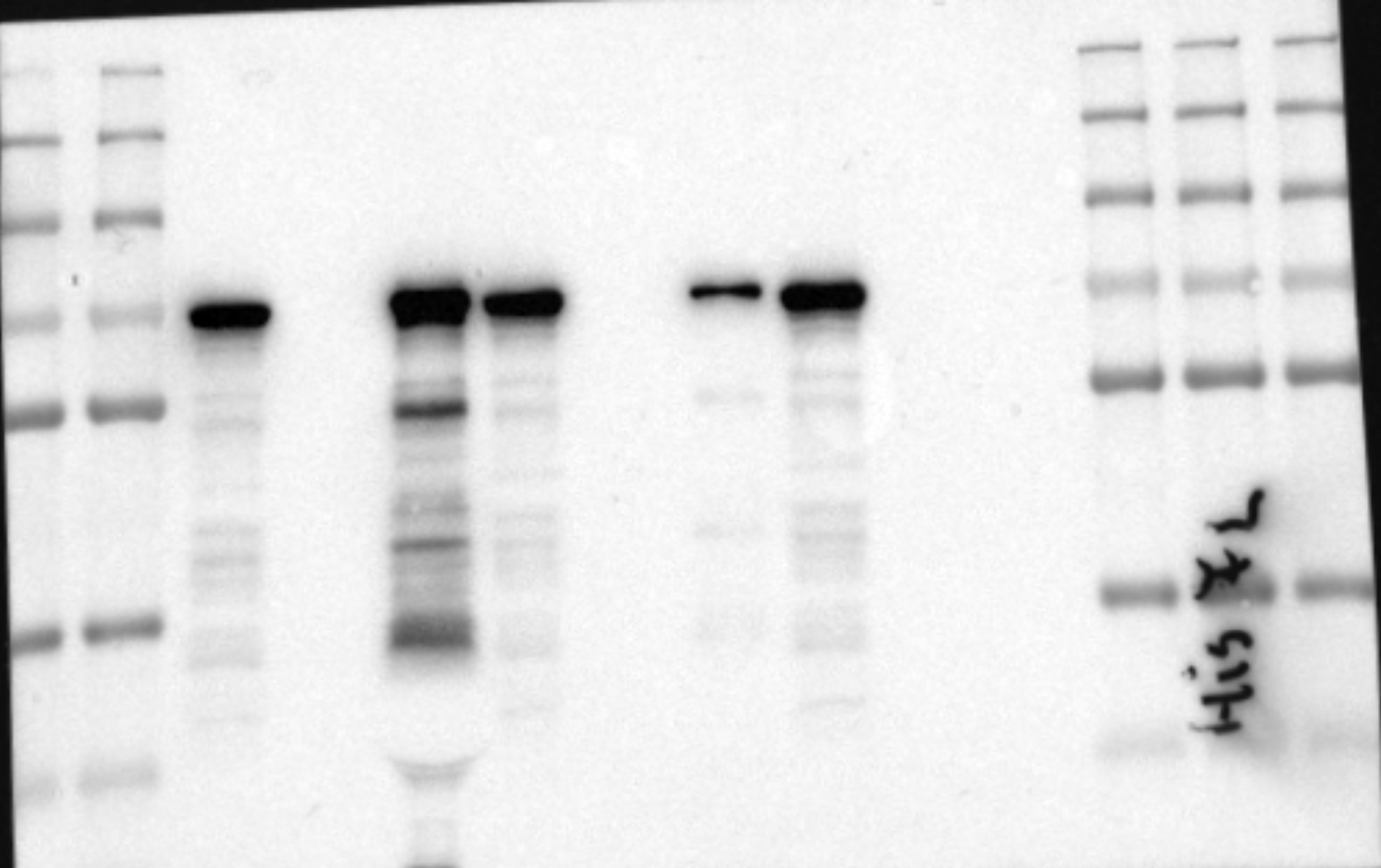

Supplement: Supplementary file 6 — Source Data Fig. 6 [file 44319_2024_107_MOESM6_ESM.zip › Figure 6/6H/His WB NBS1 FL+SQpoint mutants Replicate 2+membrane.tif]

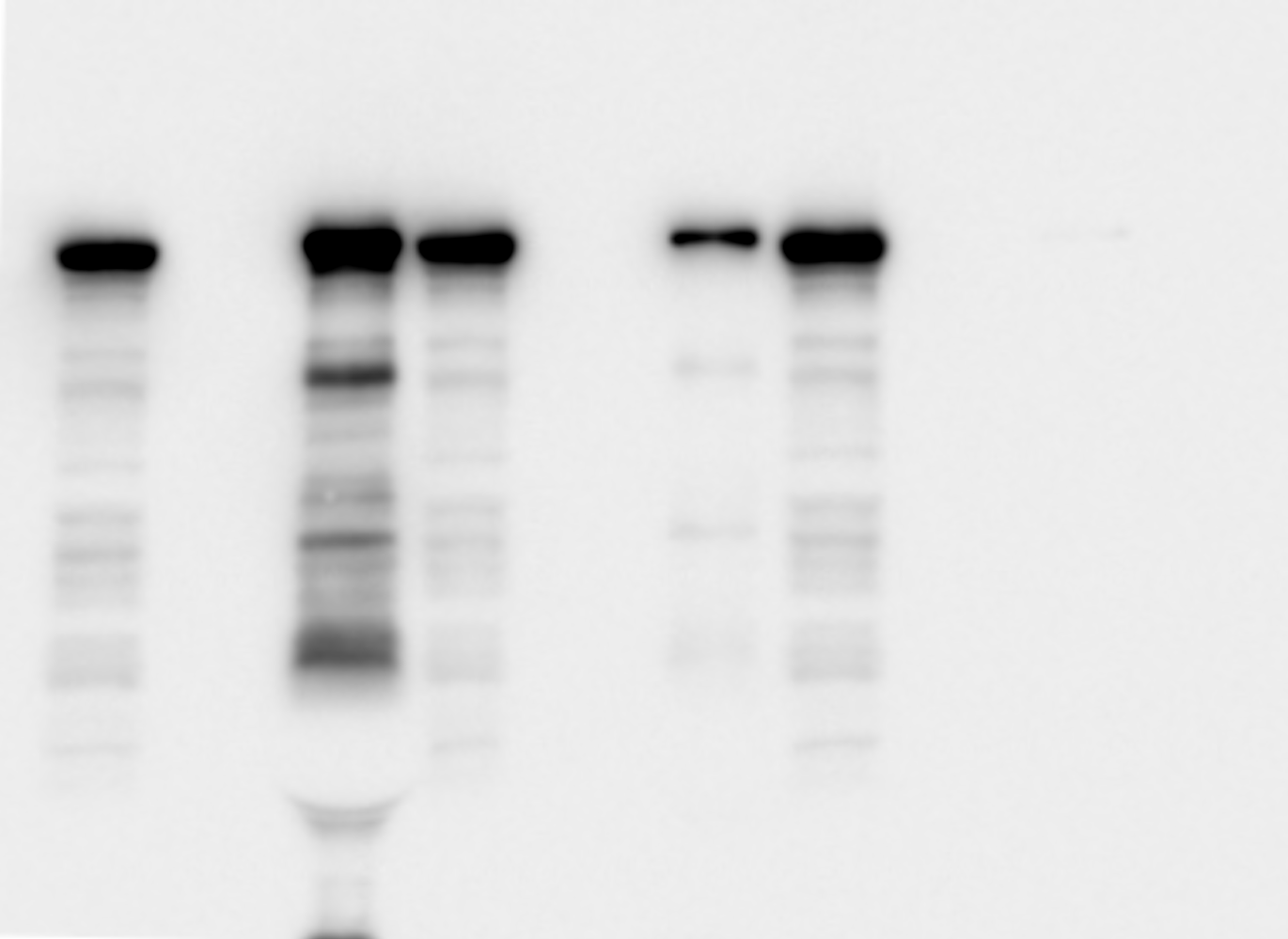

Supplement: Supplementary file 6 — Source Data Fig. 6 [file 44319_2024_107_MOESM6_ESM.zip › Figure 6/6H/His WB NBS1 FL+SQpoint mutants Replicate 2.tif]
