# Supplementary material for: Identification of plants’ functional counterpart of the metazoan mediator of DNA Damage checkpoint 1
Source: EMBO Rep. 2024 Mar 4;25(4):19. doi: 10.1038/s44319-024-00107-8 (PMC11014961; doi:10.1038/s44319-024-00107-8)
Supplement: Supplementary file 2 — Source Data Fig. 2 [file 44319_2024_107_MOESM2_ESM.zip › Figure 2/2B/EMBOR-2024-58742V1_SourceDataForFigure2B.pdf]

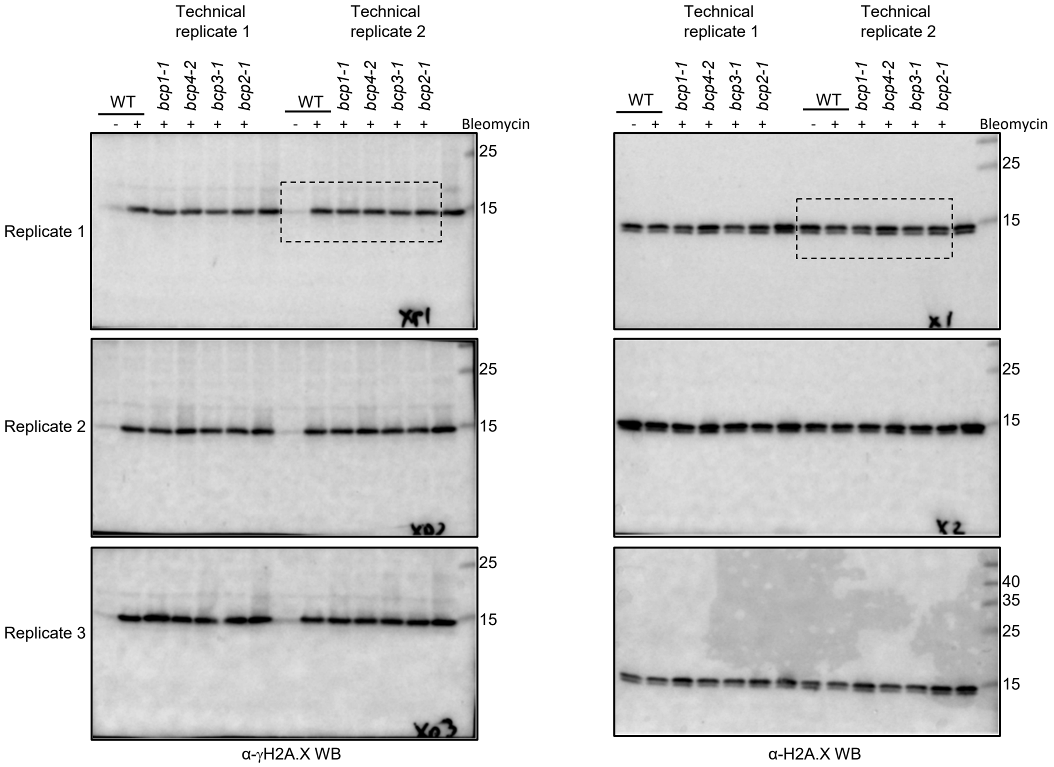

Source data for Figure 2B. Uncropped images of γH2A.X and H2A.X western blots in *bcp* mutants. Each blot represents one biological and two technical replicates. Dotted boxes represent images presented in Figure 2B. Shown are chemiluminescence signals overlayed with membranes.
