# Supplementary material for: Identification of plants’ functional counterpart of the metazoan mediator of DNA Damage checkpoint 1
Source: EMBO Rep. 2024 Mar 4;25(4):19. doi: 10.1038/s44319-024-00107-8 (PMC11014961; doi:10.1038/s44319-024-00107-8)
Supplement: Supplementary file 3 — Source Data Fig. 3 [file 44319_2024_107_MOESM3_ESM.zip › Figure 3/3E/EMBOR-2024-58742V1_SourceDataForFigure3E.pdf]

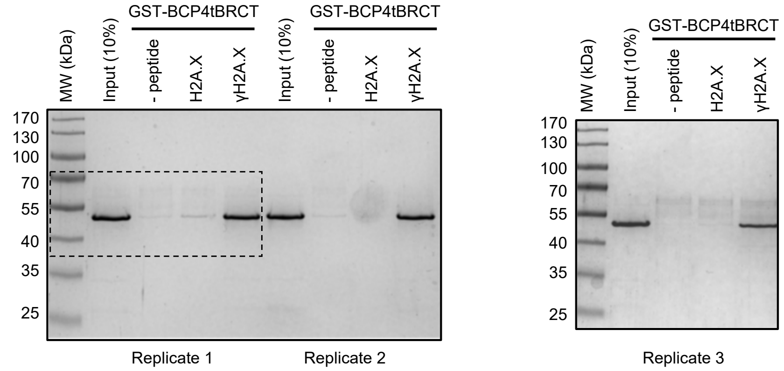

Source data for Figure 3E. Uncropped images of affinity pull-down using GST-tagged tBRCT domain of BCP4. Dashed boxes correspond to images presented in Fig 3D-F.
