# Supplementary material for: Identification of plants’ functional counterpart of the metazoan mediator of DNA Damage checkpoint 1
Source: EMBO Rep. 2024 Mar 4;25(4):19. doi: 10.1038/s44319-024-00107-8 (PMC11014961; doi:10.1038/s44319-024-00107-8)
Supplement: Supplementary file 5 — Source Data Fig. 5 [file 44319_2024_107_MOESM5_ESM.zip › Figure 5/5A,B/EMBOR-2024-58742V1_SourceDataForFigure6A,B.docx]

Source data for Figures 5A,B, EV1A,B and EV2A,C

**PLANT BCP1, BCP3, and BCP4 SEQUENCES**

**BCP1 SEQUENCES**

**MONOCOTS**

>Spolyrhiza_BCP1

MASALGPNEGEVFSGVRFVLVGFDSVSEAQYRSELVRRGGIDVGRYDSSCTHVIVFGRVH

DDPVCIAANNDGKVLVTELWIEDSADFSELANADRILYKPVKDLNGIPGSESLFICLTGY

QRRERDDIMKMVELMGAHFSKPLIASKVTHLVCYKFEGEKYELARRVNIKLVNHQWLEDC

LRAWEILPEDDYRMSGWELEIMKAEAKNSDDDTEESSRIRPTRKRSITTETSDMQSSMPV

LHSPKPSLRVLETSIIKQEDTVPSEALHSKLEEVLVGNASLSNPSNERCSAIASNMRNNG

KVTEKLFTQDGSGTKDSITKDAHLQNPAVTSDNTANFNHAGNELSPDSLNRSFSRSYAAE

MKSGSASYSGKNRRKLVSTEDLARRMGSPQRSSKANDIPSSIPRGQTSNVGPADVQSPTP

MTELSRMEGPCNALPQKRKVPTFSPKSIKSGLKNSVTSAWESYSAASVSSQLEVSVKTSH

QGGSLSSLDDLKKADGHGSPLISKSSVSRKLNEHEPFSRDNEKVCLNGKVTDSLHNAGSI

MSPLHLSSTDLSPVGALSHSGSSPSRLHQDCDLGRAPSSTLCIRNTVDDLGALSIDCQMA

DGVSTECKKPEQSPSKTSELYLKHDKVDIPMSDLSDIHTGENGQTRPLKDSGSSSGDNKD

SGMGKFSDPSNPNRRVLSKALNKRVVAKKKLTCTGKPSVESKNGSSVYSNKVVSPNLSTC

RREQQKEPEHGQKANDEGDEMVGTISKEVAMEGSLKEPQNSPSKGDRISSGENETHEFKS

MEVEGPALKGASVNTVARGKMKKAHHRPGAAPSAKNMPAAAMHGSNDKGKENKSVEDTSI

NSKTDKFGGKSGESTRRADEAATGQLQNHQHPECISEPSWFILSGHRLQRKEFQKVIKRM

KGRLCRDSHNWSYQATHFIAPDPIRRTEKFFAAAASGRWILKSDYLAACSEAGKFLPEEP

FEWHGCGLTEDGAISLEAPRKWRLLRERTGRGAFYGTRIIVYGECIAPSLDTLKRVVKAG

GGAILATSPPYTRVVNSGVDFAVISPSTPRSDPWVQEFLRREIPCVLADYLVEHVCKPGY

PLDRHVMYGSLPLAEKSLAALRRRSEVASDDLSCSVCGSLERGDVMLICGDEQGLTGCGA

AMHTDCCQPPLSAVPEDDWFCPRCAQGEPTKR

>Sitalica_BCP1

MDSSGTDYDIGDEHLFDGVRFFLVGFESDVESQYRSEMEVRGGADAGSLGNGCTHVVVSN

LFYDDPTCVAARAEGKKVVIDQWVEDSLDRGVLADVDRVIYWPVRHSNGIPGAQSLLICL

TGYQRNYREYIMKMVSLMGARFSKSLIANVVTHLICYKFEGEKYEVAKKVNIKLVNHQWL

EDCLKAWEILPVDDYSKSSWELELMEAQANDSEHEAEAAGPRSLSNRSSVRCTLNSKNCK

ETFVKYDVDARKRSPVIPSGNKEVVVGRNLNSPDHILKTEDADSKTRDITGQSSASSMVP

VSAKADVFARIQSPLGHIMNAEDADSKRHDIRGQGSPNSSLLAVSAKVDGLSPIQTSLGL

SQKRDNSVVRNNNSPNLQEAERKHVGARTQDFASGVLGTSSSKMTAFSNHHLDTLNKAPG

ILKGHTDHVSGKSSASQDHIDVTKVPLSSPLRGNQSADELDSSKVDRWQRHEKCAPSGIH

ITAAGRSNTDDKLTNHESNPKSGGDSKFNSIKNTSNSKKASRKSLLPEVHSVNHMQSPKR

AEDSTLRADSNISSLEMGHQKVFENADDQSKKGNENIKCVDGLNGAYAQKRKILVSPASL

NLQKEDLVSETGPLDSPFASWLSDASDAEANAVNFGKQQFSLSTSRQRRSRKTSLKHGGL

INGIKLPESSSSDKNVKSSLKARMSLKAMVENKCTRTPSPAVQDGKTSFSFQNKDGEDTQ

GSGNAVNQDCLHEIGNLRTKDQAHDKSVHNSSNSHVVSSSGNVGTKVTDPLKVNDYEEPV

VSNSELERVVSDANVKDKEDAKRLQDTSSNVQGETSYSKKVATPERRNAGAKRPRSASIE

AEGSAINSGKKVVTESWPAEVIPHENADPASKNGCTMASAAELKTNPSKKALICRVTDTV

AKRTRNACAKTDDTQVASSLEFSKVISQENIEANPKKFFDTGNADEQQRNSPKKIPNTRV

RNTAAKRSRKSDINTSNEPLVDKTGTVATGSLFDDLFPSDNEDYPKKLSSCASASDCGTL

SSKTVSNGRTRNAVAKRKMKTVEDKSGSKCGKVGSVIASVAEAVSSKRTEEISCNSNKIT

TDQDSDKSNKDVIKDASGLFCQDSGTVDKQGGSYNFNLRSSKRNKALTSDHEKENRLDHS

NLNSISNRTSSLQSKFDAKSIEKSTRVLSDSEHQGVKVSESGTLIVSEPALFILSGNREQ

RRDCRSILRRLKGRVCRDSHHWSYQATHFIAPDPLRRTEKFFAAAAAGRWILKKEYLTSC

TEAGKFVDEEPFEWFGSGLNDGETISLDAPRKWRNIRQQMGHGAFYGMQIVVYGQLILPT

LDTVKRAVKAGDGTILATSPPYSRFLDSGFDFAVVSASLPRADAWVQEFIRHGIPCVSAD

YLVDYVCKPGHPLDRHVLFKTNDLANKSLEKLMKNQQEMATDMEQSEDEEDDPEDLSCSA

CGCKDRGEVMLICGDEDGETGCGIGMHIDCCDPPLDAVPDDDWLCPKCAVPKAKRKPTRG

TERKARGSRRR

>Jascendens_BCP1

MPPPAAGDGGDGDEQLFAGVRFVLVGFDSVSEAQYRSEMVRRGGAEAGRYDAGCTHVVVC

GRVYDDPVCVAARKDGKALVTELWADDSLDLRALADAERIIYRPVRDLNGIPGSESLHIC

LTGYQRNGREDIMKMVSLMGAQFSKPLIANQVTHLICYKFEGEKYELAKKVNIKLVNHRW

LEDCLKAWAILPIDNYTKSGWELEILEAEARDSEDEAEDVGRRPFDIRASVRSTPSTKNG

MKISADPDVHAPSRTPIIPSGNGGAVAGRHLNNPNRIIEDEAEDVGRRALDNRADVRSTP

NTKNGIKFSLGVLAPIRTPTIPSDNRETVAGRHLNNPDQISVTNNLFTTPSRETWYVEAL

NPSNVSNKEEDKRDDGAAGDRNATADGSNVEDKLILCEANLKSGGNAAMEKIRNLSYSRK

HPRKSVSPEGPSAYQITSPQRTELRADCNVSPVAERGDQKISEFGDVKSPKDDEMIKYVD

GSGSALAQKRKSFISSVSPKLPNGVVGSATGVSKSPFVNEESAESEPATISSLENNFIES

TKADSHVEGDATVNFMQKRQSGSSKSKVSSYRKTSLKRVRPVEGVKLPEHSANGKNVDSP

GKAKALDEAMTENTTNLKCAVSPASTAANGKRNSVLSLHNEEMEEAGAQLSVGNALSRLG

SLLIPGRTRKSLMKDKTPKKSLCSSKNSKLGSRSGNTDIIMTDAPVLNKSETVASKSVLD

KVVSHENIEKEPKMLSSSASAVEYETRSPKKVPTTRVRNAVAKRSRNVDIEMTDAPIVNK

NETVASKSVFDRVVSQENAETDPKKLSSSASADEYETRSPKKVPTARVRNAIAKRSRNAD

IEMTDVPIVNKNETVASKTEFDRVVSQENAETDPKKLSSTASADEYECSPKKVPTARLRN

SVAKRSRNADIEMIDAPIVNKTETVTSKSKLDKVVPSENVETDPKKLSSTASTDDYETSS

PKKVPNTRVRNAVAKKKMKVGQHKFAGGSGKNDSVSVSTVKADTSKRTEENSRNVDKVNA

DQELQKATEDGMKDAAGSFCKDVIMVDKSEVLQNSKSRSSKRNKTVHLMEISSDPEKENR

PDHGNVSSKSKGVRNDYMISTHGTKSVQNGEHVLGEHSKMIQGNGYGTMTRLEPAWFILS

GHRLQRRDYRSILRSLKGRVCRDSHHWSFQATHFIAPDPLRRTEKFFAAAAAGRWILKTD

YLTACNEAGKFLEEEPFEWFGNGLNDRDTISLEAPRKWRLLREQTGHGAFFGIRIIVYGQ

CISPSLDTLKRAVKAGDGTILATSPPYTRFLKSGVDFAVVSASMPSADAWVQEFGRHDIP

CVSADYLVEYVCKPGYPLKKHVLFKTHDLAEKSLEKLLKDQREVIVDPAEPSEESAVDLS

CSVCGSNDREDVMLICGDEDGATGCGIGAHIDCCDPPLEAVPECDWLCPKCDITKAPKAK

RTKSRASKLR

>Sbicolor_BCP1

MASPSPGTDDDIGDKHLFDGVRFLLVGFNSDDASQYRSEMVRRGGADAGPSGNGCTHVVV

WNLIYDDPTCVAARAQGKKVVNGLWVEDSLDCGVLADADRVMYWPMRDLKGIPGAPSLLI

CLTGYQKSYREDMMKMVSLMGARFSKPLIANAVTHLICYKFEGDKYEVAKKVNIKLVNHR

WLEDCLKAWKILPVSDYSKSGWERELMDAQAIDSEHEAEASDPGSMNYRPSVSGNREVVA

GRNLNTPNHIINTEDANNKTHDIRGQSSANSMVAVSAKIDVFAPIQSPSVHIMNIEDADS

KTHHITGQDSPDSGLLASSAKADILAPIQSSGEKKDIVRNTDSPNLQEVKEKYAGARTQD

LASGVLGPPSTSKMTVFRNHHVDTLNGTLGILKVHTDHVSGKYSASHDQTDVAEVLLTSP

LIGNQSVDELDSSKDDKWQHQEKDGPSGIHIAAPGQLNVDSKLNNHESNPKPGSDSMSNN

IKNTSNDKKAYRKSFLQEGHSVNHMASPQRAEESTLRGYPNISSLETAHQKVVEHADVQS

IQGNQNTKNEDGVDGTYAEKRKSLVSPACLNLQKEDLVSETGPLDSPFVSRLSGASETAN

VSSERINLVETNAINLGNQHSSFSTSKQTRSRKTSLKHGGPIGGIKLPDYSSSDKNVKSL

RKARMSFKATTESKCTMSSSATVQDGKTSAGFQFQNKDGESTQISGNAVNQDCLNEIGNA

CTKDQAHEKSVNSSNNSQVVPYYGNAGNRIADPVKVNGNEVAVASSSELKKVVSDATVKE

STQQFQNTSRNVQAETSYSKKAPTTIRRNAGVKRHLSANIESEGSVINSDKKVVPESSPA

KVMIHHEHAGPVSKNGNSAACAAELKINPPKKAPICRVTDTVAKRTRSACTKIDGTWVGS

SLQFSKVMPQENIEINPTKNLDTANANEQQRNSPKKIPNTRVRNRAVKRSLKSDTNMSND

TLMVKAETVAAGSLFDDWFPSGNVEDEDWPKKIPGHASVNDCETLSPKTVSNARIRNAAA

KRKIKTVEDKSGGKFGKVGSAITSETKAFSSKRTEGISCNINKVIADPESEKSNKDVRRE

VSGLFCQDSCTIDKQGPYNSKLRSSKRNKALTSAHEKENRLGCSDLNSKPDRTGSLCSIS

DAESMKKSTLVLSKHQREKISESGTLITSEPALFILSGNRQQRRDYRSILRRLKGRVCRD

SHHWSYQATHFIAPDPLRRTEKFFAAAAAGRWILKREYLTSCIEAGKFLDEEPFEWFGTG

FNDGQTISFDAPRKWRNIRQQMGHGAFYRMQIVVYGQLISPTLDTVKRAVKAGDGVILAT

LPPYTRFLDSGVDFAVVSETIPRADAWVQQFISHGIPCVSADYLVEYVCKPGQPLDRHVL

FKTNHLANKSLEKLMKNQQEMAMVRSKPSEDDEDDPEEDLSCSVCGQKDRGDVMLICGDE

DGETGCGIGMHIDCCDPPLDAVPDDDWLCPKCAVPKSKTKRTTTCGTKRKGRGVSSSRRR

>Bdistachyon_BCP1

MHGSDDDVDYGDDARLFAGVRFALVGFDSVSESQYRSEMARRGGADAGGHGAAGCTHVVV

CGLVYDDPVCAAAREGGKKVVTELWVDDSLDMGALANADRVLYKPQRDLNGIPGSQSLNI

CLTGYQKNRREDIMKMVTLMGANFSKSLVAGTSTHLICYKFEGEKYELAKRVDIKLVNHR

WLEDCLEAWEILPIDNYTKSGWQQEMMETQVEDSEDEAEDVGRGLSHSRVIPRSVPITKI

RTATHVDPDRRTPIRGPTVSTGNAQVGAGGHMDTPKQVIEAEDISKMSIDIRADVQSTHN

TNGVTSSADPEAHDSVHPPVNPSNNEKAPGGHIIGDEAKYGDKRAVDTTASTLSTLNTSG

ATVQADHLVHQPIVTPSTESPLKKTLHSSHTCEKVDQKDDEPVADLAAKVVQSNVQGNVT

LCKVNLTSAGNSAPKNTPVLSYSSRRSRKSVSPGANLNSVHQTASPQSSKTSTLNDELNI

SPSVKSNHKISKHTDAKSLQDKACALAQRKSRFSSVGPITPNGSTDSATGTADSLFSSKE

IASEAAAVSDLVKKSTGSQPVKVDSNINSNATVNLVERQKSGSPKKKLSYRRTSLKLTRS

SEVEKLPESFANEKNLESLAKAKKQPRHEAAVEKGCAISPSVDSELGNTSSSLTLGNLGI

EMGETPQVNNIEVVSPNLQHDKVVSCATTQSGAHKASTSGVKKAGAKRSRNAGNETHTVS

VDGESEAAASKYKHDEVISHEDVEREAEKDCTSPNDAERTTLFPEKSSSSRARNAAANSS

LNENSEMNDVIAASNMKNPQGNINKNHKQLSSGASAHEYKESSSKKVPNATETNDVANVS

RSVKIKMTEAPSADKTKAGSLISSLSGVVPQAYTERLSSNGSANKHEICNPDKGPGKRMR

NAVAKRKVSAAQQHRSGSEPCKTGGVFSSDAEVITSKTAADSSGNANKITVDQDVQNANK

DAMTNADGSFCKDSSKVASKDLQKSKLRSSKRKFLAVVEDGSTNHNKENIQVNANITPKA

KCGNSTKAVQNSKDVLDDPSIREGYDCTTLTMLEPTRFILSGHRLLRKEYRLILRRLKGR

VCRDAHHWTFEATHFIAPELRRTEKFFAAAAAGRWILKSDYLSACNEAGKFVEEEPFEWH

GDGLNNGETISLDSPRKWRQLRQRTGHGAFYGMQIIIYGECISPSLDTLKRAVRAGDGTI

LATAPPYTRFLKPGVSFAIVSAGVPRTDVWVQEFMNHKIPCINDYLVEYVCKPGHPLKKHILFDMDDLADESLQKLQKAQRDLGAGTEEVTEGGDNTEPSCSACGSNNREGSQLICSGGEGNQASCGVAMHVDCWNPHHPEPVPDGEWLCPKCDEHMEPLKKAKKTSRSRVRKRS

>Pvirgatum_BCP1

MASPGTDDDIGDNHLFDGVRFFLVGFEGDVASQYRLEMERHGGVDAGPSGNGCTHVVVSN

LFYDDPTCVAARAEGKKVVSEQWVEDSLDHGVLADADRVIYWPARHSKGIPGAQSLLICL

TGYQRNYREYIMKMVSLMGARFSKPLLANAVTHLICYKFEGEKYEVAKKVNIKLVNHWWL

EDCLKAWEILPVDDYSKSTWDLELMDAHANDSEHEAEAAGPRPLNSRSNVRCTPNSKSCE

EAIVKPDVYASKLSPITPSGNRLVVAGRNLNTLGHIIKTEYAENKTHGVAGQSSATANST

IAVSAKVNVFAPIQSPPGHIMKTEMESSKTHDIIGQGSPNSSLLAVSAMDDVLAPIQTSL

GLSQKRDNSVVRNINSLNVQKAEEKYVGVNTQDLASGIVVPTSSSKVTLFSNHHLNTLNG

TPGILIGHTDNVSGKSSASHDQIDVSKVLLTSPSRGNQSVNVVDSSKVNRWQHQEKDGPS

GIDVTGAGWLTTDDKVTNHESNPKSGGDSKSNSIKNTRNSKKASQKSSLLEGHSVNHMAS

PKKAEESTPRADSNISSLQMGHQKAFEHADVQSMKGNENIKTVDGLDDGAYAQKRKSLVS

PSSLNLQKEDLVSETGPLDSPFVSWLSDASDAEANAVSFRKQQSSLSTSRETRSRKTSLK

HGGPINGIKLPESSSSDKNVKSLPKARMSLKAMEESKRTTSPSPTVPDGRTRSSFSFQNK

DREDAQGSGNAVNQDFLHEIGSVRTKDQARDKSVRSSSNSRVVSSSGNAGTKITDSLKVN

DNEVAVASNSELEKVVSDANVKEGKNRFQDTSSNALGETSNSKKVATHVGRNAGAKRPRG

ASIEDEGSAKKVVPESWPAIRHGHADPASRSVCSSAGAAELKTNAPKKTLICGVTDIVAK

RTRNACAKIDNARLTSRLEFSKVNSQENVEINHKKFFGTENADEHQRSSPKKIPNTRVRN

KAAKRSRKSDTNTSNETLVDKSETVAAGSLFDDLFPSDNVEECPKNLSSCASASDCGTLT

PKTISNVRIRNAVPKRKIKTVEDKSGRKCGKVGSAIASVAKAVSSKTVEEISCNINKVTA

DPDSEKSNRDMTRDVSGLFCQDSGTVDKREGPHNSKLRSSKRNKVLTSYHEKENRLDCSN

LNSKSNRTRSLCSKSDAKSIEKQTVVLSEHQRVKGNESGALILSEPSLFILSGDREQRRE

YHSILRRLKGRVCRDSHHWTYEATHFIAPDPLRRTEKFFAAAAAGRWILKREYLTSCVEA

GKLLDEEPFEWFGTGLNDGETISFDAPRKWRNIRQQMGHGAFYGMQIVVYGQLILPTLDT

LKRAVKAGDGTILATSPPYTRFLDSGLDFAVVSASIPRADAWVQEFIRHGIPCVSGDYLV

EYVCKPGHPLDRHVLFKTNNLANKSLEKLKKNQDEMATEETEQSEDEDDPEDLSCSACGL

KDRGEVMLICGDEDGETGCGIGMHIDCCDPPLDAVPDDDWLCPKCAVPKAKTKPTRGAER

KARGSRRG

>Othomaeum_BCP1

MAAPYSDDDGADPQFLAGVRFVLVGFDQLSAPQYRMEMERAGATDAGDWNGDCTHVIVCG

LLYNDPMCVAAKKAGKKVVTEQWVNDCLDLGELVDADRVLYAPVRDLRGIPGSQSLLISLTGYQKAMREEIMICIAILKAWEILPIDNYKESGWEIEIMEAQVKDSEDETEDAGRGSSSSVRVNRSTPASRTRSTVDSDVKAPTRAPIIPSSNADGAAGRHSGNAPNTNSVTVSVNSRATPCGSDEVPANQVTRGEAEDDHNRTPKTRVTVLSTPSIHKSIATPIQVDNMENNDANCLHNSNQVALNNVRWPNPSEENLSRTTLPSLESNRNVDQKEDRHTPYLKSTVRQSFVEEKPNLCESSVRSEGNSTSQNNHMVGYCRRRSPKSVSPDTNLRSVNKKSSPQSSEGNTSKIEFSTPQRKHDQDFSEPADSRSLQEAEVVKHVDISSNALAQRRKSILSSVSPKSPNGDPGSRTGLVSSLVSRMENASEGAIGSNQSRNSAECTKVDSHCNSGPTVNFTEKQMPGSFKRNLLSSRRASLKLVNSREAKRLPENVSVDKDLGLLGEVKIPALPKATTEKDCSICPSFNSEFRKESSGVSLQNGNTGMSDAEQVDKVDVAARSSESDKAIPHQNFEAGPTDIPVNAIIGQDSKTHTKMSTSRVRNSGAKRSRNARTKTATVFAKHKPEVAASKPMDHKAVSCENANTLRGEKCSSPNNGESALLLPDEEISNDKPGNEASKSELVNVIPQKNMEDNKKLSSSESADENLEKSSTKVPDSLARNSVAQGSHSADGKTTDSVTAEKPGTMPLESNFSKLVPQENRETYPKKSSRNAGKVTVDQVVQNANEDKTNDPVGSFFKDKMEDRSKDTQSSELRDSKRQKTINLVDGSLDHDKENIAVSSNLSSKTKSGIDGVSSKSSTKALGSGKVVLDENSMIKGNDRGTLDLLEPTWFILSGHRLLRKEYKSILKRLKGRVCRDSHNWSFQATHFVATELRRTEKFFAAAAAGRWILKPDYLTACNEAGKFLEEEPFEWHGHGLNSSETISLDAPRKWRQQKQRTGHGAFYGMQIIIYGDCIAPTLDTLKRVIRAGDGTILATSPPYTRMFKSNVDFAVVSTGMPSVDAWVQEFMRHKIPCISPDYLVEYVCKPGNSLSKHVLFEMYDLAEKSLEKVLKNQHDGITGEAAEADVVCSTCGSSDRDRPMLVCGSDGNSAGCGIVVHVDCCDPAVEAGARAGEWLCTKCDDESRPAKKARKSAKSRVLKGR

>Zmays_BCP1

MPPHGPEEDAADAHLFAGVRFALHGFDQISASQFRLEIERCGGVHAGGWDADCTHVIVSN

TLYDDPVCVAARKAGKKVVVDQWVEDSFDLGELADADRVLYAPVRDFKGIPGCDKLHICL

TGYQKNWRDDIMKMVSLMGANFSKSLAANIITHLICYKFEGEKYELAKRVNIKLVNHRWL

EECLKAWEILPVDHYTKSGWEVEIMGAQAKDSEDETEYAGTGSLSSRRIDRRTPIREIST

KSHVDSALHAPSGGPTVSASNVVGATGKHLSTPEQFMKAHDASTKSSDIRTDSGTAPDTK

CAAMSVDNDTLKPTRSHIYHNENDEVPADKASRDEAKDDHRRELDTRDSTLRTPSVHKSI

APTIPGDNIENTNRNCFHGFSQINVNSDLQLNSSEENFSKKILHSTNLSRKVDQKDDGHV

PDPKPNISQSSVEENLNMCEFNPRSEGNSASRNDQPLGYSRRRSSKSGSLNVHRKKSILS

SVSSKAPNEAPDSGSGISSATASDVGRSPTEPTKVDGHVNSGPTMNSTENQMSGCKSNLL

SYRRTSLKLVSSAEVEKLPENSVNDKNMVEPDVVKTPALHEATTEEPSPSVCSEVRKESL

GVHQNRDAEMTDAQLLSKTEDAAPCSKPDKVVSCQNSGLEGCKDVPVNKITDEHGIFPSK

ISTSRVKKVGAKRSRNAGSKAASEFTNSRSEVAALKPKHDKVASHENVEAQQGEGCCSPN

AAECTPPSPAEALNNKSTNEVLTSSQGPNHKTNESLVASKAESVNMTLQRNKKGNRRKLP

NTSNADENQRSSSRTVPNSKSGNLVAKGSRTADFNISDSPTVDETETVPSNSSFNEAVPP

GNGEEKHKRLSSSANADDPEACTANKVPNNRVRKVVAKRKLSDVQSHKSGSEPCKTAKVL

VSEVKVVSPARVAQSSRNANKVTVVQDLQNTNTGRTNDTVGSFCKDVTEDCSKDMQSSKT

RSSRRQKIADLVDGSTDHDKENILVDSNFTSNTKRGNNSMSSKSNTKTLQSSKAVLDESS

VIKRNGYGTLDVPEPTWFILSGHRLLRKEYRAILRRLRGRVCRDSHHWSFQATHLVTTEM

RRTEKFFAAAAAGRWILKPDYLTASNEAGKFLEEEPFEWHGEGLNIGDTISLDAPRKWRQ

LRQRTGYGAFYGMQVIIYGECIAPTLDTLKRAIRCGDGTILATSPPYTRFFKSSVDFAVV

SAGMPSADAWVQEFMRHSIPCISADYLVEYVCKPGHPLGKHVLFNMHDLAERSLQKLMKN

QEDAPGSCSACGSDNREVPMLVCGGDGRSQSSGCGVRTHADCCNPPVEGCDGGEWLCGRC

DQQKSAKKAKKTAAKPRVLKQR

>Bstacei_BCP1

MHGSDDDVGGDDDARLFAGVRFALVGFDSVSESQYRSEMVRRGGADAGGHGAAGCTHVVV

CGLVHDDPVCVAARESGKKVVTELWVDDSLDMGVRANADRVLYKPQRDLDGIPGSQSLNI

CLTGYQKNGREDIMKMAALMGANFSKSLVADTSTHLICYKFEGEKYEVAKRVNIKLVNHR

WLEDCLEAWEILPIDNYTKSGWEQEMMETQVEDSEDEAEDVGRGLLHSRVIPRSVPITKI

RTATHVDPDRRTPIRGPTVSTGNAQVGAGGHMDTPKQVTEAEDVSKRSIDTRADVQSTHN

TNGVTSSADPEAHDSVHPPVNPSSNEKAPGGHIIGDEAKDGDKRAVDTTASTLSTLNTSG

ATVQADHLVHQPIVTPSTESPLKKTLHSSHMCEKVDQKDDEPVADLAAKVVQSNVQGNVT

SCKVNLTSAGNSAPKNTPVLSYSSRRSRKSVSPGANLNSVHQTASPQSSKTSTLNVELNS

SPSVKSNHKISKHTDAKSLQDKACALAQRKNRLSSVSPIPPNGSTDSATGTANSPFSSKE

IASEAAAVSDLVKRSTGSQPVKVDSNINSNATGNLVERQKSGSPKKKLSYRGTSLKLARS

SEVEKLPESFANEKILESLAKAKKHPRHEAAVEKGCAISPSVDSELGNASSSPTLRNLGI

EMGETPQVNNIEVASPNLQHDKVVSCATMQSGAHKASTSGVKKAGAKRSRNAGNETHTVS

VDGESETAASKYKHDEVISHEDVEQEAEKDCTSRNAAERTTLFPDKSLSSRARNAAANSS

LNENSEMNDLIAASNMKTPLGNINKNHKQLSSGASAHEYKERSSKKVPNATETNDVANVS

RSVKIKMTEAPSADKTKAGSLISSLSGVISQAYTERLSSNGSADKHEICNPDKGPGKRMR

NAVAKRKVSAAQQHRSGSEPCKTGGVFTSDAEVITSKAAADSSGNAKKITVDQDVQNANK

DAMTNADGSFCKESSKVASKDLQKSKLKSSKRKFLAVVEDGSTNRNKENMQVNANLTPKS

KCGNNTKAVENIKDVLDDPSIREGNDCTTLTMLEPMRFILSGHRLLRKEYRLILRRLKGR

VCRDAHHWTFEATHFIAPELRRTEKFFAAAAAGRWILKSDYLSACNEAGKFVEEEPFEWH

GDGLNNGETISLDAPRKWRHLRLRTGHGAFYGMQIIIYGECISPSLDTLKRAVRAGDGTI

LATAPPYTRFLKPGVSFAVVSADVPRTDAWVQEFVNHNIPCINADYLVEYVCKPGHPLKKHILFDMDDLAGESLQKLQKTQRDLGAGTKEATGGGDNTELSCSCGSNNREGSQLICSGGDGSRAVCGAAMHVDCWNPHHPEPVPDGEWLCPKCDEHAEPPKKTKKTSRSRVRKCR

>Osativa_BCP1

MASPISGSDDDDEHLFAGVRFFLVGFDPLSESQYRSEMVRRSGVDAGRFGSGCTHVIVCG

LVYDDPACVAARAEGKKVVTELWVEASLDRGMLADADRVMYWPVRDLNGIAGSESLRICL

TGYQRSDREDIMSMVSLMGAQFSKSLNPDVVTHLICYKFEGEKYEAAKKAKLKFNFNIKL

VNHRWLEDCLKCWKILPVDDYSKSSWELEIMEAQAKDSEDEEDVGQRSFRNKIVRSTLNP

KGSAGTSANPVVNAPIRSPTISSGNIGMVLEKQPNIPGEIRKAEDAVNRIHDVAVQGTPR

TRLAMSANTDFSTPSQIPFIHSDSRGDAAVRDLKNADQIQGNKHKDVGTKTLDVTSGASG

TPCSSKMVVSANHNVHSLNKTNFVEDHGDTDASKADLTTPSREILPANVLDSSNVARGRS

QEDYGATCTPDAAAGQSTINDNVTNNNIGLKSGNNASLNINNKSSLKPLEKSVLPEQYSA

NRIGPLQGADGSMMRSDSSISTARKGDKIIADLAGVQVLKGGENIQDENVLDGAYSQKKK

CSISPVLFKVQNGDTGKETGALNSPSANRLSDTSEPAIWSSVGTNPSEANNVDLGKQQSG

SSKSRSRTALKHGNLVDGIKLPEYSSSETNAQPPPKPKELLATSLSATVHDVKRCPDFSF

QNKDGDYAQDSGNALNQDGSPLMRKTENVLAKVRTSDISLHSSRKSKLVPSSGNGDTEMS

DALDIEKNKAAVASNCKPEKVVPDENIKADQLKDFPGTSNNVLRQTGYLKKVASRKGMKA

STKRPRSASKVVDEPVVDDGKTKTVVSESEPDKMIAHKHIGETAKDGPDSVNAAEHRTNS

SDKVLTDGASRISRRLQNVHTMKNDRHAASNLESSKMISEENTGIGITPKKFVSNATTEG

HQTNSPKMLPNTSMRNTFAKRSRVSDTKMTGESSADKTETVAGKSLFDDLFPSQNIDHPK

KLSSSASADGCGSLSCKNASPARVRNAVAKRKIKALEDKSDSKLGKIGGAIVSAAKAVAS

RGIEESSCNINKVSSDQNSVKTDGMRDVSGLFSSDTSVIDRSENLNNSKLRCSKRNKSLS

LDHEKENMQDNGTLSSKSNGRTAIMNSNLDANSMKHGANMFNEPNRIKGNGPGTLITPEP

TCFILSGHRQQRKDYRSILRCLKARVCRDSHHWSYQATHFIAPDPLKRTEKFFAAAAAGK

WILKTDYLTSCNEVGKLLDEEPFEWSGTGLNDGETISFEAPKKWRVLRQQMGHGAFYGMQ

IIVYGQLVSPSLDTVKRAVRSGDGTILATSPPYTRFLNSGVDFAVVSSAMPSADAWVQQF

ISHDIPCITADYLVEYVCKHGHPLDRHVLFNTNDLANKSLKKLLQNQQEVATDVLKPQED

GDPDDLSCSACGSTDRGEVMLICGNEDGSTGCGVGMHIDCCDPPLEAVPEHDWLCPQCEM

PKATKKSASRVASKSRVSKRKR

>Macuminata_BCP1

MHRSHGSSSSSAPDKIFGGVRFVLFGFDSVSEAQYRSELVRRGGIDVGRYDRSCTHVIVS

GRVYEDPVCVAARNDGKILVTELWIDVSLDIGMLADAKRILYCPVKDLNGIPGSKSLHIC

LTGYQRQERDDIMKMVSLMGAKFSKPLIANQVTHLICYKFEGEKYELAKKVNIKLVNHRW

LEDCLKAWAILPIDNYTKSGWELEVLEAEAKDSDEETEDVGKRSVGRSGMGKSISDGGVL

SGDSDLSIPSRGKLSIPKDTFMTIHSAFADNHIMNNTLFTSPCTDNFSRNTLSECSPGSK

VHHQNVGDVAQTSNDHAIRDAMVGGELFNQSDSGSLKEAYPLSARTVSTSSMVGVKSVPF

SKKSRNKSVSPEESLMKVHTSPKGLYKDFDVLSSEPKGSRATDKPANIQGSMAHIGINNV

EATSSVLPQKRKVSVSGGGSKSPKSLMHSSLQRKSPDTESAQVEPVLLSPKENKYLVPMD

ANHSLEDAVPSNSVNQQTIVSRSFSCKRNSLSNGKPAMIKSSNVIEHSIPILKQGLDKQE

CRKVDLFNMAQMATNVIASMDSKALPNIPVDDVLMQVDDCEIMRSTANQFGSLAVGKHTD

EMLCNDKRPEISVGGPVEAERSTQSLNGETARIEIQNVMMKELTQNHVLQQFEASLSSDG

KAMTTRNFLTTASADACKGNNIKVASGSYIKKVVAKRSLSSSSKHNALSSRKDKHVDNPD

KYLKEAEVISGKVEKMKVPEDKKVSRNLRNGVVETFSDEVMKERVEAAGVSIPANAKKDA

TSDRKDITSMDPEKENKPEEVSRFSSNCSNSCSSKLVNKHDKKFIQNSNNSQKIEIRKTK

KIIYLEPAWFILSGQRIQRKHFQAVIRRLGGKICRDSHHWSYQATHFIAPDPVRRTEKFF

AAAAAGRWILKTDYVTASCEAGKFLEEEPFEWYRKGLTEDGAINLEAPRQWRLLRERTGH

GAFYNMSIIIYGECIAPTLDTLKRVVKAGDGTILATSPPYTRILKAGVDFAIVSPSMPRV

DSWVQEFLRHEIPCVAADYLVEYVCKPGYSLEKHVLYKTHSWAEKSLAKLLSCSEEISED

ARLLSEESSDDLSCAVCGSAGRGEVMLICGDEAGAVGCGIGTHIDCCDPPLDSVPEGDWY

CSKCSSTHNKTTPKHARFR

>Zmarina_BCP1

MPAVGGSEKEGAIFSGLRFFLFGFDPVSESQYRSEISSRGGVNVGKYDKCRCTHVVVSGR

DDPVCVVAREDGMALVTESWIEDCVDFGTIADASSRMVEMMGANFSKPLIANKITHLICY

KFEGEKYELAKRVNIKLINHRWLEDCLRAWKILSVDNYAKSGWEMEILESQANDSEEETQ

DEGARCSLVKDIIADATRQNDIHRVGGSDFSTEVLKTYTREKDPDGTFNENSLLSNRSLP

STSNNRSKNINMPIYGSKDSKKGNFILPNSDEVIDLEKEDNEVLLDMRSTERKTSPLASF

RKTPMKTEETSGHFFGSLKRNMFGIEDSAFSDFENNTHHSIINQIGTDLHPSDSSVKKRE

ISSPGIGTGSSELHQNSKSDGHCWSMSNKSTTKFRDTVTVPNRSTSTNEDAIDYYTVNHS

TPLVGTGSGNVISDLNVRSSKSKTQTCRSTNVGVLPINELVHVPEKSITVADLDWTTSSS

ANLSVLVHKASDYTAPPTCVKNTVLADGLQTDKNHAESDKDSLIPNEREVHEYSPLGGEY

GTCSNKSKKSQLASISGRLDISKKHSINLPIEASDQHQKQYEKSSPPESIKSDNTRLQNG

TNTEIVNGKTENIKTSSKKVFLKKKLCSKVRPSKRKFTEDVTSVCLNKNMNGSEELFMSR

DVELGKVENGGKSLMIDSKTVVEDKLNVTPLISSSCDNDNVSIDLTVKSKDSGRQNPAVE

IKSWSVKLDKYCSNSVGKDSGKSKQMTNIRSTIDNETAACFDENPTGYKESFMSVNMQHE

IAIIDEQNLVIQPKIVAEDNSNITPFVLSRFKNIEVGSDMGDTLKVKGSTVKLGKSCLKK

VGKENGKSKQRPNKRLSNINETADILDKNLTGSKESLVLENMEDEMPVIDDQNLVKKTEI

IVLKSDKYGSERKVKVDGKIKQKANKTQHIEVETASCLDKNSTLNEELSVLENMEHEVKV

IDEMNLINKTKILAEDKSNITPFASNRHKTIEFRSDMSGALNDSDKRKSVFEMKGLNVKS

DKYDSKRQVKVSGRFKQKAHKRKQIETTLCLDKNLTSNETFMSENIENEMLVFDKQNLVT

KPKNVGEGNINITPLISNRCKNIEIRSDISAAIKDLDKQKHALEVKGSTVKSGKCCSKKV

GKDSGKSKQSLNKRLSNNNETADVLDKNLTCSKESFTSENMEDEMADIDGQNLVDKPEIA

VEDDNNVTQLISNRYKNLKVVTDMDGSHGTSKDLDKQKSVFGKKGLSVKSDKYGSKREVN

GSGKSKQKSNKRQRTEEKTAAWSDKILTCNEVSFVSEKTEDEKADNDEQNFVIRQKIVVE

DNSYIATPLGSNRCQNLEVGDINGTIKDSDKQKPDIENQGSTVKLNKYGSKREAKVGGKP

KHIVEKVGSNVGNQMKTFLKKTVEPVWFIFCGHRWQRKHFKQVIKRLKGKMCPDSHTWYH

KATHFIVPEPVRRTEKFVAACAAGRWILKTDYLSASNEADKFLSEEPFEWFDSILTEDAN

IDLKAPRKWRHLKEKTGHGALYGLRIVIYGECIAPSLDTLKRIVMAGNGTILATSPPYTR

LLNSEIDLAVISSGIPRVDLWIQEFMRFEIPCVLADYLVDYICKPDHSLDRHVLFNTHEW

ANKSFEKLTIRSEEIITCNSSDEMLSEDENEIKCVVCKRHDRGEVMLMCGDEEGSIGCGI

ATHIDCCVPPFESVPKEDWFCEKCSERKNTTTLSKVKRRKTRKCSSGNSHQSPCNKL

>Acomosus_BCP1

MVRSRGESSSSPAKIFGGVRFVLFGFDSVSESQYRSELVRRGGEDVAKYDASCTHVIVSG

RVYDDPVCVAARNDGKVLVTELWIDDSLDLGEMADANRVLYRPIKDLNGIPGSESLHICL

TGYQRQEREDIMKMVSLMGARFSKPLIANQVTHLICYKFEGTFANVAFLNNLLISVNIRFPCCSLKEWSILPIDNYTRSGWELEILEAEAKDSEDEVENPGRKLLNSRNSGETTTIQSGTIVSTGPDKPIQSPGNSNIVLANQSNNANALATSSNHFTTPSKETSSGKAMDLYNSSYKLENSHQKDMKGEVHTNGANGDELLARCKTGLKAEADILPTISRTPFTFDEKMNKLSYSRKHSKKSVSPEGISSYQNASDCRLDTDMMEFDYNISPLERREGRSGEPASIQTPIDTEKIKHIEGSANTLPQKRRLSISTSGSKLPKTEYQNTTTSSPSCHSEDKESKNMEPATVMPLEENVAGISKISNLSEDDGKTNSPDNQHCISARSKTLSYRKNSLKRGNPILGAKIIKHSPSHAKDVELSKPTQALDECSIDNLANIKLAVTDAKVSSVDGKVTLHEEQMEEADMPLAARIADDKLWSLPFEKQSDSAASMHKVPEIPKFELSDDFGSGDVERSTTPKVSCANAEGNILQEEGTHSRNILQEEGTHSKKAVAKRSINGSKNIVKRSISGGKNKLCVESGRGGEVAVASDKAVDGKVTLHEEQMEEADMPLAARIADDKLWSLPFEKQSDSTASIHKVPEIPKFELSDDFRSGDVKRSTTPKVSCPNAEGNILQEEGTRSKKAVAKRSINGSKNIVKRSISGGKNKLCLESGRGGEVTVASDKAVASSKPEIKSVSEQTVNDSLKLEQAVSKEASEAVELYKRDAALKDKQEVLKNNISGRHKKTLAIKQKKKSLDPGKENNPEGYCSVSFESKGGEGGNLVSKSDEKSAENGHVSGGHEIDPLTNKAGKVASLGPAWFILSGHRFQRKEFQAVIKRLRGRLCRDSHHWSYQATHFIVPDPVRRTEKFFAAAAAGRWILKADYLTASSEAGKFLEEEPFEWNRKGLTEDGAVSFEAPRKWRLLRERTGHGAFYGMRIIIYGECIAPTLDTLKRVVKAGDGTILATSPPYTRFLKSGVDFAVVGPGMPSVDSWVQEFLRHEVPCITADYLVEYVCKPGYSLERHILYNTNTWAEKSFANLLNRSEEIVVDSAMPLEEHGDGDDDDDDDISCTVCGSRERGEVMLICGNEGGTVGCGIGTHIDCCNPPMEAIPEGDWFCAKCSISRAKRALQRTKTKAQFFGLGPELVLLDLPCRRLRQRPENHRFR

>Aofficinalis_BCP1

MLKSRSSSSSSDKIFDGVRFVLFGFDSVSESQYRSEIVRRGGIDVGRYDDSCTHVIVSGR

IYDDPVCVSARKDCKTLVTDLWIDDSVDFGTLADASRVLYRPVKDLNGIPGSESLYICLT

GYQRPERDDIMKMVSLMGAKFSKPLIANQVTHLICYKFEGEKYELAKKVKIKLVNHRWLE

DCLKAWDILPIDNYTKSGWELEVLEAEARDSEEETEDVGMELMGQRNTGGAPCEQGQMSV

AASSHVHTPNKGSLSIRQEGSIANRSHSVIARSHEHTPNRGSLSIQQEGSTANLPHSAEN

LPADQWLSTPYKETSFEEALSKQNLKSNVEIDHQEAGGVARKSDNNAMKDGELTVPVDVS

LQKRDNITLSDVLTTKIGSVSSRQERSSTLCYSKRTPKKEIAREKPSDSCRTELNENILN

GELDISPLEGRSRLDHFQFPTASCVLPQKRKVSDFSSIKSSELEVHDPKISPKSPTNTRF

ERLKPTSATSIHYGHADASSTLEGAAKTNSEVYQHGAPIKLTSLSYNRRSLKRGQPTSES

KFSRNADSPSTGCTQNCQQGEAWDQPSGVKVKLAVETKELCPSISPSEDRVLEDMAPSFK

PKNTMEADAGFGNVVSGVNNLASAGRNNDVNPIEEMASETSGGSRQSQEIGKSENDGDIV

MRDNSTFMANETGVPLGRQHNGGAASPSSRKTRKLSRTASTGTCKENVNEESKIVHRKKV

VSRRNLIWKPQLTTVNTLKGKDASTLDKATAPRKVESKPGKAEKSNSDIELGMEAVNDNK

EATKSFSKDVVMEHQTKPKRDPLDQTEASVDLEKDNNPKENATLASNVNAHANSKVPCED

GRKSRQGTKRVRAKNCKKELVGQMEAAMDPEKENKPKENGTLASNVNSHGKVPCDDRNNS

RQGTRRVGAKTNTDQTISCTRTVNPEPVWFILSGHRFQRKEFQAVIRRLRGKVCRDSHNW

SYNATHFIVPEPVRRTEKFFAAAAAGRWILKMDYLTASNEAGKFVNEEPFEWYGNDLTEH

GAISLEAPRKWRKLREKTGHGAFYGMCIIIYGDCISPTLDTLKRVVKAGDGTILATSPPY

TRFLKSNVDFAIVSPGMPKVDSWVQEFLRHEIPCVAADYLVEYICKPGYPLKMHVLYNTH

SWAEKSYANLLRRSEEIITDDCSPLEESVDDISCSVCGLNDRGEVMLMCGYEDGSVGCGI

GTHIDCCNPPLEAVPEGDWFCTKCSRDREPKIPLRIKKTSSKCK

>Tintermedium_BCP1

MASPGGDDDFDDHDLFAGVRFFLVGFDSVSESQYRSEMVRRGGADAGRLGGGCTHVVACG

RVYDDPVCVAARAQGNKVVSELWVDDSLDRGFLADADRVIYWPTKDLNGIPGSDSLQICL

TGYQRNDREDIMKMVSLMGAQFSKPLLGHVVTHLVCYKFEGEKYELAKRLGIKLANHRWL

EDCLKSWEILPVDDYNKSSWELEIMEVQAKDSEDEVEDDRKLFNNRSGVQRSPNPKNSIG

TSRNPEVDVPIIPNDNQNMAVDRHLNTPGQIRKEESVVNKTHDITAEGTPNISRLASSAN

TDSGAPVQTPSVISGNREEVAVRNLNGPNQVQPAEYKYVGTDIATGALGTPSSSRMTVSA

NHHSHSPDETLTVPKHIATPKAMNAGAHSASMEVDGSVVNNGNAEVSESGIEKTTPCQYA

GVTSKKDPSSASTTEKQTTSPKKAPLSRVASALAKKQQSVPTRLNDAHVGSNLELTKVTS

EENIETHSSDEALMLLKNKATPRAGNAGAKRPRSANVEVDGSVVDNGKAVVSESETDKMI

PHQHAGATSEKGTSSASATERKAMSPKKVPVGGVRKGALAKRQQSVHTKANDAQVGSDLE

LSKLTSHENIEIHSSSEAPMVPQKIATPKARNAGAKRPRGASMEVDVSVVDNGKAVVSEL

ETGKMIPRQHAGATSEKDTCSASATERKTTPPKKVPVGGARKSVLAKRRQGVQTELNDAQ

VESGLELSKVISQENIETEPKRFCSSANDDEHGRKSPKKLPNTRVKNTVVKKSRKSDTST

TIEPQVDKAETVPAESLFDDLFPSENVKDDPKKLSSSASVDGCGTVSPENILRTRVRKVV

AKRKIKNVEDKSGSKHGKIGSSIVSAAKAFSSERTENACNIEKATADQDSLKGNNDGVRD

VSVLSCKDTVTVDKSEGMHNSKLRSSGRNKAPASDHDNDNQQDHGDLSSKTSCGNGGLNS

KFALKSKKNKTDMLDKHGGIDKSEGMHTSKSRCSKRNQAPASDHDTENRQDHGSKDTVTV

DKSEGTRNSKLRPSARNKAPASEHDNENQQDHGGKDAATVDKSEGMHNSKLRSRGRNKAP

ASDHDTENRQDHGSKDTVTVDKSEGIHNSKLRSSARNRTPSDHDNENRQNNDLNSKFSLE

SNCGNGNLNSKFALESMKNNSDVLDKHGGIEGNGAGTLITLDPAYFILSGHRQQRKDYRS

ILRRLKGRVCRDSHHWSYQATHFIAPTPLRRTEKFFAAAAAGRWILKSDYLTSCNEAGKL

LAEEPFEWSGTGLNEGEMISFEAPRKWRVLRQQMGHGAFYGMQIVIYGQLIAPSLDTVKR

AVRAGDGNILATSPPYTRFLNSGIDFAVISAGMPSADAWVQEFIRHDIPCVSADYLVEYV

CKPGYPLDTHVLFKTNRLANKSLDKLLKSQQELATDNLEASEDDGDDDLSCAACGSTDRA

EVMLICGSEDGTVGCGAGMHIDCCDPPLDRVPDDDWLCPKCEAPKAKKKPPRGAASKSRG

SSKQRR

>Msinensis_BCP1

MASPGTDDDIGDEHLFDGVRFLLVGFNSDDASQYRSEMVRLGGADAGPSGNGCTHVVVEN

RIYDDPTCVAARAQGKKVVNGLWVEDSLDHGVLADADRVMYWPMRHVKGIPGAQSLLICL

TGYQKSYREDMMKMVSLMGARFSKPLIANAVTHLICYKFEGDKYEVAKKVNIKLVNHRWL

EDCLKAWKILPVSDYSKSGWELELMDAQAQAIDSEHEAEASYPGSLNNRPSVSVNREVVA

RRNVNTPVHTINTEDANNKTHDIRGQSSANSMVAVSAKVDVLAPIQSPVHIMNIEDADSK

TNDITGQDSPDSGLLASSAKADILAPIQSSGEKRDIVRNTNSPNLQEVKEKYAGARTQDL

ASGVLGPPSTSKITVFRNHHVDTLNGTLGILKGHTDHVSGKYSASHDQTDVAEVLLTNPL

RGNQSVDDLDSSKVDRWQHQEKDGPSGIHIAAAGQLNADAKLNNHESNRKPGGDSMSNNI

KNTSNDKKASQKSFLQEGHSVNHMASPQRAEESTLRGYPNISTLETGHHKVVEHADVQSI

EGNENTKSEDGLDGAYAQKRKSLVSPASLNLQKEDLVSETGPLDSRFVSRLSDASETANV

SSERINLVEANAVNLGKQHSSFSASRQTRSRKTSLKHGGPISGIKLPEYSSSDKNVKSLR

KARMSFKATAESKCTMSSSATVQDGKTSAGFQFQIKDGESTQPSGDALNEIGNACTKDQA

HEKSVHSSSNSQVVPSYGNAGNRISDPVKVNGNEVAVASNSEFEKVVSDATVKESTKQFQ

DTSRNVQAETSYSKKAPTTIRRNTGVKRHRSANIESEGSVINSDKKVVAESSPAKVIHHE

HADPVSKNGYSAACAAELKTNPPKKAPICRVADTVAKRTRSACTKIDDARVGSSLEFSKV

MSQENIEINPQKNLDTANAVEQQRNSPKKIPNSRVRNRAAKRSWKSDTNMSNDTLMVKTE

TVAAGSLFDDWFPSGNVEDEDWPKKISSYASVNDCETLSPKTVSNARIRNAAAKRKIKTV

EDKSGGKFGKIGSAITSETKAFSSKRTEGIACNINKVTADPDSEKSNKDVRREVSGLFCQ

DSCTIDKQGPYNSKLRSSKRNKALTSAHEKENRLGCSDLKSKPNRTGSLCSISDAKSMKK

STLVLSKHQREKRSESGTLIMSEPALFILSGNRQQRRDCRSILRRLKGRVCRDSHHWSYQ

ATHFIAPDPLRRTEKFFAAAAAGRWTLKREYLTSCIEAGKFVDEEPFEWFGTGSNDGETI

SFDAPRKWRNIRQQMGHGAFYGMQIVVYGQLISPTLDTVKRAVKAGDGAILATSPPYTRF

LDSGVDFAVVSETIPRTDVWVQEFIGHGIPCVSADYLVEYVCRPGQPLDRHVLFKTNHVA

NKSLEKLMKSQQEMATVNSEPSEDNEGDDPEDLSCSACFRKDRGDVMLICGEEDGEIGCG

IGMHIDCCDPPLEAVPDDDWLCPKCAALKSKAKRTTRGTERKARGGSRRR

>Sviridis_BCP1

MDSSGTDYDIGDEHLFDGVRFFLVGFESDVESQYRSEMEVRGGADAGSLGNGCTHVVVSN

LFYDDPTCVAARAEGKKVVIDQWVEDSLDRGVLADVDRVIYWPVRHSNGIPGAQSLLICL

TGYQRNYREYIMKMVSLMGARFSKSLIANVVTHLICYKFEGEKYEVAKKVNIKLVNHQWL

EDCLKAWEILPVDDYSKSSWELELMEAQANDSEHEAEAAGPRSLSNRSSVRCTLNSKNCK

ETFVKYDVDARKRSPVIPSGNKEVVVGRNLNSPDHILKTEDADSKTRDITGQSSASSMVP

VSAKADVFARIQSPLGHIMNAEDADSKRHDIRGQGSPNSSLLAVSAKVDGLSPIQTSLGL

SQKRDNSVVRNNNSPNLQEAERKHVGARTQDFASGVLGTPSSSKMTAFSNHHLDTLNKAP

GILKGHTDHVSGKSSASQDHIDVTKVPLSSPLRGNQSVDELDSSKVDRWQRHEKCAPSGI

HITAAGRSNTDDKLTDHESNPKSGGDSKFNSIKNTSNSKKASRKSLLPEVHSVNHMQSPK

RVEDSTLRADSNISSLEMGHQKVFENADDQSKKGNENIKCVDGLNGAYAQKRKILVSPAS

LNLQKEDLVSETGPLDSPFASWLSDASDAEANAVNFGKQQFSLSTSRQRRSRKTSLKHGG

LINGIKLPESSSSDKNVKSSLKARMSLKAMVENKCTRTPSPAVQDGKTSFSFQNKDGEDT

QGSGNAVNQDCLHEIGNLRTKDQAHDKSVHNSSNSHVVSSSGNVGTKVTDPLKVNDYEEP

VVSNSELERVVSDANVKDKEDAKRLQDTSSNVQGETSYSKKVATPERRNAGAKRPRSASI

EAEGSAINSGKKVVTESWPAEVIPHENADPASKNGCTMASAAELKTNPSKKALICRVTDT

VAKRTRNACAKTDDTQVASSLEFSKVISQENIEANPKKFFDTGNADEQQRNSPKKIPNTR

VRNTAAKRSRKSDINTSNEPLVDKTGTVATGSLFDDLFPSDNEDYPKKLSSCASASDCGT

LSSKTVSNGRTRNAVAKRKMKTVEDKSGSKCGKVGSVIASVAEAVSSKRTEEISCNSNKI

TTDQDSDKSNKDVIKDASGLFCQDSGTVDKQGGSYNFNLRSSKRNKALTSDHEKENRLDH

SNLNSISNRTSSLQSKFDAKSIEKSTRVLSDSEHQGVKVSESGTLIVSEPALFILSGNRE

QRRDCRSILRRLKGRVCRDSHHWSYQATHFIAPDPLRRTEKFFAAAAAGRWILKKEYLTS

CTEAGKFVDEEPFEWFGSGLNDGETISLDAPRKWRNIRQQMGHGAFYGMQIVVYGQLILP

TLDTVKRAVKAGDGTILATSPPYSRFLDSGFDFAVVSASLPRADAWVQEFIRHGIPCVSA

DYLVDYVCKPGHPLDRHVLFKTNDLANKSLEKLMKNQQEMATDMEQSEDEEDDPEDLSCS

ACGCKDRGEVMLICGDEDGETGCGIGMHIDCCDPPLDAVPDDDWLCPKCAVLKAKRKPTR

GTERKARGSRRR

**EUDICOTS**

>Acoerulea_BCP1

MIRNKEDSPQGGGGSSSPSKIFLGVRFVLIGFDNPTQQAKVRSKLISGGGVDVGEYNSRC

THLIVDRVVYDDSECVVARADGKIVITAAWVDDCLDVGMLVDTSSILYRPVKDLNGIPGS

KSLSICLTGYQRQDREDIMRMVGMMGAYFSKPLVANKVTHLICYKFEGEKYELAKKLKKI

KLVNHRWLEDCLKAWEILPENNYSKSGYELEIMEAEAKDSEEETEDRNNEQYKENVAGSP

YNLHTVYGMGIKGLEMPVLMKQNESAKENLAARVEDIVNEKRLVSPAKKARSDHVPELQQ

GNKSGAAFTCQGTGSYEGTNASKESSELSSNPTTSKGTPQSNVAAPISLSYSRKTPGRSP

LSNFSGMPSSPAVSHGVEQYKVTNGFDVSPSKLVQYKGNLDSVENQNLRKDLELHLEGGN

AYKLPQKRKASLSSSSSKSPREISANLEACGPGSPSTGARTGILLNGTFQKNSSSCYGNG

DNVDKIDIRNVASDHYGNPSNGKSISENKLTNPGMQFSRTLSSDDTEFSSDFKMLERSPE

ISDVGLEHSKNTFEASFSGKDMPRVLDFQFDNEGGPQEECQSIMASSLKSENCEAGKARS

PVDLSVHNGSKSETFAKSLPKKTVVKKSLGSKSKLSSARNKQKGSLCFNSKSEVIFPTEE

NTEQGHGKAVGGKAEMDKPALDAEPVFERMVTGTLSSENKFKNADEAEDEETEAPDKGIG

GELENSAREEVAKPIPQENGTSKKKSKVTHSAANETKSVKVTPEKDEREVVKGDTCKSVA

VHGTNADPKQIVGGSKIRKKSRVTHSEANETKSVKGTPEKDERDVVKGDTCKNVATVHCT

NADPNQLVGGSKLNKKSGVTHFAPNETKSVTVTPQKDEREVVKGDSCKSVAIGNGTNADP

NQIVGGSKIKKKSRVTHSATNETKSVTVTLEKDEREVVKGDTCRSVTTVHGTNADSKQIV

GSSKIKKKSRVTHSAANEIKSVTVTPEKDEQEVVKGDTCKSVATAPGTYANLKRKVGGSR

LVKSEPVWFILTGHRLQRKEFQQIIKRLKGRVCRDSHNWSYQATHSIIPDPIKRTEKFFA

AAASGRWILKTDYLIASNQAGKFLREEPYEWFKNGFNEDGAINLEAPRKWRLLREKTGHG

AFYGMRMVIYGECIAPSLDTLKRVVKAGDGVILATSPPYTRFLKSGVDFAIISPSTPSDD

MCVQEFIRHEIPCIVADYLVEYVCKPGCSLERHVLYQTHAWAEKSFANLISRSNEVVEDL

IPVEDDVRIDLCCEKCGSFDRAEVMLICGDETGSLGCGIGTHIDCCDPPLEAVPDEDWFC

PNCTEGKNSTHPTKKTKKGSSTKKHK

>Klaxiflora_BCP1

MMDGSRMELEPGGSGSRPFDGVRFMLLGFDVGNESEVRMKLISGGGEEIGRNNSDCTHVI

VGECVYDDPTCVAARSHGRIVVTGLWVDHSYDIGMPVDHTNVMYRPLKDRNGIPGAKDLV

MCLTGYQRQDREDIMIMVSLMGAQFSKPLIANKATHLICYKFEGEKYELAKKIPKIKIVN

HTWLEDCLSAWKLLPEDNYAKSGFELEMELEAKDSEEEENPEGSITKQFKGRINVPYGTR

VETGGVSKLAASQAMNGVNGTEGSKFLPRDKEVTFAPGEHDNKFDLVIGSRSSADVLKCD

PGNQSLEYRDANKDSKPKFPGIVEKSDEPVQSPWKEKRNVLGDSSGSFGSAKKFPESGMN

SSNTLRFASKTSQSFSLQMDLSGEARYSDDQLAKGQAFDASASLPKSEQRKESDDAMTCI

RTSSLSKEAHDNVLPSKRTQSATHDRVKVPKSSRAWTPIAPRRPVVIADNIVLTPTDSGF

VAIKRQSPSANKSTPEAQNLLKFDSLNHKGELTDDVTQQTEMTTGVQKETKCTDVSASAT

IMERSKNESEETTGQKHDIVTSPPRLMPQISNYRHNATLGSGANIKSKLIENLVSGKCSG

SRLNFEADSLSSETKNAEDQKNINPPSSENVNLQLKPGRRMGLGKTISGFNLNPEMPSHS

QEFGKSKEPDLLVEDCNTPNSKSPGVDRNGDKSVGTRLRMVARAMSESKVPDMLVEKSYK

PNSKPLVKNKTGDKSAGTRLKSMAKAASEKCSPTMTKISSMEASNGQENDSTHDAFHKGS

STNKVSSENDGLEMQRVNGSKLIDGFEEPCGPRVDEGRAPEKIDDFETNKSEEVTENRGK

QGENNSDENIDPALSFRMVVDNNLDGEEKCSQEKELGRAPLKRGVKRGKRKKGTQGHSRS

ANNAKTAASLKKLGKNCTDTVTAGNEDLPKVGTMDQQPSNSKAQKHTVKANKLKNVSQND

RPVPDAEDGNMDERILQPPNKNVWKHSAVANQLKNVSDTGKENKSIPDDPENTRFTNEHA

VNTAITSEHTPHSGKRKASAIKYYSTDKAASKNMSKSELKCFLLCGHRLQRQELKKIVRK

LKGRLCRDSHHWSYQATHLIVPEPPRRTEKFFAAAASGRWILKSDYLTDSLKAGKFLEEE

TYEWDSNDASEVGRVDLVAPRKWRHLRERTGHCAFHEMRIIVYGECFSPPLDTLKRVVKA

GDGIILATAPPYTRFLNSGVDFAVISPGISKVDAWVQEFLNHEIPCVLIDYLVEFVCKPG

SPLNNHVLFETESWAERSLANLVRRSEEIVEDSTTTEEDDLKCCVCGSGDRGHQMLICGD

ENGSVGCGIGTHVECCDPPLQEIPDDDWFCPKCSLSKDAGMKLKSGKAKKAKTSTVKSR

>Pamilis_BCP1

MLGGRSHGSAGSFSDIRFLLFGFESPQLEQAKSKLVSVGAVEVAKYGPDCTHVIVRDTVY

DDPICATARKDGKKVVTNLWLDHSLELGEPVDPANILYRPPKDLNGIPGAKDLVMCLTGY

QRLDREDIMNMVALMGAQFSKPLVATKVTHLICYKFEGEKYELAMRVKKIKLINSLWLED

CLKAWKLLPEEQYCKSTYELELMRAEAKDSEEETGVVVGNLRGNVDTIPPLQNEKAVPQK

TTSPANVIRKIKEEKISDQKSMPGGFDRTGQQSPVRHREPGMTSNSDVQCQDNNVGNDLL

FIPGEVNIGNAKEDKISGSGSSGMSPTKKSSLQLNVGNDVGNSSESSSKRVAGGSDIGKG

SPSVRKQAAGKFDGSVGGVEDNLAINDICPTNLEPDKEFGASTSVGVRSPDQSLDSDSGK

GGFLSSSKTPRRSSLSFQSVEKMQDTNRKAGEASAEKSVDACTFNMEQDKGRDVSAYVAA

SSIGNGLFDEEGLDGLGGLLPQKRKVDDHSNVSPVTPKSRKLASSASKRIDATDPKTPVT

SPLGKSKDVSLNPSSYIEENTGISPSCHKLSPTASSVKSKAKSASASRNLASEMLQSRKP

AKKAAAISSNTGSVSTSTALLVDSKKPELQNQQQNASSNSCTKDSEAARSGSLKLKSDGQ

IGNSISKPVRKKTVARRTLGARPKLSKKSTAACENSPIENPVQEDAPVHPSTGQEGHAKS

LSSDKPVVSPPTLDVPVDAGSKPQTFFSVFDAGLLSDETEPPAELEPQNENSVAVELFSA

ETRNRGRSRVVLSESDEEDVTTSPNKKRVEGVTVDETGNAKKKLVRQKGLSGKSNEKSVL

FSKDIPNGTNEAELGVEREKVECIVADKNCDALLMSESKRPEEMAEETVTIVPNVKGTSK

GVVSKKGTSKGDKHTRKLTGKKRLGTLILNRPEGSVEVGRENEPDLAGERDQSKKENIPR

KLSAKSKPTNEPVEAEKENDPVVANNAGTSKSSKLAGKLAGKKKRGTLVSNKPVGSPDEE

EENGVVLSDAQGGNNGDNCNDKLSGKPSKKMRKSDEMSVSTKPSGSRVVLCSMKDESARF

LLSGHRLQRKEFQQIIKRLNGKLCRDSHQWSYQATHLIIPEPLRRTEKFFAAAASGRWVL

KSDYLTECSQAGKFLPEEPYEWHRNGLTEDGAINLEAPRKWRLVREKTGHGAFYGMRVVI

YGECIAPPLETLKRVVKAGDGTILATSPPYSRFIKAGLDFAIVSPGMPRADIWVQEFMRH

EIPCVAADYLVEYVCKPGYPLDKHVQYNTHEWAAKSHSKLLSMSEEIIAEAVAQEDDGDE

DDIDDLACQVCGSTDRAEVMLICGDESGSSGCGLGTHIDCLDPPLESVPEEDWLCSKCCG

SSSNKLPGSVSSKKKVSSKKSRR

>Lsativa_BCP1

MENHQNSPFDRPSQVFRGIRFVLLGFDPINKAQDDPLCVAARRDGKILVSGLWADHSFDV

GVPVDPTSVMYRPVRDLNGIPGGKSLVICLTGYQREDREDIMTMVDLMGAQFSKPLIANK

VTHLICYKFEGEKYLLAKKMKRIKLINHRWLEDCGYELEMEAEAKDSEDEAEGVVTGLTE

VKKESPYHSMMLKQDASTSLQNTSASKGFENANDNVSVTMKTTSDQFLNPNKNVVGLTCE

PSNLFKGTTSSSAFKKSPSNESRNVMSSIYSRKVPMGTTPPTTDTSDMNSAKRLEKINLS

DAFNMSSPFVEKEEQNESSFGKRKMEISSGSSKLQKTSHNEDTLNKESLHMESVFQEKEK

NPSTEMSNHVPLGTGIPYVSEKKVLSSKGKSVTCDEISTPPVQDKGFDEELKEASVASMF

GSRDIDMLIQSEFEIPEAQKPEHVMQSLEKSSPSAATCDIEKSSTPNLEINEQSAGSGSK

SVKRKSIAKKFSAPKQNLGKKKTVDQKGSIYLQNIEPQNDRVDVQGVGDDKNQSNEKVED

ASEIEKEKMDDETESPEDKEEHGPHTADVVMEENEHGDDVNQSNEKVEVASEAGNSVSVN

VDKSKALREKKLPSTKKSKKKPAVSVKDASDKKEAEIREEGTPTPLRASKRSKRNVNDLQ

TAEEGVCKNDDNSTEHLYSPSEKEKEDKVQGETENVKNNKATKSKTKKDIGLSNSVKQGT

ETKTVNKKGRPSSRSCDFEAQKENIQIPVENKNKQKQALKSEKITPKSTKVNSEEPKWFI

LSGHKLQRREFQQMIRPLKGKICRVSHQWSYQATHFIVPDPIRRTEKFFAAAASGSWILK

TDYLSASNEAGRFLTEEPYEWHKNGLSEDGQINLEAPRKWRLLKEKTGHGAFYGMRIVIY

GECIAPTLDTLKRAVKAGDGTIVATSPPYTRFLDTGIDYAIVSPGMPHVDIWVQEFIKHE

IPCVSADYLVEYVCKPGYPLESHVQYDTNVWAERSFNNLKNRCLTIEEEEEESMGPTTPE

SSDVACEVCGSRDRGEEMLMCGNESGSIGCGVGMHIDCCDPPFEDVPEEDWFCPNCSNPK

ITVTNNSKKSTSKRKAK

>Soleracea_BCP1

MMYGSSIFFGLRLFLAGFTPQQYLLDKSRIISEGGVVFDEYSPDCTHVVVQNIALDDPIC

VTARRDGKTVVTNVWLNDSLEYGELLGIGNILYKPPKALTGIPGANELVICLTGYQGLPR

EDLKMMVSVMGAQISKPLVVKRVTHLICYKFEGEKYDLAKKMNLTKIVNHQWVVDCLKAW

KIIPEEPYSTTSGYECDLMEAEAKDSEDETEDITGRNNSGITNLQNEMGEAHIYSGIGRK

LPVSSAQQGFSNSILIKDGKSLQSDLLSESTSVNKLGKQYVQDHRAQSHMDAKSDNVDGQ

NAALLPSNQIGKGHPSTSGNTKNSGDFEAEKLNSSEYSKKTSKETSVPLHVEEDAEDKIG

ARKTPSSSRKAPRSRASLQTAGKIAKSDERPEEKITEMTTEGVPTFNLETDEATGFFGVS

SSKRQADPKAGKCSPLKSKNSPRKSCLSLKSEGKLSNLAERLQKPLAENVIADLCTSNTG

SDQGKAASEIGTSSAVAGLFDEKGPGGLLPQKRTADGSNASNTTPKSQKLTTSASKRVGA

SEPRTAVNKSVERSGALNSGRRIEDEFATSSAGRSSPNVSSAKSNAKNGGPSRSLAFEML

QVSEPMNTDVPTTPKTNSISTSLGVGNKIGQPQTLQKVIDASPGTNDLEAVKSGSPELKS

ADQTCKSVSNPPKKKMVARKTLGSRPKVSKRSADAQRSSAKKHPVLDDVSIHSAGQEKKE

PTAPSPAADKALVPPTVEEPIGLGSSKPQQKSDTEINDYDALLDDETEGPPEFNVDNEFN

VAEIVGTKEDLVSQKKEMAVKDRKKSKLAAKEVVKDDADEAEAGKTSDDVNEQVVGAAAK

KRRSTLLLNKSKRPVEVEKENESVFIDRAEVGRENELIRNDQFANKGVTGKSALKNKLGS

LLSNKSEGPVEVENENEPILHRDGHSDCKNFPSFNMTRDNVEVENRSDCGVSNDQGTNKV

AKRPGKLGGKKRRGSLQLKKSVEKENELVLDGGQHNGKLSSASDILDISTEVDKENEPVL

ANDQNTSKGVKRAGKLAGKKRRGGTLVLNKSEVQVHEGDANKPDLDDGKLTSTKSNLIIN

ESDAVSKGGKHAGKIAGKKRRGTLVLNKSNEVQENDECTRSNPVFDNDEDVTKDGTSKSD

LTANESDAAISSSLNIPELEKVKKQQTAYFILCGHRLQRKEFQQLIKRLKGKLCRDSHQW

SYQASHFVVPEPIRRTEKFFAAAASGSWILKTDYLTASSQAGKFLPEEPFEWHKNGLSED

GAISFEAPRKWRLLREKTGHGAFYGMRIVIYGECIAPPLETLKRVIKAGDGTILATSPPY

TRILESGVDFAIVSPGMPRVDIWVQEFLRHQIPCVLADYLVEYVCKPGYPLDRHVQYNTH

EWAAKSFSKLSSLSEEVVGVADAAATATATATPPDDHDQVSDDDDDLACQICGLTDRGEV

MLVCGNESGTTGCGVGSHIDCCDPPLKDIPEEDWFCPKCYESLE

>Rcommunis_BCP1

METASPSKTFLGVRFVLFGFDPINLRQVRAKLIDGGGVDAGQYNENCTHVIVDKIVYNDP

ICIAARNDRKTLVTGLWVDHSYDIGLVVDATSIMYRPLRDLNGIPGAKSLIMCLTGYQRQ

DRDDIMTMVSLMGAQFSKPLVANKVTHLICYKFEGEKYELANKLKKIKLVNHRWLEDCLR

DWELLPEDNYSKSGYELEMMEAEAKDSEEDTGETIDKQPSRKMENKSPHLKIGTPKPCQL

SRSTVEVPNMSHNLNEPEGFPSVANMKGILITPSGKNRDNHASGFDSNCIPAVPACQDAR

TSIGTSVGLPNPQERTPNPRNGDNDLETVSRSAERPYLGTKFSGMGYTRNTSQKSPSSVF

SGESSYNRGSSKMQLGESINISTSRVEYANDVLKSSRSEDLQKGSELFYNEASSSKKQKM

NVSCSNLGYQNVNNEAKLNMERSPSVSGKTQGLEPVSLVDGTNSLTAPRNHDCFRDDIVS

TDAAKKLHVDVSTAKLSKLEKEPIPQGQLFSENATPGPGQYKIVNERTPQTSFKGLMKSS

SASKSKGGDFEVEKSEYVVAEAGRPCHQQQDKQDPSPSNGKSEMEKSRTISSMEELQEGT

GNLISKPGRKKTIAKRTLGSGPKSKNTSNRKGFIALNKAAAQNDPAVDLSGERANYEKSS

NANELQRSTETVNVTGVKEAETVVSAKPNESETNYMDEETEALEDKDGHEDVLDDEKAGM

VDLPHQADNMMEIEQEGAQRIINNVAGVMDDCSREETDTTHLQQKETCQESGVKGKVSKG

RKQASGKTKKETVPLVSKKAESESAMKKEKGKRCSAGHSKSRLVSGKHSESSMEVEKENN

PITDEDQNISEAKVHDGKGAKLDKVSMKIKQKSRTSNSNYTSAEILKHMKTEPVWFILSG

HRLQRKEFQQVIRRLKGKFCRDSHQWSYQATHFIAPDPIRRTEKLFAAAASGRWILKTDY

LTACSQAGRFLEEDPYEWHKNGLSEDGAINLEAPRKWRLLREKTGHGAFYGMRIIIYGEC

IAPPLLRGVWASKKLEVESDSLIVVQLVTGNGTSCNENRAYAAILAKFL

>Platifolius_BCP1

MAPLGGGGGDDERLFSGVRFVLVGFDPVSESQYRSEIVRRGGADAGRYDAGCTHVVVCGR

VYDDPVCVAARKDGKKLVTEQWVDDSLDLGELSDADRVMYRPVRDLNGIPGSESLHICLT

GYQRNGREDIMKMVSLMGAKFSKPLIANQVTHLICYKFEGEKYELAKKVNINLVNHRWLE

DCLKAWQILPIDNYTQSGWELEIMEVQAKDSEDEAEDARRKPFDNIYNVRSTPLLKDEME

NYVNPDVHEPVQGPIIPSGNREVLAGRYLNTPDQIKKACKTGKSTDDIRSIVQGTPCTSG

MAISADPDVHAPSRTPNVTGDNREAAAGRHLKAPRQTRKDEAKDVGRTLDTRASAPGTAN

TSGMPVSADHDVCPSTGTPTIEMDNLENVAGKCLDNPDQMNVSRALLTTPSRVNWSAEVA

QSYNMNSKVDQMDNGATRDLNAAAIHLYVEGKLIPCEANLKSGGHATIAQKRKSLLSSVH

PKLPNGNLCSEETGTLNSSSMHRESVASEPVITSSLGRYNIESIKVDSDVEDGANVSLIG

KQQPGSCKSKLLSSSRTSLKHVRSVEGVTLPKHSANDKDIGLPEKANMHKTMTEKRCSGS

PSTTVEDEKGISALSLKSKSKNREDAQLTSGDAVNDFGSLLTHSTPKALMKDKTPDISLC

SLKNSELVSSSRNSDTEMTDALDVNRNEAVSSKSELKVASHENSETDHEKLPGTASMVRH

DTRPSKKSTASRARNAGTKRRRNAGTEMVDGSVVCKSGVTASKSQPDEMFSHGDVVPGPE

ELFSNPSTSECKTSSPKKGPTSRARDTVSKRLCNADIKTSDAPVASNLKFSKAIPQENIE

TDPRKQLNGANADGYQRTSPRKVSNTRVRNAVGKRPRDSDIKVTHAPIVDETKTMASKST

SNEVVSPEHVEASSVNDCETCSIKRVPTTGVRNPVAKRKTEVRQKFASEPGKIGSFVVSV

AKAVSSKITEECSRNVDQVTVDQVVQKAKEHGVKEVTGSFCNDTVMVDKSEGLQNSKLRN

NKRNRTTDLMDSQDRSLEQEKENRPERGNLSSKSKCGKGSMASKCGTEAIQNDKDTKGNG

CETSMILEPACFILSGHRRQRKDYRSILRRLKGRVCRDSHHWSYQATHFIAPDLLRRTEK

FFAAAAAGRWILKSDYLSACNEAGKFLEEEPFEWFGAGLNDSDTISLEAPRKWRLLRQQT

GHGAFYGMQIIVYGQCISPSLDTLKRAVRSGDGTILATSPPYTRFLNSGVDFAVVSASMP

SADAWVQEFGRYNIPCVSADYLVEYVCKPGYPLNKHVLFKTHDLAVKSLEKLLKNQQEVL

TNATEPVEECSFDISCSVCGCKDREDVMLICGEEDSTSGCGVGMHIDCCDPPIEAVPDGE

WLCPKCDVRKVPKKTIKGAKSRASKRR

>Mguttatus_BCP1

MLESKQNSEYDDPSKTFHGVRFILLGFDSDKEDEIRAKLLEGGGVDAVNYGPGCNHVIVD

KLVYGDPVCVAARRDGKRLVTSLWVDHSSDVGMPVDHISVMYRPLKDLNGIPGAKSLVVC

LTGYQRQDRDDIMTMVALMGANFSKPLVANKVTHLICYKFEGEKYELAKKMKRIQLVNHH

WLEDCLKAWELLPEANYLKSGYELEMEMEAEAKDSEEETEDMALAVEVGRKNILSSQNTR

VETKSFHQSPVMQEISRNSLYSSASKSLANVGETSKKNQSTPVKETDFQKASFSRETCEQ

NLNMASARSPAKSPAKLSSEVPSNMHHSKVILEKVDNALLSASESSKKSPNADVSKLSSK

SYVRSTPSKPTLSLRSERIESNLKGSPSSNVNKLGVSGCFDVPLKIHQDVTGFDGVKTPL

KGTLSNLDEGQSFTLSYKRKMTVPRGSSKSLNHDPKPSTEGESVTKATTEQTKVPTHGSL

VDGSRVLASDITPINLTRSFRKEASVSHKEALNISQAAEDRQEECDDQSLQRSPEGLENR

TLFNVDMNDLSSIRTNNCTSGAEMHQNDGQTVEPSSPKTNSVGRKRKMLAKKTLGSRPSS

GKASATKLKGSINLQKAVLPNESIIHSNGAVETETTDNTTVPEKVVVVLPTNGEDGPADD

EGKNEFELDGNKEKSIDFEAPLSREGIVKHGDDESIQTKNKSGGSKKKSTKAEKAVCTEK

NDLTESKSKSTGNAREKKISRCEKLPVVKSKENQKKDPSEKPVCSEKIELTESKSKSTGD

AGEKKIAKGKKRSLVKSKEHHTEDPAEKAVCSEQIELAESKSKSTGDAGEKKITKGKKRP

LVKSKEHHTEDPAEKAISSEKIELTESKSTGDAGEKRITEGKKRSLVKSKANCKKDHVEE

VANDGSNSKHDEKKTVSGNEEETAVLTGRTKGRPSKKLKSSAAMEKENIPSSIKQQSMTK

EDPKSLKRPLKNVTKAVKASPVKAIQKPKLRTEPARFILTGHKLQRKEFQQVIKRLKGRI

CRDSHHWSYQATHSIVPDPIRRTEKFFAAAASGSWILKTDYLTACNEAGKFLPEEPYEWY

EEGLTGDGAINLEAPRKWRLLKERTGHGAFYGMKIVIYGECIAPPLDTLKRVVKAGDGTI

LATSPPYTRFLQSRIDFAIVSPGMPRVDMWVQEFLRHEVPCIAADYLVEYVCKPDYPLAK

HVHYNTEAWAEKSLKNLMSRMEEVVIEEDVSTSEGYSGDDVACQVCGSRDRGEEMLICGS

EHGSVGCGVGVHLNCLDPPLVDVPKEDWFCRSCSKKIKKSKTIGSSSSKRTSKSKK

>Mesculenta_BCP1

MLETTSPSTTFLAVRFLLFGFDPIKEREVRAKLLSGGGVDAGQYSQNCTHVIVDKILFDD

PLCVAARNDGKTLVTGLWVDHSYDIGMAVDATSIMYRPLRDLNGIEGAERLIVCLTGYQR

QDRDDIMTMVGLMGAQFSKPLVANKVTHLICYKFEGEKYELAKKLKKIKLVNHRWLEDCL

RDWELLPEDNYSKSGYELEAMEAEAKDSEEEAEGPTVKQSSHEMANRSPHLRMGTPKSCQ

MPTLTGELPKMACNLSEPEGMPSVGNGKDMLGTPSRNNRSASGFISNFVSEASPCPVSDA

SNDAPSVGLHNPQEKTPNSTKGGSDLETISGSAERPYSDAKFSARSYTRKNPRTSPISTF

SGKLGNTRGSPKVQLGESIDKSSAKFESAKDLTGSGHVEVLPRTSELFHEEASSSKKQKM

DVSCFNPKSHDMGHEPPRSVTGSPSVSCNQGLEQPHLVDGLSKINNQHPDVTGSPLVSLD

AAAQKSHSDVSIGKASKFKTKQLMQDLLPLEDAASEDEQKKDADENMPQTSLKALKKSSL

ASKPEVGDFGVKKSEDLVADAEVPCHQQQDRQVPSPFNRNLEGEKSQTIANLEGLEEGNG

NLMTKRVRTKMIAKKTLGSRPKLKSTANLKGSIYLNKVAAQSDPAVGLPREIAGHENFSS

FNELEISPATVDAIAVKEVETKIDPKSGDNTENATTVMDDETEPPEDKDTHEGVHDEEKD

GVVDLSSKADDNTKVKPDVSQHSTNNTAADMDDGAKEDKIAVQSQQKDKTTCKANGMKGK

VRQGKKQPSGKSKTKTVLTLSGHAKSKQASDGEKTCNGEDSVEKVMGEEKGKPCSAGQTK

SRTISKRKSENSMEVDKENKQIVDGDQNISQFKGHVRKTALKSDVSMKANQKSRKRDPKC

VLVREVSKQLETEPIWFILSGHKLQRKEFQQVIRRLKGKVCRDSHQWSYQATHFIAPDPI

RRTEKFFAAAASGRWILKTDYLAACSQAGRFLGEEPYEWHKHGLSEDGAINLEAPRKWRL

LREKTGHGAFYGMRVIIYGDCIAPSLDTLKRVVKAGDGTILATSPPYTRFLSSGVDYAIV

SPGMPRVDLWVQEFMRHEIPCIVADYLVEYVCKPGYSLERHVLYNTQTWAERSFANLLSK

AEDIVEGVTPSDDCSNDDTTCKVCGSHDRGEVMLICGDESGSVGCGIGMHIDCCDPPLEN

IPEEDWFCPNCSNSNSSPKRRKGA

>Slycopersicum_BCP1

MLYEDSSKIFVGVRFVLIGFDPHRKEQIQSKLVEGGGVDAGKYGPDCTHLIVDSIIYDDP

ICVSARRVGKIVVTSLWVAHSFDLGMPVDHHSVMYRPPRNLNGIPGAKSLIVCLTGYQRQ

DRDDIMTMVGLMGANFSKPLVANKVTHLICYKFEGEKYELAKKMKTIKLVNHQWLEDCLK

SWEILSEAEYDKSGYELEMMEAEAKDSEDENEGIAANRSAETIAIMGPENPKSPNQFFVK

QEASTNISGLNTSRGSSGLGNRKELSLSASKQSKSDQVPAFEESPNRHDEMLNSNFYRTK

EEPPSSMQQNGSDLVSVSNSARKSPHSCLSREFPRKFSSPMTSEHMKSYSGSPAATEAGI

GFDCFNLSSQKGLEKSELGVNTPKNLSFSGKGQSSKLPEKRNMGISDSSFNSPRAGNNFE

SIPDGYLTANRSEELINSSKLHSLSHKGSHLLSRNPAPHLDKGVALETMQHYDSTISNPA

TLSTGQGHDEDAPQSSTYMIVGSKETSHAAVDVSGPLDERHDVPSPVNERRDTLKSNVLA

EFNKAGDDSVSGSKTLKKRSLSKKTLGSRQSLGKGDGRNQKGSLLINKTVLTNDSAASSS

GGREETEHQNILSSTKGEVLPADNANSSKEIERNDFLNSEKESAKTNESLNDDTEAPEDQ

EDEELNVLREKSTGTEAQHSGLQSAEKKINANKVVNKNEREDIAKSLSTVSNEFETQKTS

SGKVTKLSKSTSVGKASEEKVPEGPKCLKKKSKKTNLATKRAAVSVEGALSTESNEFETQ

KTSSGKETKLSKSTSVGKASEENVSEGPKCMKKNTKKTNLATKRAAASVEGAERKKCRTD

RNKVEVKKGKGSPSETGKASVISTENQLKSSMDVEKENMPVNGLQNAIHNDHEADRSSPY

DTKSRSLKVTEVQSEPRWFIVSGHRLQRKEFQQVIKRLKGKVCRDSHQWSYQATHFIVPA

PVRRTEKFFAAASSGRWILNTDYLTASVEAGKLLDEEPYEWHKKGFTEDLAINLEAPRKW

RLLRERTGHGALYGMKIIIYGDCIVPPLDTLKRAVKAGDGIILATSPPYSRFLESSVDFA

IVSEVMPRCDKWVQEFLRHEIPCVLPDYLVTYVCKPGYTLSNYVQYNTHSWAERSLKKHV

NRLEEIVEEMEPSDDSSNDIACQVCGSPDRGEVMLICGDESGSLGCGIGMHVDCCDPPLE

CIPEEDWFCPECSKNMSNTSTKRKSKKGTSTSKKKKK

>Csativus_BCP1

MEIDYSRQPFLGVHFVLFGFNIVDEKQVRSKLIDGGGVDVGQYGPSCSHVIVDKNKIVYD

DPVCVAARNDGKLLVTGLWVDHRYDSGLLADATSVLYRPLRELNGIPGAKSLVMCLTGYQ

RQDRDDVMTMVGLIGAQFSKPLVANKVTHLICYKFEGDKYELAKRLRTIKLVNHRWLEDS

LREWMLLPESNYNISGYDMEMLEAEAKDSEEESNSGITKHFAMRNTKSPDKMKFGLHSTS

VISNTVPASKTLDERTSFSDTKSMLTVPTTNTEFIPSGKFDKYDEVRGPICQEVDVFSTP

WDSVPFNMHTTTSESEKQKVKNEAVTSPSNAARSPQLCATSYSRRTPLKSPLPLFSGERL

ERADASCKIATGEIKDAIGVDVSLEKMEQVTYATFSGHEQNSSKGTDLFGTGDSNAGLPL

KKNSDVSYDVPRSHSMSENTKSCTLNNPSTDEKVLGLEMSRVSLNHDDSDKRRAKTLQHS

RGSTDTSSPIKKPLICDLPFGNSVRSPTEDVAGGSLKTPRTPFQISGKDLSPDKPNKPIH

DCEISGDLVGKTKETDRQQNGVLAAPESDSGTKVTKTKSASPSSLNSSVLQNNNLQSKPQ

RIKMFAKKSLGSRPKLGSGSHRGSILSSKTTSLNDSVSLSCGNGEKLFSSSPQDVSIGVK

KVVKTADKGDFSHKYEVMDEDDKTSDPENKEDFEHRMMDTENFKEVPQISDGEKVAKEIA

SGVKCNSSASVLNDTIPSGTLKEVIERKAPLSIGNVQLDELRLEDEKSKLNVGDRGPTEE

KMLINSSKAKSKQGKVCKAPAREKNGKTGKKPQLVAAGLNTEVHTIHDYISEKVNVPCEA

MDEDDKTFDVENKEADFEQQMMDMENFNGVPLMIDDDKLEKEIASGVKCNNSSRVLDDTI

PSGTLEEVIEPKAPVSIGNVQLDELSLEDEQSKLNVGDRSPTEEKMLKNSKEKSKQGKVC

KAPSRKKNVKTGKKPQLVAAGLNTEVHTIPDHKSEKENVPCDVGDKNSHIVKHFDKITVK

SNTKQRKVTKKSSEISANSSMEIEEVLSEVKPEPMCFILSGHRLERKEFQKVIKHLRGRV

CRDSHQWSYQATHFIAPDPVRRTEKFFSAAASGRWILKSDYLTDSSQAGKLLDVEPYEWY

KKGLTEDGAINLEAPRKWRLLREKTGHGAFYGMRIIIYGECIAPPLDTLKRAVKAGDGTI

LATSPPYTKFLKSGVDFAVVGPGMPRADTWVQEFLNNEIPCVAADYLVEYVCKPGYPLDK

HVLYNTHAWAERSFSNLQSKAEEVAEDLSSQDDCSDNDIACQECGSRDRGEVMLICGNED

GSSGCGIGMHTDCCNPPLLDIPEGDWFCSDCINSRNSNSSNKRKKGVSVKRK

>Csinensis_BCP1

MEIDCQSKPFIGVRFVLFGFDPINERQVRSKLIDGGGVDVGLYTQSCTHVIVDKIVFDDS

VCVAARTDGKTLVTSLWVDHSLDIGMPVDAASIMYRPLKDLNGIHGANSLVMCLTGYQRQ

DREDIMTLVRLMGSQFSKPLVANKVTHLICYKFEGEKYELAKKIKSIKLVNHRWLEDCLR

VWELLPEVNYDKSGYELETMEAEAKDSEEEIEVASLKQFGGRDVNKSPHNLNVGIINAHE

SPKSTLEGQGLLVGSTIPEASSGIDNATDMLSTPSRVSRSHQISGFDNVNITEVNGCHYT

GASRDNSHEKTPNSAKVKSDLVPTSKNAEISYHSGSKFCSLNYSRKTPRKSILTMSSGKV

DNDVLNIISSKVENAETRTVTACGEIPKRGGELCHEEESIVLPQKRMSNSTGAGSKSQKM

SHNAAECNPRSPINYKTPVSETKSSACHSFETGNHLSPGSNGHYTIETATPSTAPNKKPL

TPDLPSLKTVTSEALHSEGADRNALETCRGSKESTVASKTDNEDFGMGSVHLDGEAEDAQ

NLHQDLEGSSAKNRFLVMDKSPRPVNIDSPQVGKDKLIAKPIRKKMVAKKAFGSGHTTNR

KGSIYSNKISSLSSPAVCLSREVERANQEKFSSTSELETDPPNLSDEATKEMETTLVAKC

GDNSGDGIKTMDDETEAPDEKYEIEFEKMLNNEKSEGVQSIHKEDTMMKRVPGVGHEAHD

SMVYGRDGITGKEVMNAEVGMTVSGKRFDLDESTSKMDGMKSKRKIRPSDKANGNVSADK

ETVESWDDEAEAADEKVGNVFEEALNDEKPEGVELIHKEDAELKRIPGAGHEAHDSVVCR

HDEITGKEGINAQVRTTLSGEMFDLDESNSKRDGTKCKINRAKKRPSGKASLKTLSADKG

TVESKKDVVGEENLNGEKNEECREKEKNVLLPRSKTRVITASASKVGSSDEVEKENRPVA

DEGQTSSPGVGKSILKSMKVSMKNKIGGNVNSNSIPLDKSLNKLDKSLNKLKDEPIWFIL

SGHRLQRKEFQVVIRHLKGRLCRDSHQWSYQATHFIAPEIRRTEKFFAAAASGRWILKTD

YLSACSQAGKFLLEEPYEWHKNGLSEDGAINLEAPRKWRLLRERTGHGAFHGMRIIVYGD

CIAPPLDTLKRVVKAGDGNILATSPPYTRFLNSGVDFAVVSPGMPRVDLWVQEFLKHEIP

CVVADYLVEFVCKPGYSLERHVQYNTHAWAEKSLSNLLSKAEEIVMDLTPPNDYDTDKIT

CQACGCSDRGEVMLICGDESGSVGCGVGMHIDCCDPPLESVPEEDWFCPTCTRSRINPSK

RTSS

>Mdomestica_BCP1

MTEGSSPAKTFVGVRFLLLGFDPXDXHQVRSKLVDCGGEDXAHYSPNCTHVIVDKIVYDD

PVCVAARNDAKTLVTALWVHHSFDVGLPIDPTSPFAQIIYRPLRDLNGIPDAKRLIVCLT

GYQRQDRDDIMTMVGLMGAQFSKPLVANKVTHLVCYKFEGEKYELAKKIPKMKLVNHRWL

EDCLRDWQLLPEXNYNISGYELEMMEAEARDSEDEAENTFGKQSGVRSMYKSPHNIKAGS

PATSRLPKSEGEVRKVPLIFDNAIGNLSIPRNENKLDQTSSFSNSYVSKGLGCQDARELR

DSSGCDLNDQHHRTPDPKVRDDFISNCGSAGRASHSAGKLSYSRQTLXMPTVPTYTGDKS

SGCSVSSKVPICKSSATLKDQANDKFDPNCVEVPLKGIHLQNGEESSGILPRKRVMDLSY

ASSKSQKMSPGAKSGSMHSPSPSNKSPKGKPTSLIDGSCRTTSHYIVTNDDHSPDKTSNL

NAAGNSMAGVSPAKLSNLIQKPLACDLPFSATVISNMGKDENANMKSPLTTIRRLRKPSL

SNKPGIVDCAEEKSTAAVSKAVELQNQPQDVEGSSASNKKSVNNSSNDPADLNMLKEGNN

DLVTKHLEQKVKSKKTLGSRPRLVSANQKGSVHLYKDASLNDTAVLYNAGDSQKSPDPTK

LDVLCPDVNVEAPNELEGKDVDMSADVDEKNIESMDDETEAPEEESEHKLENVVHEAKDI

VVQATSKCDKKSEQEQHLQDHSDACTPRDAMASLGIEGNEREKSVSDNISLLVEPAADGD

AVKGKKNQGKKHALGKTKLKTVHPVADVMKPKKVVSEENTRNDXTRVTEKKKEKRLAGLV

GKAKCRSAPQNKLENSAKMKENKPIVGGDQTVSKAKQQAEKSIAKSGXTPLKIIQTSAER

SDNPSIPEGKAXSKVKIEPVRFILSGHRFQRKEFQKVIKRLKGRCCRDSHHWSYQATHFI

SPDRVGRTEKFFAAAASGRWILKSDYLEASDKEGRFLEEEPYEWYKNGLSEDGTINLEAP

RKWRLLRKRTGHGAFHGMRVIIYGECIAPPLDTLKRVVKAGDGTILATSPPYTRFLKSGV

DYAIVSPGMPRADMWVQELLNHEIPCVVADYLVEYVCKPGYPLDRHVLYNTNAWAEKSFE

RVQSRAEEVVEEAFTGEDGEDGDDASCGGGSDIPCVVCGSGERGEVMLICGNESGSVGCG

IGTHIECCNPPLESVPEDDWFCPDCSWSKNSTKSSKKRKKGRSSK

>Tcacao_BCP1

MLESDTPSKTFLGVRFCLFGFDPVNEHKVRVKLINGGGVGVGQYNQNCTHVIVDKIVYDD

PVCVAARNDGKIVVTGLWVDHSFDIGMPVDATSIMYKPLQDFNGIPGAKSLIICLTGYQR

QDRDDIMTMVSLMGAQFSKPLVANKVTHLICYKFEGEKYELAKKIKKIKLINHRWLEDCLREWKLLSEANYSKSGFDLEMIEAEAKDSEDEAEETVSKQSGQKSLNRSPNNLKAGMLSSNELPNSAVEVPTLAMPRHSPNTKEILLTPGKSHQGTNFNNINVPELRAFKDADVLGDASFIKLAEPHNRSPNSTKVDNSLTSTSKSPSLSDEKFTAISYTRKTPRKSPAKSTLPNLSGEILGNSGDFPQGIKFKDASDNSSSKMQQPKERISSFFVESPLKRDLCHGEDSAGILPQKRASELSTSSSKSQKMSHNAKAGIKGSAVGIEQLEPTSLVVDQLHIKDCSVEGTGYLNIVPNSCASNATAKSLTNDLSSFITVTAEDRQINTDEKSPKMSFRGYRESTLAGKHDMQNENADEKSPQMSFQGLRESISASGPNIGDSGLGRCVQVVREPGEPLNKKQDVKIPSLDDRKLEMENSHSPATLDLLEGGSDKLVTKPLNKKMLAKKTLGSRPKLSNISNRKGSIYSSKIASENDSTICLSGANEKAIHNSASELEASPLTINMEAAKDVVKKVVADAAGSKAQFVNDETEAPDEEDENDFEKTHEKEKSELVESACKADTIIEVEHVRQDSKVALHESLTTLENGTNGTDPKRAVGSKNSELGESTLKCDGLKRKASKRKKQLSGKAKMKTVPSESKNDLIGEDTSVGKNVEEKDDEKENFLPHPVGKINSSPVDPKEIAGKSVVEPNKKAVKTNDKFRKVNSNTQTVQKVFNRFETEPAWFILSGHRLQRKEFQQVIRRLKGKFCRDSHQWSYQATHFIAPDIRRTEKLFAAAASGRWILRTDYLSACNQAGKFLPEEPYEWHKNGLSEDGAINLAAPRKWRHLRERTGHGAFYGMRIIVYGECIAPPLDTLKRVVKAGDGTILATSPPYTRFLKSGVDFAVVSPGMPRVDLWVQEFLKHETPCVVADYLVEYVCKPGYSLERHVLFNTQEWAEKSLTNLTSRAEEIVEDLTTPQPPDDCGSNDVTCQVCGSPERGEVMLICGDESGSVGCGVGIHIDCCDPPLEDVPDDDWFCPKCSRISRNRATPPRKRKKGTSQSKGK

>Mtruncatula_BCP1

MLETSHSSRVFRGVHFALFGFDHLTENKIRFKLVNGGGVDAGKNTGNCTHVIVDKIAYDD

PVCVAAREDGKTLVTALWVEHSADIGMPVDATSVMYRPLKKLDGIPGAKDLVVCLTGYLR

QDRDDIMTMVGLMGAQFSKPLVANKVTHLICYKFEGEKYELAKRLATIKLVNHRWLEDCL

KDWVLLPEDKYNKSGFELEMMAEEAKDSEDEAEDSKLGQSGGRTISKSPLGSKFGTTATH

GLSKPLREEASNAIPNSTGPQVFPNANKGKDSSITTGNKNNSGQDVDLNNIGDLKVSCQV

PDVSLHSTSCQLPESYVRTIESKNADFPKAPGFQGQDQDITGNINSSGLRPNLHGDNLET

KISYTRSTSTSADGLAHSDEKLGATSYSRKNQKEFTFSRVVDQYEGREGNSLETPSTKVE

KTSEGIKSACVEGSGNETGVIQEVYRNNSLPQKRTNEAASSTKLKSRKITSNAKLSIEKT

PLTNGKSQGLKVPSVVDEPPVSDGFLSVDKDGINNLNTCLISKSAASASNSVAFDKPISG

NAESAQLDNAYQNSAQKTVQSLNKSKIGGEPDVTGFGKGHGDNEEEQLNVTTHLECSSPG

KNSKNEGFPGLDNLDLSNEESNKLIRKSPRKKSAAKRTLGSRPRKGVTAKLKSSVCLNKT

TQQDEGVSSSGRSKEIATSGAKERQASPQILDVNKLMEQKPVNGYAEGAGGRTDFLDDET

EAPDDKCESELGMAPNEELVHLSKKVDTSTEEKLEAVNHDKKCEEPLPPKKVTNETKKQN

LPSELDSTSKLKVKHQAIKRPASKTKKTTVAKRLAKSEKAVFGEKIPNETRDEAEIKILK

EMSLSVPSDISENSNAPKNKPENFIEEEKENRPNDGEHGLEERRNVRTTKSSVKPANIKS

KEMKHAPSTSEFNARVKPETTCFILSGHRLQRKEFQQVIKRLKGRVCRDSHQWSYQATHF

IAPDPLRRTEKFFAATASGRWILKTDFLSASSQAGKLLPEEPYEWHKNGLSEDGAINMEA

PRKWRLLKERTGHGAFYGMRIVVYGDCFAPPLDTLKRAVKAGDGTILATSPPYTRFLDTG

VDYAIVSPGMPRVDLWVQEFLKHEIPCVVADYLVEYVCKPGFSLERHVLYGTNALADRSF

AKLQSKAEEIIEEVIPHEECDNDDDDVACQVCGSRERGDVMLICGDESGSVGCGVGTHID

CCDPPLAAVPEEDWFCPKCSSTQTCSKKANKRKKGALSSSKAK

>Spurpurea_BCP1

MLQTDSPSKTFIGVRFVLFGFDPVNKTKLKSKLVGGGGIDAGQYSEDCTHVIVDNIVFDD

PVCAGARNDGKTVVTGLWVDHSLDIGMPADATSIMYRPLRDLNGIPGAKNLIICLTGYQR

QDRDDIMTMVGLMGAQFSKPLVANKVTHLICYKFEGEKYVLANKIKKIKLVNHRWLEESL

RNWELLPEDNYSKSGHELEMLEAAAKDSEDEVDGTSVMQPSYENANKSPQSLQVGTFKAC

EMPKTGEVQKISHNLSVPEGVSSVVNAKDILVTPGKRSRDDHASCFDNICVSEVPGHLDA

GGIKGATSNALPDTQGRTPISTRTSDHLDVERPSHSDAKYSTTSYTRNTPRISPSSIFSG

NSGNIRGSPIVLLGESVNMSSAKAEYSKDITSPSCAEIPRKGVELLYEEAPGSKKQKTDV

SCSSSKSQKMNHDAQAYVTGSPSVTYTSQGLEPTALVDGLSRINNHSPLGNNGHSVHDMI

GMNALQNPHANFSTAKSSKFRRNSFTKDHVFLENMILGTGENENTNKNTPQPSFSDLTKD

NLVRGPDSGGFATERSEQVVAEAGEPQDWQQNGGDPCTHEKGLETDKSCMHSDLNLPQDG

NDNIITNPARKKMIAKKTLGNRPKLTSNVNQKGSIYLNVTASQNDPTVGMAKGKGRVENR

SPNDATELETSSATVNVAEAQEMETESATKLGDNLGDNAVDKIGFTDDETEAPEEKDEGE

SLLNHEQTVMIVLSHKADNKIEMKLEADNYAANMRDGPAERKNAIEIQKRDRSTLKEGFV

IGKGSRGKKQSPGKTKTTAVTLVVKEAESKKVLDVEENLNGKKIEENAAEKESTEPCPAG

QAKSRTVSRKKSKNSVETEKENKPTVEGDHYASLDDEHVGETAANASKTPMKFNQKVKKS

NPGFTPGKEVTKQLKTELQWFILSGHRMQRKEYQQVIRRLKGKFCRDSHQWSYQATHFIA

PDPIRRTEKFFAAAASGRWILRSDYLAASSQAGRFLAEESYEWHKNSLSEDGTINLEAPR

KWRLLRERTGHGAFYGMHIIIYGECVTPPLDTLKRVVKAGDGTILATSPPYTRFLTSGVD

YAIVSPGISRADMWVQEFLRHKIPCIVADYLVEYVCKPGYSLDKHVLYNTNDWAEKSFSN

LLSKAEEIVEDSDSGDDIACEVCGSRDRGEVMLICSDESGSAGCGVGMHMDCCDPPLESI

PEEDWFCPKCSGSSKRTSPRKKRMKKALH

>Lusitatissimum_BCP1

MQEAITSSSSKPFVGLRFLLRGFDSISQQQVRSKLLSGGGVESQNYTHLIVDNTAYDDPE

CVSARNNGKTVVTGLWVDHSFDIGLVVDRTSIMYQPPKDINGIPGADKLVVCLTGYQRQD

REDIMTMVSLMGARFSKPLIANKVTHLICYKFEGEKYELASRIQKIKLINHRWLEDCLTNWKLLPEDDYHKSGYDLEMMETEAKDSEEDVTETSLMVSTGHIPNLSLRNMENGSPKIGNLSKPIPESSAMVAGDLYAHNANEVLRTPVNKKRGDMGCSPDKNHASDLIAAPSERTSGSGNGGGKMNSVSKPSLSYTDSLAMSHDRKTSQRSPSLFIGASKAPGCYPKVQTGEPINFSSAKVEVANQCLGSGFEDFERLVSEELSDGKKQRIDALESSSKSHQMFQDRQTWITGSPSLSYRALGLKSSSLETAPRLNNSSPDGSYAQSHSGKGILSSNGASNSHSMLALREQSLAEGVPFGENATMEKIGKQSTDMKHQASVSGLKVSSSPSKQDDEVLCLDKSEVVDLRNQQVRASPVGKIDITTVKPSVVTANQRQDNSLQTKQERKHTATKKTLGTRMKLKSTVKEKGSIYLKESTAQEDVATDLRRRNDRTDEENLLMAKELEDPPAAVVSMEGTGNKLVIDLRTDVQYDIDSMNDETEPPDDKEQQSDAVLKERMETGGLAGSRVEKEPEEVRPVGGNSNVCEVDGVMEDPQKKDNSISEGDCISEDDGDKRKKSISKRLTSIKSARTVSSGDGESNSSKAVEAEETHLHTVDQTAKNTSEPSAGSCPSRKKLSRRRSRISMEADKENTAITQVHRRGKMTEKHVESCEARMDDNQDVLETKPLSGKLEDISTNVVKEPACFIMSGHRLQRKEFQQVIRRLKGKSCRDSHQWSYQATHFIAPDPLRRTEKLLAAAASGRWILKTDYLSACSQAGKFLPEEPYEWYKNGLSEDGAINLEAPRKWRLLRERTGHGAFHGMQIVIYGECISPPLETLKRAVKAGDGTILATSPPYSRFLASGIDYAIVSPGMPHVDVWVQEFLRLEIPCIVADYLVEYVCKSGYSLEKHVLFGTQKWAEKSFSNLMRKAEEIVVEKAAASASEEGGGESSQEDVACQVCGSRDRGEVMLLCGDESGKVGCGGGRHIDCCDPPLDSIPEEDWFCPNCCDDSLNPCLKTKKRKKGTK

>Graimondii_BCP1

MLESDTPSKTFLGVRFCLYGFDPVNEHNVRVKLINGGGVGVGQYSQSCTHVIVDKIVYDD

PVCVAARNDGKTVVTGLWVDHSFDIGMPVDATSIMYRPLRDLNGIPGAKSLIICLTGYQR

QDRDDIMTMVGLMGAQFSKPLVASKVTHLICYKFEGEKYELAKKIKKIKLINHRWLEDCL

RDWELLSEANYSKSGYELEVMEAEAKDSDEEAEETTLKHSGQGSLNRSPHSLKAGMTSSG

ELLSTVEVSTSTVPRHSPNTKEVLVMPGKSHLGTSFDDVNDPELNSFQNSGLKNGSSIKL

AQPGNRGSNTTNMDSNLASTSKSPSLSNDLFTAISYSRKTPRNATPPNLSGEVSGNISGS

PQAMKIQDVSGISSSKIQQPEKRICASFVRSPRKGSDLCHGEDSAGILPQKRALELSGSS

SKSRKMSYNAKGSIKGSALDTVQLEPTSSVGDQLQINDYPVNETGYPNVLHSSCAGYVTT

KLSTDLFSSKSVTPDDRQNVRDEKSPNMSPRGYRGSTLAGKLDMQNENAYEKSPQMSFKG

LRESTSASRSNIGDYGLERLQVVGEPGELQNKQQDVQVPSLDRKLGKDNSHSPSKLDVLE

GGNDESVTNSVSNKQQDVQVPSLDGKLGKDNVDIPLNLDVVEGGNGKSVTKPISKKKLAK

KTLGSRPKLSNIANRKGSIYSSKIASDNHSTISMDGDNERAAHKGASELETCPPTINLDA

AKDVEKVTKCQNIGTSKTQFMDDETQAPDEEDDNGSKKVTGVEKSELVEVMHKADMLVET

EHVRHDPKVAVHASPVASENATNGADPERAVGSKNSEFGEPTLKSGGLRKTNKRKKQLSG

KARMKAVPSNSKNDLAGENTSVEKNADDKDNEKENFMPHPDDKPSGANASSKVEISGAAG

KGDTVGLKEIARKSVGKPNNKTLKTKEKSQKVDMQPVQKVFKRVKIEPTCFILSGYRQQR

KEFQLVIKRLKGKFCRDSHQWSYQATHFIAPDPIRRTEKFFAAAASGRWILKPEFLSACN

EAGNFLAEEPYEWHKNGLSEDGAINLEAPRKWRQLKERTGHGAFYGMRIIVYGECIAPPL

DTLKRVVKAGDGDILATCPPYTRFLKSGVDFAVVSPGMPRVDIWVQEFLKHEVPCVVADY

LVEYVCKPGYSLEKHVLYDTHEWAEKSFTNLTTRAEEIVDDLTHESPGSSESNDITCQVC

GSGDREDVMLICGNESGSVGCGIGIHIDCCDPPLDNVPEEDWFCPKCNKSSINNTSSKRR

KKGK

>Ptrichocarpa_BCP1

MLETDSHSKTFLGVRFVLFGFDPISETEVKSKLVNGGGVDAVQYSENCTHVIVDKIVYDD

PVCVGARNDGKTVVRGLWVNHSFDIGMPVDATSIMYRPLRDLNGIPRAKNITMCLTGYQR

QDRDDIMTMVGLMGAHFSKPLVANRVTHLICYKFEGEKYELANKMKKMKLVNHRWLEDCL

RNWELLPEDNYSKCGYELEMLEAEAKDSEDEAEGTSVKQPSCENVNKSQNLKAGTSKSCEMPKTGEVLKVSHNLSEPEGLSRVPGHLDACGFKGASSNDPPDPKERTPISTRTSNDLEFISRIVERPSHSDAKYNATISYTRRTPRRSPSSIYPGNSGNTRGSPKVLLGESVNKSSAKVLNPSVTNAVQGSEPTHLVDGPSRINNHSPLGNTGHSVHDMSSMNAVLNSHAKYSTAKSSKFSRNTVTEDNAFLANMVLETGENESANNKTPQPSSRDLRENNLVLRSDSGGFVVERYEQMVAEAGEPQNQQQDGGGPFSLKELEIDKSDMLSDLHVLRAGKDDFITKPVRKKMIAKKTLGSRSKLKSNESQKGSIYLNATASQNDPTVTMAEVKEREEDGNFSDATELETSLAIVNVAVTEKMETESATKLGNNIEVKIGFMDDETEAPDEKNECENFLEEEQADMIDLPHKADNKIEMKLEADNSAAYMRNGPVEGKNPVEIQKRDESILTEDFVKGKGRKQPSGKTNTKTVTSIVRKEESKKVLNMEENLNGKKIEENAAEKESTEPHRAGQGKSRIISRKKSKNSVEAEKENKPAVDGDQYASLDDKRVGETAAKSNKAPVKFNHKVSKSNLGSTTGREVTKQVKAEPLWFILSGHRLQRKEYQKVIKSLKGKLCRDSHQWSYQATHYIAPGPIRRTEKFFAAAASGRWILKTDYLTACSQAGRFLAEESYEWHKNGLSEDGTINLEAPRKWRLLKERTGHGAFYGMRIIIYGECMTPPLDTLKRVVKAGDGTILATSPPYTRFITSGVDFAIISPGITRADVWVQEFLKHKIPCIVADYLVDYVCKPGNSLERHVLYNTNDLAEKSFSNLLSKAEMIPEDLTMSKDCDGGDDIACEVCCSCDRGEDMLICGDECGSVGCGAGIHIDCCDPPLESIPEEDWFCPKCSGSRSTSPKKKRIKKALH

>Fvesca_BCP1

MDSRAPPPQTFLGVRFVLLGFDPINERQVRSKLVDHGGVDVGIYSPNCTHIIVDRITYDH

PVCVAARKEGKTVVTALWVHHSYDVAMPLDAASVIYRPLKQLNGIPGARNLLMCLTGYHG

QDRDDIMTMVALMGAQFSKPLVASKVTHLICYKFEGEKYRLAKQLPQIKIVNHRWLEDCL

RDWVLLREDNYNQSGYELEIMGAEARDSEDEAEDTSIKQSVGRSVSKSCHNIQAGSPGPF

HIPKSEGEAPMVPQNSLEKSISQNEDKFDQASRFSEVHVSKVPSPHDGCKFKDAMLVGHN

DLHHTTPDSKMKDDMASTFGSAERPAPHSDRKLSHSRQSPWKSTLPLYSGDKSSYGSRSS

KGPIRKLSVNDDFASSSFRTEENEKNGSNCAEALLKVNHVQNEAESSGILPQKRTPDHSY

ASFKIQKTSYDPESSIVRSPSSDKPAKEIPASLIDGSYKTTIDRNDENSLDKNMTRLNAA

ETLGAVVSPTKSYNLTEKSLTTDEPSSLTLISEIRQDGNVNKKSPLSFQRLRKSPISSKR

GIGDLAVEESTTIASGTVEPQNQQQDVQGLSPRNKMSVTNKSNDLANLDLHKGGTDVLIK

SDRKMMLSKKTLGSRPKLTSANQKFSVHLDEDATLNVATGDINAADQEKSPSAIKHDLLS

PDVNVKALKMAEVKDVTPSGDVSENKIDSLDDDTEAPEEEDQHELESVCEAKTTEIKAAV

VMEEKSEGVQHTSNISDPCVHGAAMASGENMLENEQETVSVLLGSTSKGVDDKGKKNNGR

KHPLGKTKLKTVPAVTDKMISDEGVSKDLSKHSDPCVHSDAMASEGNMQENEQGETVSEQ

VESTSKGDGVIGKKNSGKKGHLVKTKLKTVPAVSDKMNSEKGVSKDIINYSEPCVRGDAT

ASVENIKGNEQGESVSMLVESTSKGDGAKGKKNSGKKRPLGRTKLNTSPALTETENNEGT

GMEDEGLESKRRSAHKSKPEDSSELKENRPIACGDQNISKDNEQAGKSHVKCNFTPMEIN

QKLAKISRNSSIQEVKASDRVNTQPEWFILSGHQLERKEFQKIIKRLKGRCCRDSHNWSY

QATHFIIPEPIRRIEKFFAAAASGRWILKSDYLAASNQAGKFVAEEPYEWYESGLSEDGT

INLEAPRKWRLLKERTGHGAFYGMHIVIYGDCIAPRLDTLKRVVKAGDGTILATCPPYTR

FLNSGVDFAIISPGMPRVDMWVQEFLKHEIPCVVADYLVEYVCKPGYPLERHVLYNTHAW

AEKSFGRLQSKAEEVLVVVEEEEETPTEDSGSSDIPCVVCGSCDRGEVMLICGNESGSVG

CGIGTHIDCCSPPLEDIPEGDWFCPKCCSQSKNTSNPSKKRKKGKSK

>Ahypochondriacus_BCP1

MMFNTSIFTGLRFFLAGFTPQQYLQVKSNLTADGGVVVEEYGPDCTHVIVHNLVIDDPVC

VAAIKDGKILVTSLWADHSLEAGGPVETDNIMYRPVKELTGIPGANKLVICLTGYQGPER

DDIMIKVHLMGAQFTKPLTANKVTHLICYKFEGDKYELARKVPFTKIVNHQWLDDCLKTW

KILPEEPYSNKSGFEVEMQNEAQDSEEENGDISGRNRITTLQNGSKDSKNHSVTPSKLSI

PSTEQGLSKSSIVNGGELLKSDFLSESRSVNILDKLPEQSHNVLYQKDAKCDNADAQIKN

GCPLTTVNAVNETDFDGEKVNIFNSSVKKPPRSSLPLHVREDSKRKNGANSPSKKSSRSR

ASLHSAGKFSQSGRMDENLAEKIDDLPTFNLETDNEVVSGCAGAPSSKRLNGSLQSALRL

SNLDEKVEKSLSEKVSADVCSSTVGKDQGKAASAFGTVAGSLDDKGSSDLRSRKRTADGS

NLSHTTKNQKLTSSASKRVDAADPKTLVTLSGSQTRDESACRSADRSSPNFSSINSSAKN

IASRSPAFDLLPSSKPINTQVVTCPNSNNCASTSLNLGIKRGEPQNLYQSGTASPGTNGL

KDMKFGSRELNSGEKTNSSDSKPPRRKMVARKTLGSRSKISKRSTDAQRSSVIQNSFRDD

AATRPLDQDTRDEPTPFCAADKDLLPPTIDDPLGLGSKPHLDTKVKDRTELFDDDTESPP

ELVVCDEKFSTAEQLHSVKLKTGEASGIQVLQAEICNSDSDQKHRQGISVAETVGAHLIL

EPQKNKVVVKAKKKPMRVAKDAVKNADDIEGGKNIDRVKEQVVGAAGKKRCGTLLLNKSK

RPVECCNDHIKLGKEKEPECNDQYANKGLKDCGKLALKKRRGTLLLNKSEGVVDVENEHQ

GEGENDGKLCPGELAATSNTSLGDMEDKNKSDSTVHNDQCTSKVAKRPGIGKKRRGTLLL

KNSDSIDMEKENESVFDGGQPTRKLARPSGVLDKSTELEKENVTVGNNQGRGKDGKRAGK

LVGKKRRGTLLLNKSEAQVDEEKENDLVSEDKSKCEILAEKLSPKSNLTANMSDAAESTS

SNIHEVKEAQFILSGHRLQRKEFQQLIKRLKGKVCRDSHQWSYQATHFIVPDPVRRTEKF

FAAAASGSWILKTDYLTASSQAEKLLPEEPYEWHKNGLNEDGAISLEAPRKWRLLKQKTG

HGAFYGMRIVIYGECIAPSLDTLKRVVKAGDGIILATSPPYTRFLESEVDFAIVSPGMPR

VDIWVQEFLKHQIPCVLADYLVEYVCKPGYSLDRHVQYNTHEWAAKSFAKLLCLSEEVIH

DAATPDDQITDDLACQVCGSTDRGEVMLICGNENGSVGCGIGTHIDCCDPPLEKIPEEDW

FCPQCAESQ

>Tpratense_BCP1

MLETNHSSRIFRGVHFALFGFDPHTESQVFFLXXXXXXXXXXXNSGSCTHVIVDKIAYDNSVCIAARNDGKTLVTALWVEHSADIGLPVDANSVMYRPLKDLDGIPGAKDLIVCLTGYLRQDREDIMTMVSLMGAQFSKPLVANKVTHLICYKFEGEKYELAKKLATIKLVNHRWLEDCLKNWVLLPEDKYNKSGFELEMMAEEAKDSEDEAEDSKLGQSGGRNRSRSPLGSKFGTAVTHGLSKSLREEASNAIPDSTGPQVFPNADNGKDSSTTTGNKNNSDQDVNLHNIGNLNVSCQVPDVSWHSTLCQLPESYVKTIESKNADFPNSSGFQDLGITVNTNSSGQRPNLRDDTSETKKLSYTGSTSASADVLAHSDGKLGAPSYSRKNQKGFKFSRGLDECDGNNLETPSTKVEKTSEGIKSACVEGSGKETGVIQEDYGNNLLPQKRTNEATSTKSKSRKITANAKLSIERTQPANGKSQGLKVSSVVDEPPVSDGFLSADKDGKNNLDTCLISKSAASATNSVAFDKPVSRNAESSQCNNARQNSAKKAVQSLSKSKISGETDITGFEMGQSDNEAEKFNITTNLECPSPGKKSKDEGFAGLDNWDLINEESNKLIKKSPRKKSAAKRTLGFAGLDNWDLINEESNKLIKKSPRMKSAAKRTLGSRPRKGVTAKLKSPVSLNKTMQQDEGVSSSSRSKEIATSCTKKHQASPEILDVNKLMEQEKVCEYAEDVGGKTDFLDDETEAPDDKCEYELGVAPDEELVHLSKKLDTSAEEKLDAVDHDKKCEAMPSKKVSNKTEKQKLPSLLDSTSKLKVKHQATKRPASKTKKTTVAKKLAKSEEAAVSGEKIPNETKDEAEIKILKEVSVHSDISENSNVPRNKPDSFVEEKENRPNDREHGLVDRTSVGNQTIKSSVKPANIIKSKKMKHNSSISEFKARAKTEATCFILSGHRFQRKEYQQVIKSLKGRVCRDSHQWSYQATHFIAPDPIRRTEKFFAARKLLPEEPYEWHKNGLTEDGAINMEAPRKWRLLKERTGHGAFYGMRIVVYGDCFAPPLDTLKRAVKAGDGTILATSPPYTRFLDSGVDYALVSPGMPHVDLWVQEFLKHEIPCVVPDYLVEYVCKPGLSLERHVLYGTHAWADRSFAKLQSKAEEIIEEVIPTKDYNDDDDDDVACQVCGSQERGDVMLICGDESGSVGCGVGTHIDCCNPPLTVVPEEDWFCPKCSGTQKCSQNPSKRKKGALSSSKK

>Ppersica_BCP1

MSEGNSPSKTFLGVRFILLGFDPLHEQKVRSKLVGCGAVDVGHYSPNCSHVVVDKTVFDD

PICVAARNDGKTLVTALWVHHSFDVGVPVESTSIIYRPLKDLNGIPDAKSLIVCLTGYQR

QDRDDIMTMVGLMGAQFSKPLVANKVTHLICYKFEGEKYELAKKIPKIKLVNHHWLEDCL

RDWALLPEDDYNKSGYEMEMMEAEARDSEDEAENTIMKQSGGRNMYKSPINIKSPPATSG

MPKSEGDVPKVPMNFYNAMDHVSIPQNENKLGQASSFSNAYVSNGVSCQNACKFRDGTDG

ELNDQHHRTPDPKVRDDLTSNFGTAERPAHSARTLSYSRQTPLKSTLPLHVGDKSSNGSV

SSKVPICKSNAKFGLASYTFKADQENYKIDSSCVEVPLKRIHSQNGEESSGILPRKRTMD

LSHDSSKSQKMNHDAEAGIICSPSSSDKSPKAKPTSMVDGSYETTSHYVIRNDDHSLDKT

VNLNAVESSYAGTSPIKSSTVIWKPLACDLPFSATVTSETAEDGNGNKKTPPTTFQRSTK

SSLTSKAGIVDCVVEKPTFAVSKTVELQNQHQDVEGLSSNNKKSVTNNSNDPASLNLLKY

GNNHLDTQSVSKRTISKKILVSRPKLTSANQKGSVCLGVDASLNDTTFHLNSGDHEKSPL

DMLYPGATAEPPKEVEGKDVTMTADVAENNIQSMDDETEAPEEESEHKLENVLHEAKAIG

VQSTSKCVTTEEKSEGMQQISDHSDACVHGDAMASAENTDGNERETTVSDRISLLVESSS

EGDGVKGKKNKGKRRALGKTKFKAVPAVADVMKPNKFVCDEDTQNENIGETEKELEKIVG

KSKCRSVPKNKLENSSKMKENRPIVCGDQSVSTAEQQAGKSTVKSNITPLKINQTSVETS

PNSSIPEGKAPSKVKTEPVWFILSGDKFQRKDFRQVIRCLKGRCCRDSHHWSYQATHFIA

PGPIKRTEKFFAATASGRWILKSDYLAASNQAGRFLAEEPYEWHQNGLSEDGAINLEAPR

KWRLLRERTGHGAFHGMRIIIYGECIAPPLDTLKRVVKAGDGTILATCPPYTRFLDSGVD

FAIVSPGMPRVDMWVQEFLKHEIPCVATDYLVEYVCKPGYPLERHVLHNTHAWAENSFGR

LQRRAEEIVEDIFAPRDNSGSSDIPCVVCGSVERGEVMLICGNESGSVGCGVGTHIDCCN

PPLEVVPEGDWFCPKCSRSKNSTSSSKKRKKGKSK

>Dcarota_BCP1

MLPDLQFDHPSNFFHGVRFVLCGFDPINKTQVFDKLVQGGGVDVGQYGPRCTHVIVDGLT

YDDPVCVAAREDGKVVVNGLWVAHSFDAGVAVDPTSIMYRPPRDLKGIPGAKSLLVCLTG

YQRQDRDDIMTMVSLMGANFSKPLVANRVTHLICYKFEGDKYELAKRTNRIKLVNHLWLE

DCLKAWDLLPEADYNKSGFELEELEAEAKDSEDETEISLKKNQLLNPKQDFKMDRPNSSS

SKDLPTLNNTNDIASANRKDRNSHLDLNTPERLNKESEVLDSRGAEAFSNASCKKPVRSS

DRTAVPTTVGKNVASPNEMCGLSDASHLNIKSYDSKSPMRTTSFGYSGNVTGLGMAPMVN

AGDGADILSEHQKNETAIGCFRTPVKPVLPQREQSSTINENRTHQAQKTDHSPATRVSIH

SPEMNRSKTSEAASSIGGLNEIRRSPASIAPVSENCNTKNRKGAGLIEAFEIPVMGSEKA

KRENNPENGDVAKRQDSDASEALNTALPRTSETKKSNVSSNLRLPVDAHVKRPTKMSGRM

SWSKGVASSQKGSIYLSKSSSEVDPAISLNSAAEDDPAISLNVGERTKNNEMIEVIPSAT

NLNADLEKASHDDLQSGSKVQYNPLSMDEDTEAPEENEENELREGLFQVNNGKGKLDITR

KDDKSDYARKQKGGSHIETYGEDNDGAKEKEITRGIRKLGKSATSHNKRLREEKDETCKK

EATHGKRKLVEIKKKANPSGASEVDKGNEFVKAIDGENCRDVEPGSTENYVEDKLKETQH

NKSDCNVSGMHSCKSSGVNKGGSNQTKATCGKRKLVESSNLENTVNDDEKTVKKRPSSKT

QIKKKVPSTAKMTKSKEACSEDAEKETTEDNRTHPTGELKNTKSINKLNRSVEQEKENKP

VRTVDQNASHGKNVQKLASTKAINKIGKEETKHLPNFDRVEIQKKPNTEPVRFILSGHKL

QKKEFQQVIRQLKGKVCRDSHNWSYQATHFIVPEPIRRTEKFFAAAASGRWILKTDYLSD

SSQAGKFLAEEPYEWYKSGSSEDDAINLEAPRKWRLQREKTGHGAFYGMSIVIYGECITP

SLDTLKRVLKAGEGTILATSPPYTRFLNSGIDFAVVSPGMPRVDIWVQEFIRHEIPCISA

DYLVDFICKPGCSLDKHVLYDTYPWAEKSLERVVNQSEESAELASPPRDGQSPPPFEEVT

TPVNADVNDLPCEVCGSRDRGEDMLICGSESGTKGCGTGRHIDCCDPPLPDVPSEDWFCP

KCSKKPSKKKVRAAKRK

>Pvulgaris_BCP1

MMETSYASRVFRGVRFVLRGFNPAAESQIRLKLEDGGGIDAEQYGGSCTHVIVDKIAYDD

PLCVTVRNDRKTLVTALWVDHSADIGMPVDASSVMYRPPKDLDGIPGAKGLIMCLTGYLR

QDRDDIMTMVGLMGAHFSKPLVANKVTHLICYKFEGEKYVLAKRLGTIKLVNHRWLEDCL

KEWVLLPEDKYNKSGFELETMEEEAKDSEEEGEDSKLGKSGGRNIKQSPVNSKFDQAATH

GLLKSVIEASNNLPDSTGPRVLPNVKNGEDSLTIPGKKRKSDRDFSFQNVDDSKISYQTP

DISRLPDKYRKTTESKNADFPKALGCQGPGNMANTKSSGQQSGLHDNTMESEKLISNLTS

TSASAAGVAHSNEKLRTTSYSKKNQRDFSVPRILDGSSGREGNKCENSKVHEAIEFIKST

SVEISGRENDFTKEEEPISLLPQKRINETSFTKLKSRKVSSDAKISIQSANGKSQGLKGT

SLVDEPPKADDYCSKGKDGMNNSNTCLVSKPAGSNYNSLAFDERFSRNASPESAQCDSVY

QNSPQTAVHSLSESKTNGKPGVTSSAMRQVGGNEAGQHFSKSIDCSSMGNKNSNNVKSAG

CTNLDLSNEECNKQVRKSPRKKSVAKRISGSKPIVGATARHKRSLSLNKTILQSEGVTLS

SGSKEICDAKMHKGCPQSRDINNTMEQEAVSKNTDYASDRAEFLTDETEAPDDKCEFEFG

MALNEELVHPSEKPNRATEEKSEAICPATKCEEAKKGTNKTEIQKTSSLVVKNQAKKRAA

GKANATVSKYADDAGGRTEALDDETEAPDDKLENELGMAPEVELVHPSEKPNRATEEKSE

AICPATKCEEAKKGTNKTEIQKTSSLVVKNQAKKRAAGKANATVSKYADDAGGRTEALDD

ETEAPDDKLENELGMAPEVELVHPSEKPDTSTEEKPEAICPVPKCEEVMPPKRVTNKTEK

QKSSLVVKHQARKRPVGKAKATVSKDLSKSKVAVSREKVPNETGHEAEIETTEEMPFPAD

RRDNSALARNKSENLAEEEKENRPIDGVQNPVKGRSDDNPNYKSNVRPRKMKSTKVGLNP

SISESNTRVKTEAACFILSGHRLQRKDFQQVIKRLKGRVCRDSHQWSYQATHFIAPDPIR

RTEKFFAATASGRWILKTDYLSASSQEGKFLAEEPYEWHKSGLSEDGAINMEAPRKWRLV

KEKTGHGAFYGMRIVVYGDCIAPPLDTLKRVIKAGDGTILATCPPYTRFLGTEIDYAVVS

PGMPRVDMWVQEFLKHEIPCVVADYLVEYVCKPGFSLEKHVLYGTHAWAERAFDRHNSRA

EEIVAPEDSTDDDDDDVTCQVCGSRDRGDVMLICGDESGSVGCGEGTHIDCCDPPLTDVP

EYDWFCSKCSTTPNSSNNPTKRKKSVLS

>Carabica_BCP1

MIPSKIFAGVHFVLVGFDSISHQQVRSRMVEAGGVYVGRYSPDCTHAIVDKAVYDDPICV

AARSDGKTLVTALWVYHSFDVGMPVDPALIMYRPLRDLTGIPGAKSLVVCLTGYQGHERD

DIMVMVDLMGANFSKPLVANKVTHLICYKFEGMKYDLARQIKRIKLVNHRWLEDCLRTWQ

ILPEDDYDKSGYELDMMEAQAKDSEDEAQDMDTEQTRGKGMVSTSGLLSVSKMEAKPDQV

SREGTPVRLLEVPGSFVPGTAGKICTELNSMGRTPLPENVISDLTPASKVDEKSPSPNAS

KFTTLSYSRKTPRKAILPVESVQTESKAQISVIRDFDNKVHVSDSFSMSSCNMDVDGTTS

NDKRSPLKEILSCPDDGRSYSLSEKRKVAIFVGSSKLQRTDNNLDVSSDGVVVNRTKERP

PEPSMNELVKVSGHSPGKKSGYADTTTDLNPLKSSPAKVCSPITSEIEQVCSKIGPQISS

EKRNITCMDSNPEVKDLHSNRPENAVNDSTMVQNGLQDEAPPPETKVHEVERCNPMVGLD

VPGGEASTRSKPLKRKLLAKKTLGSRPSFGRGKALNQKGSIHIKEKGSAKNHSMSPLGQN

ETEEPGRFISTERVKVVHPTFDAEMDEDANMANVLESRNEEAYKTRFVDDETEATENVED

KELDAIIDNDKPGDIEVPNSVPTRTGEKVGVQIKQTADDIPGVEEQVVDSGDDKLMSEAE

NAAGKKNEQSESLLGDNAKGERITSGNKFPSTKTRKKNIPVENSGKGGQRKEAKDELSGK

KAKTRNAKGFEVKVDKDIIPAQVDMADNSMGMEKENTPLEIGSINVNNTSKKMVGMSTRK

SNIKPQKDDGEDSGSKSVAQIIVKTEPIWFILSGHKLQRKDFRQVIRHLKGRVCRDSHQW

SYQATHFIAPDPLRRTEKFFAAAASGRWILKTDYLSASNEAGKFLAEEPYEWHKKGLSED

GAINLEAPRNWRLLRERTGHGAFHGMRIIIYGECIAPPLDTLKRVVKAGDGTILATSPPY

TRFLQSGVDFAIVSSGMPRVDIWVQEFLRNEIPCVLADYLVDYVCKPGYSLDRHVQYNTL

AWAEKSLKNLVTRMEEVVENPTSEENDDDITCQVCGSRDRGEVMLICGDENGSSGCGIGT

HIDCCDPPLEEIPQEDWFCPNCRNKNGQNAQKNSRKPISRLKRK

>Hannuus_BCP1

MLENHQISPPQLFHGIRFLLLGFDPIKKSQVSRKLVDGGGVDAGQYGPNCTHVIVDKLVY

DDPLCVAARQDGKILVSSLWVDHSFDVGAPVDTTSVMYVPVRDLNGIPGAKSLVICLTGY

QREDREDIMTMVELMGAQVSKPLIATKVTHLICYKFEGEKYLLAKRVKRIKLINHRWLED

SLKAWEILPEADYSKSGYELEMEAEAKDSEDEQEGNNTRHNEAKISPRHSLLSKQEIPNA

VSNTSVSNMFSNAQETVSLTTKTTSDRFPNPHEIKIKPQHTQNVTSVASEPSNHYEKAVS

SSSAQNNNIFASTSANKSPQNEAKMVVSAGYSTKDPIRTPPTRTTPSAIDMKSNTSSAKR

PNTLNFSDAFNMSSSLVQKATPYDGSAFGSVDERTYTSSTKRMLDISCGSLKPQQMSHSN

VVETVFQELKKVPPVEISPARKPPSNISGRKSLRSKGENVICDVGISKTSTSEIQDTDAV

QRAQKEYKETNLYTKSDNRDVDMAENVFQSPFAANFDLQKSSTPNLDINEQSAGSNSKLV

RKKPVSKKLSAPIETSPARNSSADIPGRIPLGFKGKNVISTAEIGDLNAVQRPQKEYKES

SLSTKSSNRDVRIAESDMQSPSAVSSDLQKSGTMNSETNEQSGGSNSKPGRKKSISKKFS

APKLSKKNTVNQKGSIFSKNAELGINDENLLYQQFEKVPSVAKSGTEIEKEAISGVQMEN

MSEKTSLFMDDDTEPPDDKDEASKEKNEDVVDPCADKNNDENDVFVNKHKVPVHESDGNI

GNLASEIPPTIKGKKNTALSMKEVINKKSVEKNELTIDKNSEKQEIRKEATPNRAKRARV

DVNDLEKSMEVKSAGVCNDLEHNNDEKGDDVDANMDTKNAVNKKETTRNKRPSSKTKKVA

SKRTGTDLEKSVEVKRAGVCNDPERNNDEKGDDVDADMDIENVANKNETKRNKCSLSKTK

KDVSKRLVSDLEKAADVERVEGVRHDPAQTNNEKDDDVDIENVNKKEPMRNKRPLSKTKK

DASKRNTTEQKKEEKDNNVADMDTENSLNKKETKKNKRPLSRTKKDDANAVNKKGKPTTN

QAVETSDLNEAQKENVQIHVNDQSTGNKHVFKPAKEVTEKNTDKANSNALKNEPMWFILT

GHKLQRREFQQIIRRLKGKVCRVTHNWSYQATHFIVPDPIRRTEKFFAAAASGRWILKTD

YLSASNQAGKFLPEETYEWHKNGLSEDGQINLEAPRKWRLLKEKTGHGAFHGMKIVIYGE

CIAPSLDTLKRAVKAGDGTILATSPPYTRFLNTGVDFAVVSPGMPPVDVWVQEFLKHEIP

CVTADYLVEYVCKPGYPLERHVQYATNVWADRSYNGLKNRLEEEISEPRTPESTDVACEV

CGARERGDEMLICGDEGGSIGCGVGTHIDCCDPPLAEVPEEDWFCSKCRKKPKNSKGKKG

SKK

>Gsoja_BCP1

MMEATYPSPMFRGVRFVLRGFNPVAQNQIRFKLANGGGVDVGQYGGSCTHVIVDNVAYDD

PVCVAARNDRKTVVTALWVEHSADIGMPVDATSVMYRPRKDLDGIPGAKDLIMCLTGYLR

QDRDDIMTMVGLMGAQFSKPLVANKVTHLICYKFEGEKYELAKKLGTIKLVNHRWLEDCL

KEWVLLPEDNYNKSGFELEVMEEEAKDSEQEAEDSKLGQSGGRKRKQSPLSSKIGIAATP

GLSKSATEASNALPDSTGPQVLPNVNNGENSLTVPGNKSRPDQGSSFHNVDDSKVSYQAA

DISRHSTSCQLPNKYVKTSESKNADSPKAPGCQDLGNMGNTNSSDQQPDLNCDISESKKV

ASDLRETSASAAGVAHSNENLRTASYSRKNQKGFTLPRILDESSGREGNNCDNSKVQKAS

EGVKSTSVEVSGKGNDFVKEDEPISLLPQKRINKASFTKLKSRKKTSVPSANGKSQGLKV

TSQVDEPPEADDYFSIGKDGINNSNTCLVSKPSGSTSNSLAFDELLSRNASPESVQCDNV

CQNSSKTAVQSLSESKINGKPDITGSGMQQVGGNEAEQHTVTKNLDCSSLGNKKSCNVES

AGCTKLDLGTEESNKLVSKSPRKKSVAKRSLGSKPKVGATAKQKKSLSLTKTTLQGEGET

FSSGSKEVATGDARMHQGCPQIFDVNKTTEQETVSKNAGDRTEFLDDETEAPDDKCEYEL

GMALDEDLVHLSKKPDTAKEEKSEATYPATKCEEAMPPKNGTNKTEKQKTSSLAVKHQAR

KLPAGKVKATVSKYAEDDGDRTELVGDETEAPDGKCEPELRMALDEESHLSKKSDTATEE

KSEVICPETKCEEPMPPKKGTKKTEKQKPSSLVVKNQARKRPAGKTKAKVAKELPKSMAV

SGEKIRNETEHEPEIETMEEMPLPDDKSDQPAIQRNKSENFAEEKENRPIDGEQGRSNGS

STIKSSVRTAKIKSKKSGLNPSITESNTRVKTEAACFILSGHRLQRKEFQQVIKRLKGRV

CRDSHQWSYQATHFIAPDPIRRTEKFFAAAASGRWILKTDFLTASSQAGKLLAEEPYEWH

QNGLSEDGTINMEAPRKWRLLKERTGHGAFYGMRIVVYGDCIAPPLDTLKRVIKAGDGTI

LATSPPYTRFLSTGIDYAVVSPGMPRVDMWVQEFLKHEIPCVVADYLVEYVCKPGFSLER

HVLYGTHAWAERSFDKLKSKAEEIVEELVAPEDSGDDHDHDIICKVCGSRDRGDVMLICG

DESGSVGCGIGTHIDCCDPPLTHVPEEDWFCPKCSSNRNCSNNPSKRKKTVLSSSVYVR

>Ahypogaea_BCP1

MLGTSDQSEVFCGVHFVLFGFSPEDETEVRFKLVNGGAVDEGKYSGSCTHVIVDKIIYDD

PVCIAARNDGKTVVTKLWVDHSSDIAMPVDASSVMYRPLKDLNGIPGAQNLVVCLTGYLR

QDRDDIMTMVDLIGATFLKPLVANRVTHLVCYKFEGEKYELAKRMGTIKLVNHRWLEDCL

KVWALLPEENYNKSGYELEIMKLEAKDSDEETEDFDLGQSERNISRSPLTAKFGVEATSG

LLKSDRELSSAQTESTSLKNSPKVDNSKESSFIQGNNNGADRDISFSNVDEPKATDKFLD

VAIGATSCQIPETDVKIRESKKANSPKTGCQDVLITEKAEPHAETFNKLANDFGSPSTAS

AERVTHSEAKFSSIKYYSKNKRGFTLPRSLNDGLDNGAGSTVSPLADLKSGNKHKTPSNK

VLNGSGGIKSTSAKDSGDVTIGKLPQKRVNEAASNTLKSRKITSDAKLFIEKITSENGKS

EARKVTSVVEEPSKSEPCFSAGKDSFDKPNKNSFSKSIASISNSLSFDEPFTRDTSPKSA

RSDKAYQTESKINGKHEIAGHGMEQVAGNEVEQHKLAKNLEGSPDNKNPLNGESPNLDLG

NEESTKLIKKSPRKKSVAKRSFGSKPKVGATAKQKTLVSSNRTIQQGEAVNFSSRIKEVP

IDNGDKPQPMTDDNNKSEEQETVVKHADDANGRLETMDDETEAPDDKCELELGMKHDENK

NKADETAEKSEDMHHVTECEAAVPEKETAELTEKEKAPSVDDSTMKREVKHQVKKRPLSI

AKKVTVAKGLQKSKVALSENKISMERENVARMKSAKEVSHATDASNKSFAPRKAFGDSKE

QKENRPIDEGEQDMPEAKSVANRTNKLSSKPEKINPKQTGYNPPMSEANKRLKTEPPCFI

LTGHHFLRKDFQQLIKKLKGKVCRDSHQWSYQATHFIAPEPLRRIEKFFAAAASGRWILK

TDFLTASNQAGEFVEEEPYEWHDIGLNEDGTIDMEAPRKWRLLKERTGHGAFYGMRIIVY

GDCITPPLDTLKRVVKAGDGTILATSPPYTRFLGTGIDYAIVAPGMPRVDLWVQEFLRYE

IPCISADYLVEYVCKPSSSLEKHVLYDTHAWAERSFSKHQNRAEETTDIEESISSTPESN

DVACEVCGSRDRGDVMLICGDESGSVGCGVGTHIDCCDPPLMDVPEEDWFCSKCIVKRNC

SNSPKKRKKGGFSKSKRK

>Cpapaya_BCP1

MESGSPSKTFLGVRFVLFGFDPVNEHKVRSRLVGGGGIDVGQYSQSCSHVIVDKIAYDDP

VCVAARDDGKTLVTGLWVDHSFDIGMPVDSTSVMYRPPKDLNGILGAKGLIICLTGYQRQ

DRDDIMTMVGLMGAEFSKPLVANKVTHLICYKFEGEKYELAKKMKKIKLVNHRWLEDCLR

DWELLPESNYNKSGYELEMMDAEARDSEEEAEDAPLKQLGGRNMQKSPLNLKTEISNIVE

LPRVEAEVPGMQSNSSRTKDFSPVIAKRMITPDREDRRRQSPNINEANTFVGDDCQEISV

HGNALNVQLPDPCDITPKSTTLVNDLSSTTKSSGSPSPSNVRVTAMSYSRRSPRMPTVSI

SRQELSGNIDGSLKQNVGDSNVTDAHDPSTDRTGSIHFETPHKNGESRNEEGSTSILPQK

RVMSVLVDSSKSRKIELSNKSRVSGSPLVSCRKQGLEVGPLERNDSFKPGNGVSMDEPAI

SNSCASTSMTKSSCLDKKLTDDVSVPESVTSEILHNNINDEEILKTSIRGLAEPTFSRKL

NNDDLGIAKSVNGTGEAEILQNIQQNVELSSLSKRTSVTEKSQIALNLDALQEETDMLVN

KPLRKKMVARKTLGSRAKMKNTVSQKGSIYLSKNASEDNPSTCLGDGKMTTFQEIGNELE

ICPPAGAETTKKIETEIIAEVDAVRKNEFGDDETEAPEEDEQEMKKAHNQENPKVIELAP

KTDAKIGKNTRVHQALDNSKATMHNDAMASEKDMDGIKLKGVVSGRKFGVDEKVLKCELV

KKKSNKEKKQISNKAEAEIFPPVEKSTEPTKDAGERERQNRRDIDNKEMEKEKTVLCSTG

KTSRRKSVSKLVNSAEAEKENMPILCGGQNSCQGELVTKVVKSTKKPLTNSKKSAKVNSK

TVPGGKVLNKVYTEPTWFILSGHRLQRKEFQQVIRRLKGRLCRDSHHWSYQATHFITPDL

RRTEKLFAAAASGRWVLRSDYLTACNQAGRFLPEEPYEWHETGLSEDGAINLEAPRKWRL

LREKTGHGAFHGMNIIVYGECIAPPLDTLKRVVKAGGGTILATSPPYSRFLKSSIDFAII

SPGMPRVDLWIQEFVKHEIPCVVADYLVEYVCKPGYSLERHVLFNTHAWAEKSFSNLQHK

QEEIIRDSNLSPDSAASVGIVCQVCGCHDREEVMLICGDESGSTGCGTGAHIDCCDPPLH

EVPEGDWFCPKCNGNKGSSSPSKKRKKGRSSSKQK

>Carietinum_BCP1

MMETSNSPRVFRGVHFVLFGFNPLTESKIRFKLVNGGGIDAGKYSGNCTHVIVDNITYDD

PVCVAARNDGKKVVTALWVEHRADIGTCVNINSIMYRPLKDLDGIPGAKNLTVCLTGYLR

QDRDDIMTMVELMGAQFSKPLVATKVTHLVCYKFEGEKYELAKRLATIKLVNHHWLEDCL

KNWKILPEEKYFKSGFELEMMEEEAKDSEEEVEDSKLEQCRGRNRSKSPLGSKFGTALTH

GMSKSLREEASNAISDSTGPQVLPNTNKGKDSTTTPNKDNSDQDIDLHNIGDSKISRQVP

EASWHSTSRQPPESYVKTIGSKNADFPKTSGFQDLGLTENTNSPVQGPNLHDDTLETKKL

SCSRSTSASAERLACSDEKLGATSYSRKNLKGFNFSRVLDECKDREGNNLESSSTKAEKE

SEGIKSACVEGSGKETGVIQKGHKNNLLPQKRTKEAASTKLKSRKTAANAKLSIERTPLA

NGKSQGLKAPSVVDEPPVSDGYLSVDKDGINSSNTYLISKSAASTSNSLAFDKPVSRNTE

SAQCDNAYQNSAQRAVQSLSKTKISGEADITGFGMGKGDNEAKQLNVTTNLGCSSPGKKS

KNEGSTGLDNLDLCNEESNKLIRKSPRKKSVAKRTLGSRPRKGVTTKLKSSVCLNKTAQQ

DEGVSSSSGSQEIEISGAKKHQSSPQILDVNKLMEQEMVSECAEDAGGKTNILDDETEAP

DDKCEYKLGMAPDEELVNISKKVDTSTEENLEEIDHLKKCEEAMPPKKVTNETGKQKLRS

LLDSTSKLKVKHQAKKHPASRTKKTTVTKKLVKSEVAVSEEKIDNVTRDEAGINILKEMS

VPSDISENSNVPRNKSQNFIEEEKENRPNEREQGSVEGRSVVNRTTKSSVTLANITSKKM

KHNPSISGFNAKVKTEATCFILSGHRLQRKEFQQVIKRLKGRVCRDSHQWSYHATHFISP

DPIRRTEKFFAATASGRWILKTDFLTDSSEAGKFMPEEPYEWHKNGLSEDGAINMEAPRK

WRLVKERTGHGAFYGMRIVVYGDCFAPPLDTLKRVVKAGDGTILATSPPYTRFLDTGVDY

AIVSPGMPRVDLWVQEFLKHEIPCVVADYLVEYVCKPGFSLERHVLYGTHAWADKSFAKL

LSKAEEIIEVVIPAPAEDCNDGDDDVACRVCRSRERGDVMLICGDESGSVGCGMGTHIDC

CDPPLTAVPEEDWFCPKCINTQKCSKNPNKRKKGALSSSKTK

>Cquinoa_BCP1

MMYDSSIFFGIRFFLAGFTPQQILEYKSRILSEGGVIYDEYSPDCTHVVVHNIAFDDPIC

VTARRDKKTLVTIIWLKDSLRYGEPIGIGNIMYRPLKDLNGIPGANKLVICLTGYQGMDR

EDMKTMVQLMGAQISKPLVVSTVTHLICYKFEGEKYELAKKMNVTKVVNHRWLVDCLEAW

KILPEEPYSSRSGYELDMEAEAKDSEDEVEDISGRYNNGITIMQNEMGKAHSHFVIARNL

PDPSTQQGFSHNDGKLLQSDNSDFDNVKFDNGDEQRAAFPPSNQTGNGHPSTYGNPKNAG

DFDAENFNSLKHSRKTPQKTALPLHVEEGLGNTIGSLKAPLSSRKTPRSGASLQTAGKSG

KSGGRSKDKSTKKIIEDMPTFNLDIDKAEVASGFLGEPSSKRHVESDVSKLNSLKSRKSP

AKSSLSLQSPGKLSNQDGKLEIPLAEKVIADVCTSNIGSDQGKATSEFRALSAEKANLFD

ENGPGAFLPQKRTSDGASHSTPKSQKLTSSASKRVDASEPRTSVNSSHEKRSSGALNSGC

QIGDEFATPSAGRSSPNVSSSKLKAKTVASSRSLAFEMLQGVAGNNNGQPQTLQKVADTS

PGTNDLEAVRSGNLELNSTDQNDIPKPSRKKMVARKTLGSKSKVCRRSADVQRSSASKDS

FRDDAAIHSAGQENKEPPTASPAADKVFVPPAVEESNGVGSKPHLGTESNNFATLLDDET

EAPPELNVDDEPLAAEQSHLVKSKSGEASGMLIHQIDGQGMPVAATVGIKKNLISQKGLA

VKARKKSLLAAKDAAKDADEAEAGKVKEQVVGAAAKKRHGTLLLNKSKRPVEAGKEIEPI

SYDRVEVGREHELDCYDQYENKGGKHSGKLAVKNRRGTLLSNRSKGHDEVDNENNLVVVC

DDGQNGSKNCPADLAAPSNMTLDNVEIKKKNDTGVCNDQGTSKVSKCSGKLKMSDGPAEA

EMENEPVLDGGQYPRKKVGAPGILDSSMEVDKENEPILATNLDTSKGVKRAGKLAGKKRG

GTLVLNKSEGQVNEENKNKPDLDDEEVTNKDGIFTEKLTSKSNSTVNESDAVKGGKRAGK

LIGKKRRGTLVINKSEVQVNEGSGNKLVLDNEVDTSNGIEKLTSKPNLTVNESNAASKGG

KCAGKLPGKKRRGTLVLNKSEVQANEESRNKSILDGDEDASNGKEELTSKSNMIVNESDA

AVSNPLNIELKKVKNQAACFILCGHRLQRKEFQQLIKRLRGKVCRDSHQWSYQASHFVVP

EPIRRTEKFFAAAASGSWILKTDYLTASSQAGKFLPEEPYEWHNSALSEDGAISFKAPRK

WRLLREKTGHGAFYGMQIVIYGECIAPPLDTLKRVIKAGDGTILATSPPYTRILESGVDF

AIVSPGMPRVDIWVQEFLRHQIPCVLVDYLVEYVCKPGYSLDRHVQYNTHEWAAKSFSKL

LSLSEEVVADAATPPDDQVSDDLACQVCGSTDRGEVMLVCGDESGSTGCGIGTHIDCCDP

PLESIPEEDWFCPKCTD

**BRASSICACEAE**

>Athaliana_BCP1

MQSDSGLPPKTYSGVKFALVGFNPIHGNSLRSKLVSGGGVDVGQFTQSCTHLIVDKLLYD

DPICVAARNSGKVVVTGSWVDHSFDIGMLDNANSILYRPLRDLNGIPGSKALVVCLTGYQ

GHDREDIMRMVELMGGQFSKPLVANRVTHLICYKFEGEKYELAKRIKRIKLVNHRWLEDC

LKNWKLLPEVDYEISGYELDIMEASARDSEDEAEDASVKPANTSPLGLRVGAVPAVEISK

PGGKDFPLEEGSSLCNTSKDNWLTPKRTDRPFEAMVSTDLGVAQQHNYVSPIRVANKTPE

QGMSKMETDGSTSINRSIRRHSSLATYSRKTLQRSPETDTLGKESSGQNRSLRMDDKGLK

ASSAFNTSASKSGSSMERTSLFRDLGKIDMLHGEEFPPMMPQAKFTDGSVSRKDSLRVHH

NSEASIPPPSSLLLQELRPSSPNDNLRPVMSISDPTESEEAGHKSPTSELNTKLLSSNVV

PMVDALSTAENIISNCAWDEIPEKSLTERMTENVLLQEQRSGSPKQNLSVVPNLREAAHE

LDLSDSAARLFNSGVVPMEADIRTPENSTMKGALDEVPERSVTDPVMRRSSTSPGSGLIR

MKDKQETELTTKKTAPKKSLGTRGRKKNPINQKGSIYLSEPSPTDERNVCLNKGKVSAPV

TGNSNQKEISSPVLNTEVVQDMAKHIDTETEALQGIDSVDNKSLAPEEKDHLVLDLMVNQ

DKLQAKTPEAADAEVEITVLERELNDVPTEDPSDGALQSEVDKNTSKRKREAGVGKNSLQ

RGKKGSSFTAKVGKSRVKKTKISRKENDIKANGTLMKDGGDNSADGKENLALEHENGKVS

SGGDQSLVAGETLTRKEAATKDPSYAAAQLEVDTKKGKRRKQATVEENRLQTPSVKKAKV

SKKEDGAKANNTVKKDIWIHSAEVKENVAVDENCGDVSSDGAQSLVVEKSLAKKEAAAKD

PSNAAMQLEFDDNKCKHGKEGIVERSSLQSGKKGSSSRVEVGKSSVKKTKKSEKGSGTEA

TDTVMKDVGDNSAKEKENIAVDNESRKVGSGGDQSPVARKKVAKSAKTGTKAEKESKQLR

VNPLASRKVFQDQEHEPKFFIVSGPRSQRNEYQQIIRRLKGKCCRDSHQWSYQATHFIAP

EIRRTEKFFAAAASGSWILKTDYVADSKEAGKLLQEEPYEWHSSGLSADGAINLESPKKW

RLVREKTGHGALYGLRIVVYGDCTIPCLDTLKRAVKAGDGTILATAPPYTRFLNQNTDFA

LISPGMPRDDVWIQEFIRHEIPCVLSDYLVEYVCKPGYALDKHVLYNTNSWAEKSFNKMQ

LRADLCVYH

>Esalsugineum_BCP1

MLESGLPSKTFWGVRFALVGFNPIDGNTLRSKLLSGDGVDVGQYSQSCTHLIVDKLVYDD

PVCVAARSSGKVVVTRSWVDHSFDIGMLIDANSVLYRPLKDLNGIPGSKSLVVCLTGYQR

QDREDIMTMVDLMGGQFSKPLVANRVTHLICYKFEGEKYELAKRMKRIKLVNHRWLEHCL

KNWKLLPEADYEISGYELEMMEASARDSEDEAEDASVKRANNSPLGLRVGAAAAVGMSKS

EGKDIPVVQTNSGVQDGSSFCNTSKDNWLTPKKMDRPLEAMANTDAVAAQQNNYQRSSTF

QEAFNYASPVLVKKASEQEMGRMATDDSASINMSIRRHSSLATYSRKTLQRSPETGTLGN

ESSGPNGTLRMDDRGLKASTAFNISASKSTSSVERTTLFEDLDKIDKFHGKESPPSLPQA

KFTDGSVSSKGSQKVHNNSEASIPPLASLLLQEFRTGSPKESLRPVPSINDPTESGEVGR

KSPASVLNTELLRSNVVPVEGAISTAENIISKCAQDEIPERSSTEPMVGNKILQELRSGS

PEQNLRVVPNISDHIEAREVTRKLDVSDSATRLFNSSVVPMEADIRTPEITTLKGILDEV

PERSVTEAVMRRSSNSPGSGLIGMKDQLETELPKKTTIPRKSLGTRGRKKNPITQKGSIY

LSEPTPKDKRTDSLQKGNVSAPITDNGNPKEISSPILNTEAVPELAKQIDSETEALNGME

SEDNKSYVPEEKDHLGSQDKLQAKTPEEADAEVEIAMLEPELNDVPTKDPSDGALQLEVD

KNKSKRTREATVGKNSLQSGKKENSSTAEIGKSTVKKTKKSRKEHDAKANDTVMKDIRDN

SANAMENFALEKKSGKVSSGGDQSSVAGETLARKEAETKDPSDAVVKLGVDKTKGKRRKE

ATVEKTRLPSGKKRSSPTVEFGKPSVKKAKNSKKDDDAKANHTVVKDIGINSAEMMENLA

VDKKSEDDSSDGAQSPVAGEPVARSGKKGSSCTVEVGKSSVKKPKKSKKERGAKATDTVM

KDIGDNSAEEKENIAVDDKSGTEKSPVSGKPLARKEVAKSAKRVTKADKESKQLSVKPLA

RRKVLQDQEQQPRFFIVSGPRSQRKEYQQIIRGLRGKCCRDSHQWSYQATHFIASEIRRT

EKFFAAAASGSWILKTDYVADSKEAGKLLPEEPYEWHSTGLSADGAISLESPRKWRLIRE

QTGHGALYGMRIVVYGDCTIPSLDTLKRAVKAGDGTILATAPPYKRFLNENTDFALISPG

MPRDDLWIQEFIRHEIPCVLSDYIVEYVCKPGYALDKHVLYNTNSWAERSLNKLLLSAEE

KVVA

>Crubella_BCP1

MQESSSTPKTFLGVQFALVGFNPIHENSLRSKLVSGGGVDVGQNSQSCTHLIVDKLVYDD

PICVAARSSGKAVVTGSWVDHSSDIGTLLDANSVLYRPLRDLNGVPGSSTLVVCLTGYQG

QDREDIMTMVDLMGGNFSKPLVANRITHLICYKFEGEKYELAKRMKRIKLVNHRWLEDCL

KNWKLLPEVDYEISAYELEMMEASARDSEDEDASVKCVNNSPLGLRVGARAAAEMSKPGE

KNIPVVQTNSGAQEVSSLCNTSKDNGLTPKNTDRPVEAVVSTDPSVAQQHNYQRESTCQD

TSNFLSPIRLANKSRELGMRKMETDSQMSINRSIRRHSSLATYSRKTLQRSPETNTLGKE

SSGQNGSLQMDDKGQKASTSFNISASKSGSSVERTILFEDFDKIDKLHGKESPPMMPQAK

FTDESVSSKDSQKVHHKSKASIPSPTSLLLQELRPSSPKENLRLVTSINDPTESGEFGHK

SAASVFNTKSESSLAEAMAGNVLLHEPRSVSPKQNLRVVPNLSDHAGGREAAHNLDLSDS

AARLFSSGVVPMEADMRTPENSTKEEALDEVPERSVTEPVMRISSSTSPGPGLIEMKDDQ

ETELPKKKTVPKKSLGTRGRKKNPINEKGSIYLSEPTRKDERNDCLNRGQVSVPKSGNRN

QKKISSPVLNTEVVPEMAKHIDSETEAFQGIDSLDNKCLAPEEKDHLGLDLMVNQGKLQA

KTSEEADADVEITVLELELTDVPVEDPSGALQLEVDKIKSKRKREATVCKNSLQSGKKES

SSTVKVGKCSVKKTKKSRNEHDTEAIGTLMKDIGDNSSDEKDKLAFEHISAKVSSGGDQS

PVAGETLARKEGATKDPSDAEVQSEVDTNKSKRRKEATVENTWLQKPCVKKVKYSRKEDD

AKANNTVMNDIWVSSAEVQENVAVDKKSGDVNSDGAQSLVAGKLLVRKEAAAKDPSNAAM

QLEVDKNKCKHGKKAIVDRSGLQSGKMGSSSTVEVGKSSVKKTKKSKKGSGAKTTDTVMN

NIGDNSAKDKENIAADSKSGKIGSGGDQSPVSGKALARKKVAKSATTGTKVDKESTQLRA

NPLASRKVLQDQEHEPKCFIVSGPRSQRNEYQQIIRRLKGKCCRDSHQWSYQATHFIAPE

IRRTEKFFAAAASGSWILKTDYVADSKEAGKLLPEEPYEWHSSGLSADGAISLESPRKWR

LVREQTGHGALYGLRIVVYGDCTIPSLDTLKRAVKAGDGMILATAPPYTRFLNENTDFAL

ISPGMPRDDIWIQEFIRHEIPCVLSDYLVEYVCKPGYALDKHVLYNTNSWAERSFNKMQL

SADVCTI

>Cgrandiflora_BCP1

MQESSSTPKTFLGVQFGLVGFNPIHENSLRSKLVSGGGVDVGQNSQSCTHLIVDKLVYDD

PICATARSSGKAVVTGSWVDHSSDIGTLLDANSVLYRPLRDLNGVPGSSTLVVCLTGYQG

QDREDIMTMVDLMGGNFSKPLVANRITHLICYKFEGEKYELAKRMKRIKLVNHRWLEDCL

KNWKLLPEVDYEISAYELEMMEASARDSEDEDASVKCVNNSPLGLRVGARAAAEMSKPGE

KNIPVVQTNSGAQEVSSLCNTSKDNGLTPKNTDRPVEAVVSSDPSVAQQHNYQRESTCQD

TSNFLSPIRLANKSRELGMRKMETDSQMSINRSIRRHSSLATYSRKTLQRSPETNTLGKE

SSGQNGSLQMDDKGQKASTSFNISASKSGSSVERTILFEDFDKIDKLHGKESPPMMPQAK

FTDESVSSKDSQKVHHKSKASIPSPTSLLLQELRPSSPKENLRLVTSINDPTESGEFGHK

SAASVFNTKSESSLAEAMTGNVLLHEPRSVSPKQNLRVVPNLSDHAGGREAAHNLDLSDS

AARLFSSGVVPMEADMRTPENSTKEEALDEVPERSVTEPVMRISSSTSPGPGLIEIKDNQ

ETELPKKKTVPKKSLGTRGRKKNPINEKGSIYLSEPTRKDERNDCLNRGQVSVPKSGNRN

QKKISSPVLNTEVVPEMAKHIDSETEAFQGIDSLDNKCLAPEEKDHLGLDLMVNQGKLQA

KTSEEADADVEITVLELELTDVPVEDPSGALQLEVDKIKSKRKREATVCKNSLQSGKKES

SSTVKVGKCSVKKTKKSRNEHDTEAIGTLMKDIGDNSSDEKDKLAFEHISAKVSSGGDQS

PVAGETLARKEGATKDPSDAEVQSEVDTNKSKRRKEATVENTWLQKPCVKKVKYSRKEDD

AKANNTVMNDIWVSSAEVQENVAVDKKSGDVNSDGAQSLVAGKLLVRKEAAAEDPSNTAM

QLEVDKNKCKHGKKAIVDRSGIQSGKMGSSSTVEVGKSSVKKTKKSKKGSGAKTTDTVMN

NIGDNSAKDKENIAADSKSGKIGSGGDQSPVSGKALARKKVAKSATTGTKVDKESTQLRA

NPLASRKVLQDQEHEPKCFIVSGPRSQRNEYQQIIRRLKGKCCRDSHQWSYQATHFIAPE

IRRTEKFFAAAASGSWILKTDYVADSKEAGKLLPEEPYEWHSSGLSADGAISLESPRKWR

LVREQTGHGALYGLRIVVYGDCTIPSLDTLKRAVKAGDGMILATAPPYTRFLNENTDFAL

ISPGMPRDDIWIQEFIRHEIPCVLSDYLVEYVCKPGYALDKHVLYNTNSWAERSFNKMQL

SADVCTI

>Brapa_BCP1

MESGLPCMTFSGVRFALVGFNPTDANTVRSKLMSGGGVDAGQYSQEYTHLIVDKLVYDDP

VCVAARSSGKVVVTGSWVHHSFDAGMLIDANSVLYRPLRDLNGIPGAKGLIVCLTGYQRQ

DREDIMTMVDLMGGQFSKPLVANRVTHLICYKFEGEKYELAKRMKRIKLVNHRWLEDCLK

NWKLLPEVDYQISGHELEMMEASARDSEDEAEDASAKRANTSPLGLRVSAVSAVEMSKSR

GKDIPVVQTKLDEHGGSSRCNTSKEDWLTPKKMEATVSTDPITAQQSAGTFQNTSTYASP

VPVNKASERGMGKMETDDSTPINVSIRRHSSLATYSRKTLQRSSETSTLGNESSCQNGTL

RMDDRALKASTAFNISASKSSSSVERKTLFEDLDKTDTLHGEESTPLLPQVKLTDGSVSS

KGSPPAMSTAENIISKCAPDEIPERSSIEPVVGNMLLQEPRSGSPKQNLKVVPNINDNTE

GEEVAHKLDVSDSATRLCSSSVVLKEADTWTAEILKGGLDERSVTEPEMMRSSTSPVETE

PPKKKTAPRKSLGTRGRKKNPTISQKGSIYLSEPTPKDEHSDGLIKGNVSELATDDSNQK

EIPSPVLNAEAVPEMAKGSVSETEALDRIDSDKQQVKTPEEAGAEVEIKMLESELAEVPT

KDPSDGALQSEISKSKNERMSEKKGSSSRAEAADSGVKKTKKSRKEDDAKANDTVMEDIT

VLENKLGKVSSGGDQSPAAGETLVSTEAETNDQSDAAVQSGVDKDNGKLRKKATVGKTRL

QSGKKRNSSAVEVEKASVKKTKKSKADDETEANDTVMNGIEIDSAEVKEDAAVDKKPEDV

SSDRTQSPVARKPSTRKETAAKNLSNAAMQLEVPKNKRKIRKEPAVERSSLQSGEKESSS

TVEVGSSSVKKSKKSKKERGAKASDTVMKDIEDNSAEEKESTEVDNKSGPGIDKRPKKKA

AKTAKTGAKTAKESNQLSSSKKALQEQRQEPKHFIVSGPKVQRKEYQKILRLLNGKCCRD

SHQWSYQATHFIAPEIRRTEKFFAAAASGSWILKTDYVADSKEAGKLLPEEPYEWHGTGL

SADGSISLESPRKWRLVKEQTGHGALHGLRVVVYGDCAVPSLDTLKRAVKAGDGTILATA

PPYTRFLNQNTDFALISPGMPRDNVWVQEFIRHEIPCVVADYIVEYVCKPGYALDKHVLY

NTNAWAERSFNKLQLSAED

>Bstricta_BCP1

MDSGIPPNTFSGVRFALVGFNPIHGNSLRSKLVSGGGVDVGQYTQSCTHLIVDKLVYDDP

ICVAARSSGKVVVTGSWVDHSFDIGMLIDANSVLYRPLRDLNGIPGSKTLVVCLTGYQRQ

DREDIMTMVDLMGGQFSKPLVANRVTHLICYKFEGEKYELAKRMKRIKLVNHRWLEDCLK

NWKLLPEVDYEISGCELEMMEASARDSEDEAEDASVKRANNSPLGLRVGAVAAVEMSEPG

GKDIPVVQTNSGAQEGSSLCNTSKDIWLTPKKMDRPLEAMVSTDLCVAQQHNYQRESTFQ

DTSNYASPVRVANKTPEQGMSNMETDGSTSINRSIRRHSSLATYSRKTLQRSPKTNTLGK

ESSGQNGSRQMDDKGLKASTAFNISASKSGSSVERTTLFEDLDKIDKLHGEESSPMMPQA

KFTDGSVCSKDSQKVHHNSETPPTSLLLQELRPSSPNENLRPVTSINDPTESEEVGHKSP

ASVLNTKLLSSNVVPMVDALSTAENIISNCARDEIPERSLTEPMAGNVLVQEPRSGSPKQ

NLRVVPNLSDHTEGREAAHKLDLSDSAARLFNSGVVPMEADIRTLENSTMKGALDEVPER

YVTEPVMRRSSTSPGSGLIGMKDKQETELPKKKTAPKKSLGTRGRKKNPINQKGSIYLSE

RTPKDERNDCLNKGKVSAPVTGNTNQKEISTPVLNTEVVPEMAKHIDSETEALQGIGSVD

SKSLAPEEKDHLVLDLMVNQDKLQAKTPEEADADVEITVLELELNDVPVEDPSDDALQLE

VEKNKSKRKREATVGINSLQSGKKESSSTAEVGKSNVKKTKKSRKEHDTKAKGTLMKDIG

DNSADEKENLALKHKSGNGSSGGSQGPVAVETLARKEAATKDPSDAGVKFEVDTNKGKRR

KEATVEENRLQKPSVKKAKNSRKEVGAKAKNTVMKDIWINSAEVQENVAVDKKSGDASSD

GAQSLVAGKLLAKMEAAAKDPSNAAMQLEVDKNKCKHRKEAIVDRSSLQSGKKESSSTVE

VGKSNVKKTKKSKKGSGAKATDSVMKDIGDNSAKEKENIAVDNKSGKVGSGGDQSPVSGK

PSATKKVAKSAKTGTKVDKESKQLRVPLASRKVLQDQEHVPKFFIVSGPRPQRKEYQQII

KRLKGKCCRDSHQWSYQATHFIASEIRRTEKFFAAAASGSWILKTDYVADSKDAGKLLPE

EPYEWHSTGLSADGAINLESPRKWRLVREQTGHGALYGLRIVIYGDCTIPSLDTLKRAVK

TGDGTILATAPPYTRFLNQNTDFALISPGMPRDDVWIQEFIRHEIPCVLSDYLVEYVCKP

GYALDKHVLYNTNSWAERSFNKMQLSADVCTI

>Mperfoliatum_BCP1

MLEPGTFSGVRFALVGFNPVDGNTLRSKLVSGGGVDVGQYSQNSTHLIVDKLVYDDPVCV

SARSSGKVVVTGSWVDHSFDVGMLTDANSVLYRPLRDLNGIPGSKSLVVCLTGYQRQDRE

DIMAMVGLMGGQFSKPLVANRVTHLICYKFEGEKYELAKRMKRIKLVNHRWLEDCLKNWK

LLPEVEYEISGYELEMMEASARDSEDEAEDASVKRANTSPLGLRVGALAAVEMSKSGGTD

IPVVQTYPGDQDGSSLCNTSKENWLTPKNMEAMVSTDAVAAQQHDHQGASTFQNTSNYAS

PVPVNKTSERGTGRVETDDSTSINMSIRRHSSLATYSRKTLQRSPETSTLGNESSGQNGT

LRIDDRALKASSAFNISASKSGSSLERTILFEDLDKINKLHGEESPPLLPQAKLSDGSVS

SKGSQKVHPSSEESIPPPASLSLQELRTGSPKENLRPVPSVNDPAERGEVGHKLPASDLN

VVPTEEAMSTAEKIISKCALDEIPERSSTEPMAGNILLQAPRSGSPKQNLRVVPNTSDHT

EGREVAHKLDVSDSATRLFNSSGVLMEADIWTAENITLKGAIDEVPERSVTEPVMMRPST

SPGSGFIGMKDQVETELPKKKTAPRKSLGTRGRKKNPINQKGSIYLSEPTPKDEHTDCLI

KGEVSAPATDSGNQKEILSPVLNAKAVPEMAKSIGSETEALKRIDSDVNQSYAPGEKVHL

VTQDILQAKTTEEANAEVEITMPESELTDVPSKDPSDGALQLEVKKSKRTREANVGKNNP

QNGKKESSSTAEVGNSVVKKTKKSRREDDAKANDTVMKDARDKSTYEMENFASENKSGKL

SSGKDQSPVAGETLVRTEAETNDPSDAAVQLGVDKNSGKRRKEATVEKDRLQSGKKRSSS

TVEFEKPSVKKVTNSKKDGDAKANDSLMNDIGINSAEVKENVAVDKNPEDVSSDGAQSPV

AKKPLATKETAAKYSSNAAIQLEVGKNKRKNIKDSTVEISSVLSGKKRSSSTVEVGKSSV

KKRKKSKKERGSEATDTVMKDIVDNSAEEKENIAVDKKPGRDKSPVSGKPLARKEAAKSA

KTGTKADKESKQLSSKPLASKKVLQEQKQEPKFFVVSGPKSQRKEYQKIIRLLKGKCCRD

SHQWSYQATHFIAPEIRRTEKFFAAAASGSWILKTDYVADSKEAGELLPEEPYEWHSTDL

SADGAISLESPRKWRLVKEQTGHGALYGMRVVVYGDCTIPSLDTLKRAVKAGDGTILATA

PPYTRFLNQNTDFALISPGMPRDDVWIQEFIRHEIPCVLSDYLVEYVCKPGYALDKHVLY

NTNTWAERSFNKLQLSAEN

>Rislandica_BCP1

MMDSGFSSTTFSSVRFVLVGFNPTDGNTLRSKLVSGGGVDVGQYSQNCTHLIVDKRVVYD

DPICVTARSSGKVVVTGSWVHHSFDTGILLDANSVLYRPLIDVNGIPGSKSLVVCLTGYI

RQDREDVMTMVDLMGAQFSKPLVANRITHLICYKFEGEKYELAKRMKRIKLVNHRWLEDC

LKNWKLLPEADYEISGYELEIMEASARDSEDEAQDASVKRANNSPLGLRVGVVAAVGTSK

PEDKDIPDVQTNSGVQEGSSFCNTSKDNWLTPKKTDKPFEAMVSTSAVVPEQQSYQRASP

NYDSHIKVANKTPEKATSKMEPDESTSVNKSIRRHSSLATYSRKTLQRSPEPNTLGTESS

GHNDSLRMDDKGLNISASKSVSSMERTTLFEDLDKTDKLHGEESPPMLPQAKFTDGSVSS

KGLTNVHHNSESSITPTASLLLPEVNLRPVPSINDGGEVGHKSPASVFNTKVLSSNVVPM

EDAASTAEHIISKCARDEIPERSSTEAIVGNILLQEPRAGSPKQNLRVVPNTSDHIEGRE

VAGNLDLSDSAIRLFNPSVVPMDAAIWTPENITLKGELDEVPERSVTEPEIRRSSTSPGS

GLIGMKDKLETELTKKKTATRKSLGTRGRRKNPVNQKGSIYLSEPIPKDEHTDCLNKGKV

SAPATDIVNEKEEVSSPVLNTEVVPDMAKHIDSETEAVQGTDSVDKKSYAPEKKDHLELD

LMVNQDKLQANTSEEADAELGIRMLEPKLNDVPTKDSSDDGSLQIDSDKSKSKRKRKATV

AIQSGKKESSSTTDELGTDTLMKDIGDISADEKESLVLENKSGKVSFGGDKSPVAEETLS

RKEAAAIDPSNAAVELVVNKNKGKRRKDAAVEENRLRKPSVKKAKNSKKEDNAKENSTVM

KDTRINSAEVKVDVAVDKEGSSVRVEAGKPSFVKKTKKFKKGLCDTVMKDIGDNSAKEEE

NDVVDNKCGKVGSGGDQSTVSEKPLARKKVAKSAKTDKVDSGGDKSSVSVKPFARKKVAK

SAKTGTKVVEESKQLIVKPLASKKVLQDQEQEPKFFTVSGPRPQRNEYQKIIRSLKGKCC

RDSHQWSYQATHFIASEIRRTEKFFAAAASGSWILKTDYVADSKEAGKLLPEEAYEWHST

GLSADGAISLEAPRKWRLVREQTGHGALYGLRIVVYGDCTIPSLDTLKRAVKAGDGTILA

TAPPYTRFLNQHTDLALICPGMPRDDVWIQEFLRHEIPCVLSDYLVEYVCKPGYPLDKHV

LYNTNSWAERSFNKMQRSVDV

>Itinctoria_BCP1

MLESGTFSGVRFALVGFNPVDGNTLRTKLLSGGGVDVGQYSQNFTHLIVDKLVYDDPVCV

SARSSGKVVVTGSWVDHSYDVGMLTDANSVLYRPLRDLNGIPGSKSLVVCLTGYQRQDRE

DIMAMVGLMGGQFSKPLVANRVTHLICYKFEGEKYELAKRMKRIKLVNHRWLEDCLKNWK

LLPEVEYEISGYELEMMEASARDSEDEAEDASVKRANTSPLGLRVGAVAAVEMPKSGGTD

IPVVQTNPGDQDGSSLCNTSKENWLTPKKMEAMVSTDAVAAQQHNHQGASTFQNTSNYAS

PVLINKTSERGTGRVETDDSTSINMSIRRHSSLATYSRKTLHRSPEISTLGNESSGQNGT

LRIDDRALKASSAFNISASKSGSSAERTILFEDLDKINKLHGEESPPLLLQAKLSDGSIS

SKGSLKTMSTAEKIISKCALDEIPERSSTEPMAGNILLQAPRSGSPKQKLRVVSNTSDHT

EGREVAHKLDVSDSATRLFNSSGVLMEADIWTAENITLKGALDEVPERSVTEPVMMRSST

SPGSGLIGMKDQVETELPKKKTAPKKSLGTRGRKKKPINQKGSIYLSEPTLKDEHTDCLI

KGEVSAPQTDNGNQKEILSPVLNAEAVPEMAKSIHSETEVLKRIDSEVNQSYTPGEKVHL

VTQDILQAKTTEEADAEVDTMPESELTDVPSKDPSDGALQLEVKKSKRTREANVGKNSPQ

NGKKESSSTAEARSSVVKKTKKSRKEDDAKAKDTVMKDVRDKSTYEMDNFASENKSGKVS

SGKDQSPVAGETLVRTEAETNDPSDAAVQLGVDKNSGKRRKEATVEKDRLQSGKKRSSST

VEFEKPGVKRATNSKKDGDANAKDSLMNDIGINSAEVKENVAVDKTPEDVSSDGAQSPVA

KKPIATKETAAEYPSNAAIQLEVGKNKRKNRKDCTVEINSVQSGKKGSSSTVEVGKSSVK

KRKKSKKERGPEATDTVMKDIMDNSAEEKENIAVDKKSGRDKSPVSGKPLARKEAAKSGK

TGTKADKESKELSSNPLAKLIXSKELSSNPLASKKVLEEQKQEPKFFVVSGPKSQRKEYQ

KIIRLLKGKCCRDSHQWSYQATHFIAPEVRRTEKFFAAAASGSWILKTDYVADSKEAGEL

LPEEPYEWHSTDLSADGAISLESPRKWRLVKEQTGHGALYGMRVVVYGDCTIPSLDTLKR

AVKAGDGTILATAPPYTRFLNQNTDFALISPGMPRDDVWIQEFIRHEIPCVLSDYLVEYV

CKPGYALDKHVLYNTNTWAERSFNKLQLSAEN

>Lannua_BCP1

MLESEFPSKTFSGVRFALVGFNLIDGNTLRSKLLSGGGVDVGHYSQNCTHLIVDKLVYDD

SICVAARSSGKVVVTGSWVDHSFDIGMLIDANSVLYRPLRDLNGIPGSKSLVVCLTGYQR

QDREDIMTMVDLMGGQFSKPLVANRVTHLICYKFEGEKYELAKRMKVIKLVNHRWLEDCL

KNWKLLPEVDYEISGYELEMMEASARDSEDEAEDASAKRANNSPLGLRVGAVAAVEMSKP

RGNDIQVVQTNSCAKEGSPFCNASKDWLTPKNMDRPLEATASTDAATAQQHNFPRASTSQ

DTSNYAAPVLVNKTSEQGTSRMETDESTSTNMSIRRHSSLATYSRKTLQRLSETSTLGNE

SSVLNGTLGINDRRIYASTAFNISASKSGSSVKRTSLFEDLDKIDKLLGEESPPLLPQAK

FTDGSVSSKVSQKVHHNSEASLLLLELRTCSPKENLRPVPSINDPTESEEVGHKSPASVL

NTKLLSSNLVPREDAISTAEDNISKCAGDKIPERSSTESMVGNILLQERISDSPKQNLRV

VPNISEHTEGREVAHKLDVSDSATRLLNSSAVPMEADNWTPENIALNDALDEVPERSITE

PGLRRSSTSPGSGLVGIKDQLETELPKKKTASRKSLGTRGRKKNPISQKGSIYLSEPTPK

DERTSCLNKGKVSAAVTDNCNQKEIPSPVLNTEAVPEMAKHNDSETEALKGTDCADNKPY

APEEKDHLVLDLIVNQDKLQAKTPEKADAEVDITMLEPELNTASTRDPSDGALQFEVDKS

KSKRMRETTVGKNSLQSGQKESYSTAKVGKSSVKKTKKSRKEHDAKANYTVMKDIGDNSA

KEMENCGLENKSGKVSSVGDQSPVAGETLASNEAETKDPSDAAVQLVVDKNKGKHRKEDT

VEKNRLQFGKKRSSSTVGLGKPNVKKAKNSKKEDDAKANDTVMKDMGINSAEVRDKKSWD

VSPDGAQNLVAGKPLARKEAAAKCPSNAAMQLEVDKNKRKHRQEATVESSLQSRKTSSST

VEVGKSSVKKPKKSKNERGIVATDTVMKDIGDNSSEEKENIAVDNKSGREKSTVSGKSLA

RKEAAKSAKTGTKADTEAKHLSAKPLASRKVLQDQEQEPKFFIVSGPRSQRKEYQQIIRR

LKGKCCRDSHQWSYQATHFIAPEIRRTEKFFAAAASGSWILKTDYVADSKEAGKLLPEEP

YEWHSSGLSADGAISLESPRKWRLVRERTGHGALYGMRIVVYGDCTIPSLDTLKRAVKAG

DGTILATAPPYTRFLNQKTDFALISPGVARDDVWIQEFIRHEIPCVLSDYLVEYVCKPGY

ALDKHVLYNTNSWAERSFNKMMHQLCAEEEEEEEEEEEEGEEEEEEEEEEAVASHNDVT

>Mmaritima_BCP1

MQDSGYSPNTFLGVRFALVGFNPIHGNSLRSKLESGGGVDVGQYSQTCTHLIVDKLVYDD

PICVTARSSGKVVVTGSWVDHSFDTGVLLDANIILYRPLRDLNGIPGSKALVVCLTGYQG

QDREDIMIMVDMMGGQFSKPLVANRVTHLICYKFEGEKYELAKRMKRIKLVNRRWLGDCL

KNWKLLPEVDYEISDYELEMMEASARDSEDEAEDATLKRANNSPLGLRVGAVAAVEMSKP

GGKDIPVVQASSGAQEGSSFCNTSKGNWLTPRKTDRPFEAMVSTDPAAAHASNYASPIRV

ANKTPEQGMSKMETDGSTSINRSIRRHSSLATYSRKTLQRSPETNTLGKESSGQISSLRM

DDKGLKSSTAVNISASKSGSSVERTTLFEDLDKIDKLHGEVFPQMMPEAKFTDGSVSSKD

SQMVHHNSEASIPPPASLLLQELRPSSPNGNLRPVTSINFPIENGEFGHKSPASVLKPLS

SNVVPMEDTHSTAENIISNCSRDEIPERSLTELVAGNVLLQEPRSSSPKQNLRAVPNLSD

HTEGREAAHDLSDSAVRLFNSGVVPMEADIQTPENSNMKGALDEVPERSVTDPVMRRSST

SPGSGLTGMKDKQEIDLPKKKTAPKKCLGTRGRKKNPINQKGSIYLSEPTPKDECNDCPS

KGKVSAPVTGNSNQKEISSPVLNTEVVPEMAEHIDSETEAVQGIDSVDNKSLAPEEKGHF

VLDLMVNQDKLEAKTPEEADADVETTVLEPEVNDVPVEVPSDGALQLEVDKNKSKRKREA

TVGKNSLQSGKKETSSTAEVGKSSVKKTKKSRKEHDTKANGILMKDAGDNSTDEKENLEL

EHKSGKVSSGGDQSPVAGETLARKEAATQDPSDAAVQFEVVTNKGKRRKEATVEENKLQK

PSVKKAKNSRKEDGVKANNTVMKDIWINSAEIKENVAVDKKSGDVSSDGAQSPIAGKLLG

RKEAAAKDPSNAAMELEVIENECKHGKEAIVERSSLQSGKNGSSTVDVGKSGVKKTKKSK

KGSGAKTTDTVMKDIGDNSSKEKENIAVDNKSGKVGSGGDQSPVSGQPLAKKKVAKSAKT

SSKVDKASKQLRVKPLASRKVLQDQEHGPKFFIVSGPRSQRNEYQQIIKRLKGKCVRDSH

QWSYQATHFIAPEIRRTEKFFAAAASGSWILKTDYVADSKEAGKLLAEEPYEWHSTGLSA

DGSINLESPRKWRLVREQTGHGALYGLRIVVYGDCTIPSLDTLKRAVKAGDGTILATAPP

YTRFLNQNTDFALISPGMPRDDVWIQEFIRHEIPCVLADYLVEYVCKPGYALEKHVLYNT

NSWAERSFNKMQVSADVCSV

>Tarvense_BCP1

MLESGFPSKTFSGVRFALVGFNPIDGNSLRSKLLSGGGVDAGQHSQTCTHLIVDKLVYDD

PVCAAARSSGKVVVTGSWVDHSFDIGMLIDANSVLYRPLRDLNGIPGSDSLVVCLTGYQR

QDREDIMAMVDLMGGQFLKPLVANRVTHLICYKFEGEKYELAKRMKKIKLVNHRWLEDCL

KNWNLLPEDGYEISGYELEMMEASARDSEDEAEDASVKRANNSPLGLRVGAVAAVGLSKS

GGKDIPVVPSNSGAQDGSSLSKDNWLTPKKMDMPLEAMVATDAVAAQQHNYQRASTVQDT

SNHASPVPVNKTSERGMETDDSTSVNMSIRRHSSLATYSRKTLKRSPETSTLENESSEAN

GTLRMDDRALKASNASHISASKSSYCVERTTTLFEDLDKTDKLHGKASPPLMPQAKFTDG

SVSSKGSRMVQHNREACIPPPASLLLQELRTASPKENLTTVPSTNDPTESGEVGHKAPAS

VLNTELLSSNVAPVEDAISTAENIISICARNEIPERSSTEPMAGNILLQDLRSSSPKQNL

RVLPNISDHTEGREVAHKLDVSDSATRFFNSSVPREADIWTPETVTLEGAVDEAPEGSVT

EPVMRRSSTSPGSGLDGMKHQLETELPKKTTAPRKSLGTRVRKKNPISKKGSIYLSEPTN

SKDERTDCLYEGKPSAPVTDSGNQKEISSPALNTEALSEMAKHIDSETEALKRLDSEDNK

SSAPEEKDHLVNQDKMQAKASEEADADVEITMVEPERNDVPTKDPSDGASELEVDKNKRK

RTREATVGKDSLQSGEKESSSTAEVGKSSVKKTKKSRKKHDAKANDAVMKDISGNSANER

EKFALENISGKVSSGGGDQSSVAGETLARKEAETKDTSNAAVQLGVDKHKGKRRKEAIVA

KNKLQSGKKRSPSTVELGKTSVKKAKSTKKEDDAKVNDTVMKDIGTNSAEEKENMAVDNK

PGRDKSPVPGKPVARNEAAKSAKTGTKADKVSKQLSSKPLASRKALQGQEQEPKFFIVSG

PRSQRKEYQQIIKRLKGKCCRDSHQWSYQATHFISPEIRRTEKFLAAAASGSWILKTDYV

ADSKEAGKLLPEEPYEWHSAGLSADGAINLEAPRKWRLVREQTGHGALYGMRVVVYGDCS

IPSLDTLKRAVKAGDGTILATAPPYTRFLNQNTHFALISPGMPRDDVWIEELIRHEIPCV

LADYLVEYVCKPGYPLDKHVLYNTNSWAERSFKKLQHSAQE

>Salba_BCP1

MMMESGLTSKTFSGVRFALVGFNPTDGNTLRSKLMSGGGVDVGQYSQDFTHLIVDKLVYD

DPVCVAARSSGKVVVTGSWVHHSFDVGMVIDANSVLYRPLRDLNGIPGSKSLVVCLTGYQ

RQDREDIMTMVDLMGGLFSKPLVANRVTHLICYKFEGEKYELAKRMKRIKLVNHRWLEDC

LKSWKLLPEVNYEISGHELEMMEASARDSEDEAEDASVKRANTSPLGLRVGAVAAVEISK

SRGKDIPVDQTNLNEQGGSSRCNTSKEDWLTPKKMEATVSTGAVAVQQRASPCQNASSYA

SPVPVNKTSERGMDKMETDDSTPVNISIRRHSSLATYSRKILQKTPETSTLGNESSCQNG

TLKMDGRAPKASTAFNISASKSGSSVERTTLFKDLDKIDKLLGEESTPLLPQAKLTDGPV

SDEGSEKIHHSSETSIPPPATSTAENMISKCAPERSSTVPMVGNILLQEPRSGSPKQNLT

VVPNIDDNTEETNGMLMEADKRTAEVINLKGALDEVPERSVTAEPVMMRSSISPGSGFTG

MKDQVETELPKKKTAPKKSLGTRGRKKNPINQKGFIYLSEPTPKDEHTDGLIKGKELALA

TDNSNQKEKSSPVLNAEAVPEMAKRSDPETEAFERIDSDKLQTKTPEEADAEVETTRLES

EVTEVPTKDPSDDALQLEVKKSKVKRTREATVGKNSLQSVKKGSSSRAEAGNSGVKKTKK

SRKEDDAKANDTVMEDIRDKSANEMENIALENKSGDVSSGGDQSPAAGETHVSTEAETKD

PSDGAVQLGVDKNNGKHKKEATNGKNKLQNGKKRSSSAVEVGKDSVKKAKKSKKDDDAKA

NDTVMEDTGINSAEVKENVAANKKPDDLSSDGTRSPDAVIKETAAKNPSNAAMQLEVQKN

KRKNRKEPTVERSSLQSGEKESSTTVEVGTSSVKKPKKSNSERGAKASDTVMRDVGDDNS

AEEKENTAVDNKSGRDKSTVSGEPSSRKKAAKSAKTGTKVGKESKQLSSKPLASKKALQE

QKQEPKHFIVSGPKVQRKEYQKIIRLLKGKCCRDSHQWSYQATHYIAPEIRRTEKFFAAA

ASGSWILKTDYVADSKEAGKLLTEEPYEWHNTGLSADGSISLESPRKWRLVKEQTGHGAL

NGLRVVVYGDCAIPSLDTLKRAVKAGDGTILATAPPYTRFLNQNTDFALISPGMPRDDVW

IQEFIRHEIPCVLADYIVEYVCKPGYALDKHVLYNTNTWAERSFNKLQRSAED

>Cmaritima_BCP1

MMEPGLPSKTFSGVRFALIGFNPIDGNMLRSKLLSGGGVDVGKLSQDCTHLIVDNLVYDD

PVCVSARSSGKVVVTGSWVHHSFDVGMIIDANSVLYRPLRDLNGIPGSESLIVCLTGYQR

QDREDIMTMVALMGGQFSKPLVANRVTHLICYKFEGEKYELAKRMKRIKLVNHRWLEDCL

KNWKLLPEAEYEISGHELEMMEASARDSEDESEGASVKRANNSPLGLRVSAVAAVEMSKS

KGKDIPVVQTNSAEQSGSSFCNTSKENWLTPKKMEPTVSTGAVAAQQRAGTIQNSSSYVS

PVPVTKISERGMGEMETDDSTPVNMSIRRHSSLATYSRKTLQRSPETTTLGNESSCQNDT

LRIDDRGLQASTAFNISASKSGSSVKKTTLFEDLDKVDKLHGEESIPLLPQSKLTDGYVT

NKGSLHSSESSIPPPAGLLLQEVRTGSAKENRMPVPSIKDPTERMVADHEAPAENIISKC

ALVERSSTEPMVGNILLQEPRSGSPKQNLRVVQNVDDNTEVNEVAHKSDESDSATRLSNS

SRVLLEADKWTSETISLKGASDEVPEKSVTEPVMMRSSTSPGSGLIGMKDQVETELSKKK

TAPRKSLGTKGRKKNPINQKGSIYLSEPTPKDDHTDVKGKDSAPAKDNDNQKEKSSTVLN

AEAVPEIAKRSDSETEALERIDSDKQQAKTPEAADADVEIAMPDFELTEVPTKDPSDGAL

QLEVKKSKNKRMRKATVGKNSLQSEKKESSSTAEVGNSGVKKTKKSRKEDDAKASDTVME

NCALENKSGKAISGGDQSPVAVEISVRSEAETNDPSDDAVQSGVDTSNGKLRKKAAVEKN

KLQSGKKRSSSAAEVEKTSVKKAKNSKKDDDAEANDTAMNGTVINSAEVKENVAVGKKPE

DVSVDVTKSPVARKPLARKETAAKNPSNAAVQLEVQKNKRKSRKEATVESSLQSGEKPKK

SKQECGAKAKDSVMKDIGDNSEKEKENTAVDNKSGRDKSPVSGEPSSRKKAAKSARTATE

AEKESKQLRSKSLASKKAVQEQEQEPKHFIVSGPKIQRKEFQKIIKLLKGKCCRDSHQWS

YQATHLIVPELRRTEKFFAAAASGSWILKTDYVADSKEGGKLLPEEPYEWHSTGLSADGS

ISLESPRKWRLVKEQTGHGALHGLRIVVYGDCAIPSLDTLKRAVKAGDGTILATAPPYTR

FLNQNTDFALISPGMPRDDVWIQEFLRHEVPCVLPDYIVEYVCKPGYSLDKHVLYNTNSW

AERSFNKLQLNAED

>Iamara_BCP1

MMESGFPSKTFSGIRFALVGFNLIDGNTLRSKLISGGGVDVGQYCQSCTHLIVDKLVYDD

PICVTARSNGKVVVTGSWVDHSFDIGMLIDTNSVLYRPLRDLNGIQGAKNLIVCLTGYQR

QDREDIMTMVDLMGGQFSKPLVANRVTHLICYKFEGEKYELAKRMKRIKLVNHRWLEDCL

KNWKLLPEAEYEISGYELEMMEASARDSEDEAEGATVKRNSPLGLRVGAAAVVESGGKDI

QTVQRHSDAQDGSSSKENWLTPKKTDKPLEAKVSTDVVPPAHQHDYQRASTLPETSNYTS

PIPVNNTSELEKGRVETDDTTSVNMSIRRHSSLATYSRKKSPETSTLGKGSSGQNGSLNI

SASKSDFSLERTNLFKDLDKIDKLHSEEPPWLLPQEKFTDGSVSSKGSEKVHKDSAASIP

PLASSLLQELRPGSPKENLNPVLSINDPTEVEEVGYKSHVVPMKDATPTSENIISKCLSD

EISKRSPTDPMMIDILLQVPRSSSPKQNLSVVSNISDHTEGREVERTLDVSDSATKSSVV

PMEADMWNPENITMKGALDEVPEPVVKRSNTSPRSGLIEMKENLETDMTKKKTAPRKTLG

TTRGRKKSPINQKGSIYLSKSTSKDDGTEVSAPIADNGNQKEIPSPVLNTEAVPEIAKHI

DSDTEAPKEIDSGHKSSAPEDKDDPVLGLIMNQDKLHKTPEEADAQMDITMLEPELNDVS

TKDPSDGGLQVEVHKTKSKRKRVASAGKNSLQSGKKESSSTVEVEKSSVKKTRKSRKEHG

AKANDTVMKDLKECSMKEKDCSDEDQSPVGETLARKEAETKDQSDDASVELEVDKNQGKS

NKLQSGKKRSSSTVKLGKPSVKKAKANNADMKDAVIDSAEVKKTVAVDKKSEDVSSDGDQ

SLVAKKTLPKKKAAGKHPSNAAKQLEADKDKKETTVEKSSLQEGKKGSSSTVEVSKSNVK

KLKIPKGGKATDISDNTAEEKENIAVSNKSDKETSPVTVKPKKTAKSAKSDTKEDKESKQ

LSSKPSTSKKILPEQEQEPKFFIVSVRLLSQRKEYQQIIKSLKGKCCRHSHQWSYQATHF

IASEIKRTEKFFAAAASGSWILKTDYVADSKEAGKLLAEEPYEWYGTGLSPDGTISLESP

RKWRLLREKTGHGALYGMRIVVYGDCTIPSLDTLKRVVKAGDGTILATAPPYTRFLNENT

DFAVVSPTLPRDDIWIQEFLQHQIPCVLSDYIVEYVCKPGYALDKHVLFDTTSWAERSFK

KMQVNANMSL

>Evesicaria_BCP1

MMESGLPSKTFSGVRFALVGFNPTDGNTLRSKLQSGGGVDVGQYSQDFTHLIVDKLVYDD

PVCVAARSSGKVVVTGSWVHHSFDVGMLIDSNSVLYRPLRDLNGIPGSKSLIVCLTGYLR

QDREDIMTMVDLMGGQFSKPLVANRITHLICYKFEGEKYELAKRMKRIKIVNHRWLEDCL

KNWKLLPEADYEISGYELEMMEASARDSEDEAEDASVKRANNSPLGLRVSTVATVKMPKS

EGKDIPVVQTKPDEQGGSSLCNTSKENWLTPKKMETTVSTDAVAAQQKADTSQNTSSYAS

PVQVNKTSERGMGKMETDDTTPINVSIRRHSSLATYSRKTLQRSPETSTLGNESSCQNGT

LRMDDRGLKASSAFNISASKSGSSLERTTLFQDLDKIDKVDGEVSPPPLLPQGKLTDGSV

SSKGSEKIHHSSEASIPLPTSLLPQELRTGSPKENLRPVPSINDPTERREVGHNSPAIST

AEKIISKCVPDEIPERSSVEPMVGNISLQEPRSGSPKQNLMVVPNINDNTEGKEVAHNLD

MSDSATKLFSSSGALMEADKWAALDKVPERSVTEPVMMRSSTSPGTGLTGMMDQVETELP

KKKTAPRKTLGTRGRKKNPISQKGSIYLSEPTPKDEHTDSLIKGNVLASATDDGNQKEIS

SRVLNAEVVPEMAKRSDSETEALDRVDSDKLQAKTPEEADAEAEITMLESELADVPTKDP

THGALQLGATKSNNKRTGKATVGKYSLLSGKKGSSSTAEVGNSGNKKTKKSRKEDDAKAN

DTVTEDIRYKSANEMESSALENESGKVSSGGDQSPATGETLVSTEAETKDPSDAPVQLAV

GKNNGKRRKEATGGKNKLQSGKKRRSSTVEPEKDSVKKTKKSKNDDETEANDAVMNDIGI

NSAEVNEKVAVDKKPEDVSSEGVQSPVARKPLARKEKVAKDDAAMQLEVPKNKRKNRKEA

TVERSSLQSGEKESSSTIEVGTSSVKKPKKSKKESAAKVTDTVMEDVDDNSAEEKENTAV

DNKSGRDKSSRKKAAKSAKTGTKAVKESKQLSKKALQEQKQEPKHFIVSGPKVQRKEYQK

ILRLLKGKSCRDSHQWSYQATHFIAPEIRRTEKFFAAAASGSWILKTDYVADSKEAGKLL

PEEPYEWHSTGLSTDGTISLESPRKWRLVKEQTGHGALHGLRIVVYGDCSIPSLDTLKRA

VKAGDGTILATAPPYTRFLNQNTDFALVSPGTPRDDVWIQEFIRHEIPCVLADYIVEYVC

KPGYALDKHVLYNTNTWAERSFNKLQLTARN

>Chispanica_BCP1

MESNLKTFSGVRFALLGFNPTDGNTLRSKLLSGGGVDVGQYSQECTHLIVDKLVYDDQVC

VSARSSGKVVVTGSWVHHSFDVGMIIDANSVLYRPLRDLNGIPGSKSLVVCLTGYQRQDR

EDIMTMVDLMGGQFSKPLVANRVTHLICYKFEGEKYVLAKRMKRIKLVNHRWLEDCLKNW

KLLPESDYEISGYELEMMEASARDSEDEAEGASVKRENTSPLGLRVGAVAAVETSKSRGK

DIPVVQTNSVEQGCNTSKENWLTPKKMEATVSTGAVAAQQSAGTFQNTSSYASPVPVTKT

SERGMGKMETDDSTPVNMSVRRHSSLATYSRKTLQRSAETSTLGNESSCQNGTLQIDDRG

IQASTLFEDLDKSDKLHGEESSTLLPQAKLTDGSVSSKDSEKVHHKDEASITPPAMSSGE

NIISKCAPDEIPGRSSTEPLVGNILLQEQRSGSPKQNLSVVPNINDNTEGKEVAHKLDVR

DSAIRLCNPSGVLMEADKWSAETISLMGGLDAVPERSVMMRSSTSPVETELAKKKTAPRK

SLGSKGRKKNTINQKGSIYLSEPTPKDEHTDGLIEGEVSEVATDNSHQKEKSSPVLNAEA

VPEMAKETEAVERIESDKLQAKTPEEADAEVEITMLESEVTDVPSKDLSDGALQLEVKKS

KNKRTREATVGKKSLQIGKKGSSSRKEVDAEANETVMEDVRDTSVNEMESFALENKLGKV

SSGGDQRPEAGEALVSAEAETKDPSDAEVELGVDKNDGKLRKEATNGKNKLSSGKKRRSS

TVELEKSSVKKAKSSKKDDEAEANDIVMNGTVINSAEVKENVAVDNKPDDVSSDGTQSPA

AKNPSNAAMQLEVRKNKRKTKKEATIQSEEKESSSKAEVGTSSVKKPKKSKKECDTKASD

SVMKDVVDNSAEEKENTEVDNKSGRDKSPVSGKPLARKKAAKSAKTDTKADKVSKQLSKK

VLKEQEQEPKHFIVSGPKVQRKEYQKIIRLLKGKCCRDSHQWSYQATHFIAPEIRRTEKF

FAAAASGSWILKTDYVADSKEAGKLLPEESYEWHSTGLSADGSISLESPRKWRLVKEQTG

HGALHGLRVVVYGDCSIPSLDTLKRAVKAGDGTILATAPPYTRFLNENTDFALISPGMPR

EDVWIQEFIRHEIPCVLADYIVEYVCKPGYALDKHVLYNTNTWAERSFKKLQLSVVED

**BASAL DICOTS**

>Atrichopoda_BCP1

MRDPQAINDGNSQLFSGVRFVLFGFDEAAQIQYQSELENGGAIDVSPYNSSCTHVIVHRL

TYNDPVCVEARHDGKTLVSALWVEDSLEFGKLADANQILYKPVRDLNGIRGCQSMHICLT

GYQKQERSDIMKMVDLMGAQFTKPLIASKVTHLVCYKFEGEKYNLAKKLGIKLINHRWLE

DCLKAWDILPEDRYRISGWELEMLEAEARDSEEETTKAVIPTTPFRARGNSGVLPLQSEV

LRSTNSDISMPQGARSIQKKTDAAEASSAVQIDIARNDMLFSTPCKEAPNRKDHVPCDGT

NKECNEMLSYQDMGAKLMADNDHVMEDIVVGGDTTTSIDEVQNHMVGANLVSSEKKTPKR

SHSSAINVTPNSMSYSRKISKKSTMAELLDELSSGLNSSSLKTFGENDSIKNDDNVVSTA

QPFKTSVHRVGEEVINVEKLPQWSTGKGEEAVLETGAVPFREGKALESQKHIFETSLGNG

HSAETVLDGLPLHQTRINVNPSVKEENKRLECNALVNCTENGQTGSTENQCATLRKKTLK

RRPLISKKGEHKLDLSLSKMAGIPTNGELKAVLDMQMDMSNGSGESIMKNLKPTEAVTAD

VKPKTSLNGFQTLEMPTKSENDNGLLNELASLSNHGISEESKSHQSGKSPESIKSAAIKL

QSSTIVEECDNGSFDKKKDGEHNNVEDKKLVPKKSMRCRAKLSTSDLSKGKSECPSGQSG

SQGEVTICAEKADETGLYTGKSLKDLNTEMEDLTVKPDDCMAVAQENEVMSPKSISRKNV

KKNAIVSETSMDAEKENKLIPTGEETSESAKPQYRNGRVFKKILKGKKTKVENSTMNLGT

QDKVAKLKTSLKKSVPENVILNDTYMARENENKRIPRGKQTAEMAKPQSEKAAVKSETKT

FQNIDMDNGFEAKGLGRVRQSAIMIQPAQRWFILSGHPLQKKEFQQVIKKVRGKLCKDSH

NWSYQATHFIVPDPVRRTEKFFAAAAAGRWILKTDYLTSSYQAGWFLEEEPFEWHKSGLS

EDGAINLEAPRKWRLLREKTGHGAFYGMRIVVYGECIAPTLDSLKRVVKAGDGVILATSP

PYTRLLKSGVDYAVVSPGMPHADAWVQEFMHHGIPCILADYLVEYVCKPGYPLDRHVLFK

MHDAAKKSYDNLMARGQEVVKGEEANGDDTICDVCHSGERANVMLLCGDENGAGCGVGRH

IDCCDPPFEAVPEEDWFCSERCKPLG

>Ltulipifera_BCP1

MFHGVRFVLIGFDSVSEAQYRSELLNGGGVDVGRYDSSCTHVIVHDRVYDDPVCVTARRD

GKILITDLWVEDSLDLQKLADDSRILYRPLRDLKGIPGSESLHICLTGYQRQARDDIMKM

VELMGAQFSKPLIASKVTHLICYKFEGEKYELARKIGIKLVNHQWLEDCLRAWQILPVDK

YNKSGWEVEIMEAEARDSEEETEPGAGVFSSSNNVMLIHSSELPTPVREAPKIQQNTSTA

GTSTHGSELRTPAREASKIQQNASTAGTSTHGSELRTPVREAPKIQQNASTVGMSTHGSV

LRTPVREAPKVQQNASAAKGPLNISEDILMNKRLVSSPGKDTSPDIAPDYSVNNRLVSAD

STSGKETSSSKGVDGIEGSGYHDAGLHTLAASGRGMLDGGIGEGISNLDDKVTKPTIEGN

TPFSDQRTFKRSPDSNLPVEKFDLCFSRKIPKRSAASLSPTPTTHSRSSPQAHLEENTVA

GALTFEQARNDPASREIQIPTVHTEKHHKEGTHNSFPQKRKLAVSRSGLGLLKARDHNVT

GSRQNTAASAEAEHAAMMVDEPHSKMNPHITNNRSPSCVENQHANPSKTKPAKSLKRGRT

VLATKISDNSGSSSDGMVKRDIKSSHMLDEQKIISDTTNYLGAMAINNKSAVETNQLEPM

AVDGRSLVETSNEEKAPETSSGGSKESASGTKSDVGMSKSSNLPVTKIGDPQKSPNSIKS

KTENFESTAYARTLAERDDIPSSTKLRTKMLAKKNVGYKHKPSTSNTSKGSSASYSDKTD

MHGEVKNGKDGVEPKKMNSDMNVEMANSTCKGSDVIMEENVVGIPCSENKPKNKTVTLDA

KEALVDAEKENKPTENGVKSVNSGKRGRQKAAVKKSKALSMQNTEKAVGTDVDGLQADRS

SRTGSSELSWFILSGHRLQRKEFQQVIRRLRGRLCRDSHNWSYQATHFIVPDPVRRTEKF

FAAAAAGRWILKTDYLTASSQAGRFLAEEPFEWYKSGLSEDGAINLEAPQKWRLLRERTG

HGAFYGMRIVIYGECITPPLDTLKRVVKAGDGTILATSPPYTRFLKSGVDFAVISPGMPR

VDCWVQEFMRHEIPCVAADYLVEYVCKPGYSLERHVLYKTHAWAEKSFANLSSRLEEIVE

ASTPSEDGDDISCEVCGSRDRGGVMLICGDERGTLGCGVGTHIDCCKPPLEAVPKEDWFC

SKCQSSSTNPMKGTNKGSSLLKCK

>Ncolorata_BCP1

MAESPTAFSDNANPPGSIFLGVRFVLCGFDQPDEVQFRDALVDGGAVDVGRYDSTCTHVI

VHRLTRDDPVCVVAWRDGKIVVTETWVGDSLDLGRPADANRVLYMPVKDLDGIPKFREIH

ICLTGYQKQERYDIMRMVELVGAKFSKPLVATKVTHLICYKFEGEKYVLAKKIGIKLVNH

RWLEDSLRAWDKCPEDEYSKSSWELEIVDAEAGDSEEESENMRYGRPSNQRKIGGDGSYM

NKWHGDVMTAGKAYEMQYNNGTVQVSSSTPNKMLANAPLTRKSTPDKGAVDGRISDEASK

MVEIQYDNEAIQASLSTPNKVLENAALLPIQRKTTPDKGNGGRPEGILGTENFGGSLVKA

NGHVVEHAFERTPIAEHGERAAGYYDISKVISGINISNQTPYSDSSDKRTPPSCSSRTSK

LSCASYLSKDPSNRLSIPHQNSHGERSISPPASKQIADDFDAFEVTGINGQVSGKTDTLT

VNQVHVMADNGVALARAEEKPQNKSTELKSVGSEDGSSCSPAVDAHNFLKNNLESSVCNP

VKTHSVETAQDRIADPPPKKLLSFRRNSTKRLTAFSTSPADTLQAKVSELHVKDQTAFSG

KVDGANMPPIEGYVTQGGGSLEMNSNQGGTKSLLFIDSGSISDQANNNAHMSTSLIPLNG

QNNESKETNCSRVELLSSDHNFSPEKNQTGESAEACENEMNGSSSKNPSIKITKKFVARK

NTSKRHGINDMTPFDAKCGLSSGEEPITGNLTNGAVQSQADAQHEPLEGKKNFEIGLTLT

AGKVLQIEQGNNRPSAQRPSNKTKRKSAVMIENDVVIDDEEKHSTKYKQYNAKNQVCDGE

KNVSKFEHANEPNVFALRSAEGRENIEKFLGPTANNMQHIGRKNSRLEVPKSSSNLKRKS

RVVSETNLAADKENQPNETESQNIDNVKKHSTGKIVSKSTQVKSKRGGVNSEGKIDASKV

GKMSTSKQFEPQWFILSGHHLQRKEFQQVIKNLRGRICRDSHHWCYQATHLIVPDPLRRT

EKFFAAAAAGRWILKTDYLTASSQAGKFLNEEPFEWYKTGLSEDGAINLESPRKWRLLRQ

RTGHGAFYGMNIIIYGECIAPSLDTLKRVVKAGDGSILATSPPYHRFLKSHVDFAIVSPG

MPRVDAWIQEFLAHNVPCVLADYLVEFVCKPGFSLDRHVLYNTYAAAEKSHANHLQRLEV

IEVPTQEDDIVCSVCKLNDRAEVMLICGDDAGKVGCGVAMHIDCCDPPLDSVPEDDWFCS

KCCSSVSMKGAKNSSVLKRKSSLHVK

>Ckanehirae_BCP1

MFEIRSKDGSDFNQIQLQKMFKGIRFVLIGFDSVSEAQYRLELVNGGGVDVGVYDESCTH

VIVHGRVYDDPVCIAARRDGKTLVTEFWVEDSLDIGNVADARRILYRPVKDLKGIPGAES

LHICLTGYQRQDRDGIMRMVELMGAQFSKPLVASKVTHLICYKFEGEKYELAKKIGKNLV

NHRWLEDCLKTWEVLPVDNYSKSSWELELMEAEARDSEEETEPDVEGLSNLHGGSGTLIP

SSELPTPVREVPKIQQDKTADNGSPLISEDISMRSNKLLSTPSKETNPEKASELGDFDDK

GLAEYRYMSAGSHAREDNGNAASSSRIPQNASFPGCRSVELNKEETTSSSSLRTTKRSPY

SEASIEKSSDLSYSRKIPRKSESLLTELSNHQGNKIPRKSMSLLTELSNHQGNSPQVLGE

KAAVDVCNSSGLTSRQAESDLNAAEIQIQTASAVKHHDEEQHDFLPQKRKATTLNTALSL

PNKGDSAITTHGSSEVDPTSMIDAPSSNANPSLNNNDLVDMAITVNQLVNQPVEPSKTKS

ASMMKKSGKCMRPVSRIKESKKNCSLLNSNVTGLPCDVEPSYTLDKQTTKSDAAPYEGTS

AVETDMNQDAAAISGRSAVESFMKEHVPELPLGIKAKHEDVNLSRSPNCLVSESGVHQKM

AQHEEVSFCNKKVVDVEKSQNAVHSSSLTGRNPMGKVSKKLVAKRNKTFSAKLSNTSKGY

GAAKVDKTITPHELEESEDGAELVKAVSDDNAEMPIELHTKASDVAMEGGTHSVKETKVE

SKAMNKEVFMDAEKEKETNIAGFNYAVSDLVTEEGTHRSMNRAESKNSGMNRKEALLDVE

TEKRPVMDKSSSIASDAFIKERVSFVTKMCEASAVDEIDTSMGLDKVKILSGAESASNTS

GVVTKKLKAMRRPVNKTMSKISTVDNKEKFMNADKENRPSESPSVNSNKQGNQKDPVQSI

KKSTRNEVVDGADAGGLHASGVSSVVSEPAWFILSGHRLQRKEFQQVIRRLRGRLCRDSH

QWSYQATHFIAPDPVRRTEKFFSAAAAGRWILKTEYLTASNQAERFLAEEPFEWSKKGLS

EDGAISLEAPRKWRLLRERTGHGAFYGMRILIYGECLAPSLDTLKRVVKAGDGTILATSP

PYNRFLKSGVDFAIIGQGVLRVDCWVQEFLRHEIPCIVADYLVDYVCKPGSSLERHVLYK

THAWAEKSFGNLLSRSEEVVQNLTTAEQNEDIACEVCGSHERGDVMLICGDEGGSVGCGK

GTHIDCCDPPLEAVPEGDWFCSKCRSSMTASTKRQKKASSSKKCK

**NON-FLOWERING LAND PLANTS**

>Mpolymorpha_BCP1

MKKKFWGAELKEVVSELEKEYDQCHTCWPTTIFSQGSLTERKFFCCSKVVRADSDTKMTS

TEEDSGVFSGVRFLLAGFEPSYEKQYIEELEENGGENAVDYQSNPTHVIVSNLAFDDPIC

AAARKDGIALVTDSWVPDCLDNGALVDVNSVLYRPLKDLDGIPGSEDLCICLTGYQGQQR

QKIMRMVELIGARFTKPLLGNLVTHLICYKFEGEKYDLAKKTGLKLINHYWLEDCLKAWS

LLPEADYSRSGWEVETDAKQQKIIVEKDEVVPTLVPQKINQQRATDEVLSPDRVELSTNR

RSSGILDSSSAKSSSMNGTPCRNSPTPKPSSSPDKVGEQERQDIAQAEATVKQAAELQVS

ASDMTETMDGIERSNALEDPDIFHEALMIADGRGQTSPVHAKKTNTSHSRRRSSGGVANP

VSPASPGICEAAVAADQSASIDCAIERMSISPDLPAKRRLSGIQGPEPSAETSRNAVDLE

SPENKRRKGSRLSRMGLSPPIKNDCSTKVQESPKSALVTKQIASPSVTPLPDKRKANHER

SSNEEETQNGATSNSTSPEGINREDDVDMEVAEPSRHSTPGNKRINGGSKLRAVDARKVR

DSSDGLLSPPKSTTEDPVEHEVDDAFKHADTTLSAVKTGGIQPSSPTTSSAVEKEVDMED

VDRSPRSTRNSKTAGLKKKSNIPKVRKSADKSPGFIPSGSTTGETVNNSANGTTHADAVQ

NEGSRRTMEPSPPKSSSAAESKDDMDLDRSPCPTLKTKKVSGQKKGPDSKIRKSVDRSHG

VVPLEEQVEVSLASSSPNEGTLVKDYRARKSSKKAPQETGLLTASSAMAGDSGGLVEHNV

DTGAIEHEITLEATAVKQSAKKQKGSSKKVLKAASFVLPDEGNQESPKAVQQGAALETAS

AEEATTSAMKIPLSRPKKHQKSSPKSIAAISAGDKGEGIANDAPLKLSSREKSTKIPALG

SVSKQDARRGKKPKEKCKMPSNEQEKENIELQEGNHELVLKEVAAKESSKKGARISLLGG

ALPGTKKHFAVGGSTDERKNLHYIIKLLGAKVCTNRYQWKDDITHVVLPVPLRRTEKFLA

GAAAGRWILQPEYVEASRKAGMFVDEQKYEWFGEGMNEKGTISLQAPRKWRIQFARTGCG

AFYGLRVVVYGGCISPPLETLKRAIRAGGGAVLATSPPYSQHLMDGVDFAIINPECNDQD

EHVQEFLSHEVACVTSDYIVDYICHPASSRANHVLYDTHLAVSSAEQRLESNIARGPTEA

DKSTKPTSKVPSRLSKEVRVTDDFACVVCGRTDDEKVMLLCGDGKGNGCGLATHIHCCSP

PLFEVPEEDWFCRNCEDL

>Smoellendorffii_BCP1

MVGSCGGNYAATATLFRGVRFFLSGFDRAEECQDDPICAAARKDGKVLVTKSWVLDSLEQ

CELKNANSVLYMPVRDLAGIPGAKKVCVCLTGYQSASRREIEAMVAMMGASFTKPLDARK

VTHLVCYKFEGDKYELAEHMKLKLVNHRWLEDCLRTWSLVDEANYRISAHDLEIAEAQAK

DAQVSEQPNETNAAPPEIAPDPPDVDSEATTQLCEAEDDCVVEGENVTSKFMGSPMDTGN

DSATKQRVMDTVNDSAAKLAEQRVMDTGNDRATKSAEHRVMPSNARNESLSGGESESPSL

KKRKSFEASSDHKKTGEDSIRKRGRPLSSLSPRPRHSDRTMDGQSPDAVKQSPSKSSNGS

GKRIYIEPERSSFHSKSGSAAKKSGKHNAAGMGMDNKKEGSFKSRRLTRSGSKDSITDAN

GHETRRKSLSGSAEKLTSKDDAVESKHQPANEPKAQKQTDVINLDDEEEKKDEAAKEAAE

VPVKRGRGRPKKSKPIIPETTKDYRFALSGHTEEKRKHRLVIRSLHGHLCRDDHIWKEQT

THCVMGSPLRRTEKLFAALASGRWILKFDYLEACAKARRFVDEEPYEWFEPGDAKDGTID

LRAPRKWRLSKQETGCGAFEGLRVIVYGECILPSLGTLKRAIKAGGGAVVAVEPPYTIPL

ATGVDFAIVGLGIPENDPRVVEILKRKVACVRDEFFVEFVCNPSASWEKHVLFSTEAAVE

KALAKLATIKSYSSHKQSGGGEEDDGGGGAAAAVVAEEEMDRACNVCGRKDSEEVMLFCD

GDDCEVATHTFCLEPPLDKVPAGDWYCARCSS

>Dcomplanatum_BCP1

MEAGNSFEPSSSTLFLGMQFFLAGFDPATEKQVVAELERNGGRKVGSYDEGCTHVIVSQL

PYEDPICKIAREHGRKLLSDYWIVDCLDYGMIMDVKNVIYKPLQDLNGIHGSEALCICLT

GYQGQARREIMKMVEMIGAKFTKPLIASKVTHLICYKFEGDKYNLAKRLGIKLVNQCWLE

DCLKTWTLLPEENYAISGWELELANEEAKDSEEGEDTKTGVGEATPDQRSPFPIGAKSKL

SGHMDNVQNFSVSHKNPVAHTSVQLIENHILMEAAEGILIQDFSPKSMQFTSPENSCKDK

IKQRYDTHDELELEAQKEWRTPSEKKMHVSIFDDMKVEPLTEDVLKKQNRISKPSKDSPR

ANLDASGSVKKSASERTCCTAEKTKIKSHKRRSLLSEKTLMEGDNCITKVTLSVQDREEE

PYHDSKTPKSSRRSKSAGRSDSHHAYDRSSLGSKEIEVNNDVEVSKSESNHVHGLLDGTR

NSVPAYDAQSPAQEKEVEQLEVLNTVTRLSHACNAENTNEQVFETSLAGLDGAGTDNANK

ENVPLSAKVHLSSPKSKIQEGLLHTSPSKKCKSSAVKEVNVLGIVRSSKSKGKQTGKKVD

LNKENSERKTAAPVSVNISQSPTNMEVRRLDFEATNLEGDSLETVCIARELLELQNAKEL

NSSDGQTCEKSSSNPGNSEFRKPPESTDGQETLDHCSRKRHKKGQPLMHSISNKESGLLS

AAKVSPAASMIVACAPKDILKQQEDKNLGQETIDNCPRKRHKKGQPLMDSVSNNESGLLS

AAKVSPAASVIVACAPKDELEQQEDKNVEKHKISKSIEPIDLTHTRQKKGKNGSRKKTAN

TPSHEKGKLISAVQLQKHEVSSQEAQKRCFALSGHRNEKEKFLKMIKELGGHACRNDHRW

NNRVTHVVFASPLRRTEKFFAAAAAGRWILTGKYLEASRQAGRFVSEEAYEWYGAGDNDE

GTISLGAPRKWRLWKEKTGCGAFYGMRVLIYGECISPPLDTLKHAIKAGGGTILASSPPY

TRHLVSGVDYAIISFGIPREDQWVSEFLKHKVACVASDYLVEFICKPFSPLGRHVLYGSE

AAVEIAQKRLTDAVNSSVKQDTHDCKANLKKQNQKTKSQKRIREQATGSISDPLVDIACF

VCAQTDREDVMLLCGDDQGRGCGVAIHIDCCQPSLQSVPEEDWFCIACSRIEAA

>Cricardii_BCP1

MFGTPSDTRFQETPPLFAGVFFTLAGFDPRLESQLVEALEQHGAVNVGEYGASCTHVIVP

RSFLWDDPVCAAARDDGKHLCSEFWVSDSLEQGVIADVDHVVYRPLRDTQGIPGTKKLIV

CLTGYQGPARRNVMRMVQMMGAHFSKPLVANRITHLVCYKFEGEKYNLAKQLGLKLVNHQ

WLENCLKTWSLLPEDDYARSGLEVELAEAEAKDSEDDGNLKPAVLKDVSNEVLKGGLGGD

RRFESEVKTLGIEVNSNDNMKISSNILSPQNVGSTIEISGVISKVDATSVLQIEGMDGNG

ALPQIEGMDGSGALPQIEAIGYLDITSSKQQKVCVGVDIDDTSVRHENEILDGDKNAETC

FLNGHDDVSCVDRNIASFVTPLKGNGDEVEPDVAFRASTRNDGDVSVPEGTPLSMLFSDF

EKTPTDRAFHTDSTSRMKLRQSMRAVSRVSDKKKPGRQSRKTELAGTPGEEILPAGFAAD

VSSVNDKRSTGILKVSSNLKKHHTKDIANENTSDREEGNEICMVNTMSNCDVQQVGQEPV

IAGNLVIDGNELDNGDRDVRDIVASPSCIADNIDRCDSVKTDVRMSRARQQGNLSIGSKV

LRAQRKRVLTEETLVEEKISSPVHNSGPVNDVKQKGPSIREVEERQLTNEADCKKKLNGR

KTASVRKPKLSNAARKNNEMSVTSELVTNVLGLEDSAMLAAAHKDTKHDSLLSQVEGGHV

VSDVDPALKYNGRESRLIRKEEKKSSSHAKDNEMTVRSELTSKALGRRKNTKAAAALINQ

GNESNIDPSSRASEAHMNQENVSKMDASETPNLPSVSVHININSEKECQPSVMDVEDSSN

RGGKQSKTSRTINTTNKRVSTSKRKAAGKENVSEKNYGLLYENKIEDQSSTGANQKEVKG

HQSSDQKQQKITPQSFFAFSGRKIEVKPLQAIVKKLGGKICRESHQWSHQTTHLIIVGRL

RRTEKLFAAAAAGRWILRDDYVRDSGEAGVLLDEMKYEWYGSGMTEDGAISFEAPRKWRE

HRERTNCCALEGLRMLLYGECIIPSLDTLRRALRAGGAELVATCPPYTKMLASGVDFAIV

NPGTPKNDPWVREFLSHNIACVTTDFFVEFICKPSSSLERHTLYDTHRFVRQALDHHVAN

GERSKTEQKSIEDREVEDEGICCVVCGQSDREDVMLLCGDDKGSGCGTAMHTDCCNPPLK

EVPPHDWYCTHCTKSTPASKRTKRRKGAA

>Aangustus_BCP1

MASSQAGQDGADGKLFAGVRFVLAGFDEPSELEFLTELERNGGQNMGRYSAGCTHVIVCD

LTLDEPICVAALKDGKELVTEYWVADCLQFGTLMDTDTVLYRPLKDLNGIPGSEKLVLCL

TGYQGQMRTDIMRMVDMMGARFTKPLLAAEVTHLICYKFEGEKFQLANRLNIKLVNHRWL

EDCLLAWKVLPEIGYQRSGFELEFGNVVANDSEENGTPESTFRTASKTPRGGPGSNDATT

TTPKNPDKQGTREKTPRSCPSTKSRRMVSPGSRIQMLQSSRKGKQLNFQSSHTPMRGLSV

AEHQTTPTAHSTPMQPSSSKGQALVSGIGTLETCGVHHDENKTPQMHGFEVREQASGAVD

GAAEPRDVGAGLDINGNSKTDGSEKVLLQVNERSLSSFNAQTVSTACTAIKVDEADCQNG

ASEQRPDHGITSNRDALGKVIVNNHIQELKDGNGDRCSREVENTSLQGAVRSPGTGKCEM

GADCGQQGRTVQTATSTRDSPIAPLAASERLEVNAGVGPASLEKSVVLKAEIRTYKAQAV

EEIRKEPGELDELRQKSSADGLIHSESNTQMIEETALAVLKSFGKSGISKAGDSPDDMEI

LQESRQLNQIIASTSMLSAGKRKLSDSLPVSNAEDLSRDQLVAQETKTAVRRDLTSELQA

DLQGDENTPSPWRTKKAKLDPVFASPVPGVLEPNSTLDSGKLQKSSAKPKYAGLIRKLLG

SESVKRRSDFYDLEKEFCDDIGQGVTTPETNRAHRLSASGVQSVNNEEVPQEALPGQGPA

KQPPIPGNSGLQSALKKKADQVTVTVTPLKRPFGSLAKTQSNKASPAGVTKTPKLVGVQK

TRDTKTVKRPVLQNNGHGDPETKADNLELPKFPQLLPGVRNDVGDKTTHQGTVGVLAGED

SQQSSGPNEWTCLFAGNPPSNPLVKDADIPAVNDQRMGKINQCGLGENVPQGGPGASMAE

ERLQTIDRLGEAQIETTTTPKVKLRQTKQKATTRRSKGVEAVQLGLEASMPAAICETGRS

SRQPRLNKEPVKDVPEAVIEDHHSEEGLQSSMDRLGEASIETIVTPKVKVRQKKQKATTR

RSKEVEAVQLGLEAPMPAAVCETGRSSRQPCMNKVSVKDVPEAVIEDDHSVPASEEKVAE

PKCSMKDTERWFALAGRTKEKDRFKSILKKLGGKLSKDGHKWNDQSTHVILPAPLRRTEK

FFAGAAAGRWLLTADYLEASMEAKMFVKELDYEWHGAGYNGEGNIGLDAPRKWRKHREET

GCGAFHGLRIVVYGECTAPTVDTLKRAIKAGGGRLLAIAPPYGPDLSGVDFALLNRSISA

EDKWVKAFVDSGIACVAVDYLIDYVCKPSASLEKHILHGTSRAVADARKLLDSNIAAAAK

RFQACQVPGSGTREQEVKEQELKDPPSEVHQGEEVSEEGDDVFCSVCKKGDREDVMMLCG

DDKGRGCGVAMHIYCCKPPLSEVPEEDWFCKKCLKPPRKPRRVNK

>Aagrestis_BCP1

MASRQAGQDGADGKLFSGVRFVLAGFDEPSELEFLTELERNGGQNMGRYSASCTHVIVCD

LALDEPICVAALKDGKELVTEYWVADCLQFGTLMDTDTVLYRPLKDLNGIPGSEKLVLCL

TGYQGQMRTDIMRMVDMMGARFTKPLLAAEVTHLICYKFEGGKFDLANKLNIKLVNHRWL

EDCLLAWKVLPETGYQKSGFELEFGNVVANDSEENGTPESTFRTASKTPGPNDATTTTPK

TNKQGAREKTPRSCPSTKSRRMVSPGSRIQMLQSSRKGKQLNFQSLHTPMREQSVAEHQT

TPTAHSTPMQPSSSKGQALVSGIVTGETCSVRHDENKTPQVHRFEVCEQASGAMDGAQEP

RDGGAVLGMNGGSKTDEAEKVLLQVNETRLSSFNAQTVSTVSTAIKMDEADCQNEAAEQR

PDRGKASNCDVTGKVVVNNHIQELKEGNGDRCSRVVENTSLQGALRSPGTGKCEMGADGD

QQGSRTVQTATSTRDTPSAPFAGSELLEVNVGVGPTSAEKSDVLKAEMRTFKAQAVGETR

KEPGEDELRQKSPAAGLIHSESNTQMIEETALAVLKSFGKSGISKGGDSPDDMEILQESR

QLNQIIASTSMLSAGKRKLSDTSLPVSNADDLSRDQLAAQETKNPVRRDLTSEPQADLQG

DENTPSPWRTKKAKLDPVFASPVPGVLEANSVLDSSKLQKSSAKPKYAGLIRKLLGSESV

KRPTDFYDLGKEFCDDFGQGVTTPATKRAHRSLASGVQSVNKEEVPQEALPGQGHAKQPP

IPGKAGLQSALKKKADQATATVTPLKRPFDSLAKTQSNRASPAGVTKTPKLVGVQKKRDT

KTVNRPVLQTNAHGDPETKADSSELPKFPQLLAGVQSDVEDKTTHQTVEVLAGEDSQLSS

GPNEWTHLFAGNPPSNPLVKDADIPAAKDQDMSKINQCGLVENVTQGGREASKERLQSSI

DRLGEAQIETTVTLKDKVRQMKQKATTRRSKGAEAVQLGLEAPMPAAVCETGRGSRQSRM

NKEPVKDVPEVVIEDDDSEERLQSSIDRLGEAQVATTITPKVKVRQKKQKVMTRKSKEAE

AEQLGIDAPMPAAVCETGKSSRQPRMNKEPVKGVPEAVIEDDHSEERLQSSIDRLGEAQI

ETTVTPKVKARQKKEKATTRRSKKVEAVQLGLEAPMPAAVCEIGRSTRQPRTNKESVKDV

PEAVIEDDHSEEKLQSSIDRLGEAQVVTTVTPKVKVRQKNQKATTRRSKGVKAVQLGLEA

SMPAAVCETGRSSRQPRMNKDSVKDVPEAVIEDDHSVPAPEENVAEPKCSKKDTERWFAL

AGRTKEKDRFKSILKKLGGKLSKDGHKWHDRSTHVILPALCRTEKFFAGAAAGRWLLKAD

YLEASMEAKTFVEELDYEWHGAGYNGEGNIGLDAPRKWRKHREETGCGAFHSLRIVVYGE

CTAPTVDTLKRAIEAGGGRLLATAPPYGPDLSGIDFALLNRSISAEDKWVKAFVSGIACVAVDYLIDYVCKPSASLEKHILHGTSRAVADARKLLESNIAAAAKRYQAQVPGSGTREQEVKEQELKHPPSEVHQGEEVSEEGDDIFCSVCKKGDREDVMMLCGDDKGRGCGVAMHIYCCKPPLSEVPEEDWFCNKCLKPPRKTRRVNK

>Scucullata_BCP1

MASPLDDSPNDDLPAFFSGFRFFIHGFGTHIHDQLCDAIELRGGVTSDKNDTSCTHVVVS

QQLPLDDAVCAAARRDGKILVTEFWVSDCLDAGEIVDTNYIVYRPVPDPEGIPGSKKLIV

CLTGYQGQARRNIMRMVHLMGAHFCKPLVANKVTHLVCYKFEGEKFNLAKHLKIKLINHK

WLEDSLKSWSILAEDDYLKSGLELELLEAEAKDSEDDERIQIKASTQELSSEMVAEKQEF

VSTDLRGANHGHDSLETPIVPCANVLRVDLIDQTDFHAESMHIDSKDDAHLHDELHSITK

LEDDSLPRIMEGQIKNTTGKAADDRKSSSLCLTPVECPSAKKYVRRTSNLERNYGPLTAC

QPFEDASSLQLNDSECCQPCRDNLQKDTDVQGSSCFGTTTKNELVHHIQVEKEDKDTVSV

QHEIASFLKKVQIVEQSDFKRNGAGFLIPNDKTRSKNSRESMVSTVEAAIDNRHEECTES

ASSRTGNAISKKVTPVNSEINDGHVLHYELLENNDDLPNGSESVTPGEENELTGNRKRKS

WQHDTLEGAGSENNLEYSEKVLPFPGFKSKRHSDTDSVSKINKDMKIDNSSAPLVKESNS

DDRKKESLQRKCGKEIESSTPQRKAVDMGSNFKDSAYKTLDLIAVTMPLTKSHCSPTQFL

QTVAAKENSHHAIMSDIQNQSEDPFHEKDKLLDLKPSGEIVRYNNENVTIPRSCRKPGDN

QKGDKALTERKDATKSVRNGVPKKSKQQAKVESNLNDQKSDESVRKLQLKKGSRKVNAEP

IKRVIKKHMSALIDKENAVVGDAHPSKTRDDAKDTNKTLVCYNRNLSTHKCFALSGRRQQ

TKPLQALIKKLGGKCCKDSHQWSYQTTHLVIAGQLRRTEKFFAAAAAGRWILREDYLKDS

ADAGVFLDEKKYEWCGSGMTEDGSISFEAPRKWREQRENSGCGALEGLRILLYGECIIPS

LDTLKRAFKAGGADLLATSPPYTRFLVSGVDVAIVSPGTPRSDPWVQEFLNHHIACVTTD

FFVDLVCKPTSSPDKHVLYDSQNAIAEVMARLKANQTAVKKTGGAEDPGVEEIRCSACGL

SDRDDVMLLCGDGGNGCGIAMHIDCCKPPLAEVPANDWYCMQCTKSPMIEKKNEGTKKKR

RS

>Afiliculoides_BCP1

MADDDSSLEDCSDIFLRLRFFLSGFDSDTYDQLSVALEQNGGVNMEEFDASCTHVVVSKS

VLWDDPICEIARKNGKFLVTEIWVSDCLELGVIADVNHVVYRPLRDSKGIPGSDDLVVCL

TGYQNQARTNVMRMVYLMGAKFSKPLVAINVTHLVCYKFEGNNIIRGPKFILAKQIGLKL

VNHKWLEDCLKSWSILSEEDYMNSGVEAELLEAEVNDFEDNGSSRVKATSEHRISDIAAG

VCESGKQEFVSTNRVAVNGTLNSSGVEAFKLTPARCAEAPVQNLISDPQTVKYEVDTTVA

ENILIDDESGNFHRGDPARKHPISRTEETLGKICLDHVECTSGDMKTHEKPCGPARCVEA

PVQNLISDPQTVKYEVDTTVAENILIDDESGNFHRGDPARKHPISRTEETLGKICLDHVE

CTSGDMKTHEKPCGPARCVEAPVQNLISDPQTVKYEVDTTVAENILIDDESGNFHRGDPA

RKHPISRTEETLGKICLDHVECTSGDMKTHEKPCGPARCAEAPVQNLISDPQTVKYEVDT

TVAENILIDDESGNFHRGDPARKHPISRTEETLGKICLDHAECTSGDMKTHEKPCGESTN

SEQIFSTRKSEAVSSPKTNRIKTYSSPVTTNNAHSKENNKSESSPAKVVSVVGVNNSKMA

SSCSDDLLANEHTHTNEFKISDVNIVKNIDVQATPCCNINLSKEISGHMQIEENYETSSY

PSFLHKHEPLNKSNVVANAISVGQSQLKGTKKRKSLTDSSLETVVGYHLECIENTSSRLI

DTNIAIPSDIHVVLPGHTNVMTNGVGCVTPVDRSEMKGNRKRKSRETSIVKDLDDTSHTE

CMISTPFSVAISKSTGHSSGSIKSSSLEKLVSFDVTFPHSMSKTAKINENVLDAVIKDCE

KEDENEVHADYKKYNGKKHDRDMLVERAENSTKVSKKQATVSANRLQHAELNSHEREELE

KKGKVISNRRKKTPLNDNQDDCIIQKKPVKSSKAVAPKRRDAKNYMAIDATFRDKENVVT

SQGRETKSLSKISKTGSVLKDSTKVLTFPSKCMLPNSAKQTCFALSGRKQEIKPFHAIVK

RLGGKLCRESHQWSHQTTHLITVGQLRRTEKFFAAAAAGRWILQDDYLKDSADAGVFLDE

KKYEWYGSGMTEDGSICLEAPRKWREQRENSGCCALEGLRILLYGECIIPSLDTLKRAIR

AGGAELLATSPPYTRFLGSGVHVAIVSPGTPKADFWVQEFLNHNLACVTTDFFVSFVCKP

NLSLDQHVLYDSFSAVEKVVAQLKSNERAEKKWIDADACDVEEIRCSVCGSSDREDVMLL

CGDDIGNGCGIAMHTDCCKPPLSEVPVNDWFCVQCTKPALLQPKKKTKRKVV

>Mvestita_BCP1

Top of Form

Bottom of Form

Top of Form

Bottom of Form

MASSFDSPSDDGPPLFSGMYFHLSGFDAQTEDQLKGALEQHGGAYVDLEDPSCTHVIVSTLLLDDPVCVAAKKEGKFLVTEIWVSDCLNLGIIEDVTHVIYRPLPEIEGIAGSKKFVVCLTGYQGQARRNVMRMVQLMGANFSKPLIANKVTHLICYKFEGEKYNLAQQLGLKLVNHRWLEDCLKEWSIMPEENYKMSGYELEVLEAEAKDSEDDDTKDVLCSEPDRIGIANNQDQDHATRTPQSKEPGVPMAQETEEATENPEKISAGIEDVQGVSKNQMQNEESEAIKTPSELKKLSPQTSSLSRGRAQSNKPSRFYEESKNSKNPKLPVESDTILTASTTHSGDKSASPDGCDNAGEESASERAEVNAGGDHLQTTESPAQKEPGFITPVSQGQAKSKRKREYKLSIKKTVENNGTGSSILESTGTNSDVGDSTADHLQPGAYNESDHEKLEEAHSEEKQASRKKSTVNSRSRGKVKQTDKKVDMIDGSVQTLETKEKEDCLPSEPKVITPSSRNSAVIKAIDKQSSGKKNVGNRSRGKGKLNQSDENELKTIDSSILSLEKNAVIQGSVSPRDNSEEAKSKQKAASGKKSSSYIKSRGKDGNKSTSTDSKSSDDMKDGHPNTVVPCTVEIQQQENQSSIPGATDSSLDSGMAPPQILQEASATVATNSGSKKSKEIKGQGSKKESLANCQTNIEVSANAKKQTNVSKEPERRNLLEELNKNESVAQDTDHHEKLDAEPVVKKSKLKNVKRPTVLPKKPVSKKKEKPKAVLSGAALSDKENVDDDQSIVPKNKRKTADDVFAISKSYQTSSMNNISKPSNQRCFALSGHRHETKPLQVVVKKLGGKLCRDSHQWSHQTTHLIIAGQHLRRTEKFFAAAAAGRWILRADYLTESDDAGKFLDEVQYEWHGSGMTEEGSISYEAPRKWRELREKTGCCALEGLRIILYGECIIPSLDTMRRAIRAGGAELLATSPPYTRFVSSGVDIAIVSPGTPRSDEWIQEFLTNNIACVTTDFIVDFVCKPSSSLDRHILYDTHEAVEKMLDRLKENQLGLKPVSKSLNVNDQLVSKGEEISCAVCGQSDRGDVMLLCGDGEGSGCGCAMHIDCCEPPLDEVPSCDWYCMECTKPQGLQKNKKKKTISQKA

>Acapillus_BCP1

Top of Form

Bottom of Form

Top of Form

Bottom of Form

MLGVVTNSGSQDSPPPPLFAGVFFLLSGFDPSTQSQLEEALEQHGGVNVGEYNGSCTHVIVPRTFLWDDPVCAATRKDGKCLCSEIWVSDCLEHGVIADVNHVVYRPLRDSQGIPGTKNLVVCLTGYQGSARRNVMRMVYMMGAQFSKPLVAHRITHLVCYKFEGEKFSLAKQLGLKLVNHQWLEDCLRTWALLSEEDYKKSGFEVELSEVEAKDSEDDGNLKSSLLKSEVPKGPAIPCSGGEREEVKFQAEINKFSVEHSAKDKQKEEHIFSDFSSPQKQGDTVKKSNVTSIMGEQNVV
IENEFVSEMQEEGRHANLTSPQIKGTPDVLRSSKKQTIVVNVDDSCSLLEQGVRVCAGGDSPGTILLEGCDLGSSCKGKNVSKLATPNVGNSNSVEFKLAFNSSSINKDEKDKNTLIPQETPCKVSCEVEKMTLDVPVGGSKTTKRKSRLSAKEDNKVSGTKKQGKMSKKSDLTSTTEQKIHTAEEVVSDKKVTSHPEVASSPKSQNVEGSTNNNGYLFMRGGDTHGADESVCILQMEGDNPGGSLSKADKVLKVITPTKPDENAANLEPVLGRPGLNKGGVVTTPTAEVIKCDMAAIGG
KNLPNKAVPVITSMSKGKRNQTAKSDVEVSGTKKQGKQSKNLEIVSTTKKRISTAEKMVLDKENVACQSHDCKSEGKVSQEAAVGRKPHEMTGSQDKMSNVAKVTSQRCFALSGRKDDIKPLQALVKKLGGKLCRESHQWSHQTTHLVVAGQLRRTEKLFAAAAAGRWIVRDDYLRDSCEVGSFLDEKKYEWHGVGMTKDGAISFEAPRKWREQRETAGCCALEGLRILLYGECIIPSLDTLRRAIRAGGADLVATCPPYTKFLASKVDFAIVNPGTPKNDQWIQEFLSHDIACVTTDFF
VDFVCKPSSSLDRHILYDTHSLVEAVVTRLQASNAGAKRGRQESIDKGEEGAGGEDICCAVCGRSDREDVMLLCGDDKGSGCGTAMHIDCCKPPLEKVPANDCFNEFTIGLLLPESTPSFKVGPPQALSKEKSEGRSSPLKEEQLEEDEGGTIARMAYFNDFFSFLNEI

>Aspinulosa_BCP1

Top of Form

Bottom of Form

Top of Form

Bottom of Form

MSLAGSGGRRWRGWIGPGWTKVARLDRRGLDEDGEVGPARVGRRWRGWAGTGWTKVARLDRDEDGEVGPARVGRRWRGEKFNLAKQLGLKLVNHLWLEDCLRTWALLSEEDYLKSGLEMELLEAEAKDSEDDGNTEVMLTGHKVSSGTVATCPEAAKQEVISHMQVEKLDVGSLPEHVLLDGKKDGVHSPLKSILSKKHRNNAEKDSTFVSPKKEAPIRAVEDQQEWSTEDAVVCEMQVEKGDADLSPAQFSKTRKMKALDPHEMASSSKRLKSSVSADENGALSREANCQRRQDSEISF
ANEAVCEMQVESAEVGSPCKADMIHKFTSPAKEHVNTSKMEFNSISPSLKKDIKGQASPNPQRSRCNEIAYEMQVERTCEETTPAQLPSGVTPSSVKGNELQEHSNVLVKDFGSVTPVHTDFLDNNEKREGRQDNNKGTADEITCLESTEKGSSSPHISKAKRLLTIHSPSNCTSSREHEGGLQLGCSSVIANKKYKEVKSQQKQDCQKNSARSNRLKMQSKITGMDSNPGHFSPGKIVAVNSPAMGNLSESNSEKGLNNLTERSHPRKETDKQNTEGSERGIEDVKASQKQAGETGLLN
EQECNVEVHHRRKLDQPLKKQGKVKGSKKQGAISKKLALQDQEKTSHLAAESMVLDKENSVMSQGHDPKVVSNTSDDAVVVRNSSEMTMCPKVMLPKTSSNRCFALSGHRQETRPLQALVKKLGGKLCRDSHQWSHQTTHLVMAGELRRTEKLFAAASAGRWILREDYLKDSSDAGVFLDEKKYEWHESGMTKDGSISFEAPRKWRELRENTGCCALEGLRILLYGECIIPSLDTLRRAIRAGGAELVATSPPYTRFLASRIDFAIVNPGTPKSDHWIQEFLNHSLACVTTDFFVDFVCK
PSSSLDRHVLYDTHHAVQKTVARLKASEVGTEGDRKASNDDGEGCGGEEICCSVCGLSDREDVMLLCGDDKGSGCGTAMHIDCCKPPLDEVPVQDWYCKQCTRPPLLQKNRKKKKASAKG

>Itaiwanensis_BCP1

MEKTPRPGHGDGLGISNSYEDADSALFSGVRFMLAGFDPAARVRLREELETHGGHNLDQYDHSCTHVIVAQQVAYDDPICIAARKGGKSLATETWVSDCLNMGVSLDVNSVLYTPPRDRNGIPGSKSLCICLTGYQGQARQEIMRMVELIGAKFTKPLIASKVTHLICYKFEGDKYLLAKRMGLIKLVNHRWLEDCLKAWKLLPEDKYLRSGWELELAEAEAKDSEDSENIKGDPELLSATNGPLEINRHFPSINSEATTQQGDLELYSVSKAPVEVNGRFPSINSGASIPQTTLENNVNALGNGAPEAQSRAVELAGSQEVKKSLGGPDVKTLKVDCHLESTSRKLDIGAAQLGHMANEIKNGEPNKWVEQGYENGNGNNARDLNPMCSNKDVGTGQDDMLSVGHGANQLDIQNLGQSEVRTPGIAAFEVTNENSNTKKRKYNLSVGGIVQTMEGLTPRAGKKDGSSALKSSSRVKRSLGTGEQHQVESQHQETPIEIEPKDIGFPIDLEAHPVHLQHEINQSDLALQPELMPMPVSNSGDMPPILMDISKEPNPITPSVHNDPGEANMIDEQATELSAKDKIEKDKICSPKRKDRSNMNCVKSGTSNRSKTRKKQIINGMSPITGEKSQAPVNEVVSPAKENYSDNWDTKSLERPDSSKHKRGTRSYSENENVKTISKHIVCEQKRLESALEECVEGNGKNLPSPKVDGSNLPIKLSKKAQTETDLVKVADLDKELSQSAATAKVVKKSKGPYSRSCSSRSKASSTLQQRFSSNEIPVDGNPQDICNMSNLAAKPNNLIDTSCTRKTLSTSLSPDKPFDSASVDKEPKQPKTTYSRNRKKCKTQVDVASVNEAVLNVMSLQGGQGEIPVNGNPQDICNISDLAAKPNNLMDTSCTRKALSTPLSPDMPLDSATVDKKPKQPKSTYSRTRKKCKTQDDVARVNKTVVNNTSLQAEQSKADVSPDKSLDSPTVDKTPKQRKGTDSRNRKNSKTDVDVASVNKTLNDKLHCEQSKEDEKPVKRSQEYWFALTGHTHEREKFQGIIKRLGGHICRDQHQWKDRATHFVFPAPLRRTEKFFAAASAGRWILTAEYLDASSKAGKFVNEEDYEWHGSGFNEGKTISLEAPRKWRVLRQKSGCGAFFRLKVIVYGECLISPMDTLKRAIKAGGGAVTATSPPYTRPLSVGVDYAIISLGIPEDDPWILQFVKHQVACVREEYFLDYVCKPTSPLDNHILYNTKKNAEAAQRRLKERIEACSSMVRDMKSGLGHSDEDTACDACGRIDREEVMLLCGDGHGKGCGRAMHIDCCRPPLKRVPKDDWFCSQCKSS

**GYMNOSPERM**

>Tplicata_BCP1

MEPKSKNKGLADPCNEDTVECSSEGQGRIFHGVRFFLVGFDPSAEIQYCMELENGGGFNV

GRYDTSCTHVIVHHLTYDDPICLTARKDKKILVTDWWVADSLDIGLPADPSRILYRPVKD

FGGIPGSKSLCLCLTGYQSQDRSDIMRMVEMMGARFTKPLIAKEVTHLICYKFEGDKYKL

AKQMGLKLVNHRWLEDCLDAWTLLPEDNYQKSGWELELLEAEAGDSEDDGVGKIGSPTPS

RQTDKPGSLFRSPRKSLGTLNSTPLQVNKKKLSMPKSLPKVFSKANSGGEQNINKEIVGV

QSKVMPGSMKCDLDSLFQKKDLMNGDLSGKVNEVHVNEAPFLDLEVPYGKEGTGIKLPVE

QRDRDYNGLVLTEKSPASAVKAFDVTGTGRIERSAGSTQVRNILGETGKEGKDDVKSLLA

VENKASPRVVPPSSGFADDNVQLRDGEGAESNVDKKLLGDATLVKQHFEIDVGRSVNADD

QLKTSEKTKMQIYKDGLSLGSDSVLNKVRMNDVGSSLLNISSNIDDVFGSGKTVKPPAEI

SKQSFASKNNILLGNDGASNFGNGNPVDSLDRNEQKTCNVVGANGLSSGFVTPNADSRQA

LSVGEKRQYKSKGFASNKKKKTMSGGMLMSLGKTMPLGSETSHEEETHKSELSAEKENLD

DGMSEKNNKGDNGFKMSSQLHASGSLPSKAPHCTTLQSIPTNDISVLSKKRGRKQGTNKK

AVVDKTLILDVGSHTKKEGAEHIDPNMQNGTGTSCNLQEKLFADEDNEISADLICDAIQL

KSDVVNDHQQQVNEGSSATAISNPKLISSNILIERPQAITSEIPRKRGRPSSKKGKSFEI

VDKFNPKPKDAENQENDKAAMIQVRVNNGMDSVSRATVSEVESPNELLKNDILSTPRTAN

HGTPDSTNQLSFKTRPGKLTYTSNVQQSNGTFDVDHNRQVSDNLEQKPVLETKDAKHLEL

ETPQHNVGEAQQINESKNNTKIVANRKSGASKKNNSKSIANNTDTTETNLSGVDEEPLDS

MQDKKQKASIVKRTPKVKLGKRATMEIPSKAAKRSRQAYENGAVKEDKDVNQVAKQEISV

MASRSEPKWFLLSGHRLQRKEFQQLVKNLGGKLLKDSHKWSYQATHLVVPEPLRRTEKLF

AAVAAGRWVLTTDYLTASKQAGRFLNEEEYEWHKSGLNEDGTISFEAPRKWRLLREKSGH

GAFHGFRIIIYGECIAPSLDTLKRVVKAGGGLILATSPPYTRSLAAGVHFAVISPGISRD

DIWIQELLGHNVACVLVDYLVEFVCKPSYPLDKHVLFNTHGAVEKLLKSVEMNIQSALSN

SIHQLTPQVYSGAQMKVSEGVTGEHVSEITCTTCGSADRDDVMLLCGDEQGRGCGIAMHI

DCCQPPLEEVPEDDWFCFKCKSLTDLNNS

**ALGAE**

>Mendlicherianum_BCP1

MGCFSGVRAFLAGFDDEQVIEEDPACAAAKKDGKILVNTFWLDDCLEASECLKPVKGLHT

PSKHPGKVPGAAKLLICLTGYQGAARRNVKLMVDKLGARFSKPLAAGQVTHLVCFTHEGD

KYNLARKTNIKVVNERWLQDRQASELAGAVGHLEAWCLEPEDLYGKSGAEVEAEERRQEE

DLSKRLAAQGHELGCERGTGAFGDVPAAVPDGAAAQRPLQATDDVRTPPQPDEGIGALEE

ASEETEVPDLLADQAGADPVPGTLIADRKVDGGDRASCPATLQATSVAVGAGGDGRALAP

GAKTVELPGIVGSLHEWEAEGQAAGNKAHAEAGLEVAARAGGGAGAGAGLEDARLGEGEP

GKVEKGSSSGDGNTPSSEQLREVQAGVAAAVDVGGLTPSDLQEGGSPPPQGAASAVLDSL

AVAAERQGGAGRGSSGTSKRAGLRAPGSLAAGESSKATMVAAAAAVGGGEAASIVSIEEK

VLVHVSTEMEVGVALKSSPKGTVAAASVAATRNVRRRKYQAAGSSQEESAGDGDGDGDGD

AVAGGAPKQASAELVKGAASRPASNAGSSPAVLTRAAASAAVSAAKDEPPAGQSTGRRRA

QPSGGRSTGRPSSRRKQGASEDPGGMEQLLSPGALFADTATPAQTGAAVEAGATLAVASS

AETVVQKSKGGGIAKEPLETEEGEHGASEAPVSTPVDPLLQADLKGEGKEGEARAEWRAR

PMQSLSRAHHSWASPREDDSEVSPPRWHHAPRSTSGAEAEVEALVLPGSAGQAEQEAEPM

DVGVQQRGQAGKRGRSSVPSGAAKAGRSGAAASKGSRPSPLEVGASRPAARGRGSRAGSG

AGAEAGTGAGAGAKKAKEKAGPAALVDEPEAGKSGRRGRGRAAASKKRPAKGGELKVEED

GGSAGEKDAPATTAAASTKEAEGLGDGKAAAAAAAADDDRGQNTNTNTTDCYFTAQDSVR

SEAAKGEARPGGCGQGAPGGEQGAAPSGDDGARGHSSRGEPLGNGEGEAAAGEPGHTMPE

DSPEFATAPGEASGGAWPGVGAGAGAGSGAEDGAENGSGAGGGEGAEEADGVGDKEGLDE

GRGVAGSVPGLKGSAPAKAWRKVMASKDRSGTSASASRARKKAKAAEPEPEPELKPKTKP

KPERPAQSPAKHGKGKGEGEGERNGEGGEEGEGDGKKVKCFAMGGDSKESYRAMIKRLGG

RLAGEHHQWKNNATHVILPEPLRRTEKFFAGAAAGRWLLKPSYLEVSVCEEHFVAEEDYE

WQGEGENEDGTVQLDAPRKWRLHFESTGCGAFEGLAVVFYGASFTPTLDTFKRVIVAGGG

KVQRTSPPFKSALKSGLDFAVIASGMPRDDPWIQEFVEAQVACVTAEYLVNLVAKPGAPL

DAHVLYGTHAAAEAAWQRLEATWAEGAAERSEKRVRSASHSDLGRRKAVKRSLGSGGKQK

PAREAGKKRAKPRPVGVVEESEELGGGGGGGGGGPRGEDGEGSEDGVVCEACRRADMEDV

MLLCGDDEGQGCGRALHIQCMEPPLDRVPDEDWFCEYCSLQCD

>Smuscicola_BCP1

MVIAMAVAAAVLSADGLSPPPPFRPMAAPAAPAAAAAAADGGAVFRGVTAVLAGFTELTE

RQVAATIREHGGNVLSRYDTACSHVIVFNLALGDPICMAAKKDGKVLVTNRWLEDCLTAK

MAITPGKVLYQPVKDIKGIPSASELVISLTGYQGKLRRDIMLLAEMMGATFSKQLHANRV

THLICCKFEGEKYNLAVKTGRIVIINHLWLEDCLRFWELLLPDNYCASGDDVLKTEAEAA

QLVAGGSDRMPLDREDADAAKDTNNCEAAVGMAVEITRTAAGAAAVVDLDALTQPEDEPD

EAVTTEQGEVESVPSVSVLEKHVDAGASTTRPCDPLFSTPSSTAAKQSTSTCQASAFKQA

PRHQEALIGDRRNESDLLGARQVNHCIATGSVVDDKQVGRLRNIQSITSNLFSEKAEQER

GRGGDQVNRQQQGSEAGCAQESAKQVGIPAPSKSAARSGRWRQVNRDDEWLVYTRDASET

ERALKAGVNSRDIGFGLASKNLGSRSEGDGAALWCSKDDRDTLSFRQAGDSLTGVQSMLH

MPDAGSSKEEVVGSPVQPQAYHSAEGKLQGQEANIEDEVLHEPLERKGCNVGVNLGRKRC

QSRSSSQDADQDTEGRAGDTSIHKLFAEDSAFVSAPQEGVNKGFPVNSPLSRRRHPTLKR

RRPQLPGETLVVGHSDVGAHQASIDKADIDSSTVSEQSGEADNRVTATLSTSSHLHFSYI

ATRKARPDCGQPTKKDNLTSRSNAESRQGLEQEHIVSTSAEDKFCGSDTVGPDKALPDRL

TCSLQQCVRWFALGGRSAIKSRYEGIIKKLGGKLCRDQQQWDERTTHVILPEPLRRTEKF

FAGAAAGRWLLKPSYLDASLAANNFVAELDHEWFGDGANEDGSIQLAAARCWREQRQVSS

CAAFQDLKAVVHGDLVSPPTGILVRGITAGNGVVVGTGPPFARAIAGGLDLAIIGPAISR

EDPWVIRLLDAGVGCVAPDFLVELIAKGGEIPVRLILFESRPAVEKALAKVGTRVAESIK

SGAGPAACSSKQQLDVAGSEQASKPEVVGLLQATTKQGKRRSKSARASGSRRAAAAAAAA

QAGDGHGEPGTCNDKNRAKVDSIACTKCHRTDDEDIMILCGDDRGRGCSLAVHIHCLTPP

LKTVPQEDWFCSSCSTKAS

>Knitens_BCP1

MSKSVFVFGQLRSLLEGEGAQEVVDASEVPPGCTHLITAYNYDDPACLSARQAKVVVVRA

LWVEHCMRHGPVSPHRVVYQPPRPSAPLAGAASLQISLTGYKGIYRTTIMALCEQMGAVY

TKELSAGNTHLICYEFKGEKYNFARERELHLVNHRWLEDSGQEVEAAEEAEDPAAEDEAA

AQEKQCKAEGEVTGDERHETAGEGVTGGTGNEAEPMQEDDDGGNMQTEEEALQGNVEEGS

KSGGPAEENVGSVRPERREGWKAAGATVRVQKGLGYLAGSAQPPGWEEKERGTSRGSPKG

VQKYLDEMSSLASGGAEKGPLEGGGWEEEGGKDEDVSFAKMGLTEKTAKSANEETIKGAA

EPAEPMRSVPEGEAAAEVEVGPAVGKAGGGRLKKVGEAACLTPVVESGGANGVVPLEEEE

EEEGGPAQASSKKGRKRGKRILLESDESSQEGAADAATHEPTRPEAANEEQGEEKEDGMA

EPVQGPVQRELADEGGSEGSGDEAIACLAKGNKRKKVGQGQQGTAKKAAANKRKKKGSEA

SGAECEQDSRSERDGTKGGSANGDEAVIAEASERKQDEQTGAMPEQGQRASSAGDEQEAL

VGEEAGQPAEPKQDEEMETEAAGEPKSAAQRSPLAAAAAGLGRTPGKNALVRAKPKPIVR

RVGGKPNRAFRPPARVKAAETESGQEGPAKKPKGGAKKAVPTKAAVADIAEEAEEEDAQQ

GGLQPSEGASQEEEGQAGKRKGGPADGDEQGEKKVKSGKKKRKLASNGSVVVGRRSEEMV

TPAKGAERKSGSAGGAPEPAAEPTSDDLRHEAAGGGPKPEEECGTASALDPTKTGAGVVE

KECEGGKEKKEGKQSKKGGKKPQGVKKGTGEIEGGDIKEAAKADSLIEANKENIKSEAEK

AAAQGVSCEAKSSLGGAQTAPYFAFGGSGEQKDSMVKKVKALGGRVARLGHEFDRKTTHV

VHPPPLKRTEKVLAALAAGLWLLQPAFLTASKPAGGWATEEEHEWVPGTGDVGAALDLSA

PRHWRQRRAETGRRALQGLRVVLYGDFQNPTTDALKRIVLAGGGQVLASGPGPLKLPAAS

ASSVQVAIIGEGVLSADVCVTELLAAGAGCVNATWLLDTLSLPRGDHSQHVLFGTEAAVA

AALESVNSAPAEPQKGTGKGVGKMGKAGPKAKGVLQPHSAGGSLKTSAKTGKKAGKDKGT

AKSGGSVEAEDSVLEEEDDVACGVCGRVDQEAEMVLCDGRGGQCDLAVHIFCMAPPLKQV

PEGDWFCSICQEGQGGASP

>Cbraunii_BCP1

MTETGCYSATKLENDGGRLLDSATTPQVLHFRPGSGTWVAWVRASVVSSGCAQWVRAQKL

LMASQFARSPITVITTILLSYKVLYRPVPDVNGIPGAETLAVCLTGYEKPARIEIEETVK

LIGAGFTKHLSKDNTHLICYKFEGKKYEMAIRLRLHVVNHLWLEDSGIEAEQLGTGKLPG

AAAKNPVGPAVGPVSSATVGKQSDGRDPSSRMPMVDTPTSKRPGHLSPLKERNRIDFPSL

QPDKLGQNEHVTGSRSTGYRNDIKDRRMQSPTQQLNGPDDAARRFLGDGLGSPDSARKTV

GDGLASPDAARKIEGSGARDAANPSSLSKQGDIDQSAGTASKVSATVCLSSANPAAESLW

AGPDVDETWANDNVKSPGGNVVITYVKRGSRRSTQKGGPPVDPDKQLSRQKTVKDIAVET

GEAEGGISATVAGTERTPILDRRKNVEIDLDDEDTQPDDDDSVQRLWMERHGGAVGKNNE

ADSSEVNSGDGGGPARKEEGREEGVDVTVSDEQMLKAGVGTADVEENVEKQAVSASECGT

EVVGGDIAKQAVTKRSTRQGTPTGAVKKGETEGGPVGVKNSETVPSVTERRKDAPTAERK

SIELRRSDIDSPTINTSLLEDDQAAGQKSGRRRSSAGRMCHATVAGRKSPSALEDNGPLR

STGGAKPQVEENDGETRRAVDDSGDLSDKSGTGKGKDSIVSVDGKQGSSGQKRRMDDDRQ

EVDLQSDDAGDEVKADVGSPATENRKRLKGDKANDGSRRRSGGRGSGGRGSVKGSGRGSG

RGTGGRAQSKGRGRGRGRGGRTVDASPTVQEPETPNLLSRGREVAEALLQASSVPGNGGK

DDASLAYVSSGEDAGKDASPEASPAEKGGSKRTERGQKLLPVKRNLETKEPTQGSRNLRA

KVGTKMDIGEGKGESDTDGVSEDDDKDWLSRVMTGVKNAADAATKAHRAGGALRKKKAAD

KLVSGAPTQDGEEGVGGSGKAIEEDPSREVIGVEDGDHDDGSYVPDNQSKKRDGRGKAEN

RPGGGQGRGRGRGKGKGREERGRLEKLGDNDLSSLKKPLSKTGRQGKKDDGSGIVISEDR

QNDAVDGRGVDADNSARIPANAVSGLSDADEKNPGVSAVACAGDIESEVDEANEENGIHD

RIKRKVREEIEARVNIRRNMKDGLKAEEPVSKEKAPKAKGKGVQKKVRGRGGGRGKMKGE

LGSSAEQAESLEGDGSRSTNAKRLQDTRGGSGEGEAHDESVGPVVAKVGENGGGVKRSMK

ACVSKANNVTAVKCFALTGDYGEKEQAKAIIKRLKGKLCGEKHTWKDSATHLILQAPKRT

EKVFAAVAGGRWLLQPSYLLSSLEAGSFVDESKHEWSEESTDADDVIDLRAPRKWREQRE

TTGCGAFHGLKVVFYGAEGFTKPPMDTLKRAIHAGGGHVSAVKPPYTRFLSKGIDFAIVK

EGVDRDDDYVEQFLEKGVACVTPEFFLDFVLKPSAPREQHILYSTEDCVKAVLKRQRARI

DAATYVSQLPPSQHECEDSDQSANLDVLACEVCGKADKEDKMVLCGDDLGKRGCGIGTHI

FCMTPPLKRVPDEDWFCNKCKSPYDWTSL

>Corbicularis_BCP1

MVESESEMLTPPNLFGGIKFFLAGSFTATERNHYAMALNTHGAEGLDSYDSSCTHVIVKGLARDAPICCSAEKDGKVLVLEDWVQDCIKRGELVDSDQVWYRPPWELKGIPNASNLVIGLTGYQGDRRGKIMNMVLMMGAKFTKPLRTSDTHLICLKFDGPKYEMARKFNMHCVNHRWLEDCLDEWKLLPEDEYKTLSGEEVDKAGLVNETPEDFPPPPPPSSVPVFRRTPHYAQALPAPAGEAGHSTLSHDERSGHHDHTKSSHVLSETRLNARQTVPPAIEASVEDDGPSRGVLKPLPIPVRESTSLAGKKSQSGAPLVPVRGSTTFAGKKAATANGRTVRESGMDDTNRPQPYRSADPADMAVGSISGSGTPTAPGIGANPSVVWNSGGSQSGCGGAVPETTAAADDARGKQLAGEPSDEDDEEATQTDNELTQPGRAEALIKASLMGPGLMDHMGLSPDINPAERPHALAVAAAAPKEAAVAADILTSPNGTGRGRGRGRRGGRGRGAAGNGRGDREPSGSTRRKKNGPPAAVPNMEDDAGLASKELEESGNGERRRLSQALVAATGDDAQKKDGLKKATSSLETLQTDDRNEVAAGVAAAVQVGGFFKHSSSSDGVAQEPTAGQTGGKRDIRMAGSPIDKSIGAFGQRLGGALVQDGAGGQVEGGGSGREEDGEMSDSPMRVGGRNLGKRRYRGLESSQELEEEAEGRKRSKEDEPSESVQEKHGDIGSDVRRGMMESEGACAREANAEMPTALETDANDLLEAASKGKVVPTAMDVEPDQQTGKRREKLGGRSLKARKLNGGPGLQVNPMSSSPCSTLPQQGTVTAPIADSSGGNLLVSVAAAVQAPEKREVAAASAHSDGAEQQPGMTAGGKGGRLSGVKRGGGGRGRKAPPVSVASELPEEVGEKAGNVPRMPAELEAPGERAAAAAEGRHGKSTTGGDVEVVAEKPEGKRKQSRGPPKKSAVGKGGRGKQVVASPLPKQASNDDGLRAGTGAAEEDGWTEPEEEQGDVEVVVEPVVEAVDGVCEEHAASMETTEDKVPPIEDPERRDAEPVVEDQFLEKSSKGVPEKMEAKRGRPKSKKAEAKVKKSSVKTADVKAKKPSIPAADSNKKGRAIVQTTNGVKKGAEVSGSGTGVRKVLIVKKPMCFGLGGLTDQKEGFKRLIRKLKGRMCGESGEARHEWHDRATHVILTRPLRRTEKFFAGCAAGRWLLTPEYLDASVKAGAFLDERLYEWHGVGNNEDNTVDLTAPRHWRKSFEATGHGAFHRLRVVIYGDTIAPPQDTLKRAIEAGGGKVLAMVPPYNGALASGVDFAIVSQFCLRADVWVSKFLDAGLPCVSPEYFVTLVAKPKEPLDQFVLFDMEVPARAGLQKLEEARSIPDDEEEGGIFDDGRQGGGPEAQEGRHGRQDEDVDDEAESEDWGDDGTRDDMAVEENGADADDVPCQVCKRTDGDDTMILCGNEDGSKGCGAGVHLQCMNPPMTDVPEDNWYCEQCANV

**BCP4 SEQUENCES**

**MONOCOTS**

>Platifolius BCP4

MDGDAGDGGETQILDGGTPPLGSPFSDGGGTLSEREDAVLYGETQALDDDAAETQVVEGL

EGEEVAGDWDETQLVGGCEDDGLGVGEKVKTQPVGECVDEGNGGDTRDAGDWTKTQLVEE

CEVEGGNYVFDNVAKTQLVEECEEVEERVNDSGNEGVGDWGKTQLVEDTDEEIDDGGGGT

LRECTQVLSDCEELANDGRDVKLPVEGSNAVVDAGVEDEEGSTGCRPMRLAAVRVASIRT

CGLAARKAFTVSSMQQGRQQTLASETHLLPKLLNNSISCSTSVGELPPDGRIDNDSHGYV

QNHEKDGARSRGRCSTAKKLFADMMPEDDESNTKNFAGLSYVESQEPGDLSQANALEVVE

RLILINGNGSSQEATPNKFEIAKSRPISSKRGALILAEKVDCSGGSNGRAEIFDWVNSRE

DDRGGEFFSKNKDILLHSPTGKGKSQSHPPKAKKCSMKIAASINDKGECKKGTSSKLSEN

VENFSLSDSRLLKSNVKNKRTSGNRTKKNLFKDINDLSNAKYLQRQQQKVSIASHDVGPD

TQMAIEAIDLLAQCSPAITLLEGDKPPSDRDMSENGNYKGTKSSSKNSCPQKRTSSVQEG

ATTHSKRTKITEMNPEPQRERHGGSKMRENSEEPRAKTRNKRTKSAPEESKVSKTFLDGN

EYQGTPVAHCTRHSSRKGLLEYTKLCSNKHVKKIKNLTGDSSGNGKVQNKVIANKPEQLS

SERITDSSLNHVEKESTQHTSADNAQNLEPGRDERRIDVACRDPSSHPKQRRTATAVVHT

TATDLEMPPEMATDLEMPPEVARSSKRRRVFIRSVSELVKYAKREPSNVRATSMLSSIIA

RSSTAFPILNSSVRVDNKTSTDFCSTGQQQNEPSRVEDANKTPKSNTQVQNSALKTPSKV

INELSPTFSPLNPSKCSSRSLSKPFVARELLKLDCEKPLSNQQLKDSRRRQDMTSASILF

SHHLDEDVIKRQKKILKRLGICEALSISDATHFVADSFFRTRNMLEAITLGKPVVTSMWL

ENCGQAGCFVNAKKFILRDAKKEKEIGFSMPISLASACECPLLQGKRVFITPNVKPSRDV

VTSLVMSSSGQPLERIGRSLMKEKEVPDDLLVISCDEDYETCVPLLERGAGVFSSEFLLN

GIVIQKLEYDRHRLFSENVKRTRSTTWLKDKVHGRFVPVSKCS

>Spolyrhiza_BCP4

MTPMAVDAVRSPSGCDNKVISVVQNYAGVIKKNSEIINAREFFLDSRVDEQNLDFGIAMQ

DQHAEEAANKNISFTARKLNFEEKSPGKSDDGNPQSLCSPFRDNPVAGLSYIYSQEPAEQ

LQADALNAIDKFLFINDVGLSQEFNDGKSVEVKKSPVSSAAGAHNLLRMSVPRSPVGKSG

IFDWVDSLEDEGGGDFFRKRKNLLLGSSEHLKNSHTQFLKKSRLPRGAFDKLGDTEGRCP

NADSKRMRLTFSEPRLKQDISISYNSLDSSVKTSRALLAQIAGKLNTESPTMNQSNETSA

ERREEDTYDIGPNTQVAAEAMEALVCGHFSEHVDRADNVADHVMSPVYAPVNKRGPSSNL

GGSIRRSMRIKMLHADANSPGKSSKEKESLRRDLRKSARMKHGHEISEKKTRKSFRTETA

QRKGKLPSPLETEQVYKAVAKSGTERVGESLGSLPVDDQTCPSNECVLRVNSLSVTPVAH

RTRSSKENTALRHGKRSVDYETSEKGLMVANPSALKGKRSRIIVSQPETREASKEVKVHV

GNSSGEFHVEESSVLPEKPCTTNTLLEETSVDKTLRTTHENISRHTDEFVNFKGPPSSRH

GSTAASEIADISPKIVENQSKDTEKGVSPSSGVRSRSFSESHHGTSEGESVRSLKGDISI

ECDSSHGTCSASVVHEKAHIEEVKAETSVSFGCANSKQGAPAISNDQDALEKGGQERVQP

LDSTSTLPTSKLSTLSPVCTSVDSLRATCKRRSSRSLVARELIRLQDMQASPDIDFKEVR

RRRDIASIRVCLSHHLDDDIIKQQKKILARLGASIVGCVSEATHFVADKFARTRNMLEAI

ALGKPVVSPQWLESCGQACCFIGEKNYILRDVKKEKEIGFNMLDSLSRARQTPLLQGKRV

FITPNVKPGRELVADLVRAAQGQPLERIGRSAIK

DGKVPDDLLVISCEEDLAVCTPLLETGTEVFSSELLLNGIVVQRLDHERHRIFSDRIKRT

RSTIWFRASSSGRFLPVGKCSNNYCLLMGYVLDDSSPSSGPLKAFYYSGWNSMNMKNTFF

TKGEKN

>Jascendens_BCP4

MAGAAGDGQETQILDAESPPLGSPFGHCDATQEEGDGGMEEVGGGILYGETELVEGLEEE

AGNEGAGDWVKTQLVEGCEEDCNGVGEWAKTQLVEECEEEEEEAGNDGGDEGISDWGKTQ

LIDESEEDTGNDGGDELRGDTQVLSDNEGLLNDGKDLALGMEERGCKEHAGVEDGDDSES

SVDSDASTDQEGGTGYLQRRLTSVRVASVRSCGLAAARKYNSKANKHVAASTVTSVQQTK

QQILAGGTHVLPNSWHNSTSCSISFGEPPDCGIDIDSHGYIQNHQKDGAKSRGSCSTVKK

LFADMTPEKDESNTKHVAGLSYVESQEPGDLSQANALEVVERLILINDVGSSHEASTNKS

EIVKSPPVSSKRGTLILAEKVDDRSSNGKSEIFDWVDSREDDGGGEFFSKNKDLLLDDGK

GKSKSHSPKGKKSSMKITIASDKGKDKNEASAKLCGKIAKFPLSDSRLLHSAVKSKKASG

NKTKKNLLKDLDDLSNAKSLEQQQEKVSIATYDVGPDTQMAVEAIEALGHCFPVTTSFDG

DKASLDGNTRENGNVGRAKSSSTNHSLRKRTSRVLEGVTTRSKRTKIAEPNPKPEKEQSR

GSKMQERSEEPREKMKKKIKLAPEKSNVSKILVDRNDYHIRGTPVARRTRHSRRNDLLEH

TDLLSNNRVNNSNNLTDGTSIVGGGENDHIADEPTEVMVSARIADSSLNQVEKEKAEHTS

EENVQNLEPCGDEPRTDIACKDLSSHPKQRRTTTAIVSTKASSVTLAVTDHAVPTEVERP

SKKRRVFIRSVSELLQKAKREPSNSRAISMLSSLRTISSLASPTIKSSVKRDNTSAGVSS

FRKQQKEPSHVEHARDSLKNMAQVCSSAMKTPPKVINEVSPTFTPLNPSKTSSRSLSKPS

IARELLKFEPEKPLSNRQLKDSRRRTDMASACVLFSHHLDEDVIKHQKKILARLGVREAS

SISDATHFVADSFFRTRNMLEAITLGKPVVTSMWLENCGQAGCLVDEKKNILRDAKKEKE

IGFSMPSSLASACQHPLLQGKRVFITPNVKPSRELVTSLVKASYGQPMERIGRSIMKEQE

VPDDLLVISCEEDYETCVPLLERGACVLSSEFLLSGIVTQKLEYERHRLFSDHVKRTRST

IWLKDKVCGRFVPVSKCS

>Macuminata_BCP4

MVSTAGVDDEETQVLDLDSAAPGEVPVLYGETQALDGSDGSDGEGDRGIDERGETQLLDE

DEETAAVDSGGEGTDRTEVLSDDEGVSNDDATHYGDREDGGNVDSRPELRVIGGEKLCLA

EDKKDNLVDSGALTDGGDGDDDDDRNAGSKMRSCSVVPVAAMCSSGLAAAQYLVSRRLEN

VFRPFNNGMHSEMENCAVDEQIDLAGIGINNWKNRGNDISSLSNTVDKLDLDHEMAGIQR

SNQNYCIDDTKSRCCNMRVKRLFKEFLPSENHKSTSKDDSTLSKVDYSHLLTTDYALAGL

SYIDSQEPSDLSQANALEIVDKFLSISDVGSSQEIIKVETDILKSPLVFATKGVQLLAEK

TDCRSPVGKPGIFDWNDSIEDEGGGELFSKRKDSFFERSCGARKTRSHPPKSRLAISGTT

RDAVDKSGELGDNLKIYDKGTVLVNSDSRLMIPNPVISERFRISEANIRKNLFKDAKGES

KIESLEHQLDATEVEGSLDGIHNVVPDTQLAAEALEALVHESLVNAEKEETRDTFTGNLT

SNSDKSPIMKTASSKNVSLPKWTSVNDSQVVMTRSKKRKMLSTELRGSLNLPRVWSSSRM

KNSLEDTTAKRQAKRGKAKLDGQADMSFISGHDSSMSTKKTKTQAKDDRHLDEKQKQHHN

NLVERHPARCARYSKSTKLLKQNEALPHGGKDPNESVNKNALRSVESPDNHVTDKGCPSA

GLDLATEVKYRATHAKFLNGAPLGEELQTFSLTKDGCHCPKRRRTSRVNSGNLNDNLNKA

SAMSDAGASETIGRSSEQVGKRKIFIRSVTDILNKVKRKKRSIFTYASLETDREPSSTTL

VRIIRGMQSSLSTLSAEKAEQPKLCSKYVEDAAANNRLYRSLKAKGQPNDLACTTPSRDK

NAVSPIYTARYPPRSCNKSVSASSVASMLKDMRRRKDMSSVRVLFSNHLAEDTIKHQKKI

LARLGLPTASSISDATHFVTDEFVRTQNMLEAIAMGKPVVTPMWLESCGQASCFMDEKYY

ILRDSKKERKIGFNMPVSLARACQQPLLQGKRVFVTANVKPNRELIASLVKASHGQAIKR

IGRSALKQGKAPDELLVISCEEDYSICMPLLEKGTGIFSSELLLNGIVIQKLEYERHRLF

SDHIKQTRSTIRMRLYGGNQFLPVTKCA

>Sitalica_BCP4

MKAAPSPAAMGGTAGNGGGGGGDTQVLDGGTPPLGSPASDSDSDATQSGGDDGDLYDETQ

PVDEAETQLVDGVDEEEEEEEDVAGDWGETQLVESGEEDDCDDGDQVKTQLEVENGDDGG

GAEDNAGNWTRTQLIQECEVDGVNNGVGGMVETQLVEDSEEEEEEDGVNGGDELSVDEWG

KTQLVEDSDEEIGDDELSDRTQVLSDDESLSGDERDAKSGMDKMDVELGMEGNIEGLNGG

VEKLGGDENLVESDASTDEEGDTGSGHIQMKLPSVRVASVRTCGISEARGTMSVNGMQQG

KQKFSSSAIHPLPKILDESTSFSTSFGGIDNDSRGYVQNHDKDGTKSRDKCSTAKKLFAD

TTAEDGESNIRCLAGLSYIGSQEPGDLSQANAFDVVDKLISINGGLSSQETTPNKLEMAK

PRVSTKRGTLMLAEKVDIGRSSNGKAEIFEWVDSREDDGGGDFFSKNKDILLQKPAGRGK

QRSHSTRAKMSSKNSPGENKIGESKNKRSLKLPGRSETLPLSDSRLLKSDVKSKRASGNR

TKKNLFKDLDDLSNGKPLEEEQEKADVALHDVGPDTQMAVEAMEALVQCSPAKGQPLSDR

DTRAEKSRIAKSHSKNDSPQKRTSSIQGVTTHSKRRKVTASNTNPQKEKMQENSERIVKI

KHKQTKSVPLKSKVSKKFIDENKYCGTPVAHRTRHCGRNDPSEFTDLCSNKQLKRGKKLT

GDGSTVGEVQKKHIKNNPEKPLISEKTTESGSSHFEKESAEHTCANDDQDLQQSRNGSTQ

RTGVNNVQNLVACRVEPTTDVPCRGSPSHPKQRRTPTAMVRSKPTTAAKHEIPTEVARPS

KKRRIFVRSVSDLLKYAKREPSNGRSASILSSIIASPILNSSVRDDGKTSDLSSSAQRLK

ESSHVEDTSKSPKSNAQVQNSVIRTPSKVVKELSPTFSPVNPSTGSNRSLSKSSVARELL

KLDPESALSNQQRNDSRRRMDMATVSILFSHHLDDDVIKRQKKILARLGVCEAFSMADAT

HFVADSFFRTRNMLEAITLGKPVVTSMWLENCGQAGCFIDERKYILRDAKKEKELGFSMP

MSLASAVKHPLLLGKRVFVTSNVKPSQVVVTSLVKASSGQPLERVGRSIMKENDVPDDLL

VISCEEDYQTCAPLLEKGAIIFSTELLLNGIVIQKLEYERHRLFTDRVRQTRSSRWLKDT

VRDRFVHVPKRPRG

>Acomosus_BCP4

MVAAPVNNEETQILDSDSPSIGDPETRFDGAERNVVGGVDVGDGDLVKESGVLYGETQAL

DDSEGSGGDGDFDVGDWGKTQLIEESEEDAAADDDENDEGTAVLSADEGLSDDGATPGGN

ERKAEEDAELGLETAKDENLSSGDGKDENLVDSDASTDDEEGGEVRVASIRSSALAAAQN

FLSKSRNTDSRSTCSNDSTNKTQGNTIDHSHEFTQCYSKEGSANKSKSNTVKKLFYDVTP

QEEESTSKMESTHLPAIGHGIAGLSYVGSQEPGDLSQANALEVVDKFISINDFEPSQEAL

NRGKAAVLKSPPISSAKGVRFLAEKVDHVSPVGKEGIFDWVDSLEDEGGGEFFHKRKEFF

FKSKVDASKSQSHPPKNRSGIYDKSGKEDDASPNTGRKLANLAHSDSRLLIQNTGVSKRI

HQTKTKRNLSKDLDEQSNSKSLEKQVEGNYDIGPDTQMAAEAIQILVHGSPIRHTAEGDK

QCGDMNGNDNSARVKDSSKNVSRRKRSRDSKGIPTRSKRIKMLSSKSDDKRPIISSENLR

GSRRKRSLEGKTLETETNRMKSNLKGEKSSQLEFIPGEYRVYGSPIAHRTRHSKQANSKK

KVETSSVKVRNCCTRSSKKVNLVKQAEILCNGSEKYGNEMMDDTCKAAGTEVELVTNEPG

SVNNDNKCSNSNKDQPKVVDTISTKPVTATQPSKKTKRVFIRSVSEILDRVKRKKRSSST

MRASEDIRSILPGLRTRSSVHSLLVTHSSGKNSNQPTCTPSLDKAGSAGSPNEKVRQSSI

GRTTSKVVEEVSPICVTQGPSRAWDKGLSRPSIARELLRLEPPEASPNKEWKDLRRRKYM

ASTCILLSHHLDDDIIKQQKKILARLGVSVAASISDASHFVADKFVRTRNMLEAMALGKP

VVTPLWLEACGQASCFIDEKNYILRDTKKEKEIGFSMPGSLARACQCALLQGKRVFITAN

VKPSQELIASLVKAAGGQPMERIGRSVIKDEKVPDDLLIISSEEDYDICIPLLEKDAGIF

SSELVLNGIVIQKLEYERHRLFSNHIKRTRSTLWLRNKDDGQFLPRVLFLVIGS

>Sbicolor_BCP4

MRGETGNDGGGGDTQVLDGGTPPLGSPSSDGDATQREADDWALYDETQPLDDAETQLVDE

LEQEEEEAVAGNWTETQLVESGDEDGGDDGDQVKTQQEVEDGEGGNVGHGAEDNAGDWTK

TQLDEECEVDGVNTVVGDMVETQLVEESEEEDDDKDGLDDDGEHDLGELGKTQLVEDSDE

DIGDDELSDGTVVLSDNESLLGDERGVKSGMDERDAKLGMEGRIEGLNGGIKKHDDSNNL

VDSDASTDEEGGIGSGHLQMKLPSVRVASVRTCGISEPRDTTSVNSMKQGKQKASSNAIH

PLPKIVDESTTCSTSFGGVDNDSHGYVQNHDKDGATSRVKCSTAKKLFADKAAEDGENNS

RCLAGLSYVGSQEPGDLSQANAFDVVDRLISINGGLSSQENTPNKLEIAKPRVSSKRGTL

MLAEKVDIGSSSNTKAEIFAFVDSREDDGGGDFFSKNKDILLPKPTGRGKSKSHSTRAKK

SSTKNSRGENEIGEAMHKRNTKLPGRLETIPLSDSRLFKSDVKSKRASGNRTKKNILKEL

DDLSNAKSLEEQEKADVALNDVGPDTQMAVEAMEALVQCSPAKSLSAEGQPLFNRDMRAE

KSRITKSHSKNGSQRTSNIQEGVMTRSKRRKVTNFNTKPQKERLRGSDMQESSEPIVKVK

HKQTKSVPEKSKVSKKFIDENKYHGAPAAHRTRHCGRNDPSAFIELSDKHLRRSKKLTGD

TSTIGQVQNNHVATKSGLSFFEKESTEQTCTNNDQDLQQSRDGSAQRTSVNNVQNLEAHR

VEPTTDVTCRDSPSHPKRRRTPTKMIQSTAAAAANHEIPSEVAKPCKKRRIFIRSVSDLL

KYAKREPSHGRSASMMSNIIEKSLAASPVLNSSVRDDRKTSSDVISSAQRLKESSHVRDT

SKSPKNNPQVLNSAMKTPSKEVNELSPTFSPANPSKGSSRSLSKASIARELLKLDAEKVL

SNQQRKDSRRRKDMTNVSILFSHHLDDDVIKRQKKILARLGVCEAFSMADATHFVADRFC

RTKNMLEAITLGKPVVTSMWLENCGQAGCFIDERKYILRDEKKEKEIGFNMPISLASACK

HPLLLGKRVFVTSNVKPSQVVVTSLVKASSGQPLERVGRSIMKEKEVPPDLLVISCEEDY

KTCAPLLEKGASVFSVEFLLKGIVIQKLEYERHRLFMDRVKQTRSSRWMKDTVEDRFVPV

PKRPRT

>Bdistachyon_BCP4

MVGGEGEETQVVDDEGTPPLGSPVSDSDATRSGGEEDANGDTARSGGGEDDNGDTTWSEG

EEDDNGVLYGETQALDDDETQAVDDELEEEEEEEVAGDWAETQVVEDSEDDIDAAEQMVT

QAVEECGEDGDIVGEKVKTQVVAKCEEEEDGDGMRGDAGDWARTQLVEECEVEDGGNGDW

VDTQLVDECQEEERVSDSGDEDVVDWGRTQLVEDSDEEIGDDGDDELSEGTQEASDDDGL

LNDETDVKAHGDTKNSGDSDASTNDEGDTAQDTRQHSTSGTNLLTKIVDNSTSCSASLGD

HPGLGIDDESYGYVQSHDKDGSKRKGRCSTAKKLFADATPKNNESKTSCFAGLSYLGSQE

PGDLSQANALEVVEGLISINGGVSSQEPTPKKLEKAKPPVSLKMGTLKLAEKVDRCRSSN

GKPEIFAWVDSLEDDGGGDFFCKNKDILLHKPAARGKSKYPRARKCSTKIAPAENKITNS

KLCGRIETLPSSDSRLLKSDVK

SKRASGNRSKKNLLKDLDDQSNAKPMEGHQQKVSVALNDVGQDTQMAVEA

IEALAQSSPAKLSVEGEPPVNRDTGVGSKMAKIHSKNGPQKRSSSIQEGVMTRSKRMKVT

TDLNPRPQKGRSRGTKMQENAEDHGTKTKHKPEKSVPQKNKVPNIVDGSKYGGTPIAHRT

RHTRNNISEYSELCSNKHLKRSMNLTADNSRVGEVRNSHIAYEPEQPTVSERTTEYGLNS

VEKESTEHTCANNAQNLQKCRAPSSHPKQRRTPTAMVQAIAPAVTQTTTDHEIQPEVARP

SKKRRVFVTSSELLKYARREPSNGRSTSVLSSIIAKSSASSPILSSSVRVTSKTSGFSSS

DQPQKEPSDVEDASRSPKNNFPVQSSALKTPSKVLNKLSPTFSPLNPSKASSRSLSKPSV

ARELLELDPKNALPNRQRKDSRRKDMSSCSILFSHHLNEDVIKRQKKILARLGVCEAFAI

SDATHFVADSFFRTMNMLEAITLGKLVVTSMWLESCGQAGSFVDDKKYILRDAKKEREIG

FSMPISLASACKHPLLLGKRVFVTPNVKPSRQVITSLVSASSGQPLERIGRSIRKEKEAP

DDLLVISCEEDYESCVPLLEKGGKVFDSELLLNGIVIQKLEYERHRLFSDCVKQTRSTRW

LKHAVQGRFVPVSKS

>Sviridis_BCP4

MKAAPSPAAMGGTAGNGGGGGGDTQVLDGGTPPFGSPASDSDSDATQSGGDDGDLYDETQ

PVDEAETQLVDGVDEEEEEEEDVAGDWGETQLVESGEEDDCDDGDQVKTQLEVENGDDGG

GAEDNAGNWTRTQLIQECEVDGVNNGVGGMVETQLVEDSEEEEEEDGVNGGDELSVDEWG

KTQLVEDSDEEIGDDELSDRTQVLSDDESLSGDERDAKSGMDKMDVELGMEGNIEGLNGG

VEKLGGDENLVESDASTDEEGDTGSGHIQMKLPSVRVASVRTCGISEARGTMSVNGMQQG

KQKFSSSAIHPLPKILDESTSFSTSFGGIDNDSRGYVQNHDKDGTKSRDKCSTAKKLFAD

TTAEDGESNIRCLAGLSYIGSQEPGDLSQANAFDVVDKLISINGGLSSQETTPNKLEMAK

PRVSTKRGTLMLAEKVDIGRSSNGKAEIFEWVDSREDDGGGDFFSKNKDILLQKPAGRGK

QRSHSTRAKMSSKNSPGENKIGESKNKRSLKLPGRSETLPLSDSRLLKSDVKSKRASGNR

TKKNLFKDLDDLSNGKPLEEEQEKADVALHDVGPDTQMAVEAMEALVQCSPAKGQPLSDR

DTRAEKSRIAKSHSKNDSPQKRTSSIQGVTTRSKRRKVTASNTNPQKEKMQENSERIVKI

KHKQTKSVPLKSKVSKKFIDENKYCGTPVAHRTRHCGRNDPSEFTDLCSNKQLKRGKKLT

GDGSTVGEVQKKHIKNNPEKPLISEKTTESGSSHFEKESAEHTCANDDQDLQQSRNGSTQ

RTGVNNVQNLVACRVEPTTDVPCRGSPSHPKQRRTPTAMVRSKPTTAAKHEIPAEVARPS

KKRRIFVRSVSDLLKYAKREPSNGRSASILSSIIASPILNSSVRDDGKTSDLSSSAQRLK

ESSHVEDTSKSPKSNAQVQNSVIRTPSKVVKELSPTFSPVNPSTGSNRSLSKSSVARELL

KLDPESALSNQQRNDSRRRMDMATVSILFSHHLDDDVIKRQKKILARLGVCEAFSMADAT

HFVADSFFRTRNMLEAITLGKPVVTSMWLENCGQAGCFIDERKYILRDAKKEKELGFSMP

MSLASAVKHPLLLGKRVFVTSNVKPSQVVVTSLVKASSGQPLERVGRSIMKENDVPDDLL

VISCEEDYQTCAPLLEKGAIIFSTELLLNGIVIQKLEYERHRLFTDRVRQTRSSRWLKDT

VRDRFVHVPKRPRG

>Bstacei_BCP4

MGGGAGEETQVVDDGGTPPLGSPVSDSDATRSGGEEDDNGDRTQSEGEEDDNGVLYGETQ

ALDDDEIQAVDDDLEEEEEEEEEKVAVAGDWLETQVVEDSEDDIDAAEQMVTQAVEDSED

DINAAEQMVTQPVEKCGEDGDIVGEQVKTQVLAKCEEEEDGGGMRGDAGEWARTQLVEEC

EVEDGGNGDWVDTQLVDECQEEERVSDSGDEDVVDWGMTQLVEDSDEEIGDDGDDELSED

TQEASDDDGLLNDERDVKEHGDTKNSGDSDASTDDECDTAQETRQLSTSGTNLLTKIVDN

STSCSASLGDHPGPGIDDESYGYVQSHDKDGSKRKGRCSTAKKLFADTTPKNNESTTSCF

AGLSYLGSQEPGDLSQANALEVVEGLISINGGLSSQEPTPKKLEKAKPPVSLKMGTLKLA

EKVDRCRSSNGKPEIFAWVDSLEDDGGGDFFCKNKDILLHKPAARGKSKYPRASKCSTKI

APADNKITNSKLCGRIETLPSSDSRLLRSDVKKSKRASGNRSKKNLLKDLEDQSNAKPME

GQQQKVSVADSRLLRSDVKKSKRASGXRSKKNLLKDLDDQSNAKPMEGQQQKVSVALNDV

GQDTQMAVEAIEALAQSSPAKLSVEGEPPVNRDTGVGSKMAKTHSKNGPQKRSSSIQEGV

MTRSKRMKVTDLNPRPQKGRSRGTKMQENAEDHGTKTKHKPEKSVPQKNKVPNIVDGSKY

GGTPIAHRTRHTRNNISGYSELCSNKHLKRSMNLTGDNSRVGEVRNSHIAYEPDQPTVSE

RTMEGLNSVEKESTEHTCANNAQNLQKCTASSSHPKQRRTPAAMVQAIAPAVTQTTTDHE

IQPEVARPSKKRRVFIRSSELLKYARREPSNGRSTSVLSSIIAKSSVSSPILSSSVRVTS

KTSGFSSSDQPQKEPSDVEDASISPKNNSPVQSSALKTPSKVLNKLSPTFSPLNPSKASS

RSLSKPSVARELLELDPKNALPNRQRKDSRRKDMSSCSILFSHHLNEDVIKRQKKILARL

GVREAFSISDATHFVADSFFRTMNMLEAITLGKLVVTSMWLESCGQAGSFVDDKKYILRD

AKKEKEIGFSMPISLASACKHPLLLGKRVFVTPNVKPSRQVMTSLVSASSGQPLERIGRS

IRKEKEAPDDLLVISCEEDYESCVPLLEKGGKVFDSELLLNGIVIQKLEYERHRLFSDRV

KQTRSTRWLKHAVQGRFVPVSKS

>Tintermedium_BCP4

MDSGETQVVDDGGTPPLGSPVSSGDTTQSGGEEDDDGLRLGSPVSSGYTTQSGGEEDDNG

LRLGSPASSGGTTQSGGEDDGGGGVLYGETQPLDEDEETEAVDDEFGGEGEERMSDDTQV

VEDSEDDAGGIAGQTQAVEEEEEEDGGGTDDEAGDGDRTQFVEECEVEDGGSGETQLVEE

CQEEDRVNDSSDDEAAVQRGTTQLVEECQEEERVNDSSDDEAADQRGMTQLVEDSDKEMG

DDGDDELSQGTQVQSDDEGLPNNERYVKDYAEDSVDSDASTEEEGGTVRSLEQGKRHALT

NGTFLRTESHEKDGSKSKGRCSTAKKLFADPTSEQNEIKSRCFAGLSYLGSQEPGELSQA

NALDFVEGLISINGGISSQEQTPKKLEKAKPPVSIKMGTLLLAEKVDRLRSSSGKAEVFA

WVDSREDDGGGDFFRKNKDILLHQSAGKGKSKIPRPKKCSTKIAPPDNKITEGCKKGTKS

KLCGKIETLPSSDSRLLKSEVKSKRVSGKRSKKNLLKDLDDHLSTAKPIERQQVKASVDL

HDVGQDTQMAVEAIEALAQSSPAENLSAEVEPPVKRDLRVRSKVEKSNPKSGPPRKRTSS

VQEGVTTRSKRIKVTEMNHKPQKEGQGGIEMRQNLEDHATKTKHKQAKAVPQKNKVAKIV

DGNKYDSTPIAHRTRHTGRNNLYESPELCSNKNLKKATVGEVRNNHSEHGLERPMLNERT

AEYGSDSVLKENTKNACANNAQGLQQSTDASIQHTSANVAQNLEPLRDEPTTHVFRREPS

SHPKQRRTPTAVVQAMAPAVTEAATGHDVQPEVTRPSKKRRIFISSSELLTYARRERSDC

RSTSLLSSIITQSSAASPVLDSSSGVNSKTSRFSSSDRRQKKPSGVKDANNSPKCNSPVP

NSALKTPSKVVNKLSPTFSPLNPSKASKRSLSKPSVAKELLELDPENALPSRYRKDSRRK

DMTSCSIIFSHHLDEDVIKRQKKILARLGAHEAPSVEDATHFVADGFYRTKNMLEAVTRG

KLVVTSMWLESCGAAGCFVNDKKYILRDAKKEREMGFSMPISLASACRSPLLLGKRVFVT

QNVKPSREVVTSLVSASSGQPLERMGRSIMKEKEAPDDLLVISCEEDYETCAPLVEKGVD

VFDAELLLNGIVTQKLDYERHCLFLDRVKQTRSTRWLKDGAHGRFVPVSKS

>Osativa_BCP4

MAGDAGDGGGDGETQALEGGTTPPLGSPVSDDGDATPSEDGVLYGETQALDDAETQMVDD

GLGEEEDGVAVDWGETQLVEGSEEEEEEEECGGGIDDQEDTQLVEDSEENEGDGEDAGGA

CEGEDAGAAAECDNNAGDLVRTQLVEEHKEGEDCGNDGAGDGLETQFVEECPEEEEKVNN

SSDDEDAGEWGKTQLVEDSDEERDDDGDDELSVDTQVLSDDEGLSNDEREVIFDMGGSNT

RVKGALEDYTKNLVDSDASTDEEGDTVCEASEQKCEALTVNSLQQGKEQDSIHGAHLLRK

VVDNSTSFSTSLGDHPDCGIDSDSHGYVQNHDKDGTKSRSRCSTAKKLFADTIVKESENN

GRCFAGLSYIESQEPGDLSQANALELVDRLISISGMSSQEPTPQKLDSAKPHVSNKRGTL

MLAEKVDCNRSSNGMAEIFAWVDSREDDGGADRGKSKSHFPRAKKRPTKIEPGEIGDCKK

CKNTKLSGKFEALPLSDLMLSSDVKGKRASANRTKKNLLKDLDLDDLSNGKYLEVQQEKE

SVALHDVGPDTQIALEAMEALAQCSPAKNLSAKDKPQLRNGKSKKAKGHSKNSPQKRTGS

IQEGVTTRSKRRKLTELTPEPEKQMYKGSILQGNPGDLKAKTRDKEAKSVPAKSNVLKIS

RDGDKCHGAPVARRTRHFCRNNPGEHTELCSNKHSKRVMNLRGGVSKVGKVQNDHIANEP

GQPMISERTAKSTSIYVEKESTEHTCATDAQNLQLRRDASSQHASENTAQGYEPCRSKPT

IEDACRENSSHFPKQRRTQTTIVQPKDPAATQSGTNHETPQDEPRPSKKRRVFIRSVSDY

VKYAKREPSNGRSTSLLSTIIKKSSAASHILNSSLSADSKTSGFSSSGNKHKGSHVEDAS

KSPRSNSDIHSSVLKTPSKSANELSPIFSPLNPSKSSSRSLSKPSVGRELLTLDPDSNPS

NCQHKDSRRKKKNTNFSILFSHHLHGDVIKRQKKILARLGVSEALSILDATHFVADSFFR

TKKMLEAIALGKLVVTSMWLENCGQAGCFIDEKKYILRDAKKEREIGFSMPTSLAAACKH

PLLLGKRVYVTLNVKPSREVVIGLVLASSGQPLERIGRSITKEVPDDLLVISCEDDYETC

SPLLKRGASVFESELLLNGIVIQKLEYERHRLFSDCVKQTRSTRWLKDTSHGRFVPVSKC

PQF

>Msinensis_BCP4

MRGEASNDGGGGDTQVLDGGTPPLGSPSSDGDGTQREADDWALYDETQPLDDAETQLVDE

VVEKEEGVAGDWTETQLVESGDEDGGDDGDQVKTQQEVEDGKGGDVGGGAEDNAGNCTKT

QLDEECEVDVVNTVVGDMVETQLVEESEDDDDDDNDGLNGDDEHDLGEWGKTQLVEDSDE

DIGDDGLSDGTVVLSDNESLSGDERGVKSGMNKRDANLEMEGSIEGLNGGIKKHDDNNNL

VDSDASTDEEGDTGSGHLQMKLPSVRVASVRTCGISEPQDTMSVNCMKQGKQKASSNAIH

PLPKIVDESTSCSTSFGGVDNDSHGYVQNHDKGGGKSRDKCSTAKKLFADTAAEDGENNS

RCLAGLSYVGSQEPGDLSQANAFDVVDRLISINGGLSSQENTPNKLEKEKPRVSSKRGTL

MLAEKVDLGRSSNMKAEIFEFVDSREDDGGGDFFSKNKDILLPKPIGRGKPKSHSTRAKK

SSTKKSQEENKMGEAMNKRNTKLPGRFETIPLSDSRLFRSDVKSKRASGNRTKKNLLKDL

DDLSNAKSLEEQEKADVALNDVGPDTQMAAEAMEALVQCSPAKNLSAEGQPLFNRDMRAE

MSRITKSHSKNGSPQRTSNIQKGVATRSKRRKVTDFSTKPQKERLRGSKTQESSEPIVKV

KHKQTKSVPEKSKVSKKFIDENKYHGTPVAHRTRHCGRNDPSAFIELSNKHLRRGKKLTG

DSSTIGQVQNNHIATKSGLSYFEKESTEQTCTNNDQDLQQSRDGSAQRTSVNNVQNLEAH

RVEPTTDVTCRDSPSHPKRRRTPTKMIQSTAAAAANHEIPSEVARPCKKRRIFIRSVSDL

LKYAMREPSHGRSVSMMSNIIEKSLAASPVLNSSVRDDRKTSSDVISSAQRLKESSHVED

TSKSPKNNPQVPNSAMKTPSKVVNELSPTFSPVNPSKGSSRSLSKASIARELLKLDPENV

LSNQQRKDSRRRKDMASVSILFSHHLDDDVIKRQKKILARLGVCEAFSMADATHFVADRF

CRTKNMLEAITLGKPVVTSMWLENCGQAGCFIDERKYILRDEKKEKEIGFSMPISLTSAC

KHPLLLGKRVFVTSNVKPSQAVVTSLVKASSGQPLERVGRSIMKEKEVPPDLLVISCEED

YETCAPLLEKGASVFSVEFLLNGIVIQKLEYERHRLFMDRVKQTRSSRWLKDTVQDRFVP

VPKRPRT

>Zmarina_BCP4

MSLSPSPKSQTMIAVDEETQRLDSCSLPLTTEYGCTMMEDFTIPLEYNTELNSYCAETQE

IHYSEEDERTVVLGEDGDDVGISDADGASSLVVDEHTNQREECEGEDATVKDCNGSADVN

RCSGSGKRSFADFRVASFRATISSPGHKMSHKYTNPENSSPIRFEESYREKVVNRIQSSL

ENKKDDKIHFNSGDNTQKVSRKMTARKLFDEQPSDEEKDASANETISNVGEDLPGTFTTL

DFENSIAGLSYVNSQQPSDFSQSNALKAVETYLSLNDLGLSQNGGLGNPDGLKVPSVSSF

NGIKLLAKTSSKSRSEDHIRGTDAFDWFDAGEDESGGAFFNKMKDSIFVDQANPKKTRKH

PPKSSHIVPRKNGHPLDSGICKKDTVHPSLSKTKGSFSDSRIEILAGSSKSQIDAQPVII

QQMDAEKVDSGMDHTDDVGPDTQMAVEAMEALAFVPCTEEINKKTSTCVEDINIFNQNRT

TKKRSVRHASQQKIVCPVSDFEGSLRRSKRSKSSTARFKNGSNLIPSRVSDCSNEGAVTM

IGEEPIDKKAKNAEEEKKKIVKSYTTIKKKIGQKKGETGICEMKNDRTCFNDVKISLKKE

CLHIKDKSTQVRQTRQTRENRSLKNPDALFDNNSGLKHDACKTYSGLEVTTTWQPKRRRT

NLTLNCTKNSSATINPTKSTVKSTYMPQGNAELGSSQKEHIQPSETPYSSSINKINALPP

VCTSVEPHQIPKSKRLSRSSVTRELSRLEFKVEISASALKDTRKRKDISMICPLFSRHLG

DDIIKQQKKILARFRITIVSSISDATHFITNKFVRTKNMLEAMALGKPVVTHLWLESCGQ

AGCYIDEKNYILRDFKAEKDAGFVMPVSLARAFRSPLLQNKHIFITPNVKPDRNTVSSLV

KASGGQPLERTIRTIRLDKNVPGDFFVISCEEDYRVCIPLLKKGAEIFSSELLLNGIVIQ

KLQYERHRLFSNRS

>Zmays_BCP4

MRGEAGNDCGGGDTQVLDGGTPPLGSPPSDGDETQREADDWALYDETQPLDDAETQLIDE

VEEGVAGDWTETQLMDSGNEDGGCGDEDHGNDGDPVKTKQVEDEEGGDVGGGVDDNANKW

TKTQLDEKCEVDGVNNIVGDMVETQLVEEPEEDADKDGLNGHGEHDLGEWGKTQLVEDSD

EDIGDYELSDGTVVLSDNESLSGDERVVKSGMDKRDVKFGMEGRIEGLTGEIRKHDDNNL

VDSDASTDEEGDTDSGHLHMKVPFVRVASVLTCGISEPRDTMSVNCMKQGKQMASDAIHP

MPKVVDQSTKCSTSFGEVDNDSHGYVQNHDKGGAKSRDKCSTAKKLFADTTAEDGEKNSR

CLAGLSYAGSQEPGDLSQANAFDVVDRLISINCGLSSQETIPNKLEIAKPHASSKRGTLM

LAEKVDLGRSSNGKAEIFEFVDNLEDEGGGDFFSKNRDILLPKPIGRGEPKSHYTRAKKP

STKKSCGENKIGEAMNKRNSKLPGRLETIPLSHSRLFKSDVKSKQAYGSRTKKNLLKDLD

NLSNAKSLEEQERADVALNDVGPDTQMAVEAMEALVQCSPAKSLSAEDQPLLNRDTMAEK

SGIAKSHLKNGSQRTSNIQEGVTTRSKRRKVPDFSTKPQKERLRGSKVQESSEPIVKVKH

KQTKSVPEKSKVSKEFIDENKYHGTPIAHRTRHCGRNDPSAFIEFSNKHLRRGKKLTGDS

STIQQVQNNRIAIESGLSYFEKESTEQICTNNDQDLQQSKDGSTQRTSVNNAQNLETHRV

EPTTDVTCRDIPLHPKRRRTPTKLILSTSAAAANHEIPSEVARPYKKRRIFVRSVSDLLK

YAKREPSHGRSASMLSSILEKSLAASPVLNFSVCGDSKTSSDVISSAQRPKESSHDEDTS

KSPENNPQVPNRSMKTPSKVVNELSPTFSPANPSKGSSRNLSKASVARELLKLDPENVLP

NQQRKDSRRRKDMAHVSILLSHHLDDDVVKRQKKILARLRGSEAFSMADATHFVADRFCR

TKNMLEAITIGKPIVTSMWLENCGEAGCFIDERKYILRDEKKEKEIGFSMPISLASACNH

PLLLGKRVFVTSNVKPSQAVVTSLVKASSGQPLERVGRSIMNEKEVAPDLLVISCEEDYQ

TCAPLLEKGASVFSVEFLLNGIVIQKLEYERHRLFLDRVKQTRSSRWLKDTVQDRFVPVP

KRRRR

>Aofficinalis_BCP4

MTPLADLQETQPLDGDDDDDSNSQSSEDTVEADHGETLAVETERAMEDSILYGETQAVDG

GDGDDEIVAGDWIETQVVGNDEGIEDTEVVGDEGFEDTEVVGDGDDVRVLSDGVDSDAET

DDEGNDSKASVRSIASVRAASLRASGLAAARSNISKAVDVESNSSFNNSENYNNQNNGSG

GQTSINGMLTLPSSTVDYTVFDNKKCEIVRDHEMENSQKCTQDYYRDESKSRNNNQVVKK

LFSEATSAEEENTSKLNDTTGLMDSPGSLIPDNIAGLSYVESQEPGDLSQANALGIVDKF

LVINNLGLSQEADIGKSIDIVKSPPVASAKGAQVLAKKTENRSPVGNKEAFEWIDSLEDE

GGGEFFTKRKASFFERIDGARKSRSQPPKLQKVVNVGEGANSTNHQKEATLTRSDSRLMR

NGSSKNEKMHVAETRTKKNLFKDLTDKSNSKSLDQQLEVTDDDRREEGMHEFGPDTQMAA

EAMETLIYGAPINNEMNGAHASAAEVATKRTATKSAFSKTSAFSTPNTVGVSTRSKQRNM

LSTHSKKTTKFSSRSNSRGNKNLECSTGKSKLKRGKQKMEQNLNAKTSVPSNYCSGSLEG

HKELEKVGGTSKGARKCSNSLMLNDQPSSSKEVTQGQFCVNSIAVAHRTRHSKEVRLSEK

TEDIATAYGKGTNVRSRGLIAREDEIVINSGGSHKTKRKHSDTDSVQDTEVERNFMSHTK

AHNIQNLHEKEKEADCNIKGALTHPKKRRTRQANQDNPNINDKLPSSFDDIIQPKIRQRN

ITANTRSISEILDTAKRKRRSTSSHMVSDINSLASDGVKAMFVSGVRARSLKSSSCNPNM

EDAKTEESPNKKHQPAKDVDAVSPVCVAQDYPRTPGSKGRTRSLIARELLRLEASKLSPT

SIINTRRRKDMANVHVLFSHHLDDDIVKQQKKIMGRLGVSPAFSISDATHFVTDNFVRTR

NMLEAMAMGKPVVTHMWLESCGQASCFLDVKNYILRDAKKEREIGFSMPLSLARARQSPL

LQDRRVYITPNTKPSSDVISSLVKAAHGQPVERIGRSVMKDDKIPNDLLVLSCDEDYSVC

IPLLEKGAEIFSSELLLNGIVTQKLEYDRHRLFLDHVKRTRSTIWLRREDDHQFLPVTKC

T

>Pvirgatum_BCP4

MMGSKAGNGGGGTELLDGGTPPLGSPTSDSDSDATQCGEDNGALYDETQPADEAETQLVD

GEEEEDKDDVAGDWAETQLVESGEDGSDDSDQVKTQLEVENGEEGDDHSGAEDNARNCTR

TQLDEECEVDGVNNGAGSMVETQLVEDSEEDEEDGVNGGDELGVNEWGKTQLVDDSDEEI

GDDELSDHTQVPSDNESLSGDERDVKSGMDKRDVELGMERSIEGLIGEVEKLGGNTNLVE

SDASTDEEGDTDSGHIRMKLPSIRVASVRTCGISGARDTMSVNVMQQGKQNASSTAIHPL

PKIVDESTSCGIDNDSHGYVQNHDKDGTKSRDKCSTAKKLFADMTAEDGESNSRCFAGLS

YIGSQEPGDLSQANAFDVVDRLISINGGLSSQETTPNKLEIAKPRVSSKRGTLMLAEKVD

LGRSSNGKTEIFEWVDSREDDGGGDFFSKNKDILLQKPVGRGKPHSHSTRAKNCSTKKSW

GENKIGESNNKISSKLPGKFGNLPLSDSRLLKSDVDSKRASGNRTKKNLFKDLDDLSNAK

SLKEQEEDNVAVHDVGPDTQMAVEAMEALVQCSPAKGQPLLDRDTRSVKPRISKSHSKYG

CPQKRTSSIQEGVTTRSKRRKETAVDTKPHQKEKMQENSEHILKMEHKQTKSIPRKSKVS

KKFIDENKYHGTPVAHRTRHCGGNVPSENKHLRGGKKLTGDSSSVGEVQFNHIANNPEKP

LISKRTTECGSSHFEKESIKHSCANEDQELQQSSRDGSTQCTSVNNVQNLVTRLVEPTTD

VPCRDPPSHPKQRRTPTTMVQSKSRTAANHETPPEVARPSKKRRIFVRSVSDLLKYAKRE

PSNGRSASMLSSIIEKSLAASPMLNSSVRDDGKTSDLSSSAQRLKESSRVDDTSKTPKSN

AQVQNSVMNTPSKVVKELSPTFSPVSPSKGSNGSLSKSSVARELLKLDPENALSNQQRKD

SRRRKDMATVSILFSHHLDDDVIKRQKKILSRLGVCEAFSMADATHFVADSFFRTRNMLE

AIALGKPVVTSMWLENCGQTGCFIDERKYILRDAKKEKELGFSMPISLASACKHPLLLGK

RVFVTSNVKPSQVVVTSLVKTSSGQPLERLGRSIMKKKEVPNDLLVISCEEDYETCTSLL

EKGAIIFSTELLLNGIVIQKLEYERHHLFTDRVKQTRSSRWLKDTVQDRFVPVPKRPRS

**EUDICOTS**

>Ptrifoliata_BCP4

MGSLGDGDSPNDSSKTNPNDVFARAETQVFDSQFSPPPSPGEKVEDGNNYQLNIYDTVPV

EDTFETQVVGDYETQAWSLGDETQALYLGDETQALDFFNDIENMETQLLDEFDYGVANDS

DNEGSGRTEVLHDGEGIPGDDSARRGCNQSLEQEKTECTSICEQGEKDLREQRDGSNLGS

RDCTLRPVFQSTPRSEPGSVRRFTSIRAASLRASGLAARSMASKEISIDSCFVQSADLSP

DQDAVRNDGSEPKVVEEIDNIHDLKDNETEKGLRNGNSCRVGSSTVRKLFTEDSVSQDKG

LPNNGDNAAGGENLLQFPVNDGELAGLSYVESQEPGELSQANALTFVEQFIEKNNFVDFD

HQVDLGKSKGGKSKPVSTAKGPQSLAKNSNDRSKAGKTGIYDWDDSREDEGGGDLFCRRK

NEFFGTGYRAQRSLTEPRQLKKRKFDLDGNGELQDVHDGITMRSDSRGTLHNIKKNEKKA

EEAQLIVEKNLPSELDERLNADSSRGRQDAAVGKTDVSQILNVGPDTQLAAEAMEALLYG

EGIANHDVNCLQSNSKRSAEGSSRGKSKSRVSLKQSASQKRVRLSGVGVATRRKRKSKSG

TEISRCSPDTVKKFKNISEKCDRELVTLNKRRAKSMAEQNSTINGSKNTDRVSSGVIGQR

NENGSLESCQPKEFNRCLRTATQNADHSMKKQKIAKASSASTPIAFRTRSSKAVIQLKTT

DQILDNCIHDANHLMEVGAFEENVTCSKDVEASEVMHLKKKHSKLSSNQFGELKSTKPSQ

PEKLDLELTAMNNGVDGLRYPRGRRSRRNLSVQVSGCSAGMNVKVKSKDFKGSKTPNHSD

GKIVVDSQPSAENAEMNSRLDKSPREQCEALESACTSPADCITPVNAASPVCMGNGYIKQ

SCRKNLAKSCLVKEINRLIATEPEPLSPLKDLRKRRDMASIRVLFSHHLDEDIIKQQKKI

LDRLGASEVSSITDATHFVTDIFVRTRNMLEAIASGKPVVTHLWLESIAQVKIQIDEESY

LLRDTKKEKEFGFSMPASLARARKHPLLKDQRVLITPNIKPSKETISSLIKSVHGQAVER

LGRSALKDDKLPDDLLILSCEEDYKICEPFLEKGAAVYSSELLLNGIVTQKLEYERHRLF

VDNVKRTRSTIWLRKDGHKFHPVTKLR

>Rcommunis_BCP4

MGSLQDSDAQIKPFEPNKNSSFTQSNTQLFDSQIFPGEKGVDAHAGQLVQNSVPFSDTVA

VEDAFETQVIDLCDETQVLDDPDCFEHMETQVIDGLNSDGEETDKTEVLDDTNELSDGES

LRRGKCDSLDVENTSLELTNNRLVEDLDENHISIAAPRFLSVRAASFRVSGLAARRKYLE

GINSESSSLLTSNQHSEEDTVKDNGSKTWEEADQVSDEGRYTDEVKGLINRNSCKIGCPT

MRKLFDEDFEIEGLASSSNKSVEDEEMLQLPAADDGLAGLSYIDSQEPGESSQANALACV

QRLIEENKVLFDNEFDLGKSSKGKSNLISTAKGPQSLAKKANDRGTDRKTRIFDWDDGRE

DEGGGDIFRRRKEEFLGTRSLGQRSLSKSQMAKGNQLDGYRGNRGKSSVHNEKVVHSDSK

IVLHGPKQNDKRAPEADLNIRKNLVNEFDEQSNKATSAGQPEAALTIKDMLEAPNIGLDT

QMAAEAMAALFNGNGIPNSDGNDVPGNSEDFLKGSRGRKGKKSSHSKQQSFDKEYDIGVA

TRNSSKTKKICDKSSKQPSISYQKHSETFRIELDKDLVMTRSKRAKLDAEVLLTNRTNMV

GKMPYKMAEKPIESCLLDDFDGCHGTALSGSFSVMKRKLPEEAALAPIAHRTRQALVTSQ

LRTAEMASSSFEKEMNCPMDVGAVRTTKAGKSVEAAKVLDAKGKSSELVSSQSGELEDLK

SKLRTMSSGISCPRRRRSSWQLSVQLDEPCNLDAQSRPSNQPVKIEKSARMPKRSRSTAK

FITLADLNTKRKTRSSSTACPDFPSIYPNFDGKSAGSIGTLGSRGASRNCSSSDGTKISK

DQMAEKEVKLPDRQTNIFSSLSAEHELNSDNLLKEATEPSKSKCVSPVNFTTSVNAVSPV

CIGDESLKRSCQKSLSRSCLMREISSLCATGREPISSPKESRRRRDLSNVRVMFSHHLDE

DIIKQQRKIVERLKLATALSITDATHFITDEFVRTRNMLEAIASGKPVVTHLWLENVGRA

NYYIDEQKYILRDTKKEKEIGFNLPVSLAHACQHPLLEASGRRVLITPKTKPGKDIISSL

VKAVSGQAVERVGRSALKDDTIPDDLLILSCEEDYGVCVPFLEKGAAVYSSELLLNGIVI

QKLEYERHQLFADHVKRTRSTIWLRKGSDRFIPVTKHK

>Soleracea_BCP4

MGSCRTRNHESRPIIKNLNLNVGGSSSPGGNKVPVCDDKMFGNTLPYEDTFRIHDEFDTE

VMDFDVETQPLGFDDETQIMELSGETQVMEDDEDDCVQHMDTQLLDISDGEGCEYTSDMK

SSPMGSIDVELQGRNGNQTSVFEEKETPNCHQQDGGAMHSDDVAQSSGPVRKGFASVRTA

SLRAAGLAARQAAVKNIDSGTSELHPFDDNDRKSIAEIVGGENDIEKYGKEGLSDGDKSN

VARSLARKLFMDEDVAEIESDDYFSDRREGSSQFPSSDNDVAGLSYVDSQEPGELSQANA

LEFVDRFLNVNEFKSDECVKRKNAVAEFVSPGAIAKGAQSIAQQATRKSTHAETSVYEWD

DNLEDEGGGDFFIKKKEVLYGTPKSHPKLRSFQHRELRGHQSLGKLQNVKKKQNLNDSNT

GSFRSDLKSINGGGNCKIKDVDLEKKKYNCSSGQLGLGSCSQETLATVHNAQSVDMHNVG

IDTQMAAEAMEALAAVTAFVDSDIGMADKGCHSERDQNKKEAVKNDISNQWSSLQKRARL

SAAGVVTRSRSKRTIRTRGQPTMAKFDVGLINTKAKQKRSSINNFSAENRRKSSEIGPLS

VNESLERITAADCSKVDGPSCRTGDIQCTSSLDHRSGEKRYTWSADRALEINMESIARRT

RARSSTTQCAEISQDEEGLFSCRITSEDDRQLSSKKQNAASPPAKGTLGSATMHGSPGNL

QKSSNQADTTPINLRTPASAVSPICMGDGYHTPSCKKSLSLKKEFNSLIDNEQRGSSPFK

GLRRRRDMNSVRVLFSRHLDDDTIRQQKKILSRFGVSVASSILEATHFVADMFARTRNML

EAIAYAKPVVTHLWLESCGQSNCFIDEKNYILRDAKKEKELGFSMPGSLARASRYPLLEA

SLGKRVLITPNAKPGKEVILSLVKAVHGQAVERMGRSVSNIDDLLVISCEEDFEVCVPLL

EKGIAIYSSELLLNGIITQRLEYERYRLFVAHVKKTRTTLWLKKGGEQFLRVTKTK

>Lactucasativa_BCP4

MGDDAKGDSKSSESDSSTWDFGGEETQIVDTQCFDSPTSSPGNKCNGDDTEQMHILQSTI

PFDDTAVLEDDLATQLMDPDDETQVVNFYDDTQVVNLETEIEEMDILDSLERNATQLFND

SDTEELVDTDLEDTEKIEVVDDSDEDSSRRDCKHTQHTSGGNFRNFTSIRAASIRASGLA

ARNKASQRKKLNDQPETGSRCGFGRSTARKLFAEETLPETKEGNDNNDLCGKANLPLETD

LAGLNYLDSQEPGEASQQNALDFVDNFLKVNIECSDKCETNKSSERKSKPVLSAKGIQTL

AKSSNLINAVGEIEIFEWDNTREDEAGGEFIRRKRESLFAYGGRKLKSSSFSKGGRKLEE

SKGKKQMHSHVIVSGLASSDSKLVLTNRKLTDVQETTDVRFDTQMAAEAMEDLCFGLHTN

SDDSTKEGENGTNKQKGSHKSHKAPPLTNGVRTRQSKLKRSDENERETHGTVPLKEPKRA

KSAAKKNVSSDVGKRGKLTLKRKEVDTEEGQLTSVDQIPVKKQCIQSVIPVARRTRQSMS

DNKSEKVKDASNNLTEEINIFHPKGKRTSRNLSSVSRLTRSTAAEGGKESPGVEGSERET

IEPIATTCTTPLNHATPLKESSPICMGDEYLKQSWKKSSLRSSIIQEPDSLTPTRVEFSS

PMKDLRKRRDLSMIHVLFSRHLDADIVKQQKKILSRIHASEASSMSEATHFIADEFTRTR

NMLEAIALGKPVVTHLWLESCRQSHSHIHEKNFILRDFKKETELGFNLPSSLTRARKHPL

FKGHRVLITPNTKPGKEILASLVKAVGGVVVERLGRTAVKDDKVPEGLILSCEEDYALCL

PFLEKGAAVYSSELLLNGIVTQRLEYERHRLFLDNMKRTRSKNWLNKPKCEFVYCFGLHD

AMLFGRIKE

>Dcarota_BCP4

MGSIEDDDNDDKTQVGDDFSDKDTQFMDTQSFLDGSGSFDNTVPFEDAETQVVCLAGETQ

VLDFDCETQAVDNLDCTENLCTQLYNDFDMEVVGDTDDEGTKKTELVSDTEELSDVDSLK

ELGSHPGEPENILQTDVHKQSGGACKDIPVTKECNTGPVLRGFTSLRVASVRSAGLAARD

MGLRRTKGASYSSVSDNLHEAGHSGRSVVRDLYDFDKRFNLEEHDLQMKGLTSENRCRAG

KSAMRKLFNDDISREKGVNEYLNTDKGEMLPPVCTMENDLAGLSYVDSQEPGEASQTIAL

DFVDKFLKVNVTDFDEESDVIKSTGGKSKVLSGAKGTQCFAKNANRNYLASSGIYDWDDN

QEDEGGGEFYRKKKHSFFDDGCRGHKSFTQPRDSKHHNVKRRQAVKVDSGKVVQQDSSSN

VPELFYSDSKLLLHKNKENAKLMKVAKQSIGKNLIKDLDEVQTGAARDNLDMLGIGADTQ

LAAEAMETLCCGVGMTDCNSDVDNQDAEIHFSSSAERKLPKNSESDKGFLHKKASRALKS

RVSTIQSNQSKRSSRRLTKGSSIVSELESMKTRNQWDEAGDLCNLHCGIVEENLNKTELE

ECGISETERCHTAASACQKRVKKRSIEEHFSTFSPIAFRTRQKMSNQNQRDTYTSTDLRE

KMDSLAGSNASKKKRRTVSNADARGKSSELKFIQPSNSKETKETHTEQNDTERCGISTAQ

KEKPCQKLPKEKINGKSGAKSKILQVNSGIKRTTRSSVRNPASPVMDRQCGNILAKDTSV

VNNPIEQIGNATSKGSTVLLTPIQCKTPVNEASPICMGDEYHKQSCRKNLLRSPLKKNSS

RISADGPESAYACEYLQKKRNITQLQVLFSKHMDEDIIKQQKKVLTRLGVSEASSISDAT

HFVTDKFVRTRNMLEAIAKGKPVVTHFWLVSCGQAGCLVDEQNYILRDAKKEKELGFSLP

VSLARACQHPLLQGCKVFLTRNTKPGKDILAGLVKAVHGVVVERIGRSALRDERILSDLI

ILSCEEDYADCVPFLEKGASVYSSELLLNGIVIQKLEYQRYRLFAENIRKTRSTVWLRKD

GELIPVAKCK

>Cpapaya_BCP4

MRDSFTRTGETFNLPDDDNFYVHDTIPFEDNVSVEDAIDTQVMDFAGETQMLNLAGETQL

LNLPGETQVLVDLDYVETQYLDGFHDEIIPNSDGEGSDETEVLDDNEMAFDDGSSRRGNG

LSSDGEKIQHTSPCEDDEKETPKQSDSLMNGRKSTGSAACFTSVRTASCHASGLAARNAA

SKGTSSGSFSVLTNCYSSDQCTMNSKRLESKAGEEVDQLPHFGGCIETIKGLKIGTKTVR

KLFTDDSLMQNERSPCNNNNIGGGDNTLQFPSYDGELGGLSYVDSQEPGEASQANALNFI

EQFINDHCTELDKEVDNGKAAGGKSEHLSSAKGAQNFAKNRSTRTTVAATGIFDWDDNCE

DEGGGDFFCRRKEDLLGFRDRAWKSISLPKKPKGKGLSELRDKKELLNDHGKIMAYSDSR

LNLHKVKESEKSVQAVGKKSKRNLVSDLDEQFNTDPSMGLSSGNATKADMPEMPGVGVDT

QMAAEAMEALFYGEGMVSINDNDVHQCLQNKVGQECSLIGKQLSSGEQLCPSDIVGPRNC

KRTKRTNALLMEDSSVHKRPKNSVDCGTELIITKSKRTRSNAEARFPVKCSENMKKMPTK

VTKKRKKVGASESSRHESPSELSGHSSVKNQHIQEDSPIAHRTRHSMMQNQLEKDGNVSS

YPRRRRSCRNLSGKITKYDNLGTQHKLSVLPEEIRQDIGGCERSVRNGISSNIDSTMRRK

MQLSQDARPELLQQRKESLEKENSGDAACNCSTVNGQTISTALTETEVLKHSKRSNLDSL

PSEEKTEANSRIQRSVRGNSEVSNLVCNTPDNSFTKTTQVNSMMPVSTASPICAGEEYFR

KSCEKRLSGSRLLKEVTGLTEPLALPSSKESRKRRNLSNLRVLFSHHLDEDVIKQQKKIL

ARLGISLASSIMDATHFIADTFVRTRNMLEAIAAGKPIVTPLWIESIGQVNIYIDEESYI

LRDTKKEKDLGFRMPASLACARQCPLLRGRKVLVTPNMKPGKETISTLVRAVEGLVVERI

ARSALNDDKV

>Klaxyflora_BCP4

MLSLNLNYKWFGVPSTSPVKPLCHSLLPSQTLTSHFRPLPVIDRTEDMASFATDGFRFTH

TENRRSLRNCASNNRFTAQARTSPPPLPPPLLSGETVACEEDGIETQIEDLEGETQVLDG

GREMCVRDQVLMANLEGDTQVVDDLRDGLEMGVGNVGGETQVWDDFGGQTQVDDAEYGYD

TELINLGAETEVQDDEDCVRNVCSQFLRSGIDFDLAGAGGTSNESGIVKGVFCDSSTSLV

NEKKQGQSYGKPLDQDGNYVSATPPTTVDLDLGSFQRSFTSIRTASLRASGLAARCKALV

ETGISAPHAPVQNDYEISSVGHTPRSAHNHDIAEQGVNNDASMCKPVCSVVRKLFNEEPL

AENRNATNEGDGEEGANTPQLPLPDNDDEVAGLSYVGSQEPGDSTQAEAFDFIERFVNDN

ASDMLLEADNIPERRTISKPTSSAKSFLKFSKKSTVLRQPVDQAVSFNWDEELEDEGGGD

FFCRMKEKIFNGGHKQRSLPQPQKSRRCAELVGDKSDKMKFCDKRTVTQSDSKLLLFNLD

RNSKVLDTAKANIRRNLETELNGELPIRCTEEPTEAIARRNDIQAMFSVGPDTQIAAEAM

EALFGGEPISSVDADADANQWNSASLSAVDSKSRKSAKIREFREKASSDGSLIQKSQRKI

NEKKKKKIHGTILNGHGKDEVNGTHTPNRSCVIDKANPFISGRRTTRSMVQNQGNMLLNQ

AVVAQNISKDMNNETNLQIGVAIPKGKRSRNCVRTEAKYDKTHSDVVQVKLDDSVLHESR

PNVKTSGAMTSSRRQSARHLRSHLVKSQDYRKLKSKKEANKTSSNLCCNEASSSKRFVTS

PARASCSFDAADIYRDVVRTNILDNHSAKGTAVDAADCGMPVEVVGAVPITDKPSQRSSN

PPESACAISDKHSEIRNARAGNRCRRQLPINLSRSSLRKEISALLSNSPNSLSDLKRKRR

EMNEVRILFSNHLDDDALRHQKKILARLGASLAFSISDATHFVADSFLRTRNMLEAMALG

KPVVTHLWLESCNQTSCLIDEKAYILRDAKKERELGFSMPDSLVRASQKPLLQNQKVIIT

PNSKPSKDIVANLVKAVHGEVVENFDLSSLKVDEYPGSVLILSCQEDYVFCQSYLNEGVA

VHSSELLLSGIVTQRLDYARHRLFEDHVRTAVARRLRSARDCR

>Ahypochondriacus_BCP4

YKYRFRFSLMASRRGSQPGVRKVTASDDQHFDDTVPNEDDALINNDVFETQVMDFDFETQ

PIELDNETQILELAGETQLMDECNLFEHMDTQVLDVLDNEDWECTSDRRKDEDEETQLIE

DLDNAELQDTGGTQITPLGENRNFCFRHGDVDGVKEESLPLDTVAPCSGSLKRGFPSVRT

ASLRKAGLAALEKASKNNESSSTNFSSLSSELHPSENHQEISNVKKVSLEIDLSDYNDKS

LLDGKKSNVARPLARYLYVEEDDAETESDDGVRNRREDFSHIPCDNDIVGLSYVDSQEPG

ELSQANALEFVDKFLKGNLFESDQGEKCRSAAGELGNPGSIAKAAKSANEGGGEFFTKTK

EVLLGHKSCPKPQSSRHHEPSGSQSLGKLRSEKELRNLHETTRGCPDSDLKLVKGGSKGK

VQKMDIVKKISSGQQLAALPTIQSVDMQNVGIETQMAAEAMEELSAGIALLKNNSGVSDQ

GCQSNTGQNQKEAIKSGPSKQGSRKKRTYIFTSGVVTRSRIKDMLMKTRQQSKCYFTPET

RGQSVIEKFNAGPVKPKKKQNKLSLNNTLAESKTESSNTGRVIVGDKRRQSALEGQLTND

SPQPSKRQSLRGKVKPAVQEAVQRMNNADDSACNPRETHLGGGVDIGIEFTTRRTRSKRR

SSLQCAQCPKDREDLLSGAMSSFEDTHRLPCKKKDACSPPAKGTLGSPTVDGTPENLQKP

STREEITPVGFKTPSAVSPICMGDGYHTPSCKKKILLKHEVTGLIASDDRYSSPFKGLRQ

RRDMNNVCVLFSQHLDDDIVRHQKKVLTRLGVAVASTISEATHFVADSFTRTRNMLEAIA

NAKPVVTPLWLQSCGQANCFIDEKNYILRDAKKEKELNFSMPKSLAHARKEVISGLVKAV

HGLAIERLGRSMSSDEKLIDNLLVLSCEEDFSVCVPLLEKGIAIYNSELLLNGIITQRLE

YERYRLFVSQVKKTRSTLWLKKGAEQFIPVTKTK

>Hannus_BCP4

MTPINQPNPIAALGILAVKIRRCLIASLLNLHLHHSTCNRGNGDTEQLHYIQNTVPFDDT

VMCGDDLATQVMDHDGETQVMDLDGETQLVNLCGETQVVDIGDDVMDMFDSVERDETQLF

DGYDTEEVVVSDHDGSGKTEIVDDSDEASDDGSGRRECVNSADVERAKDLHQCKEDMKDC

KSNTDCERSEKHTSGSIGFKSFTSIRAASMRAAGLAALNKASKRSNTPSHPTLNSEPDIE

HRRVLSGESQSHDLEKVNQNRCEFGRSTARKLFDEDTLAETGTKEVNDKIDLGGAANSPV

LDSELGQLSYIDSQEPGDASQANALNFVDNFLKVNIECDDGFGIGRSTGGKSKPVSVSSA

KGAQTFAKSASLIDAVDKRQAFCWDDNVEDEGGGEFFRKKKEVFFASGSRKLKSSVLSKS

SRKLRQSDTKLASGKNKENHKAKKSLGTVIKELTLRKNLIKELDNVSTDKPMPSGTDIRR

TIQETADVRFDTQMAAEAMEDLCFGLQETDPDKSQPLPLSNDGVRTTRSKSKMSSDRSKG

VTRTKLAAKTRENFDNVAFDGAKRTGKMPRKRRELDSVDAPVSSADQMSVKKQCARTTRQ

SVKNNEPNKEVNILTGFYPKGKRTSRRLSSTRKEISTQSISRLTRSKATTLTKQGKGSMR

ERVEQNLSVCTTPVSHGTPLKESSPICMGDEYLKQSCRRPSLMKEVSDLFITNSSFTSPS

KETRQRRDVSMIRALFSRHLDGDIIKQQKKILSRLGASESSSMSDATHFIADSFTRTRNM

LEAIALGKPIVTHLWLESCGQACCHIDEKKFILRDAKKEKELGFSIPSSLARASQHPLLK

GHRVLITPNTKPGKEILSCLVKAVHGMVVERLGRSLLNDDKVPEGLLILSCEEDYAVSLP

FLEKGIAIYSSELLLNGIVTQRLEYERHRLFLDHVKRTRSTIWLKKDDNQYKPVGKVK

>Carietinum_BCP4

MAKNEDHHRIHSNTSLNSDFNHFDTQPFDDSSGDDDDDDECRYFEDTVPLDDDDEELETQ

VVNVDDETQVLEIAGETQLLDDFDTELLEEEIESDGTHVLENVDDEVSDDDPQCRDSGQS

ADPSNRERGRDEKETGSGSMPPRFTFIRAESLREAGLAKRNMNLKHTQDQSNSVMGMNQF

CQEPLAVESKGKSFLGCSEKVREVDQEFNHDFSRNAVRKLFNDDLPGETNGPSLSNNDFN

EGESLGKFPDYHGELERLSYINSQEPGELSQINALDCVDRFLKSNFMELNQENNCVKKLE

KKSESLPRIKGQQSLSKIINDRSKAKKTEIFDWDDNCEDEGGGELYRRRKDDFVEGGTRR

PRSLPGCRKNKSCRSNGDKEEEEQSSIPIKRKNAAHSESRLGMHNLKIRDDNIQEPTRKL

ERNLANEMDEQFNGNFSRGELDPNGNADRREMLDVGLDTQMAAEAMEALFNAGDIVDHVA

NDSIRITRSRSTYQLNDSSTGKMGLVTPKEHTGKYDRKRKADVKSDLQTSGLSKKYTKKV

GQCRKGNVTSRSQKRKLIVEGNQTTGANKSGRIVSSPIGEQRKSAEALKNHQLNELNNLD

SNDGGGTVNEKQFQGEVFHLTPIARRTRQSLAGNKMINCDKSLKSLSEKAMRIDPHEKCR

GVGLQASEVLAPKSTLGSSDHSPVDDNTELCQHEKLASKENAVGVSNDFAVDMFDYPRRR

RSLRIMKLPHHDKDSEKSVGSSKSVEHNENIGKSTSVKKKTRTSAVVKSHVNCHTEKANL

ENANSGGIPICCDNLDENDANLNSNVKNNADARLSSNHLEFTISDESPRDRYKSPDLATT

TPSNCKTPVNNASPVCMSDDYYKQSRNRNVSRSCLLKVFRKDLQRELRSLSAIRPELITP

SKDSRKRKDMTDVRILYSHHLDEDIIKHQKKILARLGVSVASSIVDATHFITDQFVRTRN

MLEAIASGKPVVTHLWIESCGQANCFIDERNYILRDAKKEKEFGFSMPVSLARASRHPLL

EGRRVLITPNIKPSKEIISSLVMAVHGQAVERVGRSALKDPKIPDDLLILSCEEDYASCV

PFLEKGAMVYSSELLLNGIVTQKLEYERHRLFADHVRKTRSTIWLKRDDRKFTPVVKSS

>Csativus_BCP4

MKTRDAWPSVIIDKDKEKSSLKDSHVDRHNGLGQSSVNDGDSGNVKCRVGSSAVRKLFTD

DYTPVGDFGDLPTKLDASDVDLHQLTACDGDGDQLAGLSYVDSQEPGDLTQDNALDFVEK

FLKDNSMEFGLGVGMHKRNAMVQPKSVPNPRGQYNLASIVNCVRVVGESRVFDWDDNRED

EGGGDIFRRRKEEFLTEPRKSKGRKLDLSGDKEASMSNQNMKSRLFCSDSRLELRKGKGN

NEPSRESNIECKRNLSYKLDKENDGDPCRGELQNNGIQPDQLEEANVGFDTQMAAEAMEA

LFNDANIHELVYNETNQHLENGSTDSFRGSPSRKSYSSSKLRRSSRGHASSSEVAPMQSK

IRNQKFSGVITKACGDEIVKLSNRSKKRDADAINGNENIGYDLKNACNKVQKQQNACNKV

QKRLLRGKVVEVSPVACRTRHSIIVNQSKKAKIASSGCERSAAKVGSFIKKSSGDRGTRD

FEAKRTKSLEAASKTLKMKSKGAKNDAKRSIGERGLCDMLAGEASLPGDLLGQTMNRRKR

SCNVKKTRASLCLLSPPSNKNLKRPTVSRTGAEKAHGGTITADTNDQLSIEDSNRPNSVQ

QLNKKNDGCSVSSVVKTTPDESPSKRHKPSVTVCTSPSDNSMTPINSVSPVCMGSEYYKQ

SCKKNLSKSSLLKELRDLTSSGFVSRSCPTESRKRKDMTDVRVLYSQHLDEGIIKQQKKT

LTRLGVTVVSSMAEATHFIADKFVRTRNMLEAIALGKLVVTHLWIDSCGQASCFIDEKNH

ILRDTKKEKEVGFSMPGSLACARQRPLLEGRRVLITPNTKPGIAIISSLVKVVKGQAVER

IGRSMLKDDQIPDDLLVLSCEEDYNTCLPFLEKGAAVYSSELLLNGIVTQKLEFERHRIF

VDHVKRTRSTIWLKKDGNKFQP

>Csinensis_BCP4

MASKEISIDSCFGQSADLSPDQDAVRNDGSEPKVVEEIDNIHDLKDNETEKGLRNGNSCR

VGSSTVRKLFTEDSVSQDKGLPNNGDNAAGGENLLQFPVNDDELAGLSYVDSQEPGEFSE

ANALTFVEQFIEKNNFVDFDHEVDLGKSKGGKSKPVSTAKGPQSLAKKSNDRSKAGKTGI

YDWDDSREDEGGGDLFCRRKDEFFGTAYRAQRSLTEPRQLKKRKFDLDGNGEQQDVHDGI

TMRSDSRVTFNNIKKNEKKAEEAQLIVEKNLLSELDERLNADSSRGQPDAAVGKTDVSQI

LNVGPDTQLAAEAMEALLSGEGIANHDANCLQSNSKRSAEGSSRGKSKSRVSLKQSSSQK

RVRLSGVGVATRQKRKSKSGTEISRCSPDNSVKNFKNISEKCDRELATLNKRRAKSMDEQ

NSTINGSKNMDRVSSGIIGQRNEDGSLESCQPKEFNRCLRTETQNADHSIKKQKIAKAPS

ASTPIAFRTRSSKAVIQLKTTGQILDYCIHDANHLMEVGAFEENVTCNKDVEASEVMHLK

KKHSKLSSNQFGELKSTKPSQPEKLDLELTAMNNGVDGLRYPRGRRSRRNLSVQVSGCSA

GMNVKVKSKDFKGSKTPNHSDGKIVVDSQPSAENAEMNSRLDKSPREQCEALESPCTSPA

NCITPVNAASPVCMGNGYIKQSCRKNLAKSCLVKEINRLIATEPEPLSPLKDLRKRRDMA

SIQVLFSHHLDEDIIKQQKKILDRLGASEVSSITDATHFVTDIFVRTRNMLEAIASGKPV

VTHLWLESIAQVKIHIDEESYLLRDTKKEKEFGFSMPASLARARKHPLLKDQRVLITPNI

KPSKETISSLIKSVHGQAVERLGRSALKDDKLPDDLLILSCEEDYEICEPFLEKGAAVYS

SELLLNGMVTQKLEYERHRLFVDNVKRTRSTIWLRKDGHKFHPVTKLR

>Lusitatissimum_BCP4

MVSTADDGGGAVRPVEDNSAAADTQPFNSDWFASSSPGENVGYDDDVFVDTRKVSFDDTV

PVEDIFETQVVNYGEETQAPDDLDFVGSMATQMIDEFSEHVVLDSEGECSDTTEILGTGD

EVSDDDGEALAEVEGQFPSYDNSKDEQSVDQTNASSNPQSNSGLVPRFTSVRIKALRSSA

LVAQGNALEDSGNGFCSNLNCSHSTEQLTSCDEIVKDSNAGENIDKEKEPDCGIRSKVNS

STVRRLFTVDSISKDVDISHTSSITGAKEIIQPLTVNEELAGLSYVDLQEPGDASQADAL

AFVERLIDETKTSFDDELSLGSCGGRKFSCIPTAKGPQSLAKKTIKRSIGGTTGIFDWDD

RLEDEGGGDIFIRRKDDFLGVKEPGKSFTDPLKSRSNMRTVCRGNKKKLEGAIHSDSKVV

VRKFKVNENKKEGTLRNTRKDLLDGFHEQANRNLTEPTGQPEVAVAPENCDVGLDTQLAA

DAVEILLNADPLSDHDGSDAKEKHYALQYPTRKSKRTASSKLCASDDSDDSGVNTRQLKK

RKNDVIKAVQTSVSSPKQSNKVVLERDAGPVLMTRRRVISDGVELSISRGKKSIGMVRRV

RPDADEYSIPHDQKTLKVLPSKTSEPKYREATLGLQLDSSNNCKVPSLRRGRSAKRKSSP

EEVAVHTTIARRTRKSFVVSPLVGNEPAEVDEHKDHTKDENGLDQSRADTVDEETVEASN

AVRLTSSKSEKPVPVTPEKMLKGVIEASISASTSPVGSVTTNAASPVCIGNGLGKNNLAT

TSLREINNLCSIDLEPIPEVKDSRKRRNLTDVRVIFSRHLKEDVFKRQKKLLDRLKVSMA

SSILDATHFVTDKFVRTRNMLEAIAAGKPVVTPLWLEHVARANYYIDEQKYILRDAKKEK

EIGFNMEVSLAHARQYALLQGRKVLITQNAKPSKEILFSLVKAVHGEPIERVGRSALKDD

SLIKDLLVLSCDEDYEVCVPLLERGAAAHVSELLLNGIVTRKLEYDRHRLFSDHVKRTRS

TIRLRKDGEKNFTPVVKNK

>Mguttatus_BCP4

MGPLEDEDNRNDNNSRKSNLNVDSSYVATQQIDSQFSPGDVSEGDEDEDFLCLNNTVPVD

DTYLLEDAFETQLMDLAGETQAVDLAGETQAVDLAGETQVLDDLDCMNDTSMEFLNEFSK

SQGANKTQALCETQVLSQDDSVKTNGSGLVGSESKIDIYPPKQGLVFRGFTSIRAASIRA

SGLAARARGANANLCTTSCEKSSVEQQTCEQSGSSGVGYLLKSDRKNDQECLQNEYNEEV

GGLKNLNKYKVGNTAVRKLFGEDKVSEVGQLEADFDHSDDNLDMPELLGNENCMAGLSYV

DSQEPGELSQAHALEVVDKFLDLNVVEDDEVFGMRVHKVEKKAKVVSGAKGSVDLAKKSI

AKIADGKCGIYDWDDTREDDCGGEFFLKKKELFFDKGCPKQRSLTEPRKPKCNDLGSVKT

VGNNGDEKEQKYAKNKLGDPVYSDSGLILNNNVRAKRKSLNCGERVLQKNLIKDLDEELS

ENDANEDVPKTVDIGPDTQLAAEAMENLCFEVQLPESNSNVPNKVAHITKGASKKRSYIA

SVGVLTRQAKQLKRASIGASNEHSPTPELPKRTRKRRNTMPLETESTVQMSEKKRHFEDQ

LGFAVPVAHRTRKCTKLNRPKATAVNSSINGSTSALIVRKGTAGKDKNAEMLTAEKQYSA

RSKGSRENGASISLGPVNNGSSKNDSAQRDSDCTNVKADLAETSGRIDALSREKRGTSLS

TCVTPANCTTPIKDLSPICRGDEYKTQSCRRNLSRLSLITVIDNSVTGSPLLYGGIKGSR

KRKDITDIRVLFSQHLDLDIVNKQKKILARLGGAVASSMTDGTHFVADEFVRTRNMLEAI

ALGKPVVTHLWLDSCGQASCLIDEKNYILRDAKKEKEFGFSLPVSLSRACQQPLLQGQKV

LVTPNTKPGTHILASLIKAVHGLAVERLGRSVLKDEKLPEDLLILSCEEDYDTCVPFLEK

GGAVYSSELLLNGIVKQKLEYERHRLFEDHVKRTRSTIWVKRKNQYLPVSKCK

>Mtruncatula_BCP4

MAKDEEHRIHANTSDTQPFDDDSSSHSSQYEDDKENRFFEDTVPFDDDETQAVDLGDETE

VFDDIAGETQKFDDFDTELLGEGYESDGTEVLEDVDDEGVDDHQCRDSGGSADREDDVNR

SFNERSSDEKHTSSGSMPLRFTFLRAESLREVGLAKRSMNSKHTEDQPNSVMGMNQFCQE

PHAVKNKGESFLGSSEKVREADQEVNHEKHNVEIEGFKSGSMSNSARTTVRKLSYDDLPV

ETNEASLSNDDFNKGDSLDKLPDYHGELERLSYVNSQEPGELSQLNALDCIDRFINSNIM

ELDEETTHVTNKEKKSEPLPCIKGPQSLSKKINDKTRAKQTEIYDWDDNHEDESGGGIYL

RRKEYFFEDGTHRPRSLPGCRKIKSRRPKGDEEEEEQSSIPVKRKTAARSESRLGMHNLK

IRDDNIQGATRKLERNIADELDEQVDANCSRGEMGPNSNEVGHEMLDVGVDTQIAAEAME

ALYNTVEVVDHVTNDATRVTRSRSSYQLNNSSTGKMGPVTPKEHTGKYDRKRKVDVKSVL

QTSGLSKKCTKKVGQCEKSNVVSRSKKSKLNAEGNQTSGANENGRIVSSPVGERRKSAKA

LKRHQLGDLNNLKSNDGGSTVNEKQFHGDDFHCTPIARRTRRSLAVDTSLKSLREGASRI

DPHEKSSGAALQAAKGLGPESTLGSSDHFAVDDTAELCQQEKFASKENIGNGVAVDTLDY

PRRRRSLRINKFSNHDEGSENLAGSSKSFKQTEDIGKGSSKSFQQTEVIEKSTTRKRKMR

TRSVVKSHVNNPSSSSSCGGLVVPSEDQMQRKNLELNLNSNVKNNADVWLSNKNLKVAIP

NESPRDGYKSPDLATTSPANCKTPVNNASPVCMGDDYFKKSCNRNLSKSCLHKVFRKDLL

KEMRSLSASRPELITPSKDSRKRKDMSDVRILYSRHLDEDIIKHQKKILARLGVSVASSV

ADATHFITDQFVRTRNMLEAIAFGKPVVTHLWIESCGQANCFMDEKNYILRDAKKEKEFG

FSMPVSLARASKHPLLEGRRVLITPNTKPSKEIISSLVSAVHGQAVERVGRSALKDHKIP

DDLLILSCEEDYASCVPFLEKGAMVYSSELLLNGIVTQKLEYERHLLFADHVKKTRSTVW

LKRDNRKFTPVTKCN

>Ppersica_BCP4

MGSLWDENDEIKPMETNPVVDCAHTQPIDSQISSPPSPDEKAKFEDAHEIVQDTVPFDDT

VLVEDAFETQVLNLAGETQVMDFGAETQVMDFGGETQVMDFGGETQVMDFGGETQVLDDI

NCVENMETQLLEFEDEVVSDTDSEESDTTEVFDDNKHLTHDESVRRGSGQVVNEEKICCT

PFENNVKGLMEQANNSIHEKQNAGLHVSTETPVDKSSPELKPGSVHMHFTSVRAASLRAS

GLAARLKGTNSESPSVPSNSQCLEPLSGKDNAVSLLWGSTIGGEKVNQEHDMGRCNEKIR

RSTNENNCRIGNSTARKLFNEDSDDEEKGFPHNSSSGEEGEGLLQFPCNLAGLSYIDSQE

PGELSQANALDFVDKFLQVNVEEFDKEVDRGTCAGENSKFVSSAKGPQRLAKKAIDKSIV

QNVGIFDWDDSRENEEGGDFFCRRKTDFFGGGSHGWRSLPQAQKSKGNRQEGQKDHKKQV

QGKKKKMGVIHSDSKLLLHNSKFDKKTEHEDEIKHKKNLASEFDKQFDINSPRGQLDANV

KKNNDPEMLDVGIDTQMAAEAIEALFNGEGISNCDAISALPDVQGNPKSSPEGSMGEKTK

NTLSSKKPSSRKRVLLSDAGVASRESRQAKKTRIGAKSGKHFSISSPEYSKTARKKCETE

LVITKSKKAKSNAKKHLNINGNKSLEKIPSVAIDLRTEGSIKRHLPDVGNFVPVGRQTRQ

SMVVNQLQKADKVSSDCGEESSYQTQNVAIREKIISFTGVQKSKVLNAKSSKLGSNKTGK

VGNTKPSQQEESDFKFEAVSNGIKLDVLNFPKRRRSRRNMSIQVYGPNNSDGPSEPSVQA

DKIGQRVNSHKRLQSGAKNICNDIKLTRRTRSSTCGDQNLDGKFAREILKGGPGEAPLHC

NSSHKDGRMISEIITGKRVVGISDRKSDANCSSATKMSDEFPRENCKPSDSSCTTPVNNK

VPVNAASPVCMGNEYFKQTCKRRLLGSSLLKEIRGLSATVCEPTSTPELRKRRDMTDVRV

LYSHHLDEDIIKKQKKILARLGVSVALSMTDATHFIADQFVRTRNMLEAIAFGKPVVTHL

WLESCGQAGCFVDEKSHILRDNKKEKEFGFSMPASLARACQHPLLQDRKVFITPNTKPGK

EIISNLVKAVKGQAVERIGRSTLNADKIPDDLLVLSCEEDYEICVPLLEKGAAVYSSELL

LNGIVTQKLEFERHLLFSDQVKKTRSTIWLKKDGNKFLPVTKNK

>Acoerulea_BCP4

MGSIEYNDDEERNSTKGKFFSSNSNNNTEIPTQDLDSESPLRSSEIDCTVPCTETVQIER

VTQVLEDLDFDEIAGTQVADSEDEGTDKTEVLSEIEGGTDDDSEKRKKLVVEDGKKTVPS

INTGLHASKAGHCGTGRAYRSFMSVRAESLRAAGLAAARKLNSIGIVSGGRTNEGINQSG

KDLVCEDNEVIAINELASNHMQGRDADRDSGALNCNDIVEKTRDKTKCSMARSTVRRLFT

EDEPDSDSSLHELNANDHELAGLSYVESQEPGELSQANALNIVDKLIAVNIEDFPLETNW

GTTIKANTPPVSSAKRVQMLAQGAIRGSPVGKADIFDWDDSHEDEGGGNLFSKRKEELLG

GSFHMRRSYTQPKNSRPVNAKKCRGRVGKLGEKEEDSKLRQKLMGLTRSDSRLVVRKLKE

NEKIQQVCNIRTRKNLSEELDEQMNPEVTTNGSEGCTRDGTRNKSCSKKVHLQTRTSVSG

GVVTRSKRVNTLDTNISGETSQKNPADQEMNGLVSDSMATGRIRKVNGRTNSKKRNAANQ

NKTSDRISSESIDQANNLTAMNQSHVNGSDIFRGPSNKILSVTNEQMQEEHRTFTPVAHR

TRKSIAGNPLQGSELLCRGIANDRNDVVIGGKRKRRSLDVDEQRPSNGGGKRSRLHSDLV

SKDNSIQQEQLPCASHENIFKTNNEKRDDNIGALSTAHKVEADTRPKKLKDKVRKNGVKL

DQKRVTHSPLHCNSSLLSSEKKSEENLISKPLRKAVVTEAVVICTSALVNEKVFRKDDFN

DTSSAFPGKGYCKDHLSPVKAAQTSMLDTSPAGKVKQSVAACVTPSSTCTTPRSDASPVC

IGNGYHKKSLSRSPLTRELNRLDANEVLHSPASLDTRKRKDLTNVRVLFSHHLDEDIIRQ

QKKILARLGAAVAGSSSEATHFVTDKFMRTRNMLEAIACGKPVVTHLWLESCGQASCFID

EKKYILRDPKKEKEIKFSMPVSLARATQSPLLEGKRVFITPSIKPSKELIASLVRAVHGQ

AVERLGRSAAKDDKMPDDLLVISCEEDYAVCVPLLEKGAVIYGSELILNGIVIQKLEYGR

HRLFTDHVRSTRSTIWLRKEDGDQFVPVAKRK

>Tpratense_BCP4

MANHEDSQDFNHCDTQPFDSSSQHSSEADDDDDKENRYFEDTVPFDDELEIQAVDLADDT

EVLDIADDTEVLDIGDETQKLDDFDTQLLDEGYESDGTEILEDVDDDGVDDLQRWESGQS

ADCEEVVGRLINERGDDEKQTSSGSVPPRFTFLRAESMREAALAKRNMDLKHTEEQSNSV

TDMNQFCQEPLAVKNRESVLGCSEKVREIDQEFNHGKNNVEIERSENESMPNFARTTVRR

LSYDDLPVETNAPPLSNNDFNEGDCLDKFPDYHGELERLSYVNSQEPGELSQINALDCVD

RFLQCNLMEVTEETIYVKNMEKKSESLPCIKGELSLSKKINDRSKAKQTRIYDWDDDCED

EGGGDIYQRRKRDFFDGGTQRPRSLPGCRKIKSRRPKGDKEDEEQPSIPMKKKTAAHSES

RLGMHNLKIRDDNIKEGRRKLARNIANELDEQSNANCSRGEMGPNGNEDGQEMLDVGIDT

QIAAEAMEALFNTVEVVDHVANDTTRVTRSRSAYQLNNSSTGKTGPVKPKDHAGKYDKKR

KFDVKSDLQASGLSKKCTKRVGQCENGNVMNKSKKSKLNAEGNQTSGARRIVSSPIGDRT

KSAEALKRHQLDELNNLKSNDGRSTVNEEQFQGNVFHCTPIARRTRRSLAVNESIKSDIS

CKRLREGAMGTDPHEKSSGVGLQASKGLGPKSTSGSSDHFAVDNTTEVCQQEKLASKENV

VSVSSGVTVDTLNYPRRRRSLRIMKISNHDEGSENLVGSSKSLKQTEDTGKGSSKSFEQT

EDIGKSTTGKRKMRTRSVVKSHANNHSPSSSSGGLVVPSDDQMQKKPSKSNLNSNIKNST

DVLLSTKNLEVTIPDESPRDGYKSPDMATTSPANFKTPVKNASPVCMGDDYFKQSCNKNL

SRSCLLKVYRKDLHRELCSLSTIRPELITPSKDSRKRKDMTDVRVLYSRHLDEDIIKHQK

KILARLGVSVASSIADATHFITDQFVRTRNMLEAIAFGKPVVTHLWIESCGQANCFIDER

NYILRDAKKEKEFGFSMPVSLARASQHPLLEGRRVLITPSTKPSKEIISSLVMAVHGQAV

ERVSRSAMKDHKIPDDLLILSCEEDYSSCVPFLEKGAVVYSSELLLNGIVTQKLEYERHR

LFVDNVKKTRSTIWLKRDDKFTPVTKCN

>Cquinoa_BCP4

MELSGETDVIDDDGGDFGEHMDTQLLEVSDDNDGSECNSGRRGIEKAETSGGAVGSIDVE

LQDRSGNQTAGFVEKETSNCRREDGGDRSESMFSGSAERGFASVRIATLRDAGLAARQKN

ADNGSAEIHCFDDRGRSSNVERAGREKDMEISEEEGSSGGEKSSVVRSLARKLFREEDVA

DLESDYNISDSDRKDDVCKNDVAGLSYVDSQEPGELSQTTALEFVDRFLKVNVFESDESL

LRKNAVAEYKSPGAIGKGAQSVAQQARRKSILAEASIYEWDDNLEDEGGGEFFIKKKDML

YGTPKSCPKPQSSRHREIRENKSLGKLRNEKKKQNLHNSVTGLLSSDLKTVNGGEKGKVK

EMHSEKKVYNCSGQELGLGSCSKEESAGVQEASAGVHDPQSTDIHNVGVDTQMAAEAMEA

LAAVTELVNNDDNVVDLGCHSEEGQTQKEAMKNDPSKQCSLQKRAWFSTSVVVTRSRSKR

TIKTRGRSTMGKFDVDLVNSKVKQNRSSMKNFSTVDRESSDGGAVSVGGKQPKSNFEGQL

INNPPQPLKRRSLRGRASTFTPVVQQAAQSIMHHHKKRKTRNNAVSENMDDAFGDSGERH

CLDLVHERKRKTRNNAVSENLNNRNDSALANSNKVDGLNCRTGHIQRASNEDQQCGDKGY

TFSADRGPEFTMGFIARRTRSRSRSSTTQRAGSTLDEIGLFSSGMLSSRNTRQFSSKKQD

ASSPLAKGTLASATIDGSPVNLPKLSIPTDSTPVNLKTPRTSVSPICMGDGYHTPSCKKS

LSLKKEVSGLIASEQRTSSPFKGLRQRREMHNVRVLFSQHLDDDIIKQQKKILSRFSVSV

ASLISEATHFVADRFVRTKNMLEAIAYAKPVVTHLWLESCGQANCFIDEKNYILRDAKKE

KELGFNLPRSLAHASRYPLLEGKRVLITPNAKPGKEVISSLVKAVHGQAIERMGRFISNN

DDLLVLSCDEDFEDCVPLLEKGVAVYSSELLLNGVITQRLEYERYRLFVANVKKTRSTLW

LKKGGEQFLPVTRT

>Pvulgaris_BCP4

MSPNHSTAKDDRGIHADFDYVDTQPFDADGVEDDDCDDNEWRYFEDTVPFDDDGVLDTET

LVLAGETQVFDEDDDILENEAMNLASETQALGDGETKLPEEECESDRTQILENVDDDELS

VDSGNSEAVGSRKDKSCQRNSSGSMPPRFAFLRVESLRQAALAIHKVDFKENQDVTNSVK

GTGQKYQESLVVKDKGESFLSCSEKVGGVDQENDNGKYSVEVGGFKGKVAKSTVRKLFSD

DIPVETNGTCLSGNVFNEGDDLDKLPIYHGELEGLSYVNSQEPGVLSQINAIDFVDRFLK

DNITEFDQESNCVKNREEKSKSIPSTKWQHSLTKTVNDKGKAGRTSIYDWDDSREDEGGG

DIYLRRKNDFFKGETHRPRSLPGFRNRRACGVNLNDDKEDGNIPNKRKSAVKSDSRVGMN

ILKVRDSVVPEATIKLKRNLAKELDEQFDIDFSRGEMEPVANAGEQMLDVGPDTQMAAEA

METLCNAKDIVDNDTAHVTRSGLNYKLNNSIGKVGLVSSKEQLAQCDRKRKVDVKSLLQT

SGLSKRSTKEVTQHRRDSIMTRSKRSKLNAEGNQTSSDNEKNGRVSLSPIIVQRKSARAL

KSNQLDELNNPDGNNEESRGSLVNKRELHNGVCHFSPIAKRTRRSLSVNQSINRDIPPKS

LRDGNIGIDSLEKSSGIGLQASKTLNSKSTTGSSDDFEVGDNSKLSHLGTSALKASVGSF

SDNVELDIVDYPKRRRSLRIRKLSDHDLGSETLVCSSKASAKPEDTGKSTARKRKMRTDS

DVKSHVNCKDHSSSYDGSVISSVDRKQGKISELNLDKANPGDNVNNSEASSDESPRERNK

VSATPSKYKRPVNDASPVCVGDEYYKQSCNINPTSCLKVSRKELNRELQSLSDIRPELLT

PSKDSRKRRDMTNVRVLYSRHLDEDIIKHQKKILARLGVSVVSSIADATHFIADQFVRTR

NMLEAIAFGKLVVTHLWIESCGQANCFIDERNHILRDAKKEKEVGFSLPVSLALAVQHPL

LKGRRVLITPSTKPSKEILSNLARAVHGQVVEKVGKSVLKGHTVPDDLLILSCEEDYAFS

VPFLEKGVMVYKSELLLTGIVTQKLEYQRHRLFADHVKKTRSAV

>Carabica_BCP4

MGQPGDDDEKKIEQAEFKSNSDLSDTETQPVENGEFSPPQFHDELAGEFEHENDWLQFIQ

DTLPVDDAFPFGDAFETQMVNFGDETQVVDITCETQVVDLGGETQVLDGLDTQVQIDSDA

GGSDKTEVLSYTQELSDDDPATKGIDCSDDLEITLDTELSKQSDGASKAQSDALCNEGRR

SGSIARTFTSVRAASMRASGLAARNMAIKRIDSSSCPKKCNDSLDVQPAEKDKSHSSRDS

LKLTDELNQKHSMEDYDPRIKELGNENTRKLGSSAVRKLFMDEVVSEIEQADDGRNGSDG

TGGVPQLASEHDLAGLSYVDSQEPGDLSQANALDVVDKFLELNAAGSDQDVTFSKSNRRK

SRSVSSAKGIQSLAKKAAVRSSHGGKGIFNWDDDLEDEGGGEFFQKKRELFFENRSLWQR

SIPHSTKPLCLTSKSSLTIPLDTDKKKIVDNTRKLKDAFLSDSRLLSKNSRANESSKPRK

ASFKKNLLPDMDEEMSDASVERVVDAGAHKDLPDKMDVGFDTQMAADAMEALQFAVSVKE

NDCMNGDEGITSVTKSARTDDRSSFNESITQKRTCSSDAELITRQSKKARRTGVKLSRES

YSSSVKQSKNLKRSKRAKANLKDLITNGTENLSTVSKVVEPRKEDRVPAGSDVDNINQTL

ATASAGRKSLKRHPLIEELGSLTPIARRTRASAKASQSKAENNLDSSRLGNGVGKLRHNN

ARKIGQSNQDRCSNVKTFGLEYPKGRRTRSKLPLVSQEAGAQNITRFKRSKRDVTSSSMN

RVENQDERTSVSQGKIILADRTDVGSSLHGNLSNIQENVVKSIISNHSGIKIDMDNSRSA

EGEIMNGSEDASPKDRRKSGASTSTTPVSFTTPISAASPICMGDEYHKQSCRKNLLGSSL

MKELNSRTNTTSPLFTGGVKDLRRRRDMTTVRAMFSRHLDADTVKQQKKILARFGALVAS

SMSEATHFITDEFVRTRNMLEAIAFGKPVVTHLWLESCGQANCFIDERNYILRDARKEKE

FGFSMPVSLSRACQHPLLQGLRVLITPNTKPGKEILGSLVKAVHGLAVERLGRSAWKDER

LPDDILILSCEEDYEICVPFLEKGAAVYSSELLLNGIVIQRLEYERHRLFVDNVKRTRST

IWLKKNSSNQYLPVTKSK

>Mdomestica_BCP4

MGSLWDGHDEIKPMEKNSLADCADTEPIDTQISSPSLSDEKGISMDADELVRDIVPSDDT

VPVEDAFETQMVDFGDETQLMDFATETQVMDFGGETEVLDFGGETQVLDFGGETQVLDLG

GETQAMDFGGETQAVDFSGETQVLDDINCFANMEDTQLLEFDDVVVSDSEESDATEVLDD

SKDLTDNESVQRGSGQLMNEENICRTPCENSENGLTEQANHLIDKQHNAGLHVSAATPVD

DGSPELGPGSVHMQFTSVHAASLQASGLAAHSTALNGTDSESRSVPSNNQSLNQLSGKDN

AVSLLGGSTIDGEEVNEEHDSRNENKWRTGSSTARKLFTEYSDAENTEISHNSASDEEAE

DLLQFPSNLAGLSYIDSQEPGELSQANALDFVDKFLQNNLDESNKEVGHGKSARDNSRFV

SSAKGPQTLAKKANDKSIDKGIFDWDDSREDEEGGEFFRRRKADFFDGRSHGWRSLPQPQ

KSKEKRQDAEKDRKKQLQGKNKRISVVHSDSKLLLHNSKVDKKTAHEDEMKHKKNLVSEF

DEQFNNDSPREKLDANINKNDAPEMMNVGFDTQMAAEAIEALCYGEGISNCDASDDYQDA

QGNQSSPESSMGEKSKNRTCSTKQSSRKRGRFTDAGVSRETQQAKKMRVGGRSSKHSSVS

PLEYSKNTRKECETKVVMAKSKKGKSNAIKLLNIIGNRNMEKMPSVAIDLRTEGSIKKHL

RQDVGTFTPIARRTRRSMVVNQLKKADDASSDCGEESSSQTEDVATREKIISFTGVPVAN

ALNAKSSKSGPNRSGEVGNHKPSQHHGSDLKFEAICNGIKLDALSFPKGKRSRRKLSDQV

YGPDNLNDPPTPSVQPDKVGQRVTRHTRPQGAAQSIFVDVKSTRRTRSATRGDKNCARKF

ARRSLKTDPWKAPLHCNSSHKDGIMISEITTGGEAVGILDRASDANPSSATKMRDESPLG

KCKPLDSACATPVNSKVPVNDASPVCMGNEYFKQSCKNTPSRPSLLKEIRDLSANGHTPT

SASKDLRKRRDMTEVRVLYSHHLDDYVIKHQKKILARLGVSVASSMTDATHFIADQFVRT

RNMLEAIAAGKPVVTHLWLDSCGQASCFIDEKNYILRDTKKEKEFGFNMPTSLVRACQHP

LLEGRKVFITPTTKPGKEIISSLVKAVHGQAIERIGRSVLEADKIPDDLLVLSCEEDYEI

CVPILEKGATVYSSELLLNGIVTQKLEFERHCLFTDQVKKTRSTIWLRKDGSKFLPVTKN

K

>Fvesca_BCP4

MGSLCHADYEIAPIKTNPPLDSDDTEVIEDDTQAVCLAGETQVMDLGGETQLLDCIGDME

TQLLDLDDRVVSDSEGEDSDATQLLDVEEEVSMRRGEGELVVEERTQCREKRLMQQESRL

VDEEHNAGLHVSTTTPVVEESPELKSGYVRMHFTSVRAASLRASGLAARNNSGSRYSLEH

LTTVGGEEDNQGNDMGRNNDKVKSSRVCSSTVRKLFTDNPEAEIEEFPHESNSFEECEDL

LQLPSCDLAGLSYADSQEPGELSQANALNFVDKFLQDNVEEFDKEGECRKSSKKITKVVS

SAKGPQILAKKANEISLVTDAGIYEWDDNREDEGGGELFRRKRTDFFSGGSHGSKSLPQP

WKVKRNRADEPHDNKQTLQGKNKKVGAVHSDSKLLVQNSKASEVMECEDEMRHKRNLTNE

FDEQFNMCSTRGQLDAHVNKPQVPEMLNVGFDTQIAAEAMEALFNGEGISNCEVNNAIHR

NSPEGSMGEKSKNTSVKKPPSRKRAHLSDAGVDSRKSQQAKKTRRVDPNSIEDSSIAPLE

NSKNARKRHESALVIIKSKKAESRAAKHVNISQRKVSERTLSVSIDRGTEGSKKQNLQHG

GTFTPIACRTRQSMLVNQFNEAENPSSDCGDESNNVMEDVAGVQLSENSSKLGLNQASRV

GNTKQNQNEHADVNFEAVGNGVKLDGLSFPRGQRSRRILSSKVHGPGNLDVPSEPSIQPQ

KFGNYGTRRKRSQDARSTTVDNTFKRETRSSTRGSLVNKNLEENFAQKNLDKGGCDGAPP

HCNSSHNDGKTISEKIIVEKTVWVPDRLHDATPSSVKMRDESPRQKGSCQQSDAACNTPV

NHNKVVVNDASPVCMGNEYFKQSCKRSLSRPSLLKELRDLSSLEYEPTSASKDLRRRRDM

TDVRVLYSHHLDEDIIKRQKKVLGRLGVSVASSMTDATHFVADQFVRTRNMLEAIATGKP

VVTHLWLESCGQANCFIDEKNYILRDSKKEKEFGFSMPASLAHACQYPLLKDRKVFITPN

TKPGKETISSLVKAVNGQAVERIGRSALTANQIPDNLLVLSCEEDYEICVPLLEKGAAVY

SSELVLNGIVTQRLEFERHRLFTDQVKKTRSTIWLRKDGNKFQPVSKK

>Gsoja_BCP4

MSPIDSTGNGDRKIHQDFDFVDTQPFDADGEEDDVCGFFEDTVPFGDDGVLETEAVNLAG

ETQALDDGDAFDDDDDGVLETEAVNLAGETQALDDGDTQLLEEESDSDRTQVLENVDDDD

VDEVSVGNVNAEAVDSKKGESSQQNSSGSMPPRFTVLRAESLRQAALACNMDLKETQDVT

NSVEGTSQFCQVPQAVKDNGGSFLRCSEKDDGVDQENKHRKYSVEVGGFKSKSMCKVANS

TVRKLFNDVLPVETNQPSLRSNDFNEGDDLDKLPIYHDELTGLSYVESQEPGVLSQDNAL

DFVDRFLKDNTLEFDQETNSVKKIEEKSKSIPSTKRQHSLAKTVNDRGKSGRTGIYDWDD

NREDEGGGDIFLRRKEDFFKGEMHRPRSLPGFQKSKVCRLNDDKEDKKQLSIPNRRKTAV

HSDSKLGMHILKARDNIIPEATMLKRNLANELDEQFNTDCSRGEMEPNANACAPEMLDVG

LDTQMAAEAMEALCNVGDIVDHVANDATHVTRSGLMYKVNNSSTGKVGSGSSKERLGQYD

KKRKVDVKSKLQTSGLSKKSTKEVRQWTKDNMMTRSKRSKLNAEGNQTSSANENGRVSLS

PIIAQRKSAGALKRHQLDELNNPGGNNGESRGSSVGKRHLQDDVLLFTPIARRTRRSLAV

NPLINVSDDAEMDTLDCPKGRRSLRIRKLSNDDKRSETLVGSSKPSAQPEDIGKHTAGKR

KMRTDSVVKSHVNCQARSSLSLYDGSAISSVDRKQGKISELNSDKANPGDNINNSEVTTL

DESPRERYKSSDLASATPAKCKTPANDASPVCMGDEYYKQSCNRNLSRSCKELHRELQSL

RDIRSELLTPSKDSRKRRDMTDVRILYSHHLDEDIVKHQKKILARLGVSVASSIADATHF

IANQFVRTRNMLEAIAFGKPVVTHLWIESCGQASCFIDERNYILRDVKKEKELGFSMPVS

LAHAIQHPLLKGRRVLVTTNTKPSKEIVSNLTRAVQGQVVEKVGRSVFKGDTISDDLLIL

SCEEDYASCVPFLEKGAMVYSSELLLNGIVTQKLEYQRHRLFADIVKKTRSTLWLKTDDR

TFIPVTKFCTLLRQCCDNCDL

>Slycopersicum_BCP4

MVGGGRKNANKCYPEKDSSVFDDTQPVDTQISPDTLPGEDDQNMAPADDGLFFNDAFETQ

LVNLCGENQILDIGGGETQVVDYGAETQVVDFGAETQLVDFGGETQQVDLDGETQLVDDH

DCLHSKRIQTSENCNIEVVDSDTEGSDGTEVLCDTQELSGDDSMEHSYSSIDQVKLPKSS

NSNTSERSSIAQSDVQRSLQRGFTSIRAASIRASGLAAYDMSRKGTKGSTRSIKSENPLE

QEGADHNGTSVVRPQSVVRKELNLNACEEYDEQLKEVGNEYRCKVGSSAVRKLFRDEILI

ETKGPEDGNYDFQKTVDLPQLDSDNVLTGLSYLDSQEPGEETQANALEAVDKFLSLNPFD

FDQHLDFGKSSIGKSKCVSAASGAKHLAQRAAGIADAEGGIYDWDDNREDEGGGEFFQKK

KELLFGRIPTTEPLKHGSLDPLRRGSKSCGPKEKHMLNCKKFKGSPCSDSRLMSSKVRVK

SELSKSRSRKKLVEELDEQFTVGAGNGMVDNGDGDNVPDLQNVGLDTQMAAEAMETLCFR

VPVLENDFSNENKCNKSLSKSSCKGRVDDESLLKQRSPKKKARSSDTRPATRLSVQKDVK

LVEVHCRETVKQQKSSKKQGNDEQGARLRMIKANMTISHSSRGKEEEFGQVERPPKESRG

SMSVKNCHLQQQHDSFTPIAHRTRHNRAESQLKSRLSAAVTSNRSGIDGDACETLMDHGT

FAADRTANLRNMKLMWGDHCAVDHPKGKRSHRKIPAMGQEATTQPCRRSKRLSGDQTSTS

IDVSAKKRKCSPETPSGIASSGRGSRKKLSNEGINKGHPEGTNISDAFADGNTKALRYKS

PEDSNMKADVATKQSVDEAHGVESLTGDQCKAPASACTTPTNSKILKSSVSPICMGDEYQ

KQSCRKNTSRSSLMREIISLHTTGTQVDSTLKDSRKRREMTNVRILFSQHLDPDIIKQQK

KIIARLGASSASSMSDATHFMADEFVRTRNMLEAIAAGKPVVTHLWLESCGQASCLIDEK

NYILRDARKEKEFGFSMPVSLARACQHPILQGYKVFITPNTKPGKEILASLVKAVHGLAV

ERLCRSAMKEEVIPDNLLVLSCEEDYEVCIPFLEKGSTVYSSELLLNGIVTQRLDFDRYH

LFSDHVKRTRSTVWMKKNNNQYLAIAKCK

>Spurpurea_BCP4

MPFEDTVRVEDAFETQVVDLGGETQALDDLDWFENVDTQLIDEIIDSDGEGTDRTEVLDD

GDELSDDESGRRGKCESLEGEKIQDTSLSKHGEKGLIEHPDDLTDEHHLSGSVLKLTSVR

VESLRVSGLVARSSASNGTNNSDSCSLVIDGQVSEQFTVNTNGSKTKIPEDVDWRHDIGR

SDDEMKEFSNGSRCKIGCSAVRNLFAENSFIETKGHFVGGKEVPICDDGVAGLSYIDSQE

PGDLSQADALLCVQKLIEESKVLFDEVDLGKIDRKNLSHISAAKGAQSLAKKTTDGSTEG

KSRIFDWDDGLEDEGGGDIFRRRKENFFGTANLGQRSFMKPSKSKGNQPGVCMDNEGKSN

VQNVIVVHPESKIDLCSLRANKKMTQETETNVSRNLFNEFDEQSEADTSTGQLEALTRKE

VPEMLDIGLDTQMAAEAMEALFHGEGIAYDATNDSQQIPAVNFKDLVEGSMRSKAKKSIC

SKQHSLSNNEDIGVTTHQSKKTLKISAKLGKQSLTPSQKCPENVRKECDMDVVLTRSKRA

RLDAGLNELDRCNGTASRGYRSVKKQDLPEELATSTPVAHRTRQSLAVSKLKSAENVSID

NREKSNRLVVLGCREWNKAGVVDVEASKVLNAKGNSSGLGSSQPGEPKTSKSRLLAIDND

ISFPRRKRSRQNFSGQLDEPHNLHARSKPSCQPGNIEKSVSWCRRSQINARISTDLNMKR

KTRSSMSVCPNLSPHHFHKQGIDGTTCNYNSVDMNGKMSNNLMGVGASVKSSHRRSNADS

SPSAANEVNVPSSDNLPSEKTKSYESACASPGICTTPVNAASPVCMGSEYKQSHRKNLSR

ACLVKEMNSLCAAWPGPISEPKDTRKRRDLSDVRVLFSHHLDEDIIKQQRKIADRLKVSI

ASSITDATHFITDNFVRTRNMLEAIASGKPVVTHLWLENVGQANYYIDEQKYIVRDSKKE

KEFGFNLAVSLAHARQHPLLHGRRVLITPKTKPGKEIISSLVKAVHGQAVERVGRSTLQD

DVVSDHLLVLSCEEDYDVCVPFLEKGAAVYSSELLLNGIVTQKLEYERYRLFADHVKRTR

STVWMKKDGHNFIPVTKRR

>Mesculenta_BCP4

MQENQNSHSFYTDSQPFDSQIFLSSSPGDKGGNANEVPLAQNTVSFDDPVPVEDAFETQV

VDETQVLDDPLCLQHMDTQLIDDFNCDGEGTDKTEVLDDSDGFSDDDSQRRGRCESLYGE

RSWQTSLEHRLNATPETPTVQISQEPKPGSTTRFTSVRAASLRASGLAARRIVLKGTNKE

SSFLQNNNQLSEEHAMKNNGSNTEVWKEVDQVCGAGRYNEVKELMNVHDCKIGRSTVRKL

FDDNYFVENEELASRNDNTAGGKEIHQFPTCYDGLVGLSYIDSQEPGELSQADAFACVQR

LIEENKALCDNEFELGISSKGKLNFVSATKGPQSLAKKINCGGTNWKTGIFDWDDEQEDE

GGGDLFCRRKEEFFGSAKLGQGSFMKPQKVKGKQLDGYTKTIGKSDIQNEIVVQSDSQVV

LHNAELNNKKAPEAEMGFRKNLVSAFDDESNIATTSGQLQSGLARNQMLQVLDVGLDTQM

AAETIEASLYGDGITNIGANNVSGNPVSQKGSPGRKVKRMMHDIGVSTRQSKKSKRIGAK

SNEQPSRKVKKRMPQAVGVATRQSKKSKRSTSISSKNHSEIVRNKSDMELVNTRRRTKSN

FEVSMTNGIRGADKMLTKMAGGPIERSLHDALDAHHEAALTGSGSVKKWNLPEEFSTLTP

IACRTRRSLVAYQLKRTENVSRGCGEEKNCPIEISALRQNEAGAGVEAAKVLYANGKSSE

LISEQSGEPENFKSRSTSMNYGISCPKRRSSRQKSNKLNEQVNLDAQSKACTSLANLNTK

RKTLSSKSICLESSSLYENFKGESSQRSIDKSSSGDAALSSIENGKKSSADQMGAKNKLP

DRKDNALLSPSMEHEVKEILDHLHNEETKPSNSMCNSPVNSTNPPVNSASPICIGNEYVK

QSGTKRLSRSCLMREVSSLCATEREPISKLKDSRKRKELGDVRVLFSHHLDENMIKRQQK

IVDRLKVSVVSSITDATHFITDKFVRTRNMLQAIASGKPVVTHLWLENVGRANYYIDEQK

YILRDMKKEKEIGFNMPVSLAHARQHPLLQGRRVLVTPSIKPAKEIILDLIKVVCGQAVE

RIGRSTLKNDMVPEDLLVLSCEEDYEVCVPFLEKGAAIYSSELLLKGIVTQKLEYERHRL

FADHVKRTRSTIWLKKGGGDFVPVAKHK

>Tcacao_BCP4

MGSLGDDNGKIKPSQMNPKTDSSLAETQPFDFDSQFSLPAVSGDKVDNEDDDGLQYLWSS

APFDDDNVPGEDAFETQVVNFCGETQVLNFGGETQVLDDVDCFENMETQLLDEFDDEVAL

DNDGEGTDVTEVLADGDEDSNDDLSRGDCGRFLGQEEKKESLEQCNASIDEQRSSAVHVS

TPDVEAVPESKPGSVRRFTSVRAASLRASGLAARNAALRGMNSESCSIRTDSQFSDQCIG

NSDGLNPKVEKINQAHDQGNHDEKSISLRNGVNCSVGCSTARKLFAEKEGPFCRGENADA

KEGLLQRDGSLAGFSYIDSQEPGELSQANALNFVERFVIDNLMELDGEVDLGKSTSGKSK

LISSAKGLQSLAKKTIERSTAGETRIFDWDDFIEDEGGGDIYCRKKEEFYGNESRARKSS

THPQKPKGRKLDESCNEDRPNSHDKKIVYSDSKLLLCKSNVNGKKVSEGSMNFRKNLFNE

SDEQFNSDFSRGQLEATASKTGAPELVNVGFDTQMAAEAMEALFYGDGATGQNANRGAES

ITKGSSKGSLRGKSRKRVSSREPTMGKGVRCSDAGPVTRRSKKTKSSKECLLLQEKHSKN

VRKECDTELLLPEMKKAKQNTDEHQIAGGIDMFKTPSKSIKQRKAGGALERSQYNGTRRS

TRGSSIKKRHPEKVHTFTPIAHRTRQSLVMNVQMSETQASDWRKGRKHQKEVDFLQENRT

GSTDIKLSAVSNAKGQLSELGSNQSGECENVKSSDNDQLHLELIARSSNHALSYPKQRRS

SWKMCVDVGESDNLKAQSKRSVLQEDKGQSIAVLKRSRSNNRSTHIHSSTRRITRSSVNS

RPVLYFSDQNPEGKLSHQSSDKEGSEDDVINYNSTEMNGRMVSTRITGPEPAKSAKHSDG

NRDAVSSPIAESVAVNVTLDKSPKEKSKSPGSKCTTPVNCPTPINAASPVCMGEEYYKQS

CKKNLSKSSLNKELKSLSPIEPEPISPLKDMRKRRDLANVRVLFSNHLDEDIIKQQKKIL

ARLGISEVSSILDATHFITDKFVRTRNMLEAIASGKPVVTYLWLESIGQVNIHIDEEAYI

LRDIRKEKELGFCMPVSLARARKRPLLQGRRVFITPNTKPGKETISHLVTAVGGQAVERI

GRSATKDDKVPDDLLVLSCEEDYVICVPFLEKGAAVYSSELLLNGIVTQKLEYERHRLFA

DHVKRTRSTIWLRKDDKFLPVTKHK

>Ahypogaea_BCP4

MASSREDKNKNNNTYSRDTSIHEEFDFLDTQPLDIPHSPSCDDESRYFEDTEPFDDCDDD

NMEAEPMNLAGETQVLDFAGETQPLDDFDGAALVDDCTQLLNEEGDVGFESGGEGEVEGL

DGTQVLEGVDDDADVSDGGDRQSAYNKQEGEHERFSDEKSFGNIDSIENELNRSGLKPPR

FTYLRVESLRKAALAARNVASKKTSNRTSSDKGNSQSCQEHVVVKDNDESFPRCYGKVEE

VNRECSGAKYSMEIEGQKNKNADKVASSAVRKLFNDALPIETNEPSLESNDFDGVDELPI

CHDGLSYVNSQEPGELSQLNALDFVDRFLHDNIMEFDQDTNRVKNTTRITTTTPLSANCA

KNVEEKSKSVANGKGQQNLAKRVTDRGKDAKAAVFDWDDSREDEGGGDIYLRRKEDFFYC

ETRGSKSLPVSQKTKFCRPNNDKDAEEKLNIPSRKTRAVHSDSRLGRHNQRVWDNNVEEA

TRRITRNLAKELGEQLDAYCSRGEMEPKANADVQETMDVGLDTQMAAEAMEALCGAESLA

NDTICNTRSSSKGQHNPVGSRDSSRQFDKKRKAEVKSDLQTSSLSKKCTEVGQCKKGNIV

TRSKMTKLNAEAIQTSGANENGRRGVLSPMVDQRKSTGALKILDHGELKIHDSNDTASGR

SRVNEKHLQDEVYHCTPIARRTRQSLTVNQFSGRIGSLEKTNGTVLHADESLDPNSTPKA

STAGISNEIEMDALDCPRRRRSLRIRKYSDHDKGSENLVDSSKSSVPPKGKSSAGNKQSS

IHSRSPIDCNVIAEKDENLKSYGKNIAAVRLSSNNFEVTNSDESPKDCHKSSDLASATTP

ANCKTPMNDASPVCMGDDYYKQSCNGNLSRSCLHKVFRKELQRELRNLSPVSPELTTPSK

DSRKRRDMTDVRILYSNHLNEDIIKHQKKILARLGVSVASSIADATHFIADQFVRTRNML

EAIAFGKPVVTHLWIESCGQASCFIDEKNYILRDAKKEKEFGFSMPVSLARASRHPLLEG

RRVLITPNTKPSKEIISTLVGAVQGQVMERISRSVLKENKILDDLLILSCEEDYASCVPF

LEKGVMVYSSELLLNGIVIQKLDYERHCLFAEQVKKTRSSIWLRKDDRTFLPVTKCT

>Ptrichocarpa_BCP4

MGSLGDDDDGEIKAGREDPNANFAPSYTQPFDSQFLPSPLPGEKGEDANELQFLQSTMLF

EDTVRVEDAFETQVVDLGGETQALDDLDWFQNVDTQLIDEIIDSDGEGTDRTEVLDDGNE

LSDDESGRRGKCESLDGEKIQDTSLSKHGEKGLVEQSDALTDEQHLSGSALKYTSVRVES

LRVSGIAARSSASNGTNNSDSCSLVTDGQISEQFTVNTNRSKTKIPEEVVWRHDMWRSDD

EVKEFSNGSRCNIGCSAMRKLFAENSFIETKGHFVGGKEVPICDDGVAGLSYIDSQEPGD

LSQADALLCVQKLIEESKVLFDEVDLGKIDRRKSSHISAAKGVQSLAKKTTDGGTKGKSR

IFDWDDGLEDEGGGDIFRRRKEMFFGTANLGKRSFMKPSKSKGNQLGVCRDNEGKSNVQN

VIEVHSESKIDLCSLRANKKMMQETETNVSRNLFNKFDEQPEADASAGQLEALTRKEVPE

MLDIGLDTQMAAEAMEALFHGEGIAYDATNDGQQIPAVNSKDLMEGSLRSKAKKSIHSKQ

HSLSNNEDIGVTTHQSKKTLKISAKLGKQSLTPSQKCPENVRKQCDKDVVMTRSKRARSD

AGLDELDRCNGTASRGHRSVKKQDLPVELATSTPVAHRTRQSLAVSKLKSAENVSIDNRE

KPNSQVELGGREWHKAGVVDVEASEVLNAKGNSSGLGSSQPGEPKTSKSRLLATDNYISF

PRRKRSRQNLSGQLDDPHNLHAQSKPSCQPGISTDLNMKKKTRSSISVCPDLSPQHVHKP

GIDGTTCNYNSVDMNGKMSNNLMGVGASVKSSHRRSNADSSPSAANEVNVPSSDNLSREK

TKSYESACASPGICMTPVNAASPVCMGSEYKQSCRKNLSRACLVKEMSSLCAAWTGPISE

PKDTRKRRDLSDVRVLFSHHLDEDILKQQRKIADRLKVSIASSITDATHFVTDKFVRTRN

MLEAIASGKPVVTHLWLENVGQANYYIDEQKYIVRDSKKEKEFGFNLAVSLAHARQHPLL

QGRRVLITPKTKPGKEIISSLVKAVRGQAVERVGRSTLKDDVVPDDLLILSCEEDYEVCV

PFLEKGAAVYSSELLLNGIVTQKLEYERHRLFADTVKRTRSTVWMKKDGHNFVPVTKRR

>Graimondii_BCP4

MGPLGSDNGDIKPSEMNPSSDFSYSDTQPFEFDSQYSFLGDKADNEDSDELDYLQSTVPF

DDYNVQVEDGLETQALNLGGETQVLNFDGETQVLDDLDCFENMETQLLDEFNDAIAADSD

SEGMEGTEILDQGDEVSNDEIVTGDCGQFLFQKKESLEQHNASTNEQMNSGIHGSTTTPD

VRAVPESKSRSVRFTSVRAAALRASGLAARKAALRGMNGKSCFIQTDCQFSDQCTIKSDG

SNPNVVEKMNQAQHLVNREENSIGLRHGTNCRVGCSTVRKLFAERSSCRSENVDARDDVL

QFPASDGQLAGLSYIDSEEPGELSQSNALNFVERFVNDKMTELDDQVDLGKSTGRKSLIS

CAIIGPQSLAKKTVERTAVETQIFDWDDALEDEGGGDIYCRRKEEFYGDGSHARKNSNHA

HKPKGGKLNESCNVDQPNAHDKEIVDSDSKSLLCKSKDNGKPVGEGQLDFRKNLLNEFDE

QCNSDSSRGQLEAAAAELNVGFDTQMAAEAMEALFYGDMATDVNGNQGFPGISKGSSKVL

CRGKCRKRISSRECILRKGVYCYGAAPVTRQSKRTRESSVLPNISSKNVRKECDTDLLLP

KTKKAKSNNDKNQNNGGINMTKMPSKSIKKRKAGGALTRSQLHGTGRSTMSSSIKKRHLE

EVCTVTPIAHRTRQSLVMNAQIAEASASDCRKGKKLEKEVGLLQENRTRSIDVTDVELSL

ASNAEEQFSKLHSNQSGEHGNVESCNDDQLHLGLIAGNNGNHGSSYPKHRSSRKMSVHVG

ESDNLEAPSGKSVQLDNEPSIPVVKKSRRNNRSTCIRSTTVRITRSSRNTCPVLHFPDQN

SEGKLSRQSSDKQGSKDNAVNCNSTKMNRRMISTSITGPEAAKEIQHSGGNHVAVSSPIA

ENLAVNVATNKSPEEKSRSLGSLCTTPVNCPTPINAASPVCMGEEYFKQSCKKNLSKSLL

IKELRSLNPIDPEPISPSKDMRKRRDLADIRVLFSNHLDEDIIKQQKKILARLGISEASS

ILAATHFVTDKFVRTRNMLEAIASGKPVVTHLWLESVGQVNIHIDEEAYILRDIKKEKEF

GFCMPASLARACRRPLLQGRRVLITPNTKPNKETIVHLVAVLHGQALERIGRSAMKDDKV

LDDLLILSCEEDYAICVPFLEKGAAVYSSELLLNGIVTQKLDYERHRLFADHVRKTRSTI

WLRKDNKFLPVTKHK

>Pamilis_BCP4

MGPLGARRIRNLPFEKDSMFLSDESPSPDECKGRAIENEHFGDTVKVDDNDAFETQVMDF

DNETQVMDFDNETQVMDFDNETQPVDLYEETQILYPSGETQVMDDFDVDEFIDTQLLDLS

DNETNCKDDELGAQRTQLLDTPEELLGENGDKDGAGENDIHGSHQDDRGAKAETLRQDDS

VLSSGSLREGFTAIRTASLRAAGLRAQRKAPKDADRSYFTELSSKSVDLLCLDNHGGISD

AETADLENDTETEANKGLSNEFRSKVARSLAAELEDVASDRRCNDSQIGEMRSSPQLAAC

DDEVAGLSYVHSQEPGELSQANALEVVERFLKINVLESDHDTPNKNPGEMGSCHNTIVKG

PQSLARQTHLRNSLAGGASVFDWDDNCEDEGGGEFFTKKKELMLGTKSRCQSAGKLRNEK

VVRNIHDGTKALAHSDSKLAKSRRISKHKEIKNKVKYPDLLEVVPCCDEELLASAPNPEP

TSMLDVGIDTQMAAEAMEVLSSGIALATSNGGAGDEDHQNMTRKAAVKNTRAKQSSVKEQ

SFNSKPGTTKSKRTTRAALKSGEGSKSSVACRTRSRCTVDEFNIGMSKSNVKRRRLPTKN

ISPIVQALVAPTTNNLKVVDHVNGTEDEPQHSSGEKESNLNARQETLDPSTVVASSNNNG

ASCRQKSLQLASNQVNGSGGKQSPLGASGLDVISQSVTRRTRSKRSVRCVEFPSIGKVLR

SGVEISSGDACQSSSEKNKPCSSPAQGAGQPREPEGSPVQVQNSANVTPVNLKTPTSTTG

SPVCVGDGYSTPSGKRRSLSHEFCRLTASKLKDASPSKELRQRRDFNTVRVLFSQHLDDE

VVRKQKKILSRLGVPMASSITEATHFVADEFVRTRNMLHAIACGKPVVTHLWLESCGESN

CFIDEKNYILRDAKKEKELGFSLPNSLARASRYPLLEGRRVLITPNVKPSKEVVSGLVKA

VHGQAVERLCRSLSTNTKLPDNLLVLSCEEDLQECLPLLEKGVAIYSSELLLSGIITQKL

EYERYRIFVDHVKRTRSTLWVKKNGNNFLPVTLAK

**BRASSICACEAE**

>Athaliana_BCP4

MAKSNQNFSPYEETQPIDSNPPSSSDSGEDKDSLLGDCDTQPVDDNALINDQYMETQVMD

TECDNEEFLLCNETQAVDLGFETGEEVLVEGKQLLEASDGLATQVLDLCDDEVVVDSDDD

VTDVLEGNSDLSDSDDSGSKAETVLSSEENRQDANEKVKSAVVFDAWSNEHGVSGKKVAR

FASVRSAAFRASAVAARVANQKSANTDCSTLINCHSSGKGTTHNSGLENSVGEVGNQQSL

TSLFVEEKKDLRTGKKTARKLFVEDFPEEKFHSTDCNVDLGNLSYIGSQEPGEESQASAL

NLVDKLISECRLEFDFEVQADYGRKTEDKSKFVQIFKGPQELAKKVSYKSGAVGNNIFDW

DDNREDEGGGDIYRRRKDEFFGVASKRREFSSLPREQKRELIPVAVDKRWARSDSKLLKH

SVTRSRKNIQGAKKNLGKELDEVREAAVLGNDTQVAAEAIDDLCSGDRGKFDGEASCLTG

KKLSPEEERGFSPGGVVTRQSKGTKRIQAMSKDELLKKRMKKASPSPAKACRTNIEGSSN

GDQLNKEGPCCWKSRKVQTASRETKKNLVDEFDEVSQESNTEMFDRHEEAEAGPDTQMAA

EVMNALHSGDGREIDPEPNNLIGKKLLLEGGISRCGVVTRKSKRIKGIQAVDNDVESLKP

KNKKARSILAKSFEKNMDRYSKNDKVDTPDEAVASTTEKRQGELSNKHCMSKLLKQSHRG

EAEVLNYPKRRRSARISQDQVNEAGRSSDPAFDTPAKSKTPSTNVSPICMGDEYHRLSCK

DSFTSHTTREFRSLTVPVAEPISETKSTRKRRDLGSICVLFSQHLDEDVTKHQKKILARF

DISEASSMKEATHFIADNFTRTRNMLEAIASGKPVVTTQWLESIDQVNIYVDEDMYILRD

SKKEKEFCFNMGVSLARARQFPLLQGRRVFITPNTKPALNTITTLVKAVHGLPVERLGRS

SLSEDKVPENLLVLSCEEDRAICIPFLERGAEVYSSELLLNGIVTQRLEYERYRLFTDHV

RRTRSTIWIKDGKGKFQRRSG

>Evesicaria_BCP4

MGKVKNNFPANLEDTQPIDSPSHSGEEKDPWIGSFAHEFMQDTVPFEDDAWISDEYTETQ

VMDNECDTEEFLLCCETQAVDLGLGTQEESLVEEDQLIQGSDGLDTQVLDRFDEDVVVAD

SDDDVTVFLEDNREREVSDCGDSHSKRGNLLLSDDKNSEHANENTKSTVGVDAEYGISGK

VARFASVRSAAFRASAVAARVAAQKLLNSDCSTLANCHSSDQAATHYSRPEPNIGDVRDQ

DCPPNMPVEKKNEGNRTARKLFPEDDSPEENCPPPGLSYIDSQEPGEASQANALEFVDKL

ISESALKFDVEVEADCGKRTEEKSRSIPSVKGPQELAKKASYKARAVGNSIFDWDDNRED

EGGGDICRRRRDEIFGVTSKARRSSPLLDKRKVHSDSRLLHRSVTRGGKMFQAATKNLGK

ELDAVSDEHNKKISDMRELVALGYDSQVAAEAIDAVCSGDRKKVDACSTGKKLPPGKGRG

FSNGGVITRQSKGTKRIHAMGKDELLQRRMKKASPSPAKSCGKSIERSSKRRKVLTASRE

TKKNLVNELDKVSKDSNTRMFDRREEVEAGPDTQMAAEAINDLHSGDAREINHESNCLIG

KKPSREGAVTRKSKRFKGIQAVDDDVESLKPKTKKAKSIHAKACEENLDSYSENEKHDMP

DTEKKRGELSNKHCKSKLLKQSSGGEAEVLSNPKRRRSARISQDRENEAGRSSDPAFDTP

VKHKAPSKSVSPSNGPSKNVSPICMGDEYHRLTCKVSRTSNTTNELRNLTLPFVQPIPEA

KSTRKRRDLASIRVLFSQHLDEDVTKHQKKILAKFDISEASSMMEATHFIADNFMRTRNM

LEAIASGKPVVTTQWLESIDQVNIYVDEELYILRDTKKEKEFGFNMGVSLTRARQNPLLK

GRRVFITPNTKPGLNTITTLVKAVHGQPVERLGRTVLSEDKVPENLLILSCEEDRDISIP

FLERGSEVYSSELLLNGIVTQKLEYERYRLFTDHVRRTRSTIWVRDGKGKFQRRRG

>Itinctoria_BCP4

MAKVNNNFSENQETQPIDSLPSSPSDSGKDKDCWIGNCGDEFMQDTVPFEDDAWINDEYM

ETQVIDNGCDNDDFLLCRETQALDLGFETQEEPLVDGEELLQTSDGLATQVLDRFDEEVV

ANSDDDVTVFLVDNSEISDCGDSHSRGRNMVPSEDSREHANGNIKPTSVLDARSNGNGIS

GKVARFASVRSSAFRASAAAARVAAQTLLNSESFTLASCHSSGQGATHNSRLEQNVGELG

NQKCPPNMSVEKKNKGNRTARKLFIEDDSPEEIFPTPGLSYIDSQEPGEASQASALKFVD

KLISESCLKFDVEVEPDCGRRTEEKLKKIPSVKGPQELAKKVSYKARAVGNSIFDWDDNR

EDEGGGDICRRRNEEFFGVASKARKSSTLPREQKRELIHENHRGLGVAVDKIKAHSDSRL

LQRSVTRGRKMIQAAKKNLGKELDAVSENGNKKISDMRELVALGYDIQVATEAIDAACSG

DRSKVDAEASCLTGKELSPGEERGFSNGGIITRQSKGTKRIHAMGKDELLKRRMKKASPS

PAKACGKSVERSPKGDQLDKEGSCFWKRRKVHSTSRETKKNLVGKLDKVSKESNTRMFDR

REEVEAGPDTQMAAEAINALQSGDGRKIESEPNCLLGKKSPQEEGCISSGGVVTRKSKRL

KGIQALDDDVESLKPKTKKAKPIPVKASEKNVESDSKNEKLDMPDGVVMSSTEKRKGGLS

NKHCTSKLLKQSSGGEAEVLTYPKRRRSARISQDQVNEAGRSSDPAFDTPVKSKAPSKNV

SPICMGDEYHRLSCKDSRTSNTTREFRNLTSSLVEPIPETKSTRKRRDLGSVRVLFSQHL

DEDVTKHQKKILARFNISEASSMMEATHFVADSFMRTRNMLEAIASGKPVVTTQWLESID

QVNIYVDEDLYLLRDSKKEKEFGFNMGVSLARARQNPLLKGRRVFITPNTKPGLNTITTL

VKAVHGQPVERLGRSVLSDDKVPENLLVLSCEEDRDISIPFLERGAEVYSSELVLNGIVT

QKLEYERYRLFTDYVRRTRSTIWIRDGKGKFQRRRG

>Cgrandiflora_BCP4

MAKANNNFSADQETQPIDDDSNPSSPSASGEDKDSLFGDCDTQPFEDSAWIDDQYMETQV

VDIGCDNEEFLLCGETQAVDLVFETQEEPFVEGTQLLEASDGLAATQVLDHFDDQVVADS

DDDVTAVLEDGSELSDDSDDSCSKAKTVLSSEENRQDGSGKVKSTCALDAWSNEHGISGK

KMARFASVRSAAFRASAVAARFAAPKIPNSDRSTLVNCHTSGQGDTHNSSLEYRVGEVGN

QQSLTSIFVEKKNDLQTGNRTARKLFQEDLPEENCHSVDYNVDLEDLSYIDSQEPGEASQ

ASALKLVDKLINESRVEFGFEVEADCGRRTEEKSKFVPIFKGPQELAKKLSYKSGAVGNC

VFDWDDNREDEGGGDIYRRRKDEFFGIASKGREFSTLPREQKRELIHESHGGLGDVVDKR

RTRSDSKLLQHSVTRSQKNIQAAKKNLAKELDDVREAVVLGNDAQVADETIDDLCSRDRS

KFVSEASCLTGKKLSPGEERGYSPGGVVTRQSRGTKRIQAMSKDELLKKRMKKASPNTEG

SSKDDQLDKEGPSCRKSRKIQTASRETEKNLVVGLDEVSKESNTKLFDRNEEVEAGPDTQ

MAAEVMNALYSGDGREIDPELNNLIGKKLSLKGGISSRGVVTRKSKRIKGMQAVDNDVES

LDTKTKKAISICAKSCEKKRDRYLKNDKVNPPDEVVVSTTEKRQGEFSNKHIMSKLPRQS

SRGDTEALNYPKRRRSARILQDQVNEAGRSTDPTFDTPVKSKMPSTNVSPICMGDEYHRL

SCKDSVTSHTTREFRSLTLPLAEPISETKSTRKRRDLGSVRVLFSQHLDEDVTKHQKKIL

ARFDISEASSMKEATHFIADNFTRTRNMLEAIASGKPVVTTQWLESIGQVNIYVDEDLYI

LRDIKKEKEFCFNMGVSLARARQFPLLQGRRVFITPNTKPGLNTITTLVKAVHGLPVERL

GRSILSKDKVPENLLVLSCEEDRTICIPFLERGAEVYSSELLLNGIVTQRLEYERYRLFI

DHVRRTRSTIWIKDAKGKFQRRSG

>Brapa_BCP4

MTKDHNFSENQDTQPIDSPPSSPSDSGEEKDGFFGNLGDEFMNDTVPFEDDAWINDEFMA

TQVIDNDEFLLCRETQAVDLGFGTQEEPFVEDEQLLQGFDGLATQVLDPSDEDSDDVDVT

VFLVDNSEVSDCGDSSSRRKLLSSEDKSREHAPSSGHENVKPTGKVARFASVRSAAFRAS

AVSAQKLLNRDTPTLASCHSTGQGATNSSILQQHFGEVGNQKFPPNTFVEKKNDGKRTAR

KLLFEDDSPEENCPSPGLSYIDSQEPGEASQASALKFVDKLISESGLNLDVEAEDDYGRR

REETLHKISSVKGPQELAKKASLKARAHGKSMFDWDDNREDEGGGDICLRRKEELFGVAS

KAQKPSSLPREQRRELGVSVDKSQRKSRSDSRLLQCTVTRGPKMIQAAKKNLGKELDAVA

EEGYNNTSDIRDLVELGYETQVAAEAVDAVRSGGGSKVNAEASPGNKLSPGEERVITRQS

KGTKRIQAMGKEELLRRRMNKASPSPAKACRKSIERSLQSDQLYKEGPNCSKRRIVHTAP

RESRDNLVDEMDEVSKESKTRMFDRREEVEAGPDTQMAAEVISALHSGDARDEAKKSSRG

VVTRKSKRLKGIQAVDDDDESLKPKTKKAKSVLVKAYSKNEKVDLPDEVVVSKLLKQPSG

GEADVLSYPKRRRSARFLQDQVTEAERSSEPAFDTPAKSKAPSKNVSPICMGDEYHKLSC

KDSRTSNTTREFRNLTSPSMEPVPETKSTRKRRDLRSVRVLFSQHLDEDVTKHQKKILAR

FDISEASSMTEATHFIADNFTRTRNMLEAIASGKPVVTTQWLESIDQVNIYVDEDLYLLR

DSKKEKEFGFNMGVSLARARQNPLLKGRRVFITPNTKPGLNTITTLVKAVHGQPVERVGR

SVLNDDKVPENLLVLSCEEDRDISIPFLERGAEVYSSELVLNGIVTQKLEYERYRLFTDH

VRRTRSTIWIRDGKGKFQRRRG

>Bstricta_BCP4

MAKVNNNFSAHQETQPIDSLPSSPSDSGEDKDNWLGNCDTQPFEDNAWINDQYMETQVMD

IECNNEDFLLCGETQAVDLVFETQEEPLVEGKQLLEASDGLATQVLDHSDDEVVADSDDD

VTAVLEDNSELSDSDDSCRKAETALSSEENRQHANEKIESTCALDAWSNEHGISGKKVAR

FASVRSAAFRASAVAARVANEKFPNTDCSTLVNCHSSGKGDTHNSSLENSVGEVGNQQSL

TSIFVEEKNDLRTGNKTARKLFLEDLPEENCHSVDCDVELGDLSYTDSQEPGEASQASAL

NLVDKLISESRLEFAFEVEADCGRRTEEKSKCVPSFKGLQELVKKVSYKSGAVGNCIFDW

DDNREDEGGGDIYRRRKDEFFGVPSKGREFSTLPREQKRDLIHESHRGLQVAVDKRRARS

DSKLLQHSVTRSRKNIQAAKKNLGKELDEVREAVVLGNDTQVATEAIDDLCSGDRGKFDA

EASCLTGKKLSPGEERGFSPGGVITTQSKGTKRVQALSKDELLKKRMKKASPIPAKACRK

NIEGSSKGDHLDKEGPCCWKSRKVQTASRGTKKNLVDEFDEVSKESNTKMFDWHEEVEAG

PDTQMAAEVINALHSGDGREIDPEPNNIIAKKLSPEGGISSCGVITRKSKRIKGIQAVDN

DVESLKPKTKKARSILAKSCEKNMNTYSKNDKFDTPDEAVVSTTEKRQGELSNKHCMSKL

LKQSSRGKAAVLNYPKRRRSARISQDQVNEGGRSSDPAFDTPVKSKTPSTNVSPICMGDE

YHRLSCKDSVTSHTTREFRSLTLPLSEPISETKSTRKRRDLGSIRVLFSQHLDEDVTKHQ

KKILARFDISEASSMKEATHFIADNFTRTRNMLEAIASGKPVVTTQWLESIGQVNIYVDE

DLHILRDSKKEKEFCFNMGVSLARARQFPLLQGRRVFITPNTKPGLNTITTLVMAVHGLP

VERLGRSALSEDKVPENLLVLSCEEDRAICIPFLERGAEVYSSELLLNGIVTQKLEYERY

RLFTDHVRRTRSTIWIKDVKGKFQRRSG

>Mperfoliatum_BCP4

MAKVNNNFSENQETQPIDSLPSSPSDSGKDKDCSIGNCGDEFMQDTVPFEDDAWINDDYM

ETQVLDNGCDNDEFLLCRETQAVDFGFETQEEPLVEGEQLLQTSDGLATQVLDRFDEEVV

ADSDDDVTVFLVDNNEISDCDDANSRGGNLLPSEDSREHANENIKPMGVLDARSNGNGTS

GKVARFASVRSAAFRASAATARVTAQTLLNSESSTLASCHSSGQGATHNSRLEQNDGELG

NSKCPPNMSVEKKIERNRTARKLFTEDDSPEEIFPSPGLSYIDSQEPGEASQASALKFVD

KLISESCLKFDLEVEADCGRSTEEKLKKIPSVKGPQELAKKIRYKARAVENSIFDWDDNR

EDEGGGDICRRRNEEFFGVASKARKSSTLPREQKRELVHENHRGLGVAVDKRKAHSDSRL

LQRSVTRGRKMIQAAKKNLGKELDAVSEDSNKKISDMRELVALGYDTQVATEAIDAVCSG

DRSKVDAEANCLTGKELSPGEERGFSNGEIITRQSKGTKRIHAMGKDELLKRRMKKASPS

PAKSCGKSIERSSKGDQLDKEGPCGWKRRKVHTASQETKKNLVNELDKVSKESNIMFDRR

EEVEAGPDTQMAAEAINALHSGDGMKIDPESNCRIGKKSSREDGGISSRGVFTRKSKRLK

GIQAADDDVESLKPKTKKARSIPAKAGEKNVDSYSKNEKLDMPDEVVVPSTEKRGGELSN

THCTSKLLKQSSGGEAEVLAYPKRRRSARISQDQVNEAGRSSDPTFDTPVKSKAPSKNVS

PICMGDEYHRLSCKDSRTSNTTREFRNLTSPLVEPIPETKSTRKRRDLGSIRVLFSQHLD

EDVTKHQKKILARFSISEASSMMEATHFVADSFMRTRNMLEAIASGKPVVTTQWLESIDQ

VNIYVDEDLYILRDSKKEKEFGFNMGVSLARARQNPLLKGRRVFITPNTKPGLNTITTLV

KAVHGQPVERLGRSVLSDDKVPENLLVLSCEEDRDISIPFLERGAEVYSSELVLNGIVTQ

KLEYERYRLFTDNVRRTRSTIWIRDGKGKFQRRRG

>Rislandica_BCP4

MAKVNNDFSAHQITQPIDSLPSSPSDSGEDKDSWLRNCDAEPFENDAWINDEYMETQVMD

IECDNDDFLLCAETQTVDLGFEIGEEPIIEGKSLLDASDGLATQVLDHFDDEVVLDSDDD

NVTDVLDDNCELSDSDDSCTKAETLLSSEENRQDGNEKSKQTTDLDARSNEHGISAGKVP

RFASVRSAAFRASAMAARVATEKIPNSDGSTPVNCHSSGQGATHNSSLENSVGELGNQQC

LPSISVEKRNDLTTGNRTARKLFLEDLPEENCPSVDFNAGLAGLSYIGSQEPGEASQASA

LNLVDKLISESCLEFAREVEADCGRRTEEKSKCVASVKGPQELAKKVSYKVGAVGNRIFD

WDDNLEDEGGGDIYRRRKEEFFGVVSKRRESSTLPREQKRKLIHESHRGVETAVDKRKAH

SDSRLLQLGVTRSRQNIQAAKKNLVNELDEVLGADTQVAAEAIDVLCSGDRGNIDTEASR

LPAKKLSPGDERGFSNGGGITRQSKGTKRIQAMSKDELLKSRLKKASPSPAKACKTNIKR

SSKGDQLDKEGPCGWKRRKVQIASRETRKNLVDELDEVSKESNTRIFDRPEEVDAGPDTQ

MAAEAMNALHSGDGMEIDPEPNCLIGKKFSPEGGISSRGVVTRQSKRIKGIQPVDNDAVS

LKPKAKKARSILAKACEKNMDRYSKNDKVDMPDEVVVSSTEKRQEELSTKHCASKLLKQS

SRDEAEVLSYPKRRRSARISKDQVNEARRSSDPAFDTPVKTKTPPKSVSPICVGDEYHKL

SCKDSSKTNTTREFRSLISRLAEPVSETKSTRKRRDLGSIRVLFSQHLDEDVTKHQKKIL

ARFDISEASSMQEATHFIADNFARTRNMLEAIASGKPVVTTQWLESINQVNIYVDEDLYI

LRDSKKEKEFCFNMGVSLARARQYPLLQGKRVFITPNTKPGITTITTLVKAVHGVPVERL

GRSALSEDKVPENLLVLSCEEDHNICIPFLERGAEVYSSELLLNGIVTQKLEYERYRLFT

EHVRRTRSTIWIKDGKGEFQRRRG

>Crubella_BCP4

MAKANNNFSADQETQPIDDDSNPSSPSASGEDKDSLFGDCDTQPFEDSAWIDDQYMETQV

VDIGCDNEEFLLCGETQAVDLVFETQEEPFVEGTQLLEASDGLAATQVLDHFDDQVVADS

DDDVTAVLEDGSELSDDSDDSCSKAKTVLSSEENRQDGSGKVKSTCALDAWSNEHGISGK

KMARFASVRSAAFRASAVAARFAAPKIPNSDRSTLVNFHTSGQGDTHNSSLEYRVGEVGN

QQSLTSIFVEKKNDLQTGNRTARKLFQEDLPEENCHSVDYNVDLEDLSYIDSQEPGEASQ

ASALKLVDKLINESRVEFGFEVEADCGRRTEEKSKFVPIFKGPQELAKKLSYKSGAVGNC

VFDWDDNREDEGGGDIYRRRKDEFFGIASKGREFSTLPREQKRELIHESHGGLGDVVDKR

RTRSDSKLLQHSVTRSQKNIQAAKKNLAKELDDVREAVVLGNDAQVADETIDDLCSRDRS

KFVSETSCLTGKKLSPGEERGYSPGGVVTRQSRGTKRIQAMSKDELLKKRRKKASPNTEG

SSKDDQLDKEGPSCWKSRKIQTASRETEKNFVVGLDEVSKESNTKLFDRNEEVEAGPDTQ

MAAEVMNALYSGDGREIDPELNNLIGKKLSLKGGISSRGVVTRKSKRIKGMQAVDNDVES

LDTKTKKARSICAKSCEKNRDRYLKNDKVNPPDEVVVSTTEKRQGEFSNKHIMSKLPRQS

SRGDTEALNYPKRRRSARILQDQVNEAGRSTDPTFDTPVKSKMPSTNVSPICMGDEYLRL

SCKDSVTSHTTREFRSLTLPLAEPISETKSTRKRRDLGSVRVLFSQHLDEDVTKHQKKIL

ARFDISEASSMKEATHFIADNFTRTRNMLEAIASGKPVVTTQWLESIGQVNIYVDEDLYI

LRDIKKEKEFCFNMGVSLARARQFPLLQGRRVFITPNTKPGLNTITTLVKAVHGLPVERL

GRSILSKDKVPENLLVLSCEEDRTICIPFLERGAEVYSSELLLNGIVTQRLEYERYRLFT

DHVRRTRSTIWIKDVKGKFQRRSG

>Lannua_BCP4

MAKVNNNFSEHQETQPIDSPPSSPSGSGEDKGKCGGESAFMQDTEPFEDDALINDQYMET

QVMDIECDDEENLLCGETQAVNLGFETQEEPLLEEKQLLEAFDGSATQLLDNFDDEVVAD

SDDDVTDVLEDNSELSESDDSYSKAETLLSSEENRQHTNENIRSTGALDSGSNEHGNSGK

VARFASVRSAAFRASAVAARVATQMFPSSDCYSSGQEVTHYSNLEYSVGEVGNQQCPPSI

SVEKKNDLRTGNRTARKLFLEEPEENCPSVHLSYIDSQEPGEASQASALNLVDKLISESC

LEFAREVQTDCGKRTEEKAKFVPSVKGPQELAKKLSYKSGVVGNGTFDWDDNREDEGGGD

FYRRRKEEFFGVSTKARESSTLPRAQKRELIHESHRGLRVAVDNSRAHSDSRMLQHSVTR

SRKKIQAAKMNLGKELDEVSEESNNRIFDIREVVALGDDTQMAAEAIDALCSGDHGKFDA

EARCLTGKKMSPREDRGVSNCGVITRQSKGTKRIQAMGKDELLKRRMKKASPIPSRSCGK

NIERSSKGDQLDKEGPCCSKRMKVQTASRETKKNTRMFDKHEEVEVGADTQMAAEVINAL

HSGDGREIYLEPNCLIGKKLSPEGGISSHGGVTRKSKRIKGIQAVDNDVESLKPKTKKAR

SIPAKACEKNMDRYSKNDKIDTLDEVVVSSTEKRRGETANKHCTSKLLKQSSRGEAEKLS

YPKRKRSARTSQAQVNEAGRSSDPAFDTPVKSKTPTMNVSPICMGDEYYRLSSFKSNTTR

EFRSLTLPLAEPIPETKNTRKRKDLGSIRVLFSQHLDEDVTKHQKKILARFNISEASSMM

EATHFIADNFMRTRNMLEAIASGKPVVTTQWLESIDQVNIYVDEDLYILRDSKKEKEFGF

NMGVSLARARQYPLLQGRRVFITSNTKPGLNTITTLVKAVHGLPVERLGRSALNEDKVPE

NLLVISCEEDRDICKPFLERGAEVYSSELLLNGIVTQKLEYERYRLFTDNVRRTRSTIWI

RDGKGEFQRRRE

>Mmaritima_BCP4

MAKSKDNFSERQETQPIDSLPSSPSNSGEEAKDSWLGNCETEPFEDDAWISDQYVETQVM

DVECDNEEFLLCNETQVVDLGLETGEEPLVEGEELVEAFDGLATQVLDRFGDEVVACSDD

DVTTAVLEDNGELSDSDETCSKAGTVLSGEENRQDGIEKVKSTCARDGWSNEHGISGKKV

ARFASVRSAAFRASAAAARVESQKFPNTDRSTRVSCHPSRQGAMHNPSPENGVGEVGKQQ

SLTSIFVEEKNDLRTGKKTARRLFPEDLPEENCHSVDCNVNMGDLSYIDSQEPGEASQAS

ALNLVDKLINESRLEFAFEVEANCGTRTEEKSKCVPSVKGPQELAKKVSYKIGAVGNCIY

DWDDNREDEGGGDIYRRRKEEFFGVASKRREFSTLPREQTKELIHESHRGLGVAVDKRRA

LSDSKLLQHSVTSRKNIQAAKKNLGKELDEVHEAVVLGNDTQVAAEAIDDLCSGGRGKFS

AEESRLTGKKLSPGEERGFSPGGLVTRQSKGTKRIQAMSKDELLKKRMKKASPISAKACR

KNIEGSSKGDQLDKDGPCCWKNRKVQTASRETKKNLVDKLDEVSKESKTKMFDRHEEVEA

GPDTQMAAEAINALHSGDNMEIDAEPNNLIGKKLSLEGAISSCGVVTRKSKRIKGIQAVD

NDIESLKPKTKKARSILAKSCEKNMDRYSKNAKVDTPDEAVVSTTDKRQGELSNTQSMSK

LLKQSSRGEAEVLNYPKRRRSARISKDQVNEAGRSSDPDFDTPVKSKTPSTNVSPICMGD

EYHRISCKDSVTSNTTREFRSLTLPLAEPIAETKSTRKRRDLGSIRVLFSQHLDENVTKH

QKKILARFDISEASSMKEATHFIADNFTRTRNMLEAIASGKPVVTTQWLESINQVNIYVD

EDMYILRDSKKEKEFCFNMGVSLARALQAPLLQGRRVFITPNTKPGLNTITTLVKAVHGL

PIERLGRSSLSEDKVPENLLVLSCEEDRAICIPFLERGAEVYSSELLLNGIVTQRLEYER

YRLFTENVRRTRSTIWIKDGKGKFQRRSG

>Tarvense_BCP4

MAEVKNNFSENQETQPIDSLPSPPSDSGEDKDPWLGNCCDEFLQDTVPFEDDAWINDVYA

DTQVIDNGCDNDEFLLCRETQAVSLGFDTQEEPLVEGEQLLGSSDGLATQVVDHSDDEVV

ADSGDDVTVFLEDISELSDCDDSHRRGGNLLSSEDNREHANENVKSTAAIGTQTNEHGTS

GKVARFASVRSAAFRASALEARVATQNLLDSDCSTLVNCQSSGQVSTRNSLLETNVGEVG

IQKCSPSMYVEKKNEGNKTARKLFLEDDSPEENCPGLSYIDSQEPGEASQANALKLVDKL

ISESCLEFAVEADADCGRRTEEKSKSVPTVKGPQELAKKASYKARAVGKGIFDWDDSRED

EGGGDICRRRKEEFFGFASKERKSSTLPREQKRELIHESDGTGVAVDKKRAHSDSRLLQH

SLTRSQKNIQATKKNLGKELDAVFEDRSNKICDMQDLIALGYDKQVAAEAVDALSSGDRS

KVDAEASCLTGKKLSPGEERGFSDGGVITRQSKGTKRIQAMGKDELLKRRMKKASPSPAK

ACGKNIKRSSKVNQLEKEGPCSRKRRKVQTTSRETKKNLFDDLDEISKESNTRMFDRREE

VEAGPATQMAAEAINALHSGDAREIDPEPKCLAGKKSLREGGISSRGVVTRKSKRLNGIH

AVDSDVELSKPRTKKARSVLAKACEKNDKLDTPDEVTVSSTEKGREELSNKHCTSKLLKQ

SSRGEATLLTTPKRRRSARISHDQDNETGRSSDPAVDTPVNSKMVPSKSVSPICMGDEYY

RLSCKGSRTSKTTRDFRRLASTLVEPIPETKSTRKRRDLGSVRVLFSQHLDEDVTKHQKK

ILARFDISEASSMMEATHFIADNFMRTRNMLEAIAAGKPVVTTQWLESIDQVNIYVDEDL

FILRDSKKEREFGFNMGVSLARARQYPLLQGKKVFITPNTKPGLNTITTLVKAVHGMPVE

RLGRSTLSEDKVPENLLVLSCEEDRDMIIPFLERGTEVYSSELLLNGIVTQKLEYERYRI

FTDHVRRTRSTTWIRDDKGEFQRRRV

>Salba_BCP4

MTKVNNFSADQETQPIDSLPSSPSDSGEEKDGCIGNFGDDFMQDTVPFEDDAWISDEYME

TQVIDNDEFLLCRETQAVDLGFGTQEEPFVEDEELLQGFDGLATQVLDCSDEDDDVAVFL

VDSDFDDTSSRGKLLSSEDKSTEHADENIKPTGGHDARETIKSTGKVARFASVRSAAFRA

SAVASQKLLNSDTSTLASSGQGATNSSLLQQNFGEVGNQKCPPNTSVEKKIEGKRTARKL

LFEDDSPEENCPSPGLSYIDSQEPGEASQASALKFVDKLISESGLKFDVEVEDDCGRRTE

EKSKSIPSVKGPLELAKKASYKARGLGNSVFEWDDNREDEGGGDICLRRKEEFFGVASKA

QKPSSLPRQQTRELIHESHRGSGVAVDKRKAHSDSRLLQCSVTRRSQAMIQAAKKNLGKE

LDAVSEEGDDKISDMRDLVDLGYDTQVAAEAIDAVCSGDRSKVNAAASCLTGKKLSPGEE

RGVITRQSRGTKRIQAMGKDELLKRRMNKASPSPAKAFRKSIGKSLKSDQLEEQEAPCFL

KSRKVHTGSRESTKNLVDELDEVSKANNTSMSDGRQEIEAGPDTQMAAEIISALHSGDAR

EIDHEGKKSSRGVVTRKSKRVEGIQAVDDDVESLKPKTKKAKSIVAKACEKNMDSFSKNE

KLDMLDGVVVSSTEKRGGELSNKHGMSKLLKQSSGGETELLRYPKRRRSARISKDQVTEP

ERSSEPAFDTPAKSKEPSKNVSPICIGDEYYRLSCKDSRTSNTTRDFRSLTSPLVEPIPK

TKSTRKRRDLSSVRVLFSQHLDEDVTKHLKKILARFDISEASSMTEATHFIADNFMRTRN

MLEAIASGKPVVTTQWLESIDQVNIYVDEDLYILRDSKKEKEFGFNMGVSLARARQNPLL

KGRRVFITPNTKPGLNTITTLVKAVHGQPVERVGRSVLSDDKVPENLLVLSCEEDRDISI

PFLERGAEVYSSELLLNGIVTQKLEYERYRLFTDYVRRTRSTIWIKDGKGKFQRRRG

>Cmaritima_BCP4

MTKANSFSADQETQPIDSLPSSPSDSGEEKDDWIGNCGDEFFQDTIPFEDDAWINDEYME

TQVIDNDEFLLCRETQALDLGFGTQEEEPFAEDEQLLQGFDGLATQVLDPSDEDSDDDDV

TVYLVGNNEVSDFGDSNTRGSNLLPTEDKSREHGNRNIESDGRSNGHENIKSAGKVARFA

SVRSAAFRASAAAKKLHNSDSSILASCHSTGQGATNSSLLQQNFGEVGNQECPPNTSVEK

KNEGKRTARKLVFEDDSPEENCPSPGLSYINSQEPGEASQASALNFVDKLISESCLKFDV

EVEADCGRRTEETFKNIPSFKGPQELAKRASYKARALGNCMFDWDDNLEDEGGGDILIRR

KEDFWGVASKSSSLPRELIHESHRRREVSVDKRKAHSDSKLLQGSVTRGRKMIQAAKKNL

GKELDAVSEEGYNKISDMRDLVDLGYDTQVAAEAIHAMCSGDRSRVNAEASTGNKLSPGE

ERGFSNGGVITRQSKGTKRIQAMSKDELLKRRMNKASPSPAKACGKCITRSSKSDQLDKE

GPRCLKSRKVHTASREIKKNLVDELNEASKESNTRMFDMREDVEAGPDTQMAAEVISALH

SGDAREIDHGSKKSSREGGISSRGAITRKSKRLKGIQAVDDDVESLKPKTKKAKSNPVKT

GDPKKDSYSKNEKRSMPDEVVSSTEKRRGELSIKQCTSKLVKQSNRGEAEVFSYPKRRRS

ARISQDLVNEAERSSEPAFDTPPKSKAPSKNVSPICIGDEYHRLSCKDSRTSNTTREFRN

LISPSVEPIPETKSTRKRRDLGSVRVLFSQHLDEDVTMHQKKILARFDISEASSMTEATH

FIADNFMRTRNMLEAIASGKPVVTTQWLESIDQVNIYVDEDLYTLRDSKKEKEFGFNMGV

SLARARRNPLLKGRRVFITPNTKPGLNTITTLVKAVHGQPVERVGRSVLSDDKVPENLLV

LSCEEDRDISIPFLERGAEVYSSELVLNGIVTQKLEYERYRLFTDHVRRTRSTIWIRDGR

GKFQRR

>Iamara_BCP4

MVKVKNNFSSAHQATQPIDSLTTSVSGSGEDKGKCGDEYMKDTDQFEDNAWINDQYMDTQ

VINIECDNEDFPLCVETQPLNLGSEMPEGNELLETSDGLATQLLDEIDDEVVADSDDDVT

HVLEDNIEHSDSDDDSCSKTETLLSSDEYRQHSNQKTKSTGVLDSWSKDHGVSEKVASFP

SVRSAAFRASAMVARFTSVRSAAFRASAMVARVATQKFPSSDCFAECSVRDVGNQQCPLS

INFEKKHDLQTGNRTARKLFLDDSPVENFPSVGLSYIDSQEPGEESQANALNLVDKLIGE

SCLEFAHEFQNDCGRRTEEKEKFVPSVKGPQELAKKLSYKSGVVGNGTFDWDDNREDDGG

GDFYCRRKEELFGVINKARESSTLPRARERELIHEINRGLGVHSDSRLLQRSMTSRKIIQ

AVKKNLGIELDEVLKESNNKIFDMPEEVVSGNDTQVAAEALGALCSGDHEKFDTGKNLSP

VNDRGFSSCGVVTRQSKGTKRIHAMGKGEILKRRMNKTSPSPSKGSQLEKESPCCSKKRK

VQTASRETKKNLVDKLDEVSKKSNTRMFDKHDEVEVGADTQLAAEAINALHSGDGREIDT

EPNGLIGKKLSPEGGISSHRVVTRKSKRIKGIEAVDNVVESLKPKTKKARSILDKACEKN

MDRCLKSDKSDRLDEVDVSSTKKRRGESASNHCTSKLLKQSSRGEVEVLSFPKRRRSARI

SQDQVNNEAGRSSDPAFDTPVKSKVSSKNVSPICMGDEYYKLSSFKSNIARELRSLTSPL

AEPIPETMSTRKRRDLGSIRVLFSQHLDENVTKHQKKILARFNISEASSMMEATHFIADN

FTRTRNMLEAIASGKPVVTTQWLESIDQVNIYVDEDLYILRDSKKEREFGFNMGVSLSRA

RQCPLLEGRRVFITSNTKPGLNTITTLVKAVHGLPVERLGRSALNEDKVPENLLVISCEE

DRDICTPLLERGAEVYSSELLLNGIVTQKLEYERYRIFTEHVRKTRSTIWIRDSKGEFQR

RCG

>Chispanica_BCP4

MGKVKNNFSANQDTQPVGSLPSSPSHSGEEKDPWIGNFGNEFMQDTVPFEDNAWISHEYT

ETQVMDNECGTKEFLLCCETQAVGSGFGTQEEPLVVEEQLLQGSDGLDTQLLDRVDEDVV

VADSDDDDVTVFFENNSERETSDSGDSHSRGGNLLLSEDKTRELANENIKSTAAVDAMSN

EHEKICGKVARFASVRSAAFHASAVAARVVAQKLFNSNCSTLANCHSSGQRVTHNSCLEK

NVGEVGNQDCPPNMSIGKKNEGSRTARKLFLEDDSPPCPGLSYVDSQEPGDASQANALEF

VDKLISESSLKFDDVEVEADCGTRTEEKSKSIPSVKGPQELAKKVSHKARAVGNSIFDWD

DNREDEGGGDIFRRRREDFFGVSSKAGRSSTLLREHKKEIHRELGVAVDKRKAHSDSRLL

QRSVTRGQKSIQAASKNLGKELDAVSDEHNKKISDMRDLVALGYDKQAAAEAIDAVCSGD

RRKVDACLTGKKLSPGEEREFSNGGVISRQSKGTKRIHAMGIDDLLKRMMNKASPAKACS

KSIEKSSKGEELDKEGPCCRNRRKVHTTKKNPVDELDKVSKESNTRMLDKREELEAGPDT

QMAAEAINDLHTGDAREIDNEANSLIVRSLHGKESFLVVELLPGNPRDLRGFRLWMMMLN

RRNQKPRRLNQFLTRLVRRMWIDMQRMRNLISQMMLLCQVQRREMENYQTNIVRLSYLSS

QVERRRSARILQDQDNEAGSISGAAFDTPLKSKASSKNVSPSKAPPKNVSPICMGDEYHR

LTCKDSRTTSNTTKKFRSLTLPLVEPIPETKSTRKRRDLARIRVLFSQHLDEDVTKHQKK

ILAKFYILEASSMMEATHFIADNFMRTRNMLEAIASGKPVVTTQWLESIDQVNIYVDEDL

YILRDSKKEKEFGFNMGVSLSRARQNPLLKGRRVFVTPNTKPGINTITTLVKAVHGQPVE

RLGRSVLSEDKVPENLLVLSCEEDRDITIPFLERGAEVYSSELLLNGIVTQKLEYERYRL

FTDHVRRTRSTIWIRDGKGKYQRLRG

**BASAL DICOTS**

>Ckanehirae_BCP4

MGSSDNDRDHRTEQRNPIFPNNTETQPIDDPDSPFSGVKFNDEGMQFHDTLPLDSELFET

EVVREVNDIGTETQVLEDSDSTAGEGAEVTEVLSSDGEDQLSDGGTSLCKVKDSTAEIGI

KDGKGGSEKRKSICSNDQPTKDILVDSDASTEDENSSGCAQRSFTSVRAASLRSSGIAST

LSMTPKRGELNLAKINSEDGPEQKQANGKSLDHSTEGIRKDNGTASFRYPTFGSMEVCAL

DHDHNGEKLHSKTQDNVGDETKTRIGKTMARKLFDEISPSGDEEMTSKANSPTLGRDSPQ

LLVSDHAIAGLSYVDSQEPGEQSQANAWNIVDKFLSINDVQLSGEVDHGKTDREKSPPMS

SAKGAKSLAERTDFKSPVGQAGVFDWIDSREDEGGGDFFSRRKNVLLEGSGKMLKSHTLP

PKPKHRTFRQIQGAVDGFEEENASPQVHHKINSSTRSDSGSGLVLNRSTTKSQTSRTITK

KNLLKEMGKALKTDVSGQKLETSVTCSGVQGICDVGVDTQMAAEALEALVCDLPPNHDVP

DAHLLKRNVTEGSIGAARKNTSLKRVCVRKKVCSSSDSEGKARQCKQTKKLSAKLCRGNP

SSQKQLKHSRAKETRGKRKQEENLNTGKSSNENVFSNGKSPDTVKKRKTEQELAGTHIHG

ADEHHLSSMSKKCVSFRVLDGTVTPVAKRTRKPLTTNSLKMAENFSNHSHPMEVSILGSK

SRGSGSVIDLPEPVTVKDKSLNFFADQVGKFSNNKTSRQEIKATEGLNEKIPRTGYMTGD

FISYPRGRRTRRRMPRECMHPESDHNTLSSGKLPKTSDFSVMNEKIPLKDQITPTSGTLT

RSGRKYGADYSLSSVKDAGGNDTIGQSNGKVQTSGTPCTTPSKELNAVSPVCAGDDRTEQ

SGRKILSRSSLMRELIRLGTQEVARTPAWKDMRRRRDMASVHVLFSHHLGEDMIKQQKKI

LARLGVPIASSSSDATHFVADKFVRTRNMLEAIALGKPVVTHLWLESCGQASCFIDEKNY

ILRDTKKEKEIGFNMPVSLARACSSPLLMGKRVFITHNVKPDRELIVNLVKAAHGQVLVK

TEETLVDGKVPSNLLVISCEEDYALCAPLLEKGAEVYSSELLLNGIVIQKLEYISRHRLF

MDHVKRTRSSIRSSEDGGRIHPKTKHK

>Atrichopoda_BCP4

MGGEREKGRLSIDSETQTISIDSPLDGGVFIETHDSDTCPAENTLVLDDTVSLENEIACE

DPDHIEDTQLVEDPNCTNVTTLLVGESEEGICVGSDHEETQRTLVLIDNEGVLGEEGSIP

LKNDGYKRKCKEGGNLSVVNATLENDWDRRNNVVDSDASTDDEVASGNSISYGSPCRQPA

SMPKSGSSQRSFASVRAASLRASGLSAAQSMGLKRPAYGTGSLNQEGLNALGDQTRGKAG

NPRVRKLFVEDELAENGNSIRKFNSLGDEETNSSLLPGHCTAGLSYLNSQEPGELSQAEA

LGVVDKLVSVNGVGLSQPVEPGKTGDMKAALNSSVKGAQTLAKLADLRNPTEKIAIFDWD

DNREDGRGGIFSSKRKEAFFQNGKTVDKALTEPHKNKRALSKGPKGLGERLREKEEERFS

YVHQGVLNFAHSDSRVVLSGSSSGHYMRCAKISRNLFKDLEEHPSQVPLGQEPASPVRFS

QGTYDVGFDTQMAAEAIEALGCVPPVTPTNQKALTNPTKGLRKSNSRSKSKAGSKAKGKI

GSDSRRVSVRKRGCSSIDSDYNLRKSKQMKTLDDKSREADHIGFSRCRKKPIAKAVEPKL

TGEREANQQKFEQEERTAKLSELGIRERRKPQLEKKEKRSFDTLSNEKPAGTNLLKETLS

SYPKTRAGKRNSSERNLNSLLSVAGCVKNLEAVNQSSSARRKRSTNAAKTTHSQTCVPNK

DSIEEIFLNPGTRKTRSASKSSAPLITVSKDVPTKAREATTHLSPAERDSMDGISCLPSE

IDGIDGVSDVPIKSSVRIRSSRNPSSRLLSPNPTSMTRQQTPEHSFYKNAANHSAALANK

DQLHNILSATTEKKTELAGSVCSTPLKGTDAVSPICMGDGAHRSFTRDPGKSTLRRELIR

LDAAEPEVSPTLKSAGRRKRYMSSVRVLFSHHLDDDIVKQQKKILARLGACSTTSSPSDA

THFVTDKFVRTRNMLETMALGKPVVTHLWLENCGQASCFLDEKKYILRDTKKEKEIGFSM

PASLNRASQSPLLQGKRVMVTPSVKPNRDLVAYLIKALNGQVVERLGKSAIKDDKILKDI

LVISCEEDYIFSAPLLEKGARAYTSELILNGIVIQKLEYERHHLFEDHVKRLRSSNLVQS

RK

>Ncolorata_BCP4

MGRAKSRRNNRKQSSSTQIVEPESPPAGENLENGKIKKTVRALENQSPTEILSGSNNLGD

TQLVDDAIGIDEETQLVDNQYTSGFADVEHDTELGTQLVDESEKEDCSDRDSNGTEIIGR

LSDDEGVHGIVSADPNEKVAAASRCLSKHADKCLSIDSDTSTGSTQANSPSIYTSSLYTI

PNQTEADLLSDMHSTEVELKEKQVRNENACKDEICDSKARIFFDEDERNKLLFDNTLEKI

EDVHSSKLGRGERVPELSYICSQEPGEFSQANALGIVEKLLDTVDGQLYVETVPKRSIGL

KMASMSGAKGPQSLARRQDLKNPMESIGTYDWIDSREDEGGGDFFTRRRESFFGNGHGRK

SFTEPCISKKLTSGKAENLAKKFLENQKGLPEDHKSSMNISLSDSRISLSRSTNLDIGQV

KNAKVIRSLFGNLKKQEHTERLEKLVEPPDHSGLIVGIHDVGVDTQMAAEAMQELGCRSP

FNSVCEDIPVISQSAEKESKRFPDSSYLFKTSEPLFTSVNTEELSKQMKCGTRVGGKKYH

ATEKCPKNSKDSIVEKRSITKRSAKQRCNTMNSARRRKNVSVKLSKCLKGEEDSDETWLY

GSSGSVIDLNRSIEPVAHRTRNSIGKQSDKSENVFRNCEDGKSDQLDARITRRTRSSLAV

ASKRQRPVSSSIPLSRHMLNDAATTTMNKMQQANEEKKQLSSQKKKASACNITSSTYPKQ

VAAEQMTSGIRHNVKVRRQSSSTNASSPPNIGQITIHLNGAGNLDGGASAPCFPCNGEVS

IKKRRPSRCPSAHASMIKKPKDDFVHQKGSKFDTASTGGYAPANVLNEKSPSALQALTTS

NTSHCSMNGAEIMKKDKDQFSSVPTTPLKDVDAASPIYTVNGDVTHCYKTRSSMCSLSRE

LIRLDAIKPELSPILKDSPRKKRDIASIRVLFSHHLGADVIKQQKKILSRLGIYTASSSS

DATHFVTDKFVRTRNMLESMALGKPVVTPSWLESCGQACCFIDEKKYILRDAKKEREIGF

NMASSLVHAGQKPLLQDHRVLVTQNVKPSKELIITLVKAAHGLVIERVGRSPIEDLLIIS

CEEDRSICIPLLEKGARAYSSELILNGIVIQKLEFDRHLLFMDNKKTRSSRTFKS

>Ltulipifera_BCP4

MGFVGGDEEGKKSLEDGETQTLALESDDDSPRPSGEKFKDGGVQFNDTLPLDSNFLFETE

LVEEGADLDGETQVLEDPDCIVHDFHGETQLLEDTDCIVHDFHDETQLLEDTDCIVHDFH

GETQLLEDTDCTTDECEKEVAVDSDTEGTERTEVLLSDTDGVADDGTSSLKVNGFNGERE

MGDRVTHSEEPYIPPCLYDQGGKDLVVDSDASTDEECSPVRQVSSTSPHVSATGSNPGSA

RKSFTSVRAESLRSSGLAAALSVTPKRTDVEPNPTLSNSGSERKQDNTKVPNPGTHGIKK

DDGLPGLSHQTPSIGIEMCERHQDHAADDCHRTRNARVRKLFNESITAGSEEIINKVDDR

TKKSDSSQLLVSSHGIAGLSYVGSQEPGEQSQANALDIIDKFLSINDLEVSGEVRPSKDL

GGKSPRISSAKGAQSLAKRSDLRSLVCKEGIFDWIDSHEDECGGEFFSKRKDALLESKQR

IRKSHTQPRKPKRAEFKITGDAVDKFGEKEEGTITQIGRKIIGLTHSDSRLILHRGDGVQ

FSEAKTKKNLLKELEEKSNAEVSGQPLEKTAIGGSPGLYDVGIDTQLAAEAMEALVCGPA

ADHDLVDVHPVARNSTEGSSRNATKNKAHSKHVSVRKRACSTSDSEETTRKPKQTKSTKF

RKGPNPFRKHSKDSSTKSSGKESAVKANSKREKRKHEEEHFTTRESPNGNECLGGKSSKS

IVRGKTRGNVDVTHIKKVASSASNGHALIEKGLLQGHGTVTPIASRTRQSKTMDQLKKTR

ISYNHLPVEDGILGSKTKGSNFVSYPKGKRTRRNMSDYLNNAANLNGLPIATDAVAEVNA

QSSAQQKRSKTDIGNYFASLDTVKRKTRSSDTPMPPSLLMGKDMLPKSGDAASCLSVNRM

AKIDGFASGPCMPSETDQTGSITVVNEEIPTGNLISPTSGTLTLAGQKYGVETTRTLVED

AGGNASLEGSPKGKVQPSQSAFTTPSKDMHAVSPICAGDDRQKQSSKKGLSRSSLARELI

RLDASEPARGHLKDMRRRRDMASVSVLFSHHLGEDIIKQQRKILARLGVSTASSSWDATH

FIADKFVRTRNMLEAMALGKPVVTHLWLESCGQASCFIDERNYILRDTKKEKEIGFSMPA

SLARACHSPLLQDKRVFITPNVKPGREVVSSLVKAVHGQAMERIGRSVMKDDKIPDDLLI

ISCEEDYTLCVPLLEKGAEVYSSELLLNGIVIQKLEYERHRIFTDHVKRTRSTIWLRNED

GNQFHPVSKHM

**NON-FLOWERING LAND PLANTS**

>Mpolymorpha_BCP4

MDADTQLIGYSPDASHSGESSDSEESSGSDSPSNQPTQLLYPQLSDSYDRYGEDMDDQIR

TQQLDAMDMATQLIDEDFREHKGMGDDCTMIIHSEDEASKDTQLVNADGDSGASSGKDDE

ADSALSPYGAAFKQKTSHWLSEMAGKRRAENASTFLTGEKSAGLSYDVHGNRFNSMGSEP

ASNLHKCNNTEAGLGNPVKMQNRDSELPHFLKDSDLNKQNYGSHRMVGTWHEIVGQRNRC

NEDERVWASAVGGLHLQDSDGSTEDERREEDDKKCNLNSSSTYHVEGNLSQSMRGRLSAY

SSGNFCQQDLNKSPEPETLMMDEDWREVNGEDLGLIGRIDEGGSNVGLLDKPEVALISQT

TPVGSRNSEGLPAEPRSLSKLHSNSRTSGSIRTLSLRASALRASSCSSSARYRHTPQGSQ

SQEVHKLETEATNETQCEEPWKTAKSADYITPTSRPSIQKGVFSSAASVASVWSNTEKAG

EYSRDAEKEMLSGKVRDHIEGDFSGDKCGRSGKSVLGLEVPKFGDKNLCRARKLFSGNDN

DADDIFSQVKVENVQKSPSPARDDGAAREKVEITPPTITDSQADKTLERRSSFGSGVFSG

GSELSYLNFQSPGEDSQARALNMVDKLVWLNAIGLPQELQLEEEKAPARCPSVAKSALQL

RVQVAEAKVGMNDPLGVFDWVDSQNDEAGNFFGIKGASALKGTSKAPVAMGHPKQTGFGT

RKSVFLDAYGLRNKKMEKVSDKPFKPAVIADRPKAVVHKNMSGPKDNKGEQHGKLSRDAT

NAGLTSTLNEPGLVQTSLHVDAPRNVIGNKMLQDNRDNRGMKNVEGDTVISAVDISRLDL

TSNAAVQRSSGLLGGHGSEGLPKGVGPQDSRQAPRCLEQPPKTPDVHKGSGKTVSDKQAD

EALSASKGKGSSDLPHVPSKPGQKGKSLQTVISPRDNGPEGFQEPAASRKNEKAESTLGD

GLSELTHSRQDSNANQQAEGRLTGLTGFPCLNASKVLIPSTKDQLGGGLTTTDLSDVPCC

PNRPVDIGNQAHSTEAEVQPQKLETAAGEDKLGEARIEVDNKDGKQIELFLKFDGVLEND

CMDCDVDGSIGARGSVSSTLLSTDKEGEDEKEAEENAEVVSVREIHMGPDTLGAVEAMEM

LSSDCLASSGDGSDEPATSKDPAGSPATVHPVSSDDKTTGLNTETHDGVVEGRRIVTRRS

RTQGRQSTRFESTDPINSFRDVKMRGRNRRVEGTDPPIGKYTDPKDRARNRRVSWAGPVE

QVPRRPVQVRRGRSARHQQHVCTEEERADNARQNSDHEEAEDVKDAGNGDRKRKVEALEV

AVSKDTDLDKIKRRFNMSDAAVARLLGTDEDEDAEVNLSPGVKIRSLSHMQPPEAEENEN

VDAPKVRKAGKGLRSSRSRGEDGSINGRRKRACTTSSDLEKQGGDEMKGSPCDTDAADTS

VDGTACVEAHPSDTEALPGRRRMRSIADVSSPVKKTLPGRPPQGSGLRRGRGRRLPRAPG

EQKADPSDAEQVEVPPQPPPPPPPPPPSTTPAFPSISGRLRGKGGPRSARKRKELEPLTV

EVSATSPTSSKEASTTSSKEDSKRRKGEAISTCVLFSHGLADDVGKQQRKIVKKLGGRVT

TSAAECTHFVADKFVRTGNMLEAMAAGKPVITAEWLDNCLEAQGFVNEQAYILKDTRKED

EMGFCMVSTLNAAQQRPLLQDIRVYITPGTSPGPEALARIVKAAGGQVVDEYIGPAPDGV

RESDYCLVLSSEEELETCSPLLEQGAKVFTPELLLSGVMCHKLDFSNDQLFKSFNNRRRR

ASLSRRTTL

>Aspinulosa_BCP4

MIYDNLSVMQTPKTALVGGVNDEHTPCVSTSGSRPRNIGSVRAASLHASGLRAAARTASNGSVGKDITYESDVTVKLTPGVVNMFEKPYHGLHGMGAQCNARSLDAGQVTCHSPCMFDSTARSQSTAFAWVASLDSSGISAANLSMEKGTVVQFDSHATMELTHEIVESLPASKKLKSVDGNNQQHAGISPLNQAKEGGYVVDADEKCRSAGRFRQPVGQKVRKLFINFKETSSASGPGTTRPATDKEVQGCDMSSLGLQAAEGGTNDAQKENTNPTPVSGGEKTQGLNQQSTPPSTIFMPSSANLSYTESQEPGEDSQLNAFATVDKLVWLNASAMWQDPEPKTVAEQSSVKSSQGVKGLQSLAHTTEIKNMQDKMGIYDWIDSQPDEDGLSSDLKEVFAPVRAQQQQLEACKPTKNRKMQQQGMKSSLDNANDSNSASVMAKGTSCIKQTTASNVRQTTYDCAMKPHTWTAQLRTDKGKPTEELSKCFEHEPYSPVRDNVTDELYDADVSSHKGETEIIIPSDISANTQIALEAIDIMRCGVVMNDENSHQDERERLFPFDIGISTQSAIEAMDIMRHGVVTDAVFIEREEREENQTDLRIQQLSRDAKKCVTPQGLAVNRGEKDVSVKTGGDLPAKSKPPPALITISRKGGLPLGVPVNRKPVCKRETSGGFSASLETHRSQKAWKFLPKPRGLVVNSRKGSPLDFLSAVIPRRKRSHLCAPVGSLNGNMVVLQRQDLEFDLHKSHQNSHEQTQQIKVSSLEFRKSVQPQKNDDNNTKLAVLAMPTKRKLSRQTKKLATLEAACKPDFCALLEERTKRRKREPGCIRVLFSHSLLEHTIKHQKKILAKLGGHLATSAPDSTHFVTNSFVRTRNMLESMAAGKAVITTLWLEGCSQAHYFVDETSYILQDVKKEKELGFCMHSSLAASRRKPLLKGMKLFVSPNTMPDFEAMRAIIISAGGQAEASDSRVFTLEVWLVVRLQIWEGAFCCELVFEYKIACIGVRAQGCGLDTCTVSLIPLGFMEQSKLDGSSYLLWKVKIRSQFVAQKLWKIV

>Itaiwanensis_BCP4

MDADTELLDCNFEETCIADTPVTCEVGEGEEAEAGRTLQLDTEEECANELVEWEKQMGVNMSSYYNDNSSHSGGSGDTVVLPDAKDEDAEVCDSTLRQELHPEMEPLNASNKAEKMTEIPCRYVLEHGRHSLAEGNMITKACLAWNNHSCTTSTGLNEINNDIIIKDEGNQGPLTMASSKNISSLKQNIDDGVWPLNIDHGDDDEIKNYPSDDLSRKTRRLIFATPELMPLQSSSQSTNLRKPVISSLEPELSMKQGKGFHVPPGNDMDYLNSQEPGEESQALALGMVDKLVWMNSIGLSQDPNLEVESDINLIPLEKSINPGMKSIVQVAEANGSANGLTVFDWVDSQQAVEFHASKENNHPSKETQATYSPQVQKSKKVRHAFVQRRTKGSPPGVKAGKNNLKQQNKTGGADLIGKEEKTRGSMKWQEVPEGSDVLGKNIHQLLHDRDEDKHASADNLAQQVAISPTNGETMLDYFENQDYPSKHKKLSPVERHGGGQGEEENVPELAIAEEVEIGMGTQIAIEAIEIMRHGSCVHVASHAPTLNQSASLPSPPSTQAAKSNNLIWGNRTRSSTTRSKRECSIANDFGKNIKQVSEQSEAKQEAKNCQVEEDINMGKPVRHSNKNDTFQIQSSANVDETGKEEKKHSCVEQILGRKSGNKSASKTMIQERSKSFLATTETLGNSGTFLMKDSTRRSARISALKPGNCAPEEITRSTKSRNFADCGLEARLESGKTVDKCATAQSTEKRAALLFPVIPRRARGTVPIKERNRITGSIRKDRSQEPSLLPETPKQKVITPKELPFRVSRSGQRRQHLDIVKLSNAPVPSATETPLTSSGRKRRKTEMMYVLFSHGLSSDIVKHQKKILMKLGGNVANNVSDCTHFVADKFVRTGNMLEAMASGKPVVTVLWLESCGMAGCYINDKPYILRDLKKEKELGFSMPTTIFAAQQRPLLQGMKVYVTRNAKPEAISSIVKAAGGEVTTRYSATCPCLVISCEEDRKLCAALLEKGVKVHSTELIFNGIVVYRLDLSKYILFSDHTGRRKAEHKKR

>Smoellendorfii_BCP4

MGGDESCDDDFQPTLILGNDTQQDAHGDGGGGGSGDCVADARGDGVEKALSDGTTVDDEE

AGAGGVQDTASSKIQERDDEVQPTLILGTQLLESPQYESTQIFGLEHAGLENPVEEKKAK

SLASIRVQSLRGSAMAAYERLVSKLEPEKDPQGKVEVDDSAKFVLQWGMKQAKTSTIAFD

VFKPAPSLVEENLVTSNGGASCSASTPVSQTLKNISLVPALCSNNKKSTENRDKKSDSAS

PRQQENSLDKIRKVLFDGKSVPGEDCCHPVQPLSAIVAPVRELSYLNSQDPGEESQAIAL

EALERLVSTNGDFSQEAQLGDFDVENKKMNVLASRIVQLQRDDPSSVYDFVTSQEDPLMK

AGGVPAAEGDQPAQVLDEEKVSEALEEQGKTPLSVAMGSKPSTNKRKTAPPRSTARGKRR

RKASPRRADQVVFRAPQVDPQTPKSTSNDAVSAEAVTKGSKSTARKRKSEAEKLIIADDT

PRRSRRRPVKVLFSHSLGKDTIEEQKKVLKKLKFQVVEKAAECTHFITDRFLRTANMLEV

LAAGKPVVDLAWLESCSVAGCAVDEKKFIVDDSKKEKELGFSMHSTYISAQQRLLLEGIP

MHITPNTKPLDMVESVVKAAGGKIVKKNEATGGCLVISCEEDRKHCLPLIKKGIKIYSTE

FLFSGVVSYHLDYSKNLLFAA

>Cricardii_BCP4

MNPEEMAHATLPDSGTGENVAQHETPHSNFSTETNASKGSQSRPSSISSIRAASIRASGLRAASRSVSSNSDRPDDFKSDPEVTMDKVPGTLDIESNKTSRNGSEKEMCGVEDLGIRDTTKFNSVESSPQLMVPCHDRGADSLCGPATRLTSMTARQNCASLSQTLVFPDNRHNEQDDQDNIRPDATILLEDKIQCKSASHGSSSLRLQHSQSQTSVVSTKLLECEVHDDAIRVPDFDLRVQAGLPSSQKARKLVFDNMDSDVGGEQNATSKSLDKSHTSIPSKQMSDNHTLTNKQRNFFLPGTRMASTNLTYLDSHEPGEESQLHALGIVDKLVWLDTLCKPEDVATSLFPPEGSLQPDTGVKGLQSLAQAAQLKAVRNKLDVFDWEDSQADEDDQTVSINARKRSSKAQETSQRSKKQKISRQPQLASERKGIQKNMISTKQELSNKKHLARDIQIIPQAPETKPHTSLGTRLRRRPCMEANASCKPRKSARVQKRLKKEDDTMKQVEESSKDLNIQDQTPANDNKRKVQQAFSEPRGSKDDVETDRAKVTMGKESTLRLVDSSAVDTELQCDGRLSLRTEEDDAAAEMWIEAFSSAENKKPLKLELVKFRRQSTLQDDIANGGKKYRDVEPIARTEHDQHFETSEQENRLKVEEMPGTGVLIESEASAKVIKEEQRSTKSHGLKEISVSKKANMKQGSKKSSSDRFCLKASMRNLESEDEVTLKQKLSLMEEDGAGKELMAQEEEVVGRGPKTRSKSQKIKIQALSIRGKKRKQAAEVRIKVDTSRDTDSKEKEPSLLTETTQDDLTREKSAARSGRSRKRKADKEREAIIEDDSESVQFSEGIAVEGPKRNGNGRMDSDASMSDSVEAHEKSVVSRRTRNSASRSEVQIEETVKPQEQKSTSDFAIEHQQVGNEVLESCGRSIMSERKKKGKVSRIMQNLSPIIVDEGKRRTRDAREIRVLFSHGLAKDAIKHQKKILAKLGGQVAASAMECTHFISNGFVRSQNMLISMAAGKTVATTLWLESCGQAHYFVDEKNYILRDDKKEREFGFSMVSSLFAARRRPLFKDFKVIICPKTTPEPKALRAIVESAGGQVKGIETKIPWSDVDTQASLIVIGNQKSLDVCSQFLVKGLKVYSEELVLDGIVTQTLDFTRYLIFEDFNKRRGLRNAVNSKATV

>Afiliculoides_BCP4

MHAKPEEEREDEYKEYEHRKDADNACLETTTKSQFKGRKDTISDNNVPRKSNFHDIDSHN

YEEHNNVLTNTQKIEDSSDKDSPSTLYASGSRPKSIGSVRAASLRASGLRAAIENARASS

SSKIGTEFLRTGKEDYRIGSVATTHKLTHNGVNDELEKQSFEFVGTELLHDTCQVCDIPV

TQTPPKITMGPDNANNFSPRMSTSRLKLNRSSTEFSQSKSRQKARKLVFDSKEEMPVFSN

CKLENSDGKVESTSLQVDQKNDMNLTPLEQTMSLASGINVSTNLTCLESQEPGEESQRNA

LDVVDKLVWLDVTCTPQETVRNNEPVLLSGEGHLGTSGLKSLAQAAQMKALHEKMDVFDW

IDSQAEEESPLAAKDISDKDKKHSKQVGASKLIVCMKKHFSSRKINQNGNYRKIAEPNIR

VENAISYPTVVQSSAITQASVKNMEPSAEQGVKMHTLLTTRLKSRSRLESPSRKLRVASY

GKRKTVSRKNTIESERLSARLLQKQKTNKVIEVKEIIEITPSERTKVSNESELGCVIQTS

REAVVETDVKKPVVMTLRKRNSLSDDTTQILKKNESIRPARTIKLGGRKVSSDRRNGDAP

ENLLKEKEKIDMMRRGPKTRAKALDAKSKMHNKRENSIATKYNPEIRMSTNGKEETTVIE

CKRKRKAVSECNVQAFKCQKTESDASLPKSKILRSNKLKSVDIEAPILCKEINDIINKNV

VQKPTGSGKKLEVHPEDKEETKGKKTNTLNSEKACQGHKGKPNTRGNRRTPISEVVSNLD

SSVFLDERRRRKRNPSCIQVLFSHSLPEEITKNQKKILTKLGGHIATSASECTHFVSSSF

VRTRNMLVSMASGKVVATPQWLESCSQAHYFVDEKNFILRDEKKEKELGFCMHSSLAAAR

RKPLLKGLDIVVTPNTTPDPEAMKEIIETAGGQVVQNTSDFLQSDASQGGKLVIADNKDY

DYCIQFLENGFSVYSTELILHGIVTQHLDFSRNQILQRRNTRKVGKTNIKHK

>Dcomplanatum_BCP4

MEDPTQLVYAFLESSTRRKADTSSHNKALGSHATDKTQVLASCSMEAVNEEGAGQTLLLE

SQDYRSMSSNQVQDCSSDCVSGDTQILSDSDDELVDQPGTSKEGKSVYELWKRKRSALNL

TDQPSMAGKGDDISRPVAADFHQADLGFDPVAKLVEVVASDASTENGEDAEGNQEVVDSD

ASTTSGANNPAGPKEGAGVVGEWTSGLNLMELLPHHESCDAMKDACLEKFEENGYQCTLR

KDGSPVNASQSTIKQQQTAPLPSNEVAFSGNHMSEDGPDIDRQVGRANFGSPCTATSKDR

LRNIASVRASSLHTTYRADERATKFPFSQGRNDKQEDFQRINSASEPGRSDTGHFNCRAD

VGQSYIETSPAADGSRQLCIDMPPVCSETKKSVDIKKLSGPEVDVNTVAAVNIQTQAEEC

RGKEEFAGQGRKVRKLLFEDGEIVQENSAQHRQVSVLLESDQIVHIDQMQHELEEKHDVL

DSKLHSKLNELSYLDSYEPGEESQANALAMVDKLVWLNTSGCSQEPVVEKDDRTEGALSK

KRWHQSAQVEENKNSKETMKVFDWVDSQFNEAGFTNKVQAEKSKRINYGECNELKGSGLR

NKKLKKLVRQDKVAKRKPGSLKQDLEVVEGANNIKPRNPSRLRSSNVSTKKMNCSNSFEK

VTAVDVKVCSDPKAYPGDGGSSIPIQNTDSTFSNSVQLLRKSPQGGLAQAVSKVAQRSLC

DLRNTEEKTLHESKLDNGAKEIYKALEHLPISSRITNECLSYSDIGSDTQIAIEAIHIMG

RACEPENKFRADQVDTDKSLQAVSKDRVTQSESRLIEGGKKGRRVSWSGDIEIVSRDRST

PRRQSVSSKVRSDPKIKLAKNREVKSRRVNAKLKRLESKTSMGHVGKTKKSAKLDSYCST

ETMRTSTIKAKKEQPSSKPSVVTGGVSTRSARHNHNSFEIQGGSTSEKTVKEKSCCPSII

RGKSVGANLDGDKEVKSILKRPRTAMMQKRSTMDSSAVDLSSPLSGHEKKVRAVVSAKEH

SDEGSPQQKPLGFSKMATRSQRPMSNLKKVAAPFSGSKSTATNTRQDNEHKSLKRESIVP

KEVSPSEGVKLRRRSSSSSKLCSISDTSQSITGTRIKGEQPKTPARVGARGELSGKYSKV

SGNNKPSHSSHKLLAPTVVEDASTSKSLPKKRFHFLKKRKASDTVLDTPCKSAAISRDAI

NGSIERKRRKREPGNVNVLFSHGVFDEIVKQQHKILKRLGGRVTTKATECTHFVTDKFVR

TGNMLEVMAAGKSVVTVSWLESCGLASYFVDEKNFILQDSKKEKELGFSMQSTLAASQQK

PVLQDWKVYVTPTTKPEAVRAIVQAAGGKVVDDYESYISVKDDGDDFLVISCDEDYKLCV

PLLDRGMKVHSPELLFSGIVVHCLDLSKNLLFIDHSAAKRGRRKNH

>Scucullata_BCP4

MTCRMLPLLSPLCRTQEPAHNRTFFFRNKLTQTMPEKTNSQDPFIRDEFTQLINQDSLEHFWGDTIVLDSDLQDDTEILGNDDGDECGDVESDLKERPASALNDAPGHETLDDAHAADPNRDVPEALEFDTLKCDSDASTECENTAFAETAVESPSSHPERRNEGVTPDAGYHERSKQGLHDIDSCKIKGDNLFLNNMGKMDLRVQGNDKNSLHMSASGSRAKSIGSVRAASLRASGLRAARQKAIGGLFGKSGTKNDTIATTKQNSRAVNDELGTLKFTGTEYVHDTSEAGDISLIQTPEKTAIFSDNRYVCSPCLSTSRPKPKANGAGELKPLSRQLNSQKARKLIFDYEDKSPTFSNNKPENGEGGLESFSFEKDPVLNRIILQQQNSLPSGVHAVSTNLTYVESQEPGEESQMNAFNVVDKLICLDSTCTPQKPTRNVEPEPSVIEGHQGTSGLKSLAQAAQMKVIHDKTNVYDWIDSQPDEGSPFAVKEIHDKNIYHGNQLEAFKLTTLKKKKLKSGKKGQHGMDRKNLEGNLQRKSATSDGAVLCSSTVMPTIVPNSEHHSPDEDGKIHAFTERLKNRSRIQLPSCGKDSDTYSKKPGSRKRVVDKSMHLPAETVQKCQRTDAAMAIDRKERTLPDNVDGHKESEFGSIIQTDKDILLTKNAKPVSKTSRKRNLSIGEARKDLKRSKMEQLKSASKLKGTVNGKSMEENKVNLRKGPQTRSKAQNAKGKLRNSIASGGKSSPKVTRKRRTVQKSHQQTDVTSTTEQHSRFDANNEVDEGGERKVKNSSITANKFADSIFQNDGQEDNAIGNSGMQMCKEEKPPIIHKGKKKSSFKCSDELLKHQKLQTKSSLSNTRSLLSDALKNTHIDVPIPKGLRNGRQKGSKNGTKTGVHFEFKEEVKGKKINSLNKKILAAHASEVKLTTRGDEVNSLSQSVSNSDNSMSLEERRQKRNPSSVQVLFSHSLPEEITKHQKKILAKLGGNLATSVTSCTHFISNSFVRTRNMLISMAAGKVIATTHWLECCSQAHYFVDEKNYILQDNKKEKELGFSMHSSLAAARRKPLLKGIYIVVTPNATPDHEALKEIIESAGGQIVQRDSDFLKSDACKEERLVIGSDKDYEYSLEFLEKGIKFLVALYEEAPEVDGNPKYLISVDCDL

>Mvestita_BCP4

MGTKKGRQHRGNGHFDQGTDTQILPEQTQLQTQLLSNQFTQLVNDDVFLGDTLPNDSDLEASGGTEIIHGSDEDADDGDVQSELGLGGPKLKENGYVDAGGGPDTINVNAAGYNDDTLPDSDATTEECEEADIDCPGAVVESGHDSGMPLDEKVQGCSGNEFIRKENTGDVRHEDLILMDTQKVESDDENSEKFSLSPSRINRSTPRSSLKKVTPESKLKTIASIRAASLRASGLRAAYRNDSTGEDGTTCDSDSIQSPQVCQKLVADAEVGSCGAENKHYMGQSDDISLVQTPTKNLGSINGCSPRMSTSKLKLKNISDLHETKQAKNGQKGRKLLFDSKEDSPSHLDKKMELYTCNKQTTCSATNGKESVVVNPGAGEDQISFPFAQRPACTILSYLESQEPGEESQMNALNVVDNLVLLNTTGMSQEMEATETTETTGELHDPFGEGQQGATGLKSLAKVAQMKPVHDKLDVYDWVDSQLDEGTCSDSENAVTDNKKQTKVSSSYKCKKKKSPPRQKNEKFTPIPKNKDNECTQKHAFAAEAINPDGFGLVQAAVQNGSNANLTTNKNCEHSDISGKNNCSAATSFDMDMTINLYMLKKNLKKKKKNSGTEVSSRKNSNVYKEAERSDEGKGKLPDIEKKNNTKDDMDTVKQLREEGAIKPKEINRQSELLTETRDTSKNKAVNLNNQLNDQEPEQCPQIEDGDFDDETVKCGPKTRSKSKVESNNSTMKKSTRKTNTRYASAKSTAKKLPLLEVQSRIDGDSIEGSGEMPASTQVSPKVSKRRRGLFPEEFPESLKHQKSEAAMNHEGCPSTNKMDGSVTRRTRNSIHLTKNNVLEPKTLSEANDIAGSLQQSEKQTKSNIKKGRKSVGIETANTHVFQSPLEENKRRRREPSSIRVLFSHSLGEDTTKQQKKILARLGGNVAKSASDCTHFICKNFVRTRNMLVSMAAGKVVATPLWLESCSQACYFVDEKNYILQDEKKEKELGFSMQSSLASARRKPLLEGMKVILSPNTVPDFETMNEIIESAAGKIIEGDEILSSLQSKDSTSIVIASDKDFQFCQQFLEKGLKVYAAELILHGIVAQNLDFTRNQLFEDSGQRRCTRRTGRSIS

>Acapillus_BCP4

MANDRTEGSLYGPISTRNQATQTLPVQTQPPTQLLSHEVHQFTQLVHHDLTPHVGGDTLVNDSDAEGSGDTVVLNDDGELHGVGDSVPNEKELMSKPVENGHNSTLHVPRDLPDSDATTEEGDGAYTGNLDADAESRPPQIDTPPDNEGAKTGDNVPAFIRANFPEVEGGSENVIRQETPHVAVVAATNGSKDSQSRPRSISSVRAASLRASGLRAAGRTASFNSSGKDGIKFDSESTIDSTLGTLDDGPKKPLNGVETELHGVDKTTAVEFDSRQSSPKVTVPFDDALARMSTSRSKSKGASSTHASLEHAFGFNDEAPGMKDRIDSDAMLMTEKSKSDAEKSSAPLEPVSLGLKRLRSQASVINGSDAGVDADVKLQSGPHNSQKARKLVFDFMETSTDKFGGAENTAFKGSNTDYGSSLRKQTSGIHAGRQGSVPSGVCAASTNLSYLESQDPGEESQMNALNVVDKLVWLDASCMPDDDATNHVPQDGSVKCHSGIKGLQSLAQAAQMKAVHDKLDVFDWEDSQLDEEASSDKKPVPVNVRGRKRQMEAPKVTMSSKKQAVFHQQKTQPVPPCPDKKGQKSAHVVSTPEMPSLGSGRLQFISGRLQDMEMTPHTVLTTRLRNRTKGESRLSSKGRQSPGLQRGRRKRDDVKKQTTEVSKTHETEQQRTENTIETRVQQPAAEPRQRAGRNKSIDGSGRNLKPEKKQERVNQEGKQEAVTKTSEIDANPPSGLGLADRTSQDDAAADIWIEAFSKAKNKTPMDHNVELIKFKKRSSLHDETGEDLKKSRTMEASPVTRDDKDLDTCEQEEVFTGKDRVQSGSKSESNLNKYNMKNLASSDQRISRRANTRLNKVAQQNNVKGIKKVDTDSLEKEALGKLKKSKISDTRQLKGSSHVEHQDASEKEWNVLEKDESVRESKKEQDTKHNSTADPIKKSRKVTSQSAGVEHVSRAQQDKTGENSVLDRGPKTRSHAQVTRLYSNSTDRTATKKLRTSEEASQADFGAEKPSRKADKSRKKEGLMPHQHSGNSVEEAPARVSTRSTRGREAADNSVLKSANAREEVPAQGLKRSTRRRDATDASVPESAKAREDAVSQPGEQDTNKFLQHIGELRSQEGDVSGALTRTSGSTTESVKRQEPVNVVKESQVKGNLTSEGQLPKNQVVKSTTKTSKPLSWQNKKAKALRTLEKLISSSVVDEGRRRKRESGEIHVLFSHSLTEETIKHQKKILAKLGGRTASSALECTHFISNGFVRSQNMLISMAAGKIVATTLWLESCGQAHYFVDEKNYVLHDEKKEKEFGFSMITSLAAARRKPLFKDVEVVICPNTIPEPTALKAIVESAGGQVVDARTCLSQSDREDDKPLVVVANEKGLETCSQFLVKGLEVYSEELVLQGVVTQALDFSRNRLFEDYRQQRGSRRT

**GYMNOSPERM**

>Tplicata_BCP4

MESGSQNKHSSSYYDCDTEIIESSIGGENMEENEIEDSLPYVDTVPLESTVPLEDTVVLE

NVALLETQLVEDDKSLEERLLPLRKRDRDAGSENAVNVVNIQGDTEATTQLLEDFQLDNG

ISSLRNHKHMYTVVDSDDESGGETVLLSEDDDASDGRSSPQRADELKLNQFPVEAVPFPT

TRHVKETSLAHSRDMGKGFSFNTVNSHHELEQKALVDSDALTEDECDLGIFPQKSSQDNH

KLDDSRALLQECEPDRKNDSHILTSPLLSDDSNKIEASQSVCSGRQVKMGSSPADIARGN

SHDLSSASVPKSKCRNIASIRVASLRASGLRASEMLSKTKELTRVQGAKKESDGNYDSDS

LSILGMSKPVQVTKSSTGFPSDAKFRTESLRARRLFPDTESIEINKMKARDEFELHNRKA

RIDNKDEFELHDNMRARHDNKEDNAPRVIHSNNDEQMTMYANKEDDKPGAEIVIKEDDGP

SETFENRKDDVLRKGASDAVDPQQLKSRGLQELGRGRKPPLYSISAGLSYADSQEPGEQS

QADALNMVDKLVWLNTTGFSQDQAPPKLLAMSCSGPPSAKGPQNLAQLVESRGPTENIGV

FDWVDSQNNEAGRQFFGKTTTQGFLASTKKGVKLKSESKRRGCRYDKSKNDSSVQDAKAE

NIGKKKSKILLQGLQKVASSGVSEANLSPASNSAHRIQNKFDVVKDQEANDTVSNGANKS

QFASDTFRFSKRLQSGKEQSHDKNKTHAADEKEFAVEKLPLNSGEACDQQVRKSLTCRDS

LDLSNIGNDTQLAAEAMQIMCLETPCNQATEDTLMVAKRGCAEWGDSRKRNNRVSGRGAH

EASEDHKSRKRTLSELRGSGENHANQRRTTRNYVATLEEKRRVPWAKDKGKLQKKDEGRK

SERLRKSKQDGKKKLESKTNPAYSEAWKENNTLVENSLKVPKRKRSNMEEALKTAEEIQL

VGADARVGCFTRQKKRIVEKQKPETQVLRTTEEDKDVLNNSAKVGRSKRKHADVEEISYE

QIISPGRQLSKSQPTKDEKTSANTGEMCLRAGEVAVTSSSLTKSLLQEKNLKLDKQSFSK

SSDAHDENKDTSVDLTKSVRRKRSKTNKEESKMCKSISTGEGLHAGTADRGLQQKVGEGT

AMRTTRQSVRNLGLEIGSSISPFTAIDGDKSDNNNMVPESSAQGQKSRGRKRAHTTPSDL

KLANHGTLAYPNKDEVRLIDTSVDVRSPFPCQGDNIKFKSPDTTGFGQKATQSSRKESLT

KGTPKSILKRELGQLVTPESGQSPILKDSARKRREPGSICVLFSHSLDDDIEKQQKKILT

KLGGQLASSAADSTHFVTDKFVRTRNMLEAMAAGKSVVTHLWLENCGQASYFIDEKKYIL

RDEKKEKEIGFSMTASLAVAQKNPLLQGKQVFVTPNTKPEKESIISMVKAANGQVLNGTQ

GALSKEETMESIIVISCEEDYKVCIPLVEKGARIYSPEFLLNGIVVQRLDFSRDRLFTDY

EKRPRSTKVHSRQRT

**ALGAE**

>Cbraunii_BCP4

MEDRAGEDVEQGAKGCADEKAWPRQNYGSKEWTGEMFNGGNHDAFAGSREDDEQMLGEVSGGDTQILSEETTYDEEDGNDDNSEVNSMAHGATQIVDGGYGVPCTLSIDWSADPEPYPEVLLTKTGGGGGGGGGGGGGEGSGSPRRMIERGATGAAYDTTPGRYNAIRSNSDTIGCAPDYAGLFSRSRLRLELDDDSEDPANNGDGDDDDILCPETQAAEETEYNHYNGDNAGATGRRGG

EEEQESNGALVPRSPTEECNFLNHRYEIGTDPSNGTCEEEEGDVLVLDTQVIGSMGCELLHDDPFDDGKEIHRSGPFFEGDGVGCRSRDLDDEEDDQELSRRRLADAQPDFSTVFETEEPVCASGDSQGGGGPCGXXXXXXXXXXXXXXXXXXXXXXXXXXXXXXXXXXXXXXXXXXDFSTVFETEEPVCASGDSQGGGGPCGEHADRDEEQGKMNSKEVEGSHPPSASLPGQEKEKEGG

GHSEGDPVKAMIRGEKKKKKKKTKKKKKRKRTRKKSSADDSNAMAGISNVPRKAAFCDETSDSCDEDDNDRKLNGVKSCASEIKKRRTNRDAAGDRRGRLTAGVHRSADPDSSSSSGSTTCDETTTGSDEEDESSDDDDDDDENDERGQEASISTKRREEEDAVHSSSREQPGIPPLPMDQELTACDSDATTCDAVTSDEGEGEGEGGAEREDDGEMKSGVNKADGAIGRLETENATAAH

EPGADDIFFVKIAGKKAGEEAEEERGSKETEKGLLRRWRGDRRWSEGSQSPDRVPASDEQAEGEGREEELQEMTIMDSRSRQADVMKRMEGSEKTTRDSGLKRNARIVVGATTGSRRFPLMGGADEAGHTGVHEKEGKEENEGSACEAAASESRMTGRVNADLADLADHGKNKRKAVSTSCLKGGGGGGGGGGGGKTLPWHDERNGAEAADTQLWVHGCSPVSGYNAMIDPAEGTQTWIY

GCSGPQEEEEEEKKKEEDGRDTVLETQAWLHGYSSVPEDEGKEKADGMTEAPAGMGNNSYHQEVAVMGRNSKMKDSVGITQEWIHGLSSAQEAEEKKTGTMEETQGWKHGCSVQLIEVSEEAEVMEEGMKDTIVGETQPWAHGLSLAVEEEYEQDGERGEKAGTVAETQAWVHGCSVAVEDAETQEEEEEEGEKRDAIGETQPWVQGFSVPSGEEPGEQEGKAGIMGETQAWVHGSSLRQ

EEEEEEEGEKRDAVGETQPWVQTFSVALEEEAEEQEEKTVAVEETQAWVHGSSVSQEEGEDGEREEECERTDTVAEKQAWANGRFGPCKRNNPVQEDRRHVRGWIEEEKKKRVGPNDDREPGLVKRNGSMSAEERRMEGSSYVGETQAWVGDDGAKDGEEGMKTEEDDTVDRALPDHVYVGETQAWVGDDAVEEEEEMQMKDDIVGSALPDDVYVGETQAWTDVDGAEEEKEEKEEEEEK

EMNMDEGGVRYEERRQPPLRMARTGSEDKQEVTGDGEAEGEGRREGKEEEVGGLLLVKDGRQKNGGMACVEETQPWKHYESDLEENEEEGEEDKDYLRGVEKGRELGGYNTVYQGETQPWIGDLECDGEEQAPQEEPTQARRPERGPADDAGGGKATWTKRAAEQDMARMENRNLKARDPRRNGGGRVTVLSLVDLQCTREGDRERTLGAEGEEKEGGRKKREESAQRDQLHQVGVDLGR

REEADRVGKKHFSLDEADTLEWVPDSSSLATEFDFIPADRYRPLQWISNPAIVVGGNRKREGENGAGREQHEKNAGVLGYGRGGGDLAGRSDENAVVTPERKDAFRSFRVPKERAIKPVEPIKVPKERARKGVEPRHCGGDMRRQGDRPADDRDVLASNEQDDLQEDLQEERGSDHPGSGALWTMKKKKQVWSAKAPTVQWEQDANAGTCANGGSIPRKDTRGRGKSPPRGRRVQENTCC

LMGRGSLSPGSSSRLHTRHDAAGYPNQGTKGADGCKSPRLVLGNLGLNLLGDPKKAGGGRGGGGGGGEVGKGKGEREEDKGWEGRNGPSIAGIGAWTDVNTIRPGEGGGGGGGGGGGGACEGEKRGVAGLLGGLLCSQVPFDDPMSHVVGSAFQESQDPGEESQAQALDMVSQLVSINAAAVLSQECPDHVESVIDLDRPFGLEWFSSDMMHSAALTKMSTASKHPPPHYLDNQKSKKAH

LAVFDYVGSQPEKEEENMEGEAGHECENDKNVRRISGAQTTGGKGRGRGRGRGRGKKKLGEGGXIPVVLSDVFDELASGQSKNEAGKRRGKGGRGEQAAEKGGKRSMEVQNHFSPSRRGRGIGQEKDGVLIGQKKKDGVLRSVEAAESSLEDGQPMTAELVSKARVARRSNECQEGSRIPSTAANTIGRTCRVDGFAPKKPLTEVHSESLLLPGKEADDGMSSGAWGTLNITDDRRTERA

DAIVVVDKHNPELLSGFGKAHSDTSSADMVVAGWQVNHGSRDLLGNKSAAVCSKAGHVLALGLPQSLAVTEVGKGAMAEGGRSRSTTALLAYHQSDAEFTLTTGKIPRDDRCGGACSGDRAHYKSSSTDFKSVPEPEHEREESQRQSPSRQFIDSVPLVGWTQDAAEIIMLMGAASAGRSAAMAERTADDLTEKMTQQNAAALLDVGGADAAPPQLANHEHPASVMLFNKRKRSAGGRGC

RTPGFNDGGVRASVTKRLRSEDGGGTQKTRDSAMAVSSLADDRGCHIYSAGEGGKPRSTACSTGPRGEGDGHRLHCNISSAELGGDDGSGTVAGRSAGLRKDAKKFGEVVITPGKKEDGVKRAETPMNLACLGAIADAKWEGEMLSTAVKTSRKRNAEGDYGGKQKRTRKSSFSSSSSSYFKSAMGDGSHGTAIQPADDLQNNNCGDDTENALRSRGGPDNPGGTQKLPSCLVGSGNDET

VKQETRDEKEKNGDDGSTEDPQGSGGLLENGKQGRCKEDREAVVGEDSRLLLRQKQQKKRQGRRAEQESKLQAKRAEQEERLFGESAEEEKCQQEEREEQQERQPKEEVRRGRRQSQKAEERKGQEGEKTKQQTRWNGEKMENYNEEAMGRALQRTVEGGAEGDAPKTFRKRTTWVVEDDSTSSEELHGERLGVKEHFDRAAGMKTRLPVQRNGVTKEGIVQLRKVKPDVLVRDMVNVGK

GWKKRKRVVEDDNSATTIRSRQGKRKRRVVEDDGTSSESSGEKGDRVGGNGSRTAADLSNRQFKVVVEDDSATEGSEETITTSRDGDNIAQKGRLESDMEQHERQGGIGRGRGRGRGRGRGRGRGRGCRGSVVVTNGRLNRKGSEDCDRSEESMEKRVEKMVLKPNGRKVVEEEATCDIDGNMDKTPRGGQRIRANSTSCTHMASPSASDLPGWAHIKASDNSDPGTRTGSKGLSPLLPS

TGPREDPMVAEGGCQDQESGGKEGLTRTPRGGWRRKRESVDPKVKVLFSQSISREVTKQQIKVIQRLGGMVVNKASDCTHFVATSFARTGNMLEAIAGGKHIVNTAWLEQCARANQFVEEGPWILEDHKKEKEMGFSLRASLALAARKPLMEGMKFFITKNTNPGPETMAAILRSAGAEVVLHSSTGWLSRCSSNRRLLVISCEDDHADCKPFLNSGCHIYSAEFVMSGIVHQRLDLEQH

RLFCSTSVIDGVTRFEERKSPASVLRASSRASVKRPR

>Knitens_BCP4

MHLVKSPSEVTSTMEDQTQATQLVGEPTQVLGEQTQLLPGATLLQSTQLVGEPTQLVTGPEWQGDEETQPVDEEIVFSTAEGGDEETQAVEEEGGDGEGQEELVKVSGLKMEGLAHFTEDREGSPSVKEETAKFSASKAEAFKATSQDEEHQQSPADSTPKSQPVPENIHKAPVAEVIGGWRTTRKSTEGETSDKAPSISKPPASERRKSLSQPVDSDAPTEDESDGEPEVEGRNEGVERRGEEEGEGRGGKTEGAGREGEEQGVERGGEEADAGRGRVEDGAGNEGNAETEEREGLQEAAELGMRQEDAHGGSQQSAEGKGIQEAAASEQQEPEVSREQSPPFRLSAAEEPVAETRGVNAPRSQGVNMPPASSWRDSIIPDSDTEGPDGREGEFGTAPSVNAASQWWVLDSSQGPIGTA

RSVATNATRETDRGIGFGSNLSLGEDVPAATLVGETGDEGVRRAPDGAGPDGAGPSGGVVGLEGEPLQAGGAGPIAVQSGGEKSRKVRGGALVESQKSASESASKRGRSRATRAQKANRSEAGEGAAETEDATRHGGSDVRGEAPPQTGQLVATDGQMAEAVRASPEREEMEDRTGHELAPPLEPVLEDAVPVSVPQQQVSLVDEGIPELRPDQREPPFTQVDEDDAPVAIYTRRTSAITPARSAPLNFSAEIGEETQMEALGVVEGLLGLNDVDMSEEQDKGLEKKHSLGPEPKPSQGVAKSSSIAVLPPPQAVPRTLSIFDYDNSQVDAEPDEPVIHPKKAPAKATPAKRTRGGKTGTSRSMGPPKSAPNKASEEAGGGTGGWISRKGVGDEKKKETPATKESREKVKAGEKGTEVGR

AKALSTGKIAQASGKGAVTIQAVPAVIQSMEGGRQKATAENKEAAQKNGEPVGGGLAQMNWTQAFAADSEPPENLPPLPPVVEETEKRKGVKRGLFAASLMSDEERAAEEKRAKEEEEKAEKDKAEKEKAEAEKQVPAKGRGRGKGKGKEAEPVAKEGAFGVATRVSPRRAGRGAAAKDPKKGRVSEEEQTAGPDAMLQTEREEEERGVGTMNEGRAVESVNGNAKGAKEAVTEPVPVGSSHRYKHTTKRRRSNEVTASERAATQKRLDRGFEDTEDADEEEEEESDDELEEDEEEGQKDAGGAGEANMLFMDSQVPILPKWKKLKGAKKGALKSALTSIGESDVNDAPKSAHKATRKEQSDTKRARPTEPSATGVPKSAPHEDKGAAGKGQLGAKRSAEGAGTGEAKRKKVEGGAKEAT

ALLVAPMTREDPSAQAEGTGTGNVTEPAARGRRKRGKEAEKAADAVGGKAEERDAKRARGPGGRIVSSRFRETPSREAKSSEPEKPQTEAAKARDGGVQKAASVAPQEAPGARRRSSMVPIASLIRTRNRRGEVEQAEDEAGGLDLAFSQVPIASLREKKEVVEKRKPGAEKGPGKGKAGVGSGDKEVSSMVTAKGKGGSKRKAETLEEQSGLELQAVAERDEGGEGGVDAAEGDQDGEIVFVAAGTDAATEKAAEKDQGSGGRTTRQGAGKESAKEEVGQSRAVETRPARKGRKPKGEVEVAKTGRTDVSEGAEKELRAGSGNETGSGTVKDQGALEDVSGAEEEEEVVEIGAAAERKGKRSVGKRGRAAAKQGGAPQEDAKVEKVDDDVAGAVIGGEEQRPTKRTRRGQLVAEGAEKK

ASDEGGKAGPSERVSSRGAKKSTNEAEEQGTSKDEAPAEGNAERRKAEEAGGSARTLTRGARTSGDDGGGQGGRGRLSVRVSGRVSDDLEGLVSPTLRKKREMGELRVFVSSRMPDRVKAAQKKILKRLGAQEAQDVPSCTHLVAETFGRTQTMLEAIVGAKKIVKASWLESCGQANYFEDEEPHMLQDEKQEKKFNFSLKASLAAAKEKRIFEGLKVYITPNTTPDRNFFKGILEMGGGELITRFTGAGADSCLVVSSDKDRNLWEPLATKGTPIYTTEFLMSAVVQQQLDTSRNLLSGGGGAAHQTGAARVRGGGKTKPQRKGAAWR*

>Catmophyticus_BCP4

MVSSPSPAVSSEDETQSIDELSDDDEEDDSSERKEQLLFNNGQLQSFQLRHGPNILGRQSVKQARDTVRAESWRSAGPSSQNAILLESPKLSARHVCIDVDDHECFVMDLGSKNGSWLKSASPEAASGSRQRLRPYRSHSIYNGDILFLADVQLSLKLQRKSQESSMFESEQPSRLQLNMSALEKQDTESETREPHHSGDVPAAVAVPAGQSRTFARISSLREWQSRGVCAVAEETQIVDVGVQALPPAATAEQTQILVVNAREVPNTVPAATVTAEELASVPPQQMDNVPETRSGTQESIPNSNSDEPLLCSEQLVLTNSSQSPKKPSAPAILKRRGSRLAFAETAVAPSNASPDSADQADGATPPSSYYPSILNTDALGTANTTSYRSDAHAPDMPNGAHEEAEEHEHEYKQPAETAAGPVGKADTSSSASDATTMSDAGADDGVDDAKLAPAIDVHNNIAGAFITEMESLGAPLQAMQAANTAPAPLYATHTQAQLLDTQDTSQLAAAADQALNAVAGMVAAVTSSQAPDSAPVASLWRPKPEHKLPDSGSIPDPPAHGSPIGACTQDVANVLIQQLPSVAERRLSFGNALAHAAPTDLEEAPVGVNAPVVGVADVALAEPSSFVPDTEVNTATDPFALKVATKRATTVDVDGNDGAPTPRMIMGRKRKGKQQPAAKASRPSSIAVPEAAQDTPDEPQSTRRAAAAQQTPSPVVEDQPAVKRQRAHKLDAQQPVAAAADTPIRVLFSTSLKDNLQLVKQAERLGAEITQLVTDCTHFVAQEFKRTVHMLSAIAAGVWVVRPGWVEDASKYLLCDKRQEKQHCFSMEQSLQRARRGPVFAGLSFIITPGVSNPSAEDVAAIIKAAGGDVIDDPTEAENKSKVLVVACEADMVSCKQYAAADVPVHTSEFVLRSVVTQQLLDKHAHRLGVCPGPITGKRSGKRKGAQ*

>Nmirabilis_BCP4

LADAGRESSKGAEDDIKKIMRMVGLATFDGKDKQQRKGKLDVNSGGVSKRAKRTPEGLEEQKVARARKNNGQNLTANNVAGNKKKGMSGGPMAMEELDTRAEVDKEKGRRMKEAKEDLCATVEDVSCKRRRKRCRILDDDETTEESDGLQVAADGLCMSAGRGTSSSGMSGLEREEVEGIRKGRSEDKSPGMGVDAICKKRGKRKRVLEDDGDTTEGEDDGQRGTESPHVGVGVGKKARSTEKAELREKQRGEGRGRHGGDKRVACETQVRVIDEVSARESNAGRPAVAGAIASGNGSDRASSGVRLEDGSERKADVRGRRAGGNGCAVPVETGVKERQGGKMNTIAVAKGGEMVIDGKTPGRQVGRGKAASPCHAPSPSLARERHGPTFKGDQYGKRGGAGMHEGHMANADIAGDSHNDEDVNARDPEDKMGHEAELRRKWGRLKEVDGAVEPLSTGLARSHSSGGRLKQNEEGGFGREWLGNLKEKGPRKRNGGEDQTIESHGVNMMRDWHECATEQGRNSSRVLANSIADSGGLCKSEVAGSNPAVERATSNAGRDLEDLALQPGGVLVTGGTRGEVTQERISHTTPSRDSRRRRESIGPKIKALFSQSIPRDVLKQQTKVLERLGGVVASSAADCTHFVALSFARTGNMLEAIGAGKHVVTTAWLEQCARVAAFVSEASYILEDRKKEKEIGFSMRVSLASATQKPLLKGMKFFITKNAKPGPESIAAIVRSAGAEVAGNPPKRRLSKCSSVDADCGFLIISCEEDHEECKTLIKKGFPVYSPELIMSGIVHQRLD

>Smuscicola_BCP4

PERTRVFDWADSQAAADDDDEPAPRPAAVVGSATAAAADGRPVAAEHKRRARLGGRHRPASTGAASTPAAIKRKRPPAPAKDVASEPAADLRQPPVVAAAVRQRIADGAAGRRLASGSKGCTLAGTPATAAKGRPLPEAAPCERDDLKEDSDGGQLSENGDGGGNDRRLSMWGGACTQVAVEAIFSMRSGGGGNEAEPVMADEKRPPPSEAEYCQPATTASTLPGDGAAHCRPVSRQEPVTDRQVPAPEEASTVPSIPTARRATRNLLRKAWPERRVRGGNDGEALVDVEGSFSDGGSCGGRRRSLRSKQLPLAASGVTAASGGRAKRKRDGQEEGGDVTDAVCPKAGGRQAADPQAGSRGVPSVLFSHHLPAKTLARQRELLCKLGWKEVKKAASCTHFVADRFVRTANMLETMVLGRPVVEPAWLERCAALGEVVAEHSHVLQDHRKEAELSFSMVDSLASARKRLLLQGMKVFLAMPSAKERSVRTLLRSLIQAGGGQILRTSKDVGSEESCIVIASGESHGVFSLGLPPGAAVFAPELILSGIIHQRLDFTKHHLRQTDTQQWGSRKRRRQLRLAGVYSADDR

>Mendlicherianum_BCP4

MALDPVANLGELGSQLTCPICLGLMKKASNLTCNHNFCKSCITTSMKRASVCPVCKVPTTKRGPGMMHDEPLQHASPAPAAPKSSMKRSAVESAQIEPATNGKRGKGNEDGIRTPAIQDSRGTPVANDEGELVHAKEQLLGEVERPSQWSKPTDVSRRRAASLSQGTPSSQVQASNSRSEKRQEVLEEWSAGSLSPAPLGGSIAPKKRIQLMPPPGFGPPLPKDVYSLKSSLSLDIGKLSPPRACHVSPSKSMLEASACNRLFQKQAPATLAPKEGTDLPPVHVMQALEPSSVQSNLPLK

AGTWSLKACKESRAKLVSCAKVSASSEAVTDAACSVPVSSSMRASTLPMLQKKSGPLSDRQNGVQKVIRSVIPTFFWLREADNEEKSTQVPPTQGTQVEPTPTPAFSDLQDSAEPRSEQIAQVPVVADSEPEVTPVADAECSKESTFCLSSPGLPKLSPAPKADSDDSLDSPVFRVKRRPQKVAAPAPSFPTLRDGSLAVELSKKDLNGEIEGPKLTQKDKIMRGVLSQLELRQQHFDLDKVCGPMLEINLAKGRVFRGDEPAGRSRVNHVHTHEFCLDWVPEVYWKGEKVCNVKQGLAR

SHRLSCASCGLKGAALGCWISKCRKSFHFACAKALPTCRWSEENFVMLCPEHGPHGFPAEILNEEEEEAEEAEEEEEELVAQNDSSEDQMTNESSEITPGDRRWPPGPASKWVLCASGLNQTGREQLAELAALAGACVVKDWSSEVTHLVTGLDQRGRAKRTLKFLSALLQGQWVVTPQWLETCISSRGPAGELPYLVEQDVHSVRGGARRSRHALVSKKEAPLFVGIHAYFHGEFQAPSKQDLAAVLMAGGGAVLQRRPLLPTPPRVDASGHQAPPKRMMVFYQRPCSAQSEPQCKELA

RAREDEAESIALPSGATALPHTWLLDSAAGYALLPF

>Corbicularis_BCP4

MLGAGFGLDDRPSSSERDEQDASTKEGDAVEDGGTVWVSAFASPKAEISKEGPTVSKRGKNKISSKQSLNGRVRVQEPEEEEMVQVNKKAEGKRQNNLPLECGEDFGRGSAPQGKRKGEDSKKRLLQRHSGRVQDRSATAVVSLGEQGGGEGGKRTLLRARIPGAASKAAMPAERGVEMEVRVNMKLPGGGPRVADMAFGVEEHGKSVLEAEVEVEVGGRRLRTSRVPAGGTPACTAGNVPAADIVGDRVLGKRRAVGEVKETVTVGRGKRSPRERGNREIGDGGVVANKRMRKEEGEGQ

RHAGDLGKGGDEEDAETSWEGPTKDSSSVPAKRQRTASLRKVLGNAKARKNCLTLSAERPREPPAAVTPSVPPPERMPNPRMRGEHDAGQASVSELARGTRSVAFKIGRGAQAATIHQPEGGDTRAATSNQEKGAQATMTKPVGGAQDVASMKGKRGLRRGPTRRQSAPPPAVLQTPPPATARALAVTSEKTSRRSDFAGSVAESVIGASARKRKRVREVKVLLSHSVVSKELEKLVTKLGGSTVKQASECTHFVVADKFVRSANMLEAIASGKYVVTQTWLTNSRLAGRFVEEDLYLLR

DAAAEREHKFSMKKSLEEASRRPLLQGKRVFIAAGPSATPNYKSLAGWVTAAGGEVVESYPPVSGQKAENSLFVISTPEATDLWSPLAKRGAHVYTPELIMSGILKQRLEFDLHAIDTADEVSAPFATTAILK

**BCP3 SEQUENCES**

**MONOCOTS**

>Osativa_BCP3

MHMMTSASGCAKLDYLSSQEPGDESQINAIDIVDRLLVEDDIETYQQISIDQTTRAKSAS

TLGSDIAQCLAKRARCSSPLKKAGNFDWVDTPTVDDCRTSIISMENTVDRANNQVKHGGC

GSSTRAWPILECIDEDLGTNCLKKTEPFCGTDDLYQEYDIGPNTQMAAEAMEALFNASTV

SYDVKENERPEDSVVKNMTKGTKVDKTCAVHSPIQKRKVNFLRHRSGVATEYKQIKVDDT

VRENGESSVSHTNTSQTRKYTKQMAGKAKRNISSGITQRDIDHEVSEVITRSGTNDSNIP

LSLDTDALIHPKRRRTYIFTSGSSKIEFIEAIKPTALRAKTTEVKQLSTANTVSVSDQDT

TSGLRMSQHSSFADHEASAGSSYFNPLAETFTVGLEKQSIPEKKGHDSSLMPSVPLRELN

GAGPQARTRTSETPKRVLKSPGSRELANLFRNEVSPVLQSSRRRRKHMSTVRVLLSQSMG

NETLNDQTKILIHFGLSVATTISEATHFVAEKFARTRNMLEAIATGIPVVTPAWLECCRE

ARSFIDEKRYILRDIKKEKELGFSMPVSLSRACKKPLLEGRRVLITPNAKPSKELLKSLV

VTAHGKVLERNAMSKMKNRSLMGAFVISCEQDYKICVPFIKNGFEVFESELVLNGIVTQK

LEFESRYRLFSG

>Macuminata_BCP3

MLCSIASVVISLVIYADLCLSVTISLFLIFQGSTVRSFTAVRTEAMRTSGLAAARYFASR

KLGHVSRSFNNIEHGEKEDSAVDGQIGLEVTGVNKWTKSRMELTPFRIKVGKLDLDHEIA

SFERSTQNYCKEGIKSRCCNMRVKRLFNELLPSEKNDSIRKDDSIFSKVDTPYLLSTDDV

VAGLSYVNSQEPSIVSQANALESVDKFLLINDVGLSQETKDVETDILKSSLVSGTKRVQL

FSEKTNCRSPVGKSQIFDWNDNLEDDGGGTVHSDSRLMISSPVRSKRICISETNIRKNLF

EDTKAQLDVTEIKRGSDGICDVGPDTQMAAEAMEALVHGFLINAEKEDTGHLDAENLTLN

SNSARIPTMNTASSKNVPQENRTSVNDPEVIVTRSKRRKMLSTKSCENRNVPRVSSTSSR

MKNKSVEAEKGCANVGLNLTTEVKRHPATHTKLLNCASPGEEFQTLSLTKDSSRYPKRRR

TGQVNSGNMNGDLNKASPVSGIDKDTEESIRTILLQSVIPTDADILSVDLQNHSLHAEKA

EKHMSSSEYVENAEENDKSFGSSEAKGLPNNLACTTPSKEMNAVSPVFTSQYPPRSCNKV

VSTSLVARELLRLDATGASTPMMKDMRRRKYMAGVRVLFSHHLAENTIKPQKKILARLGL

PTASSISDATHFVTDRFVRTQNMLEAIAVGKLVVTPMWLESCGQAGCFMDEKNYILRDLK

KEREIGFSMPVSLARACHCPLLQGKKVFITPNVKPNGDLVSSLVKASRGQVIHLFYATYP

KF

>Zmays_BCP3

MHQQMRRVTQVQVSHLQMKLPSVHVASVRTCGISEPRNSLSVNCMKQWKQKAWVNTIHPL

PKIVDESTSCSTSVGWVDNDSHGYVQNHDQDGAKSRDKCSTAKKLFADTTVEDGENNSRC

IAGLSYVGSQEPGDLSQANAFDVVDRLISINGGLSSQETTPNKLEIAKPCVSSKRGTLML

AEKVDLGRSSNGKAEIFKFVDSLEDEGGGDFFSKNKDILLLKPIGRGKPRSHFTGAKKSS

PKLSRGENKTGEALNKRNSKLPGRLETIPLTYSRLFKSDVESKRPSENRTKKNLLKVLDD

LSNDKSLEEQEKADVVLNDVGPDTQMAVEAMDALVQCSPAKSLSSEHQSVLNRDMIAEKS

RIAKSHSKNGSPQRASNIQEGVTTRSKKGKVTDFSAKPQKERLRGSKMQQSSESIVKVKH

KQTKSVPEKSKVSKKFISENKYHETPVAHRTRHCGRNDPSAFIELSNKHLRRGKKMTGDN

STIGQVQNNHIATESGLSYFEKESTEQTCTNNDQDLQQPRYGSTQRTSVNNVRNLEARRV

ESTTVVTCRDSPSHPKRQRTPTKMIQSTAPAAASHEIPSELARPCKKRRIFIRSVSDLLK

YAKREPSHGKSASMLSSIIEKSLAAYPVLNSSIRDDRKTSDVISSAQRLKESSHIENTSK

SPKTNPQVLDSAMKTPSKVVNELSPTFSPVNPSKGSSRSLLKASVARELLKLDPENVLSN

QQKKDSRRGKDKANASILFSQHLDDDVIKRQKKILARLGVCEAFSMADATHFVADRFCRT

KNMLQAITMGKPVVTSMWLENCAQAGCFIDERKYIMRDEKKEKEIGFSMLISLASACKHP

LLLGKRVFVTSNVKPSQVVMTSLVKSSSGQPLERVGRSIMEGKGVPPDLLVISCEEDYEA

CAPLLEKGASVFSVELLLNGIVIQKLEYERHRLFMDRVKQTRSSRWLKDTVQGRFVPVPK

RPRA

>Bdistachyon_BCP3

MSIMTAARERARLEYLNSQEPGDASQANAIDIVDRLLVDDDIETSQAITIDQISGTKSAS

TLGSKVAQCLANRTDRSCPVEKEGVFDWYDTPNHDDGIISNISRENPRVHVKNGISYLDY

QRCGGYGSGTRAGAMLECIDEDSGSIYLKKPETAVAANDLYEAYDVGPNTQMAAEAMEAL

CNATVSYDVRESDHLESSLGKEIEVDMVCSVNSPIQNQKLTCLPQSSGGMITQFNQLKVD

GIERPNGGSSIPLANCPSKSKTMKNTNQMTRKAKRSMDSGVLGVTNDEVSEVIMGSDPDD

PNTRLLGKDAVIHPKRKRTCMLTSGSSKVEFYKATRSTTVRAKTTGVTELPAAKTVCIFD

PGTIKGTNMSRKSSLAIHEASVSDANSADASVGITTRSKVRGIQKTSHSSQPEGTFSTRL

VKQSTSEQKDNDSSFKSRVPLRKLNSTEPQSRTHTTKKPMERGLTKSAGSRELASLFSNE

VSPVLQSSRRRKRHMSTVRVLFSQSMDSKTIKEQTKILIHFGLPVATTISEATHFVAEKF

ARTRNMLEAIAMGIPVVTPSWLECCGEARCFIDEKKYIMRDAKKEKELGFSMPASLSQAC

KKPLLEGIRVLITPNAKPTKELLKSLVLAARGKPLERFTAFMKNKNFGGAFVISCEQDHN

ICLPFIKNGLGIFDSEVLLNGIVAQKLEFERYRLFRDKM

>Msinensis_BCP3

MSTARVQGRLDYLNSQEPGDESQATAIDIVERLLVEDDIGTSQKMSAEPMSGTKSASILG

TKVAQCLAKRGDCRSPLQKTDIFDWADASSNDECTAIMISRKKQRIHANTQAKNLTSQRY

GGSGSTTIIGSILECNGGDSGLSSFKKPEPLGSTDDLYDAYDIGPCTQMAAEAMEALSNA

STVNHGVRENAHPESSVLRTNLGKESKANKICSEPPIQKRIGGSSRSVKKNPSKSKDKKG

PKQMAGKAKGSMGSITIEGDTNREVSEGIKGSGPSDSNIVGSDAVIHPKRKRTYTFISRS

SKVQFNKAGSSTTLSSKSTGVAHSSTTNQVSLSGPDLNQLARLLEKQSTSAQEDHNPSLI

NRVPLRELNSKRSQFRTQISKKPLKRGLVKSSGSRELASLLRNEASPVLQTSRQRRNMSK

VHVLLSQSMDKETIKMQTKILIYFGLPVATTISEATHFVAEKFARTRNMLEAIAMGIPVV

TPSWLECCSEARCFIDEKKYIMRDMKKEKELGFSMHVSLSRACKKPLLQGRRVLITPNAK

PSKELLKSLVVAAHGQPLGRITASSMKNKNFEGAFVISCEQDHSVCMPLIESGLEVFDSE

LLLNGIVTQRLEFARYRLFP

>Tintermedium_BCP3

MTAARERARLDYLNSQEPGDASQASAFDIVDRLLVEDDTEASQTITFEQISATKSPPTLG

SKVALCLAKRVDHSYPLEKAAVFDWLDTPNLDDCISGNISRENPRVHVKNQTSYLGSQRS

GGYVSGTRAGAMLECIDEGLGMNYLKKPEPVGASDDSYAAYDIGPNTQMAAEAMEALFNG

PTVSSDVREHEHLENTLGKENKVDRIRSVNSLVQEQKLACLPQSSGGMTPQLKQLKVDHI

ERSNGDSSIPLMNCPSKSKTRRNAKKMTGKAKRSMESGVSRGAINDEVSDVIMRSGAEGS

NSPCLLGKDAVIHPKRKRTYTFTSGSSKVGFKKATRSNADRAETTEVTGLKTAKSASIFD

PDTIKCIKMTHKSSSANLKASATTTRSKVKGVQKETSDTNQLEGTYSAELKKQSTSEQKG

SDSCLTNRVPLRELSSPEPQSKTHTSKKPLKRGLLKSPASRELASLFRNEASPVLQSSRR

RKRNMSTVRVLFSQSMDTETIKDQTKILMHFGLPVATTISEATHFVAEKFARTRNMLEAM

AMGIPVVTPSWLESCGEARCFIDEKKYIMRDTKKEKELRFSMPVSLSQACKKPLLEGRRV

LITRNAKPSKELLKCLVVAAGGKLLERITVSMMKNKNLEGAFVISCEQDCNICLPFIKNG

LGVFDSELLLNGIVVQKLELERYRLFRDKM

>Pvirgatum_BCP3

MSSARVHGRLDYLNSQEPGDESQATAIDTVERLLVEDDIEISQKTSTDLISGIKSASILG

TKVAQCLSKRAEYNSPLQKSGIFDWADNPKNDECTAIMISRKKQRIHANTQVKHLGSQSY

GGNGSSTRAGFISDCIGGDLGVDSFKGPEPVGSTDDLYEAYDIGPCTQMAAEAMEALSNA

STVNYVVRENALPESSGKESEADRICSVESPIQKRIGGISNSLKKHPSKSKNRKNPKQMA

GKAKGSMDSGSIQGAINHEVSEGTKGSGASDSIILGSDAIIHPRRKRTYMFISRNSKVLF

NKAGSSTTFTTNSTEVADSSIAKTVSASGPDFNKHARVEKRSTSVQDHYSSLTSRVPLRE

LNSTDTQSRTQMSKQSLKRGLLKSPGSRELASLFRNEASPVLQSSRQRRNMSKVRVLLSQ

SMDKETIKMQTKILIYFGLPLATTITEATHFVAEKFARTRNMLEAMAMGIPIVTPSWLEC

CGAARCFIDEKKYIMRDMKKEKELGFSMSVSIGRACKKPLLEGRRVLITPNAKPSKELLK

SLVVAAHGQPSERITASTMKTKNFEGAFVISCEQDHSVCMPLIKSGLQMFDSELLLNGIV

TQSLEFDRYRLFHEKTV

>Zmarina_BCP3

MSGGKEVSSTEDYDYFQEPDQLSQYNALKVVDALISLNDLELSPDNFLRNENLDEIKSPP

LSGLKGAQCLTKTFSRSPIGKCDVFDSSYEDESGGDFFARKKECIFSDNKRSIKPSRALY

KSEIKKDPFENGKDKTKFKALCNINISSLPDLIVRNPNNLLNSNSDKEIKNQKESEPAKN

DNCSNQSFATKKGSLRCASQQKIVSSIGGTGSSNKLTPSAAKLNNGTLMSSYARTGDVSI

AMVELVDEISKCGDNSGVKNSLNECESSSNSREKVVNAVEPLDKDKECIQSIARHTRQSR

AEKSSNKTDVLLINDTCKMKYQRSGRSTQDRESSFEKNLNLNKHHDRQVDECSNLAKCRT

EFSVGIVTLCQLKCKRICLREKMINGEKPLDKECMYAGIRSTPVARRTRQSRLEESSKKT

NALLKKDTDEGKFQENGDFIQDKESSYEKITVSNKRHNTQLNKGLNLAKCRTRSGLRLVT

SCQPKRRRTRLNYAEDVDYVANPEFESELQRSKASFSVIDSQTEATMKNTLSIHGNPELE

TLHREEIQPAKMLFSSPLNKIHAVSPVHIDVNSMQVSNNKMNKSLAMSAIGNDLHRLKSS

VAISTSALKRIRRKEDLIVIRVFFSHHLSYDVIEDQEKILSRFEIPVATSISEATHFIAD

KFSRTRNMLEAMAVGKPVVTHLWLESYEQSGFYIDEDRYILRDLKKEKEIGFIMPDSLAC

ARKRRLLQDKKVFITQNVKPDKKTITNLVMASGGQLIERFLRSTPNDKNDLLVISCEEDC

SICIPLLEKGLEVFTSELVLNGIVIQKLEYERHRLTTRLKNLIGNESLPETQFK

>Sitalica_BCP3

MSSARVPGRLDYLNSQEPGDESQATAIDTVERLLVEDDIETSQKTSTDPISGIKPASILG

TKVAQCLAQRAEYNSPLQKSGIFDWADNPNNDECTAIMISRKKQRIHANTQVKHLASQRY

CGNGSSTRAGFISDCIGGVSGVDSFKKPEPVGSTDDLYEAYDIGPCTQMAAEAMEALSNA

STVNYVVRDEAHPESSILGTNLGKESKADKICSVESQIQKRIVGSSSSLKKHPRKSKNMK

NPKQMAGKAKGNVDCGSIQGAINHEVSERTKGSGASDSNILGSHAVIHPRGKRTYMFISR

SSKVQFNKVGSSTTFTSKSTEVADSSTAKPVSTCGLDFNKHARVEKQSTSAQEGHNSSLT

SRVPLREINSTGPQSRTQMSKKPPKRGLLKSPNSRELASLFRNEASPVLQSSRRRRNMSK

VRVLFSQSMDKETIKMQTKILIYFGVPAATTIREATHFVAEKFARTRNMLEAIAMGIPIV

TPSWLQCCGEARCFIDEKKYIMRDMKKEKELGFSMPVSLGRACEKPLLEGRRVLVTPNAK

PSKELLKSLVVAARGQPSERITASTMKNKNFEGAFVISCEQDHSVCMPLIKSGLQVFDSE

LLLNGIVTQKLEFDRFRLFH

>Othomaeum_BCP3

MGTTSTIKVPGRLDYLNSQEPSDAAQATAIGVVEKLLVEDDVDSFKKVSTGQSSRTKSAS

ILGTKVAQCLAKRSEYSSPFQKLDFYWVNAPNTDECTASVISRKKQKIHAKTQVIHTQNY

GGCGSGTRTEFVPECIDDDLGVNSFKMPEPVGSSDDVYEAYDIGPCTQMAAEAMEALSNA

STVNCCVRDNEHLDSSDLRRSLGKERRADKISSFLSPVENRNGGSSKFISGNAKRSMDNG

MHGTINHEVSEGILGSGADNSNISSSNAWIHPKKKRTYMFISGRSKIQFNTSRSTTIPTK

GTEVAESSTAKTVGMSDPDFSLLTGLKEQPKSLQKHHNSSLTRRVPLRDLKGTPKSRAQI

SEKALKRGLLKPPGSRELASLFRDEVSPVLPSSRRRRNMSKIRVLFSQSMDKETIKLQTK

ILIYFGLPLASSVSDATHFVAEKFARTRNMLEAMAMGIPIVTPSWLECCDEARCFIDEKK

YIMRDMKKEKELGFNMPVSLSRACKKPLLENRRVLVTPHAKPSKEILKSLVAAAHGQPLE

RITSPIMKNKNFEGTFVISCEQDRSVCLPFIENGLEVFDSELLLNGIVTQKLDFERYRLF

HDKAL

>Sbicolor_BCP3

MSTTRVQGRLNYLNSQEPGDESQATAIDIVERLLVEDDIGTSQKISAEPMSGTKSASILG

TKVAQCLAKRGDCSSPLQKAGIFDWADDSSNDECTTIMISRKKQRIHANTQAKNLTSQTI

VGSITECNGRDSGLNSFKKPEPVGSTDDLYDAYDIGPCTQMAAEAMEALSNASTVNYAVR

ENAHPESSVLRTNLGKESKANKLCSESPIRRQIGGTSSSVKKHPSKSKDKKNQKQLAGKA

KGSMDSITIQGDTNHEVSEGIKRSSPSDSNIVGSDAVIHPKRKRTYTFISRSSKVQFNKA

GRSATLRSKSTEVADSSTTNPVSISGPDLNQLARLEKQSTSAQEDHNSSLTNREPLKELN

SKHSQFRTQISKKPLKRGLEKSPGSRELASLLRNEAPPVLQTSRQRRNMSKVHVLLSQSM

DKETIKMQTKILIYFGLPVAKTISEATHFVAEKFARTRNMLEAIAMGIPIVTPSWLESCR

EARCFIDEKKYIMRDMKKEKELGFSMPVSLGRACKKPLLQGRRVLITPNAKPSKELLTSL

VVAAHGQPLARITASTVKNKKIEVAFVLSCEQDHSVCMPLIESGLKVFDSELLLNGIVTQ

KLEFDRYRLFP

>Jascendens_BCP3

MNMLTCARECGRLDYLGSQEPGDSSQANAIEVVDKLLVEDDSETSQKVSMDKPSGMKSAT

MLAAEVAQCLAKKVEYNCSFEKPGVFDWVDSARPENGTSGNFSRRKHEAVFKNQVKNSQV

KRFSGSGVGTKSRDLLKCIAEEPSLNYLEQQAEPISAFDGTYDAYYIGPNTQMAAEAMEA

LVNAPTVSYDAREDAPPDCSIMSIDAEKEVETDNASSVHSSVQKLVCLPHSSARMATRSK

QSNVVCTRLNEENSSSLRGRSSKSTVRMNVKQMPGKAKRSKDNEVLTGNICHEGSERITG

PNPSFPHLLDQDTLVHPKRKRTHMFTSGNLKIDFNQGSKSISVKAKTTKVMQPSAAQGGN

RTNENVLPNIIDGVKRKRRSYLTNQRVSAASSTDASDGALAIVSKDRVLHKEVSHSNQVA

GSLLSNEQEKQSAPQGKVQHSSLAHATPLREINNTLLRSRMRSSGRPSKKGFSKSPGFRE

LASLVRNGTSSDLQCNGRRKKDMSTVRCLFSQHMDQEIIKQQRKILARFGVPLARSIAEA

THFITDKFARTRNMLEAIAMGKPVVTPMWLECCGLAKCFIDEKKYILRDLKKEKEIGFSM

PVSLSHACQNPLLEGTRVLITPNAKPGQELLKSLVVAAHGQPLERITGPMLKDKNFVGAF

AISCEEDYNICLPLIKKGLEVFDSELLLNGIVIQKLQFDRHCLFGGKTI

>Platifolius_BCP3

MRIMTSTREHGRLDYLNSQEPGDSSQANAIHVVDGLLVEDDLQTPQISMDQISGTKSAST

LGTKVAQCLAKRVKYSCSLEKAGVFDWVDSPDPYSCITGTVSKKKHGVDFKNQMKYLEPE

RFNGYGLGTRVRELRECIDKESRLNHLVEKSEPVSAAADDSHEEYDTGPNTQMAAEAMEA

LFNASSVSFDVRDNAHPKSSVLRIDSVKEIEINNISSVHSSAPKIICMPQSVGGMTTQYK

QSKVDDTRLNGESSSALTRHPSKSTASKNAKLMTGKAKRSMDIGILLIDINNEEPKGTMG

SGADDSYFPYLLCKNAVIHPKRRTYVFTSGNSKVEFSEASRSTALGAKATELMQLSIAKT

VSLSDKNMHPDITDGMERKQRLSLTDQKTTVTSCTAASDGTLPTAIKDTVFHKEISHFNQ

VPGTLLSIGQEKHSASEDKVQDSSLTMRVPLRERNSAWPQSRMHTSNKPLKNVFSKSPGS

RELASLVRNEPPPGFQSNRRRKRYMSTVRVLFSRSMDQETIKQQMKILTHFGVPVAITIS

EATHFVAKKFARTRNMLEAIAMGIPVVIPSWLECCGIAGCFIDEKKYILRDLKKEKELDF

SMPVSLSRACQNPLLEGQRVLITPNAKPGKELLKSLVVAVHGQPLERINVSRMKNKNFKG

AFVISVEEDYSICLPLIKNGLEVFDSELLLNGIVIQKLEFDRHRLFHGRTM

>Acomosus_BCP3

MSLKWSEDNSIHESISRTDYGGLNYLGSQDPGELSQVNAMHVVDKLVFDDDIGLSQETST

VKATEMKSAPSLSAQVAQCLVNRENPNYSVGKARIFEWVDSPEDEAGGGFFSKRKPFVSK

NRINALESKNQLPKHEQIDTCEDRETKNLETYRRRNTLTDSNSMPLPHGLERSERPYISA

AKLKEQSDYMDTNSSLGFIEQQIEATDVLGGNYDTFDIGPNTQMAAEAMEVLFHGPPLNQ

ESIEDSSLLNQIPSTSPTKGSRREKATPVKTSLPKRSKRSKEVDMNDVNNPQMLHEWCKS

EKELAQVVSAEDSFRHPKRRRTHVFPLGNSNFGNSIRRRLPDILDKAKRETRSTKLRISP

SNLDDAFDNSLGIVPYDPALLKDVKEPNSDESKQNISTRRAPVTASYKKGFSKVSASREH

IRLEKNERSSDRMFTRRKKKDMASVCVLFSQHLNQDKIKQQKKILARFGALTASSIIEAT

HFIADKFARTKNMLEAIAMGKPVVTSMWLESCGQASCFVDEKRYVLRDLKKEKEIGFNML

VSLTRASQYPLLQAKRVLITPNVKPDQELVISLVKAACGWPLDRMGRSAMKDQKVSEDFF

IISCEEDYKICLPLVKKGVKAFSSELLLNGIVIQKLEFER

>Sviridis_BCP3

MSSARVPGRLDYLNSQEPGDESQATAIDTVERLLVEDDIETSQKTSTDPISGIKPASILG

TKVAQCLAQRAEYNSPLQKSGIFDWADNPNNDECTAIMISRKKQRIHANTQVKHLASQRY

CGNGSSTRAGFISDCIGGVSGVDSFKKPEPVGSTDDLYEAYDIGPCTQMAAEAMEALSNA

STVNYVVRDEAHPESSILGTNLGKESKADKICSVESQIQKRIVGSSSSLKKHPRKSKNMK

NPKQMAGKAKGNVDCGSIQGAINHEVSERTKGSGASDSNILGSHAVIHPRGKRTYMFISR

SSKVQFNKVGSSTTFTSKSTEVADSSTAKPVSTCGPDFNKHARVEKQSTSAQEGHNSSLT

SRVPLREINSTGPQSRTHMSKKPPKRGLLKSPNSRELASLFRNEASPVLQSSRRRRNMSK

VRVLFSQSMDKETIKMQTKILIYFGVPAATTIREATHFVAEKFARTRNMLEAIAMGIPIV

TPSWLQCCGEARCFIDEKKYIMRDMKKEKELGFSMPVSLGRACEKPLLEGRRVLVTPNAK

PSKELLKSLVVAARGQPSERITASTMKNKNFEGAFVISCEQDHSVCMPLIKSGLQVFDSE

LLLNGIVTQKLEFDRFRLFH

>Bstacei_BCP3

MSIMTAARERARLEYLNSQEPGDASQANAMDIVDRLLVDDDIETSQTITIDQISGTKSAS

TLGSKVSQCLAKRADLSCPVEKEGVFNWYDTPNHDDCIISNISRENPRVHVKNGISYLDS

QRCGGYGSGTRAGAMLECIDEDSGSIYLKKPESAVAASDLYEAYDVGPNTQMAAEAMEAL

CNASTVSYDVRESDHLESSLGKEIEVDMVCSVNSQIQKQKLTCLPQSSGWMTTQFKQLKV

DGIKRPNGGSSIPLTNCPSKSKTMKDTNQMTRKAKRSMESGVLGVTNDEASELVMGSGPD

DSNTHCLLGKDAIIHPKRKRTCMLTSGSSKVEFNKATRSTTVRPKTTGVIELPAAKAVCI

FDPGTIKGTNMSHKSFLANHEVSDANSADASVGITTRSKVRAIQNTSHSNQPEGTFSAGL

VKQSTSEQKDNDSSFSSRVPLRKLNSTEPQSRTHTTKKPMERGLTKSAGSRELASLFSNE

VSPVLQSSRRRKRHMSTVRVLFSQSMDSETIKEQTKILMHFGLPVATTISEATHFVAEKF

ARTRNMLEAIAMGIPVVTPSWLECCGEARCFIDEKKYIMRDAKKEKELGFSMPASLSQAC

KKPLLEGIRVLITPNAKPTKELLKSLVLAARGKPLERFTVFMKNKNFGGAFVISCEQDHN

ICLPFIKNGLGIFDSEVLLNGIVVQKLEFERYRLFRDKM

**EUDICOTS**

>Carabica_BCP3

MEGEYENPVPLDDTIALGSPGAEAHLRTLDINTEVLNTPDSIDCGIAQSLDDIVDDSMDA

IPLDIDKDGSEVVGKIFPLKENKTSVRGHGSCMEKRKMNALGRTVMLDKDVTGHSIQQLA

STRRTDERGDTQKHELASSHADTSNHKQQFIQDSGTSEYEAALPDHAYNIVQKDYKSLES

IGLSVVDALDFVDHFLAVNNQYLCEKFENAIADGTNSPPPFTTKGAQTLASRTNLATRAR

ELGMFDWDEKQATERNSDKKQDEKFAMGSPGKPSEQDQSATNGQDSFEFGFDTQMAAEAM

EALICAPSFNSREQCTYQVPKNEIHNSSVNASKDEGLKHSANKKVADSGPQINRKRTVKL

RSPTSRLNGNKPRQFKKHSENLTGYNSPSSINKNLIYREPLAAKSSNFGHALTMKKSICG

RSSRVISQQKNGAPCESENSKGAGNDLVSLNFVGRNQHGEEMLFKKFRRSSQKTKRTEFQ

PSMAPVSSFKRRISANLEFDVPKKRRKKRLGIGVCKTIESKSLMSGSDGLAEVTRTKGPQ

QPRSTSGSFGTIDLLKLDPWCYPKQKRTHKGVRHQSNGFSNSSTLLTLADDQNKNKYPTE

NRLSSWRNCGPLLHGQTEFGSSPEKNLPGVCESAITSEEMIIMDLDRVKSSELYGKSDMM

GTIWSADCSENIRLGAVSADIRASQTTKTDVRIPGKKLISRPSLMKELTRLGYNKSLPNF

MPRELRRRRRNVCVLFSQHLKSNVLNQQKKIMARLGFSIASCCSDATHFVIDGFVRTRNM

LEAIAFGKPVVTHLWLESCGQANSFIDEKSYILRDSKKENEIGFSMPASLACARENPLLQ

GRRVFITPHVKPSIELIQSLVKAVHGKVIKGIGRAARGENFISKELLVLTSKEDYVTCLP

LLEKGAAVYDSELLLNGIVIQKLEFERHQLFTNCVEANCSKKCPEKN

>Carietinum_BCP3

MTKTQPSSAPGDEVEDANEDEKYQETELFEDTLVLDSHFTETDVEMLNLNTEIVEDSEPI

ENMITDTTCEDEPEVVLDSEDEEMNCTGKVNVAKGFLEDKSSPVVNNYSVLSKKRLFRLP

YEQANSTATTSVKSCTGHKGAAIDAESFDGNNHQYLPARLYRAHSPEPEDSIEDALGFVD

RYLSSNNVDMFQAVQNGKITMEKSPHVSSAKGILSLLSKKTKARTQIEDKDPFKWVGSDQ

NEKEAEIFCKKVNASSNFGNHGLTYKRRRQEKGSHIQNNGNCSASNRCDESSVQEPRSAA

DNKNFLKELDIESCATREDVKTYSSVACLEDMSDIGLDTQIAVEAMDALARLPPNGFHFN

DTHQPENTFDGSLSDLEQNHQKDSLCKESPGLHSIALKSYKRNVSSYRYSKVTSSSYGKH

TNNQKSDPVSGRLKKKIGSKSTVEGQFKNDSSSLMCSKHVSLEQVCLLGEDVSFKPAAKE

TKNQNKSIRTRIKHQPSHPTERNNNIEEEGIIRDNRMGNCLVADPVNINVTTKCLKLSTS

SCGVATKGMLNHQVQVNPQLSATSSFSKIYSWTYPKGPRGKRKRANARRIFCIDNKESNF

YSARSLEVRDVVHKSCLPPVSSGDAIKFQNLHNTHPLLLAHVKLISDKSVAQSSSEISAS

VAPSEGIQNSNANHKYNEHCKKACEKNLRKSSLLKELIRLGVPKSTSEMMKDMRHRRDMT

HVRVLFSQHLDDSVLKQQKKILARLNISTASSSMEATHFIADRFTRTKNMLETMALGKLV

LTHLWLESCGQANCFIGEKNYILRDMKKEKEIGFSMPVSLARARQKPLLKGKRVYITPHI

KPDKEVVAGLVTAVHGQLVDENQIFADKNDDILDDLLILSCEEDYAICRHFLERGAAVYS

SEVVLNGIVIQKLELERHKLFANQVTKNKPGIYNRFGKVYRRRLRPLS

>Dcarota_BCP3

MIKNNPMMLPSCDSVPAASSPGIKDEASDGI

EYQDTEPFDDIIIADSPLSETQLEILHADTEVLDSDIDKVVLDSDDEGIQSTETVSLMNN

LPSGKTDISLEGDTEVMDKRQLSPASNQSYVGYFRRRGSKIQSCSIYKGSDTGSKSIYAE

ATWKKQHIVGGYENAVECQAAMQGNIEEVAVDNRNWGCDGKITSSADFIKSIDVASNFST

ADARSEREVPLSKCEAVEGREGTSWVSNGHDLSPLSYVDSQEPGEHSQANALSVVDLYLS

GIDVGMSPDVQRGKVTKIVSPPSLFRKGSQALARQANLRSTYGKFEVFDWSGNKTDELAG

FHVKSNKEGLRERSTKSQHFSDLNPQKDNNFGVEKLQMAVAHNEHKDSTQMTLKINETSE

SMQATELKSDTNITNQLDNHADVESFAQQSNMDVLERDAYDMFDVGLDTQMAAEAMEALM

YVTPPSKNINAPPALENSPEDTLRSEVVTGASKCSSFHVGDDSEVTAKDPNRTAQSLDML

NKIKFSPSELDANENKTREIKKLMSQTLPVKEKNIKEAGSSKSVKRDSYIVKCRGKVKSS

PFACSTRQFLVHSPQNVEGPPCNSRESLNCIVKEVDCLERGTKRRKLNSVNSQAVDIKYK

LPKPVTNAHCGATNSNSKKQMQTDSSAAVTTSHLKLDIWTYPKRKRTHPKVLSYLLKSGN

HYASSSPGLAKNISTRSVGISQKSEADFSANHSILQAVRKPRSICQVQPLLNKKFVESFS

RENAGKDLIPDVSSIAEYNLLHAHGGAGVGKQLLKKISPRSPLMKELSRLGFAESLPEFV

SKDLRRRRNMANVQVLFSQSLNDDIRKQQKKILARLGISVASCCSKATHFITDRFVRTRN

MLEAVALGKPVVTPLWLENCAEAGCLVDEEKYILRDVKKEKEIGFDLSVSLNRARHHPLL

KGQRVLITQSVKPCKEMIESLVKSVSGQPVENILTTAQDKLIHEDLLILSCEEDYKTCVP

FLEKGMPVYNSELLLSGIVTQKLDYLSHQLFADYAKKNSMKVYSRRAKIQI

>Fvesca_BCP3

MASHSHSHPSGFKKLKDESDHNHPQFQDTEPFDEAVVPDSPSMEDTQVLDYSDCDEKMRT

GFEEEVVLDSEDEEVNGSRLVTLGNMSSDQTCAAGLSYVDSQEPGEESQANALDFVDQFV

SLNDDLSLNYPGIDTRKTTKVKSPPVTSTKGTQSLARMVELRSQNEKAKAFEWVDLPENG

VFCYLDKKMESSLVNPQGKVQSNNGARKEKVRHANETKSISSGDGRKEKSIIRNFNKDIT

EFEDKIGNESVNALEDHLNEKPTELQLEPSDEGGDVVDMFDIGFDTQIAAEAMEALAYGS

HPGCNTVDVGSGGATESTAHIEHCSLEKSDFSNVAGISKNANRRNRSTRIPRKAGASSET

PSKKQELYPDLAAATNVTKSRFFGESCSNSAEASLCTESPKSVKKMKLQTAVDYSKDGSG

KHQTTQSMLGGRSRMMEVGSNNGIVTYRRKKRSLNAQSPEIISSSEAGSDVVEKTNNVKK

NDIVTYRRKKRKAAKESPVYVSSEGNSSSKACDLKLGLRSHPKGKRTGRNARKNSCRAPV

PSTPFLLLDKDSSPSDERPERSNNMAGKEQSLCSPPVVSLNNNSDRTLLELQSGKLVGTD

PTFSGTEFNSTTGSMGSSKYVSHDYHKKQRYKNLPKSSLMKELIELGVSESMPDFTWKDL

RRRRSMAYVQVLFSQHLDDDIIKQQKKIIARLGLALASCSMDATHFIADQFARTRNMLEF

IALGKPVVTHLWLESCGEANCLISEKSYILRDAKKEKEIGFNMLVSLDSAKRCPLLKGRK

VFITPNIKPGKEMMTSLVKTAQGKPVEKAQISTAKGKKILDKLLILSCEEDEAICLPFLE

KGVAVYNSELLLNGIVTQRLEYNRHQLFTDVVKKVRP

>Hannus_BCP3

MAGFSDHEAGNKVDVSVEDGHMIATAFDYTEEEETQIVNMDEETEELDITNFAGDLRALM

VEDSDDVIALDSEDDEVISEPKDGKVRGSLWRRVSAVGVQSFEVKSSAGSNLTDKVTGNG

PNSLQHTRYQETLTGMSSEKNIISNNENVANVDQDQKTYSNNFENANEAVNESPEPGEST

QADALKFVDHYLSVSVENSSPEVKIPKTIGLQSPFTSFAKGSHELAKKANRMNRIGISTF

DWDGNQPDSDERLFDQHVEEELPKCPPEKDTFDVSFNTQMAAEVMESFLCAPPPAIYANE

NEDTNKKTLKQFSSPLRDVINLNENGDTKKQKELNVESSTGNLKVRDKSSKVVTKRKQNQ

DLEKENIRFVYKRRGSLGENQKVKTFSPVASRTRRGSTVKIPKRTRNLCDLEYITHERKT

GDLDTISGNIKFEAWNWPKKKRTRRNMRQNAALSGNTVSVVGPSGTDGNFREAFLMSSVK

RKARSGSRFRSTAGINSWKGSLMQKVEVRVLFSQNLDDAVVKQQRKIMKKLGISTAKDCS

DATHFVADRFARTKKMLEAMAFGKPIVTPLWLDGCEQATCIIDENNYILRDAKKEKEIGF

NMPVSLSRARSHPLLKDQRVFITSNVEPDREMIKKLVEAGQGQVIEGVQQASTEYKISNS

LLILSCEKDYEECVPFLEKGAAVYSAELLLNGIIVQKLDYARHRLFNGHVKMKQYARKRR

RNGIQHLHGG

>Cpapaya_BCP3

MELNLLFKFEGNKGCSIDYSDNGRKDPSQMTECGHETGSLNYISSQEPGELSQANALGFV

DEFLTFNAVNFSPGINTKRTVNKKSSFSGAKGPQILAKKIRPGTPVGERDTFEWVDIDHH

GGGEVLKGVKKSVGYGSSIRQETAGHQNGRYLTDKENSSLDDKCEKNVENLDHSCSRFVG

NIHTGTRRIEQPPEMNPMQFNKELKAEALEQQLDDGLDVFEVGINTQIAAEAIETLLFAP

LSGCSIGNANQGTGSLEDFPNYILMGKFHSEQHSLQKISSNNFEGSNRKLNQGICPARKY

CRRDSSAAEKTANYQESDPKLAITSKAKRSKTLTVGNENARNPVNTNENSGRKSFRITEK

RKAVGAVSGKNIEESENCSSSIVAEQVSRVKGQSDGKAIVLQTTSTEERFEGNKDRSDNT

GKRKNDDMQNGILTYRRRLSGRKKHSKFSSGVFWQTVESKLNQQEQTVSEVNMIPSFLNL

EAWNSSRRKRSSCKLQRHSVAASNLQASITVVKSSSKCKLSSFNGKKKNDFGNSPNYSTQ

ARGSKLSGKVNCDKCLNEASFQNAFDKCHKIPYQKNIPKSSLLKELMRLGMVGTKVDSAW

KDLRIRRGAAYVRVLFSQHLDDGIVKQQKKILARLGISTASCSMDATHFVADRFVRTRNM

LESIALGKPVVTHLWLESCGQANCLIDEKNYILRDTKKEKEVGFSMPDSLAHANRHPLLK

GKRVLVTPHIKPDKEMIVYLVKAVHGQVVERSQISMTGDQKISDDLLILSCEEDEEICFP

FLDKGAAAYSSELLLNGIVVQKLEYERHQLFLADVVERRQYKKRRSRRMQ

>Lsativa_BCP3

MLFSFLVMEQRMLWILVYSVSGNNLNVSVEDDHLIAGDFDFTEELESPLQNLDADTEELD

ISHFVGDLRALMVDDSEDEIVLDSDDEAISEQKQGKIRGRLWRHAQSVDVRSSAGSNLTD

KVTDNIPSERSEERNLVSHNENETGSRDTDTRCSKGDKLVNLNASSNNFEGENAVVDESQ

EPGESSEADALDFVDHFLSVSAVNSSPEVKILKYDGPRSPFSSCAKGSQKLAMKTSFMTK

IGVSTFDWDSDQPDHGGGFFLEKKTEPDIQRDKHLSERCKSKQLPGNDSNERDEPPDMFD

VGFNTQMAAEAMEALLYATPPHIDVNEHKRVKNPTTENPSKKFSFPLSANCDSKTNGISF

KQKRVANRRHKMSTSFHNKNQRELNLELLNLGKDENCTNGDNLTEPKKKVYEKSLKVYKR

RKQKQDADKENLKPDNEVKTFSPVASRTRRGSCVKRSQRTADATCNGKEIDVLHKRKSGD

WKFDTWKWPKKKRTCRNRRQNAKLTNTLNVQSPVVKGGNGEMEGNFKEASFMSSVKRKAR

SASIYRSTSGKKLSNTKCVSMRIPKTLNESEHSFESIPALGGEEKIKDLSSNGKNKTPSI

MSGASRTSDQGLPRKQLHKKSSTSSSLRNELPRLGFSESAPDFMSKDLRRRRSKVEIRVL

FSQSLDDDVVKQQRKILKKLGTCMATDCSDATHFVADRFARTKKMLEAMGLGKQIVTPLW

LESCDQVGCIIDEKNYILRDAKKEKQIGFSMPVSLSRATTHPLLKDRRVFITPNVEPDRE

MIKNLIKAVHGQVMEDIEQASMECKTSDDMLILSCEQDYVSCFPFLDKGVAVYSSELLLK

GIIIQKLEYAKHQLFKGHIIMKRYAKKRKKNGSGDLDVV

>Rcommunis_BCP3

MLLISCLKIQNPSMMQLPLIVPSMEHLCEDEGNSYVKLDRRTEGNAVGSQRRQPAPPSPH

LDDFKETYVGSIVGEHCGTEQVDESTDAGKIHPPMLTHDQVFAELNYIGSEEPRESSQAN

ALDFVNHYLSLNNVDSSPGTKPRNAIRKKTSPVSRIKGCQSLAKQIKTGTAVGNDGIFEW

VDNDHCGRIDFFSKRTGTALGHGGSQQRSVKGHQKFGRVESKEASCSGKKLQEKSRNLIK

EVTASCYSDSRPAVGRAKEINRFEQASETNFENVSVKNSNEQSHEVQLGRDIDAYNTEEN

APDTFDVGFGTQMAAEAMEALSYGLPTDICAGNVSQCPQSSRLDPSPGIEKSKIHFRSPC

LQKGAFSNSEGIARTSKQRERYASSSRAHSSYQELDNELEITTRRKRVKSLAGKLNGMNS

MYQNEFSARESAKPVKKRKVGEENTKECEDCGSSSIPFESILLGKECIHHGEPVTQETGR

WIREGKLEGKKDGSDNLGYGMNDAVKGSTITYKRKRSSVHANAKPSGILNVGGNCTKEKP

FTHQRHWITGDELEGTKDGPDGAGERMNAITKGKIITYKRKRSAKVFELTSAEGKSIKLC

CNISEVSRNNKLIGQKQGSLEVSSLTGSLDFNAWSCPKGKRMPRRRPSHLNEANNPSILC

IKIDRKEGHKKPYNKILPKSSLLKELIRLGIQEPKPDFTWKDLRKRRDMSHVQVLFSQHL

DDDIIKQQKKITARLGISIASCSMNATHFIADKFVRTRNMFEAIAHGKPVVTHLWLESCG

QASCLIDEKNYILRDAKKEKEIGFSMPASLARASQHPLLKGRRVLITPNIKPDVEMITSL

IKAVHGQVLQNFQISDLKIPDDLLILSCEEDHSICVPFLDKGAAVYSSELLLNGIVIQKF

EYERHQLFTNRAKRSGRPGTR

>SoleraceaBCP3

MANSSHLQIFGNKLENVKAVDTEPMDYNMKLADPLLEIQVGGVLNDTEELYSPRSIGDSQ

TGYDTEVVPDSEDEMTDPVDSKQLVHGRVLFIQRFARGLDKVESPFPPLLEKLECSDINA

DVCDDGVHVSGQTSNINVFQGNDGCSGTVHHGMLGTDLNANYEEVSKVNDTCLDNAVSCD

INCNDDGKKLSEENKDDKCGKISQSNRKDVRIVYTRKRKRTCNVGANGKEDEKRSPDRTD

DEYAGLSYVESQEPEDVSKALLFVDKYVGSNVMYSSPVANITVKDKSLSARSVKGGSSLI

RWAALKNREEGKRIFEWDDRLDGNGHSDPSDSSGQLSFRSKKYDQKYMRRSESVRRMIPK

DIGDPIFSRKEKAEGKNDLTQTKSTEVVYNHINAGMTEKTHEMEANEVSKMLDQQVSEEF

KVMQLEAGAVHEELGYAFDVGIDTQMAAEAIQALSYAVPTGCIEVDADKFQHKMLDRPLE

SRQKRTLAGDWSFPKKVRSASRGVVSRPEESRRLSCSNNRNSEIQAFKLREKRNSRNSGN

DKCVDRRISDISPKADVTHKRKVEGAKEVKLDGSKENLRSSVSTKQASTHRVKCRRKDSG

SYPDAKTDVKIDLYDKRKRRVNDKSEISDKKKNTPNPLYEKCMNAKDMKASEVVDDSANN

SSVDRRNHQMEIALFDRDNKGGAKVLSVSDPVQNLPKLPCTKFNDARSTSTGQTKAQYAP

ARSTNWYSRDYPRGKRTCKSMLRTSGGFRDFNHSFVRVSGEENGCHHLKGRLDLKGCCTP

SYTGSSIKKKRRSSAYISPCRLLSQKNSEQNLVRHNADEMMESGVVSEHLIESTITLQHD

KLDDNIDSASTVADKKRKDVPTILCDKNLISEDTASPSNLIDDCLRHGKKKASPSLTREL

VRLGFRESIPEFTSKDSRRRKTMAEIQVLLSKSLDERTLLQQKKISTRFGVSMASSPSTA

THFVADKFSRTRNMLEFIAQGKPVVSHMWLESCQQAGFYIDETNYILRDIKRERELGFRL

PFSLDRARKHPLLKGYRVIITPNTKPGQEMMTSLVEAVHGQVVDMPADHIRTEKTDLKNL

LIFSCEEDQAYCLPFLKRGASAYSSELLLNGIVIQKLEFDRHRLFK

>Csativus_BCP3

MVSKLGNDDSGGSFDDFELLQNTLSFEDSVGFDNRVVRLESPFVDTEMVYGLDDCSENLC

IPTFEYEDDVVLDSEDEGINGSRVIRVSSSLSRNEAEQEVKSDAQEENMVLDFHSSDHKP

CDAGEQVSSNCFNGLDMEKDSSQLSTRLSYCSSQEPGESTQVKAIGFVDHFVKLSTTNVN

PSQGIGQRKAARVQSPNLSRIKGPQSLAKRIGRKSIDETGNFEWVDINNQEAECNSFGKC

KKASSDLSNCRGQSYTTKPHNMAKLSNIRDCLLKRYEDKEEASAGIKFQTDSTASTFPHP

RLDIYSLETDGMSSIEIENRSNEKTNAKLVEGQLEFMDDERDAPDELDIGFSTQIAAEAM

EALCYIPDNDTLANACSSENALDSVSCSMIEHKPHLNSSYPQTIGGADGKSKRTLKSKRK

LNAKCLNTSKVQCECQELQAVLITKSKAKRSWLALQGQSNYRKSPDESNYTATPSNLLSE

TGSCQLSQRGLTIGDQVSTRLMVGLCSHPRGRRTPRNIQSHPNRSKNQNNTSLAVDESCN

NSILMKNGKVGRNSNSKSTVVIRRMFYMGFVQSRFSNILCFTVGKNTSRSRSKFKVYHNT

SRRIMLPQSSSKELARLGVSESMPDLKWKDLRRRRTMALVRVCFSQHLDEVTLKQQKRVV

LQLGISIASSSVDATHFVADKFVRTRNMLEAIALGKPVVTHSWLESCGQASCFIDEKKYI

LRDTKKEKEIGFSLPVSLSRATQCPLLQGFKVLVTQNIRPGKEIIASLVKMSQGEPIEMS

QIFTGKNEKFPNNLLILSCEEDYADCVHFLKKGAKVYSSELLLNGIVIQKLENKRHELFT

DSTKRF

>Ppersica_BCP3

MAVSHTEAQTSDFPSHPSGNKLKCVDEDEEQQLQFQDTVPFEDTFVLDSQSMEETQLLDH

SDFGDETGTGMHEYEEEVVLDSEDEEVNGSRILTVKAHVLSNQKLSLHHLQCGPGGKDEG

SPPLTRLNYIGSQEPGGESSQANALDFVDHFVSLNDDLGGSPGIDHRKTIKEKSPPVSRT

KGSQRLARMVKLESQIERAKTFEWVDPPEEHGGVYLFNKKIQSSIDFVDCNQRSIVGHQK

FRLVDDNICISSGGESKERKIIQSLHKEITESEEKIENEIVNDLEEQLDTKLTGLQSEAS

AIGTDIVDMFDVGFNTQIAAEAMQALAYGDHLDCNSADAYQGMENTADVVSGGATKNTAL

LEHPSLPKSDFPKIKAINMNAKRRKRSTRTVKRRFASSQKQSQSRELDPDLAAQTTVKRS

RFFSEYSSNSADANGNLCRQSPKPTKNRKLERATGESNLRVLENNLSSSISAGRSLLGKG

QSQGHCKNYSSVSNQTRQSKSGGKLLGTEDGPNNPGERMNNVMEDGIIKYRRKKRCLNAH

PSKTIRGVEGGSNEPVEGTNNVKKDDIITYRRKKRISAKENCPKLCVSSEGDRDINASSL

ILDLWSHPKGKRTRLNARGNSRRTTVFFTPFSIVDGKDSCSPCDEMPESCNDIKGKAQSL

LLYCCPPQHSSNKDSDRTLPALQSGKLDSTDPTSLVDVKSQISVVLSGSEHTAPSNSTTG

INVGSSKYLSHDYHRKPFNKNLPKSSLLKELIGLGIPESIMDCTWKDLRRRRNMAYVRVL

FSQHLDNDIIKQQKKVIARLGLSIASCSIDATHFVADQFARTRNMLEFIALGKPVVTHLW

LESCGQANCLISEKSYILRDAKKEKEIGFSMLVSLDFAKRCPLLKGEKVFITPNIKPDRE

MMISLVKAVQGQPVEKVQIPAAKDKKSLEDLLILSCEEDRAICLPLLEKGAAVYSSELVL

NGIVIQRLEYKRHRLFTALGKRNRP

>Tpratense_BCP3

MANTQPSSSPAGDEVRDANGNEHYQETELFEDTLVIHSPLIETEVQNLDLNTEVQNIDLN

AEVQNLDLNAEFVEDSEPVEDMITATTCEYEQEVVLDSEDEEMNNADKVTVAERFLEDGS

SPVVNNSLVLFKKKLPKLPCEEANSKSNAATSGKSGTGDERASVDAKSFDDNKHQSLHAR

LYDVRSPEPGDSTVAALGFVDQYLSSIDVDLFQGVQNGNIAREKSPNLSSARGSISLAKK

FKARTQNEDKDPFEWDDSVQNEKEAGIFCKKLEASFNIGRHGLAYKRRQKKGSHPPNQGK

PSASNRCDENLVQEPRMEADNNNYFKELDVDLCATRENVDIYSSVARTEDVYDIGLDTQI

AAEAMDALANMSPAGFHLSDAHQQENAFDASLSDFNQPHQKNSPSKENPGSHSIAVKSYK

GNVSSRRFGKVTSSSSCKDTNNQETKPVSGKMKKTMGSKSTVEGQFKNNVSPSICNEHVS

HEEVCLLGEDRSFQSASKEPKIQNKSKWTRVKCQPSHPRESNNNVEKDTIRYKRKGNCLV

ADPVKMDVKTKRLNSSISSYGVARKCSLKHQVEVSPQLSATSCFSMIDSWAYPKGPRGKR

KRANAPRFLCIDDKENNVYSTKILEGRDVVHKSCLPPVSAGGAIKFENLHDKQPLLLAHV

EILSNKTVVHSSSEISASVVPIEGIKISNATHTSNEHCKKACDKNLPKSSLLKELIRLGV

PKSTSEIMSIDPRHRRDMTNVRVLFSQHLDDSVLKQQQKILTRLNISTASSSKEATHFIA

DKFTRTKNMLETMALGNLVLTHLWLESCGQANCFIDEKNYILRDMKKEKEIGFSMPVSLA

RARQKPLLKGKRVYITPHIKPNKEVVASLVTAVHGQLVDENQIFADKNDNVLDDLLILSC

EEDFAICRHFLKRGAAVYSSELVLNGIVIQKLELERHKLFVNQVTKNKLGRCNWFGKVYR

RRLMPLS

>Pvulgaris_BCP3

MARGSSPHSLTSSPPAGYKVKYASDDDPSRETDLFQDTLVLNSPFTETEYENLNLDTEIL

EDSEPAEDMTTGKMCEYGQVVLDSEDEEMDDRHVGKEFLEDETSPTVKIPSILFQKRQPK

PPCEQVEASTTTFGSSAAEHNMLEGDKRTLVDSDSFDDGNRLYPPATLNHIHSPEFGDST

QAALGFVDQYLSSNDVDLFQGVHRGKTAREKSPHVLNARGPLNLAKKIKARTKNEEKEPF

KLVDSCQHDNRGRMFSQKSEASSNFGRYKQTYTRTKKGGHLNCSTSNTFDGKLGQGPRMA

IENDNSLKEFNVHPSSIRENADVYSGETHIEDMSDIGLGTQIAAEAMNALALMPPYGCQF

NDTHQPENALDGSLSDLTENEAHLNNSSNIQNPSLRSITTKSNKKNASSSRFSKITYSSS

CKDTDKQESNVVSDKMKKRMKGKSTAEGRFENNNSLPVCSKHVLLEEVCPPGEYTSFEPA

AEESEHRNNESGHQRIKDQPSLRTEENNNDKEKGIKHKRKETGLEADPVKLGARTKRLKF

LTDSCILSKKVRLNHLTEVSPELSATRSFSWTDSWVYPKRSRGKRKGANMGTKLNATTVL

CTDGKENNVFSTRSIEDQANASCVDDGGCLLQGDFVPPGSSGDAMKVQNWPDMHPLFLAH

VERASNRSVAQSRSGFRATVVASEGVKVSNVNHSYTEHHKKLCVKTLPKSSLLKELIRLG

VPESMSDMMWKDLRHRRDMTDVRVLFSQHLDDSIIKQQKKILKRLNISVASCSMEATHFV

ADKFTRTKNMLEAMALGKLVVNHLWLESCGQANCFIDEKNYILRDMKKEKEIGFNMAVSL

ARARQEPLLKGKRVYITPHIKPDKEVIASLVTAVHGQVVDQSEVCADMNDKILDDLLILS

CEDDYAICHRLFKRGTAVYSSELVLHGIVIQKLELERHQLFMSQVTRSSNCPSTSNRFGK

VYRRRRGLILDCS

>Gsoja_BCP3

MAEASSPRSQTSSSPGGYTVKDANEEDQFRETELFEDSFVLNSPFTEMEVENLNLNTEIV

EDTEPAEDMTTGTMCEYEQVVLDSEDEEMNDRGVGKGFLKDETSPTVKIHSTLFQKRQPK

PPCEQVDANATTFGKSTAGYKGALVDADGFDDNNHLYPPAMLSHIHSPEPGDSTEAALGF

VDQYLSSNDVDLFQVIHYGKTTREKSPYVLSSRGPLNLAKKIKARTQSEEKEPFKWVDSC

QHDNKAGIFGKKIEESSNFGRYKQTYTRKRQKEGGHLQGQGNCNTSNRCDEKLRQGPRTE

TENNNSLKELDVQPSAISENVNVYSSVTHIEDLYDIGLDTQIAAEAMNALAFVPPSGCQF

NDTHQPENALDGSLSDLTENEALLKNSSEIQNPGLHSITIKSNKKNPSSSRFSKITFSSS

CKHTDKQEPSLVSWKMKKMRSKPNSEGQIDNNTSLPICSKHVLLEAVCSLGESTSLQPAA

EEPKDWNNESRQTRIKDQPSHHTEGNNNVTEKGIKHKRKGSGLVAEPVKFGVRTKRLKLP

TNSCTVARKSRLNHLAQVSPQLSAISSFSRPDSWVYPKRSRGKRKGDNVGTNLNAPTVVC

IDGKENNVFSPRGLEDQDDVDKPCFPHTHPLCNASCVDNARCLLQGNFVPPGSAGDAMKV

DNLHDMHPLLLAHVELSSNKSIAQSRSEIPATVTASKGIKVSNANHTYTEHHKKPCEKSL

PKTSVSVLKELIRLGVPEFTSDMMWKHLRHRRDMTDVQVLFSQHLDDSIIKQQKKILARL

NISVASSSIEATHFIADKFTRTKNMLETMALGKLVVNHLWLESCGQANCFIDEENYILRD

MKKEKQIGFNMPVSLAQARQKPLLKGKRVYITPHIKPDKEVLVSLVTDVHGQVVDESGVC

ADMNDNSLDDLLILSCEDDYAICHRFLKRGTAVYSPELVLNGIVIQKLELERHQLFMNQD

TRNNPRVSNRFGKIYRRR

>Spurpurea_BCP3

MEAIQGQRQLFQDTVTFDDDTADLDSPLAETQVEKLGSYTQVLGDSLENAKHELPSQVIP

DSEDEEIHGDQLENAADEVSDVETGIRIKGNGVVGLQMRQPSPRFQWLKDIAEAFVSDGS

ASEDKGAGALINAEDSDEKTDCPRLVTCDQEFARLNYVDSEEPGESSQASALGFVDHFLS

CSDVNFSPRTELRSTNRKKFPPVLSAKGCQHLAKSIKTKTPICKNKTFGGVDNDQHGGVD

FFSKRMNDAFDSGGFRQRYVPGHQKAGYSDGKGGCRSDNDSEENYEDLHEKVRGSPQTDS

RVAGHSVKETRRTGQGYEFISENANASDNKPREQFCDLVLGHELGICSNERNTPDILDVG

FNTQIAAEAMEALFYGPAADSGAGEAFQDPKDPLVDSSKGVTNINVHLKELSYEKDALCN

LEDITRVPKQRKVYARKGASVSSWKQPSHQELHRDLPEITKRKRSKPLVGELTGRSFIYG

NESSATTSRKTVDQRKDEEPARRNNEECDNCENLSGSVESILPGKQQILQDPFASQDSLP

RLGAKPKRTNGGSANPGVRTGDFMEGSIITYRRKRSHSVAKPSKILTTTGRCPKFCFNTS

EGGRINELSQENLSSVKVSTSNSSLKLNVWSYPKGKRTRRGLPSHFNIASNQYTPFTIAD

GKDHHKKTHDINLPRSSPMKKLIRFGNPKSSPGFRWKDLRKQRDTAHVRVLFSQHLGDDI

TRQQKKILARLGISVASSSADATHFVVDRFVRTRNMLEAISLGKPVVTHSWLESCGQASL

LIDEKFFILRDAKKEKEIGFSLPVSLARANQQPLLKGQRVFITPNIKPEKEMITSLVNAL

HGQIMEKSQIFALKVPDDLLILSCEEDHVICVPLLDKGAAAYSSELLLNGIVIQKLEYER

HRLFVNGGKRNRVRKR

>Mesculenta_BCP3

MSPVSGIKGCQSLAKQIQSRTTFGKDGIFEWADSDHYGGDDFFSKRMDAAFSQEGSRRRS

VLRDQKAGHVYSKGSSSSENKCKEKFVNLDREVTSSPCSDSRTAVDCQKEINKMGQASKM

ILENNFVNNLNQHLPAVQLEQETDPCSIERNTPDVFDVGFSTQMAAEAMEALSYGLPSDS

DAGVYQYQQNLLVDSSIGVTKSCIHFKKPCLQKDALEGIARTSKQIKGSARKRSSSSSLK

RSGYDKLDNELAVTKKRKRSKSLAGSSNGINTVDKNKCPTRKSPKPAKKCTEEEATGKNN

TKGCENYGNLSKPFEPVVCQTGLRNAKGTMQATKDEPDNVGHRMNNGVQSSVVTYKRKRS

RLGPKPFGALRAGEKCAELCCEQLRKGGLFADKTVHWITGGELAETKDRPNNQWKRTSDI

IKNGIVTYRRKNSLSSAKLSESLSAEGKHAKPCCNISDTVGKNELTQKEQGGLEMSSLLR

FLKSNSWGCTKRKRTLRKRPSHSFGSSNQYISFMVIDAREGYKRSFNKNLPKSSLLKEII

RLGIPELKLDFSRRDLRKRKDTACVQVLFSQHLDDDIIRQQKKIMARLGISVASCSMDAT

HFIADKFVRTRNMLEAIAFGKPVVTHLWLESCGQASSLIDEKNYILRDVKKEKEIGFSMP

VSLARASQHPLLENRRVLITPNIQPDKKMITSLVKAVHGQVVEETQISELKIPDDLLVLS

CEEDHAICTPFLDKGAAVYSSELLLNGIVIQKLEYERHQLFRNSNKRSRHHNKRICHIMS

MKGGNIVGDFSLVKV

>Ahypogaea_BCP3

MADASYVICSSSPPGGKFKDADEDAQFQETELVNDTLVLNSPFTEPQLENLNLNTELVED

SDPSENVESGPTCENGQEVVLDSEDDEMDNGRAASAVRGLADDGTSPAARIPSMHFQKRP

VKPPFKQADSNGTAFSKAAIGEKGISVDTKMLNNIHSPEPGDSTQAALGFVDEYLSSSDM

DLLQGIYHIKPTREKSLHVLSARGSLSLARKIKTQTQNREKEPFRWDGGDQDDKGAGIWC

RKVNTASNIGSENQKEGGHLQSQGIRCAGVRCDDEIENMVQRHGIETKNESNSLKELDVQ

SSLMRENETPYSSVIHTEDMFGIGLDTQIAAEAMEALAFMPTTGCHFHDAHQPEKAPDGS

LSDLIENKGHLKNLLHKQNSGLQSITKKSKKRKHTENQEPNTVLVETRKMMKSKSTIKGQ

SENNTTSPIHSELVSIEEVCSTGEYTSFQPATTESKNKNKSRRPRKKNQSIHHTERNNNV

KADDTIRCKRKGADIEADPLKLGIRTKRLNLPTNLSRKTVKNSLNHQVEVSPQLSGSSSF

LINDSPSWLYPKRARGKRKKANVQDNLDAPTILCIDGNESNVWSINSIERQNDEDKSCVD

NSRCLSQGNSVQPGSADDTMKFESSLDKRSSLARVEISANKSSAQSSPEIPTTVSSSKDL

IMSNNKRTCDEQHNKPCNKCLRKSPLLKELIRLGVSEPTSASIGKDPRQRRDMTFVRVLF

SQHLDNNVVKRQKKVVARLNISTASSSMDATHFIADKFIRTKNMLEIMALGKLVVTHLWL

ESCGQANCFIDEKNYILRDVKKEKEIGFSMPVSLAQARQTPLLKGKRVYITPQVKPDKKV

VATLVKAVQGQVVDESQVCAEKDDKFLDDLLILSCEEDYAICHHFLSRGTAVYSSELLLN

GIIIQKLEPERHQLFVNQATGKRPGMSNRFGKVYYRRHSRPISAS

>Ptrichocarpa_BCP3

MEANQGQLQQFQDTVTFDDDTAVLDSPLVETQVEKLGFYTQVLGDSVENAKHELVSQVIP

DSEDEEIHGDQLENAADGVSDVETGTRIKGNGVVGLQMRQPSPRFQWLKDFAEDFVSDGS

AGEDKGAGALINAEDTDEKTDCPRLHTCDQEFARLNYVDSEEPGESSQASALGYVDHFLS

DNDVNFSPRTELRNTVRKKSPPVLSAKGCRNLAKSIKTRTPICKHKTFGGVDFFSKRMDE

SFDCGGCQQRYVPRHQKAGYIDGKGGCRSDNDSEENYEDLHKKVTSSPHTDSRVAGHSVK

ETYRTGQEYEFISENTSDNKPMEQFFDLASGHELEICSDERNTSDTLDIGCNTQIAAEAM

EALFYGPPADSSAGEAFQDPNDPLVDSSKDVTNSKVHLKELSYEKGVLCSLEDITRVPKQ

RKVYARKGASVSSWKQPSHQELHRDLSETTKRKRSKPLVGELTGRSFIYATKSSATTSRK

TIDQRKDEEPARRNKIKECDNYGSLSASVESISPGKQQILQDPFASQDSHPTLGAKFKRT

NGGSANPGVRTDDFMEGSIITYKRKRSHLVAKPSKISTTTGRCPKFCFNTSEGGRINGLS

QEKLASMEVSTSNSSLKLNAWSYPKGKRTRRGLPSHLNIATSQYTPFTIADGKDHPRKPL

NINLPRSSPMKKLIRLGNPKSLPGSRWKDLRKRRDTAYLGVLFSQHLGDDIIRQQKKILA

RLGISVASSLADATHFVVDRFVRTRNMLEAIALGKPVVTHLWLESCGQASLLIDEKNFIL

RDAKKEKDIGFSLPVSLARANQQPLLKGQRVFITPNIKPEKEMITSLVNALHGQIMEKSQ

IFALKIPDDLLILSCEEDHAICVPLLDKGAAVYSSELLLNGIVIQKLEYERHRLFVNEAK

GNRVSKR

>Csinensis_BCP3

MPYVIGCLFELTGENENGHKGIQDGAPFDDTVVLDCPLVETQMMMKLDFGTQVVDGSDCF

EEKSLQSFCEYEKEVVLDSEDEGVSRTYTLQADNLLLDSNASISELCSVVEGEKGCRVAV

ERTNMQKDLVIGSQEPGDSSQVDALEFVDKFLSLNKMDLSPECGMKRTVGEKSPPFSSVK

WSQSLAKRLKFRKTPVTKSAKFEWVDNDQHGKVDVFSKRTKASFEFGAVRQESVLKDHKA

MHVNGDGGRSSGNENEKRKELVNLHKEIAGSTHSDPKDDCMIEQVSKVDVKNHSDKEINE

DLHVESSDQQLDGCGTGIDTPDMFDVGFNTQIAAEAMEALSYAPFSGCTAFNPDQGQSNT

TNDFPKGVIMKKSRSEQALIQASNFCGLGSIATKSKRRKRSARKSAKNSNKVPDPELPRL

TKMRRGKSLIGEQFDCSANTNGNSGNGSPTLIIKGQAVGIVGKNNKVKEYENHTTSSILV

EQEEVGLRNITVAQESRQSLAGGTLKRTKKQSDNPRDWMEDGTIKYRRKRSRLVGNPTDV

TSANGSWSTLHSDSLAEPKSNIFDQKEETDQEIIALTSCLNLAAGGHPKGKRTPREMQSH

SNGAKEGNRSYMKSQGGAKYNSNTSFYKDMKGKPQSVNDSSPLNLPEKDLGKYFDEPGSA

VPFANNESAIISKEIPPGKYLEPPGSGYSSTINFTKGTNASSSSRVPSKYHRKPLTRNLP

KSSLLKELIRLDVSELIPDFTWKDLRRRRDMSHVRVLFSQHLDDDVIKRQKKISARLGIS

IATCPGDATHFIADRFVRTRNMMETIALGKPVVTHLWLESCGQASCLIDEKNYILRDAKK

EREIGFSMPVTYARARHHPLLKGQRVFITTSIKPDKEMITGLVKAVHGQVIDVSQVSAVK

EEKFQENPLILSCEEDHAICVPFLDKGIAAYSSELLLNGIVIQKLEFERHRLFKYHAKRH

QRRIKSRRGDVCLKRSL

>Tcacao_BCP3

MADYHKLTDTLLFESQSPSGSRLNEDGYGELQNTSVFDDTIVLDSPLNENQTQLENVCFD

TEVVDDDDLGNMEDVKAGQLLCEFDMEVVLDSEDEGVRKTKSESFVDGKITGRLEEKEVD

LPKRERQTQSLDADAVDKQFGAGKKGFSVNTDRSHGKKDVTRLSVCDHETGRLESVCSQE

PEESSQANALCFVDNFLTFNDADMCQRVEERTGARKKSPLVSTAKGTQRLARIINRGSPV

KEMVTFEWFESDQHGETDSFSRRMTASSEFGDFRQRTDKRHQNLHSKKERGLSNDHEEKK

ESVNLDKDIRCPSHSHSSFMEQGSKVSTKIEQESERNLINGSVKELDNLMRKESPWEKVE

ASGTAGDIPDMFDVGIGTQIAAEAMEALFYGPPIGCKAGDICEGPEDFHTDFPEDKTKSR

TDLEQHSLQKPAASELGDKAKQSIRRKRSTRRYSKEVFNSSWNCNYQVLHHTIKPKPCKS

KQSEAHAVSSNNLKKCVSCVSPSIPDEQTLFRKQLSRKEPVIDQTRHWDGASMKGTKDQP

AKHRVMTNNVKEGRMLIYKRKRKRVVADPPKLLNGKQKCSTLHSNTSAQALDGKLSEQEK

ISPQEAAIARFLRLIPKGKRTRRKVPVHYGGASNMLASLTSVGTEEHNLHSVRSQKMPED

DETTFNNFNMKGKKCSAISLLSLEHNSDESLSRRNCNEQIAGIVTNSDSAVTSTRISASN

LDRSKTVQTGKLDYMDSTLVINGSENYSFGILQKKSVESSGTECNTRVSCRESVNETSFN

NMPYVYHRRHCNKNLPKPSILKELNGLGVPDSISDFTRKGFRTRREVAFVRVLFSQHLGD

DIIKQQKKISARLGISITSCSMDATHFIADEFVRTKNMLEAIALGKPVVTHLWLDSCGQA

SCLLDEKNYMLRDSKKEKEIGFSMPVSLARARQYPLLKGRRVCITQNVKPNKEMITSLVR

AVGGEVVGTSQKLAAKDQKIPDDLLILSCEEDLAICGPLLDKGAAVYSSELLLSGIVIQK

LEYERHQLLVKFVKEKRKISEGNRHSRRLSKR

>Graimondii_BCP3

MDSDDGDDYMGHVEDVKVEQVMCELDMEIVLDSEDEGSCRTETITLFNCRKVTGGSEKNV

SKKREGQSPCSDVDSIDKQLSAGTKRSSMDHDRSDGKKDVPQLSIGDHEIRRLEYVDSQE

PGESSQAIALCFVDNFLSFNSVDLCQGVEERTRTKSPLVSSAKGTRHLAKIINRGSSVKK

VGTFEWFESCHQGETDSFSKRMTGASEFGDLSHQHLHNKGQISLSNEHEEKHARRHSLSG

FKDQGLKASIGIEKESEGNMVNGSFKEVDELIKTKSSSEKFEASSTARDIPDMFDVGFST

QIAAEAMEALCYGLPPSCKACDTCEAVEGALTDLLEGEAMSRTHLVHHSLQKVAACEIGE

VGKESIRRKRSARRYNKNISSSSWNCNYQELSHKLKPETTKSKQSKLDESVSQNNLENCE

TYVTAFTPDMQNSCRKQLSQEEPIIHQTRHCKGGANVKKIKDQMEKPRVMTNNVKEGSTL

TYKRKRKSVVADPPKLLSGKQKCTKLHSYASAETLDGKLSEQRSSPQEAAIARYLRLDTW

NCPKGKRTQRKVPIHSSGKSNMHASFTSVGAEEHKLDPVRNKKMPEDDETNSSNFNMKGR

MCTSLSWPSLESNSDESLSRQNCKEQVSGVTTNSDLAAPNIRESAWDLDGVNAAQTEKPY

YVDSTSIINGLKNHNFGEPLRNTIEPSGKECITTLCCKKGVNEASLNNRPYVYHRKPCNK

NLPKPSLLKELIGLGVPKLMSDFTHRGFRARKELAYIRVLFSQHLDDDVVKQQKKIAARL

GISITSCSLDATHFIADKFVRTRNMLEAIALGKSVVTNLWLDSCGQASCLLDERNYILRD

SKREKEIGFNMAVSLARARRYPLLKDRRICITQTVKPNKEMIASLAKAVGGEVVEAKDQK

IPDDLLILSCEQDLAICKPLLEKGATVYSSELLLNGIVIQKLEYQRHQLFSERNRRDQSS

RKH

>Mtruncatula_BCP3

MANTQPSSAPSGDEVNDTNGNENYHETELFEDTLVIDDKFTETGLEILDLNNTEIVEDSE

PVEDMITGTACEYETEVVLDSEDEEMNNTGKLTVGERFLQAKSSPAVNNSSVLFKKRLPK

LPCEQDNSKSNATTCGKTGTGDKGASNDAESFDDNNHQYPPAMSYDAHSPTVAALGFVDQ

YLSSIDVDLFQGFQNGKIAMEKSPQVSSARGSISLAKKVKAQTQNEDKDPFKWADKDQNL

KEAGIFCKKLEASFNFGSHGLTYKRRKQQKGSRLQNQGKCSASNGCDENQGNYSDENLVQ

EPTMAIDNNNSLKELYVESRAARDDVDIYSSVAGTEDMSDIGLDTQIAAEAMGALANLSP

VGFHFSDAHQPKNMFDASLSDLKQAHKENSSFKENHGSHSIALKSNKRNVSSCRFSKVTS

KSSSEQTYNQDSNLVSGKMKKIMGSKSTIEGQFKNNTSSSVCSESVSHKEVCLLEEDISF

QPASKEPKIQNKSRWTRMTHQPSHPIEKNNNVEEDIIRYKRKGKCLVADPVVSDVKTKRL

NLSTTSYGVARKSSLNHQIEFSPQISVTSSFSKIDSWACPKGPRGKRKRANAPRVLGIDD

KENNVYSTRSLEGRNAVRKSRLLPVSAGDAIKFENLHDMRPLLLAHVEILSNKSVVQSSS

EISASVGPSEGIKISNANHTCNEHRKKACEKNLPKSSLLKELIRLGVPKSTSEMMSKDLR

HRRDMTNVRVLFSQHLDDNVLKQQQKILARLNISTASSSMEATHFIADKFTRTKNMLEAM

ALGNLVLTHSWLESCGQANFLIDEKNYILRDMKKEKEIGFSMPVSLARARQKPLLKGKRV

YITPHIKPNKEVVASLVTAVHGQLVDENQIFADKNDNILDDLLILSCEEDFAICRHFLKR

GAAVYSSELVLNGIIIQKLELERHKLFVNKLSRCNWFGKVYRKRLSPLS

>Mguttatus_BCP3

MVRESLVEDSLDERESHESKFMDTGKDANNDCEYAGLNYIQSPEVSESHDKALEFVDRYL

SVSDLGSNKYVESRMTNRMQSPPSLWSKGSQCLARRVNLGCTLNKSTPFDWVENPIEKFE

YASPRMNKGSVYGSEDDKYGYVSVNQDSGKVKMQNEIPVPQSEELPGSILSTDNFDRFVF

KSDGIDKMGSNSGICATHDSSELDKQFDTGLSLQNGENDEEWMLNPDAFDAGLNTQMAAE

AMEALMHASPLMVDACSAHQGSENTILDSPNNVNEKNNSKYLASYDEAFVGWRCKGKRSK

CVKISTVDHTNVSLPAAKQLKNKRRTVSKLLLSQSLSTKKLMTGKVLNGRNRRKVEGAYL

KLSSDSSSEVKTDTAKREENFNLELADRTLSKMNMWNYPKRKRSGHCLPCHSIRSSNQCS

PCTAIENIVEKHPVVNEEANNRVKELLVYKRRRKFSVEREQIGSALKLAGHLYSSGKDNA

VSDGLQNSVEFHTSVAPGKMKSDSITLQVLENTDKSKQACNPISKSPLMKELMRLGYTES

LPDFPSKDSRRRRATEKVSILFSQNLNTSILKKQKKIVARLGFSTASCCSDATHFVTDRF

MRTKNMLEAIALGKPVVTHLWLESCEQAGCLIDEKSYILRDEKKEKEIGFSMTVSLSLAS

QHPLLKGREVLITPNVKPGMDVVKGLVEAVHGQALENIRNTKTKVIADDLLILSCKEDYT

TCLPFLEKGASIYDSELLLNGIITHKLEYERYKLFKDFMPPRRNGKMACAA

>Slycopersicum_BCP3

MEAKNAYTLPFYDTFNIESPSNGVDNLDFPTMILDVPDCVENEVSHIANECEEEVVLDSD

DERMHGTEVASVSKLRSSDDTRYKQQLGEWKLSPVPKQTYVGCLRRSKNLFKFLDSARST

SDFEKTDQQKQCSEINGRLSLQIDKSSVNAKPRVEEDWCQAGELTNEEIYKGNLETEGST

VYGVNGDKDFPLHSCKIELPKLNKTESQVSGELSEANALDFVDHYLSVYNEDALNEVKDR

GVNKIVSPPFFSRVGSQKLASRMNVQNAVKNSGVFDWPERQSDSANGSFSRHRKILTYHR

KNYGLKKQKVSCIQSSKEPMESRTDLNKAEPKFLPEAHMNVESNFLRKSDEQFDMGTFEQ

QVDYDGNRSDGLDTYDVGLDTQLAAEAMETLLHAPPLKSDLLLAPPIPKTSLVKERKYPE

IAISREFNIGDSSPEPTLCSSTEVELSSVVHRTRHQASLNLRRLENPATNSIRESNFPKK

RRKQHELEELNDNLFKVATVRGKVSKSSTNTSRKSRKVSMNNRGEICLSVAATLSQVKLE

NWVSKGKRTHKGVRRISNGSSNLYPLLMPADQGNDFKFPVLNHKAERKCQPVEFKSQNQS

LQKTQSGLLSIQTASTNVLTKEMKSDVLLNGISDPSYCLNDHKKGKQHMRSLSRSPLSQE

LIRLGYAEQLPDFLPRGSRRRKGAGDICVLFSQGLDSKLIKQQKKILARLGFISTSNCSD

ATHFVTDSFVRTKNMLEAIACGKPIVTHLWLESCGRASCFVDEKSYILRDAKKEKELGFS

MPVSLAHARKHPLLEGQRVIITPNAKPNRDTLLTLVKAVRGEVVDECNSKITSDDLLILS

CEEDYKACIPYLEKGTLVYSSELLLNGIVIQKLEYNRHQLFTKFHDENCKE

>Pamilis_BCP3

MAAPSEVPFYGDKLGGMIEVYAESLDTVTKVNGLSGESQVGDALHDTEVLHDIDSEGNLE

PQMLEGYDTEVVPDSDDEVTDDIEGKEFSCATGSFVDGLAPPCSPCKQQRHPSVACADSG

HVSGQSTLMTICKGDDNTPFTKQREIQDTEVLFEESRASLVKDVDLVNSISCAVFTGDCH

VRTREIHLVYARKKRRTSLEEKREPNAAECECAGLSYVDSQEPVDISKALLFVDNYVSLN

AINYSPVAKGNVNVKASSPPMSSVKGVQRLVRSGKLRDKAAEVGNFEWDQEEDRVGKSES

DRAWLGSKHGKVFVRGSRKKTVNFSENHTRKDQDEKTKNLSENHTLKHQDEKTENLSENH

TLKEPDHNFEIGIDTQIAAEAIQELSFAHFCSASGSIVSQPADRKDGEKLTGKCLDEGSF

EDSGKAEFMKKASVKRAKRVNANGCKDDLSSFVDHESKSFTLPHDTWFLSDYSKGKRTCN

RELCHSEGSTKRCNLLSLRSRCNMQTLLDSSSFGSYVKRKRRSSVYISPCSYPMKENGPV

LVGKKQSICEQVIKGGRIEMASNKDFNHSPLTDETNLTRISGDGFDEQAGKKKATPSPLT

RELVRLGINESSPELTSRYSRRRKMMSHIRVLLSNNLDKGTLKQQKKILARLGVPMVSCP

SAATHFIADKFARTRNMLEFIARGKPVVTHTWLESCEQAGSYIDERSYILRDVKKEQEIG

FKLTLSLDKARQHPLLKGYKFFITQNTRPGPEMLTSLIKAAHGQAVDVIMDCKAGCLNDL

IILSCEEDADYCVPFLKRGAKAYNPELILNGIVIQKLEFERHRLFKSHGRRL

**BRASSICACEAE**

>Athaliana_BCP3

METEDFASDLQSPSASSDHRFEVNRILGCGEIEDTEAIDEALVLNSSLLETPFQVLYDDT

ELVDNGNCGDEESLNQECYGLDQVVVDDSDDEDGSVAAGEPMCLPELSPKAGDILLESDG

SNDHECQTGKQESNCDTVTGFQGSLRITASSHGPGLDFLDSQEPGESTQADALGFVDHFL

MDKDLNLSPVDLPVNSCRRKSSPVSGAKGCQSLAKRIMSRSPTRKMSVFDWSSDQCDVSD

PKTSPVTRASITCSKIREDQVAGDDFGVKKRSRDPCDIRKVSSHPTQRVMKNSSAKHLKM

EQASGLSEGIMFISQKDAQLEDKAPKEPLEPEEDFIDIGINTQIAAEAMSALVYAPCTTE

EACESDPIPGSVSEMRDQVSNLSRRNNDTIEGEPERDKLNGLFSAAPHRERNSKKKRKFT

NEERTRANVSVKTCLLNLCEWRHPRAKRSRLMQRHHVPPRRSWGASLVKDRSETNTLSSS

LRGSLSGTRQASSCQSGVIDLNVAYHASPRKIYDGSHESPCNKDFPRLFLQKELTTSLGG

PGKIGDFVWKDLRKRRNLAHVRVLFSQNLDDETVKQQKKIMVRLGISPASSSADSTHFIA

DRFARTRNMLEAIALGKFVVTPIWLESCAQTRCLIDEKSYILRDIKKEKDGFCLLTSLAR

AKQHPLLKGFKVCITPSIKPSRGMITDLVKMTQGQVVEASEIIAAEDRNFPEDVLILSCK

EDRDFCLPFVNQGAVIFTSELLLNGIVIQKLEYARFATHYHQNQRL

>Itinctoria_BCP3

MLNHSDAEGSASDSKSPSASSDYRFEVNGISGDTEFQDTEPIDDDSSLLEAPFHKLYDDT

ELVDNGDCGDGETLDHEFGGLEEVVDDSEDDEGGKGSVASAKPTSFPERSVKPADMLLES

DGSNDHECRHTGKQASNCDSHGPALDYLDSQEPGESTQAEALGFVDQFLLDKDLNLSPVN

LLETSRRRKSPPFSGAKGRQSLAKIIKTMSPTNKMRVFDWDSDQCDVSGPQNSPPVIGAN

IKCFKNSEDLLKDDDPGVKKGSMDSCENRKVSTHPTQRFMENSSAKHHNMEQTSNLCQGI

MLISQMDAQLQDKGSKQQPEPEEDFIDVGMLKFTMKEPTGTNASARTCLLNLCEWRHPRA

KRSRLSQKHHVPPRKSWGASSAKDRSETKTLSSRLRVSLNGTRQASSCQSGVGVLDVANH

ASPRKIYGRSHESTCNKDLPRPFLPKEITRLGGSGKVGDLMWKDLRRRRNLAHVRVLFSH

NLDDETIKQQKKIMGRLGISPASSSADSTHFIAERFSRTRNMLEAIALGKPVVTPLWLES

CGQTRCLIDEKKYILKDSKKEKVGFCMLTSLARAKQHPLLKGLQVCITPNIKPDRGMIAH

LVKLTQGQVVDINDIISAADREIPDDLLILSCEDDRDLCLPFINQGAEIYTSELLLNGIV

IQKLEYARHCLF

>Evesicaria_BCP3

MENHSDAEAFASDSKSPSSSSDHRFEVNENHDTEPIDDNDDDISLHEIPFQNLYNDTELV

DNEEALDLEFGGFDQLVDDSKDEGGGKVFVLERSLKVSDTLLESDGSNDQECQIGKQESM

CDSHGQGLDYLDSQEPGDATQAEALGFVDQFLMDKDLDFSPVSLPEASVRRKSPPFSGAK

GRQSLAKRIKTMSPVKKMSVFDWDCEDQCDVSGPQNSSVIVAKKREDPVEDDDPCENRKV

STHPTKRFRQGNFAEHNKMERASGLSQGIMLISQIDAQLQDKASKEQEHSEPEEDFIDVG

INTQIAAEAMSALLFAPCPEVEAGESEIRDQVSNISGRNTMEGRPERDNWSDLLSAPNKK

RNSKKKRKFTMKERIGTNASAITCLLNLCEWRHPRAKRSRLIQTHHVRPRKSWGASSAED

RSETNTLSGKLVVSLSGTRQTSSCQSGVNLDVANHASPMKIYGRSHISSCDKDLPRPFLR

KEIKRLGGSEKVGDFMWKDLRRRRNLAHVRVLFSNNLDDETIKQQIKIMVRLGISPAASS

AESTHFIAERFSRTRNMLEAIALGKPVVTPLWLESCGQTRCLLDEKNYILRDSKTEKDGF

CLLTSLARAKQRPLLKGLKVCITPNIKPDRGMIVQLVKLTQGKVVEISEIMAREIPDDLL

ILSCEEDRDLCLPFIKQGADIYTSELLLNGIVIQKLDYARHCLF

>Esalsugineum_BCP3

MSQIDDCSSLETPFQKLYDDTELVDNGDCGDKETLDQEFGGLDEVVDDSEDEDRGNGSVA

AAESTCLRELSPKAADILLESDGSNDHECETGKQESNCGIATGFQGSSRITADSQGPGLD

FLDSQEPGESTQAAALGFVDQFLMDKDLNLSPVNLPETSRRRKSPPFSGAKGRQSLAKRI

KTRSPTRKMSVFDWDSDQCDVSDLQNSPVTRANVTCFKRREDHVQDDDPGVKEGSMDLCE

NRKVSKGPTKRFMQNSFAKHHKMEQTPSLSQGIMPISKMDAQLQDKASNEQFEPENEFTD

VGISTQIAAEAMSVLLFAPCTKAEAHESETMDLSRKNNDTIGSRPERDNWSDLLSAPNKK

RNIKKKKRKLTMKQRAGTNVSAITCLLSLCEWRHPRAKRSRLTQRRHVPPRKSWDTSSAK

DRSETNTLSRRLRVSLSGTRQASSCHSGVNDLDVANHASPMKIYGGSHEISCNKDLPRPF

LLKEIARLGGSGKVDDFMWKDLRRRRNLAHVRVLFSHNLDEETIKQQKKIMVRLGITPAS

CSADSTHFVAERFSRTRNMLEAIALGKPVVTPLWLESCGLTRCLMDEKKYILRDSKKEKE

GFCMLTSLARAKQHPLLKGLKVCITPNIKPDRGMTADLVKLTQGQVVEISEIIAAEDRKI

PDDLLILSCEDDRDLCLPFINQGAEIYTSELLLNGIVVQKLEYARHCLF

>Cgrandiflora_BCP3

MEIEVFASDSQSPSASSDHRFEVNGVLGCGEIEDTEPIDEEIVLDSSLLESPFQNLYDDT

ELVDNENYVDEETLGLDQVVIDSEDEDGGSGKAAAGELTYLRERSPKAAGVLLESDGSND

HECQIGKQESNCDIVTGFQGSSRINDESHGPGLDFLDSQEPGEATQADALGFVDHLLKDK

DLNLSPVDLRENCSRRKSPPVSVAKGYQYLAKRIKSRSPTRKMSVFDWSSDQCDVSDPQN

SLVSRANITCFKKREDHATEDDPGVTKGFMDLCADRNVSTHPTQRFMQNSSVEQASGLNQ

GILFISQEDAQLQDKASKEHSEPEEDFIDIGINTQIAAEAMSALVYSPCIKEEARESDPI

PRRISEMRDQGSNIPRGNNDTIEGGPERDNGNGPLSGLHKERHSKRKRKFNKEERTGTNV

CVMTCLLNLCEWRHPRGKRSCLMQRHHVPPRRSWGASSIKHRSETNTLSSRSQVSLSGTR

QASSCQSGVIDVNVANHAAPKNIYGGSHESPCKKDFPKLFLQKELTTRLGGPGKVGDFVW

KDLRRRRNLAYVRVLFSQHLDDETIKQQKKIMVRLGISLASSSADSTHFIADRFSRTRNM

LEAISLGKPVVTPLWLESCGQTRCLIDEKNYILRDSKKEKDGFCMLTSLARAKQHPLLKG

FKVCITPNIKPSRGMIADLVMLTQGQVVEISEIVSVEDRNFPEDLLILSCEDDRDICVPF

INQGAVVYTSELLLNGIVIQKLEYARFGTQK

>BrapaFPsc_BCP3

MVNDSDAEACASDSBCP3SPSASSDHGLEVNGIHDTEPIDDDDNDGISLVEDLYNDTELVDDGETLDQVVDDSEDEEGGGKGSVASSKQPCLLERSVRAVDMLLESDGSNDQECHTGKQESNCDVVTGFQGSSRIAADDSHGQGLDYLDSQEPGDATQAEALGFVDQLLMDKDLNLSPPVNLQETSLRRKSPPFSGAKGRQSLAKRIKTMSPTKKMSVFDWDCEDQCDVSGPRNSPANGGNVTCFKKREAAAPVKDDDPCENREVSTHPTRRYMQKDSAKHNKMDAQLQEKASKEHSEPEEDFVDVGINTQIAAEAMSALLFAPCTIEEASESETRDQACNLSGRNNDTIEGSAPNKKRNSKKKRKFTMKERTGTNASATTCLLNLCEWRHPRAKRSRLTPRHHVPPRKSWGASLAKDRSETNTLSGRLVVSLSGRRQASSCQSGVDLDVLNHASPKKIYGRSHESSPDKDLPRPFLPKEIKRLGGSGKVGDFKWKDLRRRRNLAHVRVLFSHNLDDETIKQQQKIMGRLGISQASSSAESTHFIAERFCRTRNMLEAIALGKPVVTSLWLESCGQTRCLLDEKNYILRDSKTEKDGFSLRTSLARAKQHPLLKGLKVCITPNIKPDRGMIAHLVKLTQGQVVEISEIIAAADREFPDDLLIISCEDDRDLCLPFINQGAEIYTSELLLNGIVIQKLEHAR

>Bstricta_BCP3

MVNLMETEVFASDSQSPSASSDHFNGILGCGEIEDTEPIDEAIVLDSSLLETPFQKLYDD

TELVENGNCGDEETLGQECYGLDEVVGDSEDEDGGSGNVAAGELTCLQERSPNAADILFE

SDGSNDHKCQIGKQESNCDTVTGFQGSSRITADSHGPGLDFLDSQEPGEATQADALAFVD

HFLMDKDLNLSPVNLPENSWRRKSPPVSGAKGCQSLAKIIKSRSPTRKMSVFDWGSDQCD

VSDPKNSPVTRANITCFKRREDHVADDDPGVKKGFMDLCANRKVSTHPTQRFMQNSFAKH

HTMEQASGLNQGIMFVSQKDAQLQDKASKEHSEPEEDFIDIGINTQIAAEAMSALVYAPC

TKEEACESDPIPGRISEMRDQVSNLSRRNNDTIEGGPERDNWSGPLSAPHKEINSKKKRK

FTKEERTGTNVFVMTCLLNLCEWRHPRGKRSCLMQRHHVPPRRSWGASSVKDRSETNTLS

SRLRVSLSGTRQASSCQSGVIDVDVANHASPKKIYDGSHESPCKKDFPRLFLQKELTTRL

GGPGKVGDFVWKDLRRRRNLASVRVLFSQNLDDETIKQQKKIMVRLGISPASSSANSTHF

IADRFSRTRNMLEAIALGKPVVTPLWLESCGQTRCLIDEKSYILRDSKKEKDGFCMLTSL

ARAKQHPLLKGLKVCITPNIKPSSGMIADLVNLTQGQVVEISEIIAAKDRNFPADLLILS

CEDDRDFCLPFINQGAVVYTSELLLNGIVIQKLEYARHCLF

>Mperfoliatum_BCP3

MVNHCDAEVSASDLKSPSASSDHRFGVNGISGYGEFQDTEPIDDDSSLLESPFHKLYDDT

ELVDYGETLDHEFGGLEKVVDDSEDEEGGKESVASAKPASFPERSVKAADILLESDGSND

HELGKQEPNCDRHGPALDYLDSQEPGESTQAEALGFVDQFLLDKDLNLSPVNLLETSRRR

KSPPFSGAKGRQSLAKIITTVSPTKKMRVFDWDGDQCDVSGPQNSPPVIGANITCFKKTE

DFLKNNDPRVKKGFTDPCENIKVSTHPTQRFMENSSAKHHNMEQTSNLCQGIMLISQMDA

QLQDKASKQQPEPEEDFIDVGINTQIAAEAMSALLYAPCTNEEARVSEMRDQVSNLSRRN

SDTIEGRPERDNWNDLLSAPNKKGNSKKKRKFTMKEPVGTNASARTCLLNLCEWKHPRAK

RSRLSQKHHVPPKKSWGASSAEDRIETKTLSTRLRVSLNGTRQASSCQSGVSDLDVANHA

SPRKIYGRSRGSTCNNKLPRPFLPKEITRLGGSGKVGDLMWKDLRRRRNLAHVRVLFSHN

LDDETIKQQKKIMVRLGISPASSSADSTHFIAERFSRTRNMLETMALGKPVVTPLWLESC

GQTRCLIDEKNYILKDSKRQKDGLCMFTSLARAKQHPLLKGLKVCITPNIKPDRGMIAHL

VKLTQGQVVEISEIITAADREIPDDLLILSCEDDRHLCLPFINQGAEIYTSELLLNGIVI

QKLEYARHCLF

>Rislandica_BCP3

MANQIDTEVFASDSESPSASSYHRFEVNRILGCGEIQDTEPIDEAIVLDSSLLQNLYEDT

ELVDNGNCGDEETLDLEYEGLNEVVDDSEDEGGGTGNVAAAESICLREPSPKAVDVLLES

DGSNDHECQTEKQESNCDMVTGLEGSPRITATSDGPGLDFLDSQEPGESTQEAALGFVDN

FLMDKDLNLSPVDLPKNSWRMKSPPVSGAKGCQFLAKRIKTRSPTRKTSVFDWGSDQCDV

RDPQNSPVTRTSIKCYKRRKDHVADDDPGVKKGFMDLCENRKVASHPTKFFMQNSDPKHD

KTEEGSGFSHGNMFISQMDAQLQDKASLEHLEPEEDFIDIGINTQIAAEAMSDLLYASCT

KEVACESDPHPGSISEMRDQVSNLSRRNNDGTIENGPERDNWSGPHSAPHKERNSKKKRK

FTMEERTGTNVSAITCLLSLCEWTHPRAKRSRLMQRHHVPPKRSWGASFKDRSGTNNLSS

RLRVSLSGTRQASSCQSGVNDSDVAKHASPRKIYRGTPESPCNKDLPRPFLVKELTRLGE

PGKVGDFMWKDLRRRRNLAHVRVLFSQNLDDETIKQQKKIMVRLGISPASSSADSTHFIA

DRFSRTRNMLEAIAQGKPIVTPLWLESCGQTRCLMDEKSYILRDSKKEKEGFCLLTSLAH

AKQHPLLKGLKVCITPNIKPNRGMIADLVKMTQGQVVEISEIIAADDKKFTDDLLIISCE

EDRDFCLPFINQGAVIYTSELLLNGIVIQKLEYARFATTDHQIQKL

>Crubella_BCP3

MVDQMEIEVFASDSQSPSASSDHRFEVNGVLGCGEIEDTEPIDEEIVLDSSLLESPFQNL

YEDTELVDNENYVDEETLGLDQVVVDSEDEDGGSGKAAAGELTYLRERSPKAAGVLLESD

GSNDHECQIGKQESNCDTVTGFQGSSRINDESHGPGLDFLDSQEPGEATQADALGFVDHL

LMDKDLNLSPVDLRENCSRRKSPPVSVAKGYQSLSKRIKSRSPTRKMSVFDWSSDQCDVS

DPQNALVSRANITCFKKREDHAAEDDPGVTKGFMDLCVDINVSTHPTQRFMQNSSVEQAS

GLNQGILFISQEDAQLQDKASKEHSEPEEDFIDIGINTQIAAEAMSALVYSPCTKEEARE

SDPIPRRISEMRDQGSNIPRGNNDTIEGGPERDNGNGPLSGLHKERHSKRKRKFNKEERT

GTNVSVMTCLLNLCEWRHPRGKRSRLMQRHHVPPRRSWGASSIKHRSEANTLSSRSQVSL

SGTRQASSCQSGVIDVNVANHAAPKNIYGGSHESSCKKDFPKLFLQKELTTRLGGPGKVG

DFVWKDLRRRRNLAYVRVLFSQHLDDETIKQQKKIMVRLGISLASSSADSTHFIADRFSR

TRNMLEAISLGKPVVTPLWLESCGQTRCLIDEKNYILRDSKKEKDGFCMLTSLARAKQHP

LLKGFKVCITPNIKPSRGMIADLVMLTQGQVVEISEIVSAKDRNFPEDLLILSCEDDRDI

CVPFINQGAVVYNSELLLNGIVIQKLEYARHCLF

>Lannua_BCP3

MVNQSDVEVFASDSKSPSASSEHRFEVIGISRCGEIYDTEQIDEAIALDSSSLEFPFQKL

YDDTELVDNGNFGDEERLGQELDGLDEVVDDSEDEDGGMGSYAAVDSTCFRERISKAADI

LLESDGSNDQECQRGKQESNCDTVTCFQGSSRNSQGPVLDFLDSQEPGESTQAAALGFVD

HFLMDKDLNLSPMDLPETSWRMKSPPFSGAKGRQSLANRIKTTNPTRKMRVFDWESDQCN

VSDPQNSPVTRANITCFKRRENHVADDDPGVKKGIMDLCENRKVTSHPTQRFMQNSSAKH

HETEQGSGFNQGIMFISQMDGQLQDKASKEQLEPEEDFIDIGINTQIAAEAMSALMYAPC

TKEETSESGPCLHSIPGRISEMRDQVSNLSRRNNDTIKGRPERDKWSGPISVPHKERNSK

KKRKFSMEKRTVTNVYAITCLLNLCEWRHPRAKRSRLTRRHHVPPRISWGASSVTYSSEA

NTLSNRLRVSLRETRQASSCQSGVNDSDVVNDTSARKIYGRSHENPCNKDLPRPFLLKEL

TRLGGPGKVGDFALKDLRRRRNLAHVRVLFSQNLDGETIKQQKKIMVQLGISPASSSADS

THFIADRFARTRHMLEAIALGKPVVTPLWLESCGQTRCLIDEKSYILRDSKKEKNGFCLL

ASLARAKQHPLLKGLKVCVTPNIKPSRGIIADLVEVAQGQVVEMSDIISAEDGNIPEDLL

ILSCEDDRHICLPFINQGAVIYTSELLLNGIMIQKLEYARHGLF

>Mmaritima_BCP3

MVNHMDTEVFVCDSQSPSASSDHRFEVSGVLGCGEIEDTEPIDEATELDSSLLEIPAHKL

YYDTELVDDGGCGDEETLDQECYGLDEVVDDSEDEDGRKGSVAADEPTCLRERSPKAADI

LLESDGSNDHECQIGKQESNCDIVTGFQGSSRITANSHGPGLDFLDSQEPGESTQADALG

FVDHFLMDKDLNLSPLNLPENNRRMKSPPVSGAKGCQSLAKRIKSRSPTKKMSVFDWGSD

QCDVSDPQYSPVTRASITCSKRREDHVADDDPGVKKGFMDPCENRKLSGHPTQRFVQNSS

AKDHKMEQASGLSREVMFRSQMNAQLQDKVLKEQSEPEEDFIDIGINTQIAAEAMSTLLY

APCSKEEACESDPIPGRISEMRDQVSNRSNMNNDTIADGPERDNWSRPLSALQKKRNSKK

KRNFTKIEGTRANVSPITCLLNLCEWRHPRAKRSRLMQRHHVSSRKPWGASSVIEANTLS

SGLQVALSGTRQASSCQSVVRQASLRKIYGGAHESPCNKDLQRPFLLKELTRLGGPGKVG

DFMCKDLRRRRSLAHVRVLFSQSLDDETIKQQKKIMVRLGISPASSSADSTHFIADRFAR

TRNMLEAIALGKFVVTPLWLESCGLTRCLMDEKSYILRDSKKEKKEGFCMLTSLARAKHH

PLLKGLKVCITPNIKPSRGMIADLVKMTQGQLVEISDINAAEDRNFPQDLLILSCEEDRE

FCLPFINQGNVAYTSELLLNGIVIQKLDFARHCLF

>Tarvense_BCP3

MANHIDAEVFASDSKSPSASSEHRFEVNGVLDFGEFQDTEPIDDSSLLETPFQKLYDDTE

LVDNGDCGDSEETLDQECGGLDEVVDDSEDEDGGHGSVAASDSTCLKEGSLKAADILLES

DGSNDHECQRGKHESNCDIVTGFQASSLITADRQGPRLDYLDSQEPGESTQADALGFVDN

FLMDKDLNLSPANLPETSRRRKSPPFSGAKGRQSLAKRIKTSSPTKKMRVFDWDSDQCDV

SCPQNSPVIRANITCYKKSEDHVNDDDPGVKKGSMDPSENRSFPTQPTERVMQNSSAKHH

KVEQTSGLSQSIMLMSQKDAQLEDKASTEQLEPEEDFIDVGISTQIAAEAMSALLYAPCT

REEAHESEMRDQDSNLYRKKKDTISDDRPEKDNRSDLRSAPSKKRNSEKKDKFTRKERTA

TNVSAITCLLNYCEWRHPRAKRSRLTQRHHVPPRKPWGASSAKDRSKTNTRSSRLRVSLS

GTRQASSCQSGVLDVANHASPREIYGGTHERSCKKDLARPFLLKEITRLGGSGKVDNFMW

KDLRRRRNMAHVRVLFSHNLDDETIKQQTKIMVRLGITPASSSADSTHFIAERFSRTRNM

LEAIALGKPVVTPLWLESCGQTRCLIDEKNYILRDIKKEKDGFCMLTSLSRAKQHPLLKG

LKVYMTPNIKPDRRMLADLVKLTQGQVVEISEIIAAEDSKIPDDVLILSCEDDRDFCLPF

INQGAEIYTSELLLNGIVVQKLDYARFGTAFYTTVH

>Salba_BCP3

MVNHSDAEVYASDSTSPSASSDHRFEVNEIHDTEPIDDDDDISLLEIPFQNLYNDTEPVD

NCETLDLEFGGVEEEVVDDSEDEEGGGKGSVASSKQTCLLERIVKGADTLLESDGSNDHE

CQTGKQESNCDIVTCFQGSSRITAGDSHGQGLDYLDSQEPGDATQAEALGFVDQFLMDKD

LDFSPVSLPDASLRRKSPPFSGAKGRQSLAKRIKTMSPTKKMSVFDWDCEDQCDVSGPQN

SSVIGANVTCFKKREDPVKDDDPCENRKVSTRPSKRCMPNDSAKQNKMEQASGLSQGTML

ISQMDAQLQDKASKEHSEPEEDFIDIGINTQIAAEAMSALLFAPCTIEEASDSEMRDQVS

NLSGRNYDTIEGSAPNKKGNSKKKRKFDMKEKIDTNASAITLLLNSCEWRHPRAKRSRLT

PRHHAPPRKAWGAASDKDRSETNTLSSRLRVPLSGTRQASSCQSGVDLDVANRASPRKTY

GRGHESSRDKDLPRPFLPKEIKRLGGSGKVGDITWKDLRRRRNLAHVRVLFSNNLDDETI

KQQKKIMGRLGISPAASSAESTHFIAERFSRTRNMLEAIALGKPVVTPLWLESCGQTKCL

LDEKNYILKDSKKEKEGFSMGTSLARAKQHPLLKGLKVCITPNIRPDRGMIAHLVKLTQG

QVVEISEIIAAADREIPDDLLILSCEDDRKLCLPFIDQGAEIYTAELLLNGIVIQKLDYA

RHGLF

>Cmaritima_BCP3

MVTQSDIEAYASDSKSPSASSDHRLEVNEIHDTEPIDDDISLLETPFRNLYEDTELVDDG

ETLDHELGGFDQVVDDSEDEEGGKGSVASSKQTCLVERNVKGADILLESDGSNDQECHRG

KQESNCDIVTGFQGSSRITADDSHGQGLDYLDSQEPGEATQAEALGFVDQFLIDKGLDLS

PVSLPETSLRRKSPPFSGAKGRQSLAKRIKTMSPIKKMSVFDWDCEDQCDLSGPQNSAVI

GANVTCFEKREDPVKDDDDPRENRKVSTHPTERCMQNDSSKHNKMKQAPGLNQGIMLISQ

MDAQLQDKSSKEQSEPEDDFHDVGISTQIAAEAMSALLFAPCTIEEARESEIRDQVCDLS

GRNNDSIEGSAPNKRRNSKKKRIFTMKERTDTNASAITCLLNLCEWRHPRAKRTRLTQRH

HVPPRKSWGASSAKDRSDTNTLSSRLRVSLSGTRHASSCQSGVNMDVANHASPRKIYGRR

HESSCDKVLPRPFLPKEIKRLGGSVEVGDFVWKDLRRRRNLAHVRVLFSHNLDDETIKQQ

QKIMGRLGISQASSSAESTHFIAERFCRTRNMLEAIALGKPVVTPLWLESCGQTRCLLDE

KNYILKDSKMEKDGFCLLTSLSRAKKRPLLKGLKVCITPNIKPDRGMIAHLVKLTQGQVV

EISEIIAAADREIPDDLLILSCEDDKGLCLPFINQGAEIYTAELLLNGIVIQKLEYARHC

LF

>Iamara_BCP3

MDEFLLGFEVIGISRCREFQDTEPIDVALRLDRSLLEMPLQKLYEDTELVDCGDDEIMNQ

EFDEVVNDSEDEYGSEVPVQTEAANILLESDGSNDNECQRGLDILDSQEPGESTQANALG

FVDRFLMDKDLNLSPVGLPETSWRMKSPPFSGAKGRQSLAKRIKSSSPTKNMRVFDWDSD

QFDVSDPQNLTVTSENITCSKRREDRDSDDDPGMKKGSIDSCENIEHPTQRSMQNSSEKH

QKIEQASGLSQGVMFISHMNSQLDDKALKEQSEPEEDFIDVGINTQIAAEAMSALLYAPC

SKEGVRESKVREQVSTLSIRNNDTIEGRPEKGNWNCLHTDPNKKRNYKKKRTFTMKERAG

QNVSVTPCLLKLCEWRHPRAKRSRLTQVHNISPRKHWDASSTKDRSEANTLSGSLQVSVN

GTRQASSCQSVVNGFDVANHASSRKMYSRSHESSFSKDLYRPFLLKENTRLEGSGKVGNF

MWKDLRIRRNLKHVRVLFSQNLDDETIKHQKKIMIRLGISLASSSTDSTHFIADRFSRTK

NMLEAIALGKPIVTPLWLESCRQTRCLIDEKSYILRDSKKEKDGFCLLTSLSRAKNHPLL

KGFKVWITPNIKPNRDMIADLVKLSQGQVVEISEIIGIEKRKFPEDLLILSCEEDRDFCL

PFTNQGAVIYTSELLLNGIVIQKLEYARHRLF

>Chispanica_BCP3

MVNHSDAEVYASDSKSPSGSSDHRFEVNEIYETEPIDDDDDDDSSLLQKLYDDTELVDDE

ETLDHEIGGVDQVVDDSEDEEGGGKGSVDSSKPRCLLERSVKAADTLLESDGSNDQECQT

GKQESNCDTFTGFQGSSRITIDDSHGPGLDYLDSQEPGDATQAEALGFVDQFLMDKDLDI

SPVSLPETSLRRKSPPFSGAKGRQSLAKRIKTMSPTKKMSVFDWDCEDQYDVSGPQNSPV

IGVKITCLEKREDLVEDDDPCENRKVSTHPTRRFMQENNAKHNKMEQASGSSQGIVLISQ

MDAHLQDKASKEHFEPEEDFVDVGINTQIAAEAMSALLFAPCTIEEASESEMRDQVSNLS

GRNNDTIKGSVPNKKRKFTMKERTSTNASAIECLLNSCEWRHPRAKRSRLTQRHHVPPKQ

SWGASSAKDISETNTLSGRLVVLLSGRREASSCQSAADLDVAIHASPRKIYGRRHGSSGD

KDLPSKFLPKEIKRLGGSGKVGDFMWKDLRRRRNLAHVRVLFSHNLDDETIKQQKKIMSR

LGISQASSSAESTHFIAERFCRTRNMLEAIALGKPVVTPLWLESCGQTRCLLDEKKYILR

DSKTEKDGFCLLTSLARARHHPLLKGVKVCITPNIKPDRGMIVHLVKLTQGQVVEISEII

AAADREIPDDLLILSCEDDRDLCLPFINQGAEIYTAELLLNGIVIQKLEYARHGLF
