# Supplementary material for: Identification of plants’ functional counterpart of the metazoan mediator of DNA Damage checkpoint 1
Source: EMBO Rep. 2024 Mar 4;25(4):19. doi: 10.1038/s44319-024-00107-8 (PMC11014961; doi:10.1038/s44319-024-00107-8)
Supplement: Supplementary file 5 — Source Data Fig. 5 [file 44319_2024_107_MOESM5_ESM.zip › Figure 5/5C/EMBOR-2024-58742V1_SourceDataForFigure5C.docx]

Source data for Figure 5C

**METAZOAN MDC1 SEQUENCES**

>Rhipicephalus sanguineus

MDLNDLEMTQVINGPDDSEDEIDREIRAVLKLHDQDAPAAFQTFSLKPGPNVVGRSRTCDVIIENYAVSK

QHAVIDVGGHSCTIVDLGSQNKVKIGKRTLKPNCQYNLDYGEEFTIAGLRARVLCDQNNDQEGVDIKALA

GCDSNEKQDLPPAASELDVAAVTAEMSSVNALENQAAEKSQDAKAHSSEHQSSGSFNLPEMPNLDYTQTD

SSPGTEPYVGAGHEPQRSQEGAETVVPAAEVPASGNAPTAKDAHPCTSKQSYSNDAGDGNARDTCKLAAA

DEESCLSAETQAYTECPEVPFLSSHGEGVDAAADEMERLNAPTQAYEDDASAEAERLNAPTQAYTEKEDV

ERLNAVTQPYAADVGMDEEERLNAVTQPYAADNCDDEEKLNAVTQPYTVSVNEEEEERLNAVTQPYAAAN

VSQSEHDRMNDVTHSFNDEEDDMVGDSVCAPTQPEERHRRLLTGPSELSFVLCEASQPCREDYGEGDDEE

FCAPTQKDESPPLKVHALLKHRRERGPVAEDDDKTPPLSPKTTVDETPPPSPGFVPESDPEDDGDASMVT

ALHSPSLLNVTGTTVYEDCVTPGSGITSPVLGKVSARRQRRGMSLKKPTCDTVLELPSQESAAGAEWMQQ

QQQQEDSSSQSKASRKLTYVAEAGEDNDLDLRAADAPLAEPLVSEVERTDGIVTTTCSSPSIPVKPQESV

NANESMSAEYSEMPLLHMSEDIDLDGAATDKEDSTACDTTAVQEKGPAEACTSSEIPASTGRSSSDGTVP

KEACEEEQEETLEKDEPANAPSKNESSTASLRKGSEEPSAEPETVPEKTCEEEQALKNDEPANVSSESEE

EGSRVSKRRGKKKPPARTARRTRKAASTNEAKKDPVENNTVPTRRSSGRQNAGSRMKSLLSLEKRTSASG

AFKESELSQEVNDERGTRDDDGPAGESHGKAGRPRTRRGLKGKAAAKEDEPEVAPPCPSKNLPSARESDV

PSSSDGDENKKPSVKPGEDDVEGEAETSDGTRVDNNEECQPKAGPSGHQEGEDADDALSTASLTPSLGDG

DVSCTNATIDTCFSEPLPTFSEVMAECQPYIGDTEAQDEKNEPAEEQEDTAAEPAAESKATAPAVSEPEP

AVVEPVRASRSKRPRGGVSTTNLKASKVDEELPKTRRKVARGAREEPLAEAEVLAEDPASSVEQPEAPQL

SNGAKRGRLYSSAVGRRAVPSVADVVSKISEDVEESEGETDKDLAEDHTSSVQRSEAKQSAASQQSNRAR

RGRPFHHGAGKNAVPSVSNKIVEDVEQSEGETEPHLAEERLSGVEQPEAAQSAASQQSNRARRGRPFRSA

VGRKAVPNITAAKVTEDLEQSEGDRNTDRLSAQETESIDGTLSEASECSPSSRRKAIPHIEVSKATCRGA

RRLLQLNAKRGGEEAASESDTDHLSAQDTESIHGTPSNAGETLTSEAEPVPSQSLSRASARRGAKNARLS

LRRDTEQADQAASEGPAAENIEEPPQNVRASQRGRTSAKTRSGREEEASDVAAADVSLECVELSQSPRTS

QRDKRGAKISREKDEPFVQSLLDDSTQSLQTEAHSQNSRSSQRGRRNAKKPETQDNLTSEENQDVPPVQS

AVDDSTQSLSTESHSQASRSAQRGRRNAKKPETLDNLTPEENQDAPPVQSAVDDSTQSLDTESHSQTSRS

SQRTRRNVKQIETQDLTPEETEDTHVESVLDDSTQSLSMESHSKTSQGSLLGKRNAKKPKMQNKLTAEGT

GDAPPVQSTLDDSTHSLSKECQSQNSRSSRLGRRNVKQPETMNNATPEGTDVQTDVDAQDAKPSRHGRKA

LKLSQEVGSRTIASAGENTLASAEPGTQRTRRGGDTAETSSQNEQVRLAPEAAEGVEPSENASTEVVPRS

APTRRGRKAAQPTRKRGGSKNVPEDDTVTETFDTPEPTNPRSSKALRTANLSLPKPTEDTEVLVKVEPPS

PRTSQRRSAKTAQVSSEETEQTNNGDSAKVSSRFKRKVTKVNTKDVTAEAETEEALSSESTSRSPTSRMP

APPVPVTRKRDARRAGRKDDPSPPTVEPVLETSEETSLLCPVQRVGRKRHIASMSFGEARHESDDEAVDN

EQETLDEVTVKLGSKSRRKAPAKEAAIKQEVVEKAPARRGKRKADLPKAEAPQEAKARQDEQVDSSPAPK

KTAKVKPKVLFTGIDDTRTEEQVVRDLGGIIATNASACTHLVTDKFRRTVKALCCIGKGTPIVDVAWIKK

CQEAGAFVDHVPHMLLDKKAEKALNFNLRDTLTKASTGGVLRGWSVHATAHVLPSPSDMKEIVACAGGKY

LDNLPARTSTGTTVVISCKQDLKACARVRNHGIPVVAAEFILSGLLQHNLNVDAHRLE

>Chelonia mydas

MTGTHSDQSEQEKAMPEGSGWPIRGLPAGKRREGPGGPQLEQEAMEQTQLLEWGEDEDPPGGEAPGEAPR

PVGRLHLLSSKYGPEQDFWIYPGENVVGRLPSCQVCLPAPSVSKAHAVIEVPVPDGPHLLYDRGSLNRTR

RQRAVLLPHVRYSLEDGDTLMFGDVGCQYFLLPRAGADPEDSLEVPPTQPRVPNTLAIEETPAPSKRMGY

GALLARDSEDEEESQGGGRLLQLPGSNGSNSSSDTGVRSDAAFSSPCATVVPESDEEGGETPDAPCPSLR

LSYGGERPPGPPENGVAALGAEQPRAMGVVGTAREPALAACRLVKGPGHTGIATGMGQGPAVGGLGNGCP

AEVGSVTDVEEVRHPDVVRKSHGPAVLGDSDTDVEEAVENLDVVDPKSHPPAIELGSDTDVEEAENADVH

PKGHQLARNGDSDTDVEGAGVNPDVVGPKSHQPRVEVDSDTDVEAAAAKNPDVQQNNRHTLPGGRETDVE

GEVENLNDTHPESHHSAKDGESDTDVEEVAENPDVQKSHQPAVEVGSDTDVEEEKAETPVVVPNNHQLVS

PGGGDADVARAVGKPDVHPKGHRPAGNEDSDTDVEEFVGNPDVTAKSHLPAIDVGSDTDVEEEETKNPDV

ATKNHQLALPGDRDTDVTGTVGKPDVHPKGHRPAGNEDSDTDVEEFVGNPDVTAKSHLPAIDVGSDTDVE

ESVGNPDVQRSHQPPVEGGNNTDVEEAEGKPDVGRQKDPLPVATGDSDTDVEENPDVGQQTLHLPTASGD

SDTDVEGAPGAEFDQAPGLVGGPAQAGGEELPAPQSHVSPEAKVMDTEGNKEPAWGPAQPGDDSETDVED

DADFALQATQCYLAEETPSSGAEAARAPAAADSGSSLEEEATQTFVFRSPPPAKGSVMSPPAGPALHAPV

CASSEKEEDSDEDLYVVGATQSFCEDPGLHSDQPTQPFTPEEDTQLLLRHPPPGGALSQEPAQPAVPSPA

SGGLLPLGTQAPAAARLGPREESEPPVRALPAGGAGGELPEEEESQPVRLVLSAPRRPALALQGEGGRAG

AEPGGSRSGQRLRRQGGPEAAERWETPAAVGVTGAGAHPPGARPEDAGKVPEVHPSPSTPVRHRSLRSAP

VPAPPPPAPAPERRSRRGGAEKPVGSGDPSQPTPAAPQRRGRRQLCSQTVYMEAPEPGSVEEVDQPGPVK

RPRRAVRTQDPSTEPEASSQGAPGRARRSRGAAVTEPAPANKPRRGSGGPEPEASQGRARRSQRAPAQEP

APRRRGAAEPAKEARGKRRGVATPDPPQARGEGQVGSATAQEDAGSDARKRQEAPVPTLRRQSTENKVEG

LRARAPRRSQGAAGGTTSPRVLFTGVIDEAGERVVSALGGALAQSVFDCTHLVTDRVRRTVKFLCALARG

VPIVTLDWLDKSGRSACFLSPSGFLVRDPEQERNFHFSLAQSLQRARRGALLQGYEIHVTPNVKPEPEHM

KDIIQCSGGVFLPHMPRAYKDKRVVISCPEDLPRCRPALSARLPVASTEFLLTGILQQAVDLASYRLDGT

PAPPSAATPATRGSKRKGAAGPAPAPPRSAKRRR

>Mauremys reevesii

MEQTQLLEWEEDGDPPGGEADRPVGRLHLLSSKYGPEQDFWIYPGENVVGRLPSCRVCLPAPSVSKAHAV

IEVLAPDGPHLLYDRGSLNRTRRQRGVLLPHVRYSLEDGDTLMFGDVGCQYFVLPPGGADPEDSLEVPPT

QPRAPNTLAIEETPAPSKRMGYGPLLARDSEDEEELQSGGRLLHLPGSNGSNSSSDAGVRSDAGFSSPGA

TVVPESDEEGGETPDAPCPSLRLSYGSERTPGPPENGVAALGTEQPRATGVVGMDPEPVLAACRLVKGPG

HTGITSGPGAAVRGLGNGCPTEVGSITDVEEEVRHPDVVQKNHGPALLGDSDTDDDEAAVENLDVVDPKC

HPPAIEMGSDTDVEEEAQNPDVHPKDHQLAGNGDSDTDVEAPLENPDVVQGMKNQHAAPGDSDTDGEGAD

VNPDVDPKSHQPSFEVDSDTDVEEEVAAAKNPDVQKNHRHTLPRGSAADVEGEVENPDDVHPESHHSAKN

GESDTDVEEVAENPDVVQKSHQPAVEVGSDTDVEEEKAETPVVVQNNHQLISPGGGDADVARVVEKPDVR

QTNDELAAPGDSDTDVEESDRNPDVVTAKSNPLAVDVGSDTDVEETGNPDVGTKSHPLAIEVGSDTDVEE

EEANNPDVIMKNHQHALPGDRDPDVTGTVGQPDVHPKGHQPAENEDSNTDVEESGGNPAVVQRSHELTAE

AGDDTDVEEAEEKSDVGQQNDPLPVATGDSDTDVEENPDVGQQTLHWPTGDSDTDVEGAPWAESDQAPGP

VGGPAQAGGEQPPAARSHLSLETKLMDTEGSKEPAWGPAQPGDDSETDVEDDADFALQATQCYLVEETPS

SGAKATRAPTAADSGSGLEEEATQTFVFRSPLPGKGSVMSPPAGPALHAPVCTSSEKEEDSDEDLCVVEA

TQSFCEDPGLHSDQPTQPFIPEEDTQLLPRPPPPGGALSQEPAQPAVPGLASRGLLPLGTQAPTVPRLSP

KEESEPPGGALPAGGAGREPPEEEESQPLRLVLSAPRRPALVLQAEGRRAGAEPRGSSSGRRLRGQGGPE

AAERRETPAAVGVTGTGPHPMGARPEDAGKVPEAHSSPSTPVRRRSLRSAPVPAPPPPTPAPERRSRRGG

AEKAVGSGEPSQPAPAAPQRRGRRQLCSPSVYMEAPEPGSVEVEQPGPVKRPRRAVQAQDPPTEPEASSQ

GAPGRALRSRGAAGAEPAPADEPRMGSGGAEPEASQGRARRSQRAPAQEPAPRRRGAAEPAREAQGKWRG

VATPDPPPAQGEGRAGSAAAQEDAGSDARKRQEAPVPPLRRQSTENKVEGLRARAPRRSQGAAGGTASPR

VLFTGVIDEAGERVVSALGGALARSVFDCTHLVTDRVRRTVKFLCALARGVPIVTLDWLDKSGRSACFLS

PSGFLVRDAEQEQNFHFSLAQSLRRARRGALLQGYEIHVTPSVKPEPEHMKDIIQCSGGVFLPHMPRTYK

DKRIVISCPEDLPRCRPARSARLPIASTEFLLTGILQQALDLPPYRLDGPPAPPPPSAAAPATRASKRKG

ASGPAPALPSSAKRRR

>Gopherus evgoodei

MVPGRHSTRTSASSLCSKRETGPQGAAGREQGRRNPPTPMTDTHDDQSGQDKALPVGPGWPIRGLPAGKH

QEGPVGLPLEPEAMEQTQLLEWEEDGDPPGGEADREAPQPVGRLHLLSSKYGPEQDFWIYPGENVVGRLP

GCQVCLPAPSVSKAHAVIEVPAPGGPHLLYDRGSLNRTRRQRGVLLPHVRYSLEDGDTLMFGDVGCQYFL

LPPESADPEDSLEVPPTQPRAPNTLAIEETPAPSKRMGYGPLLARDSEDEEELQSGGRLLHLPGSNGSAS

SSDAGVHSHTAFSSPGATVVPESDEEGGDTPDAPCPSLRLSYGGERTPGPPENGVAALGTEQSRAPGVVG

TDPEPMLAACRFVKCPGHMGIASGAGPGAAVRGLGNGCPAEVGSITDVEEVVQHPDVVQKNHGPALLGDS

DTDDDVDPKCHPSAIEMGSDTDVEEEARNPDVHPKDHQLAGNGDCDTDMEAPLENPDVVQGMKNQPAAPG

DSDTDVEGAGVNPDVGPKSYQPSFEVDSDTDVEEEAAAAAANNPDLRKNHRHPLPRGSATDVEGEVENPD

DVHLESHHSAKNGESDTDVEEVAENPDVVQKSHQPAVEVGSDTDVEEEKETPVVVQNNHQLVSPGGGDAD

VAWVVGKPNVRQTNNELAASEDSDTDVEESDRNPDVVTAKSHPLAVDMGSDTDVGETGNPDVGTKSHPLA

IDVGSDTDVEEEEAKNPDVVMKNDPHALPGDRDPDVTGTVGKRDVHPKGHHFAENEDSNTDVEESVGNPD

VVQRSHQPTLEAGDDTDVEEAEEKSDVGRRNDPLPVATGDSDTDVEENPDVGQQTLHWPSGDSDTDVEGA

PGAEFDQTPGPVGGPVQAGGEQPPAAQSCVSPETKLMDTEGNKDPAWGPGQPGDDSETDVEDDADFALQA

TQCYLAEETLSSGAEATRAPTAADSGSSLEEEATQTFVFRSPLPAQGSVMSPPAGPALHAPVCTSSEKEE

DSDEDLCVVEATQSFCKDPGPHSDQPTQPFLPEEDTQLLPRPPPPGGVLSQEPAQPAVPGLASGGLLPLG

PKEESEPPGGAVPAGGARREPPEEEESQPVRLVLSAPRRPALVLRGEARQAGAEPRGSSSGQRLRGQGGP

EAAERRETPAAVGVTGAGPHPMGPRPEDAGKVPEAHSSPSTPMRRRSLRSAPVPAPPPPAPAPERRSRRG

GAEKSVGSGEPSQPAPAAPQRRGRHQLCSTSMYVEAPEPGSVKEVEQPGPVKRPRRAVRTQEDPPTEPET

SSQGAPGRARRSRGAAGTEPAAANEPRRGSGGAEQEALQGRARRSQRAPVQEPAPRRRGAAEPARETQGK

RRGVATLDPPPAQGEGRVGSAAAQEDAGSDARKRQEVPVAPLRRQSTENKVEGLRTRAPRRSQGAAGGMA

SPRVLFTGVIDEAGERVVSALGGALARSVFDCTHLVTDRVRRTVKFLCALARGVPIVTLDWLDKSGRSAC

FLSPSGFLVRDAEQERNFHFSLAQSLQRARRGPLLQGYEIHVTPNVKPEPEHMKDIIQCSGGVFLPHMPR

TYKDKRMVISCPEDLPRCRPALSARLPVASTELILTGILQQAVDLASYRLAGPPAAPPPSATAPATRASK

RKGASGPAPAPPRSAKRRR

>Gopherus flavomarginatus

MVPGRHSTRTSASSLCSKRETGPQGAAGREQGRRNPPTPMTDTHDDQSGQDKALRLGPGWPIRGLPAGKR

QEGPVGLPLEPEAMEQTQLLEWEEDGDPPGGEADREAPQPVGRLHLLSSKYGPEQDFWIYPGENVVGRLP

GCQVCLPAPSVSKAHAVIEVPAPGGPHLLYDRGSLNRTRRQRGVLLPQVRYSLEDGDTLMFGDVGCQYFL

LPPGSADPEDSLEVPPTQPRAPNTLAIEETPAPSKRMGYGPLLARDSEDEEELQGGGRLLHLLGSNGSAS

SSDAGVHSHAASSSPGATVVPESDEEGGETPDAPCPSLRLSYGSERTLGPPENGVTALGTEQPRATGVVG

TDPEPMLAACRFVKGPGHMGIASGAGPGAAVRGLGNGCPVEVGSITDVEEVVQHPDVVQKNRGPALLGDS

DTDDDVDPKCHPSAIEMDSDTDVEEEARNPDVHPKDHQLAGNGDCDTDVEAPLENPDVQGMKNQPAAPGD

SDTDVEGAGVNPDVVGPKSYQPSFEVDSDTDVEEAAAAAANNPDLRKNHRHPLPRGSATDVEGEVENPDD

VHPESHHSAKNGESDTDVEEVAENPDVVQKSHQPAVEVGSDTDVEEEKETPVVVQNNHQLVSPGGGDADV

AWVVGKPNVRQTNNELAAPEDSDTDVEESDRNPDVVTAKSHPLAVDMGSDTDVEETGNPDVSTKSHPLAI

DVGSDTDVEEEEAKNPDVVMKNDQHALPGDRDPDVTGTVGKPDVHPKGHHFAENEDSNTDVEESVGNPDV

VQRSHQPTLEAGDDTDVEEAEEKSDAGRRNDPLSVATGDSDTDVEENPDVGQQTLHWPSGDSDTDVEGAP

GAEFDQTPGPVGGPVQAGVEQPPAAQSYVSPETKLMDTEGNKDPAWGPGQPGDDNETDVEDDADFALQAT

QCYLAEETPSSGAEAARAPTAADSGSSLEEEATQTFVFRSPLPAQGSVMSPPAGPALHAPVCTSSEKEED

SDEDLCVVEVTQSFCKDPGPHSDQPTQPFLPKEDTQLLPRPPPLGGVLSRELAQPEVPGLASGRLLPLGP

KEESEPPGGAVPAGGARREPPEEEESQPVRLVLSAPRRPALVLQGEGRRAGAEPRGSSSGQRLRGQGGPE

AAERRETPAVVGVTGAGPHPMGAQPEDAGKVPEAHSSPSIPMRRRSLRSALVPAPPPPAPAPERRSRRGG

AEKSVGSEERSQSVPAAPQRRGRRQLCSTSMYVEAPEPGSVEEVEQPGPLKRPRRAVRTQEDPHTEPEAS

SQGAPGRARRSRVAAGAEPAAASEPRRGSRGAEQEALQGQARRSQRAPVQEPAPRRRGAAEPARETQGKR

RGVATLDLPPAQGEGRVGSAVAQEDAGSDARKRQEVPVAPLRRQSTENKVEGLRTRAPRRSQGAAGGLAS

PRVLFTGVIDEAGERVVSALGGALARSVFDCTHLVTDRVRRTVKFLCALARGVPIVTLDWLDKSGRSACF

LSPSGFLVRDAEQERNFHFSLAQSLQRARRGPLLQVRPPCPLVRPPCAMREALLQVRAPRPLV

>Dermochelys coriacea

MTGTDSDQSEQEKALREAPGWPIRGLPAGTRLEGAGGLQLEQEAMEQTQLLEWGEDGDPPGGEADGEAPQ

PVGRLHLLSSKYGPEQDFWIYPGENVVGRLPSCQVCLPAPSVSKAHAVIEIPVPDGPHLLYDRGSLNRTR

RQRAVLLPHVRYSLEDGDTLMFGDVGCQYFLLPLVDADPEDSLEVPPTQPRVPKTLAIEETPAPSKRMGY

GALLARDSEDEEESQGGGRLLQLLGSNGSVSSSDTGARSDTAFSSPCATVVPESDEEGGETPDAPCPSLR

LSYGSEKTPGPPENRVTALGPKQPRAMGVVGTDREPALAACCSVKGPGHTGIASGTGHGPAVGGLGNGFP

AEVGSVTDVEEEERHPDLVQKNHGPAVLGDSDTDVEEVVENLDVVDPKSHAPAIEMGSDTDVEEEVENAD

VYSKGHQLAGNGDSDTDVEGAGVNPDVGPKRHQPRDQMDSDTDVEEDVAKNPDVQQNHQPTFPGGRETDV

EGGVENPDDMHPESHHSAKDGESDTDVEEVVENPDVQKSHQPAVEVGSDTDVEEEKAETPVVVQNNHQLV

SPGGRDVDVAQAVGKPDVHPKGHRPAGNEDSDTDVEEFVGNPDVTAKSHPPTIDVGSDTDVEEEAAKNPD

VVTKNHQLALPGDRAIDVMGMVGKPDVHPKGYRPAGNEDSDTDVEESGGNPDVVQRSHRPTVEAGDDTDV

EEAEGKPYVGRQTGPLPVTTGDSDTDVEENPDVGQQTLHLPTASGDSDTDVEGAPGAEFNQAPGLVGGPA

QAGGEELPAPQSHVSPEAKVMDTEGNKEPAWGPAQPGDDSETDVEDDADFALQATQCYLVEETPSSGAEA

ARAPAAADSGSTLEEEATQTFVFRSPLPAKGSVMSPPAGPALHAPVCTSSEKEEEEEDLYVLGATQSFCE

DPGLHSDQPTQPFTPEEDTQLLPHHPPLGGALSQEPAQPAVPGPASGGLLPLVTQAPAAARLSPKEESEP

PGRALPAGGAGRELPEEEESQPVRLVLSAPRRVTLALQGEGGRAGAEPGGSSSGQRLRRQGGLEAAGRWE

TPAAVGVTGAGAHPKGAQPEDARKVPEAHPSPSTPVRRRSLRSAPVPAPPPPAPAPERRSRRGGTEKPVG

SGDPNQPIPAAPQRRGRRQLCSPAVYMEAPEPGSVEEVEQPGPVKRPKGALRTQDPPTEPEALSQAAPGR

ARRSRGAAGTEPARVHEPRRGSGRAEPEAPQGRAQRSQRAPAQEPVPRRRGAAEPAREARGKRRGVATPD

LPQARGEGQVGSATAREDAGSGARKRQEAPVPPLCHQSTENKVEGLRARAPRRSQGAASGMTSPRVLFTG

VIDEAGERVVSALGGALALSVFDCTHLVTDRVRRTVKFLCALARGVPIVTLDWLDKGYEIHVTPNVKPEP

EHMKDIIQCSGGVFLPHMPRTYKDKRIVISCPEDLPCCRPALSARLPVASTEFLLTGILQQAVDLASYRL

DGTPAPPPPSAATPATRGSKRKGASGPAPTLPRSAKRRR

>Drosophila melanogaster

MADVSLFFGGLPAILLKADTIYRIGRQKGLEISIADESMELAHATACILRRGVVRLAALV

GKIFVNDQEETVVDIGMENAVAGKVKLRFGNVEARLEFGEDHDEVHDSSGFGECLKNGLN

NTTLDSMDVPETQPPSANTSVNTTADSLFIPETQAVLCERPSTGQRVSLGDDFMIPETQD

MLADLPPEPPVFKPPVIPVVDKPGPTIDSDEESSQGSIIRMCTQDYNEDAIDDFDTSQVL

CDVLLPLPPPTAPIEGLENQDTKRDQLDTTDMEMSALNWSASNSKCCALSSTKADDILPR

GDACITPDLTAPSVDRNICTPDLFDLIMGGDRRRDGSSSPDPFVRPADNTNTATPQFGGV

KQLVVATIETPTATPDTNSQEKNQDMIATQRFPRHKLLESDEEDNGQNNQDFVATQAFNL

GRPQAPQENEANQDLIATQAFNLAPPQKVDSPKPSTANDASNQDFIETQAFNLGIPQANM

ASPENNEISNQDLIATQAFPAKINSSRCQDFVATQPFHVVNQHSVSLQDKENIPLDDSVK

VASDSKFFEATCGEIDAVLNEMISGTVATSPVSPNEESIPFFEPCVIKDKNHYQKICQIE

GVFSNVSNRSRGRTDGTGFGRLKRVAKSESPPETPIRKDRRRSEGSERPGSNSRERLNNL

VDKKSEALNKSVIREHGIDSIPEEITKEDKSDSEEKGDCFNNVVNQWRSRKRRIQSRPGT

PGSSSERNEVNADVVDKPNEEPPTKNIRARKAKKAEKADSSKDIPKPTTRTRRQTSDEDV

RTPDLGKGRKAKNTASSKADTSKNIAKGSKRVTRQNSADDAELLEEDSSVQTESGKRVKK

VKSSKGETSKDIPKAKPRTRRQTADEDAMTPKAEKERQIQGEETKDMSKPAARTRRKASA

EETESAAQAVKPKRGGKGKKNETTKAEATVEKPMVRTRRMTIAEDSSTLTEVKTGGALIK

RAVVRISRVSIEQLESSTSAGNNARVTGAAQSRSTTPSIEEPSSSSAAAKEPNVSTATTR

RRAKRLTSGEEPTSSSDVPTKKPKMSVDEEVLKNRSGATTTRFSQPDADPLRAINLYVRK

AKTTTGKIKVAFTMCNRPALETVLKSLKHVVEITEDPLQCDLLVMDKGERTYKFLTVIAS

NKPVLSTNWLHSVKKTRSIDIKADHLFSDPTFEETYKFKPSSVLEHPRLLYGLHFMLGKD

IVPKANEMKVIIHSAGGKVHAQPPSLAISVDLYVVTTSKDTKSKRRLNNYEKVHFIKTEA

VMQALVQHNIEMLQEHTLKL

>Bactrocera tryoni

MSEKKWTLDVDSNQYDLVAGKLYLIGCWKDGENIKQCVDIPLKSQSVDTKHCIVDVSSSKIYIFDLYSAS

GTFVDNKRLNPMIKEIINVNSELRFGELSGSLKEIENLNFADPFLAPTQRAPRSSLSNTSTRLFSSPDVS

LLNDTRNESFDIPETQNMKQRASIGNSSILSSSRNQSYDGKSFCDESFIPETQMPSKTEQSYEASELGSN

LQKSGDFIRICTQDFNENLFEDGDDDEAMFSSLVIPNIQHKPVILTDVTLNMDVDEDVSQIDKCDVEIEA

LNTQAPKNGTSNDTAQNTICTERDDVCTPDLFDLPEMAQINVQANTNGSTVENLEKDILKEGQVNEVIGG

DGYAEEVDMMATQVFVPISKQPTPVVTGLTHTSEELSLSGKENIANTDFSLEQTQVFAPIKEGNLVASRS

FDKSATTTIENIQENTSLLAPTQIFTSNLNDSVSQSKIWNKTEPNDKTIKPAVRPKPNLTITPNEIKAKA

KSDGDYSNETDNGSARKEGEQALFKKPNLNKINKGSKRSTSSINSSQSEAEGSLLCTPKWICEKFDLLSE

EHVKMPNHKRNLFGSNSEEESLELNKLDKRKDSTDFDKLLCNVKEKQPLTKYVPAKSPMIKQEDRADVKK

LGSNKKDQSAKEEAILEKPEKKLEEKSYLKKPKSSPPSIKNSTKEMSKTDGKLEPSSGNRRSSGRNNKHV

EEDEKREERKKSTKNISKKDEHKHEIEAGSRKVDHKKEVEKNEKHKKQNKNVSSKDKNTDEIEEAPTRRI

TRLKSRTSEKDNDAATCSTAGPTPAKLPKIEHTDKPEISKKRVTRSRSKTDSQESITSTKSTSSSRSASK

KKAREVEKRGKNNSDDEDDKTSANASNTGKVLENNEPESKTSAKDHNRKRSAGEPSVEESKKLRSNARIL

QIAMTMVEPKLFQSLVENSPGNWCVANDPTDADVLVMDKGNRTLKFLIAMAKGIPIVTSKWLQSFNSTKT

VPRGITHFFRDHDFEKRHKFSLFKSLELARTQKLFQGYDFVTTPSILPRPSEIKQIIECAGGKVYDEPPP

PKSDQKIYVISTLNDKKYWHKYRRSNTNIRITDSEGVMASVMRQSSQPLDLNVFA

>Lucilia sericata

MTDNASFTMFVEINSQRIPLDIGQLYLLGKQNEESVNKANNFIKLESEEDVEHKHCVIEVGHGEDVYVFD

LFSSKGTLVNNTLLKGLEKLRLKNGDILKVGKQEIKFHHDQQEVSHDSSGFLEVNKNTSDIIPSSPDSTS

QHESRIKRTNYNNFLVPALPAKKATTPNNSLVIPETEAANASRNSLNVSTNSRRSDSFTIPETQYCRARN

SLNASDLSIAESNPDVSGNKIKFNFGNLDDFDNDDDFCIPETQEVLPAGGGISSSQRPRLLSEVKEKSIL

LEESTKEEAGESANEIDGSQFRICTQDYNEGFGEEADQGLHSQIIPLIKHHTVILNTTAKSCNENENSRN

HSKLDEEDKEISSVKWSNSKIDQETTALRADCSTPDIFDFEGLQADTEKAKEPDNVTKSSLEVKLNMPVE

DEEDLANTSFLPSATKHIENKGSNVEDKPCLEKEKEEVLTLSDKENRCPITLEPITDVPPTQLFEKSCYI

LCRNGENSFKAENFASIIFQSSRLRKTSSATSTTSETGFRLXMCTPQLIKEHLHVDQTEDXRKNILAVKN

NICLGDDTDEEEKEEEXXSSDLVKLINIPKDSLDFDKLLPHLKSQPEANKLNKEQQAAKEAQKLYKFNIS

FGKEQDKNQAKAAQSKSSQLSRDERHAARDKERELAERKGRSKTKDSSRNHSKDESKEKSQEKKTKQKET

KAKKAQSSTKQDQEEQKSLAKQDEGEQKSSTKQEEDKHKSKTKQEQEEQKSSTKPEKTTRQRKRKASSDT

TESAKEIKKDETTKEMPARRMTRAKSRHEEKPSTSKEAIVSKTTKHKPETVDDLDSISTQPYEEDDMDSI

ATQPYEDDLNIKSTTPIIRLTRCRSTSKQSESQDSITTNNLLRKTEQHNKSKNDSSLSHNTSANGSITTK

TSGERKRKAEATPPVVPQKIERKRLRSTHSQESATTKTNSTPTTPTLISMTMVDPELFQDLIRNSKGFWN

VAKYPADSEILVMDKAFRTFKFLLAMARGIPIVTSKYLKKLNESKSPKSVKINDYLFTDEEFEKKHKFSL

FKSLQMAKKHKLFQGYEFVMTNNILPNPQEIKAIIEASGGYVHEKNPPAAKDNQKIYLVSTKEDKKDWHK

YRRINKNIIIVSTEAVMSSIMRQTCERLNCYTLS

>Zeugodacus cucurbitae

MVEKKWTLDVESNQYDLVSGSFYLVGCWSDGENIKQCVDIPLKAQSVDTKHCILDVSDNDIYIYDLYSAF

GTFVNNRRLNPMRKECIDVNCELRFGDLNGSLKAVEVLNTTDTFLAPSHRAPRASLSNTSTRLFSSPDVS

LLNDTKNDSFDIPETQNIRQRASIENSSILSSSRNQSYDGKSFCDESFIPETQMPSRTDPSYEASELGSN

LQKSGDFIRICTQDFNENLFEDVEDDEAMFSSLVIPNIQHKPVILADVSLNMDVDEDASKTLECDGEVES

LNCGNQTAKSVTLTDPARNGICTDRDDVCTPDLFDLPELAHINVQTDTNLPAEKNMGKDNDLAERQVSEV

IGGDGYAEEVDMMATQVFVPIPKQPEVKVTDTITTTNGLSLSGKENIANTDFSLEQTQIFAPVKERSLGA

QNLNSTSSDKSAAPTIQNNDENSSLLAATQIFINTVESNDKSIKSAVKQNLNNTLISSGINENAKSEGNF

SHEMNKSSSKKEYDREKQACFKKPKLSNKNKPVKESTSSIGSSQSEPEESILCTPKWITDKFDLLSKQRS

KIIMKRNLFGSDSEEDSIELNKLDAKKDSKDFDKLLCNLKEIQPPKFVPSKCSVVKQEELTDVRKVSNNK

NNRDSRAEVVLENTDKKLEGVSPGRKEKNSPPSTRRSSKEKSKPDKKVESHSSHRSSSGRHIGHNEDREK

SEEPKKRTRKISQKDEHTNEIEEVSTRRMTRHSDHKQELEKIEKNKKPAKNVSSKGENTNEIEEAPTRRM

TRLKSRTSDMEYDKASSSTSGPTQAKLRKIEQTDEPDINKKRVTRSRSKNDSQESITSIKSTTSSRSSSR

KKNAREMESREKIVTDDNTSAKVSNSAKVVEKNETGTTTSTKDHSRKSSAEEPTVAESKKLRNNARILQV

AMSMVEPKLFQSLVDNSPGNWCVANDPTDADVLVMDKGNRTLKFLIAMAKGIPIVTSKWLQSFNSTKTVP

RGITHFFRDHDFEKRHKFSLFKSLELARSQKVFEGYDFVTTPSILPRPTEIKQIIECAGGKVYDEPPSPK

SDQKIYVISSMNDKKYWHKYRRCNSNIRITDSEGVMASVMRQSTLPLDTNVFA

>Drosophila teissieri

MLPYCSLSTVFGARCNVQGSPARMADVSLFFGGLPAILLEPDIIYRIGRKKGLEISIADESMELAHATAC

ILRRGVVRLAALVGSIFVNDQEKTVVDIGRQDTLAGKVKLRFGNVEARLEIGEDHDEVHDSSGFGECLKD

RSSNTTLDSMDIPKTQPPSANTSVNTTADSFFIPETQAVAFERPSTGGRVSLADDFMIPETQDMLACLPP

EPPVVKPSVIPVVNNRDPTMDSDGESSEGSMIRMCTQDYNEDAIDDFDASQVLCDVLLPLPPPPKPMETI

DKKEDKRDQLDTTDLEISALNWSASDSKCCALSSTKADEMLPRGDACITPDLTAPSVDRNICTPDLFDLI

LGREGRDGSSSPDPFVRPADKTNTATPQFGGVKQLVVATIETPTATPDTDSSSSQEKNQDMVATQRFPRH

KLLECDEEDDEQSNQDLVATQAFNLGQPQAQKDKETNQDFIATQAFNLGRPQITDSPKPSTANDSSNQDF

IETQAFNLGRPLANNSFGPPKDNETSDQDLIATQVFPAKINSSRCQDFIATQPFHVVNQHSVSLRDKENI

PLDDSAKVATDSKFFEEPCEEIDAVLNEMISGTVVTSPVNPNEEPIKFFEPCVIKDKNHYQKICQIEGVF

SNSSNKSRWRTEKSGGRSRKRVAKSESPPATPSRKNRRISEGSERPESNLIKRRDNPGDKKSEPLNKSVI

REQDIDSIPEEETQKDKANDPFKKVVNQWQSVQNRSGTPGSSSEKNEANAEVVDKSNEEPPTKSKRGRKA

KKAETNKADTSKDIPKPTTRTRRQTSDDDALAPDLGKGRKAKKAASSKADTSKNIPKGVKRVTRQNSADD

AEVHMEESSAQTESEKKVKNVKSSKEETSKDIAKPKPRTRRQTADEDAATPKAEKEVQVQVDKAKDMPKP

AARTRRKASVVEAESTEEIVKSKRGRKRKETETNQTDAPVEKSMTRTRRKTIVEDSDTLTEVKPADALIK

RAVVRISRASIENPVSSLSSANNARITRAANSRSATPSIEEPSSSTAAAKGPNVSATTRAPRTARAATSS

STAPNPDQSSTSFGSSRGSKRLASREEPTSSSVTTKKPKILADEEVLKNRSGAISTRFSQPDSDPLRAFN

LYVRKAKTTGKIKIAFTMCNRPALESVLKSLKHVVEITEDPLQCDLLMMDKGERTYKFLTVIASNKPVLS

TNWLHSVKKTRSIDIKADHLFSDATFEEIFKFKPSCVLEHPRLLYGLHFMLGEDIVPKATEMKVIIQSAG

GKVHNQPPSLAFSVELYVVTTSKDTKSKRRLNNYEKVHFIKAEAVMQALVQHNIEKLQEFKLKL

>Pangasianodon hypophthalmus

MDATQQIEDSFFGEEDDSDDEHELGEGKKQREPLALLKVFKNNHIPETEVPLYQGENVLGRDPASCSVAL

QARSVSGRHAVISISVLGPNDRHAHGEATEALLWDLGSLNGTRKGRLKLTPHVRYALTEGDSVVLADLPC

QYVSLKNTERNTHTSTADGGREKAKGPTSTSSSSEGGTGKGVENGGKKSALPPVPVWTDEAKSLQSLQTT

PKKPERTLVPESDSDSDGEKHGRKERRRIVASDSASSDLSSPTCSTFLTPANKVIPESEDESSITPSSAL

KGRFLKTGNDTEVSSDSSKPDPLHFNIDSDTDVEDEEVEMTKATSGVEVAVTAAVDDVNPADLHMDSDTD

VEEEEMEKAKRDPEASAVPVKQDSVNPAAVTMDSDTDVEENEPVKTDTTALSGPEAALEKKKEPARSAQL

NMESDTDVEDEDVAHIQDSRPPTSHVAEFNMNSDTDVEEDEETTKAIEPPSGSETGSKDLGKQAKSVDSN

KVPDAKMLHHQSDSDTDVEDDITEIRTPTVAKATGNSQTRSEIQTADECSTDTWPPAETVRDEFRLDSDT

DVEEDEEKMEEKEQKCAEAAVQIFHSSTPRGAGLSEEEMETEAFLSPSQLFKRPVLPSLLHPSVSPGGIS

HTDDDFAVAETQSFVCDAAQADATLDETPQSLDSPEPRRSDGASSFQLGLSDGSHQLPEAEEHDQEPAEE

DWQLQATQLYAMKNSEGNTKAGQTQLDLDATQAYTDLMGQEEEEDGRKEEEEEETQPLAALGHSSIHTAE

TQLIKHRAQNEDNSDHDANEEVVASEPERKAKEDVNDEMQVDSHLSTADTLLLVRSPRQEEHTQPYAFLA

AQIQKEQEETEKEEHKSRDEEVQETDEGERAQNEMKESVEQGNDDEEEGRQTKEMKNEVTRDGPEEEKKE

RDENKRVENDNEVDEARTQPAEADTIVETLPMCEEEEGQEEEQLNEPSSSRGTQPAKAESTQPLEPDIHT

RDAIAETLPMCEEEEGQEEEENKPKNSRRPSRRRQTAKAEPKLPVESDKVTHVTTAETQPMCEEDKEEQE

EEQQSEAMSTRRSSMRRHNAKAKPAKPVKSHVAIPETLPKSCRGRQTAKIESTQPLKPDSTAETMKIEEK

EEEQDTRRSLRGKERRRAESGKGKGRGRAATEKEKRGKVEQEADNEEEVDGGNRGRGRKDLRQKSKDDDK

ASLELVEAENEEIGHLKTQEDEEESKRVQREKKEEEARDRLERERKEQEENTRLEKEREEREECEKREQE

EKDRVERERIEREKKEREEKAIKEREEMKRFEREKQLEKERREQEEKDRLEQERIERENKVKEEQEKLEK

EMRERQELEQKLEKERKKQEEKDRLAREKRDREEKVQLETEMREEKEKQEKELEQKEKEKESKDNKEGVE

KEKRQARGKQKTGLGRKGRKSKKEEEQELEEGTKERVETEQESCKIQKQQCEEASGKKQEEEQADSDTKP

RQGRRTTRKSVAPPAGVEDEGGPAKRTRSRSNSSNSVCSEQSTQDSLSEGRRTGGRRKMAEEVNERSRPS

GRRTITAAASSEKEDIKQAAPSRSNSRSSDRSSSSVATQSRGRGGKGRKSVKVEEPEEEEKGEQSAAVGR

GRGRGRGRGGRRPGANDIKAEASGEAEKEEDEVMAVAEDSVTSQSSSRGRKRGADVSMLTAESPQPTPKT

PRRSLAGQTHKVLFTGVVDEDGEKVVVRLGGGLAKGVGDMTHLVTDKVRRTVKFLCAVARGVPIVTPDWL

SKCGKAGSFLSPNAFLVKDVEQEKRFNFSLQEALRAASSQPLLQGYEIHVTPSVKPEPAHMKEIITCCGA

RFLPRMPSAHKVQTVVVSCEEDRALCERARSLSFPVVSAEFLLTGILQQKVDMQTHTLSLSPTATVPKPT

ARTRGK

>Alosa alosa

MDATQVVQDFPFDDDEEEQEDDSADEEEAQPVAKLKVFKNVHVPETELLLYPGENILGRDATFCTVPLPA

SSMSKRHATISISVFRSNGQHASGAIEALIWDMGSLNGTRKGRLKLTPHVRYALSEGDKVTLADLPCQYA

PVGTAEGETGKGRARTPDCLGGERGEQTLVPQGDCYKVNLRTETHTEAVRAVERAANGERGFLTPSSGQH

GDAKPRGSHGMSARLEQKDQSPAPLVTASDSESDGEREGWRPRATKTLVSSDSDSPRMPQPTCSTFQSPS

GTSYIPESPSSSVRGRVNETVSSGGSIWTDRSTEAALNKPRPDPLEFNMDSDTDVEGEEETGEVEGRSSP

HAANGEASADLVAGASAGPATKHPDYHIDRNTDVEGDDHEVSDINSTKASVCEVGENVSSPAAVAAAATA

APSDFHLESDTDVEDEDEVGQTVQMAPPPAHTAPGTDVTESMPANAASAEFHMDSDTDVEEEGDDQADVT

QADVNTAASTAAQDKPEEPQSHSASDTTDDDDPFKSGSSSKNTEACAGQQPISAAALEIQSDSDTDVEDE

SAGQQAAPSAPLGSPHPSGLQPTAEAAPGAPESTADVEEGTGGGKQPASPPAVTPAKPDDFNMDSDTDVE

EALQEDTRPRKPGERASAAVLQSSTPMGAAGLRLEEMETQLFLSPADHFARPVGLPPFNPATSSDDDFVP

ETQSFVAKPHGGGAPPGLSALDETALEEETQLFCVGGVTERSAQLQLSLSDSSRLQQQLATEATQAYALP

EIRDSDSDGDSDLDATQDYGALQATQAYAAEPVRDEEDGHVDSATAETQAFFITAPRSTDIAVGLAEATP

LPQREVLKEVAGPGEEEEATQLTELDTFSHISTADTVILPRSQRLGGEEAGRMSDVTPTDLVASATAHES

KQANVPAAEICMDSDTDVEEEKDGGLKESRPTTAQIKAEELQLHSDSDSADTGDDDPFKPGTSATTVTST

GGQASHTTSSVSCSETQPMSALNDDDEEEVEEEEPKVAPSRRKSRARRGQGRHDGQPSETLLIAETQPVS

VNDDDDGDAEEEASKPASPKRSERTRRGKRRPVGDEVESAEAAFSSCLLSTAQTQPMANVDEEDEEEEEE

AKPVGPRRRGRTHGGRGAQVEDGLEVTETLVNSDLAVIETQPVSAVGDENGQDKGEQGPASPKRRGRARR

GKGKQEEDESKPAEMPSSTCLTTAETQPMAACEEEEEEEEDTQAAPSTSRAKTRGAGKEPVTTRSTRGRG

KVEGEEREGEEVEEGTSVESRRQTRGKTPAVKRGGRRRGEGAGEEEEEAEEKPVQEVRRGRGRKSVNERK

EQEEKNRLEKERLEKERQQKEKRERIEKERKEAEARERQEKERLEKEKEERERIEREQREKEERERKEQE

EKERLERERLEKKRQEKEERERLEREKREKEQKERKEAEEKERLEKERLEKERQEKEEKEKVERERREKE

EKERKEAEERERLEKERLEKERKEAEEEERLQMEKRKKEEREQEKRETSDPKMEKPESDDKPQTSTRGRR

AASRRAAPAPPSGQEEPVLPSDDCPARRTRSRSSSSNSVSSERSTSSVRSSASRQGAPEPHSRRSSRRAS

RAPPAGEQTPALEDGSSESQSGRRSRRPSTSSNSVCSEVTEAASVASQSKGRGRGRGRKSVKGKDPVAEP

EPAPSETQGTEPSATKTSGRGRRGRKSGAGDEATVGVSAQADDEDKPAEEEAGPAPRGRRGAIAPKPDSV

PQKATGRGRGRGRKAGLSSQSTSAEEDSESADVASADVSAPASQDRGKKRSHDEELEELDETEEVRFKIP

RSKGKVPKGHRKAESEDAAGGAELDRTDEETAPGPAERKGRGRPSVAQKKKEVKKEVEESEEAAAAEPDE

SSAKGSRKRGAAAAESPTPTKSARRSVGTSRQAHKVLFTGVSDEAAEAVVARLGGSMAKGVSDMTHLVTD

AVRRTVKFMCAVARGVPIVTPDWLKKCGRAGSFLPADEFLVKDAEQERKFNFRLQESLSVASSQPLLQGY

EIHVTRSVKPEPAQMKDIIVSCGARYLPKMPSVNKPQTVVVSCAEDAALCSSAVAASIPVVSSEFLLTGI

LQQQADVLAHALTIAQAPARGRKKT

>Myripristis murdjan

MDATQLIDDSIVESEEEETEEEESENKKREPLAKLQLLKNTYLPETELPLYLGDNVLGRDPNLCTLPLSA

PSVSKRHTVISISVYPRGGRHGAADMEALVWDLGSMNGTRRGRLRLTPHVRYALSEGDRLEVADIPCQYF

SCAADKKSGQGDMRSPVNRNSGESARRTDPLMEKRCDTSTDSAKCVNGGPEASTMVSSPDQQNTMKTPAR

TRCLSFEQTPNEPQCTLVPESDCDSDGEKGGGRGRQEKAVVSDSDSCISSPTCSTFLSPTNKIIPESEDE

SPITPSSTKNRPNKSVSFSKEEKDVDVGRQLKKKKAQVIVSDSDEEEGREEERAATGERQPEESGQDVPV

KWENNVSLSGGDGLPVSTPAVSTDAMPVFNMDSDTDVEGEEEEAVAVSAIPVTSNATSKADHSSKQVQFH

MDSDTDVDEEDDALAKVSNSVPASMDSTKPADSVPVVQAIGVMLDSDTDVDDDADASVSNAAAKAAPTSH

QVAHTADSAPSAEQKDFHLDSDTDVDEEEENDSSKVDETPSRLELKDSRVESASAAPQIMHLDSDTDDEV

IPAPATGKPPVVVADTEPPTIADTGADLDILSNSDTDLEEDAPLLPPVVMARNAVPSTAKDESVSLSAAA

LTTSTALQSDYDGDTDLDEPSVPPTGETAEKSEFRMDSDTDVEDKEVDGEVGEGQMSGLSRETRSRLPTP

SVPPLQKCSTPLELSDTQEDQDFVIAETQSFVLENRDHQGSHSYDPTLDSTQAFGLEISSGDEKDGQSSR

GGSFQLGLSDSSHLLPQAQALAMENTQAFIPVAGNVDMEDTQKYEAISSLERTSVGKDSDLEATQAYGGD

EEPARCSAKKESRVDFALEATQAYIPETQSDTEAGAEEEDKINIVTAETQPVDFPTTLLSVAETQPMSAF

EEEDNQVKDRPCSSVCQGKRIKQNERQEKGDDEEATQRQEMPIVSTAETQPLATVENEESEDLIPAPRKR

KAKALQLEDEETQPMTSSELSAAETLPMATCEDEESEDLIPVPRKRKAKALQLEETQPMTSSELSAAETL

PMATCEDEESEDLIPVLRKRKAKALQLEETQPMTSSELSSSETQPMATCEDEESEDLIPVPRKRKAKALQ

LEETQPMTSSELSSSETQPMATCEDEESEDLIPAPRKRRAKPLQLEETQPMSSSELSAAETQPVHDGNSN

SEEDEKVAKLPPPTNARSVPKNQKVLTSTLTTNSETQPIDTCAMDEEEDHEEADKKVELQKTTKQPQRGK

RQESEAGTSSISSASKRGGRAVIAEEKLEYADPPRRQTRGNGKALSTTRGRGRKVPEEDYESEETQAVEQ

SKLSRGRRTRRHQKDNSMEEERKEEVEKDRLLKEQEEKERIEREQRQEKERLERERRAEQERLERERKEK

EQQERLEHERAEREEKERLEKEENARLEREENARLERERKEQEEKERYRIQKEKEEQERLEMERKKKEEQ

ERLERERAEREEQDRLQREEKARIERERKEQEENERLEHERAKTEEKERLEREQKARIDRERKEQEEKER

FQHEKAEEEKRRSEREKQGREQLGEEAKEKLIKEQHENKDEENKLKSATRGRRAVRRTIADPSTAQVEQD

QGISTSEDGPARRTRSRSNSSDSVSSVMSAASVTTQESKGRGRGRGRGRGAKKTTNITRDSRRRTVAGEP

SAERQAAEQAGDDTPPLGVLSRSNSINSLNSEVSSCSVSSQNRGRGGRGRGRGRKTQPETDSIPPLNSQS

DQSSAPKATARGRKSRKTEGSFSDVSEIDGEEKADSMQSSTTRGQKRASENSEPPAVHGDDQSSQEEENV

GEESLVPKRNVRDKGQKEILTTLIAAAVNDGDKIKEKGRGKGRKRELEEEAEEDEGSVSKEKGKAQGGRG

IEAAKEEKEETHEENPAPIPAKKQGRASTAQGKKNTQESLPKVEVKEESKKIEEIADKKRKGRQSVAHKK

KEEKQEEDGTSAQSSMNQDASAETPETPTSSASRRRVAPADSSPMAKTPRSSSASPVVAAQSRPASQVYK

VLFTGVVDEAGEKVVARLGGGLAKGVADVTCLVTDKVRRTVKFLCAVAKGVPIVTPSWLEKSGKAGSFLS

PNAFIVKDAEQEKKFNFCLQESLRTACSQPLLQGYEIHVTKSVKPEPVQMKDIILCCGASFLPRMPSSHK

AQTVVISCEEDWSLCGAALAASLPVVTAEFILTGILQQKVDLEAHALPAPTQPAGGRGRGRKKT

>Colossoma macropomum

MDATQQIDDSFCEDDTDEEKEPSGEKKEREPLAALKVFKNNYIPETEVPLYQGENVLGRDPASCSFPLQA

RSISSRHAVISITFFHDNNRRLSDGDTMEALLWDLGSLNGTRKGRIKLTPHVRYALTEGDSVVLADLPCQ

YISLKHAERNAHTATAKEKGPSPAKSGSEGGMGRGVENGGKRSALPPVPLWEDKEANLQSPQKTPKEPER

TLVPESDYDSDGEQGGRRERRRFLDSASSDLSSPTCSTFLTPANKVIPESEDESSITPSSAGMGRFIKKE

NHSEASSDSLKPALLNFNMDSDTDGEEEDAEVRNRKPEVKPANPPQVDPVDPACLHMDSDTDVEDEEMEK

TKGPETAAGVAQKDMATPAGHAMDSDTDVEENEPRRTETSLHSGSKEQKTSSEFHMDSDTDVEDEDVPMM

TKPERPVSVPTANPVAEFHMDSDTDAEVEEKPTEAIKLISDSDTDDDDPFKPTKGRTIENVKALQAKTVQ

PDSNSDTDMEDDITAANSKPRKDTGIAPDAHCESPAQVQPPAEKAQGEFRMDSDTDVEEEEEKLEERGSG

TAAEAVQSSTPRGAGLSEEDMETQAFVSPSQPFKRPALPSLLRSSVSPGSRSQDSDDFTVAETQSFVSDA

LAADATLDETQQSLRGCGASPFQLGLSDSSHQQPDPEEHAGALDPSEDDWTLQPTQPYAVKNLQGKGAST

EARQRQLDLEATQAYAVDIDNEEEQTQPLKEGNDDSDTNGVPDTGDRRGRVVDDDGEVLDSHPSTADTLI

LVRSPKPEEQTQAYALFTAQTLPLNEEEEEEEEFSSGPGPSGRLCKRRQIDEEQTQPIDPGAKTQVAVAE

TLPMFEEEEAQEEEDSTETMSSRRSLRGGKAEETQPIEPGASTHVAVAETQPVGENEKEEQSETTLDSKP

RRGRKVAKAEPTQPLELDSNTDVTVAETQPLEGDEEEGEEEQQSEITSSRRSRRGRKVAKVEETQPLEPD

ATAHVAIEETQPLDPDGSTHVAIAETQPVGEDEEEGQSETTLDSKPRRGTKVAEAEPTQPLELDSNTDVT

IAETQPLGDDEEEGEEEQQSAITSRRSRRGRKAAKIEETQPLEPDATAHVAKAEALGEDEEAHDEEQQGG

SRRPRRGRKTAKAEPTRPPKSDASTRTATTETLDGEEEAQEEEQQASKRSLRGKGRGVSGKGRGRATAEK

EGKRKRGRAVSEEEEDSEEETDGGRKGGGRKSTRQKSKEQEDRDKLEEEKERETQEERREEREQKEQEER

ECLERERVEHEERELAERLEQERKENEERGRLKKEEEERERKEKEENQSRERKRKEEEAREKLEKERKER

EERERLEKEEKDRLERERKEREEKERLEKEKREREEKERLEKEKREREELEQRLEKERSEQEEKDRLERE

QREREEKEQIEKEVREKEDREQEEKEKKKKEKGDKDGAEKERGRLQGKRRAGLVRKEKGKKEDEEEEDID

GQSENSKRRMLEKEKDTVETEKQEHEGGKGKKKVQEKAESEAKPGRGRRPTRKSTAPPAAAEESVSVDVP

AKRTRSRSNSSNSVGSEQSTSTLDDQTRGQGRGRGRGRKPAEEVVDNSRPSGRRKTATAASTETERDSGA

RVRSCSNSRSSERSSSSAGIGTKDKGGKGRKSVKVEKQEEGQQGSAAAGRGRGRGGKRLEAGDVKVEATA

EAHREKERSEAVSVNEESVISQTPSRGRKRGADTTVLAAETPQPTPKTPRRSVAGPTHKVLFTGVVDEDG

EKVLVRLGGGLAKGVGDMTHLVTDKVRRTVKFLCAVARGVPIVTQDWLTKCGKAGSFLSPNEFLVKDPEQ

EKKFNFNLQDSLRAASHQPLLQGYEIHVTPSVKPEPVQMKDIITCCGARYLPKMPTAHKAQVVVVSCEED

RALCNKALSLGVPVVSAEFLLTGILQQKVDLKTHALSTSPSAVSKPATQGRRK

>Cyprinus carpio

MDATQQIDDPFSEEEEEEEEEEVEKEGPEREQLATLKVFKNGHIPEAEFPLYIGENVIGRDPAACSVLLP

AQSVSSRHAVISVSVFCSRKDRFGNGDDVEALLWDMGSLNGTRKGRFKLTPQVRYALTEGESVVFADVPC

QYIGLNISKKDTHITPEKGGVSKKEKKSSPALSSSDSESELSKGVRKRLSVLPPVPLWSPEDEEPKTSSP

QPAHKQPEITLVPESDSDGENTTDERKDFVSDSASSSHLCSSTNSSFLTPGKKVIPESEDESSITPSSAS

IDRFRLQTPDEPGSNSSKPGPLVLNLDSDVDFEEKDMEKSKPEPEAHPELAPKVEPVSSAEFHMDSDTDV

EEEEPEASKAEPEAQEAVTLEKSVSSVGLHMDSDTDVEEDEKSKTEQKVHTEEAPNVKSDLHMDSDTDVE

ENEAYVTENVSKKEAVTESPPSAPQHTGFHLDSDTDDEDDNPMKVSITTEVSHPAGEGSDVRPTSTPATE

LHMDSDTDVDEENEDKEKVKARDLLSNSETDDEDPFKPLPDKAVKAAWSHKASVGSKAEQSEKRTHKKQD

HYSHAKTNFEEPTQAFGHLEDDWDLLPTQAYGTAGASARLKPKQLDLEATQAYGTETDREDEQEPLNHTQ

PYSNLLTAETQLIHEAKPDDDEEEDKDTQDDSHLSTADTLIIASTPKREEQTQQFSLFTAQTQLVCEGED

KEVYQSEPTQLMTDTIDETHEGEEEIMKQGRRTAHKSKNTTSSLIIAETQPMCEEEEATDADLSGDGFQK

PSTRQQVEKYDSVHLAETNFSSHLNIAETQSMCDDIEAPDQDNMVSSTRIEIAETQPMFDIDEAPEDDLN

NEVNRRQTEGEAIRHIEKASTSSRAIPETQSVEDDEGQEKELSSKTSSRRSRRCRPKKEKEVSQTAEAVA

NHTITETQPIYEEKQIEDLKSEVSSGKQSKRQQAHELKSPELSETDLKACVTVAKTHPMCQEEAAFEELN

NVLGTRQIDEDRATELPVPSSSHINVDETQPIHEEDERQEKDLSRKMASISSRRGRPKKDDEVTQTAEAA

VLQIITETQPVPEEVIEKDEDLNSGVKSRRSRRGRPIKKDSVPQPDEAAVSSLSINTETESVHEDRTERD

EDLSGIRSKRPRRGRQKDEDEPAQSAEPNSDLSDVGTQPMVEENARGEPSGRSSRRQRKDKTEVESETTA

SSEVQGKTRKGQEGKRKRGKALSEDEEVSEEEKNTRRGTRRTGIKLKCNEKKEEERLEERNAQEEKEKME

KELQEPEKTEREEKERLEYERKKREECEQRERVETEMRQKLEREQKELEEKERLKREEEEKVRKENERIQ

REKELLEKEEKERFERERRELEEQRRKEEEERIKKEKEQKEKERLELERERLEKERRENEGLQMRARDQK

KHQKDQKNERLEENKAELGAPLVPPGRRTRRCSSSSMNSDQSVSSQREVSSQRGRGRGRGRGRGKPTTDE

QPTSARKSGRRGPASGVEDTEKDSNSTTRSRSRSSSRSSERSAHSIGPSNQGTRGRGRGRKSMKLPEPEE

VSQAKTSGKGRGRGGRRSGVENVNVGMDNEHEQHVEVVEDSTAVPQTNSRGRKRAANTSASPEEAPSPTP

KTSRRSVAIQAHKVLFTGLTDQDGERVVSQLGGSLASGVNDMTHLVTDKARRTVKFLCAVARGVPIVTPD

WLKKCGKAGHFLSTDEYILKDAEQEKKFSFSLQKSLQTAQTQPLLKGYEIHVTPSVMPEPSQMKEIITCC

GARFLPKMPSAHKEHTVVVSCEQDRALCNKALSMSLPVVSTEFLLTGILQQRVDLQAYSLTSSLNTSNQP

AAPKAAARGRRK

>Polyodon spathula

MDQTQLLDEALSAEEEEPGVNEDSASEGSQKLPMGRLRLFSSARVKETDFQLFAGENTVGREKPCTVALL

AGSVSKRHAVIEIERGSHLLWDCGSLNGTRKGRTQLKPQVRYDLQDGELLVFADLPCQYFILPPGRAIET

SNQIAVSVRKKEAGPACDFKTPSSKNVRSVPVETTATPKESKGLIERTANGSAKFQGTDPAMERPANQKA

EEGNGGGTGGGIRMVDSVSDDDDDEDYLMLQDTQAQVGTRSLALEETPAPPGRGFATPLVVDSEEEEEGE

RKLNDTSEGSGPINSTFLSPGATVIPESEDESSITPGTGPNVQAEKLLYDTDTDASKDQSTNRSKGVKPM

PLLVSSDTDSEDLAAQKGHSKSKLKTKRPAERRRSTAGKTRGERSSIEGPQDSDSEELTGTNGQEEKAVW

TRIKDQQVSEHSRYIQRLEEKGVQMVGRTPGDKTVESNEKDEDSRLGENSKGGKALAVFQLDSDTDIEEE

EERKEKTDSAAGSGNQVGAPELREATVRSAPVGLDAFHLDSDTDVEQEAEEQPVEKPSICAVKAGLNADS

ISEQQPQSGTGPIVLNAFHADSDTDVDEGEKEQPVENSYNSKGGFDVNSSSQERSPTATAAPGLIALNAF

HLDSDTDVDEEDIEEQPVGEPSSSATKAGLDVESSTEQQASTTPVAPVEVKAFHLDSDTDVEEDEVKSFS

SATKAGLDVAISTEQQAPSTLTTSATGLQSIFQSDRDTGVEDKVELSEPDATAEVEEGKPSSPPDKGATS

LHGDSDTDVEEENLAPKAGKTGLDLMKQDSDSEAEAGAAADTSSLDEEATQAFTFLPAKGRDQGAFKEPF

ASSFIRKILPSASGRGSQRPLGHPVEQSDEEDLVVAETQSFCTDADSVDSVDLALEATQQYECGLDQSNE

TAEEPTQVYSFDLGNKARATQGGGLRLALSEGTETEPTQAFMHEADTQHLAQTANALESVWSAEQEETTL

DFNCVQSAVSKPTTDRKPTCASNATADHDAETQPMLFDPFDDIEAAEDAHVQCESRTAPSFTTAETQFIE

FETQEHNDTQPIVSHQQPCESKQAQSVLPYEMETAAAETQPVDSEAAGADTQLIGFKLESQAESLTSGDA

KVEAVQFTSKTFQKQLGSESEKESVLEFMQDTVTVRARGHSRGSTTVSSAKISDDGSRSAGTTTRESEES

CACLDASTQSISFQLYLPDGEACSSEQEKKDARNDDAHPVDSTDPVPLDREEPLTKDTVAVGKTPEEISS

QGGKSGNKGKKGKAAGIVGQAAGDSGSNPLVEDECRSKGAAKRGGRLQLGRHQKRKETEAEKGQGEGESN

VASGENVEQPVVGGRSDGGDGPSCEECMEGASEMLVESTEQVATVEETGPGREEYPSGSAEASGSKSEQK

DGEKQEEEEVEAGTTPGGLSSRRKSRVTKQQTPRDRGRAKAASKAASSASGAGRGLVLAMEEHGSENQQR

KENEEPQGMKEPKIAPIFLRKSMKGPTAEAQETPVRRSGRAARTSTALETAEVSQAAGRVGLRKKRGVTT

AREEEENVMEEQEHVGLKRTRTAEDRGDAVEILHLAEQQQPSEGDSRQSEREIMEKISEVKGRESSRKRL

LSKAAPVLVLEASEEEAPNARGTRQADSGYIQPEPRGLEKTVEAGKNPKGRGGKQKLQSESDPLLLETSE

ETASEVSQEEVLKARGRKQEKSVLPEARKEAASVVEQFKARGRNLVRFGASEQDAAGEVPEARAKIGKKQ

ASMSDNLSLEAGSEEAFKVIQTLVPEIQMEPAEQEASEEVPERKGKRARKQAKTDSEASVGEVEQTPVPR

GRRQAGSIQLEANEQRGKKKPEDNEASETIAGPKARGRGGRRQPRTARDEEEEEQLQPASEGENFKIPSV

KGKGGRVSRARSKDSSEEAAGSKEDTPTATGKTAKSEEPQLQAEAEQRVPEKGRRRGRQAKTEPQDEEAG

SSELEKGSSLEGKGKRRRGGKSTASQREVEEDRVDTESNASASSEEQGDKRRRKQEVPRTPSPQGSTGSA

SSSRKRRPQSLVTGSQGTPPDPKTPRRSTSRGSITGVSPRLTGSSSVPKILFTGVIDDCGLEVIERLGGE

MAESGHDCTHLITDRVRRTVKFLCAVARGIPVVTPEWLEKCGKNGCFLSPNAFLVKDVEQEKNFSFSLAD

SLIKARRKPLLEGYEVHVTPNVKPEPAQMKEIIQCSGASYLPKMPKVYKDRTVVISCVEDAAKCKSALGA

SIPVVNAEFLLTGILQQSAELERHSLQGPGFNRQGAPADRTSTAGGRRRR

>Maylandia zebra

MACEGMDATQMISDSILESDEEDNEEEIENKRGRPLAKLCILKNKHIPEAEFPLFLGDNVLGRDPNSCTV

PLPASSVSKQHATICLCVYRRRACRSEVDMEALVWDLGSMNGTRKGRLKLTPNVRYALSEGDSLVVADIP

CQFVICGVDSSQGDASSPLRQNSGVKTKLPDASGEKGDDTSTDSKKCVNGGTETPVRASCLTFEQTPHQP

QGTLVPESDSDSDSETGVRGERRRRTLVSDSDSHKSSPISSSFLSPPNKVVPESDNESPMTVSSSSKNKP

NRHDSFTTQETDVDAARQQLEEKKVVALVEDSEEEEERAVQAGTKSLKGGWHMPVEKESNASFTGDGELS

TPAVSTDAIPAFNMDSDTDVEGEDEEVASAGPVTLNTNQQVDQPPKTAQFHMDSDTDIDEDDDASGKAPK

ALPSSDENAKTPHAVSVIPPEGVTMDSDTDVDDDGVVSDAATKAGPTVCQSAHTADSAFSVQAEDFHLHS

DTDVDEDEAKNTTDETPNKSDIKLTALKSAPVAPDNLQVESDTDDEALPVPATSKPSVVAAVADSRPTAV

ADADLEILSDSDTDVEDASLLAIPVGVTTLSASPGPTLKASLADSDADTDVDESSVPPVGDEAKPADVRV

DGDTEDKKSDIGEEGEDQVPRLHRETTPGLLAPLLQNCSTPVQMSDEEVEDMATQAFVSPSSGSLRRAAV

PAVRPVVLSSCSDIQEEEDFAVAETQSFTFQSRDRVSSEEPTQAFVLESSCDNKNDQSTKEESFQLGLSD

SSHLQCPDQALAMENTQAFVSVERGVNLEDTQAYAAMSAADRSSAENDANLEATQDYVEDKETERYQVDV

ALEATQVYVAEPYSHAEDDADDDACETQPSDFTASSTLAIAETQPMLPIAGEEDLAAENPVCSVQQIQPR

SLKEDGPKAAQPQEKGLSDAQSVTETQPVCTSDSEASDDEGSFPAQRNRTAKPLPLEEAQTQDLTCSELS

VSETQPMHGGIAETQPMATCDDDDEDSMPGPRKRKAKQLHLEEEETQPVADSELSTAETQPVATCDDDDE

DSMPGPRKRKAKQLQPEEEMQTLASSEVSALETQPLSTNEGQQSDEEDVIACTRKRKAKPLQLEEEQTQP

LTSADVETPAVVTGKCDDKDSIPGPRKRKAKQMKLEEKTQPVDNSKMTHVETRPLTTKTGLSPSEESEAG

TSGACVPNLRVTRAKPREEEDQVACPGPSKRQTRGRSGKPLSDGDNNKEMVKRARGRKITKQQKHDGEEE

DETPVRERNKQAVNKSLIKQQDGKDREEEDGADLRQEREENDNERRTTDERKKHQDEIEMQLMEEKLETE

GRVNKEQERLQAEDAARAEREIKEKEEQQKIESERIEREERERLEREEKERLERERKEQEKETAKREQEE

RLEREQQLQARLEREEREMEERERVEKEKEAQEKQLKELEEKKEEQDKSKTPSRGRRTTRRTIASQSTAE

PDQDSTVSTNDDFPARRTRSRSNSSNSVSSERSASSVHAQESKGRGRGRGRGTRETSETPQKPVARSSNR

RTTVAAGAVEHEPDSSNSLAPEVSSCSVSSHNRGRGGRQRGRARKTEHDPDSIPSVSQSQSDQNLSPARG

RKGKKAEESSNEALHKNGKEDSQPATTTRGRQQPSAATDEEDQSKQEEACTSEESLPKRNVRGRGQRAVK

SETVEKPVAPAVSDDDEAKDKRKATRDLEANTGADLSCSKRKERSSKTAETDEEEGKGKTNDEIPVSLQG

KKRGRASTAQAKKSAKECLSEVKEEEREKMEGETVEKKVRGRQSAVSRKKKEELEESGTSLTATNQDANL

EASEPQTPTSNASRKRQAPANFSPVAKSPRSSSASPAASGRLQTEGQAYKVLFTGVMDEAGEKVLARLGG

SLAKGVGDMNCLVTDKVRRTVKFLCALAKGIPIVTTDWLEKSGKAGSFLSPSSFIVKDPEQEKKFNFSLH

ESLRIASSQPLLKGYEVHVTRSVKPEPVHMKDIISSSGATFLPKMPSSNKPHTVVVSCEEDWSLCRPALS

ASLPVVTAEFILTGILQQKLDFQSHTLSPSTANVQPAGGRGRGRRKT

>Boleophthalmus pectinirostris

MDATQMIDDSVLESDEEEQEEEENNARGDPLGKLCILKNPHIPEKECPLYLGENVLGRDPNTCSLSFLAP

SVSKQHATISVSVYRRRGCKDQVEALLWDMGSMNGTRKGHLKLTPRVRYALSEGDSFVVADIPCKYMKCS

ADVQDTRTPVTPNLKTRDVLRENGGNVNTSKTKSPAASTIKDAIADAESTRGTPDRTGGLSFEQTPTQPQ

SSLVPESESDSEEDKSRAGDRRRKNIVSDSDSYKSSPTCSTFMSPTNKVVPESEDESPITPSSSYKNRTR

RVSFNTEESDSDFNRQEQKKIVPLDLVDDSEEETGNDNKTSVDNSSVQPQISKVEPNVGVQAKAVLEFNM

DSDTDVEEDEEKPTADKAATTTAVTSSNAQTESEFHMDSDTDVEDNEPPISSDNIPEKHQNIPVVQPEGI

SLDSDTDVEEEEEKPAKTSVAPEISNNVQASNAAEFNMDSDTDVEDEDLVSAALEKPTESQTAKLEMPKT

SEINFNLDADTDVDEEEADITATPSHLDAKTPISKSAAPQDVQLESDTDDEGIINVPVASKPPEDLELLS

NSDTDVEDSSADVKPTASGLTVAECEVDSVEGAGLIKAIVSENTGDKAAPVSADSDTDVEDNENEIKETG

DNEIPALQREITPDIQGASLKNCSTPIQQQEGKMEEMETQAFLNPSISAYRRAVAPVRSDALSSCSSQED

DYAVAETQSFILNDRAQDPNTSVGSDSSVKEKDVSSTSGGHFHLELSDTSVLQNLTMESTQAYINANLEE

TQPYADAIITAAKSTPADIALQATQAYIPMQDSEEEEEGSEGGKQAKNDEGAEEEEEEEATQLVDFLSAA

PTQPMCVDEESDDEDDGVFRTKKAKQIHEMATQMFTTVDVAPTQPMEIQDDSETDEDVDVSSKKISEENE

EETQANSDVTIAQTVPMEMQEDSESDEDFGVFKKKKVAKIQEEDTQQNSDLIDTQTVPMEMPGDSESDDD

GPVFKRRTAKQLQSEEEASQEIHKDEGTDDEDSGPIFRKRKVKRVDRIEDTQPSLESEITVDPTQPIPTN

EESDEEELIPIGEKTAKTTKKKVESELAVAPTQPVVLSEDEENEEDVFPAPRKRKARKLEDTQQQNSQAD

IDDGKESGEGSSVGVRGTRSGRSKIGKEKQNEEVVVEPTKRQTRGKTKATPVSKGRRRRGKVEPESEEEE

EEEEEEKANKMAAKVQKEKEDKEKKKKEREEMERLELEKREREEKERLEKEREEKERLEKERIEQEKEEQ

AAKLQKEKEEKERKEKERLEKERIKQEKKEQAAKLRKEKEEKKQREKEERERAEKEREEKERSEAAKKAE

EEARERVKMEEEARLEKEKQKEEDQALRRGRRTGRRTIAAPESSSSTVADEDDVPAKRTRSRSNSSTSVG

SERSLSSDVSQTTRGRGRGRGRGGRKTEVQVEKAPSRXXEAPPSSPPRTLSRSNSNTSLNSELSVNSVSS

QGRGRGGRQRGRGRGRKSEIEQVSTENNSQRGRKSTRTCPKDDDEDEGDSNQPSVSTRGKRGAKDNTSKT

PENDEEQGDALTSSTERRKASTKQTETKNESKTTASQAEEQNDEDKDASLSGKRKAAARKASTKQTETKN

ESKTTASQVEDEQKDEDKDASLSGKRKAAARNPPTRKSMKVEEDDAKTDKPKARGRASTVQAKSVEEHGD

SGSSANNSVEQEEPPQTPKSSVSRKRSSPDSESDPLAKTPRSSTESPSVSRSRAASQSYKVLFTGVVDEA

GEKVLTRLGGAIAKDVFDMNYLVTDKVRRTVKFLCAVAKGVPIVTTAWLEKSGKAGSFLCPSTYLVKDRE

QENKFEFNLQESLNVANSQPLLQGYEIHVTKSVKPEPVQMKDIISCSGAVFLPRMPTSKKPNTVVISCED

DWSLCAPALSCGLPVVSSEFILTGILQQKVDLEKHKLHGPESAPTTGGRGKGRKKT

>Xenopus laevis

MDDTQRLQWDDEEQNVSLDRDQPIGKLHMFNGIHGPAQDFPIYPGTNIIGRHANCDVTLPAQSVSKKHAI

IEVQSGCHTLCDQGSLNKTRRGKAALAPHVRYALSDGDLLLFADVACSYSFTECNTKTTEEEAANEESED

DMLVPGTQATLSIEETPGLAIRRMGKVSVLAMDSGDEEEEELKEKDLHYEAKGFKTSREKCKLPASVPFS

HNMDTIVPESDEENDTSASEPRFPSLNLRCDSDTDTHETPVKAGTSYIPSKDCVFSPPMRGQNMSDNSVS

NKDEITKSTLIGHQESFADEQANTEHKTLVKCDQDTSIENDRETKEEEDASTVNTSNIAEESGTGVKDSP

LISNPKVESQDIDSVKVENQSKEFDYNKTLQAGIKQQESADISSDNDTNMEKNQTSKAKLVNTGSEGIIL

ESDTDGEEEDCTNSKEADDQKREGAAFHLDSDTDIEEGDNVSSGMNMHKVENSGNKTDSDTDVEEAKPKS

EETAVQKKVEEGFHLDSDTDFEDDDSNLLNIELKNTDKAKMIQDSDTDVEDITTSKVEVDRKTASNADTK

GDNRSTSPNAEVKMQEKDLSILDSDTDVGDGCSLSVVKKQNKAETSHTNLTDTTAVEESCTSADTEKVVK

SADRHESHTDGVIQEQKATPEFQLDSDTDIEDDDNSLDVSSTSAKVKITEVHSDNLEKQRASSEEIQEKR

VVEIDLDISTDMDEADETDVSRKEEVDEADETEVDRKEAFHMDSDTDLEDNDSGPSVVHGAEKQSTATEH

SDSTSDAGASVKNVTSTIVEKTEKGETDGIDCEGAEYDMMATQCYLEPEEKESDIQDEDNCAEEATQAFI

LSSTWAELDPFKRPANPIGVLQISAVTVSSSEEEMDENVIAETQPFCCEIRQPEEHSVQELAKQGSSAEE

KNNAACSMPQEEISQDDTQPVSQFLNTTASSNVGNWVATATTNSHQEKHLDRCNVEQIKQVDEHVPDMEG

QADECASIMAEDATQPYILDLPTVGDTITQPCNLSEPVSEDGLIQPLNTVVPAVVNTDSLPSNPSVQLSD

EEAMQPYSLNEAVATQSHVLNMPLTEDNTVSISEGEKASVRQGTCSGEISIICEQLDASNKDISNRPGEV

LPRSPVEKATEHVAEEQQQSNGATRITEEDREASKGRRPFRRSRGGEKDKDSAEVIISTTRGKRGTWKKT

EAAAPASEDTKSKKIAKLQKMEKELPEAKIEDECKEDHSSELKTSSILVDSKTGRKGKQDTKECLDDTVE

DHVKEHQVEEDVKSFTSRRSVRKRKDEKDQEAESSKSLQVSFSKKRSTRQSNLDLGESVGEKAIAVSSDE

HGKHQVDDEVFTPNKTTRNLKSKGAKRNVKDEEDNMEKGEISEKRPTGKNRENVTEKNNEKADLKRRVTR

RNSSQLETVETIPDNIETVDSERQDQSRKSPNIKKELKGDDLKVAEKCGGTTKEDTEKCISRRTKKGCKE

EMNEQSKEQTDGKQRVTRRNSRLSETVESKPEDTDIVDSERQAQPRKSRNIKKNLKRDEDETLEDTNQEN

TEKFTSRRTKRQCKERLNMEGHEKSKGEPSIEHCLPINNSKEAESEQENAKAKEQDQSKKSRKTKKDLKQ

PVVNEEEHLLETISKDPEVRTSRRTKKGCREEDPIDGSVPVISKSKQDESTRKNVSRSTRKNSKEEPKEV

DTEENVVVSKKENLDGIQTTRRARNPKVDDMHSVDKTLCASEPKSGNPDGRTRKKNQKEASGTQKNTEVE

KEMSGGSVAIEKEQLSPHEDISPTGVRKSRRTSTKEELSKSSTLAPVRKRGQEPKTGADEVKRKKVNEEL

EQEQTVGRRDQARQPNLEDENTESNSIEKDSMISLPSPLGNSRLRPSTAFESPPEARTPRRAVRNLTTSP

YTSQRSITAKVLFTGVVDPAGEETIRNLGGEVAESIFDCTHLVTDRIRRTVKFLCALARGIPIVTLDWLD

KCKKSKCFLSPAQFLVKDKEQEKNFNFVLSESLQKAKKKPLFQGYEIHVTPNVKPEPEHMKDIIQCSGAT

FLAKMPKVYKEKYIIVSCKEDSSRYKSVPSRIPVTSAEFILTGILRQEINPHAYLLSAEEALPTPAKRRR

>Danio rerio

MMDATQQIEDPFCEEEEEEEDKGDPKREQLATLKVFKNDHIPEAEFPLYVGENILGRDPAACSVLLPARS

VSSLHAVISISVFHSSKRSDNGEAVEALIWDKGSLNGTRKGRLKLTPQVRYALTEGESVVLADVPCQYIG

LDISKRDTLKTPEKGVCVKEKKESPVLPNSDSKLRKSVQNGLNDKEKTALPPVPLWSPEEEEPSICSPQS

AHKQPEITLVPESDSDGESATDERKDFVSDSASSSNLSSSASSSFLTPAKKVIPESEDESSFTPSSASVN

RFRRSESNCEPSSNSSKHGPLALNLDSGTDFEEEEMKTSKTEPLATPEVAARIEPVSLAEFHMDSDTDVE

EEELETDNAGPEAQVAKKSVTSAGLRVDSDTDVEEEENNKTEPEAQIEQSPKVGPVSSGDLHLESDTDVE

EEEEDKSKPDPVAQTETPRLAPVSSADLHIDSDTDVEEEESDASKAKPEAHNAVTLDKPAPSPGLHIDSD

TDVEEDEEEKSKTKQKAEIIKSVASTDLHMDSDTDVEEEEPEASNAKPGDLDKSESSVGQCHMDSDTDVE

DDNPVQVPQPIGKGIDVKPATSTPGTELNMDSDTDVDEENEDKEKTKDYLSNGGTDDEDPFKPVARKTVK

VFKSQEASGGGDEGEQSEGSILKKQDQHSQAKASLEEPTQAFGHLEDDWDFIPTQAYGAASTSAVLKPKR

LDLEATQAYGIETDREEEQEEPLSHIQPYTNLSTAETQLIPVAKPGDYAEEEDEETPNDSHLSTADTVII

ASTPRREEQTQAFSLFTAQTQLVWEGEGKEVDQSEPTQLIEAESNDEMQEGENEEMIKQGMKTHGTGAVQ

NSKNSSSYLIIPETQPMCEDEEAIAADLSGDEFQKPSIRKQDKKYDTSHYDKNIPSSHVTLAETQPMCDD

DELPGQDDVLSSVQTEKHVSSHIAIAETQPMFEEEEVQDDDLKTEASRRHTDKDEPVQYTEEASTSGFSI

SKTQPVCGENVRQELRSKTSSRRSCRGLSKKGKDVPLPDEAVSSPAITETQPICMEEKAPDDNLKGEVSS

GSTRQQAGEFKSIELAETEPISDEQQAPGEDMPDAARPRRIDEDNSIEFQKPSISHFTIDETQPVVEENE

GHEKMFSNKTASRRSCRGKAKKEEEVSQTAETAENQPIPEELIERAELNSEVKSRRSRRTRLIKEEKVPQ

PSDVVIGSVSTISKTLSVYEDETERDEDLSVIRSKRSCRGRQKDEGEPPKAAELNAGLTVVEAHHMFEEN

AQEEPSSRTSRRQGKDKAKEESVIPASSKVQVKSRKVQDGKRKRGKELSEDEVESEEEKIIRKGTRRTGL

KQKCSEKEEEESVEERNVQEEKEKMKKEQVETERLQKEENERNEQEHEESERAETERQKLEQEQKEQLKR

EEDEKRQENERIQREKDEEREEKERLERKRNEQEEQRQKEEEGIQRDKEQKENEILQAEKEQLEKERQEN

EARAQKERDEGSLKKGQKNKQLDKNKSELEPPLLPPTRRSRRCSSSSLNSDQSATSQQEPTSQRGRGRGR

GRGRGKQTTGEQPISARQSSRRGPAFEVADTEQDSSLTTRSRSRSSSRSSERSANSVQSSNQGTRGRGRA

RKSVNLPQPEEVSQTKPSERGRGRRGRTSGVENVDVGMSNELKQDAKMVEDNTAVSQTNSRGRKRTAITS

TTAEEALSPTPKTPRRSLASQAHKVLFTGLTDESGERVVSRLGGTLAKGVNDMTHLVTDKARRTVKFLCA

VARGVPIVTPDWLKKCGKAGHFISADDYILKDIEQEKKFDFCLEKSLQTAQSQSLLKGYEIHVTPSVMPE

PSQMKEIITCCGARFLPKMPSAHKEHVVVVSCEQDRVLCAKAVGMSLPVVSTEFLLTGILQQRVDLQAYS

LTSSLDTSNQPPAPKKAAARGRRK

>Octopus bimaculoides

MDLDVTQAITFSDVEDDAEKSKLPVAHLTILNHQALKNKLFPIFRGKNIIGRHEHSCEVCIPVKSLSKKH

ACIEVTGRSHLLYDMSSRNKSRRGTLFLTPEVRYELKHKDTLVFADVSCQYAIEEIARASDSGSNTESMS

DLEDELDEITTNTPTILKSNISLQLENSNSSSSSDILLPTQDFPVHELTKHSSQGLANISPVKAEVLAVD

SSPENQGESSYLKDRIFFQNNANKEDNIDAEILAAATQAYVADSDDDESKSEVYDTENPIAIVADTQSPK

ESQKSNHDKPEDLPKETNKPKLNSSRSSPSQSACGESEGFKTKSSSEMPVNTKPEEKVDYDLFFMSTYKN

DIEISDEELSPASKSPMKEPVTITDQGTLPATEAPVDQDPNESLSSANKSPESKLSSTINKSTVSSPVMD

TVPVGSFQKLASPVDKTVKAPSNTSTPMKETVPLSSTLMETVEVVECVSPEKQPKVAMPLETVAIETFSE

TLRVQEDKVDTSSPAKNRTTITSRNLSLVSSPKAHALTECSENFSLDATQPYALDSSISSPNTKKLSAST

PKTPDKTQPLRQSCGLDIEVTNDSVGVMKDSTPVKNKQSESPVQTPKRSRAHSISSDEESLTDEPNFKDK

PILEGDSNNQSKDLQENIPSSVNTLSGDIFSSGKSSQTTSSSTPDVTETKYSGESNAASLVPAVEEQTDL

ETKASEKSNVIVVSTSKATDEELPVELEDNETISDIEEDIKRSKRLKKDATSTPVSQEGKRLLRNTRKNT

KSTDTPSELIEIPELQSGGKRSLRNHPETSDSHSKDKKSKVRKSIRFDLPDEESSSEEENYLDKNVKRSS

NRSKKSSADIKDDKANKKLSKGSDSKLADLKNEECSEDSKTKMKSKTGRGKRTKMVKSLIESEMEADEDF

VEKKSLKSNEITVEETENISLEVKEENVGDISVLSENTVDSRKSGRGLRGKSKQNKEKAREEISDFSNTT

KTYLHVDIVSEDSKVTRKSGRALRSKSVKEVKEPPDGSALETSNNNTPSSRGRSTRGAKKEVAEDKAQSK

KGGKVSSKKSLDSNKQIEEEPCQKSSNEETKSVNQKEDMEVDKDDDSKTKSNIEEENTEEKKNSQSRTPR

NKRKKSDEDVDSKISERNSKKKKIASNEETGSSQNTPCSLKQKSVSASKDHAENLNLSSDVSADTTAPSK

TTPAKRTLSKRQLSHLQDTKEDASEMQEMKTPAKRRENQRTASQSSNESSNMATPKNLKSNQFISPRRNN

GQLKPNVMFTGLTDDTGQRIVTELGGIVGTNFNCCTHLITDKVRRTVKFLCCLARGIPIVSLSWLTKCKS

AKTFIDYTPYILSDSDAEKQYNFSLSQSLELARSSSPLKGYKIHVTKSVKPNAEQMKEIIESCKAKFLAK

LPTEFKEETIVISCEDDKSSCKKAIQAGIPIVEAEFLLTGILQQDLSIDKYQIFQTSPTSEKCKQPAPTE

KKSRTTRKTKK

>Octopusvulgaris

MDLDITQAITFSDVEDDAEKIKLPVAHLTILNHQTLKNKTFPIFRGKNIIGRHEHSCEVC

IPVKSLSKKHACIEVAGRSHLLYDMSSRNKSRRGTLFLTPEVRYELKHKDTLIFADVSCQ

YAIEEIARASDSGSDTESMSDLEDNLDEISTNTPTMLKSNISLQLEDSNSSSSSDILLPT

QDFPVHELTKHSSQVLADMSPEKAEVLAVDSSPEIQGESSYLKDRIFFQNNANKEVNIDA

EILAAATQAYVADSDDDDDESKSEVYDAENPIAFVADTQSPLQSQKSNHMPEDLPKEPNK

PKLNSSRSSPSQSACGDSEGFKLKSSSEIPVNTKPEEKVDYDLFFMSTYKNDIEISDEEL

PPASQSPVKKPVTSTAQGTLPAILQMSEAPVNQDANESLNSANKSPESKLSSTINKSTVS

SPVMDTVPVGSFQKLASPVDKTDKAPSNTSTPVKETVPLSSTLMETVEVVEHVECVSPKK

QPQVAEPLETVAIETFSKTLCVQDDEADTSSPAKNHSTITNRNLSLVSSPKVHALTECSE

NFSLDATQPYALDSSISSPNIKKLSASTPKTSVGDKTQPLQQSCGLDIEVANDSVGVMKD

STPVKTKQSESPVETPKRSRAHSISSDEESVTDEPNIKDKPILEGDSNDQPKDLQENIPS

SVNTLSGDIFASSKSSQTASSSTPNVTGTKYSGESNTASLESLVPAVEEQTDLETKASEK

SNESQVTVVSTSKAAEEELAVGLEDNETIYDIEEDIIKRSKRLKKDDTPTPASQEGKRLP

RNTRKSTKSTDTPSELVEIPEVQSSGKRSLRNHPESIDSHSKDKKSKVRKSIRFDLPDEE

DSSEEENYSVRSVKRSSNRSKKSSADIKDVKANKKLSKGSDSKLADVKNEECSEDSKMEV

KSKTGRGRRTKMVKSAIESEMEADEDFVEKKSLRTKEITVEETENISLEVKEENIGDISV

LSENADDSRKSGRGLRGKSKQNKEKTQEEISDSLNTSKTNQHVDTVSEDSKVTRKSGRAS

RSKSVKEEKEPPDGSALETSNNNTPSSRGRSTRGAKKEVAEVKSKKGGRVSSKKSLDSNK

QIEEEPCQKSSSEETKSVNQKEDMEVDEEDDSKTKSNIEEENTEEKKNSQSRTPRNKRKK

SEEDGDSKTSGKNSKKKKIASNEETESSQNSSCSLKQENVSASKDHAENLNLSSDVSADT

TTPSKTTPAKRTLSKRQLSHLQDTKEETSEIQEMKTPAKRRDNERTASQSNNESSNMATP

KHLKSNQFISPRRSNGQLKPNVMFTGLTDDKGQRIVTELGGIVGTNFNYCTHLITDKVRR

TVKFLCCLARGIPIISLSWLIKCKSAKTFIDYAPYILSDSDAEKQYSFSLSQSLELARSL

SPLKGYKIHVTKSVKPSAEQMKEIIESCKAKFLVKLPTEFKEETIVISCEDDKSSCEKAI

QAGIPIVEAEFLLTGILQQDLTIDKYQIFHTSSTSEKFKQPAPTEKKSRTTRKSKK

>Mytiluscalifornianus mollusca

MADDQDFDQTQALFLDEFEQEIDEDNVSDNKNSVAFLKVSCQKSFPEKSFPLYEGNNVIGRHEESCNVCI

SLKGLSREHACIEIKGESFLIYDKNSRNKTKRNQLYLSPDVRYELKNGDTLMFGDVTCKFYIGNKDVDAV

SETGSESEYQTANDVKDVTVEFEEDYDSDNSVDLLQPTQAYTNHTQRHNATKSLLADDSFVEKETTVLVG

ETPAPARQKVTSGPVIDDSGSETEDEDALSKNVPQTLVIEDTQKDESGDAEILYAQTQAYVGESDVGDVT

DEEDKKLLTEPTQAFTDVQDRQQIEPTQTFVAESDEEEEDDRKKALQYGATQAYHTDYFDEPTQTTEDEG

ETVVESDEDVSHLFATSTLACDVDDFPEEGGRQVITADDTVDQEMETQAILDEPTQAVDGGDEATQVFPD

VRVNGDDATQVVKEVACPSKKVAFDDATVAVQEDATVAVQEEATVIVQDDATVAVQEDATVAVHEDATLA

VQDDATVAVHGDATVNIHGDATVDVHGDATLALPEATVSVHGDATVAFHGDATLSVHGDATLALQGDSTV

AIQEEATLAIQGDTVAVAEDATTSIQDAATVAIQDDTSDAVQDDATLAVPPDTTDIVEDDHHLGAQTKGK

GRGRPKRSETASIKSQDNIDKAATSKGKRKAPVKQTAKAETDMKREDSVTSSQESDDGNIRRSSRSKRKS

FRMLEMEQSMDDMKGIDKSIRKGEDYSPEISDTDDGSPKRGHKPTKTAGQSQEKDIGEAETLTLSVEEEI

ALVQKNIPPLPKETVKKTEAEFLMSLPALQDKESPAPVLTEATQAYTLEPEEELPQPEMVEATQPYVLSE

DDSTPLLDEGGSKEERDTTPPIEEVLAATQNYSLEEEVVADSEETDIRSPLISIPSRSPLKPTLVSPDKR

EKKSPSPKRVQFTVKKIDSSSTSASENNDIPTDSETAEKTEQKNTSTNRPSRSKVKEAIIKKVRNKRTAE

AKSHPASQGENENLTEGKKGRQSVLSEQETNKTETKTTRRSAITDIKTEEHINSKADQSANDSGETTKIT

RGRRSTALIENVKSEDTASTSKGRGRKSTATRKETNKGVKKEDKTETVALISDGENEDLVKPANGGSQGI

STEQVDKTGEPETLKTFGSSGKFSVQELSEPGTSHSETSLAKQDEKIIENKPETKRSRGRKVKASIDPVV

DNEKETVIENVDIDKRVSKTIKGGRRGRSSVVPEMTDKARSEAEEFKASKSAGRRGRSVAESESNTTTNN

DTGKTDDVITKQTGRRGRTSVLGESKNMSETESQESYAGSKTTKQTGRRGRTSVLRESESKNMSETESQE

SDAGSNTKRAGRRGSGRTSVSGESESRNSCETESRASEAVSKTTKQTGRRGRTSVLLEESEGKNSCKTES

QESEAESKRGKSKALGSTSEAQNKQLEVSKDDNRGRRGRSSVLSGTENKDELMLPPKSGGRRGKKNADDS

ATSEIQNNRGTSRQSKVNEFINENEKEKSFNLDIHEKDKSESSDSNTRGRSSSRTRKIENKEIVKENYSK

PTNIKRKRDSSINVSDVSPSTSKRAKIAVNTNVLETKTTQKSKGRTSNVRNDSPVEEDTSRSSSRTSTHK

SDHSSVSSEHSKEQKTMKSESKKRGRSNDENVSETPAKKTRVKEEQSTPKQNKTLAVSSPSLRRKSVEPN

KPKVMFTGVVDEHGQKIIKDLGGEFVNSVQECTHLVTDKVRRTVKFLCCLARGIPIVTLQWIESCKQSAM

FVDCHKFPVKDTATEKQYKFSLSRSLENAKESCLLQGYKVHVTKSVKPEPSQMEEIIECAGGQYLTTMPK

KGGDNIVIVSCPDDESLCTAVMKAGVPVVNSEFILTGILRQEVNINSYPF

>Ostreaedulis ostreida

MSDLEQTQAIYLEDDDEETDEESTEGKKKIVSFLKVLKQEGYPEKLFPLYDGDNVIGRQEDACNIAIPLK

ALSRQHACIEIRGENHLIYDKGSRNKTKKGKLFLTPQVRYELKDKDTLLFGDVQCVYIVGEPDNLDDSET

GSESGFQTAPTDPVENRGTSHVTTLVYDSEEDKYNSDDSVDLLQPTQAFMPKQDESKRSRINFDMDTDDE

SERKSPDITVKETPAPRKMQGVLSTVAVLPESGSETDDEKSSPSRKKDGQSNKSGNSRLFQAPTQAFLVD

SEGETTEEDSPKKRLLFAATQPFLEEKSKQTGSPQGVVLESDNQEDQDENIPVVEVEEEEDEEDKSHLFA

NPTLACDLPEDIVESSDEQEAEEENNASTEDPEAETQVFTDSTVAVSDETLDVSAVKGASKTKAGKKEEK

VAKGASTVEIKIKEGENDATQEFSDITGGATLAVGNEGECESTQVFDNVTEAETVAVQCEATQVFDEKVT

EAETVAVECEATQVFDEKVTEAETVAVECEATQVFDEKVTEAETVAIECEATQVFDEKVTEAETVAIECE

ATQVFDEKVTEAETVAIECEATQVFDDNMAAEVETMAIDNVADFEKEAETVAVECEATQMFVKKTTKAVK

EKSKSTRVFDEKISVAETIETECEATQVLDNVSSNTSSRKECSDSSVETVALECEPTQVFDDVDVGEDNR

TESNKSSTAASKTDAKDKDEQKQCFDESDVKNRDKAGNSLTKSKPKCREGEGSGEVLPKRSGRISKRKSA

VEDKPVEKIQETATKKGRERRGRGRKKTRDATEELIGSGEEATETKGDKTSTGLDEIDSRVINTKSDRSE

AGIEQTVQYNMEGIEQTVQYNMECDEDLQPTQAYCMEADEEESDTLSISEVLEVEDRTPTEEVKIMEDTP

DILPVPIPPASPHRSAMASPGRRSPSPKKVHFEKRESEVTIDEASGSKLKPKDEISAGKSSGRNRRSLPN

LSQPTAPARGRRGRLSENLTIKAEALSSVAVTSKGRRGAKTQKTEMPETAGKGKAAKVISEEDTPPLEEI

ENVNTQKLEENPANEEETTQVMGRGKRGKRKHHDAQPHTDVSTSAQSDEGEQEKKMAEEAGEVNSRKGKV

GRRSQVPVSDEQGMENSEHHEESSSRGRGRRKTKILDDEKEQSLSDTKTAKPGKGKRKIIENSDGEGSEK

TDSDNGGRKSLKSRRRGLVEEINEPAVEEIQTDEPPAELETYKRTKGRKSLSNTETESVPAGRKVRTRKS

DTTAISKDEEVVARQTSSENVVEDLDLEESKTNQKGSKRSRKKETLNIISEETVKTSGELPSTSSHNTRT

KQRTSSECSEEINEVKNSVENKNETTPSRKGRGKGKIKQTKDDVQENEISDNNSNKKVQRNQRKLVTKQA

QDNVAETGGSQESVVSTESIPTRRGRGKTAAKQMVDSQDIQEDSGSKRKSRGRDVSAGRRSASGSQSRES

SAGRRSAAESQSRESSVTRDSETDKIISSAKTKGSNKKVKRDQKNTTPVASDTEMSSDSQDSTKPRGASK

RGRGRHATEEAKEEVPVKKSKMEPPSTPQQAKKSAKSTDSPSSALRRKSMDPSKPKVMFTGVTDEQGQKV

VKDLGGQLVDAVQECTHLVTDKVRRTVKFLCCLARGIPIVNPLWLDSCKSSGMFGDHAPFLIRDEAAQRQ

YKFVLQKSLEKASEASLLSGYQIHVTKSVKPDPANMKDIITCAGAEYLATLPKKAGDKVVVISCPDDKGA

CDAAVKAGVTIVNAEFILTGILRQEVAIENYILFQDKKRSRDSSAGGQPSKRRR

>Mytilus coruscus

MKSFILVVTTGPVIDDSGSETEDEDALSKNVPQTLVIEDTQKDESGDAEILYAQTQAYVGESDVGYDTDE

EDKKLLTEPTQAFTDVQDRQQIEPTQTFVAESDEEEEDDRKKALQYGATQAYHTDDFDEPTQTTEDEGGT

VVESDEDVSHLFATSTLACDVDDFPEEDGRQVITADDTVDQEIETQAILDEPTQAVDGGDEATQVFPDVR

VNGDDATQVVKEVASPSKKVAFDDATVAVQEDATVAVLEEATVAVQDDATVAVQEDATVAVHEDATLAVQ

EDATVAVHGDATVNIHGDTTVDVHGDATLALPEATVSVHGDATVDFHGDATLLVHGDATLALQGDSTVAI

QEEATLAIQDDTVAVAEDATSSIHDAATVAIQNDTSDAVQDDATLAVPPDTTDIVEDYHHLGAQTKGKGR

GRPKRSETASIKSQDNIDKAATSKGNRKAPVKQTAKAETDMKREDSVTSSQESDDGNIRRSSRSKRKSFR

MLEMEQSMDDMKGIDKSIRKGEDYSPEISDTDDGSPKRGHKPTKTAGQSPEKDIGEAETLTLSVEEEIAL

VQKNIPPLPKETVKKTEAEFLMSLPALQDKESPAPVLTEATQAYTLEPEEELPQPEMVEATQPYVLSEDD

STPPLDEGGSKEERDTTPPIEEVLAATQNYSLEEEVVADSEETDTRSPLISIPSRSPLKPTLVSPDKREK

KSPSPKRVQFTVKKIDSSSTSASENNDIPTDNETAEKTEQNDTSASRPSRSKVKEAIIKKVRNKRTAEAE

SHPASQGENENLTERKKGRQSVLSEQETNKTETKTTRSRKSAITDINQKTEEHINSKADQSANDSGETTK

TTRGRQSTALIENVKSEDTASTSKGRGRKSTATGKETNKGVKKEDKTETVALISDGENEDLVKPANGGSQ

GVTTEKVDKTGEPETLKTFGSSGRFSVQELSEPGTSHSETNLAKQDEKIIENKPETKRSRGRKVKASIDP

VVDNEKETVIENEDGDMEKTVDFDKSVSKTTKAGRRGRSSVVPEMTGNARSESEEFKATKPAGRRGRSVA

ESESNTTTNNDTGKIDDGKTKQTGRRGRTSVLGESKNMSETESQESEAGSKTKQTGRRGRTSVLGESKKM

SETESHESEAGSKTKQTGRRGRTSVLGDSKNMSETESQEIEAGSKTKQTGRRGRTSVLGESESKNMSETE

SWASDAGSKTTKQTGRRGRTSVLGESDQSKNSCETECQASDAGSNTKRAGRRGRTSVLLEESEVKNSCKT

ESQESEAESKRGKSKALGSTSEAQNNQLEVSKHDNRGRRGRSSVLSGTENKDELMLPPKSVGRRGKKNAD

DSATSEIQNNRGTSRQSKVNEFINENEKDESSEKEKSFNLDIHEKDKSESSDSNIRGRSSSRTRKIENEE

IVKENYSKPTNIKRKRDSSINASEVSPSTSKRAKTAVNTIVLETKTTQRSKGRTSNVKNESPNKDDTSSS

RTSTHKSDESSVSSEHSKEQKTIKSESKKRGRSNDENVSETPAKKSRVKEQSTPKQNKTLAVNSPSLRRK

SVEPNKPKVMFTGVVDEHGQKIIKELGGEFVNSVQECTHLVTDKVRRTVKFLCCLARGIPIVTLQWIESC

KQSAMFVDCHKFLVKDTATEKQYKFSLSRSLEKAKESCLLQGYKVHVTKSVKPEPSQMEEIIECAGGQYL

TTMPKKGGDNVVIVSCPDDESLCTAAIKAGVPVVNSEFILTGILRQEVNINSYTLFKDQTDSHGATSKRK

>Biomphalaria glabrata

MDSSDLDQTQAIQLPFDDDDDLTDELESDVEDGGTKKPVSFLKVVLQCGLQETSYPLYEGDNIIGRSSDT

CHICISSKSLSKEHACIQIVNDTHMIFDKRSRNKTRRGKLLLLPDVRYELKDQDEIYFADVKCIYLLPSE

ESPAKSLDHSDVLVANTPEAEKKSVVHTLRVSDSESEPEPELDRIKLRIEETVLCEDSDHDSNDQDVYYG

ATQRYPNVVAKGQNSDDEDSNLSETFPLQQKEDTKLNNSSEEKSETKTSDSGDINFEDDRGRREDVNPSL

FSVSTLACDTTVTTNQRAGSQFAKDGSSVESVDSETLKVPKMVDSADTSPSSESSTTSNKRLRKRKTQNK

TDETLVDSTLVLVDNLTTETHEVSAKMLRMSPGRRGRPRGAKQNRGSQRKQKECLTMMDEDFSNEATTQM

LQTSLMEATQDFRGAAEKIQSSDLKPISGSQLGDDTEDFSEASEDRQADDLNPDGESRITLMDDTQDFRG

IAEKQQQADLKADGSSQPFTVMDETQECDKSTENESGDEEFGTEELTQAYALGCDSSPAEESALDETQKY

DMIENDENLAEVNERESHQDKQDIEEAVEEDNSYDDSDVEAESVLKTDIVCQDNQLSDESAGDNTTLQLD

DDNNNEDGTKLFNTKEQNENLQDTICESPVKSTEEVSDPPVSSLAKSPHKSALATKRSPRHSPTPKRVAF

TEDALAVKGHDVNSNSGSPSEKRSSEQTLIITSEIISQVAEEKIDIHPVGDMKATSKNEFGSLQNELPES

VPDVAPLQPENSPAVKRRRGRPSTKNPPEKKLVSVVQETEINPAQETQIGRRRSRKSQPGGSASVVTETS

AEDIMNNSCLTGENTLVKQKNVKVTEAASEEHNSDVGNKDLHSNVNVASSSTDEATPVSTRRKRVSTHPA

YLKDFETVQSKVRKSNSEENSLDGSNSIKSIGRKSARAVRSSLIKVQPSDVDLNNSTSNGSIKTQPEPEP

INTDKKSNDFVETSSTSNKNQDNSLQAASTPTESFNEVVETLNSLKKTLNVESASAAHQNSEDLVEQCNN

VNAKPETENLKITNSLESGDIASTSPTNSEESVEEASTKRVRGRASRRQSKSHSDTANNVPAAVKSSTRA

QKKTKETIKSTDEAGNNKNVAAESSSKGGQIGRNTREATHSNNSSDRQKPNQQESRAQKKTVRTSKTKNE

VEIAVSEADSSGNDREQGESTKGTNTSTEEHKLKQSSKRAPKKNKEPNKKKVESVELAEGSNSDGAAVVD

METTEVTPLNTPASDTQKSKRTSARSSTAVQSSVQETGSQSSDQGTKRRTRKSEIVPAQSTSDVDQQADG

HFSKNGEAASKANKKATSGSSKKVTPPSSQSSTEEKTTSFSPSLRKSSLCPKPKVMFTGLVDEPGEKIVK

DLGGEIATAIQDCTHLVTDKVRRTVKFLSGLSRGIPIVSPHWLENSKRAGTFLDCHKFLVSDSAMEKQYK

FTLSSSITKAASVALLKGYKIHVTKSVKPNPEQMQEILVCAGAAYLKSMPNKVADKTVVISCPEDKKLCQ

PAIKCGIPIVEAEFLLTGILRQELDAKQFALFPEDNQPTEKRKKK

>Crassostrea virginica ostreida

MSDLEQTQAIHLEDYDEETDEEVGNGEKKIVSFLKVLKQDEFPEKLYPLYDGDNVIGRQENTCSVVIPLK

ALSREHACIEIHGENHLIYDKGSRNKTKRGKFYLIPEVRYELKDKDALLFGDVNCVYIIGEPENTVDSET

GSESGFQTAPTDPIGSGTGKSPQVTTLVYESEEDQYDSDNSVDLLQPTQAYVQRQDGNKKSRINFDMDTD

EDESDKKSPDITVKETPAPRKTKGFRSAAVILPESGSETEEEDGGKQLEKKSLLLAPTQAFLAESEGETT

EEDSPKKKILCAETQSFLEDKGKKSRSPHGIILESENEEDKDGDVPVVEIEEEEEDSEDKSHLFANPTLA

CDLPEEEEEYGEDQDTEKESHDTEEDPEAETQAVNDQTVAVTDETLDVSAVKGKSNVRVAVDKKKQRNAK

DALQVENAANAATQKFSDGTDGDTLAVDKNECDATQLFDNKVTEAETVAMECEATLALNKKVSDMNALKS

GKKATEAETVEFEYEATQDLDDKAAAAETVAMESEATQAFSRKIVEAETVPVECDATQILEEKMTEAETA

ALECEATQVLDENISEVETVPVECDATQVLDEKGAELGTTSFEVELDVKSAQDEDMVVDDSSTNVKQAAT

VVVEAEADVKIDNAETVPVGCDETQVLEDLTKESKDSQLKIVAVECEPTQILDDTQGVGDKSTDEVVKKR

GRGKKDTSKTETKPNVPEDHEEVQPKRSGRLSKRKSLQSEDKPIEKTQEASKKGRGKRGKGGNKVEDIAE

ETDEDRENTTEKESDGEKKTTEDEKECITEKTTDAMDGTNSKLKNLSPEKGTNVKDSTGPMTTVSDEELE

PTQAYCMEIDGTNVKDSTGPMTKVSDEEIEPTQAYCMEIDDQEESNTPPISEVLGVEVEDGTPKEPVKFM

DSEPDILPIPIPPSSPHRSALASPHRKSPTPKKVHFEKRESEFVLNEKTGSKLKIEIENKVPMEKAGGRT

RRSLPNLSQPASPSKGRRGRSSVDQMLKVEDMDEPTKAKGRRGRAKAQKPSNEESTKKGGRENHWREVNE

EETPPLEETENASEETQAIDKESTSKVEQETVKEAVEIKGKGKRGRTKAQKPCNEETTKKGGRGNHLSGV

IEEETPPLEEIQAFEKESIPERVSIGEHETVKEAVEIKGKGKRGKRGSKAPAVVDKDPSGVDNEQSEEIN

NQRPGTSQKKDKEKVEEPTKLEGKVVKSGRGKRKKNETSEAEEIDKNKFDDFEMDNKQSKTTKNKDSGKE

LKDSLVEESVADEEDSSKTQSTKRTRGRKSILKPENLPSGKRGGRRSKVEEKTEEEDKHTSNENFEEESE

REKTEENKTSSRKSRKRDSLVVVNEETLTDSQQSSSSSRNKKSIRGTPEEDELKDMSEELQDDDKVASES

ESKPVRKGRSKMMEEVSDVADSGKSQPKGKRKSVTKHRPDNEDNSEQSQENVLSTETTIARRGSGKAHPK

QTSQSQDSETNSGPTRKSRARESSVTKKAEEESESQELTEQKDSKTVKSSSKSRKSNVKDTSAVDLDTEM

SSDSQDNSQPKAAKRGRGRHKTDEAKDEVSAKKTKMEPPSTPQHAKKSTKYTDSPSASLRRTSIDPSKPK

VMFTGVTDEQGQKVVKDLGGHLVDAVHDCTHLVTDKVRRTVKFLCCLARGIPIVNPLWLDSCKSSGMFVD

HTPFLIKDEAAERQHKFVLHLSLEKASESSLLSGYKIHVTKSVKPDPANMKDIITCAGGEYLTTMPKKAG

DKVLVISCPDDKAGCDSAIKAGITIVNAEFILTGILRQENAAENYILFEDKKRSRDSSVGGPPNKKRR

>Sepia pharaonis

MNGDWSQLASLEKCWQQTDKMDFDMTQVVTHSDTEGEDEENEKSAPVAHIKVLNQPSLKK

KLYPIHKGDNTVGRHDLCQVSIPVRSLSKKHACIEVRGRSHFIYDMGSRNKTRRGTHYLT

PEVRYELRNNDMVVFADVSCIYLVEDSARASDSGSETDPMSDSETVFHTVAVDMYNSVEP

TVNRVVEPELIDVAVTSDPNSCDSAASSDILMPTQDFAVMKIKQESQNQKDAVPSNESPV

IKNASREAIKFLVVDSDSENEQENLNTKETLHFQNNGNNEDSLDMELLAAPTQICVADSE

DDDNKSEILDMVQDYKTTDNSFFVADTQSDDSPKPPITDSEEPTDGKTDRNGSASPTSPF

RTSFVGSPRTYSKKDACNKSRPELMPSKSINTAQKDEEKPVEEDCEETQDPNLFALSTLV

PDENILRCPTSPDSKSPPASPNSNKNTTLNTTNDETETVTADEKDTLPVSGEGATVPVNS

EEAETVPVTNDTATNNTADTQMVSDQETSVAVSDTIPVTSQNAISFLANKDSDNSIILDN

KTPLLVSGQDLATVEITTDETMVVALDIDVRDSETVEVDQLQEDSSQQKQSKEDNSGAEP

PLKLEEESDMNESKRHPETCTTSTPSKEPSSPFQLLSDIENCTKAQKEPVLKNIATPVKG

NPLNADLLSDVSVGIFDVHVSVPLPVTPSAKGKSPKIPMRQSDDEILEATQPYMIDCLDS

PPVQKKNVKAKRNLPFDMGITEESDANNEDETQPYMLEDNKQDVESHSRASFRRGADHEI

SDDEEGVSVKASPKEKSLKNVTSYQESDTLPLLAENESDTDKIVSESEGSQTSRSTAGRN

GSKKVQAVKDLKEETVTSLKTGQETQNVELTKTEELQSEEKFLNAPKECSSLVNAKMTET

SKLSKILQSKEASPSVSIAQTILGEKQPTRRNSKDSEESGEAAKLKPNKRGRKRKTAEEK

ESADKVALPAEHVGAEIEVEEEEIGSKTKKTRRSVHFVGISSSDEYSQESTETRSSRSRR

KESQETENETVGRKTRGRTSNAREKGKKGTISKKTDVTETEESSSKGRRSRGPPAGRGQR

SEKKAVSQEKALSPPPQKSSNVSSDSFESSESMDLAKQEPKTPTARKRNQSSQSSSQSSP

FTTPKRQQLNSSLSPNTPSSRSLSSGSKPKVMFTGVTDDKLQKIVTDLGGSVVTGFDSCT

HLVTDKIRRTVKFLCCLARGIPIVSPSWLANCKTAKMFTDHTTHLVSDSNTEKQYKFSLK

NSLQHARTGPLLENYKIHVTKSVKPNVDQMKEIMDSCKAQFLKRMPSEEAENTIVISCEE

DRTLCQPALDAGIPIVNAEFILTGILQYSISVSKYQIFDSSSGSKRKLVATDKGNAKRRR

R

>Mizuhopecten yessoensis

MDLEQTQALDFGGDFDDEDETDDEEAVKRPVAHLKVKSQKEFEEKVFEINEGDNVVGRHE

ITCSIWIPLKALSREHACIEVKGDSHFIYDKGSRNKTRRGKAVLKPDVRYELTNNTSVTF

GDVEAVYYIGEEINDKGSETGSESFQTAPMEDEGMNGKEGAAGVTLLVTEEEEEDSDASV

DLLQPTQAYHGGRTLNLAPDSDEEEGPQHTPNVTVQDTPLPPKRTGAVGATAMSTLVLPE

SDTDDEEEDRRKARALQSLQTQVVTDTQQDDDEEEEEEGEENNNVNPAMFGATQAYVMES

EADDDATDDENTRNVSPAIFAAETQAFGEESDDKKDDDQPIMTFVADSDEDSDVENGEAV

MDDDTEGEDDNVDVSHIFAASTLACDMVDEEEEQASVDLADNDDNVATQMVEEDATEVVE

ATKKIAEDEEEGTQVIEEEGDSENITTQCLEEATVAITEVNPIAVTTGDATVAVQEGETM

AITDTETFSVKNDATVAVTEDATMAVTEDATMAVTDDPTMAVTEDPTMFVTEDATMAVTE

DATMAVTDDPTMAVTEDPTVAVTDDPTMAVTEDPTVAVTEDATMAVTEDDTMAVTEDATM

AVTEDATMTVTEDVTMAVTEDATMAVEAREDVTEATMAVNEEAATQVVDNVADFENDATQ

VIEDESEHEKHNHKKKSGRGRQGRKSKTNDGIEEDATQVLDDATAGDYGATLVAEDINNP

QETPKENISRRGRRGKAGKVPEGDTNKTESDTTEDEATQVIDDDVSIESEVQEKVSNKPK

TGVGKGRKSKAREVFEEEATQVVEDPDLGEDQATQVIEDVSADTPKESTGTVKGRRGRPG

KRGKKKEITEDEATQVLEDESTEVPEEHKPSSRLGRSKEGETTKGKKTAEETDVSGGEEA

TQVYGLEVDEEELAQPRGRSGRVKKSSPRRGTDRVQKDLVNLPALKPSREETDPAACMDV

EPATQMYGEDSDEEDTPPLSEEVVQDSENVLGKSPIIPLPPNSPHKSALASPRKRSKSPS

PKRVKFAIESQSSESTVGEEEEKAGPSTGHTATRSKLTQAGVKVKTTVTRGRRSLPATAV

KEKKEIASRLSKGRRSLPASDLDNVSSKKKGKKEKMEKEDIKEDEYKEPEVVVEPTTAGK

RGGRRSVGVKVNEEPNVKGKGRNSATVEKEEMNVTENKGTERGKHSTKFESEKDAEEKEN

EAGQSTVDSTESKVEASVEEVRGRGRGRRSIQAGGSGDCSTIEKNKEKTTKLVRIVGKRK

QAVAEEPASNESTPDVLVQAEDSPSVKEEEVKGRTSSRGRKSKQAAEESKSTEKAQENQP

ISEVVEKGKKTSRGRRSTQLAEEETKEEEVVEEKNVPAGGRGKKSKLKPLEEEDTPKSAE

EEGEEFISNSKHECKNDKEVKDTSEDKLGGPVASTSRGRRSKQVSNISDSVEPIEEKTGR

TAGSRSKKREASHEEMSPTEVVSEQENRRRTGRGSIQGRKSKQVVEEKEDETVKETAEVT

EPPKPRRGTRGKKATEPFQEVSAENEDVSAADLANMSGISKRKPRGRVKKIEEVMSVDSK

IEKRTSRGKGNQTVNVSMDSEKTEEESQSEVSASRQTKAKGANKTKEENVSKGRSKTQED

SVAKGKTSKEENTQEESELEQSKIRTKGRGKASNSKEDVDVTKGNEESDSGDVRKDRGRK

ANTSVSVSDQEGADPGTSRKGKTATRGKRGHSQEQDVVETPAKKIRDETDLGTPVQSKKD

VLAVDSPTLRRKSMDPKPRVMFTGMVNEQGQKTVKDLGGEMVTSVQTCTHLVTDKVRRTV

KLLCCLSRGIPIVLPTWLSSSKQTGTFIQDVTPYLVKDTATEKQYKFSLQRSIERASQAS

VLLDYNIHVTKSVRPDPTQMKDIVECAGAVYLESMPCKFAEKTVVVSCEEDQKLCQVAMK

ADIPVVNAEFVLTGILQQDANPSNYQIFNNSKKRPADSSAEGPSKRRRR

>Phallusia mammillata

MDFPCTQKLDDDLDETFAEEEGKSERKRVGTIVVQDASKQNISSIEFPIYEGDNIIGRHGNCDISIPEGV

ALSKKHACIEATVSMAFVYDCGSSNKTRLKSMRLKPEVRYAIADGDELCFANIKCTFRQFKEEDKKQSPH

NIFESVPETPFANKTGEPLAVDTPASELSAGKRNDSHQVDDSVFEYSEIVPDSQPCSDQMDVNDENPSEV

VDESLLELNANHDEGSYLHKPSLPELSQLSQEPLMQSKTRKKLVIESDSDTTDIEENDSEDLKQKSSSVQ

EADQHYDSDTDVEESSPVIDPYDEETDVEEDRANISKLDLDLQRPSGSKPTVKFVDQKPDAIPVTVDSDD

TDIHDSINDSFSILETQAYFSKPNPTGAKQSETNASLANDSFAELETQAFASPKKTSCVIGEGIKSPVTE

KDLVTKLNNGVSPAKNGLTTDDSFAELETQAFMAKSTHLELNKSNASDNLADESFSALETQAYVTKKTEC

ENQTKMLDDSVYDAETQAYDPDMSINEVPDAKTENPAVNVSSDESDFEDSLLASMQTQKYETLKPSLTTE

NNKTVTATTIQTDLDLPSSHEFFASSLDSPVDSVVCEDTEPILPSQSESSVTYTSDLVDTPEFAYSLESE

NDHTLPVIAESQSFQLSLKSQLQSTLEDTQPVNDDKIQYTKTSTLQETLENTVTCINEDVTIGVIPDNEE

LNVGKNENIIERDVLAHRETDDNVKAVRETTEDNGVISDAESKSSASSKAIDNTCTSVSFNDATPTLDVT

KTITDNKPDDCKPSEPTKKGNHILNENEKDVNTVNKAKCKDEALPDELDKTTNIKIHTDNVAANGKNSDE

NNMEICLNEEKLDCTDNRTIVVSKSSVEISDDMETQVYSLESDDDGQINAERKLPTKGSTPIVLIRSEFA

VDTEGKSGETELRNPEEKSAELCLNEPTVLEDDNSLTQVYSLPSFAENAQTTLVENLDDDIMQTQVYSEE

SLANTKTVSGDTFNQVSRDVSAKVDSNNNETGTLHPKPARRSVRARKSSKRLSESKMEPSPLTTRSRRKN

KTVSVASESVQQKTELKNNIARKSKDASTSKNRSTRTKKQKKSTAAKQIKKEVDIEFGECSINPQPELVK

IQFQPDSVKAESPQEDKKPAINREKRSRKRKKIMNDSVNESDSSTTAKKIKNESDENDIKRSASLSLRPR

RSKPKIMFTGVVDKIGESIILSLGGSLTDDMHECTHLVTDKIRRTVKFLCAMVRGIHIVSPLWLRESNGN

GRFVGEEDFQLHHDPNKSVDDSPPVDLESQYNFNLSSSLCTSRSRKEPLFFGFSIHVMKSVLPPPDHMHQ

ILVCGGAEVVKKLPRARDEEQWKTLVVIATEKDEKACVTALKHGAKVVSNEFVLTGILRQELELDKFSLF

ADCNAATTPPSGEGDETKMSTRGRSKATTSKKPVAKRKRKT

>Styela clava

MISILPKYLNFLDLKIVEYFLLNIMESDFLCTQAIDDDLDETHDDESQSSDEDARKEVATLKVLPNKKNG

IPAIEFFIYNEENVVGRHTNCNISIPWSIALSKQHAVIEAVSDTCFVSDCGSSNNTRLNKVKLTPNVKYG

LRDGDQLVFGDVQCTFVQKRDESTAESASGEPFTTHATTSKLPPTSSTPASNKTRILATDTPENLTYTSI

HAKLDESSATIPESQPICADTPSTVNHRFQESVNGKATASTSSPLPIFNLDETIEKAQSNDGQITTSGGA

DLPANVSHVPASDASDDETDYHEDSDTDIEDNATIAAFTNQKGDLVSEDVYEADTIELTSGRSSQVSADT

NDKNKDSVKLNIDNISDSDTEDEEMSMIKDLDTLQTQMFSIPSNKNKTSPCPEIDIPGPSSKAENPAGST

HSSDTDDEAILSVNLDSLASEGMEEEAGGVKDVEQDNNESNDDSSSPVMRDAFDDLCTQGFSGGASRNME

DTLGMMETQGFGANLVSEEDEDAEDEGMVDSMIFNMATQAFPGCMKSPKSATKLSAKERDVPKQEHMPDV

GDSIIQDLGESTIPLEEDENMEISTKDRLDDSIQPNDSLLLQSSTISADENSGFSAAVIKNEQSGLSEVT

IDTEPFDTIPLVTKEKNKPQNDSDDVTLPLVRSNETTVDASMYTELTLDPTSSVDQADSDGNENLLNRTL

NASSNRESVACTATTNEANQKINKENSYNGDTIVPSTNVNVPDSENDDFLIPKENSKQNNNEGTILPEAT

LQINLPPKKDDSNESEIQSSFHKDQLLEEENIDDQETQVYDFDDLDATENDEIKGVSPSDKIEDNECETQ

VYEMDVDMSENIAASTSSEIGQQGTKSTDLQSSEAEPANVHSNVNLPSNVSKKYTNSDTDHTVFTSDQDI

QGNSRDLSDSVVIDENKNVVKESFHLKRDYKDGSTNVEVENSTSLLIEEPVVDKNLVLHGRKMDSANCHS

QQDNDEAMAATQSYICPSNSVLEKLSSVPIVMNKEECENDITPTQSHAIIQTTSFKSKNLENSEEIEPTQ

RYIKNNPSQKDDITEEKNINLHRIVKQNDQMETQSYVLQPSSYSDDINQESHSELYNVLHAVEDVGTKSA

IKPVTTNNKTKPKISRRQNSRSASSTLTIDAPIEPVRKSRRTSRKDYASIAKGKEDNTETLSSLKEEKKT

KKSLKSDSKKKTEMKATEKLSTSMTVSENTTLTSVEDDSSVNPKTRTTRKSQSKRTESKEKDEVILNNNE

PENTENRKYSQDAIKSKTDSIEQPSPSLPVSENTASTEFTESISFVEPMTRTTRKSRSNTIKSKEKNDVI

LNNNSNEQSSTSSPVSENAAPTEFTESSSFMEPRTRTTKNSRSKINSKRSNEVLLNGNQLESIGGGKFSK

NSELVSIEKPSSSFSISEDAVVTEFTENSRSGNLKTESSKKGNEPNHSRTNENAQVKSSVANISTKSSRK

TRNKVDEIKENINANLPTSSKVIKEKTTRATRIQNKVAGVVSENNIEDENGDIHIEEKEAPGCSNITEKD

AFEAMTTKQTRNTRASKSHHMVDEVPSSSNRRISARAKKKQDDVTVKQERVTSNVTAESTTSSINLPPEP

AASAKPVVGKRKRRKASMSSNSSTSTTSSATRSQRNSAKKVKSEASVDSDLQHAVAMALRPKKLKPKVMF

TGVLDNVAEGIVVSLGGILVTDISECTHLVTDKMRRTIKFLCALTKGLHIVTLEWLRSSSQQQRFLPEED

FELKEDFQLPNGEFLQEKYKFKLGETLKKQERVNSHCFIISTFLLMRNVSPPPDQMHQVLLCAGAKILPR

MPSSGREEITPNTVIIATP

>Stylophora pistillata

MDFDQTQVIDDFGVEEEVSEEENNGRSKKIEVAHLKVFSQQGFKESVFPVFKGDNFIGRDGKCNITIPIK

ALSKKHACIEVQRDLHLLYDCKSKNRTRKGKSLLKPMVRYELKHGDMLTFGDVTCQYLVGMEEEEEDGDE

TGSETGSESMLMDVNVDEELKKTDKSIVSEDVSLSSTIDEHVKGAIPRESLLQATPGHKTKKPSKDDEDA

YAADTDSDTDEERPLVTTAVPTIMFSSEDEKEIPNDQRASSELLSVPIKQDTSMEGQTLAFGLGSPTNVF

LSRDSTGSSGSHSAQTGSIPPTLKISSESDSLSDVSPFRHPGARGSVTQPPCGPTLLYGSESDDGSPIKR

PRHPTVRVADKGPSCEPTLLYGSESDGSPKKKPRLPMASNEPTLLYESDSDESNDKAAKRLRALGDSHQG

NQDQTLLYSDISEGKLPSIPGKSSADGENKDSTDGSTKERPNTEEEMGVVEDTDATLPYCRAEMSSTDDE

GESEGVRTAKRTSVDETPAPILRTLACNTKEIEEDLTDDEQHNRDADDYGADVATQAYAVQSDSELEIES

VSTRDEAVARDLNQVANPSDLEATQAYCIEEGEDSDSSDSQPLPIGIAKTAATDDIQATLAYGIGEPERV

SDSERVDNNDGDHKRGQAQALVYDDLQATLAYVIGGGDDEEGCTETDGEQPSDLDAGVSRGDDVQATTAY

GLEATQAYGAEEIDEEETAVKNRDDIGGYQGRGTGPDANAATLAYDFGSTQPYCGNDNGATEDDKGDNAF

EEHITKMENLATLAYGLEETQAYAFDNNDSESSHNDMIEATQAYGIDGPSAEETGPLAQLYAASTVQGGV

TGIDENKNGVSAVSEVDISKPSSSSAVDVVDDSQDREDVMPRRSRSTRGRKKPVIEDSQEVGSAENYAVS

VVEISNNDKREETKVESTPGSILNRNGKNSRVTLSSRKGRRGAMRVTIDSGMPDEDEGKETLSRGGQSGT

KETNASESQDKETSKVPASTGRKRRRQAGKGKFIEEMEGTTIRKSRRGQKGKGAQMEATFLPETPPMPST

DTTAKEIRESPPDSSAEAVPTRGKGKGRGKGRGRKNEQLVEESVARESIGSEISTISNEPEPPETVSTPA

RGKGRGKGRGIKAQPKIGMDHELEASASTSPQKTSESIGSEISTISSEPEPPETVSTPARGKGRGKGRGI

KAQPKIGMDHESGASANTSPQTETPTVNVNELQPSESPASVSTSKRKSRGRKRIQPTAAESEALVSSEGT

EYSETIADSEESSTAGSSQQSCRVRPRKQQPEDSESVEMVETPAKRGRRGKEPSVNRSPSLQRGRSSTEN

SPRIIFTGLYDKQGEKVVISLGGQLVDNIHNCTHLVTDKVRRTVKFLCGLAGGQIIVLPSWLEACKKAKS

FVDTSPFLVKDKDAEKQYNFDLQRSHEVALTKGLLEGYKVHVTKKVKPEPSQMKDIIQSAKGEFLTSMPR

SKEDRVFVISCNDDRSVCRKPMEAGIPVVSAEVLLTGVLQQELNLEEYKLFAEEMSDTTQETSSLSGQNK

RRNETSEGGGATSSSSTPGAKNSGSKRRKR

>Amphiprion ocellaris

MELNFVLASLDMDATQMISDSTLESDEEENEETSDNPRGRPVAKLCVLKNEHISETELPL

FLGDNVLGRDPNTCTLPLSAASVSKQHAIICISVYRGRGCHTEDQMEALLWDLGSTNGTR

KGRLKLTPNVRYALSGGDSVVVADIPCQYVICGADSVVSSQEDTRTPVSRNPSVKARVTD

DLRVREGDTCTGSEKRVNGGTKPRVSQTKTLVRASCLSFEQTPTHPQQTLVPESDSDSDG

ETAGGGERRCVALVSDSDSHKSSPICSTFLSPTNKVIPERYVSEEEEEEERAAQRATKSK

ESGENVTMKQESNGCTGKDELPVSTPAVSTDCTPAFDIDSDTDMEEEEEGVASAGPSTLN

TNQQVDQPPNTVQFHMDSDTDIEEDHDMLGKVPKTLPSSNENTKPPHCSLVIQSEDISTD

SNTDVDEDAAVLTDAAMKAKPASSQIAQIADSASSMQIHDFHLDSDTDVDEEEEEGECET

EKTDETVNKLDMKVIRPESAAAAPHNLHLDADDEAIPASTISEPTVAAAVRESCSSANAG

AVLDTLSDGDTDVEDNPPVGVPALQVDSDKDVEDKEAGIKEAGEDQIPSLHRENTPGFLV

PLLPNCSTPIQLSGNYSLCVTKWNKFRLQLAASSGELSSCSDIQEDVDFAVAETQVFALE

ASDYKEDQSSSRGESLQLGLSDSSHLQCHNQTLATEATQPFFSVEGDVISEATNTCAAVS

TADGLSAEDDLHLEVTEAHAENEDSDRCSVASKNVESQSDLALEATQAYISVLDSDPDDN

TNQDEGQNTPATETPPIVSAFAFAETQLMPFHVEEQSLAVDNSVCSVQQVNPRTQTKMEE

REENGKDAHPQERNHSEAQYMAATQPICTSSNEESDDDSFPGPRRRKAKPLQLSEEQTQC

LIDSNADGSQATAISEDHESGDMDLKPGPKKQKTKQLEEETQPLTSSEVSTVESQSLHTN

SLQLEDGKTQPSTSSNISAVETQQIVTSKEQDSDGPHRRTVKQLHLTEDNRQHSELSAAE

NQPIATCEDDAADSMLGPRKRKAKHLEDKTQLLTSNEVFADDDSVPVLPKRKATLQLEKE

ETQSEMSPVDTQPLKTTTGLKSQKRKRIESTADTTRAKMLGMEGNTHTVNKSKQKEGKQQ

EREKTEKEAKETDGRKEEQEETERQDGREEKEKIENERKVNKEKSQQENEEEVKPKAPAR

NSTASTNDDFPARRTRSRSNSSNSISSERSVSSVNTQESKGSGRGRGAKTPSKSPQAAIV

RTSYRRATVAAGPTEQDSSDVLPQRLLSKNNSSNSRSPEVSSCSVRSLNRGRRGRKAEPD

PDFIPAVTHHSDQNSKEKADSQQTRTTRGRQHSECAAADETDQSPHQEGCASEELLLPKR

NVRGRSQKAVRSEAVEESVTSTASGDRAKNRGRGRKTELKVLFTGVVDEAGEKVLARLGG

SMAKGIADMNCLVTDKVRRTVKFLCAVAKGIPIVTTDWLEKSGKAGSFLSPNAFVVKDPE

QEKKFTFCLQDSLRTASSQPLLQGYNIHVTKSVKPEPVHMKDIISSSGANFLPKMPTSNK

PQTVVISCEEDWLLCGPAVSASIPVVTAEFILTGILQQKLDFQYHELSPPAAALQSPAGG

KGRGRRKT

>Delphinapterus leucas

MLGATTMIMEDTQVINWEVEQEEEVEERPSESLGCSLEPLGRLRIFSSSYGPEKDFPLYLGKNVVGRMPD

CSVALPYSSISKQHAVIEILAWDKAPVLRDCGSLNGTQILRPPKVLGPGVSHRLRDRELILFADLPCQYH

RLDVPLPFVSRGPLTIEETPRVQGGTQPHRLLLAEDSEEEVDSLSEKCVVKGPRTSFLATVVPESDEEGP

SSPLDVPGPPFAFNLNSDTDEEESQQPGAGEGSSAARRVTAAETEQPKPVTTGIQLEKDQCSVKEKNNDT

KVERSARSRVVPVGVILERSQPAGEDSDTDVDDESGPLRRLTGVHLERAQPCGFIDSDTDVEEEGIPATP

AVVPVRKRHSFHEVGTESPRAPGVAHQQESPDGSDTDIEEGEAPLTVPLDKSRASVVIDSNTDDKEEVSA

ALTLAHLRESRAVAWNRDPDAEDDRAQPVALLEQSQASAGRDSDTDVKEKGLPVEKTGTVPRGHTGKAYS

EKSHPSLRDSDTEVTEEKSSPGFQASATVHVNTQVVEEVPPGPAVILLEKHQVPVAWTHQTDVEAEGGPA

KLPVVYLEEARPPPARDCDPDAEENASLAASAVADVRKSQLQAEEDTGTERAVAVLEQGRAFMAGAQGGS

PVAQVEQDLLPGSRNNIADLVVDTGTPGEPTQPQRRGAQTPTEREREPHVDRTMDSGDNHDDSEDLDLQA

TQCFVERENQSLEVPSVEDEPTQAFLFTLPQEPGPSRCSFQATGSLDEPWEVLATQPFCPRESEASEPQP

IATHIEAHASCPSPPTAAPQEQHPESPVHAEPLGIQGRGMQTVEEDMGTPRETAERVTPEREPLERETEK

LPSEGEREDVMGEEESTRGIQDREQKQVLARDTQKQESDKKVKSASTERDMESLKVEIETPEEIQEKERE

KQTLTREIFDREAEKPVAERECEAGGLQGKVPKVMLDRGPQTGETEAGGQDQKGQASGSTPEPGVGAGDL

QGLASDPIASGSQSGGGRGAPVGPRRQQRGYLNCKMPPAEKASRGDQESPDACRPPALQEASAPLQNPLI

SQSQKHPAPQSLLSPSLPPLERPIPRTRQNESQEALETPFSSELDSLHPKPKVKPQGSSPVSSVPLEPHP

TASTDQLVTPKPTSRATRGRTLRSSVKTPERNVSTVPELQPSAHTDQAVTPKPTSRATRGRTQRASVKTP

EPVISTAPGPQPSTPTDQAVTPKPTSRATRGRTQRASVKTPEPVIATAPEPHPSTPTSQATRGRTQRASV

KAPEPVISTAPEPQPSTPTDQPVTPKPTSQATRGRTQRASVKAPEPVIATAPEPQPSTPTDQPVTPKPTS

RATRGRTQRASVKTPEPDISTAPEPQPSTPTDQPVTPKPTSQATRGRTQRASVKAPEPVISTAPEPQPST

PTDQPVTPKPTSPATRGRTQRASVKAPEPVISTAPESQPSTPTDQPVTHKPTSRATRGRTQRASVKTPEP

VIATAPAPQPSTPTDQPVTPKPTSQATRGRTLRSSVKTPERNVPTAPELQPSAHTDQPVTPKPTSRAPQG

RTLRSSAKAPEPVVPITPEPQPSTSKDQSLTPEPTSQATRGRTQRSSVKTSQPTEPTAPDLEPSSPTEQP

VTPKVIAQGGQSRTLRSSTVNAVPAPTTPESHSPVPTGEPIPPEPIPEANCSRRPRATRKHGSLTAHVHE

PYSAPSEPNSSRNQRRGAVRAAESLSTIPEPAFAQLPEAPTRAPQIPKGEAADRSGFTPEPQPEASQNRK

RPLATADSPPLQKRLQRGEVPQKTAFLKEEEENPAAKQRKEEDVVIPGPGKRKREQTEEEPREIPSRSLR

RTKPLQESTAPKVLFTGVVDAHGERAVLALGGSLASSVAEASHLVTDRIRRTVKFLCALGRGIPILSLDW

LHQSHKAGCFLPPAEYVVTDPEQEKNFGFSLREALSRARERRLLEGYEIHVTPGVQPPPPQMGEIISCCG

GTVLPSMPRSYKPQRVVITCSQDFPRCAIPFRVGLPILSPEFLLTGVLKQEAKPEAFVLSTLEVSST

>Phocoena sinus

MEDTQVINWEVEQEEEVEERPSESLECSLEPLGRLRIFSSSYGPEKDFPLYLGKNVVGRMPDCSVALPYS

SISKQHAVIEILAWDKAPVLRDCGSLNGTQILRPPKVLGPGVSHRLRDRELILFADLPCQYHRLDVPLPF

VSRGPLTIEETPGVQGGTQPHRLLLAEDSEEEVDSLSEKCVVKGPRTSFLATVVPESDEEGSSSPLDIPG

PPFAFNLNSDTDEEESQQPGAGEGSSAARRVTAAETEQPKPVTTGIQLEKDQCSVKEKNNDTKVERSARS

RVVPVGVILERSQPTGEDSDTDVDDESGPLRRLTGVHLERAQPCGFIDSDTDVEEEGIPATPAVVPVRKR

HSFHEVGTESPRAPGVAHQQESLAGSDTDIEEGEAPLTVPLDKSRASVVIDSNTDDKEEVSAALTLAHLR

ESRAVAWNRDPDAEDDRAQPVALLEQSQASAGRDSDTDVKEKGLPVEKTGTVPRGHTGKAYSEKSHPPLR

DSDTEVTEEKSSPGFHLERSQASATVHINTQVVEEVPPGPAVILLEKHQVPVAWTHQTDVEAEGGPAKLP

VVYLEEARPPLARDCDPDAEENTSLAASAVADVRKSQLQAEEDTGTEWAVAVLEQGRAFMAGAQGGSPAA

QVEQDLLPVSRNNIADLVVDTVTPGEPTQPQRRGAQTPTEREREPHVDRTMDSGDNHDDSEDMDLQATQC

FVERENQSLEVPSMEDEPTQAFLFTLPQEPGPSRCSFQATGSLDEPWEVLATQPFCPRESEASEPQPIAT

HIEAHASCPSPPTAAPQEQHPESPVHAEPLGIQGRGMQTVEEDMGTPRETAERVTPEREPLERETEKLPS

EGEREDVMGEEESTRGIQDREQKQVLARDTQKQESDKKVKSASTERDMESLKVEIETPEEIQEKEREKQT

LTREIFDREAEKPVAERECEAGGLQGKVPKVMLDRGPQTGETEAGGQDQKGQASGSTPEPGVGAGDLQGL

ASDPIASGSQSGGGRGAPVGPRRQQRGYLNCKMPPAEKASRGNQESPDACRPPALQEASAPLQNPLISQS

QKHPAPQSLLSPSLPPLERPIPRTRQNESQEALETPFSSELDSLHPKPKVKPQGSSPISSVPLEPHPTAS

TDQLVTPKPTSRATGGRTLRSSVKTPERNVSTVPELQPSAHTDQAVTPKPTTRATRGRTQRASVKTPEPV

ISTAPGPQPSTPTDQAVTPKPTSRATRGRTQRASVKTPEPVFTTAPETHPSTPTDQPVTPKPTSQATRGR

TQRASVKAPKPVIATAPEPQPSTPTDQPVTPKSTSRATRGRTQRASVKTPEPVISTAPEPQPSTPTDQPV

TPKPTSQATRGRTQRASVKAPEPVISTAPEPQPSTPTDQPVTPKPTSPATRDRTQRASVKTPEPVISTAP

EPQPSTPTDQPVTPKPTSRATRGRTQRASVKAPEPVISTAPEPQPSTPTDQPVTPKPTSQATRGRTQRAS

VKAPEPVISTAPEPQPSTPTDQPVTQKPTSRATRGRTQRVSVKAPKPVIATAPEPQPSTPTDQPVTPKPT

SQATRGRTQRASVKTPEPVIATAPAPQPSTPTDQPVTPKLTSQATRGRTLRSSVKTPERNVPTAPELQPS

AHTDQPVTPKPTSQATRGRTLRSSVKTPERNVPTAPELQPSAHTDQPVTPKPTSRAPQGRTLRSSAKAPE

PVVPITPEPQPSTSKDQSLTPEPTSQATRGRTQRSSVKTSQSTEATAPDLEPSSPTEQPVTPKVIAQGGQ

SRTLRSSTVNAVPVPTTPESHSPVPTGEPIPPEPIPEANCSRRPRATRKHGSLTAHVHEPYSAPSEPNSS

RDQRRGAVRAAESLSTIPEPAFAQLPEAPTRAPQIPKGEAADRSGFTPEPQPEASQNRKRPLATADSPPL

QKRLQRGEVPQKTAFLKEEEENPAAKQRKEEVRRGLESPSLERAWGLGNSHQVLSQPQDVVIPGPGKRKR

EQTEEEPREIPSRSLRRTKPLQESTAPKVLFTGVVDAHGERAVLALGGSLASSVAEASHLVTDRIRRTVK

FLCALGRGIPILSLDWLHQSHKAGCFLPPAEYVVTDPEQEKNFGFSLREALSRARERRLLEGYEIHVTPG

VQPPPPQMGEIISCCGGTVLPSMPRSYKPQRVVITCSQDFPRCAIPFRVGLPILSPEFLLTGVLKQEAKP

EAFVLSTLEVSST

>Balaenoptera acutorostrata scammoni

MEDTQVINWEVEEEEEVEERPSESLGCSSEPLGRLRIFSSSYGPEKDFPLYLGKNVVGRMPDCSVALPYS

SISKQHAVIEILAWDKAPVLRDCGSLNGTQILRPPKVLGPGVSHRLRDRELILFADLPCQYHRLDVPLPF

VSRGPLTVEETPRVQGGTQPHRLLLAEDSEEEVDSLSEKCVVKGPRTSFLATVVPESDEEGPSSALDGPG

PPFAFNLNSDTDEEESQQPGAGEASSAVRRVTAAETEQPKPVTTEIQLEKDQCSVKEKNNDTKVERNARS

GVVPVGVILERSQPAGEDSDTDVDDESGPLRRLTGVHLERAQPCGFIDSDTDVEEEGIPATPAVVPVRKR

HIFHEVGTESPRAPGVAHRQESPAGSDTDIEEREAPLTVPLDRSRASVVIDSNTDDKEEVSAALTLAHLR

ESRAVAWNRDADAEEDRAQPVALLEQSQASAGRDSDTDVKEKGLPVEKTGTVPRGHTGKAYSEKSQPPLR

DSDTEVMEEKSSLGFHLQRSQASATVHVNTQVVEEVLPGPAVILLEKHQVPVAWTHQTDVEAEGGPAKLP

VVYLEEAQPPLAGDCGPDAEENTSLAASAVADVRKSQLHAEEDTGTEWAAAVLEQGRAFMAGAQGGSPAA

QVEQDLLPVSRNNIAHLVVDTGTPGEPTQPQREGAQTPTEREREPHVDRTMNSGDNHDDSEDLDLQATQC

FVERENQSLEVPSMEDEPTQAFLFTLPQEPGPSRCSFQATGSLDEPWEVLATQPFCPRESEASETQPIAT

HIEAHGPCPSPPTAAPQEQHPQSPVHAESLGIQGRGMQTVEEDMGTPRETAERVTPERGPLERETEKLPS

EGEREDVMGEEESTRGIQDREQKQVLARDTQKQESDKRVKSASTERDMESLKVEIETPKEIQEKEREKQT

LTSEIFDREAEKPVAERECEAGGLEGKVPKVMLDRGPQTGETEAGGQDQKGQASGSTPEPGAGAGDLQGL

ASDPIASGSQSGGGRGAPVRPRRQQRGYLNCKMPPAEKASRGDQESPAACRPPAVQEASTPLQNPLISQS

PKRPAPQSLLSPSPPPSEPPIPRTRQNESQEALETPFSSELDALHPKPKVKPQGSSPVSSVPLEPHPTTS

TDQPVTPKPTSRATRGRTLRSSVKTPEQNVPTAPELQPSAHTDQPVTPKPTSQATRGRALRSSVKTPEQN

VPTAPELQPSAHTDQPVTPKPTSQATRGRALRSSVKTPEQNVPTAPELQPSAHTDQPVTPKPTSQATRGR

ALRSSVKTPEQNVPTAPELQPSAHTDQPVTPKPTSQATRGRALRSSVKTPEQNVPTAPELQPSAHTDQPV

TPKPTSRGRTQRASVKTPEPVISTAPEPQPSTSTDQPVTPKPTSRATRGRTLRASVKTPERNVSTAPELR

PSAHTDHPVTPKPTSQAPRGRTLRSSAKTPEPVVPITPEPQPSTSKDQSLTPEPTSQATRGRTHRSSVKT

SQPTEPTAPDLEPSSPTHQPVTPKVIAQGGQSRTLRSSTVSAVPVPTTPEVHSPVPTEQPIPPEPIPEAN

CSRRPRATRKPGSLTAHVHEPYSAPSEPNSRSSRNQRRGAVRAAESLSTIPEPAFAQLPEAPTHAPQIPK

GEAADRSGFTPEPQPEASQNHKRPLATVDSPPLQKRLQRGEVPQKTAFLKEEEENPAAKLRKIEDVVIPE

PGKRKREQTEEEPREIPSRSLRRTKPIQESTAPKVLFTGVVDAHGERAVLALGGSMASSVAEASHLVTDR

IRRTVKFLCALGRGIPILSLDWLHQSRKAGCFLPPAEYVVTDPEQEKNFGFSLREALSRARERRLLEGYE

IHVTPGVQPPPPQMGEIISCCGGTVLPSMPRSYKPQRVVITCSQDFPRCAIPFRVGLPILSPEFLLTGVL

KQEAKPEAFVLSTLEMSST

>Bubalus bubalis

MEDTQILNWEVEEEEEVEERPSESLGYSLEPLGQLRIFSSSYGPEKDFPLYLGKNMIGRMPDCSVALPFS

SISKQHAVIEISAWDKAPVLRDCGSLNGTQILRPPKVLGPGVSHRLRDRELILFADLPCQYHRLDVPRPF

VSRGPLTVEETPRVQGGTQPPRLLLAEDSEEEVDSFLDKCVVKGPRTSSLATVVPESDEEGPSPAPDGPG

PPSAFNLNSDTDEEESQESGAGEASSAPRRGSAAETEQPEPVTAEIQIEKDQCSVKEKNRDTEIERDVRN

GVVPTGVILERSQPSGEDSDTDVSDESGPPRRLAGVRPKRAWSCNFIDSDTDGEDEGIPATPAVVPMKER

QIFHEAGTQSPQAPGVARQQESPADGDTDIEEGEVPPDRSQASMVIDSNTDDEEEVSAALTLARLRESQA

GKWTRDPDAEEDRAQPVALLEQSQASAGGDSDTDVEEEGLPVERRGMVPKGHMDREYSKKSQHPPRDSDT

EGKEDKSSPGVHLERSQASAQVEDEVPLGPAVALPEKCQVQGIVWTHHTDAEAEGGPARLPVLRLEEAWP

PLAGDCKLDAENTSSAAAGVRKSQLPAEKDAGTTWDAAVPEQDRALATGTQGGSSTAPGEQDLLPVSREN

LADLVVDTGTPGEPQPQREGAQTTTGREREPHGNRATDSGESLHDSEDLDLPATQCFADRKNQSLEAPSM

EDEPTQAFLFTLPQEPGPSCCSSQATGSLNEAWEVLATQPFCPREYEASETQTAVTLLDTRASCPPPSRT

AQQEQHPESPVRAEALGMEGRGMQTVEKDKGTQRETTERVIPEGGPPQNETKKLPSEGEREDVTGEEELI

RGIQGREQNQVLARDTQSQESDKKVKSASTGRGMEIVKLETETPKETQEKEREKQTLAGEIFESEAGKLV

VERESEVGGLEVKGPQELLDRGPQMRETEAGGQDQKGQASGPPSEPGAGAGDLQGFTSDPVASGSQAGGG

RGAPGSPRRQQRGDLNCEMPPAEKASRGDQESPDACLPPAAPEASAALPNSLISQIQKHPAPQSLLFPSP

APLELPIPRTRENENQEAPETPFSSELNSVHPEPKVRPQGSSPVSSLPLEPHPTTPTGQPIALEPTSGVS

RSGTHSSFDVTASSVVPTALALQPSTSTDQPVAPKPTLRAPRGRAQRSSVKTPEPNVRTDQPIAPELTAK

ATRGRAQRSSVKTPKPDNPTIPKPQPSTSTDQPVTPKPTSRAPRGRTPKSSAKTPEPAVPTASELQPAAP

KDQPVAPELTSRATRGRTQRSSIKTSKPDMSTAPEPQPSTSTDQPVTPKPTSRAPRGRTPRSSSKTPEPV

VSTASELQPSALTDQPVTPELTSRATRGRAQRSSVKTPDPVTTTTPELQPSTSTDQLVTPKRPSRAPRGR

TRRSSAKTPEPVVPTASELQPSAPADQPVGPWATQCRRHRSSVKTLEPVVPTAPEPQPSTSKDQSVAPEP

TSQATQSQTHRSSVKTSQPTEPTAPDLKPSSPTDQPVTPKVIAQGGPSRTRRASTASAVLVPTTPEFQCP

VPSEQPLSPDPIPEVNCSLRPRATRKHGSPTAHVHEPCTAPPEPNSRSSRNQTHGAMKAAKLLSTIPEPA

FAQLPEAPPHTPQMPKEEAADGSGFTPEPQPRASQNRKRPSATAHSPPLQKRLQRGRVPQKAASLKEEEN

PAARPRKEEGVVIPGPGKRKREQTEEESQGRPSRSLRRTKPVQESTAPKVLFTGVVDARGERTVLALGGS

LASSVAEASHLVTDRIRRTVKFLCALGRGIPILSLAWLHESRKAGCFLPPDEYLVTDPEQEKNFGFSLRE

ALSRARERRLLEGYEIHVTPGVQPPPPQMGEIINCCGGAILPSMPRSYKPQRVVITCSQDFPRCAIPSRV

GLPVLSPEFLLTGVLKQEVKPEAFAFSTVEMSST

>Bos taurus

MEDTQILNWEVEEEEEVEERPSESLRCSLEPLGQLRIFSSSYGPEKDFPLYLGKNMIGRMPDCSVALPFS

SISKQHAVIEISAWDKAPVLRDCGSLNGTQILRPPKVLGPGMSHRLRDRELILFADLPCQYHRLDVPRPF

VSRGPLTVEETPRVQGGTQPPRLLLAEDSEEEVDSLLDKCVVKGPRTSSLATVVPESDEEGPSPAPDGPG

PPSAFNLNSDTDEEESQESGAGEASSAPRRGTAAETEQPKPVTAEIQIEKDQCSVKERNRDTEIERDVKN

GVVPTGVILERSQPSGEDSDTDVSDESGPPRRLAGVRPKRAWSCNFIDSDTDGEDKGIPAAPAVVPMKER

QIFHEAGTQSPQAPGVACRQESPADGDTDIEEGEAPLDRSQASMVIDSNTDDEEEVSAALTLARLRESQA

GKWNRDPDAEEDRAQPVALLERSQASARGDSDTDVEEEGLPVGRRGMVPKGHMDREYSKKSQHPPRDSDT

EGKKDESSPGVHLERSQASAQVEDEVPLGPAVALPEKHQVQGIVWTHHTDAEAEGGPAQLPVLRLEEARP

PLAGDCELDAENTSSAAAGVRKSQLPAEKDAGTTWAAAIPEQDRTLATRTQGGSSTAPGEQDLLPVSREN

LANPVVDTGTPREPQPQREGAQTTTGREKEPHGNRATDSGESLHDSEDLDLPATQCFADRENQSLEAAPS

MEDEPTQAFLFTLPQEPGPSCCSSQATGSLNEAWEVLATQPFCPREYEASETQTAVTLLDTRASCPPPSR

TAQQEQHSESPVHAEALGMEGRGMQTVEKDMGTQRETAERVIPEGGPPQNETKKLPSEGEREDVTGEEEL

IGGIQGREQNQVLARDTQSQESDKTVKSASTGRGMEIVKLETETPKETQEKEREKQTLAGEIFESEAGKL

VAERESEVGGLEVKGPQELLDRGPQMRETEAGGQDQKGQASGPPSEPGAGAGDLQGFTSDPVASGSQAGG

GRGAPGSPRRQQRGDLNFEMPPAEKASRGDQESPDACLPPAAPEASAALPNSLISQMQKHPAPQSLLFPS

PAPLELPIPRTRRNESQEAPETPFSSELNSVHPEPKVRPQGSSPVSSLPLEPHPATPTGQPIALEPTSGV

SGSGTHSSFDVTASSVVPTALALQPSTSTDQPVTPKPTLGAPRGRAQRSSVKTPEPSVRIDQPIAPELTA

KATRGRAQRSSVKTPKPDNSTTPKPQPSTSTDQPVTPKPTSQAPRGRTPKSSAKTPEPAVPTASELQPAA

PKDQPVAPELTSRATRGRTQRSSIKTSKPDTSTAPKPQPSTSTDQPVTPKPTSRASRGRTPRSSTKTPEP

VVSTASELQPSALTDQPITPELTSRATRGRAQRSSVKTPDPVTTTTPELQPSTSTDQLVTPKRPSRAPRG

RTRRSSAKTPEPVVPTASELQPSAPADQPVGPWATQCRRHRSSVKTPEPVVPTVPEPQPSTSKDQSVAPE

PTSQATQSQTHRSSVKTSQPTEPTAPDLKPSSPTDQPVTPKVIAQGGPSRTRRASTASAVLVPTTPEFQS

PVRSEQPLSPDPIPEVNCSLRPRATRRHGSPTAHVHEPCTTPPEPNSRSSRNQTHGAMKAAKPLSTISEP

AFAQLPEAPPHTPQMPKEEAADGSGFTPEPQPRASQNRKRPSATAHSPPLQKRLQRGRVPQKAASLKEEE

NPAVRPRKEEGVVIPGPGKRRREQTEEESQGRPSRSLRRTKPVQESTAPKVLFTGVVDARGERTVLALGG

SLASSVAEASHLVTDRIRRTVKFLCALGRGIPILSLAWLHESRKAGCFLPPDEYLVTDPEQEKNFGFSLR

EALSRARERRLLEGYEIHVTPGVQPPPPQMGEIINCCGGAILPSMPRSYKPQRVVITCSQDFPRCAVPYR

VGLPVLSPEFLLTGVLKQEVKPEAFAFSTVEMSST

>Dromiciops gliroides

MEDTQLVDWEAPEEEPEDPNGSPPRFGLEPVGRLHLFSSVRGPEKDFLLYPGENVVGRIPGCAVALPFPS

ISKHHAIIEIPAQGRAPILRDCGSLNHTRLLRPPKLLSPGVSHQLRDQDLVLFADLPCQYHRLVGNTNPG

SRGALSVEETPRVPGTGGPRFPGTLLAEDSEEEGDSPLDRSVEIPATYSPSETVVPESDVEGASPGTKGA

LPLTFILDSDTDEEEDPPPMEPSLATKRGSTADKEWSRTGGENHPSAVGRDSDRDVERDEGIMTVPSGPH

LKENLPTDRDSDTDANEEGSLQRTLVVAPLVGTQPSSLEDSGTDVEEEGLSVRSANAHLEMCQPDMGDSD

TDIEEDSIPAAPPVVHLGKDQVPKGGTNNTDIKEEGAPAELSTIHLEKDQMGIPGGNGDNDTDADDNGSP

THMKKRQLPAEEDNGVEWAPAGDQLERTTKKVGTQGRIPLTELEQGLSPFPRVSSVGMVVETSTLEKSAQ

VLKEGTQNYIEGDSDPDVEGTEDVQNKSDDTSDLDLQDTQCFVEEANQNHRDGPDEPWEILSTQPFCTDQ

SEASGTQPINSCIAVHGACPFPPKTASDLQPGSTEKTDPLRSQGTENPNTEKQEQTMENDMENQVIEIQT

PTITPERKMGKGTFKRKTLANKGKRSKKKRVGPVVQAEIPERGQRHRAGRGIQNVMTERDIKSCGETKAL

EKEMERQTPGKDIFERQAPEKITENLAVDVKIPREMLEKEAEGEMSDYGQEVQNPVPVPKSEVGEGNLQE

LASNSQILSNSRHQRGRDDSKILPLEEVPMDNQDSSHVPHVASKVPVPEAPAPTSTPRITRSQNRRTSKP

LHSSSSPASQESPVLGTTRSRRQGILLAPNIEPLPQDSRDRSESFQSQTQKPFSTSSAPTAPLAPELHPP

NPKELPVIPEPRLQARQSRKRGTTCESSTVASVLPEHPPSIPTERSETLVPPLRVLRSQRAVVGETSESL

TATPEPSNSLTPEAPAPRATRSQRVGAVGSTPETQPRLPGRKRPPATKEPSPCSKKPRRGRSQRQESSKE

EEDEKPVEVETAVKTPVGVAEPAVQAKKEVKGTPSRSLRRARLSRESRTPKVLFTGVVDSRGEQAVLALG

GSLANSVAEASHLVTDRVRRTVKFLCALGRGIPILSLEWLHQSRKAGRFLPPDEFVVNDPEQEKNFGFSL

REALRRAQERGLLEGYEIYVTPGVQPPPPQMGEIITCCGGTALPSMPRVYKPQRLVISCAQDLSRCSAAA

RVKLPLLSPEFLLTGVLRQEAQLEAFLLSTSDPLPS

>Ailuropoda melanoleuca

MEDTQAVNWEVEEEEETKRPNESLGCSLEPVGRLHIFSSAHGPEKDFPLYLGKNMVGRMPDCSVTLPFSS

ISKQHAVIEIAAWDKAPVLQDCGSLNGTQVLRPPKVLSPGVSHRLRDQELILFADLPCQYHRLNVPLPFV

SRGFLTVEETPRVQGGTQPQRLLLAEDSEEEVDSPSERCVMKEPRTSPLAAVVPESDEEGPSPAPDGRGL

PFAFNLDSDTDEEESQHPAAGEASLAARTGSTAETEQPKVLATEIQLEKDQCSVKERNNDTKVEKDARNE

VAPLGATLERNQTAGEDSDTDMDESRPAEVHLERSQPSGVIDSDTDVEEEGIPATPAVVPMKRRQIFHGV

STESPQAPALVHLQESPPGSDMDVEQSELQLAVPPERNQASVVIDSNTDDEEEVLAALTLARLKESRADT

WSRDTDVEEDRAQPVALLEQSRTSAGRDSDTDVEEERIPMEKRGTVPKCPTDKAHSEKRQSPLRDSELGV

DKNKSSLGVHLERSQASATVDNNTQVEEKALTGPAVTLVGKRQVPAVRTSQTDAEVEGGPAKPPVMHPEE

APRPPLGDRGTDAEEGTSLAASAVADTGKCQFLAKRDAGTEWAAAVLERERALEARAQDSEDLDLQATQC

FVERENQSPEAVQSMEDEATQAFLVTLPQEPGPSCCSFQAPGALDEPWEVLATQPFCPTESEASEPQPVA

ALLDAHGSCPSTPRTTPQAQRPESPIHEEPLGIQGRGMQTVQKDMGTPREAVGGVAPERGPLNRETKSLP

AGERGDVIEEEALTRGIQVLAGDNQGQESDQKVKSASIKRKMESLNIEIEIPSEVQEEGIGRETFEREAE

KLVLERGGEPSGLGIEVPEVKVERSPQRGETGKGSQDQEGQASSLTTEPTAGTGDHQTLASAPGASGSQS

GGAPMSPRRQQRGHMTCKMPPSDKASVGDQESADACLPPAVPEASTPHQNPLLSQSRKHPVPQSFLSPSL

SSLEPIPRTTQNGNQEVPETPLSSEMEPFHPKSKVRLRGSSRKTPSTISSLALEPHSTIPTDQPLSPKLT

SRVTRGRTHRSSVKTPEPVVPTAPELQISTSKDQPITSESTLQVTRGRTHRFSVKTPELVVPMVPEVQPS

TSKEQPVTAELVSQGRTRKSVKIPEPVVSPATRGRAHRSSVKSPKPVIPTAAELQPSTSKDQCVTPEPTS

QVAWGRTRRSSVKTPEPTVPTAPELQPSTSKHQSVTPEPTSQVVWGRTRRSSVKTPEPTVSTAPELQPST

SKDQSVITEPTSGATHSRTHRPSVKTPEPIIPTAPEVQPSVPTDQPVIPKPTCQGRTPRSAKTPDPIVPT

ALDLQPTTPGGQPVTPKRTSRGRTPRSSSKTPKSVVTMVPELQASTPTDQPVTPKLTSQATRGRTQRSSI

KTSEPVAPTAPEQPSISTDQPVTPEPTSRATRSRTHRSFVKIPQPTEPTAPDLESLTPTDGLVTPKAQGS

QGKTLRSSTVSALPVLTNPEFQSPVPTDQPIPPEPIPQATCPRRLRATRKHGSLTAPIVCEPYSALPEPK

SRSSRNQRQGAVRAVESLGTVPKPAFSQLPEAPTQATQIQNVEATGGSELTQVPLPKAAQSRKRPLATVD

KPPLQKRLQRGEVTQKTVFLKEEKEDPMERPRKEEDVVVPGPGKRKRDQAKEEPKGIPSRNLRRTKPNQE

STAPKVLFTGVVDARGERAVLALGGSLASSVAEASHLVTDRVRRTVKFLCALGRGIPILSLDWLHQSRKA

GCFLPPDEYVVTDPEQEKNFGFSLRDALSRAQERRLLEGYEIHVTPGVQPPPPQMGEIISCCGGTVLPSM

PRSYKPQRVVITCPQDFPRCSIPSRVGLPILSPEFLLTGVLKQEAKPEAFILSTLEMSSS

>Ursus maritimus

MEDTQAVNWEVEEEEETETPNESLGCSLEPVGRLHIFSSAHGPEKDFPLYLGKNMVGRMPDCSVTLPFSS

ISKQHAVIEITAWDKAPVLQDCGSLNGTQVLRPPKVLSPGVSHRLRDQELILFADLPCQYHRLNVPLPFV

SRGFLTVEETPRVQGGTQPQRLLLAEDSEEEVDSPSERCVMKEPRTSSLAAVVPESDEEGPSPAPDGPGL

PFAFNLDSDTDEEESQHPAAGEASLAARTGSTAETEQPKVLATEIQLEKDQCSVKERNNDTKVEKDARNE

VVPLGATLERNQTAGEDSDTDMDESRPAGLHLERAQASGVIDSDTDVEEEGIPATPAVVPIKKRQIFHGV

STESPQAPALVHLQESPTGSDTDVEQSELQLAVPPERNQASVVIDSNTDDEEEVLAALTLARLKESRANT

WKRDTDVEEDRAQPVALLEQSQTSAGRDSDTDVEEEGIPMEKRGTVPKCHTDKAHSEKRQSPLRDSELGV

DKDKSSLGVHLERSQASATVDINTQVEEKALSGPAVTLVGKHQVPMVWTSQTDAEVEGGQAKLPVMHLEE

AHPPPLGDCETDAEEGTSLAASVVADTGKCQLPAKRDAGTEWAAAVLERERALEARAQGESLVSQVEQDL

LPVSRENLIDLVVDTGTSGEPIQPQREGAQTPTERERDPHGNKTKDFGDNHGDSEDLDLQATQCFVEREN

QSPEGALDEPWEVLATQPFCPRESEASEPQPIAAHLDAHGSCPSTPRTTPQAQHPEGPIHEEPLGIQGRG

MQTVQKDMGTPREAVEGVAPERGPLNRETKNLPAGEREDMIEEEALTRGIRVLARDNQGQESDQKVKSAS

IKRNMESLNIEIEIPNEVQEEGIGKQTLAREIFEREAEKLVLERGGEPSGLGIEVPEVKLERGPQRGETG

KGSQDQEEQASSLTSEPRAGTGDHQGLASAPGASGSQSGGAPMSPRRQQRGHVTCKMPPADKASVGDQES

ADACLPPAVPEASTPHQNPLLSQSQKHPVPQSFLSPSLSSLEPIPRTTQHGNQEVPETPLSSEMEPLHPK

SKVRLRGSSRKTPSAISSLALEPHSTSPTDQPLSPKLTSRVTRGRTHRSSVKTPDPVVPTAPELQISTSK

DQPVTSESTLQVTRSRTHRFSVKTPELVVPMVPEVQPSTSKEQPVTAELISPGRTRKSVKIPEPVVSPAT

RGKAHRSSVKTPKPVIPTATELQPSTSKDQSVTPEPISRVTWGRTRRSSVKIPEPTVPTVPELQPSTSKD

QSITPEPTSQVAWGRTRRSSVKTPEPTVSTAPELQPSTSKDQSVITEPTSGATHSRTHRSSVKTPEPIIP

TAPEVQPSIPTDQPVIPKPTSQVRTPRSAKTPDPIVPTALDLQPTTPGGQPVTPKRTSRGRTPRSSSKTP

KSVVPTVPELQTSTPTDQPVTPKLTSQATRGRTQRSSIKTPEPVAPTAPEQPSISTDQPVTPEPTSRATR

GRTHRSFVKIPQPTEPTAPDLESLTPTDRLVTPKAQGSQGKTLRSSTVSALPVLTNPEFQSPVPTDQPIP

PEPIPQATCSRRLRATRKHGSLTAPIVCEPSSALPEPKSRSSRNQRQGAVRAVESLRTIPKPAFSQLPEA

PTQATQIQNVEAAGGSELTPEPLPKAAQSRKRPLATVDKPPLQKRLQRGEVTQKTVFLKEEEEDPTERPG

KEEDVVVPGPGKRKRDQAKEEPKGIPSHNLRRTKPNQESTAPKVLFTGVVDARGERAVLALGGSLASSVA

EASHLVTDRVRRTVKFLCALGRGIPILSLDWLHQSRKAGCFLPPDEYVVTDPEQEENFGFSLRDALSRAQ

ERRLLEGYEIHVTPGVQPPPPQMGEIISCCGGTVLPSMPRSYKPQRVVITCPQDFPRCSIPFRVGLPILS

PEFLLTGVLKQEAKPEAFILSTLEMSSS

>Sciurus carolinensis

MEDTQAIDWDVEEEEETEQSSESLGCILEPVGRLHVFSGAHGPEKDFPLFLGKNVIGRLPDCSVALPFAS

ISKQHAVIEISAWNKAPVLQDCGSLNGTQILRPPKVLSPGMNHRLRDQELILFADLPCQYHCLDVRPPGV

SRGHLTVEETPRVQEGTQPSRLPLAEDSEEEVDFSKGCVVKESRTTSSPLAMVVPESDEEGPSLAPGDSL

AFNLDSDTDEEDGQQPAAREASSAARKGAAVEAEHPEANGMRTDSWLGEAQPSLESCKDTKVKRGKGNRI

VSVEVILERSQSPGDNSDTDVDEENRLPGEPAEAHLERIQPSGFLDSDTDVEEEGIPATPAVVPMKKRQG

FHGFGTRNPGTLGLAHLQESPAGSDTNMEEDEAPLTGPLERSQTSMVIDSDTDDEEEVSAALTLARLKES

GAILWNRNADIEKDRAQSVLLQEKSLTTSGRDSDTDMEEELPVEKRETVLNGHRDKEETRITAHSEKGQP

PLGDSDVGNEADMSSSGIFLEKSQVSSTMRDVNIEVKQEVPSGPTVTCCGKHQVSVEGTNQRDREANGGP

AKLPVMPLEAVQPSDEEGETDMEVGMSSAVADIRKSQLLAEGDAGTERATAVLESEGTLKERAYGGSLMA

QIQQMVVHTGAPGEPLQPQREESQTLTGREKKLHIGETKDSKDSHDDSEDLDLQATQCFVERESQNLDGA

LDEPWEVLATQPFCPRESEAAEPQLIATHLEANGSCLSPPRATPQDQHPESPVHTEPLGTQGIEIQTVEK

DMGTLKETERVNPERGPLEREIREGPPEGERKDVMGEEERTKGIQDRQQKQALARDTQRPESDKKVKSMS

PERHRENLKVEMEISEETEETEIEKQTLTREIFEKVVEKPVPERVCEATELEVNLERGEIEGGIQNQKGQ

AASPTLEPRVGAGELQGLASAPVVSGNQSGGGRGTPVSPRRQQRGHLNCKMLPAETTSRGDPESPDVCLP

ATMPEASALPPNSFISQSQKHPALQSLLSPHSSSSEPPIPRTRHKGSQETPEPPLPTELEPFHSKPGVRP

RRSSRMTPSPLSSAALELHSVAPTAQPAIPKSTSQATKRRTHRSSVKTPELQLSTPTGQPITPTPISRVT

RVKTNRSSVKTPESVVHTAPEPQPSISTDQPDTSKLTSRVTRGRTLRSSVNTPEPVFPIAPEFQSSVSTD

QPVDLKPTSRITRGRTHRSSVKTPEPVFPVTPELRASTPTEQLFTPKSTFVDSQGRTHRSSVNTSEAILP

TDSELQPSTSTDQPVIPQPTSRVTRGRTHRTSVKTSEPILFTGPKLQPSTSTVQPVTPNPTSRVTRGRTH

RSSVKTPEPIVPTAPELQPSTSTEQPVTPKRTSWATQGRTHRSSVKSPEVVVTMASELQASSPIDRSIPR

KPTPRISRGRTRKSVETPEPVEPSAPDLEPPSSTHQPVTSKVIHQSGALESSTLSDATLPVTPEFHPPVT

TDQPVPFESTPQTSCTRRQRAAGKHGSLTPLIVHKPSSAPPEPKSRSSRNQRAGAQRETEGLGTIPEPAF

PQLPEAPTHASQIQKVETAGRSGFTPEPPPKTSQSRKRPSTTMDSPPLQKRPQRGEVSQETAFPKEEEDA

QERPVKEEDIEIPEPDKRKRDQAEEEPKGIPSRSLRRTKPNQESAAPKVLFTGVVDARGERAVLALGGSL

ASSVAEASHLVTDRIRRTVKFLCALGKGIPILSLDWLHQSRKAGCFLPPDQYVVTDPEQEKNFGFNLRDA

LHRARERRLLEGYEIHVTPGVQPPPSQMGEIISCCGGTVLSSMPRSYKPQRVVITCPQDFPRCSIPSRVG

LPLLSPEFLLTGVLKQEAKPEAFVLSTLEMRST

>Peromyscus californicus insignis

MENTQVINWDAEEEEETEISSGSLGYSLEPIGRLRLFSSTHGPERDFPLYLGKNVIGRSPDCSVALPFPS

ISKQHAVIEISACNKAPILQDCGSLNGTQLVKPPKVLTPGVSHRLRDRELILFADFPCQYHRLDVPPPLV

SRGLLTIEKTPRIQGGSQTSRVLLAEDSEEEMDFPTGRCVANGSRNTTSPSATVVPESDEEGSSPAPSVP

RPSLPLDLGSDTDEEQGQQPAVGGSSSAARNGIRLVQAQPAEQKFKDPKVTSGAGSGAALAGPVVEKSPT

PGEDSDTEVDEEHQPSVDSDTDMEEERIPVTPPVVPGGKKKKKKRVLLGVGTKDPGAPGAAHLQDSPAGS

DTDVEEGKMPLAVPQERNHTSMVIDSDTDEEDEVSAAFTLAHLKERGITLWSRDPGMEEVKSQPQVLVER

SQSASGRDSDTDVEEGKREMVPDSPMDLEEALIVTHSENQPPHRPGDVDEDVDMSSPGGHLEGNQAFSAT

VDNNGAQEEEEILPEPAVTLGEKHQLPPEEAQPPEEDWETAVEEGSSSAVADVRKSQQPVAEDTGTEWAA

SGSEQESTLEVGTQSRPPATPVEQVMVCTGTPGGPTQPQRKEAQTPTGREKGAQMDRTQNAKDCCDESED

LCLPATQCFVKRESQSSEAVQSLEDEPTQVFSCILPQEPGPSRASLQTPGPGALDVPWEVLATQPFCLRE

PEASEPRIIATHLEAQGSSPSPTSAPPGHQHLVHKELLGLEGREIQTVEKAMGIPKETAGSVTSEREPLE

RTPNSGREVVMEEEELTQGKEDREPEKGLARDRQGQESDKVKGRGREGSGESSKVETEMSKDTPKREREV

GNPEPEREWEPAGSEVTLDSGLTEGGSHDQTEQIADLTLKPGFGMGDLEELASALVVSGSQGDGGKGDPV

SPGRQQRGHLSFGMTSAEKASEGDPEPPAHGLLSPVPEASAPPQSLLTSQSQKQSTPQPLFLTSSPSEIQ

LPGTFHTKPNVRPRRSSRMTPSPHSSAAPRPASRTTRGRANRSSTRTPEPTVPTDSELQPPTSTEQPVIP

KPTSQGTQGSTNSSFVNTPEPVVLTGPETQPPTSTEQPVNPNSTPRATRSRPSRSSIKTTEPLIPIGPEL

QPLPSTEQPVIPKPTSPTTRGRPRKSSVRTPELVVATVSELQPPTSTEQPVNPKPTSRGRPRKFSIRTPE

PTVPTVSELQPPTSTEQPVTPNLTSQSSQGRTRRSVRTPEPIVPTGHELQPPTSTEQPATPEPSSQGRTH

RSVRTPEASITTTSELQSFTSREQDAHKPTALGTRGRRCKASNENSESVGPVAPDFEPPISKDHLVAPEV

IGQSITLKSSPLSAPPVSTTPKLQPPVPTAQPVPLEPIPQASNRRRRKAAGKQGSHTVPIGHEPYSAPSE

PESQSSTSQGSGALEVAESITVTPEPTVPQVPETPTHSPLMQNEAAGRLGSIPKPQPEASRVRKKPSTTT

DSPVQKRPRRRVPQKTTEPKEEDLSETQVKEESQEKAITTPDKRKRHCAEDETQGNPSRSRRAKPNQEAA

APKVLFTGVVDSRGERAVLALGGSLASSVNEASHLVTDRIRRTVKFLCALGKGIPILSLNWLYQSRKAGC

FLPPDDYLVTDPEQEKNFSFSLRDALTRARERRLLEGYEIHVTPGVQPPPPQMGEIISCCGGTILPSMPH

SYKPHRVVITCTEDLPRCAIPSRLGLPLLSSEFLLTGVLKQEATPEAFVLSNLEMSST

>Ochotona curzoniae

MEDTQAITWDEEEEDTQQPSYSSGGSSEPVGRLHVFSSNYGPEKDFPLYLGKNVVGRLPDCSVTLPFSSI

SKQHAVIEILARDRAPLLQDCGSLNGTQILRPTKVLSPGVSHRLRDQQLILFADLPCQYHRLDAPQPSVS

RGPLTVEETPRAQGGIQPTRLLLAEDSEEELDSHLKKCVMKDPRSAFSPWATVVPESDEEGLSPAPGGPG

PSSSFKLESDTDEEEGQLSAAEETSSAARRSAAIVAKQPNGVAAKQQPAVTETGNDTEVQKNADNRLVPD

GVILERNQAPAEESNSDVDDNRLPRRPTVSHVQRAQPSDVMDSDTDVEEEKIPATPAVVPVKKRHGFCGV

GPSSPGALGPQESQAGGDADGEESKASLAIPLVRSQAPMVINSDTDEEEEISAALTLARLKESPAAGWNR

DRHVEGDRGQPKVLLHQNQSTSERDSDTDMEEEKGVPEGQTVPSGHTNRNGTLYTAHSTMSAPPPGEKDI

KVETEMSSPGIHLQRSQDCTTVVVKTEKEEEVSREPAVGHLQKGQVLGETTNQIKVEAEGDPAKLGCEAD

AEEGTSLAASVVTDAKKNQIPVKEAADTDYTTARQESAVQLGAQDRSPVAQVEQNFLPVSKKNLTDEVVD

TGSPGGTTQSQREGAQTPIGTERELHMGSTKGLEEDSDDFDDLELQATQCFVDRDVQHLEDLNVEDESLL

NLPPEPGSSCCSSATMDVSWELMATQPFCTTESDASETQSTATHPEAHGFCLSPPSTVPQEQRAESPEHT

ELLGSQGRAMQTMEKDMGTAKETADRVTPERGPCERDTSQLPPGEQEATLGGGQSTREVEDREQQQLLAG

DTENQEPDHREESASPERDQESLQVEMETAEEIQSRETEKQTLGSEVFEREVERPVPEKECEPARLEVTP

VGERREQTEGSQDQREQASSPRPQPGVGEGEPQGLASTPIVSESQSGGGRGAPGSPTRQLRDHLKCNLSR

DETSKGDPECPDACRPEALTPLPDPLISQDQQHPAPQPFLPPSPPAFESSIVRTRQNGNQTPESSLSSEL

EHEVRPQRSSPVFSAALEPHPPTPPEQCDTPKPIPRATMGRSHRSSIKTLELAELTVPDFQPSTSTEQSD

IPRPTVRATRGRTHRSSAKTPEPAKPPVPTEQPDTSKPTPRGRACRSSTKTPEPAEPPVPTEQPDTSKPT

SRGRARRSSTKTPEPAEPPVPTEQPDTSKPTSRGRARRSSTKTPEPVELPIPTEQPETSKRTSRGRARRS

STKTPEPAELPVPTEQPDSAKPTSRGRTCESSTNTLEPVEPPVPTQQPNTPKPTPRATRGRTRGSSGKAL

EPADPPVPPEQPDTPKPTAQATRGRTRGSSVKTPEPAKSPVPTEQSDTSKPSSRGRTRGSSVKTPEPAKS

LVPTEQPDTSKPSSRGRTRKSSTKTPEQVEPIASDLEPSSTRDQPVTPKAIAQSNQSKTLRSSRSSVVPV

SATSEFQSAVTAEQQTLQPIPQGSNSRAQRVTRKQSSPKAPVVHKPCSALPEPKSRSSRNQRQGAVRAAE

SLGTVPEPSFSQPPGVPTHAPQIQKVEASDISEVAQELPPKVSSSCKRPSSFIDSPPRHKRLRRGQVSPK

TVFPREEEEGASEKPRREEDIVVPGPGKRKRGQAEKEPQEVPNRSRRRTKPLEPSAAPKVLFTGVLDPRG

EQTVLALGGSLASSVAEASHLVTDRICRTVKFLCALGRGIPILSLDWLHQSRKAGCFLPPDEYVVTDPEQ

EKNYGFRLRDALSRARERRLLEGYEIHVTPGVQPPPPQMGEIISCCGGTVLPNMPRSYKPHRVVITCHQD

FSRCSGPLRLGLPVLSPEFLLTGVLKQEATPEAFVFSTLEMPST

>Felis catus

MVMEDTQAINWEVEEEEEKEIPSESLGCSLEPVGRLHIFSSAHGPEKDFPLYLGKNMVGRMPDCSVILPF

SSISKQHAVIEILAWDKAPVLQDCGSLNGTQVLRPPKVLSPGVSHRLRDQELILFADLPCQYHRLNVPLA

FVSRGPLTIEETPRVQGRTQPQGLLLAEDSEEEVDSPSERCVVKEPRTCPLAAVVPESDEEGPSPAPDGP

GPPFAFNLDSDTDEEESQHPASGEASSTARRGSTAETKQSTAMATEIQLEKNQCSVKERNNDTEVERDAR

NGVVPLGVILERNQPAGEDSDTDVDDESRPPGRPAVVHLERAQPSDFIDSDTDVEEEEIPATPAVVPMKK

RQIFHGISTESPRTHALVHLQESPTGSDTDVGEGEIQLAVPLERSRASVVIDSNTDGEEEVLAALALAHL

KESRATTWNRDTDLEEDRAQPVALREQNQTSTGRDSDTDMEEEGLPMEKRGTVFKGHTDKAYSEKRQPPP

QNSDLGVDKDKSSLAVHLERSQASATVDINIQVKEKVPPGSAVILVEKHQVPVVWTDQTDVEVEEGQAKL

PVMHLEEAQPPSGDCETDVEGISLAASVVADIRKSQLPAEEDAGAKRAVAVLEHKRDLEARAQGGSLVSQ

VEQDHLPVSREDINDLVVTGTSGESIQPQREGAQTSIEREREPHMDRTQDSGDNHGDSEDLDLQATQCFV

ERDNQNLEAQSMEDEATQAFLVTLPQEPGPSCCSFQDTGTLDEPWEVLATQPFCPRESEAPEPEPIVAPL

DAHGSCLSTPWTIPQGQNPGSPVHIEPLGIQDKGMQTMEKDMGIPREAAEGVAPERGPLDRETENLPSGE

QEDVIGEEELTRGIQVLARDTQGQESDQKVKIASIKRNMESLNVEIEITREIQEKEIEKQILAREIFERE

TEKLVLEREGEPNGLGVEVPEIILERGPQRGETEKGSQDQEGQASSPTLELETGTGSHQGLASASVASGS

QSGGGEGVPMSPRRQQRGHLTCEVPPAEKAFRGDQESTDACQPPAVPEASAPHQNPLLFQSQKHPVPQPF

CSSSPSSLEPIPRTRQNRNQKVPETPLPKSKVRLRGSSRKTPSPVSSVALEPHTAIPTDQPLSAEPTSQV

TRGRRRRSSVKTPELVVPTAPELQPSTSKDQPVTPELTSRITRGRTQRFSVKAPEVVVPTAPDVQPSTSK

DQSVTPELISQDRTHKFVKTPEPVISTPSQLQPSTSKGQFVLTESVSGATQGRARRSCVKTPKAIVPTAP

KLQPSTSKDQPVTPEPTSRVTRGRTRGSSVKIPPESTVPTAPELQPTTSKDQSVLTGATHSRIPRSSVKI

PELVVPTAPEVQSSIPTDQSVTPKPTSQRRTSRSFVKTHEPTVPTAPELQPTTPKGQSVTPKRKSQGRTP

RSSSKTPKPVVPTVPELQASTPTDKPVTPKLTPRATRGRTQRSSVKTPEPVVPTASELQPSISTDQPVTP

EPTFRATRGRIHRSSVKIPQPIESTASDLESLNPIDQLVTPKAIAEGGQGKTLRSTVSAVPVLTTHESQN

PDPIGQPVPPEPIPQANCSRKRRATRKHGSFTAPIVHEPHSAPPEPNSRSSKNQRRAVRAVESLRTIPEP

ALAQLPEAPTHATQIQKVEAAGRSEFTPEPQSKPSQSRKRPLATVDSPPLQKRLQQKTVSLHKEEEDSAE

SPRKDEVAVIPGPGKRKRDQTEEEPRGIPSHSLRRVKPNQESTAPKVLFTGVVDARGERAVLALGGSLAS

SVAEASHLVTDRVRRTVKFLCALGRGIPILSLDWLHQSRKAGCFLPPDEYVVTDPEQEKNFGFSLRDALS

RARERRLLEGYEIHVTPGVQPPPPQMGEIISCCGGTVLPSMPRSYKPQRVVITCSQDFSRCSIPFRVGLP

ILSPEFLLTGVLKQEAKPEAFILSTLEMSSS

>Erinaceus europaeus

MEDTQAINWDVEEEEQPEIPGEYFGCSLEPVGRLRIFSSTHGPETDFPLYLGKNVIGRMP

ECSVVLPFPSISKQHAVIEILSWSKAPILKDYGSLNGTQILRPPKVLSPGTSHRLRDQEL

ILFGDLPCQYRRLSTPPSLVCRGPLIVEESPRVQGGTQSQGILLAEDSEEEVDSLLERCV

AEGPRTTSSPLVTVVPESDEEGPSPAPGGPGPSFTFNLDSDTDEEESRPPAAGESSPATR

RGATTETELCKAERVVTESQLLKDQCSVKERDSDIKLERDAKKEVIPKRSPPAGEDSDTD

VDDESTSTEKPAEAHLNSDTKVEEKRSPKISAEVSVKEEEISHGADTKIPELPGSAHLQK

GLPDSDTDVKDSKAPLMVPPGKSEIPMTTESSTDDKEEVSAALTVARLKESHAIPCKRNS

EDRAKPVVLGQSYSSTGRDSNTDVEEKGLVEKRETVSKDHTTKTHSEKSQLSLGDSDIKK

EEDTSTSGVSLESSQASATTVISANVEEDKHQVPGVRVNQTYVEAEGGPAKLPEVHIKEA

CTPPAGDFEIDAQEGTSLVPSTMTGIKKRELPAERDSETKCAASVLKAGTQNGSSVAQVE

QDCLPILRENLANPGVDANPPEEPTNSQRERTHTHRERGRKLHADMTKDSGNNHDNSEDL

DLQSTQYFLRESHSLEAQSIEDEPTQAFLLPLPQKSGPSQCNLEAQDAVDEPWEILATQP

FCPRESEASETQAIADCLEPHGSCSLSRRAAPQDQHPDSPVQAEPLAIESKGLQTLEKDT

GHLNCRMPPAKDDQDYPSVSLPPSVPEASGSLQNPLISQNQNHVAPQLPLSPCPPPLEPP

IPITRQNGSQEAPEIPLSSELESFHPKVRPRKSSRKTSPPVSSLNLEPHLTAPTDQPVRS

ESKSQVIRGRARNRSSVKTPEPDVPFLEPQLSTSRGQPVNPKPISRVTRGRTHRSSINTP

EPDVPSTSELQPSTSVEQLVICKPVSRVTRGRVHRSSVNTPEPGVSIAAELQSPAPIDQP

ISPEPVSQVSRGRTHRSSVKTPESVVPIASEIQPSTPTDQSVISGITQGRTHKSVKTPKP

VNPTPCELQPSTSTDQLIIPEPTSRATRGRTHRSSVKTPQTCELTSPKLEPSTPTNQPVT

PGAIAQRDQSKTLRSSKVSVPVPATPEFQSPEPTPQTNGSRRQKTTRKQTSLTVTISHET

YPGPPEPKPRSLRNQKREAMREAKSSRTTSEPAFAQPPELPTHATQIPKVEAPGRSGSTP

DSQSTAPQSHKRSSSTVHSPPLQKRLQRGEDHQKKDFINEDKEDTSERPGKKEDIVTSGP

GKRKRDQAEEEPKAIPSRSRRRTKSTQDYTTPKVLFTGVVDVRGEKAVLALGGSLASSVE

EASHLVTDRVRRTVKFLCALGRGIPILSLDWLHQSRKAGCFLPPDEYIVTDPEQEKNFGF

SLRKALSRAREQKLLEGYEIHVTPGVQPPPPQMGEIISCCGGTVLPSMPRSYKPQRVVIT

CSQDFHRCSVPCRVGLPILSPEFLLTGVLKQEAKPEAFILSTMELSPT

>Anolis carolinensis

MEQTQLLDWDEEGDTIESSNGDTPKPVGRLHLLSSKYGPEKDFWIYPGENVIGRLESCQV

CLPASSVSKAHAVIEVPSSDGPHLLYDKGSLNRTRRQRMVLIPQVRYSLQDGDSLIFGDV

GCQYFMLTPEAELESPNDSVEIPPTQTRVEASTLVIEETPAPGRKMRMRFGGVLVQDSDK

EEEEEVNEAGRSVPHRRGDGSVSSLEDARQPNLASSMFSSPSVVPESDEESGELSVSDLP

CPSLHLRFESQDSEVTPLENGGPPPLDKEKATVQSGTPEAEPKGPPSMEEDAAEKQGAAP

SGHSLVENLQLDSDTDVEDEEIAGSMSRSSGPGNLEEDDKALEISSDTDVDDPVLVDPDA

TSQKTHPAVIYVSSASDQEEVAKDSGIPKAQKTAENGDDDTDAEDGMENPSVVYLENHEP

VPQMEGGCQSMGKAEEPHPKGSQPGENEDSDTDVEEITQEPKEKATSQRSHECQNRGADV

DDTPLKMDNPSETLKTHKAALDSDEDADVDDTLPKAETLKTHKAAPDSDGDTDVEMSNLV

LENSHIAQLHSSHPISFKDNDSDLKDIPHKKAVCQVSSCLDQPSADTKEVSNLNVQQQSS

EEAVNEQSKSSDGKENPEIIDKGAALPQIQCLPALSVDSDTDVEEEEVEIQDVAPKVEHK

PIVAGSSGDGSRSNPENNAVESPNSLGPIEVEDSDTDVEVVSPSHKESVAQDDDADVEEV

VAPLPMKPIEEQETQLLASERSQANGEKLDGSAVDVRIHHEPGKEDDDTDAEEKKSCSGE

ESSTDDDQDIDLQATQCFLPSEPSSPGIEPARDPIVADSHNNLEEEPTQDFRTPPAQTRL

LSGKKWKSPQKEEDSDLDAYALEATQAFCTEPRSLSEEPTQAFVVEEEEEIIQNTAERGK

CSVPETQNVTVSATSKQQATSFSGKSTQPYSIGVACSEPNSETAVEEVAEECNKEEEIRA

VQSVQMPLLGSSQPLTLQEVQRKSMTEERVCMMVPEVGSAGGGQLEERCSVPVKQPRDAD

QVPGCSKEEPLQPEASAPVQRHNLRSSLTPSPAPVSQRRSLRCRIRAVCTDTQQSEEPAA

PVSRLRRLRQQTSSALYMKELEGKTDQPKEEKAHPNKKSKTAESTVTTRLSRTRSTQRES

DATGRLKGDMTVAAGSPGTRELTRRGRKGATTPPMKKEEPELPQTRSSRRSNSSVNTPSP

KGTRRAVKPEQDSPAQSTPSSGRRLRRQSTESKLVGMRSQPQSQSSVGSGAPSPKVLFTG

VIDEEAEQVVRELGGSLAESVFDCTHLVTDRVCRTVKFLCALAQGIPIVTLEWLQKSRQN

SFFLAPKSFLVRDPEQEKKFRFSLATSLRTAQRDGGLFQGYEIHVTPNVKPEPEHMRDII

KCSGGTYLPRMPRTYKDKRVIVSCPDDLPHCKPAQNGKVPITNSEFILTGILQQKIDLDA

HQLNAVAISSPTTSPATRASKRRAVAQSAPAPPSRAKRLR

>Pocillopora damicornis

MDFDQTQVIDDFELEEAESEEENKVRSKKIEVAHLKVFSQQGFKESIFPVFKGDNFIGRDGKCNITIPIK

ALSKKHACIEVQRDLHLLYDCESKNRTRKGKSVLKPKVRYELKHGDMLTFGDVTCQYLMVMEEEEDDGDE

TVSETGSESMLTDLNTEEGMKKIDKPIESEDVSMSSTIDVHVKGAAPRESLLQAKPDHETTKPSKDYEDA

YAADTDSDTDEERPLVTTAVPTILYSSEDEKEISNEQRVSSVPIKQDACLEGQTLAFGLGSPSHVFQCRD

STGSSGSHSAQTRSIPQTLKVSSESDSLSDASPFRHPGARGSVTQPPCQPTLIYGSESDDGSPIKRPRHP

AARVSDKGPSCEPTLLYGSESDESPKKKPGPPMVLNEPTLLYESNSDEDNDKAAKRLKESPADVVEKDKA

DSLLEERPNTEKEMVAVKDTDATLPYYRAEMSSTDDEGDSEGDGPDNKTSVDETPAPIQQTVAYNTKEIQ

KDDSTDDEQNNEAVDDYGADVATQAYTVQSDSEFEIESVANGDKAVARDLHKAVDPCDLEATQAYCIEEG

EDSDSLDSQPLPIGTAITAATDEIQATLAYGIGEPESVSDSERDNTCNNDDDSKRGQVQTLAYDLQATQA

YGIGGGDDEEEDLQKNSTKTEGEQPSDLDAGVSRGDDVQATIAYGLEATQAYGAEEIDDEGTAIEGRSCT

ASSHNDMIEATQAYGIDDPSAEETEPPAQVTDKDETENGVSAVSGGDKNKPSASPAVDVVDDSQESEDVT

PRRSRSRRGRKKPVIEDSQGEAREQAGSAENPTESEVEITNTDKRAENNVDSTPSSIFNRNGKNPRVTVS

SRKGRRGAMRVTIDPGTPDEDEGKETSSASSGRKGAKETNGSENQDKETSKVPASTSGRKRGKQAGKGKL

DEEVEETPVRKSRRGQKGKAAQMDATFLSEMPLEPSIDTTAKELGEVSGDAVESQPDSSAEAVPTRGKGK

GKGRGKGKGRKNEASVEESLKRESIGSEVSIISNEPVPGEAVSTPTRGRGRGKGKGRGKKTQPKRAIDCE

SEAFASTSPQIVQETPTVNVNELQPSESPASVSASKGKGRGRKRIQPSAAELEALASSDGTEYSETIADL

EESPTTGSSKQSRKGRQRKHPQENSESLEMEETPAKRGRRGKEPSVNRSPSLQRGKSSTENSPRIMFTGL

YDKQGEKVVTSLGGQLVDNIHNCTHLVTDKVRRTVKFLCGLAGGQVIVLPSWLEACKKAKGFVDTSPFLV

KDEDAEKQYNFDLQRSHEVALTKGLLEGCKVHVTKKVKPEPSQMKDIIKSAKGEFLTSMPRSRENGVFVI

SCEDDRSVCRKPMEAGIPVVSAEVLLTGVLRQELILEEYKLFADEILDTSQDTSSSSGQSKRKKETLGEV

ATSSGTKNSASKRRKR

>Nematostella vectensis

MDLDATQAIEWDGCEDTDDTAELDRTRTKKLIGHLKVFQHHQTEEKIFEIYEGDNFIGRSDCEITIPQKS

LSKKHACIEAHEDLLLVYDNGSKNKTRKDKLILRPQVRYQLNDSDMLTFADVTCQYLHGVLQVDDNDDTG

SETGSESMLPTDSEQQRAGDDNGRDSRDEDMTYAADTEPDTDDEGQKGEKAELNCRKVSDKDNQPARPVI

PAVLYDSDQELPTPSRRNQTTVLESASDSEEPAGHSLDKHSSVPAAPERTIIEATPDRPEATPLTLIYSP

DTPTSHHKLQGVTQTPSCPPTLVYDKNSPRKSIGEIMPSCPATLVYEKSSQQKGSEETPSYAPTQMYSSE

HTTGSLQSTQPTAEPPIDKTPSKTPSKVKPHGVGDHVVYAPNTQGFSPGEEEELGLEATLAYAPKSPVDN

PSVMQLPESGDEGKVGIDASDNSATQEYRLDETNEEEDEDLALLPTQAYLAKDVQDSESDENLFATEAAR

DMPFVTSKKPPPRETNNNKAAKDNEDDDVTDDDDSDDANDIGVQPTVPYNISNMAIHAYEAVIGRSGDDG

DDDGATDDDDADNIGVQPTVPYDIRKTKKNPTNEDIKKQTGLSATTATVPYGGEAATQAYGNADDDDEKN

TNQAETSIQVQATVPYGIEMETQTYDPATGHNDDDMTDDENNKNFAVAATVPYDLETQAYGVADDDDDKS

DDENDKITKPNNPLLKREQSAMPSTLPYDIEMATQAYSAGDANDDDDTDSEMDTNAKRTKTSQPKDGENT

QKGENISPTLPYCLDTQDYGDNNSDTNDELQEVKRLSSEEHEDNIAASILPETQAYGVDVSPAADDADGE

DDPSGDGNESNDISESDGLGDTQPYGADEDIIPPSTTKETTEAEELDFPVATRLTFGETDDSEEDFRKPQ

GRQKKANTITTVPRRTPARKNKGRPAKRLDDEEEEQSTAMAKRAKHFDVDVEESPASSTTETSNRGKPAQ

HQDNEEDEPENKVKRNKGKPARLQDHEEDEEPVASTSATGITKTPGKGPNPKSARSTRSSRKKKNEKEEE

DDDLSVPSGECEDEKGKMNSMMANKSTARRRAKRDHSEKDIVEQHDNDSVTGDLETTKRIVRARMTPATT

TRNKRSACLQDDYKEEAVMGQDCGRKSSARGGRGRASKGKDNSEATNFVQKDVTNKDNVETEKTTFLTGN

ENGPQSSGRSGRAFKEKNNKEVKATTVEQQEEKKLPSRGSGRGKATRGKNNEATTAENVEEMTKEEPVEE

GTTSLTEDDTGKKSTRRRGRGKGASGRHNKEVTTTHQEEQINEEESDEEEVASPVKRKGRGKARKSPQKI

TSHGTSSTPAPNSTPAKRTRGRGVKVKDEPETPVISSSSATSQTEMSLPDVTPLMPIFGRRGRGRVTLSS

EGPELPEPFDDTPTPSRPEPGPLSVVKAISRRGKRGGRVVKTKDELLPDSSETLDLNEDTRSETPSEATS

EAASETHSEPPQVFTTAYTRKGRGRAGKDSQASTDSTLSTGGGKGRAKKPKENRRSPSSSQTADSTLVDS

PSAQAERPKGGVVLKRDSQESQEVQATPAKKGRRHEDGAAEAPSPSLRERKPEGKPRVMFTGLVDKQGEK

VVTSLGGSLVNSVYECTHLVTEKVRRTVKFLCGLASGQLLVQPAWLEACKLAKTFVDPSPFFVHDRAAEK

QYNFKLHESHQRALEGGLLQGYRVHVTKGVKPEPSQMKDIIKCAKGEVLPRMPRAKDDGILVISCEEDHQ

ACKPAVNAGVPVYSAELLLTGILRHQLSLDENRLFSDGSAEESPSNQTRKRKNDTHSGDAPPKTARASRR

KKK

>Hydra vulgaris

MEDLDSTQLIYSEKEPSADSHDIRTKVGQLEILSEKKEVFPLYEGPNTIGRDKAANISINHQTLSKIHAC

IHVNDGNCSIQDNQSQNKTYIGKRVLYPWQLYQLFGKENIITFGDLNCKVIISSETICDDTESLTASEPD

SVSMIPDDDISLDAIFDSTFDDEESKFVSKDSNVICESPHEELVSSCKLVSCIPNQSNNFSELFLKKNFL

APKIMVGKIISEFNGTLPVGTDCCALPISQDAFTKVADKFSEIKNKEDLGMLKGSQEMFSQEMCSQEFKY

PLSVKHSKTSVFFDFSMDSPTSSIQNCNEKKSLEDEPKKVLTSPCHKEDLNNFINNSLPGSSLIAQDLIK

LPAFSNQNEFQEHSVESNTLDDMDMLATQAYCMTENFHDSQYANIETQTPSSASASMEPNISSATQDFCM

ISQKYMDHVEVDNDENKGNEDNAANNGEDNSVASNELCNFANIEEGKSVSNNRYGDANKVLSDSTANKED

SDNIANKGDSDKEDSNKAAYEKDCISVKTNENDNSVAKDLKHKKYVHNPKKIQTRKSLKSYVCVANDKEN

DNVKMLNNTLKDSIQNKSHIQTRKSLRTICKRNDYSGSTFERVSNKNCEDVEQSSIVDLCTNELNKNSID

SVQSAVEILEKDLNNTKSIFKLAGEDFNELSKKVKDTKRKKSKSINKIELNKIKTNYKEKENIEKIKKET

NSNKNLTNKSIDFTDINLEMKICLEEVDDNKPKNRRGKKSIYSSKRNVEKEQHLICEEKKTIKKEPNESP

RESCFTDVIDLGKNQNLKLLKQKETIEFSPQDIKPIIKRKLIENDPEKFVVCKRKTIDKSKSENSVIAKN

LSSNKENSFNNLSSKIVSKRRLSSSKPKVVFTGVTDKNIEKIVKELGGELVDNVGSATHLVTDKVRRTVK

FLCAVARGIPIVSLEWLKAGKTASMFVPHHHHLLKDNDAERQYNFDLVESLETAAHTHLFENVQIYVTRN

VKPEPKAIKEMIEFSGGKFLHKAPTKYMENTYIVSCIEDHKEILKFEKLNYRILSNEFILTGILLQKFSP

DQYYLT

>Dendronephthya gigantea

MDATQILEDLDEEDINHSGTKKTVAFLKVFAVGSFPETLFPVYEGENVVGRHNCDITLPLQAVSKKHACI

DVHGKNHLICDYNSRNKTRRNQMFLKPQAFYELVNEVELMFADVKCQYLLAQTEDVVEKEIADSGSETAS

ESMMPESIEDEDEELKEEPRKESLEKYLDQPTLEYDYENPGEHSTLFCAETDDDETDDDKTDDESETNDN

SNNTSCNTKPLFSKEKTVEYSAEATANDSTLETTMPYNTIPNTQEPDVIPETPFPQTNSTESPFKLKRSV

DVKVDTSRRVSAGNTLAYGLSTLESPADTTRESIDVSQNDSAKSETSGECEKVRNKLLFDNTPDKESPRP

SCPLPKQDEAFDDLLATQLYEPMEIPESPEYLESDKNDEKLTTENTPKTTARETPEDTIPNCKNQGPENK

FSEDKISEGGNPENKVSDDKKSYGRDSGNVTSEDTIPNCSTLEETQAYVLPQDEYDDMPTQAYGLLDTDT

AGTEDEMAETQAYGVESDLEIPKLAAPTKSVFKVPGAMNTQDLHKDICDGGGAKEEYNDEMDFQPTQKYV

EETSESSDPDNSLNVSRFLGLLSESDSDFMSTKEVVKPETSTPAVRKSSVVIEGGKTLSTGKRCRVELPE

DEISDVKLQTSLLDPSAENDDFVPKCKIMREGKGVARRSRKGRKGELESNDGSEGKSDSKKEDAKPVRKG

RSRKKQELDIEEDERDETIDDEKHTVSEKEDTGVKHTKKGRGKEIKDIEKDERKVSNKDTPGQHASEERT

SLGQDLTDTGTPEASESKTSGKKGRQATAKTSRRKVGSKFEDVQGVDVNEDIVVDEPKKIQPRTPARKRK

SSENEQQKKGTAKKSRRNTRTEVISPENDGMKFDTEDILPPTSSLVKRSSSTASTSSNASSKVSRSGRKG

KKQMKENSEVLDDSADVKIVFTGLQDSAFNKANKIVQALGGKVVEHLEDCTHLVTDKVRRTVKLLCAISR

GIPVVDMCWLEASKKRKSFADSESYILKDDDAQQKFSFSMERSLKLARDDSLLDGYKIHVTPNVRPSPPE

MKEIIKSAKGEVLTRMPTTRDAKNDNVIVLSCEEDKNLCIKAKIRKAYTAELLLTGILKQQLDLEPYELP

LNTETKPSKRRQKSLK

>Paramuricea clavata

MDATQVLDDFDEEELNNSGSKKTVAFLKVQAVGDFPETLFPVYEGENIIGRHDNCDITIPLQAVSKKHAC

IDVHGKNHLICDYNSRNKTQRNQMFLKPQAFYELVNEVELSFADVTCQYIFAQTEDVVEKDTVSDSGSET

ASESMMPQSTEDDEEPEEAKKEKVADSPKNEEPEESVEKYLNQPTQDYSYKNPDENSPVFCAETDDDETD

DERDNKDETSPGIGSLSLSKEQTPQHSLESNDPTTAATMPCNTLPNTLEPDTIPETPFTQSNDFEPPFKL

KRSVDVKVDTSRRISAGNTLAYGLSTLESPNSTRESIDVSESEKEELSCEGTSQVKNKLLFDNTPDQRSP

NNNDKRLPDSMSKQDEILDDLLATQLYEPMEIPESPEFLQPFGTEESTEIKDKEPPENKTPEGEKISEGT

VSDCQTSEGKTSEGKISEGTVSEGQISEGETSKGKISEGTVSESQTSEGKTSKGKISEGITSEGKISEDK

IFDDMMETQAYGLDTLPQDEYEDMATQAYGLMFDADSADTGDELEATQAYGDDIEVPKMSGIFKVPGAMN

TQELNEDGSIGARDETAAHNEKVGNEDYDDEMDIQATQNYIENQVKESGETSESSDNFPSLSSFMGLKSD

SDSDFKSAKKSSRRKVETKAPPQEVVNESLTSAAVKSSSSIVIEGDNPISTEKRCYVECPGVEDETSRDK

EHNDRRTSTRKKSVRGGKKSAATRSKVQDEPNEDTAGVSTTPAKDDLPTTRNDTMTPEDTDEQLETIVVK

RGKSRSTRGKTSAATTRSSRGKKNEAVAEDVAEEAEVESLPKGRRSRRKRKISEGNPKTESKNEAESSIV

KGDVENMETENKAEKLNMSDDEENNDMFKLQMSAVDSPETDDFIPKKKPVLETKRPARRGKKTTTEELES

NDESETTNVEVEKQLAPRAKRSVSTKSARKARGKKNQDLAEVDNDENVTESVSKQAEMIDSNKDGQTLGN

QKDSMVKPTKKGRSKKSQGVTEVDSNENIREDVAKQDEMVEKKKGKNTQGQQKDFVEKSTESVEQELPDV

MTPVVNTRISRGGRKGKQTTTKKSRNGASGNAVQNLEQVQDKNEMDQPANNMNETKMKEPKTPARGRARK

RKSSENSNETTQQKKGTAKKSRKNTTTEENSETKDETHHGRETTASPQDDIKFESPELTPRSLSKRSSST

ASTSSTSSVSRSARKGKKQEKEDSEGSVDHSSNVKIIFTGLQDNAFVKAKKIVQTLGGKVVERLEDCTHL

VTDKVRRTVKLLCAVSRGIPVVDMSWLDASKKSKSFVDSSSFILEDKEAQKKFNFSMERSVELAQNAGLL

DGYKIHVTPHVRPPPNDMKEIIKSAKGEIVSKMPTSRDNDVIVLSCDEDKELCEKAHVEKAYTAELLLTG

ILRQKLDLDQNRLTFSTTADINSDLNTPVKSKGRRRR

>Actinia tenebrosa

MDLDDTQALDLDEYDETEEEQEVKRNRKQVGQLKVFRTGDFEEKIYDIFEGENFIGRDER

TDVYIPRKSLSKTHACIEVSEGLHLVYDDNSKNRTRRDKMILKPQVRYELHHGDMLTLAD

TKCQYFKGKMEQDNEDNDTGSETGSESMLPVGSAIHVSKGTSKEKDVFAADTDSETDDEK

NPVNIKKPAMYSSDEKLPQIDAIENSEQPKPIVPAFMYDSSDDDDHGDDKHCTKSSNDIK

THADVSADSETLAFGLISPDDLPIIRPNSKKVPEVENTPLTLVYSPDTQDRTPDHPSILP

KGKEEGSSCPPTLIYGSESPYQEKNPVKEKEGSSQPEYSNKRDFVKMSTDEPDTPDKMHV

KTEIASCAETLPVQEIESLEETPVRDVEPTMLHGAGSLEETPLKDMPKLGETPSEFNVYL

PETQGFSPGNEETSFCEPTQAYLQESPVDTPKETPGEMVSDVTSIPDSQEEEGEEEEVKK

TLSYDDDTATQAYTWDNSDATDDGDDDELRFQATQAYPSLIQQPKKDDDDDDIQQGADVK

DSSPCASSSPHADMQQTIAYDVAEMETQVYVTERENVSVDHTKGKGKHPEDKPVEIEPTL

PYNIMDMETQAYEAETEETTSPASNSLNKSSTDDEDKGTPEELKMSAVVVGDRRGVTIEP

TLPYDLETETQDFDNNEKDAQETSRQTSKKNAEDDNLVIEATVPYDMEMETQAYDRKDID

DSETDDECNLEKKEGNMADMMETQAYGDEVPVEARENAINTFNMADMMETQAYGVEVEAL

ENAINTGASDVNDADGEPQAYGLEDQATVTDDVIPDSEDLLSQEIPLKRISEDEDTVNNN

ASVKTPTIQHDKQQVEQSGNKEEEKEEEKEEAEIIVRPKRRGKSRVTHSSPEEENQQPAM

LNKLEDTTTAKTAEDISLNEQATAPEDVGVQRSKRGKRSKKNKVETEIQDTLGDASEEEQ

FKIATKQEKSTRERKSTRGKTCKISKTDDTEIEQSTSMSEKRPTRGRRSKKSEVEIRVQG

ILPDDTEEEQSMLEKRPTRGRQLKKSEVEPSRVEDVLPDNTEEERQESPSTTKKRPTRGR

PKKTVVKTAMIEDSLPEDTEKDEMSLQPVQTTHNEHLPEKKSLRGRRSMKTGKELDIEEK

IKPVDNAEDKNKSESVPQKRVQQKMSTRGTKKDEEDRLEGTGSGDNKEKLDTCAENLDIS

PGHSEEEQFAVKHKGKGKDKKSLQVDQDSSCKDEEELPSLEIIFSDKKVTKGKRKGKTKD

AETPVLLSGKDTSTAEAVRNEGMSFETIEVKPFFPRRGRGRVTQSDDSASAVPGSFGSEL

SEFKEEDFMSPQSVAKSISALKKEKKKKGEEQNLKETEMDINATQSNSSSPRGSKTRKRG

KEVRTDLDESQSSVTSSVGREARGKGKGKKAKSTKVDDTHVHSASQASSDLSESQNSTAS

SGESRARGKGRRRTGKPSTLDDSQEQEVQETPVKKGRTVKQEDSPTVVHSPSLRERKPIT

PKIMFTGVSDKQAEKIVTSLGGILVDSVYECTHLITDKVRRTVKFLCGVASAQMIVQPQW

LTACKKAKCFIEPVPFIVSDEAAEKQYSFSLQQSLERAKKGGLLEGYKVHITKNVKPDPS

QMKDIIKSAKGEYLPRMPRTMENNILVVSSEEDRDLCKAALDCSIPVYSSELLLTGVLRQ

ELDLDQNRLFTGEKEDRKRKESEDEETSVKSASRKRRKR

>Macrostomum lignano

AASFPCTQVLCTQQFDACADSDNDDNNGSADGRGQPLAQLVLMGPAGGAQHPLYTGENTVGRMDSCDVRI

EDPSVSGRHACLELGRGQQLVYDLHSTNRSRKNDMPLKPEIRYDLRDGDQLRFGFVMCRFELLKPPAKPS

KPSKSPQPQSPRLLAEDADGGGDETREALLLEQSQFFVPTTPPPAGEDDDNQETDIEEDAGTSTAAAADG

RPPAATVLLAEDSHNSNVVQDDEPPAHQQCATAAAAVVVPDSILLMQSTERLHEDDADVQGASIAATEPL

PAATAAVVDSSSTSLIAAATDTLPVQTSRDVSLAVPTEAIKETAPLPDSSALDETLPLTTASPADAVAVT

TATAVRAATGAFRLFGDVPNGNASTVPKNSFAGDSALFSAETQRLDWAAPAASAAAATAAAAEDVGDDDE

NDAALFGMETQALPSGSAVTPPAAAATEADKAKQLAEFDEADVVAETPCKAAESEDPQLLSQATQAAVSD

QPPSDSLSLPIDRGLVGAVRRPRIDSSPSSSSPSSPESAAAAPAVSGSAASGSSGADRGGWLSKAHLALP

LTAKPAAASRGGPGGGRGRGRGRGARQSVTTGSDNAEPEVAGSDAVRGGRGRGTRQSVPTTKDSPEPEVA

RSDAVRGGRGRGRGRGRGARQSVTTGSDNAEPEVAGSDAVRGGRGRGRGRGRGARQSVATREDSPKPEVA

GSDRGKISQQAGQTGCASTKPEVAGSDAVRDGSGRPAKRSRAASSLPDAGSCSADAAGGEAVGPGSPAQA

KSIKLTTDPAPGDSASPASISARRSARGSPSPSPGRPTRASASPCNALAGAGPCGSPAATKTASSDVGEI

QADRLSMSQTKTSSAASEKGESQVSEASESQTSEAGVTKRARGRPARGRTTKTADLDEPSDASTSTSQAA

ATTVPKKSRGRVTKSQTKTGASAEAGPAEDSAKQEPESTFRGRGRATKSQTKTEATEAKPAENSVNQESE

SVVSKKSRGRGRVTKSQTKTSAPAEEPSIDGAASQSASERSSTTSRGRRGRQTKTASVDDDDDGASSTRG

SEVGSASNRRPRKRATSVGDEAEADEPARKRSTSRKAAAAAADAADSVAVMFTGVDEATLAPIVRRLGGR

VTEVPSEATHLVTDRVRRTVKLLCCVARGVPVVSVDWLTDSRSAGRFLDSAAGYPVAGEADFGVNLTQVC

ERARRGQQLLSGQTVAATPSVRPDPASLAQIVASAGGRFVGVGAAAFKACSLVFTCEADAKALARLPAGV

TALQVEALLSGVLKGRLDTEAFRMR

>Rousettus aegyptiacus

MEDTQAISWEVEEEEETERPSESLGRSLEPLGRLHIFSSAHGPEKDFPLYLGKNVVGRMP

ACSVALPFPSISKQHAVIEILAWDKAPVLRDCGSLNGTQILRPPKVLSPGMSHRLRDQEL

VLFADLPCQYHRLNVPLPFVSRGPLTVEETPRVQGRTQPQGLLLAEDSEEEVDSLSERCV

AKEPRTTSSPVATVVPESDEEGPSLAPDGPGPPFTFNLDSDTDKEENQQRAAGEASSAAT

RDATAETEQPKAVVTEIQLEKNQCSVKERNNDTKVESDVRNGVVPTGVISERCQPAGEDS

ETDVDDESRPPGRPVDVHLERAQPFGFIDSDTDLEEEGVPATPAIDPVKRKQVLHGVSTN

SPGGPDLAHLQETLADSDTDVEEGEASLMVPLERSQASMVIDSNTDDEEEVSAALTLACL

KESRAVVWNRDTDVEELQHSTSMDQPVSSRPTSRATQDRTHRSSVKTPQSIVLTAPELQS

STSKGQSVTTELISGATWGKVHRSFVKTPEPIVPIVPELQPSTPTDQPVTSKPISRGRTR

RSSVKTPESIVPTAPEVQLSTSTNQPVTPKPTSRGRTHRFSVKTPEPIIPSAHELQHSTS

MDQPNPSRPTSRATRVLRRSSVKTHQLVESTALDLELPNPTDQFVTPKAIAQGAQSRTLR

STTSSVLVSTKLEFQPPTDQPIPPEPFPPGNCSGRARATRKHGPLSVPIVHEPCSAPPEP

NSRSSRNQRRGAVRAAKSRRTIPEPAFAQLPEAPTHAPQIQKAEEVGIYKFTPEPQPKVS

QNHKRPLGTADLLPLQKRLQRGEVPQKTAFLKEKEDPAERPGKEEDVVITGPGKRKRDQT

EEEPEGMPGRSLRRTKLNESTAPKVLFTGVVDARGERTVLALGGSLASSVAEASHLVTDR

IRRTVKFLCALGRGIPILSLDWLHQSRKAGCFLPPDEYVVTDPEQENNFGFSLRDALSRA

QKRRLLEGYEIHVTPGVQPPPLQMGEIISCCGGTVLSSMPRSYKPQRVVITCSQDFPRCS

IPFRVGLPILSPEFLLTGVLKQEAKPEAFVLSTLEMSST

>Vulpes vulpes

MEDTQAINWEVEEEEETERPSEALGRGLEPVGRLHIFSSAHGPEKDFPLYLGKNMVGRMP

DCSVTLPFSSISKQHAVIEILAWDKAPVLQDCGSLNGTQVLRPPKVLSPGVSHRLRDQEL

ILFADLPCQYHRLNVPLPFVSRGPLTVEETPRVQGGTQPQGLLLAEDSEEEVDSPSERCV

VKEPRTSPLAAVVPESDEEGPSPAPDGPGPPFAFNLDSDTDEEESQHSAAGEASLSARRG

STAETEQLKAMTPATQLGKDQCSVKERNNNTKVERDARNGVVSLGGILERNRSAGEDSDT

DVDESRPPVRPAEVHLERAQPSEFIDSDTDVEEERIPATPAVVPMKKRQIFHRVSTKSLQ

EPALVHLQENPAGSDTDVEESEIQPAVPLERNQISMVIDSNTDDEEEVLAALTLARLKES

GSDTWNRGTDVEEDRAQPVALLEQSQTSAGRDSKTDMEEEGLPMENRRTVPKCHTDKACS

EKRQFPLQDNDLEVDKSLLEVHLERNQASATIDITQVEEKVLPGPAVILVEKHQVPVVWT

NQTNVEVEGDQARLPVMHLGEPQPPLSEDCGTDVEEDTSLAASLVADTGKHQLLAEGDAG

TESAAPVLEQERVLEARAQGDSLVSQVEQDLLPVSKENLIDQVVDTGTSGETIQPQREGA

QTPTERKREPHVDRTKNSGDNHGDSEDLDLQATQCFVERENQSPEVQNMEDEATQAFLAT

LPQESGPSCYSFQASGHLTCKAPPAEKASVGDQESADAHLPAAVPEASTPHHNPLLSQSQ

KHPVSQPFLSSSPSSLEPIPRTRQNGNQEVPGTPLSSEMEPLYPKSRVRLRGSSRKALSA

ISSLALEPHSTIPTDQPLCPEPTSRVTRGRTRRSSLKTPELLVPEVQPSTSKDQSVTAEP

ISQGRTRKSVKTPEPVVSTATQSRALRSSVKTPKVDISTTPALQPSTSKDQPVTPEPTSQ

VTWGRTRRSSVKIPEPTVPTAPALQPSTSEDQSVITEHTSGGTHSRTRRSSVKTPEPIVP

TAPEVQPTISKDQPVTPEPTSPITWGRTRRSSVKTPEPTVPTAPELQPSTSKDHSVITEP

TSGATHSRTHRSSLKTPKPTVPTATELQPTTHKGQPVTPKRISQGRTPKSSSKTPTSVVS

TVPELQACTPTDQPVTPKLAFQATRGRTQRSSIKTPETVAPTVPEPQPSISTDQPVTPEP

TSRATRGRTHRSFVKMLQPIEPTAPDLESVTPTDQLFSPKVQGSQGKMLRSSTISAVPVL

TTPEFQPSVPTDQPIPPEPISQANCSRRLRATRNHKSLIAPIICEPYSALPEPKSRSSRN

QRQRAVRAVESLRTIPKPAFAQLPEAPTHATQIHKLEAAGRSEFTLGPQPKASQSHKRPL

ATVDSPPPQKRHQRGEVTQETVFLKEEEEDPMERPREEEDVVVPGPGKRKRDQAEEEPKG

IPSRSLRRTKPNQESTAPKVLFTGVVDARGERVVLALGGSLASSVAEASHLVTDRVRRTV

KFLCALGRGIPILSLDWLHQSRKAGCFLPPDEYVVTDPEQEKNFGFSLRDALSRARKQRL

LEGYEIHVTPGVQPPPPQMGEIISCCGGTVLPSMPRSYKPQRVVITCSQDFPRCSIPFRI

GLPILSPEFLLTGVLKQEAKPEAFIFSTLEMSSS

>Thamnophis elegans

MQGRMEQTQLLWDGEGDSTDGSDAARETPNPVGRLHLLSSKYGPEKDFWIYPGENTIGRLESCHVCLPAN

SVSKVHAVIEVPSSHGPHLLYDQGSLNRTRRQRMNLIPQVRYSLQDGDTLLFADVGCQYFILVPEADYDS

HDDSMAVPPTQGRVEASALVIEETPAPGRKIGFGQVLVQDSDKEEEGEEVINGTGSYLHHASKDESGCSN

KNGTGEGQASLASFGVSSLSATVVPESDDENGDPPVGGSPCPALRLSFESQEAERSPVEDGGSPSSNRED

CGEPIDEEEAASAEHGLVEGFHLDSDTDVEDENQMNRISEVPASLDPEKRDRAPELDPDTDLNESSLVAP

AVHCPQTPMELGSDTDAEESVESPAVSRLKLPSASMEKEEDDTNAEAEVENPSVVCPGKHEPVPPKEGAF

KQLEDNEDNAQLGGRKDDDNTDVEEDLDNPESGVNTLPPTVHKDVDTDAEDSSVKGETPGRTKNNGVGGH

SDSEDVDTDVEDSSAKGKTPSRTKNHVVSGHSDGEDVDTDVEDSSAKGKTPSRTKNRVVGGHSDGEDVDT

DVEDSSVKGRTPNRTKNCVVGGHSDGEDVDRDVEDSSAKGKTPGRTKNRVVGGHSDGEDVDTDVEDSSVK

GKTPGRIKNCGVGGQSNGADTKEETIKPQLENSEDTCAQRTCSSSGKESSTDMEEVSLKKIESGGHSVSE

RHGDVVGVTKNSEVELEMSYEDSDKSPRDFDKKEPSPNPMQPNLPMLFLGSDTDEEEEAGNPDVESKKRP

PVSGAESSKGSRVSCLGDSDVGSREKPPDQDKDSDINEEVTSVGHDKSRAQDSDTDTKDVAPFLLRKPLE

VKSFQIIIPGRSQVEGEPLASSAVGIGGAFKPREDNEDTDAEREESYSADESNTDDELDVSLQATQCYLS

TESTPSGPEKAGGITATVSDSDCALEEEPTQSFDFSPHFLPTKHQFRNVTSSPLKDNSSDQEDDILEATQ

PFCEEPEQVSAEPCEALTGKEKRLAQDQPEEDKCFEPVPQPDFPAATSPGEETTSSLPGDHLPPQAVALP

SMQSPLQTPEGAPEEDPQPLPSVQIPLVASKGSLTPQEEKRVEVLGSGSPSSEVVPRQSEATPVGDGVEE

NSELVRETRNTKKVLVPTQEDPVSATLPARELRRSLRSSTTSPSPTHVPERRSLRRHGSGAVVASSTPAA

PETRRRGLRQLSNASQRMRIVKERPQQPEEEEGPRKKARNEDLNAAPIPQGRTRSSQRESTAARKVTEVA

KAVSGSNPGAREPTKRVRGAPAPDRGLEGQREHPRTRASKLSSSSSSASVSTPSPKDSGKDAKPAQEPVS

TPSSGRRLRCHSPESKATSQKASSGSRSRRATGPGSPAPKVLFTGVIDEEGERVVAELGGTLAESVFDCT

HLVTDRVRRTVKFLCALARGIPIVTLDWLEKSRRNAFFLAPNNFLVRDPEQEKNLRFNLSTSLQTAQQKG

ALFQGYEIHVTPNVKPEPEHMKDIVKCSGGTLLPRMPRAFKEKRIVVSCPEDLLRCKPAQEARVPITNAE

FILTGILQQTVDLEAHRLDGNTGPSPASSPLVPSTRTSKRRAATQTAPAPPSTAKRRR

>Crotalus tigris

MEQTQLLEWDGEGDSTDGSDAAGETPKPVGRLHQLSSTYGPDKDFWIYPGENAIGRLEHCHVCLPASSVS

KAHAVIEVPSSRGPHLLYDRGSLNRTRRQRVALIPQVRYSLQDGDTLVFGDVTCQYFILAPDSDCDSLEE

SMAVPPTQERVEASALAIEETPAPGRRIGFGQVLVQDSDKEEEGEEVINGTGSNLPHTARDESGCSNKNG

VGEGQVSLASFGVSSPSSTVVPESDDESGDLSASGPPCPALRLSFESQDAERGPTENGDGHSSSRKDSGE

PTAEEEGASTEHGLVEGFHLDSDTDVEDESQMGCVSEAPASLDPSKRDRDLEVDTEPDLNESSVMAPEIH

CPQTSVELGSDTDAEETMESPAAFRLKLPSTSLEKEDDDTDMEAEMESPSVVGPGKHKPVSPKEDDCQQL

EGNVQLGGEKDDDTNLEEASDNSESGVNALPPTAHEDVDTDVEGSSVKGEAPGRTKDHEVGGDSDGDDTE

EEVINPWLENSEDTCPQRTCFSSAKKNSMDREEIHLKRIVPDDHRVGEGLSDVVGLTQNSNLGLEMSYEV

GDKSPRDFDEKEPLPNPEHHNLPLLFLGSDTDGEEEAGHPDAESKENPQSSGVESSKGSRVSHLGDPDIG

SQEKPTSVQDKDSDTDVEMMPVGHDKSTAQDSDTDTEDVAAPLLRKPLEEKGAQSIIPVKSEAGEEQLAS

SAVGVGVSLKSREDDEDTDAEGEESYSADESNTDDEIDVSLQATQCYLPTETTSPRPEKAGGITATDSDC

ALEEEPTQAFDFSSPFLPAKCQLRNVTSLPLKDNSSDQEDDMLEATQPYCQEPEPEALAELCEALAGKKK

LDPERLVQNQPEGEKCFEQVPHPDFPASTSPGEETTPSLPGDNPLPQAMELPPPQSSSKTAGGASEEETQ

PLCSLQIPLVASQWSLTPQEEKRVEVPEGGSPSEVMLRQSEATPLDDQVEENSELVRETRNPKKVPAEKD

PASTAPPARELRRSLRSSTASASPAPVLERRSLRQHGSGAVVASGKPAAPESRRRGLRQLSKASQRKREV

KERPEQPGEEGPRKKARNGDLTATPVPQAQTRSSQRESRAASKVTEVAAAVSVSPGAREPTKRARRGLAP

SPGLEGQPERPRTRASKLSSSSSISMSTPSPKDSGKSAKPGQEQILSTPSSGRKLRHHSPEDRATSEKAS

SGSRNQRSTGFASPAPKVWRVLFTGVIDEEGERVVAELGGSLAKSVFDCTHLVTDRVRRTVKFLCALARG

IPIVTLDWLEKSRRNAFFLAPNSFLVRDPEQEKNLRFSLSTSLQKAQQKGALFQGYEIHVTPNVKPEPEY

MKDIVKCSGGTLLPRMPRAFKEKRIVVSCPEDLSCCKPAQEAGVPVTNAEFILTGILQQTVDLQAHRLDV

RVGLSPASSPLVPSTRTSKRRAATRTAPAPPSTAKRRR

>Notechis scutatus

MEQTQLLEWDGEGDSPDRSDGAGETPKPVGRLHLLSSKYGPEKDFWIYPGENTIGRLESCRVCLPANSVS

KTHAMIEVLSANGPHLLYDQGSLNRTRRQRITLIPQVRYSLQDGDTLLFADMGCQYFILVPDTDCNSPND

SMAVPPTQERVEASALAIEETPAPGRRIGCGQVLVQDSDKEEEGEEVIYGRGSYLHHASKEESGCSNKNG

TGEGQASLASFAVFSPSTTVVPESDDENGDPSVVGPPCPALRLSFESQDAGRTPIENGGSGPSSNREDCG

EPTTKEGGASAEHGLVEGFHLDSDTDVEDENQMDCISEAPAKRDRGPEVDPDSDLNESSVVAPAACCPQT

SMELGSDTDAEETIESPAVFRLKLPSNSMEKEEVDTDVEAEVENPSVVCPRKHEPVAGDCKRLEDNEDNV

QLGGRKDDDNTDVEEDLDNPGSGVNAPLPAAHEDVDTDVEDSSAKGETPGGTKNRGVGRHSNGEDVDTDV

EDSSAKGETPGGTKNHGVGRHSNGEDVDTDVEDSSVKGKTPGRIKNRGVGGHGDGEDVDTDVEDLSAKGE

TPGRTKNRGVDGHRDTEEEAIQPRLENSEDTCPQRTYSSSGKESSTGKKKVPLKRIVPEGHSVGERHSDV

VGLTKNSDVELETSYEVGDKSPRDFDEKEPSTNLAQHNLPIFFLGSDTDEEEEAGNPGVESKSQQVSGGE

SSKGSRASRLRDPDIGSRGKPSSVQDKDSDTDAEGTSMDRDKSTAQDSDTDTEDVAASLLRKPLEKKSSR

LIIPVTSEAEGEKLASSAVGIGVSFKSREDNEDTDAEGGRSYSADESSTDDELDVSLQATQCYLPTETTP

PGLEKAGGITATDVSLQATQSYLPTETTPPGLEKAGGITATVTSSPLKDNTSDQEDDILEATQPYCEASE

QVSTEPREALAGKEKADPERLIQNQPEGDKSFEQVSQPDLPAAASLGEETTSSLPGDNPLPQVVEFPSLQ

SSLQTPEGAPEEEAQIPLVVSKGSLTSQEKKRVEVPRQSEATPVDDRVAENSELVRKIKKPKKVLVPTQE

DPASATPPARELRRRLRSSTASASPTPVPERRSLRSSTPAAPETRCRQLSNTGQRGRGVKERPEEPGEEV

PRKKARNGDPTAAPTPQGRTRGSQKEPRAASKVAEVAKAIPGSAGPREPTKRVRKAPAPGLEGQAERRNT

RASKHSSSSTSVSTPSPKDSGKGAKPGQEPVFTPSSGRRLRCHSPESKATSPKASSDSHTRRATGPGSPA

PKVLFTGVIDEEGERVVAELGGSLAESVFDCTHLVTDRVRRTVKFLCALARGIPIVTLDWLEKSRRNAFF

LAPNNFLVRDPEQEKNLRFSLLTSLQKVQQKGPLFQGYKIHVTPNVKPEPEYMKDIIKCSGGTVLARMPR

AFKEKCVVVSCPEDLSRCKPAQEAGVPITNAEFLLTGILQQAVNLEAHRLDGSGGLSPASSPLVPSTRTG

KKRAAATRTAPAPASTAKRRR

>Pantherophis guttatus

MEQTQLLEWDGEGDSTDGSDAAARETPKAVGRLHLLSSKYGPEKDFWIYPGENTIGRLES

CHVCLPANSVSKTHAVIEVPSSHGPHLLYDQGSLNRTRRQRMNLVPQVRYSLQDGDTLLF

GDVGCQYFILVPDTDYDSHDDSMAVPPTQGRVEASALAIEETPAPGRRIGFGQVLVQDSD

KEEEGEEVMNGTGSYLHHTSKDESDCSNKNGSGEGQASLASFGVSSPSATVVPESDDETG

DPSVDGPLCPALRLSFESQEAERSPAENGGSPSSNREDRGESTAAEHGLVEGFRLDSDTD

VEDENQMGGISEAPASLDPEKRDRGPEVDPDTDLNESSVVAPVVRCPQTSMELGSDTDAE

ETVESPAVFRLKLPSTSMEKEEDDTDVEAEVENPSVVCPGKHEPVPPKEGDCKQLEDNVD

NVQLGGKKDEDTDVEEDLDNPESSVNALPPTAHEDVDTDVEDSSVKGKTLSRTKNRVVGG

HSDSEDVDTDVEDSSAKGKTLSRTKNRVVGGHSDSEDVDTDVEDSSAKGKTLSRTKNRVV

GGHSNGDDTKEEAIKPQLENSEDTSPQRTCSSSGKESSTDMKDVPLKRIIPAGHSVGERH

GDVVGLTKNPNVELEMSYAVGDQSPRDFDKKEISSNAMQHNLPMLLLGSDTDGEEEAGNP

DVESMKSQHISGAEPSKGSRVSRLGDPDIGSLEKPSPGQDKDSDTDAEVTSMGRDKSTAQ

DSDTDAEDVAPSLLRKPLEEKSAQIIIPGRSQAGGEHSSTVGIGVSFKSREDNEDKDAER

EESYSADESNTDDELDVSLQATQCYLPTATIPSGPEKAGGIPATVTSSPLKDNSSDQEDD

MLEATQPYCEEPQQVSAEPCDKKKLDPERIVQNQPEGDKCFEQVPQPDFPAATSPGKEST

SSLSGDNPPPQAVELPAMQSPLQTSEGAPEEETQPLGSVQIPLVVSKGSFTSQEEKREEV

PESGSPSSEVVVPRQSEATPVDDRVEENSELVRKTKNSKKVLVPAQEDPASATTPARELR

RSLRSSTTSASPTPVPERRSLRCHGSGAVVASSTPAAPESRRRGLRQLSNASQQMREVKE

RPEQPGEEGPRKKARNGDLNAAPVPQGRTRGTQRASRAASKVTEVAKAISGSSPGAREPT

KRVRRAPAPNPRLEGQTEHPRTRASKHSSSSSSTSVSTPSPKDSGKGAKLGQELVFTPSS

GRRLRRHSPESKATSQKASSGSRTRRTTGSGSPAPKVLFTGVIDEEGERIVAELGGTLAE

SVFDCTHLVTDRVRRTVKFLCALARGIPIVTLDWLEKSRRNAFFLAPNNFLVRDPEQEEN

LRFSLSNSLQKAQQKGALFQGYEIHVTPNVKPEPEHMKDIVKCSGGTLLPRMPRAFKEKR

IVVSCPEDLSRCKPAQEARVPITNAEFILTGILQQTVDLEAHRLHGSVGLSPASSPLVPS

TRTSKRRAATQTAPAPPSTAKRRR

>Bombus vosnesenskii

MDILATQIYEDYDSTPTQKISYSSQQSEIVIGVLSIDSKTFQIKEGITKIGRHPDCNVVL

NNPTVSKKHAEIEANCQGTESWICDLNSSNKTKLNNTILRPNRCYELKNEDVLEFGTVRA

IFKICRSMEDSLVPETPAANHQKAQQTIIPGTPDSSFDNSSTVGNVSVIPATQDKEEKSV

FRHPTLPPKTATNSTQKNGIQNTSIDAQDNLQNFDVEEKQNVGESRISIYDMETQNFSFN

FHDETDVDIEDIDTQKICISRSIKNLTKSVQEQKVDMHDIQAQAEAPEEVTDIHDIETQY

DINIQDMKISKKINTHNLKTRDSDKLEATRIEKEGNLTNEVRSNVNNTVVQKDKGNESTS

NPNNTDEGENQNKKLPGIEKGLQNRLEEIETSEDETSELEMSRNLLGPKELEDFIEDDDL

SDEVKSKSPTLIKTSCVNNNCDVNNKSTDNENIFEAATQVNKSDEDAFRSLSERKYTFQA

SLVDDDSDHTDNEAVFQRYSHKDSQESKKSLNNRVSDSEDSITDEEGRFTEIAVKMKQAV

EFSQLNRSKEIKNSANSSKDSDDLFDMLTQPVNRKDEDSSPKSNEKPKDNKYESEVDSNS

PTQVINKKEMKNNMEINDVIQTRELNDITPTQVLSTNKSSLREASCTISTGNNKKFDKED

ITEIEDNTPTQIINTKKKPLDVSNNEPPCSLDPLVLNVTDRCNIEDIDYEMACTQPINDI

EKQKSVSSISGKRKNESNFEALARVNLDDSVERNLKAMFADIKEERIEEQLEISTQVLEH

VLESSDCENISPTQNHKSNVDSNSLTDKKAMKLSISNSIPTSSSTPKSIPKNPVTSKSVS

TDSSTLKSTIKDSSTSISLREKKSKISNIAKMIQEETDISKNGTSKLNKYSKNIDDQASS

TSIDCEQIQQTSKALQSDDEDILAGLPEVNISGTLSNPESPTSTTSSEYRININHNRGRQ

TSVKIAPRKKEALRKSSRRTSTCRNMENAVSYHTISEPNSSNISETSSNSYTNNFDINKI

PVCKHDEKGTSRKSKRLIKKTKNLCKSKESDSFVELKSVTSIHSQEKARQSPPISNNRRT

RNSSKENTDRSSVFQVEKEVPVEIAPAELTKKNVSNASRVRKRSLSTTDAIDSNTSKKRK

NEVNEDKPVSNKSRKKNTIDDEGNSANVDKKNSSNSINGSNNRRISNEASLDKQAIVRVQ

RMSLSTPTESISSTPTELVNESDNNCKIRQNRLTNVICDIQTSDSRIVSSISSNENYRTT

KNVETRKNPKRKTTKKTELVADDISSQIGEESQEIEMIMNSALKKQNTGSNIERKEDTER

NTSTKAKNTRKRGNTDINTDVDNKEISSTSSILSESDNTHFEVPTLRSKRAKVSKNISSI

ISTTVNESTKRNGKLSIRLTRSQQSIVDSSLEESAEMIANQTTSNNSANKRNNFRKKQQK

QTTGKTKNKRQKEDFVEETSISETNSSIETTSVLSTPNRTRRSMSSSFAAQSPFKIKHKI

LFTGISSNDYNKLLTKLGASQVEDPTKCTVLVTDKVRRTVKFLCALALPVPIVSVDWLIN

SGKAGRFIELENYILKDLAAEAKFRFKLGKSLEKAKEHKLLKGYTLVLTPNTAPPPLELK

NIIISCGGKALLRPPPKLWPQQSVIISPKEDLTDAKKFLAKAPKTVTVQSTEFILTGILR

QELEFNEFKLI

>Apis cerana

MYKILHVKLKTVSKRHAEIEANNHETAVWICDLNSSNKTKLNNSILRPNRFYELKNGNVL

EFGMVRAVFKFYHSMDESLIPETPALSRQKLQKLIIPGTPDSSLNNSSTLGENISMISAI

QTNEKETIFRCPNPISTRTSTSSTRKNDLQNSSVNNLDNSQNSDAEKKGNVGVSRINIYD

METQKSFESLNGTNIDVHDIVTQKICLNNKEIFSKSLNGQKVNMHDIETQNEINAQENVI

DIHDVETQHDIDIHDMKTPKKIHVKSHNDKTQDNEKLETRIEENGNLIMKKENNIIIQKD

EAIASTSYFNDTNKCENKIEYRRSIKKHLQKNSQKCLQSSIATTFENEVSELDTSRNLLG

SQCLLEDLIDDDDNDDLLDESKSRSSTPSKTSCVNDDEDVNNKSTDSENIFEAATQINQT

DKDTNHRINYSFKASLMDPNDSDETDNECVFQRYSFEENKKNEKSSIIRVSESDDSFTDE

EGRFIEMAAIEKRAASNSFEKQNKNKEIRNDSSKDSEDLFNMLTQPINKKNKNLLPKCDE

KTQTTVDEVEIDFDTPTQVIDKKNNSEINKDIEIKEKIKNTEVEQVDDFTPTQVLSTLKS

SSKEMNSLASIKNKIIPDDENITEMEDNTPTQIINIIEKTSNIDNNKYSNSVNSVRLNTI

EEFSIENIDYETAATQPINDTEMQKSVLVINKSKNILNLSEKSNNINLDDTIERNLNAMF

EDVNEGHSEEQQQISTQLLKNMLETSHCENKSPINNLNINDNSIDCNIEKSIKNSKTIDP

SSNLESNKKLLKSNLKNIDNIPKDLSMPRSSPKKNHEESKIVNTIQEIQEENKKKECIFK

ENKADSENIGNQVLNISIDSNQLQQTSKVLENNDDDILAGLPEVKIAGTYPTSPTSSTSS

EYRININHNRTKQISIKIAPKKRRSSRKTFNHKNMQNTELDHTINKSNSTIILKNFSNSF

SSNFNDDRVSVEETNKKKKNKKTKESIEKTNPENLYKPRKESNTSSKSVEKEALVKKVAD

ESAEKNLPNTCVKKRSLNMTDTIEINNSKEHKNNIKENEFINTRNRKINKVTRRRQSANT

PDISSLKNSSIACNRKLSDEDISLDKQPIIKIEKLSVYTPTESISSTPTREVIDNKRKNK

KSNQHTNIIDDQVVSIENEKTSQNTKTQKIKFQIDEISSQIGEESQEIEMIMNNALKEQS

TETKKDVDNNATNIKTRNNKKHKNIHINADNTESSSTSSIIYESDNANFEIPTSKIKHMK

MSKNRSNITNILKNESVKEMKRKNKQSTKSTRVRRQSIIDSSTDENKKIINKSIEIKRNS

KQSIKLIQTHQQPIVDSSTDENTKIVNEHIETKKKNKKSTRSTRTHEQLIMDSSMETNQT

MLISNTDKINNLRNKRQRQTTEKIESNKKKKMDFIEETNSETNSSIEVTNSSLISTPNRT

RRSMNSSFATSSPFKIKHKVLFTGISSNDYSKLLTKLGASQVEDPTKCTVLVTDKIRRTV

KFLCALALSIPIVSTNWLHDSEKIGHFEELENYILEDPEAEAKFHFKLKKSLEKAKKYKL

LEGYTLILTPNIAPPPPELKSIIISCGGKALLRPPSSWPEKGMIISNKEDLTNAKKFLAK

APKNITIQSTEFILTGILRQELEFNEFRLYN

>Heterotrigona itama

IGVLCIDSTTFPIKKGINQIGRHPDCCIVLNNPTVSAKHAEIEANNHETASWICDLNSSN

KTKLNNLILRQNRYYELKTGDVLEFGMVRAVFKISPSLNDSLIPETPAPNHQKVQQRVIP

GTPDSSLSNSSTNVSVISATQDERKKSVFQYPTLHLRTSTSSVRKSNVQNSSVDDQDNSQ

SFGVKKQNVGESRISIYDMETQKFSCSSYNKTNVDIHDAEGQQICILNNIKNFTKFVQEQ

NVDMGKQSKIGAPEEVTDIHDIETQHDIDIETAKKISIRNSKTRDNSIRDSEKFEKTGIE

EEKNSAMEGRSNVNNIVIQKKKANDSTSHCNITNKGKNKSEKLSDIEKDLPKRLEVVEIS

EDDASEFDMSRNLLSPKDLEEFEEFIEDNNLSDEIKSTSPISIKASCVNNDSGVNSKSID

NENIYEAATQVNKIDKNDFRASLADNSDDTDNEAVFQRYLRNDSQESKKSSNLQVSDSDD

SITDEEGHFTEIAAKMKKAIETSLEIRHNRSKEMKNSNSSRDSEDLFNVLTQPSNMKNEN

SSFKCNKKSKDNEDENQVDSDSPTQVIDKKGTKNNMEVSEEIGIEEIDDIVSTQILPMNK

SSFRETSSIISTRNNKDSNEDITELKYNRPTQIINTEEKVLDVSNNEPLTSSNSSISITV

EEYNTENIDYEMACTQPIGDIEKQKSVPSVTDKNENKSKSATINFDDSVERKLKVMFENT

MEEHIEDQPQISIQALENILELSQSEDESPNVNSDTTVSRKAKKSKKGRKSKPAIVKTSP

SSVSHKESCNSPLSLRRHNISNSESVRNIINKGSEKETEDLLISNSVPTDLSTPKSTIEN

PSIPKSLCKHPSTSKSVSKDSSTPKSLGQKYLKESKIVKTYQEETNMLKHDNDKLKNVDD

QESNTSINGEQYQQISKLLMSEDDILAGLPEVRISGTLSNPPSPTSSEYRININKNRAKQ

ISIKLAPKKRRISRRTLDHRITENATHTIIEPNSMATSESSFINNLSADKISVCEKKVEK

EILVEKAAAEPAKENVIKTCFQKRSLSMTDVVDNSSSKKRKDDVDEDVSVENRNRKTISG

RKSANILEFIVRKNLSNNGKMGEEANLNKEAIVKVERISLSTPTELISSTPIKSVIEDDN

RNRNRQNRHKNVVNSQIASSTSTKENERTIQNVEMRRGRRVTKKAKPSVDDISSEVGEES

QEVEMIMNGALKEQNTNSNTKTKKDTQKNARMRSTRKRANTNVDTDTNNTETSSISSVVS

ESDNAYFEVPISRNKRAKVTKNSLTVTNTNEYIKETRERNKRSTRSTRGQQSIMDSSVKE

STETIANKTNLDNSTKNTLNVINTMVNKSIKETKRNGKQLTKSSRSQQSITDSSIIDSTE

ENTKNNRTGLGTSKTNSLKDKRQMQTTGKAKNNKQRQKDFIEETSSETNTSNETTSIFST

PSRTRRSMSSSFAMQSPLRIKHKILFTGISSNDYSKLLTKLGASQVNNPTKCTVLVTDKV

RRTVKFLCALALSVPIVSVDWLITSEKTGHFIELENYILKDPAAETKFRFKLEESLEKAK

EHKLLEGYTLVVTPNVIPPPLELKNIIISCGGKALFRPPSLWRQNSIIISHEEDLTNAKK

FLQKAPKTVTVHSTEFVLT

>Atta cephalotes

MDFSATQVYEDEDNLFTDSHALTEEESVQVGTLSINSTDYQIKTGITKIGRLDICNIVIR

NETVSKLHAEIEASSRGSTWICDLKSLNKTKLNNLILRPNRSYELKDGSVIEFGGVRATY

RIYCPANDEVIPETPAPSRQKTANIIIPNTPDSSLNNSSSVDNDGSIIFGTQKNDETRSV

FRRPRVPQQSPSPSINKKNTSKNESNDLLSAIALHTSDVSENQVSIYEAETQKFDEKYKT

TSNSIYDMETQGDLVDESKIKTNVKSSKQLPKLNVCDKTAGRSVTDIHDIETQNCLDNIN

EMETQKDNISIRNVTAKKFMTDIHTMETQADTDNIDEGSSDAQNSEIEGNSQEKNLTKLG

RSIHDLETQKFDDNYIVKNISDLETQLELNSIARGEQNKDISDMETQFETDGMANEISND

RVNKEKDRNDIANTIKDGINHEDITKPPRSSSRNRKYLNLSSSRVESRLPPSLNQSAYLL

ESSGLLELFSEGIDEQEMQASNASTPKPLAKILSKKNSNVEHVQNLINVEENDEDIFEAP

TQCVNRKFEALMSDDSETDEKDAVIMSKGSKKKPRSKQINDTLSETDTHENIAELAEKQY

KLRMLNKPSDEVVNDKNAPGTSIESEDMFDVQTQLNNPVTNNTSNWSIDQSQNSNKINEA

KISDMPTQIINNNHDNQRSTSVPDIDDVAPTQIILSREISPSNSIDLASKNNQNDVNKRI

INAESVDYRTTENLDCEDIDYELAPTQVIGEIENKRKGISTREKGSSQVNLNDTLEEKLN

EMFDDVNNDMNSIHESPHMSTQYLEKILSSQSDDSINKANDNDRDTCNVPQKQTEKKEYI

SHNQRSHNLLDNEINTNNVNKTETESQNSDIYFSTITTKRKRNILKDTQEFLDSVKDITS

FGQDADFSQTSKASNEKVMDDNVTQESNKXKKRIAKTKHKSDSIMETLNDNDKNKIISSG

NNKRSLRSSKLMKYDNEPTSKMRKQILKLESDPIKLKVDVAKEKTICTPCPSENDGQRAQ

ILDTLYESDDDILTRLPAVRISGTLLNPASPSASSTSTVHSTKSKRDIARSKRKESIPLK

GKSLRKQDNSGKHNKSSIDNQSNLVHDNLMTDRIVGLMDTSESDDSSESTYKRFQQIADR

MLSNEHNCPKRQKKRNKESSCVLSNMSENPKQSTDDESKKSWISTRITRHFSKQNDESPH

DLQKETTSYEATRSTKSKTKSIIGKKKIPLNVIEKSVEQTTSKKRKTVAEECPVSTRSRR

TAIKITTDRQSPNILEHIAKIRSRENISENIKSSEDNKIISKTTLEETQEISLNMRTDGN

VYPGRSNVKRTRQTIYDNQVITHINDVTASKNNRKNKPAPTKGTETQTNQQLSRVPKIIL

SPLKNLKSHSDNASQEVENIMARVPSNTWNKNSSIREGSKTKIFKRELRTRSKKQQNSDI

EINSVLTESSLSSEIEDCDNTQLDNAVAKAKRKRPAKSSTSSLSETTISSLSQVISIKRK

EEIFKKPSRIKNSTSSLIDISIENITGESSQSSTESDTSINSRSSRLRTARKKMESNQNT

CRLIDESASSLNTSTETTSLILTPSRTRRSISILNNSISSAMKHKVLFTGITGVIENYSE

IVKKLGGSKVEDPAQCTVLVTDKVRRTYKFLCALAKSVPIVAIDWLTESKTKKEFIDWEK

HILKDLEAETKYDFKLRESLDKAREKKMLDGYIVVLTPNVGPPPIKELKDIISSCGGKTL

LRPPTKWPERTMILSRKEDLLNAKKFLAKAPKTVTVQDIEFILTGILRQETDFVKYKLT

>Thrips palmi

MASEEFECTQVLHTSFDTSCEDLSATKVQVATLKINEAEYSIFNGDTKIGRDPKQCLVAI

DRKSVSSHHALIEVESGHHIICDLGSSNSTRLNGMLLKPNVRYNLPDGAIVQVADLRGTY

HILPLKKPEMETSVQDIFDSPCNKINTPKRFSLNDSDDIIESTPMRKVFKKPFPKIQPST

LNESVHLQDTIYGNQNDMLYEAETQIGSDLENGSRNSSSILSETLDDSVAVRDAPSFFVP

CTQDMLSESSLNLHYDTDKSVDASTSETKEGTVPTDSAKREESSEQGEVDLFLNAEEQTV

SDPSGSNNCQSEYDMDCEDLVKHVEKIERSLSSESMNASRCQETMDHLSPNKQPAADRHT

EMQNSCSAHDSSTRLTDANGSDEIKSCAEGVENPNNIHDETDEEGLVRDSSNANIHDAKT

QCLPGGVDSEPETGSVNMHASDGTLNIHDAKTQIVSANDTDSETDEEGVYGACTEMPHHP

GSSVASKGEKKDSDDIHDAKTQFVSAEHSDSETDEEGVYGASTERAPLYNTSEKNIFKKK

NAEDIHDAKTQIVPVNNSDSETDEEGVYGACTEKPQILASGDTSEDTAAEKSKMEEVHNA

ETQYVVANCSASDTDHGDIYRAGTEKPESLHNIHDAKTQLFTIESASNSQLDSESILDMN

TNEGEEADIHDAKTQMVSKISDSETDDERVYGASTERPGNVAVGSASEEDISAHKESRDI

HDAKTQMVCAKDSDSETDEEGVYGACTEKPGNLATTFAVEKGGSEGKESEDIHDAKTQMV

CAKDSDSETDEEGVYGACTEKPGNLATTFAVEKGGSESKESEDIHDAKTQMVLANDSDSG

TDDNHVYGACTEKAEHIDNIHNAQTQLLTLNRATSHIVKSKPGSIGTKNTNGVEVEEDNG

IHDAKTQMLISSSHLKIDDLGPGENFEEADSEKKDSDNIHDAKTQIVHANGSDSETDEEG

MYGACTERLTSAKASEDIHEAKTQVVSVNDSDSETDEEGVYGACTEQHLDVALTNASDAS

VAEKKGSEDIHGAKTQIISANESGSETDEEGVYGACTERIPEMTVDSVSSASKTPIMSNN

TRSKTFDSKEEANNIHIASTEMAQFTVDKTGTAGNQTSDSAFYDAETQILPELLSKSENV

HHQNVAKQSIALKASPKKRLSLSYRKKMGGANDDSKRRSVSPSKGTDQTNEDNISTAATQ

LLPNIEDDISTAATQLLPKVEDDISSAATQMLPKVEDDICSAATQKCESPRFSRRLKGIT

ANETAHSFDSPKTPSLRQIRNTDTSTPFWSAKSKLSKDDSKSSEEDMCFAATQILPDPGS

SRIGVSPSPIPTRNSPGKSLNLSHDIFNDSTRIWPSPGSSRKTRSQNSQDNVNPCDSSEE

KPSQTRSSSENVNHNSPGPSYRRSSQNLLKETDNISEPSSVATRRTPGLLPTPELAGPGP

RSSRRTSDQSPCKENSFSASGALEVSCTTPSMGDSSKSPSKESTVPVKPSPKSAEMRPAL

GSTELSKVDPISPKPSSSRRSVSFAENIQVLSNPGSTTQVAGIEKLSKSTAQTRSKRSPK

KRPSLEKQGNKKKRHLGSSVLEVDIEESSSDSNKSQDCLIDEGSPLPAQQNEDNAISDAE

EPVQARTSTSAQSPMKNVNQQQTSLFEHESTSPIVSRSKGRRTLSLSRSKFSPQGFMSPE

GLAGKMSSDDSSPKLEGVMASPTEPNDEKPNEIIGNVTSAPNSQPSKQEAAAKVARIIPS

SKQPKNEEPNEIKNVTTVTRNSQPSEDEAATKGSKPPKSLEGSTLEKKDGPIREPKSKGA

STKGKSKKTTNSQSNVSAEAHESAQPQVEETSEKSNSKIGLSGQSRLSRSTQQASQDISS

EASVVSKISIRTKNQPSKKPIKKSGTVTEAESSTTLEPSDNKLANTSKETKCNSQKVADS

KSGDSDSTSLAKDSASVNESTPAKSSRRSSLKKQDGVTKESKPKPGPAKKIKASKNKDSQ

SQDNLVSDGEGRRTTRARRQPDKDIKDVSAPQSQGGRPTRAKRQLVTVPEDHTSAAGSAS

QTSNSSAAGDRRSSRLRKQTVALKDYDCSGESQSQESSSTDQVSAASSSDHPKGRKSSRN

TKQKNDAKSGPQVKGVKRISSDVADQDTMPSKQPRTKSDLSLTLETSVQTTNVLDISAGP

SSLKRENTLNDSVDSEPESKRRRGRLDSVASINSSPRRSPRQSFRGAASTSTIRILFSFY

NNPRQESFMKQLGASIVNDVDSADVLVTDKLRRTVKLLSIVGKGLPVVSPSWLLQCKRAG

RLLDPWEHLLSDLESEAKFNMKLEESLKKAAGQPILQDQHIFVTESVKPEPEEMKAIILS

CGGHCLPRVPSVWPNNSIIISCEEDKNVWSNLKGKVSIVSAEWLLSGVLRQQLESKKFLL

KRI

>Lasius niger

MFPADASIHQANTKTSKQLQSKLNVQDEVASTSATNIYDMETQHYASSINELETQKSEDN

IRNVTSKKFTTDIHNAQTQNYIDDTDKDTDKNKNERKGLDQVECNINDLETQKLDNKDIA

EDVSNVETQPELNGIRNTTRNICDVETQLEMDGSVNDVNDDRDYTDKDITKTTKNGTNDN

EKDKDNMAETKINYNVTHSPNSRSSSPGSLNLSSPGVDEDCLSSLQHSDHLLESSDLLEY

FGEGIDKREEIRASRNTSTPKSQSKALSERDNAENVSSVSNNDEDNIFDAPTQRSIREFE

ERLDEINEKDVSLIRKLPKKKRESKESIDDDPEMDTEEHSKELTKKQHKSSEISNESCND

SVDNRNDPATSVESEDIFDALTQQSNSPAIKDTSNSPINQLSKINETDAVVDDMAPTQII

NNDENTHDQSNLTNTHKLLTGINSINSAIEDRQKKHVGRKKTEESFQAIANDSLEQKLNE

MFDNVSSNDIDEPPHISTQTLEDILESSQCDNDLSANKSTVNESSPSDNVSQTQLRKKSR

APCDQSRDPADTEANNVNKVEIDSQNYFSTPTTRQKRNILKETQELVDSMQIVISSRQDS

NTSQISKVDNEKVDDNTATEFNKREKKISNSRNESEAMADTSNDDNVTETISNGNNERNL

RRSMRRKDGTASERSKRVLRRDPGKVKIDDVEKDSGSESNVCAPCPSEDAERMQTSDTCE

SDDDILTRLPAVRISGTLSNPVSPSASSTSTVRSDRSKRGIVEDKNKEIVSSRNKSLREQ

AVEDSEKDESPNRIHDKPSVSLLDNNMPSFNALKTDKMTRNLMDTSEDSDSETNYKRFKQ

MADRMLNNEFDSLKRQSKRNVQNKKSDKKKGARSSPNLSNDLKRDTDDELKSSSQTSLRM

TRHSSRQNDKSSDVSRKEACMENATYDKSTKPEARSIVPGRKRTLSTIEKDVEQTNSRKR

KACQVEERPVLTRARRNTMRTTIDRQSPNIVDHFTSSRSNSPVIGNTKSFRSNKTVPETA

LEESQKTLNLKTDENIYSGRSYTKRKINDDQAAMCVDDVTANESSNSNKQVQAQESKTEV

NLSQDKILKIVLSPIKSSIINPIEDESQEVEMIMGKSPSNVQDKNSSIRETNTAKIFQRR

LRIRSRKQLNADTKTDSTLTESSTSSSISDSNNTQSDDSTSTTKTRRRFTRSSAAGTQAS

KKKEIFKKPTRVNQNSASSVFDSSTENTTSESSQNSMENDTLSSSRASRSKVAGLRKTEK

IEMNQSMRRMINDSASRINTSIEIASPPSTPSTRMRRSASVLSNSTLSAARHKILFTGIT

EDYSKIVKTLGGIKVEDPAKCSVLVTDKVRRTYKFLCALAKGVPIVAIDWLRDSESAARF

LDWEGYILKDPAAEAKFGFRLRKSLDRAKEQKLLDGYTVVLTSNVAPPPIEELKGKLKHY

IFMPLYKTYLADLR

>Zootermopsis nevadensis

MAESVIPDTPKPEMREAEEPHGSTLRAEKAGDGERGMEGLRKPGIPTSGVQKENNEVSGMTESKKTNDSV

LGTHGNGRGTSDMNEIKKPGDNMASDQGCFNGKHLISNSKKSDIKSTQKEDDSKCEGAGSEIPSDDKSSI

HDGSGDKTDSENVLEANSQKIQVDRSSELNKVKKSAVPIDAHKNVDVVDKLIDDNLNDTFELIMPSSQDL

MKAVKAYEAKQSPFKPEEVIPSEDEEPSSLTLKIPRHSHLKSKRAKVRKRPCTKSKASDATVVGSVKHLP

DESSVDVSTISNAAAGTSRRRKLSYEDIDQSDKSDIILQASKQRRLSNKDPLENDSKNEVNDMTSRRRSR

RCPGEIQQIETEDVVNTDKENGKARTSKIVKGQDEIVASADSQTETNVKLSIETSRREKSLRSGVHRNKK

EEQGFKSEQSNKPRKGRRRLKEVVESTEDNDNKETGKRSVDKKEDVENALATSIRPSIQQKMTRKIRDSL

GSDKSEGSPAPEEERSKSRSRGRLSSNSQNEVNERVELSTHRTLRSRKVSLSKPPSEELLKNSEDFGGNT

SKDVKEKKITDSVPGRQNVSLRKSPGQSKGDGEVTSVTDSSKNRSPQKVARRNDIEIVSNRERKRKTEET

VTEAASKRSKKAKSQENSADRCSPETSKNVLNTDESFKDLDSDFRTRISGRKERQTANVEAMQEPSTNIH

SVLKSSSSSSIESVQSCTGRSVRRRMAVAVKDTNLSFLNVSGRPQRGVKHAKSADEERAEHLPAKQTKTE

EKLSKTHSPSFENSVQNDEVKCVQSPRRGRTRRKVMSSQKSEITETAKHDTKSGQRERTSRISKTDKTYK

GLENEPTTPKSRKINLSADQDITPQHARSSRGRGTPQSNIRLSSQKSDSNVSTQRRSTPRRMASAGGTAH

HVLFTGFSDSKQEYVVRQLGGRVVDLPESCSVLVTDRVRRTYKFLCIMGRGKPIVSPEWLAQCQRSGCFV

DPWKFLIKDHESESKFKFQLRESLEVAARTNLLAGYSVYVTPKVNPPPSEMKGIIESCGGVFMSLGAVKS

WPVNSFIISCSDDKASWSKLKKSGKPIVGADVLLLGVLHQKLDLKSNTLV

>Bombus pyrosoma

MEDSLVPETPVANLQKTQQTIIPGTPDSSFDNSSTVGNVSVIPATQGKEKKSVFRYPTLPPKTASNSTQK

NSIQNTSVDAQDNSQNFDVEEKQNVGGSRISIYDMETQNSSFNFHNETDVDIDDIDTQKICISRSIKNLT

KSVQEQKVDIHDIQTQAEALEKVTDIHDIETQHDINIQDMKISKKINTHNLETQDNRTRDSEKLEATGIE

KEGNLTNDVRSNVNDTVVQIDKGNESTSNPNNTDKDENQNKKLPGIEKSLQNRLEEIETSEDETSELEMS

RNLLGPKELEDFIEDDDLSDEVKSKSPTLIKTSCVNNNSDVNNKSTDNENIFKAVTQVNKSDEDAFKSLS

ERKYTFQASLVADDSDHTNNEVVIQRYSRKDSQENKKFSNNRVSDSEDSITDEEGRFTEIAVKMKQAVEF

SQLNRSKEIKDSANSSKDSDDLFDMLTQPVNRKDEDSSPKSNEKPKDNKYESGVDSINPTQVINKKEVKN

NMEINDEIQTKELDDITPTQVLSTNKSSLREVSSTISTGNNKKSDKEDITEIEDNTPNQIVNTKKKPLDV

SNNEPPSSLDPSILNVTDRCSIENIDYEMACTQPINDIEKQKSVSSISDKRKNESNFEALARANLDDSVE

RNLKAMFADVKEERIEEQLEISTQVLEHVLESSDCENISPMQNHKSNMDSNSSTDKKAMKLSISNSIPTS

SSTPKSIPKNPLTSKSVSTDSSTLKSAIKDPSTSISLKEKKSKKSNIAKMIQEETDISKNGKSKLNKYSK

NIDEQASSISIDCEQFQQTSKALQSDDEDILAGLPEVNISGTSSNPGSPISSTSSEYRININRNWEKQTS

VKIAPRKKEALRKSSRRTSSCRNVENAVSYHTISEPNSSTISETSSNSYTNNFNINKIPVCKHDEKRTSR

KSKRLIKKTKNLWKSKESDSFAELKSVTSIHSQEKAKQSPPISNNRRTRNSSKENTDRSSVFQVEKEVPV

EIAPAELTKKNVSSASRFRKRSLSITDAIDNNISKKRKNDVNEDKPVSNRSRKKNTIDDEGNSANVDKKN

SSSSINGLNNRRMSNEASLDKQAIVKVQRISLSTPTGSISSTPTELVDESDNNCKIRQNRRTNVICDIQT

SDSRIVSSISSNENYRTTKNVETRKNPNRKTTKKTELVADDISSQIGEESQEIEMIMNSALKKQNTGSNS

ERKEDTDRNISTKTKNTRKRGNADINTDVDNTEISSTSSILSESDNAYFEVPTLRSKRAKVSKNISSIIS

TTVNESTKETKRNSKLSIRSTRSQQSIVDSSLEESAEMIANQTTSNNSANKRNNFRNKQQKQTTGKTKNK

RQKEDFVEETPTSETNSSIETTSVLSTPNRTRRSMSSSFTTQSPFKIKHKILFTGISSNDYNKLLTKLGA

SQVEDPTKCSVLVTDKVRRTVKFLCALALPVPIVSVDWLINSEKAGHFIELENYILKDLAAEAKFRFKLG

KSLEKAKEHKLLKGYTLVLTPNTAPPPLELKNIIISCGGKALLRPPPRLWPQQSVIISPKEDLTDAKKFL

AKAPKTVTVQSTEFILTGILRQELEFNEFKLI

>Apis mellifera

MDIIATQVYENCDVTSSEISYSTQDNKIQIGELIVGTETYPIKKGINQIGRHPDCNIIFNNPTVSKRHAE

IEANNHETAVWICDLNSSNKTKLNNSILRPNRFYELKNGNVLEFGMVRAVFKFYHSMDESLIPETPALSR

QKLQKLIIPGTPDSSLNNSSTLGENISMISSIQTNEKETIFRCPNPIPARTSTSSTRKNNLQNSSVNNLD

NSQNSDGEKKQNVGVSRINIYDMEKSFESLNGTNIDVHDVETQKIRLNNKEIVLKSLNEQKVNTHDIETQ

HEINAQENVIDIHDIETQHDIDIHDMKTPKKIHAISRNDNKTQDNEKLKTEIEENGNLIMEKENNIIIQK

DEAIASTSYFNDTNKCKNKIEHGQSIKKHLQKNSQRCSQSSIATNFENELSELDTSRNLLGSQCLLEDLI

DDDDNDDLLDESKSRSSTPLKTSYVNDDEDVNSKSTDSENIFEAATQINQTDKDTIHRINYSFKASLTDP

NDSDETDNECVFQRYSFEENKKNEKSSIIRVSESDDSFTDEEGRFIEMAAIEKRAASNSFEKRNRNKEIK

NDSSKDSEDLFNMLTQPINKKDLLSKCDEKTQNIRDKVDKEIDFDTPTQVIDKKNNSEINKDIEIKERIK

NIEVEQVDDFTPTQVVSTLKSSSKEMNSLAIKNKIIPDDENITEMEDNTPTQIINIIEKTSNIDNNKYSN

SLNSIKLNTIEDASIENIDYEMAATQPINDTEMRKSVSVINKSKNILNLSEKSNNINLDDTIEKNLNAMF

EDVNEEHNEEQQQISTQLLKNMLETSHCENKSPTNNLNINDNSIDCNIEKSIKEKNSKTIDPSSNLESSK

KLNKSNLKNIDNIPEDLSISKSSTKKEHEESKTVNTIQEENKKKECIFKENKADSENIGNQVLNMSIDSN

QLQQTSKVLENNDDDILAGLPEVKIAGTYPTSPTSSTSSEYRININHNRTKQISIKIAPKKRRSSRKTFN

HKNTQNTELDHTINNSNSTTILKNFSNSFSNNFNDDRISVEETNKKKKNKKTKESIRKTNPENLHKSKES

NTSPKSVAFINSQEKARQSPFISTNRRTRNSSKENIDHLSIFQVEKEALIKKVAGESAEKNLPNTCVKKR

SLSMTDRIEINNSKKHKSNIKENEFINTRSRKISKVTGRRQSANTLNISSLKNSSISCNEKLPDEDISLD

KQPIIKIEKMSVYTPTESISSTPTGEIINKKRKNKQRNQRTNIIDDQVVSIENEKTSQNTKTQRIEFQID

EISSQIGEESQEIEMIMNNALKEQSTETKKDIDNNATNIKTRNSKKHKNIHMNADNAESSNTSSIIYESD

NANFEIPTSKIKHMKMSKNKSNITNTLKNESIKEMKKKSKQSTRSTRAHRRSITDSSTDENTKIVNEHIE

TKKKSKQSTRSTRTHEQSIMDSSMETNQTTLTNNINKINSLRNKRQRQTTEKIESNKKKKMDFTEETNSE

TNSSIEVTSLVSTPNRTRRSMNSSFATSSPFKIKHKVLFTGISSNDYNKLLTKLGASQVEDPTKCTVLVT

DKIRRTVKFLCALALSIPIVSTNWLHDSEKIGHFEELESYILEDPEAEAKFHFKLKKSLEKAKEYKLLEG

YTLILTPNIAPPPPELKSIIISCGGKALLRPPSSWPEKAMIISNKEDLTNAKKFLAKAPKNITIQSTEFI

LTGILRQELEFNEFRLYN

>Frieseomelitta varia

MISVIHLTIAILLLNRYNTVSKKHAEIEANSCETASWICDLNSSNKTKLNNSILRPNRCYELKTGDVLEF

GLVRAIFKVSPPLDDSLIPDTPALNHQKTQQRVIPGTPDSSLNNSSANVSLIPATQDERKKSVFRYPTLP

LRTSTTSIRKSVQNSSIDDQDNSQSFGVEKQNVGESRISIYDMETQTFSCNSYNETNIDSILNSKNLTKS

VQEQNDMGKQSKIGAPEEVTDIHDIETQHDIDFHDIETAKKINIHNFKTRDNSIRDSEKLEETGIEEEKN

STMEGRSNVNNTIIQEKKDNDSTSHCNITNKDKNKSEKLSDIENDLQKRVEIVETSEDDASDFDMSRNLL

SPKDLEEFEEYIEDDSLSDELKSRSPTSIKVSCVNDDSDVNSKSIDNENIYEAATQVNKIDKNDFRVPLV

DDSDDTDNEVVFQRYSRNDSQESKKSSKSQASNSDDSVTDEEGHFTEIAAKMKKAIETSLEIRYNRSKEM

KNSNSSRDSEDLFNMLTQPSKKNEDLSSKCNKKLKDNEDENEVDSDSPTQVIDKKSTNMEISKNIGIEEI

DDMVSTQILPMNKSSFRETSSIISIRNNKNSSEEDITEMEYNRPTQIINTEEKALYASNNNRLISSNSSV

LITMEEYNTENIDYETACTQPIGDIEKQTSVPSVTDKNENKSKSTTMNFDDSVERKLKVMFENTMEEHIE

DQPQISIQALENILELSQSGDESPNVNSNSTASRKAKKSKKGRKSKTAIVKTSPSSVSHKKSCNSPLSLR

QHNIANSESVRNIINEESEKETEDLLISNSMPTDLSTPKSTIENPLTPKSVCKYPSTSKSPKDPSTPKSL

RQKHLKESKIVKTYQEKTNTVKNDNDKLKNCVENVDDQELGTSNGEQLQQISKILISEDEDILAGLPEVR

ISGTLSNPPSPTMSEYRININKNRAKQISIKIAPKKRGISRKSSRKTLDYRIIENATHTINEPNSMATSE

NSSNSFINNLNADKISVCENYEKKVSEKSKGSMKKTKNLCSSKRSDSLPKLKSINSQEKTKQSLSIPSNR

RTRNSVKENADFSSVFQVEKEVLVEKVAAEPAIENVVRKSRFRKRSLSMTDVDNSSPKKCKNINDDVSVE

NKNKKIINGRQSENILDFIVRKNLSNNGKMSEEANLNKEAIVKVERISLSTPTGSILNTSTESVIENDNR

NRNRQNRYKNVVNSQIASSTSTKENEKTTQNVEMRRNTSRRATKKNKSPADDISSEVGEESQEVEMIMNG

ALKEQNANSNTETKKDTQKNARNTRMRSTRKRGNTNVYIDTDNTKTSSISSVLSESDNAYLEVPISRNKR

AKVTKNSLTVTNTTVNEYIKETTGRNKRSTRSTRGQQSVTDSSVESIADQINLNNNTKTTLGVINTMVNK

STKETKKNDKQSTSSSRSQQSIMDSSVKESTETITNKTNLGNSTKNTLNVINTMINKSTKETKRNNKQST

KLSRNQQSVIDSSIIDSIEENTKNNRTDLGISKTNSLRNKRQTHTTGKAKNNKQWKKDFIEETSSSETNT

SNEITSILSTPNRTRRSMSSSFAMQSPLRIKHKILFTGISSNDYNKLLTKLGASQVDNPTKCTVLVTDKV

RRTVKFLCALALSVPIVSVDWLIASEKTGHFIELENYILKDSAAETKFRFKLEESLEKAKEHKLLEGYTL

VVTPNVTPPPPELKNIIISCGGKALLRPPSSWPRNSIIISHEEDLTNAKKFLEKAPKTVTVHSTEFVLTG

ILRQELEFTEFKLI

>Nezara viridula

MEYTQVLVSESFDIKNTATEKIPVATLLIEDEYHTIYNTENVIGREKTCDIVISNNAVSSKHAVIEAEDS

DAHLIYDLGSTNKTKLGKMVLKPHVRYHINDGDSVTFGNVSAQYNISRKYLTTPVKGNTTLNSCSIPETP

ERLVSTLELDATESTKQKVESSALELSDTDLFDEKEADTNLYLKEIPSSPSKPSNDIYELDTLSFDEHIE

NHVKKTDHLEDLNKINVIPANNDVEEKGFDIDPQELGHDEKPVSKSPPMHDKDKTKTNFGWEDFPEITSH

VELDSSSANNLVHSKSKVSSSFLDITNDDLKPDQHIPKNIGGVILSDLCKPACIDSDKQDVSSTSKDGEL

KIVDETEKGLLSEKTYIEPKTPGIGGTKDSLYLENDCYIPMSQNIDMFEESPEKTDERPEQGDVYEMLTQ

VSIENKEVNNSIYDIQTQMVIADNDSESKVDKENESISITSPNTKLSMLLESASQPAENNDKEEMNNTSY

LKTKVIDETDNNCSFSESIYEAETQPFLQKVSKEPISEISNCLPERMDKNGSVTSAIVVTTQPCIHGSLE

IQDSTSSEINNKADEKENLSTTIIGRTTQLCPVEISIDPSKQSHSPKAVVNVDDTEIKISSSSSSVVGAG

TKPTSLVNQQLLHEDIESNSSEKISAKDLSICDWYEIATQPCQPTSENVLIEKEIQKNELTKNDDLTDDV

FKKEAMQDDKFSTSMYEIATQPCPKDILSENNESINETTLNPELTSMRDDNNTNNNFSNSICEIATQPCP

KDIFPEKSETTLSPEFTMGDESNKNNLFSSSICEIATQPCPKEIFPKKRESIDNETTPGSELKTMSEESN

KNNIFSTSLCEIGTQPCSKDIFPEKAGSNNDTILNSELTIITDENNKNSSFSNSICEIATQPYLKDIYPE

KAESIDAKVSDESNKNDIFSNSICEIATQPCPKDIFPEKVESINDTTLNSELTIITDENNKNKIFSDSIC

EIATQPCPKDIFPEKSEKINETTLNSEITTMGDESNKNNFFSTSICEIATQPCPKDIFPEKVESFNVTTL

NPELTTITDEINKNNIFSDSICEIATQPCPKDIFPEKNNETTLNSEITTMGDESNKNNFFSTSICEIATQ

PCPKDIFPGKAESDNEKTLSPQSKTVNDENIKNSSFSISICEIATQPCSLDSKGITNQYESIEKGACDKM

NVKETVALQVGNEIEMAIDPKIVNSKNLLPPEIIHELDTLPLLHAETIPNIFNKSIMVDKKSANDYSEGE

SVNLLLVDKTKQPVITELIVNQKEIMPILDISANQFQPAPCQVDSNLPSNEENLSLITKVLATSCEVEKV

LVEASESDINIYKAKNILVTTKANDFSLTATLPNALSDSFFEAATQPYKNDQGQDRTFPDPLTSICNDLS

DDKIGEITDELMYGNISTKLEDNNKILDNNNPTKERNKPRKNVETSDDNIFEALTGQDSFCSQKKILLNK

INTENPPEKISTQDLQRILESDDIIQKYDEFKKTDATSTASDPVLNRGNNVPQDDGNEDLLKLGTKELCN

EPVPDLHILGSNVVCIDSDSSQELNIRSNKKRSNVFPPDSQSFSNKFLDNEDSSQELNIKSNKKRCNIFP

PDSQSFTYKFLDNEDSSQELNIRSNKKRSNVFPPDSQSFTDKFLDNEDSSQELNIKSNKNRCNVFPPDSQ

SFTNKFLDNEDSSQEINTISNKKRCNVFPLDSQDSSYKNLESASNVSEEECIKIVGKDSVLESTKSSGFL

NKINNDIQKSNKDDSSEIVKNTSVENDDEADIKMGLKKLDTTTSNQNKVLKEKLNERISTSGKRNNKENK

NKSPPERERESTYISRGNITLHSPEVLSELEEQFRLTKVAMSPTQLSVVSDIESEINSSQEVDELDKQGI

RRSIRNRKNTVSVKSLKNTKSPILHKMIEPPIVTSNENLLKENESKRKEANSSSTTSKGEKSFKRFKKVS

PVKNNTPTNEKLVKVTTNEGYIEIANNYSDNSQNSEELGRGKRKRTRPSYEGFIIEEEKKRTSRKKLSFS

NSDDSENMSNVAANIEEINNSVPDESVKKQNDVPSDSIKKHVSVVLDIQKVPKEVPELNVEKSRVSKKKP

SKKNETSPVLVDKISNVPNSSMKDEAKAKQNETSSKDKNCSFSFSEKLDELFNIWGPADRVKLSPRGAKK

SYKLLAEGKTESVSPTTSGCKSQTSNSLAKEPKPSTSRSNKNSIKADEKKNEPICIVETKTVITSRKRNV

SQKSEVVELSDAALEQSKKRRKTNSENSETSQEPDTNDSEVRKSSRIMRKKTPDILLADCKHEKNPGQTN

RTSNELQKRVGNRSTESTEDSTSVSNKNIKTSLRNSRNISMKPPLSTVIVNVKNKNSKTKEKKTPIHIKE

NSSPEIIEENYCFSQEYSFHENKIKKGRKKPSENCSDLPLKIRRSSRKKNEKTAERNVENVMFTGFADKK

TRSLVNVLGGQIVESPFTCTVLVTDKVRRTTKFLSALSRGLPIVSPQWLIDSNENGRFLDSSDFLLSDNE

AEKMFSFNLKNSIEKAQNGKVLDGWAIFVTPSVKPSPEEMKSIIECSGGRFLSKELTRWPINSIIVSCDE

DRTMWPKFSKKGKAPVIVNNEAILLGILRQELDIESHKLL

>Penaeus chinensis

MDDTQALECTQALPADWDEDDDNNSGEKRIVAWLEIEGVRHDIFEGETKIGRDQATCGIVLQNKVLSKEH

ALFDIEGETHTLADLGSMNKTRIGKMVLKPKVRYALHGDELIRFGDIRAKYIINEKIVLDDSGSETGSES

MLLIDNTQEAPNSPVLSHQLPGLDITNNNSSCSTPKVSQIVPQDISDFIPETPTVEQPRGSSSMKTFIPE

SPSASQSTPLPHKSKAFSVAESPCNISVIQNMSAGVGDEDSFFFDPSQPVKHKIDKRSEKDEIATSDDVR

SRVNDDNSIRDEDDDSPTQIFDSPKDVKEKNDTGKELESKTPEMNSEGDRNLHANDNSGANIDNKEDIFN

QPTQAFTHIDKSPNKILEKKKVTISSFVDSKDNDDENTDSEEDIFNQPTQAFSNLNPSPKKLLKQKVSEL

SSSVQGDPDNTQDIMEAFEMVPVKSNSENSAQNEDSDDESLIATQPFVVKGSPAKAKHQDDDDYVSTQLF

LDDDDEIVFKKPFTVAPKFRKSNPSVAISNDDIQDELFNDDNQDVLDTPTQAFVADDKDDLEAPTQAFAG

DDKDALEDPTQTLVFDDKVASEVPTQAFYNDPVPSEAPTQAYDDDLPPTQKFDPKQEAVSKEKEPEAKLE

GDDDYDSDVDDYFNQPTQPFLAENTDRSLLERPTQCFRETSPKNITKDDNDEIDDFFNEPTQAFAEAKHS

ADIDFDAPTQLFGKRSEDVGARSEDESLPPTHSADIDSDAPTQLFGKKSEEVGALSEDESLPPTQIFTAP

SPNVLTPKRDEQVDDSLMPTQPLPKDSTNTPKTSENTSAHSEPYDIDAPTQIFQEPLVAQDTNEPPQVFH

DEDLAKSAENIAPTQVFESDANATDGDCRASTQLFIPEDKTSLVNRPQRGISEIWDKLDADATQDISLNV

DVNIDETRLANESNLQVLSERKELVNKEDYKGSDMNYDDDNSSTVSENLLEQTPQPHYQDCQLMEVGQET

KDENYYSDESTDMEDEILAPKDTVKVQEVKNNASILQTSNMSRLELSSDSDIIPEVKDTVTQPPKNETSH

QELEGQKDKPLDGEKKQIYGDNGSQQCKAQKEGASGKPGIHFTRVSKVVASDPVVLKSVGKAANYSQSTQ

DSTCVEDIKIYSSDSDDSDSMDSKNCKSVSEKVKEVAEILSDSETDDDVGNVSQRLFEFSQENEVDKGIG

KQTSNKDKFEEEYHHNTKGRDKTEKQDKQIHALQCRKAEKRTVENMTKDNKQNIQKEDKKETLIKSKINT

RKSLMKQIQSPIDSHSKNYMRNMINKKEIFEKSDKCREVGAEETKCNRTTRGRRRDKRQSLDLDSNTEQK

TKSGRISREPRRFYPDIDSEVADTSSASVVSKGKTVNISKNMEEHVGVLLSKRTQSITPSPLKKDYKNEE

ETTISSSAKLGNSPETDPNRVDSLKKGLQRSVPQTKEVQCETVGEQVKKDPGEREQRSTSKTVKEQKIEE

QIQQSSLETVGHEKKMEVQRKTNRASITLKKNLVTVSEEQSLEPVRRGRRSAINNPKEGKTEKASESNRE

TRVTVADIVHKEENVHTQSRQCRSVYGRPKENRNISTNQITKPETEEKKIIRKQEDSVEPCRRGRKSAIT

KSEEVMIEEADENVKQKSIGTSVKNNPEELTKRANQKMLQKSVEDSIEEPFKQKVKIQPSRRGRRSAVPK

SKDGNTENISVTDNKGIEIENEPSRQGRRSAIQKSVESGILNQVRETEDNDTGNGSKEKTAGRQKRARRW

VVSETKEMDESTDSEKTSSLKLTIKSMQKESETTSRPQRRGRASIATSHPTVKEVPRSTRQVRLSMIPER

LDDENGGPSKSKKLKKSQGKELGGCSFVEEKLSEMKVIDKGKQKQKNSAEESSDSESSSKRSSSQESQRS

QGRKRARRNSNSSEDLHSTPKSARTRTKVDSPYSKGILWSPSQRQQQADIRPKVLFTGYKDQQDEKIVTD

LGGMVVESPKECSVLVTMNIRRTCKLLAVIGKGMPIVTPQWLSASKLARNFVDPWKYIVKDTESEKKFAF

QLHQSLQSARKSLLFEGLSFHATRSVKPPPDQMKEIIVCSGGIYMDVLPKRYSPEIRIISCAEDKNQWGA

FKKLQIPVLGTEFILTGLLRHQLLLDDFILA

>Homarus americanus

MSRKHPSRLTGGREEVTAADRDVLTLPSACVVIKYFILFQILDRYSITKMDDTQELECTQVLSDNWDDEG

SVSGEPCLAGWLEVYGIRHDIYEGETKIGRDAASCKIVLQHKVLSNQHAVIDVEEGLHTIADLGSRNKTR

LGKMILKPNVRYALQGGETITFGNVQTKYIVKPKEVQNNDSGSETGSESMFLLDGSEDRPTSPVSTHRLS

DIIPATPATTRAHTTTTGSDGNLFVSESPSTSQSTPFPDKSTRMTVSESPNNLSAIRNLDEDKDGSSFFF

EPSQPPKAKPTNTPEYSNAKPGRVSFEKSEIEESISDASTDIEDDEVPPQLFGENECQNVSNTSHTTSRD

KDNTKEGSEGDLPDSKVMGALNDKDSETTGDSEETKNNNSNTNIEEDIFNQPTQAFSIVEKSPKLAGNIS

DRSNLSPIQKYIDSTQEIMEAFELTDEKNIDDDDDDTDDETLLPTQPFIASQSPTCNKFQNVDDDVPTQL

FLDSEDFVFKKPFATVPKLKNKIADSSATGKDDDIPDELLSETTNGDILDTPTQPYLYDDQHETAKCNDS

ILDAPTLPFDDTSRILQNKKSDEDDDDDDDDIESIFNQPTQPYSAIDNDTSCNEDAPTQYFDPVPENEIS

REENKSIKSEDESDIFNQPTQVYSAPNDDTDVDPNALTQPYADEPENKGVKESACAKRRSITSKQILSPV

PTGEHVMADDINDDLPPTQIFTVHSPVTEAPLPAAADDNDITPTQLYVLETDKNKLFPTPTGAHDMADNI

NDDDDDDLPPTQIFTVDSPVAEAPLPAVMDDDMVQMKPTQVFTDDSNKKVDGPPHSTPGDTRQHSKEPLR

GISQTWGKLDAEVTQDISLDSLSGIKETGSDPRESKLMLEEKDKFKDTNLNDTFSDASETLLNDKQIEDE

DDEYLSDLKYKNDNNASSSSKGNLLFKSESEKYDLDQAGKKIENIMKDDESDNIVCGMKEHINKNSNEVP

VVEQMKEDYNSDESTDIEDEISTRHKIVKLISNASSTPMKESVKSCPETAYNYKMTFEFKRGSRMAPVAV

ASTAARHSTQDSDTSVDLTYLKFSLPDSEENESQENFFETSQTPGKERKVTGPHDPQALSSSDSEDVGWN

ISQRLFELSPECDGVNNEKVTDGENTNKIEDNASEKAEVFRKSSDIGQKHQDQEGNTTLLKRIEKGNPKE

LKKLLDAGEINNEPNNSIRNETVCGSGLGNYFSPIVTPKRVKGQRPTRRIRSSLSFDNVSDDKFDAKRKT

KRKTLYNDESGSDISDKFSGFDESSCERQKECTTDKVNEEKPKSESRTVQEVITKARMKDDEISSNACEG

KSSEAGVTTASDDYVEDQQQMKDKIKETEPNKSDKQSLTGRQTRRKEKENKEGLLPVRVQRKSSRVMKIE

VEEKNSPKHKTRIQTKESDCEPSSKQFSASRGSVAGVTIDDVQSSRRSGRKRNNSDASNLSHLSSISNQS

TSRRSIPHTRSSKDHVKGGRSQAHNIDECKPVVSSVELKEDESKVRESKMEVLGNSPSVTEDNREPEKKP

DSALGENSAKSGDKTKSIKKQTRRSSRNPEPLVIKSGKNKASNKEHTKEDVLNENTCSSKIIIQEDSNAE

LKKPDNLTEGLARTSRVRKVPKRFSPGKDVAHETSQATSTRSRGRLRKLDISEVLLESSTEILPAKTRRG

SAIPKLHVSTATENCELEILPARQRSNTKLSLATDDTEKTKKSAPVSKKKCKSTSKNPQNLEEKSEATQH

ESTNHQSKKKQTQTARKARASIATPLLTFKEDPVIPKKLRHSIIVQQKINEDSKNIAEVKNIQTKDKKTK

MSAKEKVTLEDKSKPAAADQLHISKKEILKQGQLKRGNTNDNSEEETGSSQSSDDSHVSLRSKRMRGSGY

SKAPSSSSSRSSRSGRSRMGSPSQKDILLWSPSQRQQQASTKPRVLFTGYKDTLDEKIVTDLGGMVVESP

RECSVLVTTNVRRTFKLLAVIGRGLPIVTPTWISTSKLAGNFVDPWKYLVKDTESENKFGFQLSQSLKSA

KTVLLFEGLSIHATDSVKPAPDQMKEIVECSGGEYLDMPPKKYAPQLRIVSCLEDKHQWTSFKKMGIPIL

GTEFILTGLLRHELLLDDFYLE

>Eurytemora affinis

MEETQMLCTQMLVESDPEGEEEVEERQIGWLEVEGIKYRINQGENKIGRDPSCSIVLQKSSLSRTHAIIE

GDVDGSTIHDVGSSNGTKKGNIKLKRNVRYNLCDAEEIMFGDVSSVWRIYSQDQTFQVDETGSVGSQGSV

SLLDLQEDQNIQEAPDPGEENGDKKTDNQENLPPNFVPDTPAQSKFKGRSFDDFSFIPESQSSPLPSALK

PANPKILDSPFSDLNDSSFLAPSQPVQGRMQKLLNQPSRNKTSTQRLQVSEIHRVEDSLDEDDLFSGSNC

STRLDASIEQSNLRENQDKSGLNQTEVTTFTDKSRLSDSELLTAPTQGVNKLSDSELMLVPTQAVNKLSE

SELLIASTQAVGKLSDSELMFFPIQAVSKLSESEILAVDTQTVDTAVLGADTQAVLAADSQAVDVLAADN

QTNDGLAVDTEADDVNAAASPKAGNVGSSMINAENVTDDSTSQVIKGTVDDSVFEVEDNFDDSDLLLAAT

QEPFTTERTRVVDKTEAPTVILAQPGDKDDLENGSYNPSLCNLSYVSLVPGTQEETEDYQNPVEEEEDEN

EMSQNLLEYLSDGEYDSLVNDKEDENNAEEISGAEAESTGITEFDEAEHGDDTEHAASTEHEAATEHEAA

TENEAATENEAATENEAKSNNEAATEHEAATENEATINNEATTEQEAADKQETTAEHDTEETNKTETKAD

TKAEAEHDFINLEPGKITHSDSLIKSHEVEIRAVVNTSGIKTSIAEESNVKTAPVLNKQDSDMIVATSQE

ASEPSSRRIFNGTNYAELSMEQTVMLNKTHPSCSHASLPGENKITEISEITKTDNSEVNIPKLDKQDSNM

IISSSQDSLPHRVAGVRGILTETNYATLSMEQTTMVATDIVFANSQGSLGSILKTPRSKNPKIKSANPSP

VQEETSSPIICKSQSVENRSLRLCLESSDLDSPIISKSSRSGSVASKLYESMGIGSDDEDVDGSVLEPEK

DKDVLPLPQELASESDNSVPLKSDSLIVAKTPVQPKNAQIVETPQRVNNSPSCQEKPDSGPLLPETQEIV

GRLLHGIKSIDKPEPSKDEVKPSKAENEEELGRGRRSRRSTKKSLENGPEPKASNRNSLTTEKGSRGRKG

QLQTQTEEVLGPGETKHDSLSKPADKKKASRWSRAVLDQETDPNPGQMEEDKKLKENIPETSISKLAPDK

KTSRRMLAVLESSPNPAPRNQKRKQRPSIVINSSEREGLILQEESLKQKPASKTDLESTKSVLNIDEDSR

DSEILFPGEQSNVDISDDLTQVKPSPEPSPDPSPEPFSEPSPEPSPEPSPELEPSSASKPSKEQKKLDKK

DSDISDNSEICNKPVKEGKAKRGRKSAERKSKIEKVNEILKTEATKEEDHNEISKPGKRKSSRRETSIKS

LSKQDKPETNTNPAEHVERSESIETASTVSHDEPEILNQRRSIKSWKVKTENAKEEDAGLEPIKVGRRGR

INVKSNLTPETSNKGKGLRGRSKNESETETKAIDQTKEENDISISIPEPNVRKTRGKRGKSNEEKVEIAE

DENMNPDKASNLESSVERKSKRGGKSRINVQVDKEKEEEEEDQPIVSASSGKRRKINDGNEDEIKLLDTG

SIRRKGVSKKGNNSILTKLESKSHEDLKEESPVQKPGTENADGKRNASSRKGRGNIIGEEKTELIEPAVE

LGSKRLGRKRKTSLDTTKSTVDTSTRSSRGVSHPSPAPSNDTSTTSKTDKKRSKSNPVVQEISENREEIK

SQTPQFNDHTSSERKSVKRKMESNSSEIVRRPKLSKGLSPSEQVDHDASLCGSPALRRATLVKKCKVLFT

GYFCEKENQIVLDLGGEVISENISDCTVLVTDKIRRTAKFLCMVARGVPIVSPAWISQSKASGTFLDPWG

FILKDDENEKKWGFKLESTLQQAARQGLLKGVSIHVTRKVNPPPVQFKDIITYSGGVYLPSPPTTKDQGV

YVLSCPEDKQSLVKIKKSRVPVMDKEFILSGILRYKLDLSLLLQY

>Xenopus tropicalis

MDDTQRLQWDDEEQDVSFDRDKPVGNLHMFNGIHGPAQDFPIYPGTNIIGRHTNCDVTLP

AQSVSKKHAIIEVEADCHTLCDQGSLNKTRRGKAAIAPHVRYALSDGDLLLFADVACRYS

FTKKLEEEEAENEESEDDILVPGTQATLSIEKTPGVAIRRMGRGAVLAMDSGDEEEEELK

GKDLNYEAKGEEDNYIQEGLKTSGEECKSPASASFSHNMDTIVPESDEENDTSVSEPRFP

SLNLRCDSDTDTHDTPMKAGTSYTPSQDCVLSPSFKEQVVGDHSEITKSTLISNQEMFAD

EQVNTESKVSKCSPDTADKNDRETKEENNTPIVNTNIAEEGGTGVTDDPLIDGLKVENQK

KENDCYETLQVDIKQQESVKIILDSNQEENRTLKVKLVNTGSADIILDKNIDGEEGCVNT

KEIDNQIREGAFHLDSDTDVEESDTSIPGINVPRVENSGNNTDSDTDTEEDKPKILQPAI

QQNVEEGFNLDSDTDVEGNDSNSVNKRLKNTEKAKMIQDSDTDADDSIMSKVEERRNPAG

TKKDYSSTSLNTEGKRQENYLSTLDSDTDVEDIADGSSPSVVKKQDKAEAAHTNVTDTTA

GEETCTDAGIEKIVVSADRNECHTGGVTQEQEATSEFHLDSDTDIEEDDNLLDMSSASTR

VKVTEVHSVILEKLRTSPGERKEKRAIEIDLDSNTDVDEADETEVDRKETFHMDSDTDLE

ENDSGPPVVGGIEKKFTAIAHSDCTSDTGSNVKNVTSSITENTEKGETDGIDCEDAEYQM

MATQCYLEPQEKETDIQDEDDCAEEATQAFIFSSTWAEPEPFKRPADPIGVLQISAVTVS

SSEEEPDENAIAETQPFCCEIKQPEEHSDQEPAKKSSIAEEKNNVACSLPQEEISQDDTQ

PVSQYLNKTASSNLVAPNLTCTTNFQQEKYLDGINEEKVKQANESVPNREEQLDEGVQDI

EEDATQPYVLSLPTVGDMATQPCSMSESVSGDGASQYLNTVVPAVASVASLPCNPSMQLS

DGEPMQPSSLNEADATQCHGFSMPLKEGDTVSISEEKVSVKQGPCRGESSMISEQLDPSN

NVMSKHPGEVLVHSVPPLEKATEQVAEQPQAATRMTEEDREENKGKRRSTEGEDSKDSAE

VTTSVTRGKKRTWKKTASPASEETKGKKTAKMQVMEKALPEAKMEEECKEEQNNKPNTSS

ILARLKTGRKGKQDIEEPLDYNVEGYNVSVQQVEEDMKRFTSRRSVRKPKDGNDQETGSR

NLPEITRHSSLDLSESVVEKKVDMSSDEHMKYQKDDEFLTANGTTINVKRKGAKRNVKEE

ENKFVVDNIPGKRPTRNNRPNVTEKNNEEADDKQRITRRNSRQLEIVEIKPDNTEILDSK

RQNQSRKSPNIKKNLKVDESKETEKCEGKTRENTPKRTSRRTKKKCTEELNMEEWEKSKE

EADSKQRVTRRNSRLSESVESIPEDTDIVDSEQQDQSRKSRNIKKNLKRGEEEKLEGTNR

DNTEKWTSRRTRKECKEKLNMEDHEKSKVEPSIEQSEQEHTEAEGQDQSRKSRKTKKDLP

QPVINEEETLVETIRKDSVGRTSRRRKKSCIEEDLMDSEVLVISKPEQEDPPTKNVPRSK

RKNLKDEPKEGDGSMTIDSHEDVSPTGMRKSRRTTTKEELLKSTTLAPVRKRGQQPKTGG

DEVKRKKVNEEFEQEKTVGRRGQARQPKLEDENTDGNSFGKDSAVPFPSPSGNSRLRPST

SFESPTEGRTPRRAARSLTTSPYMSQRSIAAKVLFTGVVDPAGEETIRNLGGEVAESIFD

CTHLVTDRIRRTVKFLCALAKGIPIVTLDWLDKCKKSRCFLSPAQFLVNDKEQEKSFNFD

LSESLQKAKKKLLFEGYEIHVTPNVKPEPEHMKDIIQCSGATFLPKMPKVYKDKCIIVSC

KEDATRCKSAPSTIPVTSAEFILTGILRQEINPHAYLLSTGPEEALPTPAKRRR

>Leptobrachium leishanense

MPIVAYHRAPLYPACEQFHTNRLQEQQIRPEVRMDLTQRLISDDEEEDAPLDKNKPLALL

HVFKGTYGPAQDFNLYPGQNIIGRHASCHITLPSQSVSKKHAVLDVRPNCHILYDCGSLN

KTRRGKVALPPNVRFALNSGDFLHFADVACRYTIFTDPVKEPEKEKPAVQEVESDDDSFV

VPGTQAALAVEKTPGAAIRRMVQGVVLAKDSGDEDENKGEDVKSDARKGFDSSKGDHRTI

PGAFLSPTTDTVVPESDEEDDASVSQHRYPSLGLRSDADSHDTSLVMSSQSFFTSPTPSD

KKTRDPGPTREGSIASGSAGTEEKTLLGEIEGPPMEKKENGSDSDLSVIGPAAGEKDDLS

TGSSRNPPKSDQEARTESSGAERNVAEATSSSHEDVKGDLSASKEVLEENESPGTVPESD

SEVGEHTRAPPAETEAKSAIYAHAGNDDDDARASGTGKTAESEQHRDSRAGEGDDRGTAA

EGKAKPTFHLDSDTDVEEEGGSPVRAAAKVRKATAILSDSDSDSEEASATNSIAGIRVTR

SSKVTIDSDTDVEDVAPVKPGRRRKIARVPSDSDTDVEEEEEDVVPVKPGRRKRNIGRVA

SDSDTDVEGDVTSNQAETSPDSETDTNETAKSPLKAEATKEPVELNMDSDTDVEDATKSD

NGLFEKPMTSACVEADVGEDLKTSGTDAGSAAAIVPHMDSDTDVEESEEEHLTTSSTAGS

GTGVQTGNRETSADLEIPGEAGKASDDQDSGTSEKRHGSDAEFHMDTDTDVEEDDAASSV

SVKDDQGGAGEDRSASTGAPEEDMDTTGSNLASDASVVIEEQKNAAEEMSENLTETAKKT

EPEGTDLYMCETQCYLDESSGPEESDSHVPDLAEEPTQAFISSTYVEPDPFKRPADPIAS

LQISPVRANTSGDEYENAVAETQPYFCETETSGEECVQESETDTQPLPDPQTSEDDTQPL

SQYLAATPALGMATCVSPRHEVPAGVSEEVAQVQECVEDEESSADEVATLAYSLAVPDID

EEATQAFIAEDDAVIPECNDSLNGPDDSSASGQTREEDSMQPRGVTEPAAAAGDETKVLS

SDDQRQNQEHAEQEEAPPNAPPVDDPEESMQLHLSPSLVLTGQDAGDLRRSPPGRGTTDR

DQSEEGGKRQEVTRDAAESEDENSSREDEDDSLSRSEGQSKEENIPQPSTSGDSGDGWKE

RPATQGKDVEECKEITSSEATTSETEPDVAAVSSANEKADLPEESAEKTDEWGSRDSAVQ

DAAGAESDVQEEGNNQQAAPEKYFQTEALEETEEMQSQSEAEAKEPVDLEGEDVAAGVSP

SGDVEVKSEEEIPETGTLKASQRTSAEEVPGQTPEPPGDKPSKRKAKSTKRETDTNQEQQ

DTRTPEEGPARASRRTKSNLTGEGTREQKEADGKQEEVPVSRPVPRRMRKNSAGERASEQ

KEGAAKPEEAQASRPTSRRTRRNLEEEEDGKEKEETASRPVSRRTRTNSAGSGTSEQKEE

PVGRPSTRRTRKNSAESGTSEQKEEPATTPASRRTKKNPAGSGTNEQKEEPATTPASRST

RKNPAGSRMSEQKEEPATTPASRSTRKNPAGSRMSEQKEEPATTPASRSTRKNPAGSRMS

EQKEEPASTPASRRTRNNPAGGGTSEQKEEPATTPASRSTRKNPAGSRMSEQKEEPATTP

ASRRTKNNPVGSGTSEQKEEPATTPASRRIRKNSTGSGTSVDKSEDNPVSTRVSSRTRKT

STSEGTNVQKEDSDRSDGTQARGAVSRRTRKTADEGSTALKEGLEKLEKGPARPATRRGK

NSGGGTKGENEKSEQGPSGRAGPRSTRKDARAEEEEKAERETMAEIKDGRQAARTDLVEI

ENGDPVRQAVCTRAKRGLKGAEGEKSQLGAPSQKVELVGRRKGQDEEDPSTAENDARQDD

RSGRGASLRGRKNVGEDQKKRPNEAPQTEPRGSARTRKARAEESAAVQVATGGDAVEANE

ELQGDVATCPQVTTPLQTRKRGLGQKDGAVEVKRKKSTEEAEMGGGAKKTSPKPKGKRGR

PRSLVVEAAEEDDGDIGSDKEALNPVPSLAGSGRRTEKAASDNSPADSQSRRHTSRALHP

AGASRNYVSGLGAAPKVLFTGITDYAGEEVIQSLGGVIADSVFDCTHLVTDRVRRTVKFL

CALARGVPIVTLDWIAKCKNNRYFLSPNGFLVSDKEQEKKFNFVLSKSLQQAKRKALFEG

YEIHVTPNVKPEPEQMKDIIQCSGATFLPTMPKANKEKCVIVSCEEDAAQWKGVPSNVPI

TSVEFILSGILQQVVNPKAYLLDTSARPAAPTPAKRRR

>Hymenochirus boettgeri

MDNTQPLELVEEEHDPSADRTRPVGTLHLFKGNYGPAQDFTIYPGKNLIGRHASCDVILPAQSVSKQHAI

LEVHSDCHTLCDQGSLNKTRRGKAALTPHVRYALSDGDLLLFADVACQYTIETEGTSKEMDTSIGEIENE

ESEDDILVPGTQCALAIEKTPGVAIRRLGRGAVLAVDSGDENEDDFKYKCEDFNLVGEESKSPTSAFSTH

TFVPESDEENDTSVTEPQLPSLNLRCDSDTDAHDTSIRIGTILNSTQDYIMSQSLKEQLYFRKSEQLENT

KCNPTINKEEFSSRHLNADVTTLKLFHQDITFKNNSKGEREGADPGVKERISDATVKEHSVNTANVQNSL

LKETPQSNSDRDSEEKQKATIESADIILEGYMQNRKRAVFQMNSDTDTEESSTATSKRNLHTMQKSKDTT

KMQNKGGEVICLDSDTDIEDENCVHVNTDKTNAGKHQDSNTDVDVNIQMEEKISTFLHSDTGIEKDDKGM

TLNTVTKKQENADSDTDLEDTGGVFKRVTKKEETVHINLSQVGNYASNDINMEITESAKPPERLTEENKE

EIKVINEFQLNSDTDVEEDENAVETSNATREKKEDHNINGNEKKDQMVTVGDKKYDEEINFDSDTDVDEK

EMDNQIEANQKGEAALTMDTDRKVAAALTMDCDTDVDEKEMDNEIEADRKGEAALTMDSDTDVDEKEMDN

ETEADRKVGAALTMDSDTDVDEKEMDNETEADRKREAALTMDSDTDVDEKEMDNEIEADRKGEAALTMDR

VDREVRDLGASNTTEGGEGGGGSIEDKEACAVYDMATQCYLEPQEKESDIEDELNCAEEATQAFVFSSTW

TEPNPFKRPADPVGVLQISAVTMNSSEEEMDENEIAETQPFCCEIEQREGDCVNVTDTQDSELKRDVAWT

LTQGTISQDDTEPVSQYLKAGPSIAVCNSVSPAFVCPTDLNNKHDKDQTAGENTKRFDRNARNLEEDATQ

PHTQSLPTLDNSATQPSSLSKSVTDIGSVKFCNPAPVLPANVIKRCNTTDTLPDGNYVQNQIKIEVDTCS

YIIEDTKDGARGNPSEEVCISKTEKTDRVLDTTNGAQEISKHSEEILEPPNTPEKETYEVLEKAIGIKQQ

KRSKGIDERKSISQTGSIIDAKGRRRNWTEIMPETGMSTTEVTKGKKVAKKSETGKKATETKMEDACNEE

AGTTPETAISCFSTEVASTFFREPVLCETELTEEPVKRTVMEEIVEQPKDVITEHQGYSGTKENEHYSQS

QSTAIKNVINMEKGIEVKKTSDEPVPSSKPAGSRKGRSKTQENKDLLGEKKEETEPSIREGSKGRRLRGH

QEQDNAKRTILRRSRVKDDEEKKTDSRKEITSNKKKLELDNSLKEITSSSSTSLQLQNYDDVKELCASNT

RTQNEKNRSLNRFETVEKETDKLDLVKIPLKQSLRKQRQNYKAGENREDHKEIKKTANKQNIKKKDLKVE

GKVETKHNDVADSELERQNPENNLSDTIKEDTEKTMSRRKRNECPKDVTSEDNEKSKEDLDSEQDISTKK

ARAMQESDTTQDDASVEDSEKQGLFRKSRKTNKNVQRDKVKEKNDLGDTVRKECEDEAVMSKELLMVTKN

KSIRNEDPGACELEASTTNISGRTRKNLRGVISEEKIEEKTGGKQNTRRTRNNSKKEDKMRNENKTEKSL

SEKDSKAGITITGILNEQKEDTVMLSSPEKNKHSLEDSVTRNQEESLIAVTKDKLKMAPTTGRKRGQEPK

SIRQEGKKQKMEKESMQQEEPLLHVSRRGRARPISENETTERIINTKNVTLPSPSSTGNTSLNSSKAFES

PSAARTPRRTVRSLITSPYMPHQGISPKVLFTGVVDTAGEETIRSLGGDIAESIFDCTHLVTDRVRRTVK

FLCALAKGIPIVTLDWIDKCRRSECFLSPSQFLVKDKEQEKNFNFVLSESLQKAKKKPLFEGFAIHVTSN

VKPEPEHMKDIIQCSGATFLPKIPKVHKEKCIIVSCQEDAGCCKSTPSSIPITTAEFILTGILRQEVNPN

AYLLCSRLVEASHTQAKRRR

>Microcaecilia unicolor

MEQTQLLNWEDESLTCETLEGKDGNQETVGRLHVFSSSYGPEKDFWIHCGENIIGRQTTCHIPIPASSIS

KRHAVIEAEVDGSHFIYDCDSLNRTRRQKSTLKPKARYALNDKDLLLFADVACQYFILPQPNENGTLKAG

AAEHGLRGSPHARDTDSDDSLLVPGTQSGPAQPLAIEKTPAVKKMGYGGVLAKDSDDDSEESNRSRSRPR

QGERASQESTVSAEGRLVPNSSAVMTPTSSTVVPESDTEEGQESTTGKDQPEPQPEQRPQDLFHLDSDTD

VEDEDFSSKYSQRPFRKGGSSTSQNNDMEEKGTEQPAVGIEKCRMLSSGLDSDTDTDSGRTERSSLFLGD

SSNTSSDQDSDTDVERERERRPGVGEGDSRHANADQDSDTDMEGEGIARSGAGEGDSRQVNTDQDSDTDE

GIERPGGGEVRIRQVTAYQDSDTDMEGEETEKLSTGKEGIRQATADLDSDTDMEGEGTERLSTQKGDIRQ

ASADLDSDIDMDGEGTERLSTQKGGIRQATADLTSDTDVEGEGTERLSTQKGGIRQATADLTSDTDVEGE

GAERLSTGEGGLRKATADLNSDTDVEGEGTKRPSRGEGCIRQVTADLTSDTDMEGEGTKRPNTGEGCIRQ

ITADLTSDTDVEGEGITRPSTGEGCIRQITADLTTDTDVEGEGTKRLSTGGGCIRQITADLTTDTDVEGE

GTKRLSTGGGCIRQITADLTTDTDVEGEGTTRPSTEEGCIRQITADLTSDTYVEGEGTERLSTGEGGLRK

TTADLDTETDVEEEGAGRPGTGGESIGNPVVHHDPDTDMEQGQGTDATDHSTPVEQRHSWRRPGPCDTKT

ESEEEADTDDGSSFAMLDTQCYLSPASNIWEEEREASIDTGEEATQIFTSKASSDLFKKPFVLRPSTGLL

PTSECASSEKTDNWADDQLVVAETQPFCDNHDLSAISSKGRAENIEVEPTQAFLRQGESEEVSQEETQPI

ALYLSLRAASEPQRVHCGLDTMTVLQRQAVSENHAKANTTKPRSLPPLEKGEKPKICAQSSVLQLSVSEA

QTQTHAIDIKEENVTESSLHPEGEIPKESKEMAIAFSDETQTCVLQLSVSEEQTQTYSIENVTESSLHLQ

GEIPKESKDMEIAFSDETQTCAVTLAEEEQEIGTLKVCREKGEPQEQETAKILSEAKQVSKDNTLPTQPL

TASTISNHPCTEKLVDTLDGPDPERVWGEEKVSPKEARTCENTEKDSGNDATQEEVPLPPLIKGTDRTRE

EEEVKKEGRKKGRKEKKEEEKTTKATSDALEAGESDAVQLPEEGRSSTPGPVEEDKGGTPQEKKTHKKGG

LPCGYQRKQSKVQEEPSSAPGNQGSTVDQAAESKNGNRKKTSVPSQVLEPRAKSTRSTRRKTGPITEVKE

EEKETEQEHQPPAAPSRATKRTKKAVSESTANEAPAELEVPTLTRRTRARRGLVGTEEVPTKVTARPEHN

RKSVVEKGAVKEEEKKPQNPECVREKNPLGTRETKEPTALERHQVGADFMDLPHADEEDKKARTGAITAP

EKLPAAEEHTSAMSQETPGARGTRKRKPARPAQEDGSKPTQIVANIEPKRRRTRAAAAVAGVAGDDTANS

QKSLSREASALPSSEESKVGRERTRRGSASSKASQETLDFAQTTPSRSSQRLRLTVAMSENDILSKRDRR

SNSKDSSHGSSEKQDQDLATSSQESLVAGRRLRKQNTASSEDKAEDAEVVASQEGSTPRRTCRALGSSSS

PAANREPAVPKVMFTGVIDEDGEQVVCKLGGELAESVYECTHLVTDRVRRTVKFLCALARGIPIVTLDWL

KKCGRSGCFLCPSGFLVKDAEQEHNFSFSLSVSLQRARRGRLFEGYEIHVTPNVKPEPEYMKDIIQCSGA

TFLPNMPRTYKEKRIVISCPEDLAKCKPAFNATLPVANTEFMLTGILQQVIDLQAYALEAAADVQESTAK

RTSKRGTLTLDTASATTTTTDTKSKRRR

>Rhinatrema bivittatum

MEQTQPLDWDAENLGCETLESKDGNQEPVGRLHVFSSSYGPEKDFWIHFGENSIGRQDTCHVAIPAPSIS

KQHAVIEAEAEGSHLIYDCGSLNRTRRRKSALKPHARYALEDGDLLLFADVACQYFILPQPSENGAYQAN

VAGQGLRRSLCVRESDSDDSILVPGTQGCPALPLVIEKTPAVGAARKMGYGGVLAKDSDDESEEGDRSWS

HPHQGERGSRESSTLAEGRHALGSTVVSTPAASTVIPESDAEEVQETPAGKDQEEPQPKQQTVDVWPERI

PQDLFHLDSDTDVEDEDSGSERNWKKGSSCRRTQRSARRDWPGTSQAINTIMEGKGAAEMPPVGLKKSRR

FSPELDSETDTEGDRTERSSVGQGNNSNTSTDQDSDTDVERERERRDSAGEGDSRQSDTDMDGDVMERPA

VGEGDSRRPTADQDSDTDVEGEGMERSDAGEGDSRQTATNQDSDTDMEGEGTKRPDAEEEGIGQATANQD

RETDVEGEGTKRPDAEEEGIGQATANQDRETDVEGDETERPVAMEGGIRQDSDTNVDRERTEGPDVGEGG

IRQATADWDSVTVVEGEGTERLAAGEDGIRQSSAKQDSDTNVDEEGSKRPGAGEESMRQPCAQQVINTDV

EAGLSIAQEDHSTPVEQQSCRRSRPFDANTDSEEDDTDGSPRFALQDTQCYLYTASKVREEEMESSVAET

EEEDTQVFASRPSTSVLFKKPFFLRPSEGFLPSAGFTSLEKAEDWPDDQLVMAETQPFCNNPDLNAISSR

SGAESAEMEPTQAFPQKGDSEEVSQEETQPIALYLSLRAASEPPWPVRTTPILKHQAVSEIQNKASTIEL

RPPPPLVKGEENPISIQPSNLRLSLSEEETQMYTLQVEENLTEETVHHEGQTAKESKETEPTLDVESQIY

TVNVAESLEACRKEAKPPEQETLEDFMETDHMPKEDIQTMLPQTSPAAHHLLVPGDDVPTLTAMTISNHS

CAERSGDAVDNLNLKEEWKEEKVSPAKEKTCRNTCQDSRNDATQGEVPQPPLVKDADKTGEKGEEKEGRK

KGRKEKKHLNEEENLSKTTSQALEDREASIIQLPEEGVSSTLDPEKEEGGAPKKDVSKRNWLPKEKEELA

PPRDHENTVDQAHGRRRGVKQQTAVPAEALEPKSRNTRRKTGPIDETERQLQPTPFQAQKRMKKSISEIT

ANEPPVVPEAPTLTRRTRARRGFTGPEEEPKEVAVLPKRNRKSLIAEKSEVEEEAKEPQNLEEKNEGSSL

GTGEMRDKPTLKRRQVGVGTVEPPREKEEDKGPRTRGTAASSTAEEHITTKSQETVSGRETRKRGPTRPT

QEEGAKPAPMDANVGQKRRCTRVAAALAATNSRRRSREAVASSLSEESEVSRERARRESSSSKSSLESLE

SAQALPVRRSQRQRLTATRNKEDMPSTTDWENNSRSSSRGSTGKQDQGLASSSQGSLTTGRRGKKQSTAS

SEGGAEDVAVVTPKEETTPRRVCRVHGLMGSPRINRGQAMPKVMFTGVIDEDGEQVVRRLGGKMAESVHE

CTHLVTDRVRRTVKFLCALARGIPIVTLDWLEKCGRKGYFVCPYGSLVKDAEQEQNFSFSLSGSLQRAFQ

GRLFKGYEIHVTPNVKPEPEHMKEIIQCSGAKFLPRMPRTYKAKRVVVSCPEDLAMCKPAFDVFLPVVHT

EFMLTGILQQVVDLQAYALAPATNTQESASKRSSKPGGCGR

>Bufo bufo

MDDTQRLDYNDDEEEPSANRDRPMGNLHMFAGIHGGTQDFPIYRGQNIIGRHANCDITLPAQSVSKKHAI

LEVRGDCHTICDNNSLNKTRRGKTALAPNVHYALSDGDFLLFADVACRYTIVKVQAEATEVEDSEDDSVL

VPATQGALAIEKTPGAAIRRIARGIVLARDSGDEENEEEEEGQTRWHEGGSGSVRDGHKTSGSGTTFPPD

ADTIVPESDEENDTSTSEMPLPSLSLRCDSDTDTSRRSSFVPSSQSISTPLPKSKHDGRTAVDKEEMIPR

KPEDGEIKTSSAVEENQEEQKASTSGGSVSQPEVGIVSTVESERMLQHKALKEPCSESKPASSPCVEVDN

VALNRSENVQETPSELKPNVDVKVDHVSLDSAANIQEDTTTSGSDVNTAETSEIQTNKSKEEGDSRSSGS

REAAFHMDSDTDEDEAESDLEKSKSLQVAYIEKSEKNASVPVTCRKEGDSDSDTDVEDDQNVKKSKSDND

EAKRTMGEPIKTEGTPGLSLDSDTDDDDDADEPKPLENKADQDSRSNTDEKKEGFHMDSDTDVEEEVFTA

EVKEAKPEPLKTSAKETKGVLNLDSDTDVDEEDNVSSRHMTHGLQDSSTASTNVQDATGVESDTNVDEDE

QIPGGAKDNTDVVEAKEALHVGSDTDDDNDDRLPVVPPLEKVRAPEVSTDSAVKDTEIAKEPADLHLDSD

TDVEESDARAEEDEMDTTGSQEEADQKTSVSSVNEIAAVSGSSAEETKNIPTEVSMDEDETQKSDSESVD

LEMMPTQCYLEPEAESELPDEEEEEATQAYIFSSTWAEPNPFKSPADPIGVLQISSVTSVNTSEEEIDEN

ALAETQAFCTEAERVGPSVQETPELVERSLDESMQSTTSEKESGKEVQQLSHQTMSPDATQPVSQCLSSR

QSEETGTWMHLKREVPASVWIRGIQQGGDSTDLEGGSDATQAEDQSLNLELEATQIYGVESPSEQKPLAH

TEIQLVPAAGEDKETPTMPAAGKDKETPTLPAAGEDKETPTVPAAGEDKETPTVPAAGEDKETPTVPAAG

EDKETPTVPAAGEDKETPTVPAAGEDKETPTVPAAGEDKETPTVPAAGEDKETPTVPAAGEDKETPTVPA

AGEDKETPTVPAAGEDKETPTVPAAGEDKETPTVPAAGEDKETPTVPAAGEDKETPTVPAAGENKETPTV

PAAGEDKETPTVPAAGEDKETPTVPAAGENKETPTVPAAGENKETPTVPAAGEDKETPTVPTADKDYKEP

NVPAETTEQPLSDDIQASDNDATQAYSLDVPGSGSGTQACVLSDETTSDATQATNVYTVATEDNAQVAGP

SVPNAEEVVPEKSTESKKKLPLRKGLSRSKKKVESAKNVPQTSSEIEECPGKDPVGPGSLKEEPGEQGAG

EPQLSEPGIQEVQVERGLRRGGRRIAESQTKTTVKEEIHVATTSRKTTSKTSDDEPSASGINEKRGRRQV

SRKTAVKVVDEVYKEEHKQSSSNTEEAKEGSAPEILPINSPEEIESLNAEDHQKGNTAEQTSKESSDQSS

LENKDILSTEAFTSSAVSESNNPMESNDNSADKESDSRDTNVNEKPGRNKRRVATRLESEAADKTEFTDN

LEKEKDESRGRCNVRTTRKTDTSKQSSESPQSQQITQEQSQGRKATRKSRTKPSGPQSDKVEDTTISVTD

ENKKVDEICPADSVSGNEKPRRTRRSLKEEIEEESENLQDRDKKGKNVRSASVKDAEPRTKDKEQGNLED

LAASNKISRRTRNNPKGETTKPEQVKEENEVVKKSRRTQKNLKEEQKTEDNTETQGTSSSETPTSRRTRK

ENREEDSVVKEETVKETTPRRTRRHSKEDSIKLEDDQTKRSRRTRRDSKEEAKTQESVEESVLEENKEET

VGRKTTRKTRKNVKDDEKTILHEESKQTEDNSKSERSSRTRVKASDENRELHEEGVNKGVEESQKLSPHK

EKSKPAVGRGRRAAKKEDTPEVSTPVASRKRGQAAKAEVEIKRKKSDEGEEQEKLEVVETPKRGRPRKLV

TQTESPRAGKEISTPDPSPSRSTRQRSSSALSNPPEAITPRRTNRMSTSTTSPYVAQSGSAPKILFTGVV

DTAGEETIRSLGAEIAESVFDCTHLVTDRVRRTVKFLCALARGIPIVTLDWIDKCKKSGCFLSPTGFLVN

DKEQEKNFSFTLSESLQKAKRRPLLEGYEIHVTANVKPEPDHMKDIIRCSGATFLSKMPRSFKDKCVVVS

CPEDAARCKSVPASVPITSAEFILSGILRQEVNPTAHLLNPAVQDSGPPPAKRRR

>Geotrypetes seraphini

MDQTQLLDWDEESLTCETLEGKDGNKEPVGRLHVFSNNYGPEKDFLIHCGENIIGRQTTCHIPIPASSIS

KRHAVIEAEAEGSHFIFDCGSLNRTRWRKSTLKPQVRYALEDGDLLLFADVACQYFILPQPNENGTCQAD

AARQGLSGSPHAKDSDSDDSLLVPGTQGGPARLLALEKTPAVGAVKMGYGGILAKDSDDDTEESDRSRSY

PQQEERASQESTVSAEGRHVLNSSAVFTPTTSTIVPESDTEEGQENTTEKDQLEQQRGAARPEQRPQDLF

HLDSDTDVEDEDVNSKHEQRPFRKGGPSTSQNNDVEEKGAEQPAAGLEKRRMLSSGLDSDTDTEGGETER

SSLELGDSSDTDVEGERERRPGIGEGDSRHANTNQDSDTDMEGEGTERPGAGEGDSRQINTDQDSDTDEG

IERPGGGEVGIRQTTEYQDSDTDMEGEGMHRPGAAEGYSRQVSTDQDSDTDEGIVGIRQATAHHQDSDTD

VEEEGTEKLSTGKEDIRQTTADLDTGVEGEGMERLNTGKGGIGQIASDLTSDTNVKGDRTERLSTGEGCI

RQIAADLDSDADVEEDGTERLSTENGSLRNATADLDNDTNMEGEGSRRLGTGGEGTIKLCAQQNSDTDVE

GAGLKRLSTEEERIRQLCMLHDTDMEQELGIGVADHSTPVEQRHIQRRPGHRDAKTESEEETSTDDTEEE

EATQIFTSRASTSELFKKPFVLRPSVGLLSTSDCASSENTDELADDQLEVAETQPFCDNHDLCSISSKDR

AEDIEVEPTQAFLQQGESEDVSQEETQPIALYLSLRAALEPQRHHCGLDPMNVLQRRAVSDNHANSTESE

PLSSLEKGEKPRITAQPSALPLNISEEQTQAYAIEIAEENVTESSLHPEGEIPKESREMESAFDDETQTY

DVSLAEEVQATETLKVCREEAESQEQEIAENLSETEQVSKDNTVTTLPKSSAATDNVTQLLTASTMNNHL

RTAKLVDTLDGPDPKRERGEEKVNPEKARTFENTEQDFGNDATQEVVPLPPLEKDADRTREKEKVVKKGR

KKEKTEKKCLKEEEKTTKATSDAAGIGESGAVQLPEEGRSSTPGPAEEDKGGTPQEKKRRNKGGLPGGYQ

RKQSKVQKEPASIPGDPGSTVDEAAAESNSGDKQETPVPSQVVEPRSKSTRSTRRKTAPITEAKETGQEH

QPSPAPSRTTKRTKKAVSESMANKTPAELEAPILTRRTRSRVGLAGTEDVPKEVAARPKRSRKSAIVGKE

EVKEEETPPQNLECVREKSPLGTEATKEQALLERCRMGADITALPYEDEEDKGARTEATVASEEKLPAAG

SQETTGARGTRKRRPARPAQEDGSEPIKINANIEQKGRRTRAGTNSQKSTSKAAAAVPSSEESEMGPERT

RKGSASSKASQESLDFVHTTPSRSSQRSRLTVAVSENDIPSKRDKRSKSSSHESSEKQDQDLAPSSQESS

AGGRRFRKQSTVSSSEGKAEDSDAVAPQEGSTPRRTCRALGIGNSPAANRDPAVPKVMFTGVIDEDGEQV

VRKLGGELADSVYECTHLVTDRVRRTVKLLCALARGIPIVTLDWLRKCGRSDCFLSPSGFLVKDAEQERN

FGFSLSMSLQRARRGRLFEGYEIHVTPNVKPEPEYMKDIIQCSGATFLPNMPRTYKEKRIVISCPEDLAK

CKPAFNAALPVANTEFMLTGILQQVIDLQAYALEAAAEAQESSGKRSSKRSALNIDTAATITDTKSKRRR

>Rana temporaria

MDLTQRLDWEDEVEETAVERHKPVGNLHMFAGTHGPAQDYPIYQGPNVIGRHSSCDITLPAQSVSKKHAV

LCVTGDCHTICDNGSLNKTRRSKMALEPDVRYSLSTGDFLLFADVACRYTILKKIEAETTIAEDSEDDSM

LVPGTQAPLVIEKTPGAAIRRMGCGAVLARDSGDEDEEVRRGWSDGGEGGSKDALRTSRPAAGAFFSPDA

DADTVVPESDEENDTSSSGTRFTSLHLHCDSDIDNRGTPTKESSFITSSLDKFTPAAVKEPAKEESSRLL

QTNKDEKPVGEQTDEEKELSSVGRDVVSDGELLEERKETSKDGAGDRSPCLGGTVVQKDTLDCGLKDGGT

KAINRSNSHEDKNLKNNAASIENLEVSSTSSHRLKDNDDETSRRDIAATTAIESTQEEDNLKSTEEKLKV

AADLCLDSDTDSDEDVPTTSGTDVKKKDDPEIKSDKAPEQSSVDGTSDAGVHMDRDTSVDEDKEKISAVE

HGGVGEINIDSDTDVEDDKEMSETGDGNSGEVHKVHSVSGTNVSTGFDIDSDTDVEDADITRSDGKVEEK

KEKFHLDSDTDEEDNGDVSSTKVRKGDLDSVAVVLLKETKDEIHMDSDTDVEEDVNVSSTDVTKGKQTLN

IAAQEGFHVDSDTDVEDVDVSSTVVTKEKQDFSKSVKTARDGLPLDSDTDVDEEDQGPGPSSAAVQSRTT

ESHIDELQFEMVKNEKKAVSVINKDAADLHFDSDTDDDDGTSTEPKQAGGSVKTDAEKNKTEVANEADAL

HIDSDTDEDDGTSTGPKKAGGSVKTDAEQNEAEAANEADALHMDSDTDLDEDDASMIINKPQTAETAEGE

LHSDGVSEGGDKNLVEVAGSSVGVMGEVPKADTSTGSVKSNEVEAPKADSQGADYELMATQCFLEEPQEP

EEDLPDEEEATQAYILSSTWAEPHTFKRPSDPVGVLQISAVTLDKSEEEVDENAIAETQLFCFGVDQSPG

SDFKETPQLEEQVMDNQQDTQPTSSVEGSRQKDQEPPHGEMIQEDTEPVSQCLSANPPADTGTLLHLKRD

VPAAVWMKGLQQQLETSDVKEDSTPEDLTEDEQDLPPLELEATQSFILEVPNIQEHRKEHHDPSSLAVHV

DTKLAESAEPATENPPKDPIALGPGVDVSATEILPTGLSIPERDASPVPSTESTPTGPSTLELANESPLT

ESSNLETGISAASLTEGPPTELSNLELDVSAVPATEIPPTELSNLELDVSAVPATESPPNELSNVEKEVN

EDDATQAYSLNLPVSDANVPQSNLEVHSTEDATQPCSSDVASIASKEAQSTGVSMLAVGEKETELTDTGK

GKPSSKRGLSRSRKKEQPERSTEISNEKAQLSEQAAPAAHSAPEETAELRAGELGQSGPRNVASETRRKT

STEDSGKLTENTEVEEEQSEGTKKIADEETSRKGRTSRASAKEEPPQVSTPVANRKRGQALKADVNLKRK

KSDETSEAEEVRPAGRRGRPRKLSTTDDSQTSAGGSRISNEKAQLSEQAAPAAHSAPEETAELHAGELGQ

SGPRIVASETTEEIEPEKDGRRRGRRKATEDSETKAKTKNELVVPTASRKRSSRIALEAEPSTSGNNEIS

RKQPNRKSIVKMQEEEKQDESKKDFGSASEKCKKSDVFETLSDNHPEKIGLRSEFENVTGPTSMELIVGQ

MESDQERDVTSTLLESNRDKKVSTSSNNTEKNESTIVDLPEQLETISAPQNKKPVRGKRQAATKAKQDNT

QETISNDKTESETSGTNLETQDDFKIPTPRTNDLQDGGNKGPRGRRTRTSHISTSELLEHEKPKGADTKA

SQNEEANKTSPGDQNSADTRSTRQRRNVKEEAKEDLQKEEQIGRSKRTINLGGNQKNKETNSERTLEDNL

GRSSLPKRTRKNSKEETPKPEPDVPEVEKIEEDNMAKLPRRTRKGYKEEQKLEENKGSLESSNTAEAQTT

KRTQRSSKGQDIVDEETVKETVTRKGRNSKSEDAQLGEDQGKRSVRTRRLSKEEVVEETTTDTTKENAAG

QNTSRRTRRNVKADEKTESEHAGDIIPDKELSQSERSSSRTRRKTSTEDSGKLTENTEVEEEQSEGTKKI

ADEETPRKGRTSRASAKEEPPQVSTPVANRKRGQALKADVNLKRKKSDEISEAEEERPAGRRGRPRKLST

TDDSQTSAGGSREMSTPEPSPSRSSRLRQSPALSNLPEVLTPRRTGRLSVSTLTASPYIPQSGAPPKVLF

TGVVDAMGEEIIRSLGGDIADSVFDCTHLITDRVRRTVKFLCALARGIPIVTLDWIDKCKKSGCFLSHTK

YLVKDKEQEKNFNFVLSQSLQKAKRSPLFEGYDIHITPSVKPDPESMKDIVCCSGANFLPKMPRTFKEKC

VIVSCPEDAARCKSVPSKIPVTTAEFILSGILRQEVNPSAYLLSSEMEEDNAPAPAKRRR

>Pleurodeles waltl

MGIDGVILKWFESFLMGAPGGDHGRGSMEQTQVLDWDETLLSDDGTEFSQEPVGKLRLCGGAHGPERDFW

IHVGDNIIGRHENCHITLPAQSVSKKHAVIEAEADCHTIYDCGSLNKTRRRKAILKPHIRYALADGDLLL

FADVACQYFILPSENNREESTSPQHQAVQKQEQGSPVQAAASREDAPCEAPEDFDSDDESVLVPATQANA

VKSLVIEKTPATRRMGYGGVLAKDSDEDDNDGENTIYPTGRSRGSEKSTPSRECGDLCTSVALESPYAAT

VIPESDDECAETSAVSAPSMHLQYNSDTEAEESLVRPHSAVKPCCNTALPSQTDGPSPRGMEVVASVQME

LLNVDQGKTAGSHSGEALEQRTLHYHLDSDTDVDEEEEMKANKGGQKNAPRIEQTAAQLHVDSDTDVEEE

PDAGTNGDNPGSKEKNENLGNGSAAPANPTAAELNMDSDTDVEEEVDTTSKAAMPVVDLGSDTDVEEAVD

APHGVDLLRGSSAAAKHDNYTDTEEDGVSPGKAVSTSHQPAQDSDTDVEDKSLTPEKATVQQNNAQDSDT

DVEEMVGTTSKSPIKKKGARVSSRTQTSVVPESELLRGEDDEDTDADEPTEPTVKDDSDTEADDSPSTMA

LEATQCYLDAAAGEVDDEHSSAVSGDEAEATQAFVFKSPPVKPPTFKKPSVPSPGGSALPMPVCTSSEKE

DLSDEEQFAVAETQSFLRGPDISDIDLEDEPTQAFFVGGGTRLQGPSTSTQKVAGSVTRIASNDALIAHS

IEVEKEEEEATTLAEAETQLFFNDNLEAAPGGPIRPPEDPSQDETQPIALYLGLQAGHTQPTEACTSSPV

QRVTQPLLVRETHQGQQSPIQHRATARGNSQTNKNVFTVNPIKGPAPSMEKTITFCADSASLLESQEPQK

QSVESGQPDESTQHLSLILSEEPTQAYSIDVSEEVHPEPSLEPTGSKHAELSSNLGNAEEISMLPKVCLD

GVVAKASIEEDGAPTQTLESPVHDGEASSELAPNQETAANPSILPQISQASEPSDVGSTSAQEVQPRSDH

STHAKKKQPSLAKRGRKKKATVEEEEDAEATTSGPTPEPAEVSTHPAKNETTQLLDVEPQANEGPQTARG

RPARRASATSSAPARSKKINDTLGESLQGQEMQPLAETEDDQVGRKGRRGMGLRRRAAIEAPQYEAAAEA

DPLPGLYIPEVCTEAPVTTGPLAPGVEASVTLSVDTPAAMHTPTSHVEALHAKPVSTGDAEVVISNQLPV

VKNAAMISNPPMTSVEQASIDPPPVNATSTCADPTDAEEADFTATPVMQRRNAKRGEKTASLPTTEQSVT

LAEPETRGTRNRRKASATIAENREEQKPELVPARGRGRQKKEVSSDVSEKQQDSVVVPVDLPAPTIASRS

RKSFSGGITEEVVTPTVSVLSRTRRRGPVEKEGTREEHDQPKASGARKGTKKVPTIEATKITELESPSEM

SMTIDPTAEQTQEAQTKDSHLSNRHEGRTCTAEQPQSTGAQLVTKSQEGGSLEKKDLVGSQAASLSMDQA

VPNDQMEDINAEQALKSIETATSDQTGYASVRTPRKRRTVGNGEQVTENVASSTSDAHASRKRRTIGKEK

GVMEKDEDVQQSKKTDAKEEVEVEAGMFLDIQTIKKRGTRKTIEETRSAIQTSGKKGQKEAEASTLAPQA

SNKKEETADACEGIMNTAEKVATTYSRNRRTIQVGPEAAEEVRENSLLCREPRKGGSREAASEEVAEVQN

STSSTPLSRKRGATKQSEEDSNLGLSVSKEQKVHEIDSASNSAASSAAGDEKSERELEKPTEAAGRRSRK

QPSKDLAAYNRAEQTQSSEHARQTEDIVPDTAIAKAEAHSSQSGRNLEGTGKKAEEHQKLDSGVRGAVLK

GGAAGRRKRQNKSEEGGQDSADHEESTEQKRRRNNTAPNEVAEASATIEEAENTQSQGNVESTASEGTRS

QKQAATSKKAKSQAEATQPDVLQVRGRGSQRKNARKTVEAKITEGEQLASTEAAEVFESVPSQEELHSLA

SSRSSSRGRKQAQQTDSSQESVAPGGRHRKKSSASSEGKAEEVSAPPQADTPRRRSRTPNLSHSPGVFKE

HSAPKVMFTGVIDETGEQVVQRLGGELADSVYECTHLVTDRIRRTVKFLCAVARGIPIVTLEWLEKSGKN

KCFLSPSSFLVKDREQELNFSFRLSEALQKAQRQRLFEGYEIHVTPSVKPEPEYMKDIIQCSGAAFLPKM

PKEYKDKRVIVSCSQDLAKCKPALGASLPITNSEFILTGILQQVVNVDAFRLEGTPSGAEASKPQGGKRA

SANCSTPPPATTKRRR

>Engystomops pustulosus

MDETQRLDLDDEEETSFNRDKPVGNLHMFAGIHGPAQDFSIYRGKNLIGRHASCDITLPAQSVSKKHAIL

DVTGNCHTICDSGSLNKTRRGKVALAPNVRYALSDGDFLLFADVACRYTIPVNVEAETTGVVESEDDSML

VPATQGALAIEKTPGAAIRRIARGAVLARDSGDEEEQTPWNEGGSGSFKDSHKTLGPGTICPPEADTVVP

ESDEEIDTSTSEVRLPSLNVSCDSDTDTSRKSSFVPSSQNISTPLVLKNPSKHDERTWTAMDTSREESSK

TTCAENKLRLGMEENRGYEQAVESTSACSVTVPCVEVAGAVDENTKKEQTFEKILEDDTLTKPSVAESDE

NMVSSKIETAQEAPCELNSDVSAKLKEDDNNREEDKTTSEAKTSVLQSNKTNEGGSSHAVFHMDSDTDDD

DDEAATNGSHIEKSRSREVADTIDSEKKASPPLLLGNEGDIDADVESDKNVVKLKRDREELSKTEVVTGL

AIDSDTDDEAVDIPKPVEINAAQDLTSKAEEKKEGFYLDSDTDVEEDVSTAEVKEAQPEPSKVAVKEVTA

ALNLDSDTDVEEEADVSIQDTAHKPQVSSNESTNVQEVSGVDSDTDVEEETDVSIQDTTHEPQISSTEST

NVQEATGAGSDNDVEDDQIQNVNLEKTSTDDDPKTAVVAVALHVDSDTDVDDDDEELPVTPRTVDASESS

TNADGAKQVISLNSDTDVEESGGSSIVPRPEDEGNTSAVQKKADVDKSSVSSVIEISGISTKETQNVSAE

ISTEEDETQKSESESIDLEMMPTQCYLEPQEESDPAEEEEEATQAYVFSSTWAEPDPFKRPADPISVLQI

SSVTLDTSEDEIDELEIAETQPFCTEAELVGPSVQETPEPVERSVDETSSEEESEKETQQVSHETLSSDA

TQPVSQCLSTKPAEDTGTWLHLKRDVPAAVWIKSLQQEENTEDVGEASETTKDATQADEQSLKLELEATQ

LYVEEEKPPETQTPPAANESKGLKVPEKAAEQLEQPFSDATQAKDDDATQTIDIDATKDEDSTQVIDNEA

TEAKDDDATQDKDDNDTQTIDSNAAETKEKDATQATDNDATETKDKDATQAIDNATETKGDDATQAIDND

ATETKDDDATQAIDNDATETKDDDATQAIDNDATETKGDDATLAIDNDATETKDKDATQAIDNDAIETKD

DDAIQAVDNTATQAKDDASQACSMSGDDAVQVTSVNDPSVSSTELPDERKEKPSSRRGLSRSKKKKEPAQ

NVLVPSETEKLPDTAPAEEPGEPSAVEPETEIKEVHEEKEVKRRGGRRTAASQDTTNEEAVTSRKRTSKK

SSEDEPSGINEPRGKKQVTRKIAKKEGNEELKEEHSNTVEVTSEVTQSDKKGRVVKPTSAEGGDQASVEN

KEIVSTEPSKSPIVVEGEDLIEPNDSGAKSDSLKKPARNKRRVATRLKKADAEEAVNPEDVDNKNSLESQ

EETQGRKATRKSRVKAPEPESDKDEATTEDYKKVEDVSPAEPVSENEKSRRTRRSLKEESKEESQEVQEG

KGKSRRSASIKDIEPKNKDKVQVNEDKPASNNMAKRTRNNSRQETSKTEQEVKENANIEDNEAVRKSRRT

QKNQKEEKTEDSIATQEISSSDANTTRKSRRGQRVEDSVTKEEIIKETTSRRTRRHSKEGDDVKVVQDQT

KKSRRTRKNSEEANPEDTLEESLAAEDKEEAAGKKNTRKNKKQVKEDQKKGAGDEESQPSEEKYKRSPRT

RAKAADGSQGEEVKKGSEESPKVSPQEKSKPGRGRPAAKKEDLPQLSTPVTSRKRGQTTKTEGEVKRKKS

DDGEEKPEELVVIETPKSRRGRPRKLLPETENTQTEKEISPPDPSPSRSTRQRPSSASSNPQEARTPRRT

NRTSTAATSPYPAQSGSAPKILFTGVVDTAGEETIRSLGGDIAESVFDCTHLVTDRVRRTVKFLCALARG

IPIVTLDWIDKCKKSGCFLSPNGFLVHDKEQEKNFSFVLSESLQKARKRPLFEGYEIHVTQQVKPEPDHM

KDIIRCSGATFLPKMPRSFKEKCIVVSCPEDAARCKSVPASVPITSAEFILSGILRQEVNPTAYLLNPTA

QDTGPTPAKRRR

>Equus asinus

MMMEDTQAINWEVEEEEETERPSESLGCSLAPVGRLRIFSSAHGPEKDFPLYLGKNVVGRMPDCSVALPF

PSISKQHAVIEILAWGKAPILRDSGSLNGTQILRPPKVLSPGVSHRLRDQELILFADLLCQYHRLDAPLP

FVSRGPLTVEETPKVQGGTHPQGLLLAEDSEEEVDPLSERHVVKEPRTTSSSLATVVPESDEEGPSPGPG

GPGPPFAFNLDSDTDEEESQQPATGEAFSAAMTDATVETEPPKAITTEIQLEKDQCSVEERDNATKVKRD

ARNEVVPVGVILEKTQPAEEDSDTDVDDESRPPGRPAEVHLESAQPSGFIDSDTDVEEEGIPTTPAVVPM

KKRQVFHGDDAKSPGAPGLANLQESPAGSDTDVEEGEALLTVPLERSQASMVIDSNTDDEEEVSAALTLA

RLKESRALTWHRDTDVEEDKAQPVVLLEQSQTSARRDSDTDVEEEGPPVEKRGTVPKDCTDKAHSEKSQP

PLGDSDIEMEKDKSSPAVHLERSEASATVDVNTQVKEEVLPGPAVTPLEKHQVPVAWTNQTDVEADRGPA

KLPMVCLEEAQPPPVGDCEITSLNASAVTDVRKSQFPTGGDAGTEWAVAVLEQERALEAGAQDGSPVALV

EQGLLPVSRENLTDLVVDTGTPGEPTQPRREGAQTPKEGKREPRMDGTKDSADARDVLKAEKSTFKVPAY

SVSDSEDLDLQATQCFVEKEGQSLEAVQSTEDEPTQAFLLSPPQEPGPSRCSFQAEEKNAVLSQTYGATS

VASFLKRLPRELLYEGALDELWEVLATQPYCPRESEASETQPIAAHLEAHGSCPSPPRATPGEQHPESPV

HAEPLGIQGRGMQTVEKDMGTPGETADRVNPERGPLERATKKPPPEGERKDVMGEQELTRGLRDSQQKQV

LARDTQRQESDKKVTSASPESGMESLKVEIETAREIQEKEREKQTLAREIFEREAEKLVPGRVCEVGGLE

VKVSKVIQERGPEAGEPERGTQDQEGQASSPTPEPRVGAGGLQALASAVVASGSQSGGGRGVPVSPRRQE

RDHLNCKMPPAEKASRGDQESPEACLPPAVTEASAPLQNALMPQSQKHPAPQPLPSLELPIPRARQNGSQ

GAPEIPPSELEPLHPKPKVRPRGSSRMLPSPMSSIAPESHPTTPTDQPVSPEPTSRATRSRTYRSSEMTP

APVVPTAPELQSSIFKDQPVTAKLTSRATRGRTHRSSVKSPEPVVPTAPELQPSTSKDQPVTPEPTSRGR

THRSSVKAPEQVVPTAPELQPSASKDQSVIPTPISRATRGRTHRSSVQTPEPVVPTAPEFQPPTPTDQPV

TLELTSRATRGRTHRASVKTPEPVVPTAPELQPPTSKDQSGILTPTSRATRGRTHRFSVKSPEPIVPIAP

ELQPSTPTDQSVASEPTSGATQGRTHRSSVKTPELVVPTGPEFQPSTSINQLVTPKPTSQPRTHRSSVKT

PEPIVPTTSELQPSTPTDQPVTPKPTSRATRGRKHRSVNTSELIVPTASELQPSTPTDKPVTRKPTSRAT

RGRTHRSSVKTPEPIVPTAPELQPSTPTDKPVTCKPTSRATRGRKHRSSVKTPKPIVPTASQLQPSTPTD

QSVTPESTTQDIRGRKHRSSVKTPQPMEPTAPGPEPPSHTDQPVTPEAIAPASQSRTLRTSIISAVPVPT

TPEFRSPVPTDQPIPPETIPQANCSRRPRATRKQGSPTAPIVHEPCSAPPEPNSRNQRRRAVRAAESLTT

IPEPAFAQLPEAPTHAPHIEKVEAAGTSGFTPEPQRKASQSHKRPLATLDLPPLQKRLQRGKVSQKTVFL

QEEEDDPTERPGKKENVVMPGPGKRKRDQAEEEGILSRSLRRTKPNQESTAPKVLFTGVVDVRGERAVLA

LGGSLASSVAEASHLVTDRIRRTVKFLCALGRGIPILSLDWLHQSRKAGCFLPPDEYVVTDPEQEENFGF

SLRDALSRARERRLLEGYEIHVTPGVQPPPLQMGEIISCCGGTVLPSMPRSYKPQRVVITCSQDFPRCAI

PSRVGLPILSPEFLLTGVLKQEAKPEAFVLSALEMSST

>Pan troglodytes

MYHRLDVSLPFVSRGPLTVEETPRVQGGTQPQRLLLAEDSEEEVDFLSERRMVKKSRTTSSSVIVPESDE

EGHSPVLGGLGPPFAFNLNSDTDVEEGQQPATEEASSAARRGATVEAKQSEAEVVTEIQLEKDQPLVKER

DDDTKVKRGAENGVVPAGVILERSQPPGEDSDTDVDDDSRPPGRPAEVHLERAQPFGFINSDTDAEEERI

PATPVVIPMKKRKIFHGVGTRGPGAPGLAHLQESQAGSDTDVEEGKAPQAVPLEKSQASMVINSDTDDEE

EVSAALTLAHLKESQPAIWNRDAEEDMPQCVVLLQRSQTTTERDSDTDVEEEELPVENREAVLKDHTKIR

ALVRAHSEKDQPPFGDSDDSVEADKSSPGIHLERSQASTTVDINTQVEKEVPPGSAIIHIKKHQVSVEGT

NQTDVKAVGGPAKLLVVSLEEAWPLHGDCETDAEEDTSLAASAVADVRKSQLPAEGDAGAEWAAAVLKQE

RAHEVGAQGGPPVAQVEQDLPISRENLTDLVVDTDTLGESTQPQREGAQVPTGREREQHVGGTKDSEDNY

GDSEDLDLQATQCFLENQGLEAVQSMEDEPTQAFMLTPPQELGPSHCSFQTTGTLDEPWEVLATQPFCQR

ESEDSETQPFDTHLEAYGPCLSPPRAIPGDQHPESPVHTEPMGIQGRGRQTVDKVMGIPKETAERVGPER

GPLERETEKLLPERQTDVTGEEELTKGKQDREQKQLLARDTQRQESDKNGESASPERDRESLKVEIETSE

EIQEKQVQKQTLPSKAFEREVERPVANRECDPAELEEKVPKVILERDTQRGEPEGGSQDQKGQASSPIPE

PGVEAGDLPGPTSAPVTSGSQSGGRGSPVSPRRHQKGLLNCKMPPAEKASRIRAAEKVSRGDQESPDACL

PPTVPEAPAPPQKPLNSQSQKHLAPPPLLSPLLPSIKPTVRKTRQDGSQEAPEAPLSSELEPFHPKPKIR

TRKSSRMTTFPATSAAPEPHPSTSTAQPVTPKPTSQATRSRTNRSSVKTPEPVVPTAPELQPCTSTDQPV

TSEPTSQVTRGRKSRSSVKTPETVVPTALELQPSTSTDRPVTSEPTSHATRGRKNRSSVKTPEPVVPTAP

ELQPSTSTDQPVTSEPTYQATRGRKNRSSVKTPEPVVPTAPELQPSTSTDQPVTPKPTSRTTRSRTNMSS

VKNPESTVPIAPELPPSTSTEQPVTPEPTSRATRGRKNRSSGKTPETLVPTAPKLEPSTSTDQPVTPEPT

SQATRGRTNRSSVKTPETVVPTAPELQLSTSTDQAVTPKPTSRTTRSRTNMSSVKNPESTVPIAPELPPS

TSTEQPVTPEPTSRATRGRKNRSSGKTPETLVPTAPKLEPSTSTDQPVTPEPTSQATRGRTNRSSVKTPE

TDVPTAPELQPSTSTDQPVTPEPTSQVTRGRTDRSSVKTPETVVPTAPELQASASTDQPVTSEPTSRTTR

GRKNRSSVKTPETVVPTAPELQPSTSTDQPVTPEPTSQATRGRTNRSSVKTPESIVPIAPELQPSTSRNQ

LVTPEPTSRATRCRTNRSSVKTPEPVVPTAPEPHPTTSTDQPVTPKLTSRATRRKTNRSSVKTPKPVEPA

ASDLEPFTPTDQSVTPEAIAQGGQSKTLRSSTVRAMPVPTTPEFQSPVTTDQPISPEPITQPSCIKRQRA

AGNPGSLAAPIDHKPCSAPLEPKSQASRNQRWGAVRAAESLTAIPEPASPQLLETPIHASQIQKVEPAGR

SRFTPELQPKASQSRKRSLATMDSPPHQKQPQRGEVSQKTVIIKEEEEDTAEKPGKEEDVVTPKPGKRKR

DQAEEEPNRIPSRSLRRTKLNQESTAPKVLFTGVVDARGERAVLALGGSLAGSAAEASHLVTDRIRRTVK

FLCALGRGIPILSLDWLHQSHKAGFFLPPDEYVVTDPEQEKNFGFSLQDALSRARERRLLEGYEIYVTPG

VQPPPPQMGEIISCCGGTYLPSMPRSYKPQRVVITCPQDFPHCSIPLRVGLPLLSPEFLLTGVLKQEAKP

EAFVLSPLEMSST

>Macaca fascicularis

MRIIDNVYPSQIMEDTQAIDWDVEEEEETEQSSESLRCNVEPVGRLHIFSGAHGPEKDFPLHLGKNVVGR

MPDCSVALPFPSISKQHAEIEILAWDKAPILRDCGSLNGTQILRPPKVLSPGVSHRLRDQELILFADLLC

QYHRLDVSLPFVSRGPLTVEETPRVQGGTQPQRLLLAEDSEEEVDFLSERHVVKKSRTTSSPVAMIVPES

DEEGHSPVLGGPGPPFAFNLNSDTDAEEGQQSATEEASSAARRGATIEAEQSEAEVVTEIQLEKDQPSVK

ERDNDTKVKRGAGNGVVPAGMILERSQPPGEDSDTDVDDDSRPPGRPAEVHLERAQPFGFIDSDTDAEEE

GIPATPVVVPMKKRKIFHGVGTRGPGAPGLSHLQESQAGSDTDVEEGKAPQAVPLEKSQASMVINSDTDD

EEEVSAALTLARLKESQPAVWNRDAEEDMAHHAVLLQRSQTTTGRDSDTDVEEEELPVENKQTVPKAHTK

IRALVRAHSEKDQPPFGDSDDSVEADKSSPGIHLERSQASITVDINTQVEEEVPPGSAIVHMKKHQVSME

GTNQTDVKADGGPAKLLVVSLEEASPPHGDCEIDAEEGTSLAASAVADVRKSQLPAEGDAGAEWTAACLK

QERAYEVGAQGGSPVAQVEQDLPTSRENLTDLVVDTDTPGESTQPQREGAQVPTGREREQHVGRTKDSED

NCDDSEDPDLQATQCFLENQGLEAVQSMEDEPTQAFMLTPPQELGSSHCSFQTTGTLDEPWEVLATQPFC

LRESEDSETQPFDTHLEAYGPCLSPPRAIPGDQHPESPVHTEPMGIQGRGRQTVDKGMGIPKETAERVGP

ERGPLERETEKLLPERQTDVTGEEELTRGIQDREQKQLLARDTQRQESDKNGESASPERDRESLKVEIET

SKEIQGKQVQKQTLPSKAFEREVERPVADRECEPAELEEKVPKVILERDAQRGEPKGGSQDQKGQASSPT

SEPGVGAGDLPGPTSAPVPSGSQSGGRGSPVSPRRHQKGLLNCKMPPTEKASRIGAAEKASRIGAAEKAS

RIGAAEKASRGDQESPDACLPPTVPEASAPPQKPLNSQSQKHLAPQPLLSPLSPSIEPTIRKTGQDRSQE

APETPLSSELEPFHPKPKIITRKSSRMTPFPATSAAPEPHPSTSTAQPVTPKPTSQATRSRTNRSSVKTP

EPVVPTVPELQPSTSTDQPVASEPTSQATRGRKNRSSVKTPEAVVPTALELHPSNSTDQPVTPKPTSQAT

RSRTNRSSVKTPEAVVPTALELHPSNSTDQPVTPKPTSRTTRSRTNMSSVKTPESTVPIAPELPPSTSTE

QPVITEPTYQPTRGRKNRSSVKTPETVVATAPKLQSSTSTDQPITPEPTSQATRGRTNRSSVKSPETVLR

TAPELQPSTSTHQPVTAKHTSQATRGRTNRSSVKTPEPVVSTAPELQPSTSTHQPITPEPTSQATRGRTD

RTSVKTPKIVVPTVPELQASTSTDQPVTSEPTSRTTRGRKNRSSVKTPETVVPTAPEPRPTTSTDQPITP

KPTSRATRGRTNRSSVKTPELIVPIAPEFHPSTSRSQLVTPEPTSRATRGRKNRSSVKTPEPAVPTAPEL

HPTTSTDQPVTPKPTSRATRGRTNRSSVKTPEPVEPAASDLEPFTPTDQPVTPEAIPQGSQSKTLRSSTV

SAMLIPTTPEFQSPVTTDQPISPEPIPQASCIKRQRATGNPGSLTAPIDHKPCSAPLEPKSRPSRNQRWG

AVRADESLTAIPEPASPQLLDIPTHASQIQKVEPAGRSRFTPELQPKASQSRKRSLAIMDSPPHQKQPQR

GEVSQKTVIIKEEEEDTAEKPGKEEDVMTPKPGKRKRDQAEEEPNRIPNRSLRRTKLNQESTAPKVLFTG

VVDAQGERAVLALGGSLAGSAAEASHLVTDRIRRTVKFLCALGRGIPILSLDWLHQSRKAGCFLPPDEYV

VTDPEQEKNFGFSLQDALSRARERRLLEGYEIYVTPGVQPPPPQMGEIISCCGGTYLPSMPRSYKPQRVV

ITCPQDFPRCSVPLRVGLPLLSPEFLLTGVLKQEAKPEAFVLSPLEMSST

>Anser cygnoides

MQRRRHGGLCTTGGTVQGPELRHPIASDSEDDETWRCPTTGNTASPQVVPESDPEEPDVCPDIRVLRKRR

PTPALGLRTDLAQSHLDPDVGGPKKRRLCPKTTPAPDVGNKTAISDIRPQNHSPNPKGQVDPDVKHLGGS

TVALGIESDTDDEMHPKSALFHPNRHQAALEASGDPNVEKRGPNPGAEGAQNGRWMLAVDSDTDVEEADV

LPSVGCPKMRRKAQRVPKAPGVEMKPPNPDAGSSKNGFQTLVVDSDTDVEMESSNSAVEDPQRGHQAFGV

DSDTDVEEGGANPAVGLAKTPQTTENTPNSSNIKMKSPHSAVKGPHEEHPPLTVDSDTDVEEDRANPDVV

CLPTPQTAQNAPKPPNIELETPNPDADGCQKENWMLVVDSDTDVEDNILPDVVSPKPHAMTHRDPGVETR

GQDAEGPKMRCRSPLVDSNTDVDEEPSSAEGRDPKPHQITQNAQRNLDAEMRTPSPHPREPQSGHEMVKV

DSDTDVDEEPSSAEGRDPKPHQITQNAQRNLDAEMQTLSPRPGEPQSGHEMVKVDSDTDVDEDPSDPEGR

DPKPHQITQNAQRNTDVEMETPSPHPGELQSGHEMVKVDSDTDVEEDGLNPDVGCRETHRMTPKIHNDSD

VAIKTPNPDLEGPQNRGQNLEVDSDTDVEGEDLNPDVPGLKNHKTPQNAQKDPTVVMETPNPDVVGLHRG

SMPPGSDSDTDVEGLEDIETPKTRRTTREVTGMGAKNAAAPDVDPRWHAWTKGGLNVKGTAPNPDVGQHV

DLNVDSDTRVEDNGVVPDVGAVQGGQGTPGDPDVAMAPLDPDVSPPVTPQSGSDTDVEEVAPTPYVRSLR

SKTRPRNPDVVDTAMGSDTNVGLTDPKCQKPVQNRQGLPPKSCSDGGVEGEVPKRHDLAPNTDAEDPNPD

DKVQQHNPQTSVMGSDTDVAPAGLAPKSPITAPNLSLGGDAAWGSRSAQATPHGDTDAEVAESETEGEWG

GLCHLCPFLDHLGSLATWVPSLVLLVPLSPSLATWVPSLPTASPPCPQVLFTGVVASQDMEVALGSLGGS

MATSVFDCTHLVTDRVRRTVKFLCAVARGIPIVTPKWLHESARSGRVLAPGSFLVRDSQQERHFGFSLSQ

ALSHARRHPLLQGYEVHVTPSVRPEPEQMRDIVTCSGGTFLPTMPCTYGPRRLVISCGEDSGCWAPALSA

RLPLASAELLLTGLLRQRLQLQDFLLAPPEIPPGPSGVPQDPPPKSPQPPPASPRRLRAPPSTQGRTRRD

PPSTRRHPQPRNK

>Aquila chrysaetos chrysaetos

MHRRRHQGLSTVGGPLSRLSPHPPEDPDLFLEPTQRFLLLAGQGWSPDPGPCLVSDSEDDKLPWRPALGD

AGSPRVVLESNPEETGACSDVLRPQKHCHTSARGHLQDVAAPGPDPDVREPKKHRFGRQTTPDPDVGKKM

AIPNVQPQNHSVNPKMLANPDVKHLEGSNLTLNIESNTGVKVPPKTGLFNPNRHWPTMKVSSNTDVEERG

PDPDVGCPESHRTAPGDPDVKTETTDRDVEGRQTLVVESDTDVEEDVSNPDAEPPKTHRATQNVLRNPHL

EMESPNPDAEGPQKKQWTIVVDSDTDVEENEVNPDAGHPKIHRIVCRGPDVEGETPNPSVKEPQKKYWNL

EVDSDTDIEEDWSQAALRPKSHKTPQNTRKDPTVVMETPNPDVGGLSRGSVASNGDSDTDVEDLDALPKV

GAPKARGTAPEVMDTAAKMAAAPDVDPEGRPRTNAHPDVKVSFPNPDVSALKAECPLVDVGSDTDVEDNG

AIPDVGTVQGCQGASGDPDVAVMSPNPDVSPPASPRESSDTDVEMLPPTPDVRGLRSRIRFQNHPHPDVA

GPTTGRDTDVKETARKRRKLSPKRQGSPPPIXSVARMLPKHHDLAPNPAVEAPNPGVGAERRDAETPVRG

KDPAVPPDVSAPKCPRLTPDLSDAADTDGGARSARTTGAVVTESDTEGWNPDPGPCLVSDSEDDKLPWRP

ALGDAGSPRVVLESDVEDTDVCPDVEERGPDPDVGCPESHRTAPGDPDVKTETTDRDVEGRQTLLVESDT

DVEEDVSNPDAEPPKTHRATQNVPRNPHLEMESPNPDAEGPQKKQWTIVVDSDTDVEENEVNPDAGHPKI

HRIVCRGPDVEGETPNPSVKEPEKKYWNLEVDSDTDIEEDWSQAALRPKSHKTPQNTRKDPTVVMETPNP

DVGGLSRGSVASNGDSDTDVEDLDALPKVGAPKARGTTPEVMDTAAKMAAAPDVDPEGRPRTNAHPDVKV

SFPNPDVSALKAECPLVDVGSDTDVEDNGAIPDVGTVQGCQGASGDPDVAMTSPNPDVSPPASPRESSDT

DVEMLPPTPDVRGLRSRIRFQNHPHPDVAGPTTGRDTDVKETARKRRKLSPKRQGSPRQSESVAGMLPKH

HDLAPNPGVGTERRDVETPVRGKDPAVPPDVSAPKCPRLTPDLSDAADTDGGARSARTTGAVVTESDTEE

DPDVFLEPTQSFLPPAAEGTALGWDPEQPTQPFCPPEEEEEEEEEEEEQPPREPPQEPPAAWVPPAEPVT

VTRAQEGTGTTAAPSEDVGGGPRRSQRLARSRGGGASGEGGASRVRGGAPPVTGPVRRSPRLQARPPPPE

PPAMKGRGQVEPRPPAKPRPCRAGPGPSQRQAQVGEPPEVTGPQLRPRGGAVSPHPQVLFTGVAASPEME

VALRTLGGSMATSVFDCTHLVTDRVRRTVKFLCAVARGVPIVTPEWLHKSTLSGHVLVPDPFLVRDSQQE

RHFGFSLAEALRRARHHPLLQGYEVHVTPSVHPEPELMRDIITCSGGTFLPTMPRTYGPQRLVISCEADK

GCWAPALGARLPLVSAELLLTGLLRQQLQLQPFLLLPVTPPPFSRDPPKSPQGFYSPSGVLSHPPEDPQG

IPGSPRESRKVSGRSSRNRRPPRETSRGNRRQPAAPQ

>Chiroxiphia lanceolata

MAREGPAEDPAVAPQKRPLCPQTPNPDVGGPKNGGRMLGGDSDTDVEGEEPHPDVGAARRPKMALNHPKP

PDVAVKTPNPDVLGPQIGPGQFLVDSDTDVEEEEPHPDTQNGGQMLVVDSDTDVEEEVTNPDVGATRRPK

MALKDPKLPDVAVKTLNPDVLRPQIGPGQFLVDSDTDVEEETQNPDVGTSRRPKMAPKVPETPGLGVQTP

NPDVSRPKNGGQMLVVDSDTDVEGEEPHPDVGAARRPQMTPKVPETLGLGVQTPNPDVEECKNGRGMLVV

DSDTDVEEEEPHPDVGSLKPPQMTLKDPKTPGVVVETPNPDVGAPKIGCGMLLVDSDTDVEEEATNPDVG

SVKRPKMALNHPKTPDVAMKTPNPDVGIPKRPRMTPNVPETPDVAVESDTDVEGEEPHPDVGSPKPPKMA

PKVPETPGLGVQTPNPDVEGSKNGCGMLVVDSDTDVEEEATNPDVGSLKRPKMALRHPKTPDVAMKTPNP

DVGGPKNHCGLLLVDSDTDVEDEEPNPDVGSPKTPRVTQNVAETPDVAVETPNPDVGTSRRPKMAPKVPK

TPGLGVQTPNPDVGMEQRTLTVDSDTDVEEEATNPDVGSPKTPRMTQNIPETPDVEEETQNPDVGTSRRP

KMAPKVPETPGLGVQTPNPDVSRPKNGGQMLVVDSDTDVEGEEPHPDVGAARRPQMTPKVPETLGLGVQT

PNPDVEECKNGRGMLVVDSDTDVEEEEPHPDVGSLKPPQMTLKDPKTPGVVVETPNPDVGAPKIGCGMLL

VDSDTDVEEEATNPDVGSVKRPKMALNHPKTPDVAMKTPNPDVGIPKRPRMTPNVPETPDVAVESDTDVE

GEEPHPDVGSPKPPKMAPKVPETPGLGVQTPNPDVEGSKNGCGMLVVDSDTDVEEEEPNPDVGSPKTPRV

TQNVAETPDVAVETPNPDVGTSRRPKMAPKVPKTPGLGVQTPNPDVGMEQRTLTVDSDTDVEEEATNPDV

GSPKTPRMTQNIPETPGLGVQTPNPDVEGPKTGCGLLLVDSDTDVEEEEEEGSEADVATQLFLPDPDEEE

GEGPDPDVATQLFVLAPDVEEPDPDVRPPKRAKRAPKGPDVEGGEGPDPGVAPQLFRPPPDVGEGGAESS

HLEPNLGPRGTKRGKGPQGDPQDPARRSGPAHQEPEAPPTPQVRRSQRLAGSRGGGASPGPAPTRKGRDQ

TVPKPHPSPKPRPQRRPAQEEEQEEGACPAEGTQRRLRPRATPGPAHIRVLFTGVVASPALLVALGALGG

VVASSVHDCTHLVTDRVRRTLKFLCALARGVPVVTPNWLLESSRCHRPLSPAPFLLRDPPRERHFGFRLR

EALDRARGHPLLQGYSIHVTPGVTPGPEAMRDIVTCCGGTFLDTLPRAQRPRCLVVSCPEDSGELWDRAG

AAGLPLVSPELLLSGVLRQRLDVTPFLLTPNPNPRGQGNPKNTARDTQNPKNTARDPKNPKNTARDPKNP

ENTARDTENPKNTAGNTLNSETPKNAPRDPKNPKNTARDSPNSETPKNAPRDPKNPKNTAGHTLNLETPK

NTPQDPKSTAWDTPNSETPKNTPQDPKNTARDPKKPKNTIRDTPNSETPKNTAQDPKNRARDTPNSETPK

NTARDPQNAPKEPKKHKFSAVGPARVGE

>Gallus gallus

MQRRHPRGLGAVGVTGWGSAPRQPLASDTEDEEPPRCPSAGDTASPRVVPESDPEESDVSPNVRVLRKRL

PPPSAVGCAQLDVGGPKKRRLEPKTPPNPGVEPRNPSVSSEEVVGTDVEGWRGSDPTLGIESDTDDEEQP

KSAVSHPNRHQSALEGNVDPNGAQRTPNPGVGSTGNGLCVLTVDSDTDEEAELLPPIRQPEIQQTAPNVP

QTVGVGVQNPQFGGPRGPNEGFQVVAVGSGTDGEVESSGLGVGSPQGGQWGLLVDSDTDVEESRAHPDVE

RPQKLQLNPNVPTVGMETPTPPPGGPQEGREVLTADSDTDLEDHGADPDGLCSTTHRATQNAPNPPGIEA

DGPQKGRRAPEVGSDPDVGGTPSGEPDPDVGGPKSGRQLPLVDSDTDVEEDGACPGIPIGTPKPLVGSAV

VMETPNPDPKGAQSGCQSLEVDSDTDVEEEELNPDVPHLKNPKTPQSAPQNPAVVMETPNPHLKGSQSGG

QNLEVGSDTDVEGEDVNPDVPDLKNPKRPQITPKDLAVVVGTPNSDLRGSQVGGRSVEVDSDTDVEELNA

DVPHPENRRAPQSALKDPAVVMETPNPDRKGAQSGCQSLEVDSDTDVEAEELNPDVPHLKNPKTPQNAPQ

DPAVVMETPNPHLKGSQVGDRSAEVDSDTDVEGEELNPDVPDLKNPKRPQITPPDPAVVMETPNPHLKGS

QRGGQNLEMDSDTDVEGEDVNPDVPDLKSPKRPQNTPKDPAVVMETPNSDLKGSQVGGQSAEADSDTDVE

AEELNPDVPHLKNPKTPQSAPKDPAVVMETPNPDLKGSQVGDRSVEVDSDTDVEGEDINPDVPDPKDPKR

PQITPQDPAVVVETPNPDVVGLNGDSDTDEDLSGVGAPQTPKATRGVTGSGAVGASAPDVTPDVAVTAPI

PAVPPPMSLWGDSDTDGEEEVAPTPHIRRFRSRIRLRNPNVGSTAMGGGADVGQTDPKCGKLTPKRQSSP

PEQRGDGGVGGTAPKRHNSAPDTDAESPKPNDGTQRRSGETLRMGGDTAAAPAPKSPDSAPNLGRPNGGT

DVTESDTEAEGDSDLFLAPTQSFLPPTQSFLPPPTQDPTPAWDPEEPTQCFYHPEEEEGKEEEPPQSRVA

TRVAAVTPAQEDSGTIRAPSEAVAEGPRRSQRQARSRGGGASREGGGAKAAGGGATADPPPLRRSPRLLA

RSSPAEPKRGRGQDEPRPPPAPRPRRGGHAPSQKKAQEEEPTDITRAQLRPRGSSASSSPKVLFTGVVAS

PGMEAALGALGGSMAASVFDCTHLVTDRVRRTIKFLCAVARGIAIVTPTWLHESSRSGRILPPGPFLVRD

SQQEQHFGFSLSQALSRARRRPLLQGYEIHVTPSVRPEPEHMRDIITCSGGTFLPTMPCTYGPRRLVISC

PEDSGRWGPALGAQLPLLSAELLLTGLLRQQLQLQPFLLTPPGPPQDPPVSPRRLRDRAQRDPPGTRRRG

RLGVK

>Cygnus olor

MQRRRHGGLCTTGGTVQGPEPRHPIASDSEDDETWRCPTAGNTASPQVVPESDPEEPDVCPDIRVLRKRR

PTPALGLRTDLAQSHLDPDVGGPKKRRLCPKTTPAPDVGSKMAISDIRPQNHSPNPKGQVDPDVKHLGGS

TVALGIESDTDDEVHPKSALFHPNRHQAALEASGDPNVEKRGPNPGAEGAQNGRWMLAVDSDTDVEEADV

LPSVGCPKIRRKAHRVPKAPGVEMKPPNPDAGSSKNGFQTLVVDSDTDVEMESSNSAVEDPQRGHQAPGV

DSDTDVEEGGANPVVGLAKTPQMTENTPNSSNIKMKSPHPAIKGPHKEHPPLTVDSDTDVEEDRANPDVV

CLPTPQTTQNAPKPPNIEMETPNPDADGCQKENCMLVVDSDTDVEDNILLDVVSPKPHAMTPRDPGVETR

GQDAGGPKMRRQSPLVDSDTDVDEDPSSAEGRDPKPHQITQNARRNLDAEMQTLSPRPGEPQSGHEMLKV

DSDTDVDEDPSDPEGRDPKPHQITQNAQRNTDVEMETPSLHPGEPQSGHEMLKVDSDTDVDEDPSDPEGR

DPKPHQITQNAQRNTDVEMQTLSPRPGEPQSGHEMLKVDSDTDVEEDGLNPDVGCPETHRMTPKIHSDSD

VAIKTPNPDLEGPQNRGQNLEVDSDTDVEGEDLNPDIPGLKNRKTPQNAQKDPTVVMETPNPDAVGLHRG

SMPPGSDSDTDVEGLEDTETPKTHRTTREVTGMGAKNAAAPDVDPRWHAWTKGGLNVEGTAPNPDVGQHM

DLNVDSDTGVEDNGVVPDVGAVQGGQGTPGHPDVAMAPLDPDVSPPMTPQSGSDTDVEEVAPTPYVRSLR

SKTQPRNPDVVDTAMGSDTNVGLTDPKCQKPAQKRQGLPPKSCSDGGVEGEVPKQHDLAPNTDAEDPNPD

DKVQHNPQTSVMGSDTDVAPAGLAPKSPIPAPNLSLSGDAAWGSRSAQATPHGDMDVEVAESETEDDPDL

FLEPTQSFLPPPVTEADAAPGWDPEEATQLFCHPKLEEEEEEEEEEKEEKKEEEENGKEEEEKEEEEEKE

EEEEKEEEPPQDIPATRVPPAEPVVVTPAQEGAGTGTAPSGDVTEGPRRSQRLARGHGGRAPGGGRASAV

GGASGGGVASQARGGALPRPTPPRRSPRLQACPSPAEPPAKKGRGQEEPRPPPRPRPKRSGHAPSQKQTR

EEELPEVAGLQLRPRGGAGSSSPKVLFTGVVASQDMEVALGSLGGSMATSVFDCTHLVTDRVRRTVKFLC

AVARGIPIVTPKWLHESARSGRVLAPGSFLVRDSQQERHFGFSLSQALSHARRHPLLQGYEVHVTPSVRP

EPEQMRDIVTCSGGTFLPTMPCTYGPRRLVISCGEDSGCWAPALSARLPLASAELLLTGLLRQRLQLQDF

LLAPPEIPPGPSGVPQDPPPKSPQPPPVSPRRLRAPLPTQGRTRRDPPSTRRHPQPRNK

>Serinus canaria

MIDTHNSQWKVQIGVDAWRDQPMGAQGARPAVMAREGALEDPAVPPDVEGPQKRLPCSQN

SPDVIGEEPDPDVGDLKMPKVAQDTSEASNVAAEAPNPDVLWARNGHRTLLVESDTDVEE

EEPNPNVGPQKWLKMTQNVPETPDVGLKTPNPEVLWLKNEHGTLMVESDTDVEEEGPNPD

VGPQKWLKMTQNVPETPDVGLKTPNPEVLWLKNAHGTLLVESDTDVEEEEADPEVEPPKR

LKITQNIPGTPDVAGKTPNPDVSGPKMQHRMELVDSDTDVDEDPDVQPKKRLKVTQNMPG

IPDVEVETPSPVVKTPKIGHWRILMDSDTDVEEEENPDVQPQNQLRMTQNMSETPDVAAK

PLNPDVSGPKRHSQMLLLDSDTDVEDEVNPDVQAPKRLKVTQNMPRIPNVEVATPNPAVE

RPKIGHQTLVEDSDTDVEEDPDVQPLKRLRMTQKFPKTPDVRWGAPNPDVSGPTTRSQML

LVDSDTDVEEDPDVQPLKRLRVTQNVPETPDMSAKPPNPDVSGSGIRCQAFLVDSDTDVE

EEEEVNPDIETLKKLEMTQNVPESPDVVAQTPSPDVGMTKNGCQALLVDSDTDVEKEVNA

DVRPSKRWEIPQNVPQTSDVPSQPPNPAVKGSQRGHGALLVDSDTDVEEEENPDVWSLKR

LKPAPNVSETPDVRPKMPNPDVSGTKIGSGMLLVDSETDVEEDEAGPDVQPFKRRKITQN

VPETPDVLTPTQNLAVGSSEISCGALVVESDPDVEEDEADPDVRPSKKKKITQDVPGTPD

VGRLQRTQNVTKTPDVEGEEEGWDPDVATQLFLPPNSDEGEAEVDPDVGSPEQSKMTQKP

LEIPDVEEEEVSDPNVATQLFLPSPDVGSPKPLKMTQNPPKIPDVEEETPDPDEEEEQES

DPDVATQLFLPSPDVGNPESSKTALKDTRIPDVEEKEESDPDVATQLFLSSPGVGSSEGP

RTTPKDPKIPDVEGLPSLDPDVATQLFLPSHPGVEDGLSPDVLGPKQPRMASNDPRIPVV

KAEIPDPDVEGEEDLDVATQLFLPPDPDVEGEGPSQPGAGAPKPPKSPLNPLKIPDVDME

SPNPDEEESLRGRRLLLGDAGPDVEEVSPNPDVEVSKRPQTASKVPEIPDAEVDTVNPDV

EGPRGPGLNPDVATQLFLPPDPDVERPESPKKALDEPQIPDVEEAAPNPDVEGPPSPAQE

EAEAPPTPQVRRSRRLAGSGGGGASHGPTPTQNGTNQGSKPCPSPKPHPQRGQDPSVAVL

EEAPPSMSIKGNGRGSMEAPPPEEEEPAAGAKRQLRPRAAPGSAQIRVLFTGLVASPALR

VALGTLGGTEATSVHDCSHLVTDGIRRTLKFLCALGRGVPIVTPQWLLESSHSGRLLSPG

PFLPRDPPCERRFGFRLRPALARARERPLLQGYQVHVTPSVQPCPEDMRDLVTCCGGTFL

PQLPREHAPRVLVISCPQDRWLWPPAMAAQLPLLSAELLLSGVLRQCLELAPFLLSPWQP

PENTPEPHQNTPNPTQNQKNPTQNTPNSPNPTQKTLNPSQKRKNPPPKTPKPSPKSKNPP

QKPSNPAQNTPNPPPKPPNPAQTQKNPPKKSQNPPKTKESPPKTPKSHPKYPTSNPFPGP

LSGVPPLPQAPPEPGVDEDPPKIPPKNPKFRDPPKNPKSTQKTP

>Geospiza parvula

MPRVAQNTSEAPDVAAETPNPDVLWARNGHRTLQVDSDTDVEEEGPNPDVWPQKCLKMTQ

NVPETPDVRLKTPNPDVLWLRSGHGSLLVESDTDVEQEEADPEVEAPKRLKIAQNIPETP

DVAGRTPNPDVSGPKMQHRMELVDSDTDVDEDPDVQPQKRPKVTQNIPGIPDVEVETPSP

AVTAPKIAHWRILMDSDTDVEGEEENPDVQPQKQLIMPPNLPETPDVAAEPLNPDVSGPK

LRSQMLLVDSDTDVEDEVNPDVQPPKRLKVPRIPDVNVATPNPAVERPQIGHQTLVEDSD

TDVEDDPDVQPLKRLRRIQHLPKTPDVRWGTPNPDVPGPTVRSQMLLVDSDTDVEGDPDV

QPLKRLRATQNVPETPDVAAKPPNPDVLGPEIRCQAFLVDSDTDVEEEEEGNPDVETLKK

LEMTQNVPESPDVVAQTPSPDVGMTKNGCQALLVDSDTDVEEDEVNPDVRPSERLKIPQN

VPQTPDVPPQPPDPAVKGSQRGQGALLDDSDTDVEEEANPDVGSLKRPKPAPNMPETPDV

RLKTPNPDVSGTKIGCGTLLVDSETDVEEDEMSPDVQPLKRLKMTQNVTETPDVPTPTPN

PAVGGSESRCGALVGESDPDVEEDEADPDVRPSKRQKIAQNVPETPDVGLKPPNPDVGRL

QRTQNVAKTPDVEGEEEGWDPDVATQLFLPPNPDVGEAEADPDVRSPEQLKMTQKPSEIP

DVEEEEESDPNVATQLFLPSPDVGSPKRPKMTQNPPKIPDVEEETPDPDEEEQESDPDVA

TQLFLPSPDVGSPESSKTAPKDLRIPDVEEKEESDPDVATRLFLPSPDAGSPEGPRTAPK

DPKIPDVEGLPSLDPDVATQLFLPPHPGVENGLNPDVLGPKQTEMASNAPKIPIVRVETP

DPDVEGEEDLDVATQLFLPPDPDVEGERPSEPAVGSPKPPKSPLNPPEIPDVAMESPNPD

VGESPRGHRTLLGDVDPNVEEAGPNPDVAASKRPQTTPEVPEIPDVEVDTPNPDVEGPGS

PGLNPDVATQLFLPPAPDVERPESPKRVLDEPQVPDVEEAAPNPDVEGPPSPAQEEAEAP

PTPQVRRSRRLAVSGGGGASRGPTPTQNGRTQGSKPRPSPKPRPSPKPRPQRGQDQSVAV

LEKATPSTSIKGNGRGSIAAPPPEEEEPPEGAKRRLRPWAAPGSAQIRVLFTGLVASPAL

RVALGTLGGTEATSVHDCSHLVTDGIRRTLKFLCALGRGVPIVTPQWLLESSHSGRPLSP

GPFLPRDPAVRRFGFSLRPALARARERPLLQGYQVHVTPSVQPCPEDMRDIVTCCGGTFL

PQLPREHAPRVLVISCPQDRWLWPPAVAARLPLLSAELLLSGVLRQRLELAPFLLSPWQP

PENSPEPPKTTPDSPQNTPNPAQNSPSPTRNQKNLTHKTPNPAQKRKNPPRKTPNPPPKP

QIPPQKPQIPPKTKRILPRNAQIPPKVPQSHPKPIPVPILGAPPLPRAPPGPGVGGDPPK

IPPKTPNSGTPQKTPNPPKKTPNPPQKAPKRPRGHSEGPNPEFGVPDPPGAPQDPEPSGT

PQNPPSGPPKFRDIQKPLKPTPQNPKPTPKKPKTAPGAPWGSQPQFWGPKSPPGAPPDPE

RAQTPPKCREPPKSPKSHPKTHPGSPRGFPPQFWGPRAPPEPPPDPEPPGSPNKVNADSG

IWGLGFLGSKIPNLGR

>Rotaria sp. Silwood1

MTDLDNTQIITADDSLIQNSSTIESEASLKIISGTDSNTYQIGKETIIGRTNPSDLIISAPSLSKQHAKI

TFNNGQYFITDLGSSNKTFHNKVQLQPNVCYALHDGDEIKLGDIICLFEEQQTQTNKNTLSEPIYETYIP

PVNTLVEEDSPWPSDDEIPPLVITETNVDVIPSTQTITDSYDDQEPTQPMKIWNRNSNNTNGLTRTSIKS

SDDEILKQNGLNESSSNKSSSFIPSTLSSILNNDDDDSLVIGATPLPATVPLVIETTQINNDDQIEIISS

SRTEQIIKDDDNNQITIETTTQIEIDKTQAYTLQPSTEEEISSNEQIVNKTQSYEHEHEEIVSDVIETKR

EEVHQVVMDGDKMIEEIITTTTTTTTTTTTISENDDLPMETQVYDLQSTNEETTTILTENENPSAETQMY

DLQPTNEEIFKTSTIEDNNTLLETIQPINEEISKTTTTTVEDNYRPVEIIQSTNEQTTVNLPENENLPME

TQVYDLQPTNDETKETSTFENNNLPMETLAYDLQPTNEETTTVLTGNENLPVETQAYDLQPTNEETTTIL

TENENPSAETQIYDLQLTNEEISKTSTIEDNNTPLETIQPISEEISKTTTTIEDNNPQVENVQPSNEQIN

LDATAPAVCADTQKYNLDEQIDDTSPRPASETCEIVPMSQLVEQLQHVDNTVDDATPKQQIEVTIDQNVG

KEVAEDEQMDTNQTELPSATVNDEPIITPTPAIDEQQASVNKKEDEIIATTKTDAISINETNQTDTKVQP

TTIDQETKLDTSLTTTTIESEQKMNESIPTSASEDIPSSTLDTTDKQQEEIEGDEEHEGEEVGEETEESV

PVTGRNQRGIPRRAARRARGRRGGATRARVVSTRIHGGRRNPVLPPTIDNQNNNTTLEETVNTEAEESPQ

KSSTTDIEQKSSDTQPISPTPADETSSPTQTNMNANIRVSARIKARASSGRNRSFPYTDDYVDLDDLEKQ

TKTPITPPSTTTIGRRSTRGGRGSSTSQKRISLRQQPIDTSESPEEKTKKQKDNTDIYEQMDTGGENEEP

TLSTKTVGRKRKSTTPVASTTTKRTRSTTTPQTAPATNTRRKQPSTTDESSSPDPVTPRRGRRSTKLTTP

TEQSSQEQTSTDDRPVRIALSSHLNFDQSHLETLRKLGFEIMDESCQVDALVVDRIRRTKKFFMCLARGA

HILSPTWIETMIKENRYLPYEKYYLQDTNAETRYGFQLRESVRLAKQHPIFENYKIFCTKDTSPPYDDLK

DIIEAAGGKFIEKINLNKPGKDLVCIVAQVHKNEYEDLCKKGVPIVSEEFALSGISKQKLDFEAFSLFQN

VATTVKPTGSK

>Adineta steineri

MTDLDNTQIISADDSLLQNSSTIDTDASLKIISGADSNTYQIGKETIIGRSNPSDIIISAPSLSKQHAKI

TFNNGQYFITDLSSSNKTFQNKTQLQPNVCYALHNDDEIKFGDIVCSFQEQMQTNKNTISDPTYQTYIPV

TNTLVEEDSPWPLDDDISPSMTPETNIDVIPSTQTISDSYDDQEPTQPMKIWNGNSHNTNRLSRTSLKSS

DDVTSKSNGTIESSSNKSSSFISPSISNNDHDDDSLVIGASTLPATVPLLIESTQINNDDQLEIISSSSQ

TKKVIEDDTNTFTIETTTTDVEIENTQAYELEPTSEIISSDNQIADTQPYELEDEQIVTDVVETKREEIH

KIVINGDAMIDQVTTKTTTTTTTVIENGNPSTETLAYELQPTLEEITTSSTKTTTTVIENDNPPMETLAY

DLPSTNDQIDLDTAAPALCTDTQEYNLDEELDNSSPRPASETLEVVPASELAEQLENIEKLPDDTSSKQQ

VEVTIDPDVIREVATHELMDTNQTEHSSTINSNESETNIVDKQEENETIPIKNTDIMPVDEVDKTNSEIQ

STNMEQETAPDLNKSFTSTSVESKEKIDELSPASTPEEVPLSIPESEENQQEEADEEEETEESSSISTRG

GQRGVPRRAGRRARGRRGGATRARVVSTSLHRGRRNVTVPSTMDDEANNVTLEETVNTETEDSPKKSIKT

PDVEQKPIDTQPTTPTTADVPSLPDQANSNSNVRVSARIKARASSGRNRQFPYTDDYVDLDDLEKQSKTP

SASTSSASTAAPSATTAGRRSTRGGRSSSAPQKRISLRQQPIDTAESPEDETEKQKDNADVYDQMDTGGE

NEEPVLTTKTVGRKRKSTTPVASSPATTKRTRPTTPQTAPITNTRRKQTSNVDLSTPNTTITPKRGRKPT

KVTTPTEQSSEEQTSADDRPVRIALSSHLNFDQNHLATLNKLGFEIMDESCHVDALVVDRIRRTKKFFMC

LARGAHILSPTWIEAMIKENRYIPYDKYYLEDTNAETRYGFQLRESVRLAKQHPIFENYKIFCTKDTSPP

YEDLKDIIEAAGGKFIEKINMNKPGKDLICIVAQIHKNEYEELYKKGVPIVSEEFALSGISKQKLDFETF

SLFQNAATSVKATGSK

>Rotaria sordida

MTDLDNTQIITADDSFVQNSSTIESEALLKIVSGTDPNTYQIGKETIIGRANPSDLIISAPSLSKQHAKI

TFNNGQYFITDLGSSNKTYHNKVQLQPNVCYALRDGDEIKLGDIICLFQEQQQSQTNKNMLSEPIYETYI

PPINTLVEEDSPWPVDDDIPPLVITETNVDVIPSTQTITDSYDDQEPTQPMKIWNGNSNNTNGLTRTSIK

NSDDETLKQNGLNESSSRKSSSFIPSTLSSILNNDNEDDDSLVIGATTLPATVPIVIESTQINNDDQIEI

ISSLRTEKIIEDDNNQITIETTTTTTEIDKTQAYTLKSANEEISSNEQIINETQLYELKNEQIITDVVET

KREEVHQLVMDGDKIIEEIITTTTTTTTTETISENDNLPMETQVYDLQPINEETTTNIKNIPVETQVGDL

QPTNEETSKTSTVEDNNPPVETLAYDLQSTNEETTINIKNISVETQVYNLQPINEETPKTSTVENNNPPV

ETLAYDLQPTNEETTTNIKNIPVETQVYDLQPTNEETTKTSTADDDNLPMETQVYDLEPTNDETTKTSTI

ETKNLPMETLAYDLQPTNEETTTVHTEDENLPSETQAYNLQLTNEETTKTTTIEDNNPPMETIQSSSNEQ

IDLDATAPALCTDTQQYDLDEQIDDSSPRPASEECEVVPMSKLAEQLQNVENIVDDTTPKQHIEVTINQN

LVEKNTEDERMDTNQTEPSSTSINDEPIITTTTDTKPETTIDEQQESVNQKKDEIISTTDTSVVPINETD

KTDTKVQPTIMEQEKTSNTSLTTTPIESIATSTSEDVPSSTLDTTNKQQEIEEDAEDEGEETEEAAPVSG

RNQRGIPRRAARRARGRRGGATRARVVSTRIHGGRRNVTVPPTIDNQNNNTTLEETVNTETDESPQKLND

TQPISPTPAEETSSPNQTNTNSNIRVSARIKARASSGRNRPFPYTDDYVDLDDLEKQSKIPTTPTTTGRR

STRGGRSLSASQKRISLRQQPIDTSESPDEKTKKQKDNTTDIYEQMDTGGENEEPILSTKTVGRKRKSTT

PVVASTTKRTRSTTTPQAVPATNTRRKQSSTTEQSSPPNPITPRRGRRSTKLTTPTEQSSEEQTSIHDRP

VRIALSSHLNFDQNHLDTLRKLGFEIMDESCQVDALVVDRIRRTKKFFMCLARGAHILSPTWIETMIKEN

RYIPYDKYYLEDTNAETRYGFQLRESVRLAKQHPIFENYKIFCTKDTSPPYDDLKDIIEAAGGKFIEKIN

LNKPGKDLVCIVAQVHKNEYEDLCKKGVPIVSEEFALSGISKQKLDFEAFSLFQNVATTVKPTGSK

>Lingula anatina

MKKMDFDQTQVLPLSDEFDEMDDEADGFGQQKVVGYLKVFCQKGFPETSFPVYEGDNVIGRQSDKCNIHI

PIKALSKQHACIEVRGLSHMIYDKGSVNKTRKGSRLLCPEVRYDIGHEDKLTLADVDCVYYIAEELKKAE

ISSHFISMEAKAGDESGSETGSESMFTLKEAGGGDWSRGNLQKPLTLETDEDDNSSDVLPPTQVQEEEKV

LVAESDQSDAEDGKDQMKILQGNNNAVAETPFASKPDNMVNKPLEIQQTLLYEEYVGESELEISKSASQP

GVFGAEFVEESDVDEESDDNRSYVFEAATQAFPDGEDKKDLVSVHKAPSATQLFEEDVKDSICQDLSSSK

RRDIHDEPTMVYDVDNTQTFVEGIQAKAESTRMSSFTSDSSDDRSVSVHRGKGKKLSVSFDEQPTQMYTT

EEGTESDASTLPVSASPVKSPDQVHLARGAPTLKRLYPQDHNKDGANIGKDHMRLEETNHSEETAQSDNS

FSTLAVTSSFEKSPVHSSAAAGKPALNRLVPQSPGEDGISNQETQAFADMAAPTLADSNSPEIYSKMASK

PVDDAEATGETQPYFVGATQEYSACSPTQVVGEGNIEVFPLGKKNLNGSSAMSGNGYTDDADATQPYEVD

DAEALEPTQAYGITLQPKSDEIKDTGTHTSCTNSNGKTSLVSGTPEKNEVCADRNTSDNASLEETVAYED

IEPTQVYGASEATQSYGVEDSHPSESDDSTQPITVEGTQDHGCAEIQGYDLAKGTDKSDEIKRVELEATQ

AYGLQEEKIPESDESDDETLLHPIIPIPINNSLHGEKNKIESVSKPEGVAEKDMQTPLKGESSSNDLDET

KEESDFMSPVIAVQESVRCSAASVEGRSQGRRSKSPLNKLEEDESTSEGSEQPGRRKSTRQKIKKKPFDS

NDVSITQKNSGREKRSSVRNRKEADAGTEICTSQVNVKGKQDKVTLKSVQPNEETEIQGKSRVNPGRKSV

GKSDFEQNANLSSDYSKEHKNESSLQTDVDEDLAQIAVEKGGPKTEYVTQVSKEKASESKVRSSRTRRSG

IQPNKERNSEEANLTDGKKSRVRKSLTFKKQPIVEEEDKTLNSEQNVKQLEKKTGGKKSLTFKDETDGEE

NNSKGNAEDGNTSKGRKNRGKRSLASPIQTENENKNLEDLHSSKSKTKALRSQQNDEVVARSGTNSETEL

PTRTRGAQSRTAVKQKEQKSKVEKADHVEKPQSSTRRSRSLGTNRNTDEQDSDRTDNASEVSDELSSSVR

RSSRGHSSKEKQENDSAERPKKAADIKQKESESKLTSISIAAPETSTRTGGRNKRQRQSCVASINVSEEN

KDEQLKGPSPNKRDTHITENQTSKTVRKSRTKQKSAGRDNMTIEPAVNDETPAATRSARSRQASAKVEQK

KEGFVKPEEIPVQTTRGQRASARNKSEESILIEPERVGRKKRDLSSDSQSSNDSRDTGSKRNKRTSLGTP

AREHDTSQTIESPSLRHRTFTKPKIMFTGVLDEDGSKTIKNLGGELVHSIQDCTHLVTDKVRRTLKFLCA

LGRGIPIVSIEWIDRCKDAHTFIDAHPFLVKDGAAEQKFKFNLRDSIQKAAMRPLLTGCKIHATKSTAPP

PADIKEIVKCAGGQYLATMPKKVGDNIFVIVGENEETKNFKAVSDMGIPCVSPEFLLTGLLQQKIDVDSF

AIKPEISDGGKNRKRNITGTASSATKKKRN

>Patiria miniata

MDFDGFDQTQAIALDDYSDDTDDLGDLDNKPPVGYLKVFSQKSFLETSFPVFEGDNFIGRHESNQICIPL

KALSKKHACIEIQGDSHLIYDLESRNKTRRGKLFLKPSVRYELQDPDSLLFGDIRCEYTRAEIQKKPTEL

AEDSDDDESDTGSESMLLDSQNAKDKAQPVKGPDLGSSSSYDILQPTQAHQDRESRHTIILETPQREGAT

LVFAESSDDNAPAEAAGRSKLSELDVSALAMAPTQPYGGMDELETQAYPANTDSDDDEIRQSLFDAPTQA

FAVPESEDEMESHKGTRKKNKRTSVEPTMILNLSTDDETSPVKRPARPKPYPQQEAAGAPEKHPQGYTDE

DGETSTLAYTDLATQAYCADTDDQSDDDADKSSKERPQARDTSENDDEPTLAFPDLATQAFCTETDEQSD

EDDEFGIRSKRRAVQTLDPTVKYDMQEESPDAGPDQGETDSDADVQKRRVVEPTLKYDMDVEDDDKRSKV

KVDIGLEPTIAYTTEEDDEEDMDVSALAIAEAQAYTTTDSDLDEEQPKPNTGRRRQQTAREQATVFSDAP

TLAYDDIKEDLASGQPKAGLGDEGSSDAGYGPCDEATLAYNDDDEDDATDTGMPLDEGEERMMGYAEMET

QPAGKESDIAGQPDVPTLQYNTTEDEDDTTAATQAYGVDKDEDETPDIHSHPDAPTLQYNTTEDEDDTTA

ATQAYGTDDTDDDTLDIITNPSTSLGHVTQPANQNGGQKETSAGPSIAGTKPGGNRGDGLEETQVPDSQD

GAVDEMQPLVGMSRISSMLRKVPVRSAMASPEKKHPQRTRRVAFKEQTPPEEAASEEVNPPSRKSARGRK

APARFREETPTSSVTETDAKPTRRKSKEFRSAPGEEEASDVAKPKKAAKGKQKGSKDVLEGSKDVLATQG

KLADGDSEEDTLELQEGSDMVWTSVGGFQLIEANNNETEPKGKAPARRTSRRQASYKKAVEGHDSDDTES

MSSTSSLQVEVGDLSGSAVQVQVTVCDGKQSEDASKPRRGRRSNTGKAAGTSSGRAEKLAKEVDEPSPES

SRQPATTSGRRQSRRSAVIKSDVAETLPKPITKVGRGQHNQQSTDVPKQDQKSKEDIANLTQPSRDHKNV

TGSETKEITAPARRGRKPKSAATPQTKGPKQSSITDFTVAVAEDKVGSRSHPEESSSISENKTDSLESND

SPSLLPSNLKPAKTSAPSSHKKQTTPQTKSKSPDTASQKQDAFVAPEPVKRGGRPRKGAKQDEGQRSTES

PPVTSQPQTATSDKAETATQGRSKKKGRNSGTMETPQKEEQPAATPTSNRRGKRAGPAQTTPSPTPTKKR

KDPETPESEVSSPSLRKRPSETKPKVMFTGVVNKTWEKIVTSLGGELVESVFDCTHLVTDKVRRTVKFLC

CLSRGALIIAPKWLDQCQLHKTFVDPSPYLLQDKAAEKQYSFSHVTSHQRALQGGALGALSVFVTANVKP

EPAQMKEIIQSAGGKCLSIMPKKPDPSTIIVSCDADEARCQPAIKAGLQVVSAEFILTGMLRQETRPELY

QLFTPSSTSSEPSSKRKVGDTPSSSKRRR

>Lytechinus variegatus

MDATQAIIFSDEEDSEQEQGSGEKNAVAFLHVLAQKELSAVKHPIYEGDNYIGRHDSNSIHMPFKALSKQ

HACIEVQGNSHLIYDMESRNKTRRGKMFLKPNVRYELRDDDVITFGDVKCQYILQSEQEEDDDDTGSDTD

SELMLQPPDSEDLDQDDYKRKEDENDNESSFSINDFIQPTQPCIQNGAIDVIATQAVDNMETQAVDNMAT

LAVDSPETQAIDDMETQAVDNMATLAVDSPVTQAIDGMETQAVDNMATLAVDSPEPQDIDGMETQAVDNM

ATLAVDNSETQAVDGMETQAVDNMATLAVDNLETQSVDGMETQAIDNMATLAVDCQATQAVDGVETQAVD

GMETQAVDNMATLAVDNLETQAIDYGIHSDSSDTDIDDDVRQSILEAPTQAVALNDSRQQGTPIKECTRT

LVLDSSADSSDIDDSPVKQARNVYDDPTQICDSVVSDSGSETDIEDPSMIPETQADVTGASEVDEMATIA

IDQDDEILKDRREDKTDSSEGDRLNAPQPPSSDDNTELDSDMGNTSQEYFLNYRTNEAPGDETPSNPADI

EVPSRAADIEATQPYQGHSDDDNTDEMPSKPSDIEPTQPYQGHPDDDNTDEMPSKPSDIEPTQPYQGHPD

GDSADETPSRPDDIEATQPYQGLLDDEGTEEPTPTNQDKVNVTNLEATQRYGDINKDKTTEEPTPALSVT

MEMPSSATMVFPSTQADDAKEKQPDSCDDDETDPTVPYHTGDDVAMETGDSEIESTQAYGMQGGNTQVPD

SQDGDDEPTSSRFPVFKTQSPSKSCLKQPNTPDRKATKARRVMFQSTEDEESSQETDGDTEPLKRSVRSR

GQAKGSKSLGVDEEQSTSKGSRRSSSRVSQRKEDEEVKEAQNVVSGRKGRRSKTVEDAAMKPGQKRKADA

LEQPSASSAMVNKVESNTKREKKQGRHKIEDEIKEDTVDGALTVTDAKPTRKKDFDEENPRPSSGKESKV

KDKRKLSDSSISSVGSEGSKAGRALRTIVQIGKKLTRGSKRGKKSVGGGESTNDSLPVVTKTLQSTAEVI

DDGDSDDTASISSTASSIRDFEEAKPSTIPTRGTRSRRSKVADEPDSQSSSGIRRSQRGGTEKKEMTPPE

ESKKKVGGRAKKGVAPETVVTEVDGPSTQSSSRTRRSERSTQGDELTSQDDSQKGVSTRGKKSEKNEEEV

KSRRTRRGAMQDAKPDTQETNPKGNENIKAVELKSEMGKTSKRGKTKAQSSAEGEQNVSADASIREDKES

QEAGSKRKRNATSESQEGSTKDTTEVNVRQSGRRRTLPARFVDSEVKGKDGEKKAASEHEVEDKTSADAG

VFAVPKAVGRPSRKTQKKEPSPSSQGEVESEMVQTSSRGRRSTAKVKEEVAEKEKSEQPVKENTRGSRKR

GQEVLATKPAADSKGEETMATPKQRRGLAAAVNTSEGPSKVEMSEDLKEDAKESRRRGRQKPTEDAAASE

KILVTPSSRRGQTSVAASPAKTSKDAERDEQSKEAKSTEAAKMRGKRGATSLTPSPTSSPSSAAKRSRQT

AVSSPSISPARRSTQRRVGENKPKVMFTGVMDDSWQKAVTTLGGELGNSVDDCTHLVTDKVRRTVKFLCS

MARGIVIVKPTWLDASMKAKAFIDPSPYQLKDKAAERQYSFSLNVSLDKAKETKLFDGYKLHVTPGVKPD

PQQMKDIIRCAGGEYVAKLPTKHIPQLVIVSCDGDKSLWAGPRKAGIPVVSSEFILTGILRQAILLDDYQ

LK

>Acanthaster planci

MMEFDGFDQTQAIPLDDYSDDTDDLGDPDDKQPAGYLKVFSQKSFNETSFPVYEGDNFIGRHESNEICIP

LKAMSKKHACIEIQGDSHLIYDLESRNKTRRGKLFLKPSVRYELQDQDPLVFGDIRCQYTREEIQQRPRG

RDLVPDSDDDGSETGSESVLLDAQNGKDTAQPVKDTDFGSSSSYDIVQPTQAPPETEKKQTVILETPQRE

LATLVFAESSDENIPTETVKAVADSSISPGSVAETPASKPLAESTVVNESDIEESTTGRSRHSALDVNAL

AVAPTQPYGGTDEQETQAYLATTESDEDDADEIRRQGLFDAPTQAFGVPDSEDEMEPAKGKTKNKKISLE

PTLIFNLSTDDETSPAKRPGRPKHYPQHKDAIEAKKSPQGHSDEDGETSTLAYTDLATQAYCPDTDDQSD

KEADESVGGKTAVAKGRPRGNMSPDDMEASTLAFPDLATQAYCADTDDQSDEDDEFDLRKRRAMNTLDPT

LKYDMESESPGTGPDQAKTDSDINDQRTKEAEPTLRYDMGVAVDKSGPEGNVDICLEPTLAYTTEEDNEE

DMDISALVAAETQAYTTTDSDLDEEQAKPKTYRRRPQTDRKQHSKIVSAEAPTLAYDDNSDSVASRQARE

TPDDDEAVDSHCEEATLAYDEEEPMDTEMSPDAGEERINRFAEMETQLADGMKQSDKADTPVLQCSTTED

DDDTTVTTQAYGTGKDEDETPDIHSEPDAPTLQYDTTTEDEDDTTATTLAYGIDKDNKTPGTRAERDAPT

LQYNTTEDGDDTAAASRNYGLDKEDGQMQEIHGKPSTSTTDRPMLPADQDKAIVMAKRKLHGSSARGKRQ

DNEEVRQEETDGTSMETKVIGAEAGSETQVPDSQDEREEENQPLAGISRVSSMLRKVPARSAMASPEKRL

PQGGRRVAFKEQTPPEEATPEEKIPPLRKSARGRTVPARFKDDTPMTTRVTETDAKPTRRKSKEFRSAPG

VEQASDEVRPKRAVKVKQKESRDVVEGTGNVTGTPSKPADGDSEEDTLELKEGGGMVWSSVGGLEVPEAN

NNENCLKGKAPARRTSRRQASYKKAIEGHDSDDTESLSSTSSLPVETAELNISTVRIQVTACDGKQSDDS

VKPRRGRRSNASKTAGMASGKMDKSAQQVSEAPAEEGRLPATPAGKRQPRRSAASKGNLAETSEKTQTKS

DRDQQASNASETVEKSRDVTSSSAEGNRSSRNQTKEAAIESDKIAVPAKRGRKPKSVVAPQTKGLKQSSI

TDFSIAVTEDQGRLKSQPEKSSSKMENMLDSLESNESPSLLSSESLSSRTTARSSRAKPSTPETGGKSPS

KLAWKQDAFVAPEPVKRAARGRRGAKQEEGQRSTEPPPATSEPQTVTPDKVAVASQGRGSKKGRNSTVSE

TPQKEEQPAATPSSNRRGKRAAPAQTTPSPTPHKKRKDPYTPEHEVTSPSLRKRSSETKPKVMFTGVVNK

AWEKIVTSLGGELVESVFDCTHLVTDKVRRTVKFLCCLSRGSLIIDPKWLDQCQLHKAFIDPSPYLLQDK

AAEKQYSFSHATSHQRALQGGVLTQLSLFVTPNVKPEPAQMKEIIRSAGGNFLSSMPRKADPSTLIVSCD

ADEARCQAAIRAGLQVVSAEFILTGMLRQEARPELYQLFTSSSSNQPGSKGKVRDTPSSGKRRR

>Strongylocentrotus purpuratus

MDATQAIDFSDEESEEEHEHEPGQKKAVAFLHILAQKDVSASKHPIYEGDNFIGRHDSNSIHMPYKALSK

QHACIEVQGNSHLIYDLDSRNKTRRGKMFLKPNVRYELRESDVIIFGDVKCQYIIQSEEDDDETGSETDS

ELMLQPPDSEDMDDKNSNEDENENETSFNLNDFLHPTQPCMPSPKLVFKTPRRLNEGKVFAADSDEESPN

NSNAGRFGDDSFIPETQLSAKKKNDISAVEESDSDTDIDETGPFDILLASAQTQAVMATQAVDNMATLAV

DNMETQAIDNMETQAIDNMATLAVDNMETQAVDNMATQAVDGMPTLAVDGMETQAVDNMETQAIDNMTTL

AVDNMETQAVDNMATLAVDNMATLAVEDGETQAIDNIETQAIDNTATIAVDSMATQAVDYSPDSDSSDTD

LDEVGQSMLEAPTQAVGLDGSRRQGTPREWSRTLVLDSSTASSDGDDSPVKQARSSRSIYDEPTQACDGI

VSDSGSETDIEDRGTEAETQAVLGDSENEIDNMATVAIGQEEEEEDTTEDKETMETNSKIPITDTTWDVE

NMATVPIEMDDYDDDTDVEDSDDKGANSDTVKIKDMADMATIAIATANDDDDDDTDVEEGSKDEEEKSVI

NDTKLKDGNRDIIKEDKPIIAPSNSDHDDDNTEMEKNNSSQEYFLNYRTEQNPAAEEPPGSCEVEATQPY

GGPSDDTSTEDVLSSNHGNDDGIDATQPYGGMNEEETMEEPTSATSITMALSFTQPYGTEGENKQSDSHD

DDTEPIIPRETEEDIAMETGDSEIESTQAYGMQNSDTQVPDSQDGDDQPTTSSRFPDFKTQSPSKSCLKP

PNTPERKATKARRVTFEPADNEEEGQESEGESQPLRRGSRSRGNQSRGEGQSSSSGSRVSRRNKDEKVDI

ETKDDVTSGRRSTRFEAVSDEAAAKPGRKRKVGALEPVSADSSAPATKDKSDAKMKRGEKQRGSKMEEDP

NGDPVSVDEGLTMTDAKPTRKKEFDAENPRPSVRQGSKVKDERKLSDGSISSVGSEGSRAGRALKTIVQI

GKKLTRMSKREKKSSEDSEPNATCNPVSDSVATRRPQRTAEVVDDGDSDDTASISSTASSVRDFAEVEPS

TRPTRGTRNRKGSKKITEEHAGHVTPGARRSQRGGSEKEETTDLQESEKKAGSRTRRGGVAIETVETEVD

GQSTQSRSRARKRELAIKGDETPSQDDDQGEVSTRRRKNERIHSQNDEEVSSKRTRRGTTQGVKPDVQEE

EPIGNISTKRGEIPSKVVKPSTRGRAITQHAGGKEQGTQGETSVQADKESQETSTRRNRRATSESPEGSA

NETGETNVRHSGRRRTVPARFLNPDGKDKATGSHRSSLEQEAEDTSSSNAGVFAVPKALAKPSRKIQQKE

LPSSSQTAVEAETVQLSSRGRRSTAKIPEAAVEPVKEASRVSRKRGHEAPAAMSGTLCVEESKAEENKTT

FNQRRGRATTRTSAAPSDDGKSEDLKEASRDSSRRGRVKPVEEPVSEKSSVTPTTRGSRAAVPAPTAKTS

KEFKERGDRSTEGDKCNEVVKTRAKRGATATPPPCSPAKRPRQATIPTSQSSPSSSPATRSGHQAYQTST

LSPATRSGHQTDQTSALSPSLRRRLSDVRPKVMFTGVMDGSWQKTVTTLGGELVDSVHECTHLITDKVRR

TVKFLCCMARGIIIITPNWLEDSKTAKMFIDPGPFQLKDKASERQHGFNLQTSLQKASQARLLTGYKIHV

TPGVKPEPQQMKDIITCAGAQYVAKLPIKSSQQTVVVSCDGDKSLWAGLSKAGNLLVSSEFILTGILRQD

VLLKDYKLK

>Anneissia japonica

MDFDLTQAIPLSDVESDVTDDLDESLSKKPIGFIKTIKQKGYPETTFPIYEGVNLIGRGDAANVLIPLKA

LSKEHACIEADGESIFIYDKGSRNKTRRGKLFLKPNVRYELKDSDKLIFGDVKCEFLINHGQEQAKEDDM

GIESNSGSETGSECMLQINVVLPSGSSSAVIDTKSKTANNNACIVSPDQKQPNKRTACETFVEESFLEDK

EKKEIFAEDSGSDTDIEDGLQQTKQTDTKADKDLTNVDENETQIHDGSVNQHSTPASKDQEKDTKIEANI

FADEPTQACTMDDISTLKYNTASDTDDEEVDKAALTTAPTQPYTAAEAATQRYGDGDETDEDVDAGVANA

PTLAYTVAEAATQMYGEKSDSEDGNDASEALTAAPTLAYDNIDENTIKDQSCEDAKDGNSDGDKTDDEVD

LAGLATAPTQAYTVAEAATQRYSDGDETDEEVDLAGLATAPTQAYTVAEAATQRYSDGDETDEEVDLAGL

ATAPTQAYTVAEAATQRYDDGVETDEDVDAEIAAAPTQAYTAAEAATQIYGEESDSEDAASGGLAAAPTL

AYDNIDEKEAEESSEDVAAAPTLAYEAQTQNAETLDVQGSAVESQVQGKLEEKGSCSPQPLDDAPTQVFG

VDDENAPIQVFGVDDETAATQAFGEDDETAATQAFGEDDETAATQAFGEDDETAATQAFGEDDETAATQA

FGEDDETATTQAFGEDDETAATQAFGEDDETAASHVFGDDEQGPGPARNVVDSVKEQKPSAQLQPDELIE

TQSYGVENEPMDTADSNTLEIDVTGDTQVPDSQETDELEIESLLRKVPGKSVLVSPEKKSKNQEKKKVVF

SSVPEFEPQKKSHDLKEGKKNTKGKKQKGIKDKKEGDDKEVLDASRRSGRSRKQTTRMKDSVDQKKRRSM

SSNAEIAFSISKDENNVGIGEQPIKDVCGEKSPSQSGENKVLAVEEQSNGQSMTARKRRQVEEGMKEKES

DVINTTQNTVTQTPRGRSNRTKNMKSSLKEDKVNKVNGEEQMEEKQTRGRRSKINILEEEKEECTKPVED

AFEIEKTIKNTVPQIPRGRSNKTRNMKNSLKEDMVYKTGGEEQLEEKQTRGRRSKRNIVEEEKEESTETV

KDAFETDNTLRSTVAETPRGRRHKANNMKTSVTEDRVVKTDRDEQMEEKQTRGRGSKRKVVEEGKQESTQ

SLKDAFETDNTLQSTVTETPRGKSNKTKNIRNSRMKEDMVDTTDRCKQVEEKQTRGRGSKRKLVEEEKEK

TTEPRSRVSKRGKAENSEEIDSQKITKNKSSTICQSPTPSKKAKKAQDEDLPCSPSLRKRGNDSKPKIMF

TGVVDEGWVKIVKSLGGELVTSIHDCTHLITDKIRRTVKFLCGLAKGAYLIDPKWLEESKKRKSFVNPDN

FLVTDRASEKQHGFSLKRSHDMAIETRLFEDYKIHVTASVKPDTNQMKDIIHCAGGEFLSKMPNKYEEKI

VVVSSEDDETRCNSALKAGIPIVSSEFILTGILRQEIHIEPYRLFVEVPSSASKSKPSASAKKGTTSKRR

R

>Asterias rubens

MEFDGFDQTQAIALDDYSDDTDDLGDPENKQPVGQLKVFSQKSFQETLFPVFEGDNFIGRHESNQICIPL

KALSKRHACIEIQGDSHLIYDMESRNKTRRGKLFLKPSVRYELHHDDSLIFGDVTCQFIQVEKKYEAESD

DGSDTDSELMFQDLPKVGEKTSKGKDADFGSSSSYDILQPTQAYGTSETERKIENDHLNKEKKTILLETP

QRDPATLIFAEASDENIPTEPDKSGADNSMCHRSIEETPGTKPLAESTVINDTFDEDERNEQSDLDISTL

ALAPTQAYGHFAELETQAYPTVTDSDAEEEEVRQSLFDAPTQAFTVPDSEEEDTPRRRKRISLEPTLLLN

LSSDDDKNKPYQRSLSSDHHGIEEEATMAYDETTSESDEHPQGPNNEEAETLAFADLATQAFCPEDSDEA

VDVPGSKSVPVLEPTLKYDMQSASLEDCKPKQAEAETVPDIPKTESDTDDDKREQGVEPTLKYDMGIEGS

KEEERGDLATEPTLAYTTEEDDDEVMDVSTLAGAETQAYVTTDSDSDHDCPEMKNSRKPVHDQANTKPPV

ANIAEEPTLAYEGMDEAPKMSYSEEATLAYSNDDDEPMDTDMSLADGEARGTVGFAEMETQQADGVESEA

AATADPNAPTLQYSAEDEDDDATMATQAYGADTSDGDKATVEKPSTSLAKQHSAVKEGETERGGEGLNKE

EVELGEIDAHEDDDTTMATQAYGADTSDDDDDEANPETEGEKPSSSLAKEQSGVVENGIAGRQEGLNKEE

IKPGENDADDDFTLATQVYGAIEESETQVPDSQEGIEEEEGPLVGLTHRVSSMLRKVPERSAMASPEKKQ

TQRVRRVAFMEQTQSDEEDSPQEETKATRKSARGRKPKVKEPQRTRFTETDAKPTRRKSKDLTLPSEASR

KRKESRDVVEGEKKSVDGDSEESAVEVKEDQGPVWKSTSGLEMKEVNNNILDKPAARRTSRRQASYKKAV

HDEDSDDTESMSSTSSLQVAVGEPSGRSLEALGITEVTVCDGKLSEEVVKPRRGRRSKASKEAASKEAAS

KEAASKASNVPKRKYSSSVITDPETDTPAVNRQAKVPKLKISLGRQESESDKIPKSKQSVDEDSNSSSKG

TRSTRGRKAADVSQESNEVKETPVSARRGRKPKAAAKTLQESTKTPKGKGQKQSSITDYVVAEDTSRLTN

TLSSDPSIEKVQKKQTKETKERCSLPATNSEPSCSRRARTSSAETVTKPSTKPSAEQETFLAPEPVKKGG

RKRRGTKDEDQGSDASQSDASPLGTNKKEDGTQGKGGKRGRKAAVLETPKKEEQSLTTPNNRRGKRASTP

TLSTSTGKKVSASETPEGEVASPSLRRRPSDLKPKVMFTGVIDKSWQKIVSSLGGELVESVFDCTHLVTD

KVRRTVKFLCCLSRGALIVVPDWLDKCKVSKTFVDPAPYLVKDKAAEKQHGFTHVTSQQRALQGGVMTKY

RVFVTGSVKPEPQQMKEIILCAGGKFLSSLPVKPDPYTIIISCEEDKAKCQSAIKAGLQIVSAEFILTGM

LRQEIQPELYQLFTSTPSTSERGTKRKVSETPSSSKRKR

>Amphimedon queenslandica

MATNSTGQIRIFHPSPSLSEENSFTDYELFEGETVIGIGCKKRSKCPVINIPFSGSSVAKEHCRVLIDDN

EHFIMDLGTKGKTKRKCHILKPNVYYELSDGVDLMLGDLKCQYFTSPMSAREKEEEKKPVEETDLNETRS

LNTDATATPSPRPAHSLQMSPLPTLPSFDADNADITGCGSPQLLADPPALTASEQEPTLAYQLSSLPQED

EESHKGTDTTDENDQTLPFDSPVPEDRPITNGVTMDANDDKADDKVEAGGDIVNASDEGEDDLDTTVIIA

EDTTDTEPYNLEEEQEKEGGGGKEDKTKASVDPSLNTKSGPIASLFNEDDDEAPPLYEPTIAYNLEEDEK

RELSSVHSEETGTREKSEEEEKDKEDEEEKKEREEEEGKKEEVKEKEKTEEEEGATTRGRKGKRGRGGPP

ASTSRGGRKRKSPKDESPESEDKEKVKGAGSSLIDDNAVKDEITKQTEASCGDISTNTGSSSTTKGNTKK

RRKTPSPSPSLPDDDRLVTTSRRTRSRRDPPKSDKYSFDELYSVPEEPIASEELPVIPTVSSTDDSHDTV

VKKGKGKRGQPKRSKKTEQNPLSLESEEPLVAIPEEPPTATSEEPPTTTLGEPPTATSEEPPTVTSEEPP

PASVSSIASSEDPVKPKSTRSKRQPRATKKSKKMATEEQLSDQESLDKEPLPDKSGEHPMSASEDPPPSH

EEPPSSKSKRPRRQPAASKKKKVTESLSEIESEDPLATDDDIDTKKTLPKSKKLKGSVLELEKLKPSSNG

KKKETASLDECPPIPSLSHTARSTSTRSTPRRTLKKGATDLPLPPSVLFTGVMDEEAVGIVTELGGSLVD

SAADCTHCVTDKVRRTVKFLCCLAKGCHIVSTKWLKDCHREGRFIPVDPYIIKDSATEKQYKFSLKNSIA

AARKNSLLSGWRVFLTENIKPSPSDMTFIINCAGGEVIKKCPTESEDDTFIISCDMDKKSLSSVSSLGIP

VCTSEVILTGVLQQKLELDKYQLNTGSLSSVSGSATPKKASKRRRQ

>Dermacentor silvarum

MDGNDLEMTQVIHGQDDSEDEAEREIRAILKLHDQDAPAAFQSFHLKPGTNVVGRSRTCDVIIENYAVSK

QHAIIDVGSDSCTIMDLGSQNKVKIGKRTLKPNCQYNLDYNEEFCIAGLRARILYDQNNGAKSDTGSDTC

SESLLTAIEVSKDQEGVDAEACDNADKPEPSAAAAGLDEETGAAVESKLEVSNKTASKSSQNAKTCNSEY

QSSGSFTMPEMPMLDYTQTDSSQGTEPYAGADRDAQTNQEGAGAGAPAAQVPVGANAPAAKDTDAFLCAP

TQPYSDATCGGNAGDTFKPATADQESRLNAETQPYTDNAERPEVPSSNPGEIIDLEMDETDRLNAPTQAY

ENDPSAEAERLNAPTQAYTEQDEDERLNAVTQPYAPDINQEEEERLNAVTQPYAGDVNQEEEERLNAVTQ

PYATDVNQEEERLNAATQPYAADVNQEEQDRLNAVTQSFNDNAEEDNMVEESVCAPTQPEERHRRLLCGP

SELSFVLCEASQPCRDDGGDDDEEEEEFCAPTQKDESPPLKVHALLKYRRERGTVAAEDDNDKTPPLSPK

TTIDETPPPSPGFVPESDPEDDDGDTSMVTARRSSSIFNMTGASVYEDCVTPGGAIGGSPIIGSIGKVSG

KRPRRGMSLKKPTCDTVLELPSQESTAGAEWMQQQQAASHSKASKKLTYIEEADDSRDLSAKTSDASLAE

LSVSGDEHTSKNSSIITTCSPQKVPAEPQESADTNKNRVTTEDGEMPVLHMSEVTDVDTTATDKEDSTIQ

DSAVKEGNRLLKPAQARKCQQTLASLLQPASESEEESSRVSRRKGRKEPPSKTTRTRRKVASPSDEKEAS

IEKTAAPARRSSGRQNAGSRMKSLLSLEKRTSASGDFEESTLSQEANDKRDAPKDDAATEEPQGKAGRSR

TRRGLKGKAVAEVPPLPPNDDLASANQPKVVSSSSEDKGENEEPSVKPSENDSEGEAENGEGTHDNKDEE

CPSNRGLLVDQEGGNAEDKGKVGRSRTRRGLKGKAVAEVPPLPPKDDLASASQPKVVSNSSEDKGENEEP

SVKPSGIDSEGEAENGEGTHDNKDEECPPNGGLLVDQEGGNAEDVMSTASLTPSLVDGDNNCTSGTIDTC

FSEPFPTFSEVMAECQAYIEKTEGQHEEKEAAEAQEETVADLADESKAKAIAPATSEPEHVAEEQPRASR

SKDRKEGVDHEKAKRTRSGGGVAYNTAQGHSKRCTRRAAYRNWKTVPRVAVTNIVEDTEQSEGEPDADRL

SAQETESIDGTPSDTSESLTPRGKAVPRPRSSKAPRSKDPKEGVASRKRKGPEAAEELLSTRRKTTRRGA

QERPPTGTELLAEDSPGDIEQPEALQSPQQSNKPRHGRPPRGTVGRKTAPKVVDTKIVEDTEQSEGEPDA

DRLSAQETESIEGTPLETSSSLTPSQETAVPHPRYGARGTRQLLQQDAKRVDEAASKDESGTDHLSAQDT

ESINGTPSETPEKLTASEGNVVPSLSKASRRSAKTAKQSLQQDVEEEDKTALDGPVAGNSEEQLQGITTC

KLGELGVAEMLPYRPKMSKSCRLQKLQKTPRRTVAQRPNLALQQLVPLEVSVKTEPPSPGTSRRSSTKTA

QVQLEETEHLDNNDSAEVSSKPKRRVTELKQEHISEAEASSSGSTTRRQPFKMPALAPVTRKRDARRTGK

KEDPPPTSVMPVLKIMEETSPQCSLRRIDRKRHVASMSFGVPRHESDDEASSNEEETLEEVTVKLGPKSR

QKAPVKEVAVKQEVVEEAPVKRGKRKADAPEVKPEVPRKARSRRDEQADSSLAPKKAVKVKPKVLFTGID

STSTEEVVRDLGGTIATNVSTCTHLVTDKFRRTVKALCCIGKGTPIVDVAWIKKCQEAGAFVDHMPHMLL

DKKAEKTLRFSLRDTLAKASAGGVLRGWSVHATPRVLPSPSDMKEIVMCAGGKYLDNLPARSSTSTTVVV

SCKEDLKACARARNNGVPVVAAEFVLSGLLQHKLDIEAHRLE

>Ixodes scapularis

MGKATLVPNVQYKLEYEEDISLGGLKAQVLKHLESAATEDNQGSDTCSESLLTAIQIPQDQQLGGIGGTP

GDKGTAADSSGSQTSTVPCSNSNDGSQTGLSKARREIGEDGAAGVAAVASTSGPQTDATSEAQSGDDFRL

PLMPHIDFTQTESSERADSERSESFQEEGDQGMSSASGCGNKGEDSDAAVTKEPEAFACTPARLCADADG

AVGEAGNGDLDGRSAVLQNVPTQDVVVADDVCFDDGCDAFRNAPTQAYASTEVSAKENEDGASSHKKEDA

FRDAPTQAYAQMESAVEDDNDDEKDDAFRNAPTQAYAQTESAVEDDDDDDDGKDDAFRNASTQAYVQTES

AVEDDDDDGGKYDGFRNAPTQAYAQTESAVEDDRKDDAFRSAPTQAYAQMESAVEGDEDDRKDDAIRNAP

TQAYVQTESAVEDDEDDGKDDDFRNAPTQAYAQTESAVKGDEDDGRDDAFRNAPTQAYVQSESAVEDSDD

NGEDDVSHNAPIQTFPGAQDVVEESDAEDGDRFDEKDYAFRNAETQGYARTEDLVIERDAKDGARSDGKD

DSSLNAATRAYKEGTSHESADDEPCSPDGETRIRVRDSETNGAMPIFTKNDEGKDAEEPSTAVAPRLGPS

TNLSRLLFADTEPACDSFVLCAASQSDGDQTDDEEDFCAPTQMDAGSATPKVHPVDLKRSTPVSVNETPP

LSPKSAIEETPPSSPSIVPESDPEDADDSMVTARHSLSLLESTGTSLFQDCEEYGDSEVILPVVGRSDKR

RALLSSLKRSGGKLVKEHLRTTHREASQLKALEEEPDSVDGNLVGEQRSKQSGAAKKLEYAGSTSSALAI

PEEESTPGDVSRCKSASEGAPFSIPGVAPLQLSSLVSLNEETAESNDVVLDSTFTVGGSNATADSSVHSA

VAEDHRETAEGAVEEAGFQEADNRLRESSGRTELPNQSVDQTREKHESVLDEEETQVSDVEDVEDTKGVS

SGVEAEPAEPLRLSEEADVNGSTLSEVAVKDAESEQEPKVPSLEATEDVLESNLEHENQTEATPLPFKLP

KSPGRTKNADSEAADKVSKLTKKGSERKFLSKKTETTKEVTLNLDVVQPPTRSRRSNAGARMKEFLSIEK

RTGASGNFREDLASQEANKKEAPPASKEQSNVEGRTSKPKKRGRKSTKEQMDESNEGQKLPSLPQPTEEL

QLQPLVSAAPESFSAANPAVEGKKTYQPFSRGIWDSQFQSTPYTSGTIDSCLSQAFPTFSQLMVGAGDDC

RAADEVDEAAATDMDERKKPPQQKAIASNVSMVVADLDPKEVHPKTIGSTLGFSGKMSEASSRSDTGPDR

SKNSLDAAVVQVMDAKAGHSMQRTMSQDLKREGPQDLAVSEPLLEDAGCVPDSDETQDLDALIPAESTRP

KWATRKSTPFVPSGLANKPNRKIKLESAEDTGGSGDEFQDDSQTKKVSGAKQVVAASRPSRNAVKPAPLA

TTSTVQDKQPCTPTKEALPARRRTRKQVLDVLASESNDLSAVREAGEKELGDAIDDKKKSSRRLQSKRAA

SGDVEGPQSSLASHEVPQGKRRKNKAVSEVPDVTIPEPNVAGSATKEVEDQKGTIRGKEGRLSSSLPGGI

VLQVKEAASSESRDSQPTRSCQGRARARKVVDTPVSDVVKNNSEGFNEASAVDKDKREPRSARLKGRTVP

EAASRGPQGSQSSPSSQGQKRTQKAANAFFLDVADAGREESDEASAVDEKRDPCSSLSKETTAPPVEQAP

SGEFRDSQSSQSNRGKRKTGKEIRDTRALDSDATDAGTEESQEASTAEKNERHSCSSRSKRTVPQHPLNQ

LQGSQLSQVTRRKQKEAPLPSHSEEPGEKQANARGRKKGLEKRLDSCEPTALFTDCNPRVVMVPMEEVVG

FGYLSQSKQTARETPAAPVEGLLTKKAVEEASTTRGRRIKEVESAELPKPVLGGKRRKVALVDVEKKPSS

NNEEAEGVEGVAPPPKLRPGRPARNAAVKVEAASQEPSTSRGRKREGLAQDPSPPARKKGRGGRAAATNV

SSPKADVGVAPASKGRRSSSLKPRVMFTGLADTTGEEIVKSLGGLVAASPSMCTHLVTDKFRRTVKALSC

IAKGIPILSMAWLDSCRASGSFIDHMPFLLKDKAAEKTMKFNLEATLGRAASEGGILNGWGLHATPGVLP

PPQDMKEIVSCAGGKYLAKMPTRYADKIVIVSCEEDRRTLAQAKKSSIPVVTAEFVLSGLLRYQLDVKKH

TLT

>Dermacentor andersoni

MDGNDLEMTQVIHGQDDSDDEAEREIHAILKLDDQDAPAAYQTFNLKPGKNMVGRSRTCDVIIENYAVSK

QHAIIDVGGNSCTIMDLGSQNKVKIGKRTLKPNCQYNLDYNEEFCIAGLRARVLCDQNNGAKSDAGSDTC

SESLLTAIEVATDQKGVDAEVGDNADEPEPSAPVAGLDEAMGTTVNSKLETSNDRTAAKSQNAKACSSEC

QSSGSFTMPEMPNLDYTQTDSSQGTEPCAGADRDAQTNQEGAGAGAPAAPVPEGGNSPAVKHTGAFLCAP

TQPYSDAACGGNAGDTLEPAMADKESRLNAETQPYAGSAECPEVPSSNCGEIVNAEADETDRLNAPTQAY

EDDPSAEAERLNAPTQAYTEQDEDERLNAVTQAYTGDDDQEEEERLNAVTQPYAADVDQEEQERLNAVTQ

PYAAEVSKEEEERLNAVTQPYVEDVYQDEHDRLNAVTQAFNDKAEEENTGDGSACAPTQSEERHRRLLLG

AAELSFVLCEASQPCRDDGGDDDEEEEEFCAPTQKDESPPLKVHALLKLRRERGAVAEEDDDKTPPLSPK

TTIDETPPPSPGFVPESDPEYDDGETSMVTARRSPSIFDMTSASVYEDCVTPGAANGSPIIGSIGKVSGK

RPKRGMSLKKPTCDTVLELPSQESTAGAEWMQQQQQASSQSKASKKLTYIEEADDSRDLSAKTTDTSLAE

LSVSGAEHTTKHSSIITTCSPPKVPMELQGSAVTNKKSATTDDCDMPLLHMSESTDLDTTATDKEDSTAQ

DPAVKEGKNALEACISSEMPADTGTGTSEQAFSRKEETLEKDRPASISSESEEESGRVSRRKGRKEPSSR

TTRTKRKVASPQHEKEAPIENTAAPARRSSGRQNAGSRMKSLLSLEKRTSASGDFEEGALSQEVNDERDA

PKDDAAMEEPQGKAGRSRTRRGLKGKAAAEVPPLPPKDGLASASQPKVVSSSNEDKGENEEPSMKPSEND

SEGEADGREGTHADKDECPPKRGPLVDQKDDNAEDVMSTASLTPSLGDGDDNCTNATIDTCFSEPFPTFS

EVMAECQPFIEKTEGQDEKSEAAEAQEVTMVDLADESKAEAPATSEPEPVAAKEKARTLRSKNLKEGVAT

RKRKGSEVVEELPSARGKATRRGARKGPLTGAELLAEDSPSDIEQLEVLQSASPQQSNIARRGRPPRNAV

SRKTAPRLVVTNIVEDTELSEGEHDAGSLSAQETESIDRTPSQTSESLTPSRGKAAPRPRLAKAPLSKDL

NEGVATRKRKGPEVAEELPSMRRKATRRGAQETPPTRAALLAEDSPGDIEQPEAPQSTSPQQSNIARRGR

PLRSAVGRKTAPRVVDTKIVEDTEQSEGEADADRLSAQETESIEGTPLETSESLTPSRGEAVPRPHSSKA

PRSGARGRRQLLLLDAEQADEAASKDESDPDRLSAQDTESINGTPEELTASEGKAVPPSSKASRRGTKNA

KQSLQQDVKEADKAASDGLVAGNSGEQLHSVRTSQRGKASTKTRSGQDKEADAGAAADVSLTSVQSSQSS

QTSRRGRRGVKHSQEKIEPPVQSRLNDSTQSLSTEPQSQTSRSSQLGRRNTKQPETQENWPSEETGDNLT

EGEVHGKKLSRSGRKGTKLSQEAASKSVANAGESNPEPGSADHELQTRRTRRGRNAAEPSQNEQVMPTPE

ATGDPSENSGTEAEPPSSQTRRGRKGLKQTRNQRNKKNAPEDSNMKETPEVPKQPNSRSSKPSRTTNLSL

PRRTAAASSMEASVKMEPPSPGTSRRSSTKTAQVQLEEAEHLANSDFAEVPSKPKHKVAELKCEDISEAE

PLSSVSTTRRQLSKKPAPAPVTRKRDARRTGKKEDPTLTSVAPVLEATEETSPQCSLRRVDRKRHVASMS

FGVARHESDDEASSNEEETLDEVTVMLGPKSRRKAPVKEVAVKQEMVEEASVRRGKRKADVPVVKPEVPQ

KAKSRRDEQADSSLAPKKVTKVKPKVLFTGIDSTSTEEQVVRDLGGAIATNASMCTHLVTDKFRRTVKAL

CCIGKGTPIVDVAWIKKCQEAGAFVDHMPHMLLDKKAEKTLHFSLRDTLAKASAGGVLRGWSVHATPHVL

PSPSDMKEIVMCAGGKYLDNLPARTSTSTTVVVSCKEDLKACSRARNNGVPIVAAEFVLSGLLQHKLDME

AHRLE

>Homo sapiens

MEDTQAIDWDVEEEEETEQSSESLRCNVEPVGRLHIFSGAHGPEKDFPLHLGKNVVGRMP

DCSVALPFPSISKQHAEIEILAWDKAPILRDCGSLNGTQILRPPKVLSPGVSHRLRDQEL

ILFADLLCQYHRLDVSLPFVSRGPLTVEETPRVQGETQPQRLLLAEDSEEEVDFLSERRM

VKKSRTTSSSVIVPESDEEGHSPVLGGLGPPFAFNLNSDTDVEEGQQPATEEASSAARRG

ATVEAKQSEAEVVTEIQLEKDQPLVKERDNDTKVKRGAGNGVVPAGVILERSQPPGEDSD

TDVDDDSRPPGRPAEVHLERAQPFGFIDSDTDAEEERIPATPVVIPMKKRKIFHGVGTRG

PGAPGLAHLQESQAGSDTDVEEGKAPQAVPLEKSQASMVINSDTDDEEEVSAALTLAHLK

ESQPAIWNRDAEEDMPQRVVLLQRSQTTTERDSDTDVEEEELPVENREAVLKDHTKIRAL

VRAHSEKDQPPFGDSDDSVEADKSSPGIHLERSQASTTVDINTQVEKEVPPGSAIIHIKK

HQVSVEGTNQTDVKAVGGPAKLLVVSLEEAWPLHGDCETDAEEGTSLTASVVADVRKSQL

PAEGDAGAEWAAAVLKQERAHEVGAQGGPPVAQVEQDLPISRENLTDLVVDTDTLGESTQ

PQREGAQVPTGREREQHVGGTKDSEDNYGDSEDLDLQATQCFLENQGLEAVQSMEDEPTQ

AFMLTPPQELGPSHCSFQTTGTLDEPWEVLATQPFCLRESEDSETQPFDTHLEAYGPCLS

PPRAIPGDQHPESPVHTEPMGIQGRGRQTVDKVMGIPKETAERVGPERGPLERETEKLLP

ERQTDVTGEEELTKGKQDREQKQLLARDTQRQESDKNGESASPERDRESLKVEIETSEEI

QEKQVQKQTLPSKAFEREVERPVANRECDPAELEEKVPKVILERDTQRGEPEGGSQDQKG

QASSPTPEPGVGAGDLPGPTSAPVPSGSQSGGRGSPVSPRRHQKGLLNCKMPPAEKASRI

RAAEKVSRGDQESPDACLPPTVPEAPAPPQKPLNSQSQKHLAPPPLLSPLLPSIKPTVRK

TRQDGSQEAPEAPLSSELEPFHPKPKIRTRKSSRMTPFPATSAAPEPHPSTSTAQPVTPK

PTSQATRSRTNRSSVKTPEPVVPTAPELQPSTSTDQPVTSEPTSQVTRGRKSRSSVKTPE

TVVPTALELQPSTSTDRPVTSEPTSQATRGRKNRSSVKTPEPVVPTAPELQPSTSTDQPV

TSEPTYQATRGRKNRSSVKTPEPVVPTAPELRPSTSTDRPVTPKPTSRTTRSRTNMSSVK

TPETVVPTAPELQISTSTDQPVTPKPTSRTTRSRTNMSSVKNPESTVPIAPELPPSTSTE

QPVTPEPTSRATRGRKNRSSGKTPETLVPTAPKLEPSTSTDQPVTPEPTSQATRGRTNRS

SVKTPETVVPTAPELQPSTSTDQPVTPEPTSQATRGRTDRSSVKTPETVVPTAPELQASA

STDQPVTSEPTSRTTRGRKNRSSVKTPETVVPAAPELQPSTSTDQPVTPEPTSRATRGRT

NRSSVKTPESIVPIAPELQPSTSRNQLVTPEPTSRATRCRTNRSSVKTPEPVVPTAPEPH

PTTSTDQPVTPKLTSRATRRKTNRSSVKTPKPVEPAASDLEPFTPTDQSVTPEAIAQGGQ

SKTLRSSTVRAMPVPTTPEFQSPVTTDQPISPEPITQPSCIKRQRAAGNPGSLAAPIDHK

PCSAPLEPKSQASRNQRWGAVRAAESLTAIPEPASPQLLETPIHASQIQKVEPAGRSRFT

PELQPKASQSRKRSLATMDSPPHQKQPQRGEVSQKTVIIKEEEEDTAEKPGKEEDVVTPK

PGKRKRDQAEEEPNRIPSRSLRRTKLNQESTAPKVLFTGVVDARGERAVLALGGSLAGSA

AEASHLVTDRIRRTVKFLCALGRGIPILSLDWLHQSRKAGFFLPPDEYVVTDPEQEKNFG

FSLQDALSRARERRLLEGYEIYVTPGVQPPPPQMGEIISCCGGTYLPSMPRSYKPQRVVI

TCPQDFPHCSIPLRVGLPLLSPEFLLTGVLKQEAKPEAFVLSPLEMSST
