# Supplementary material for: Identification of plants’ functional counterpart of the metazoan mediator of DNA Damage checkpoint 1
Source: EMBO Rep. 2024 Mar 4;25(4):19. doi: 10.1038/s44319-024-00107-8 (PMC11014961; doi:10.1038/s44319-024-00107-8)
Supplement: Supplementary file 6 — Source Data Fig. 6 [file 44319_2024_107_MOESM6_ESM.zip › Figure 6/6B/EMBOR-2024-58742V1_SourceDataForFigure6B.pdf]

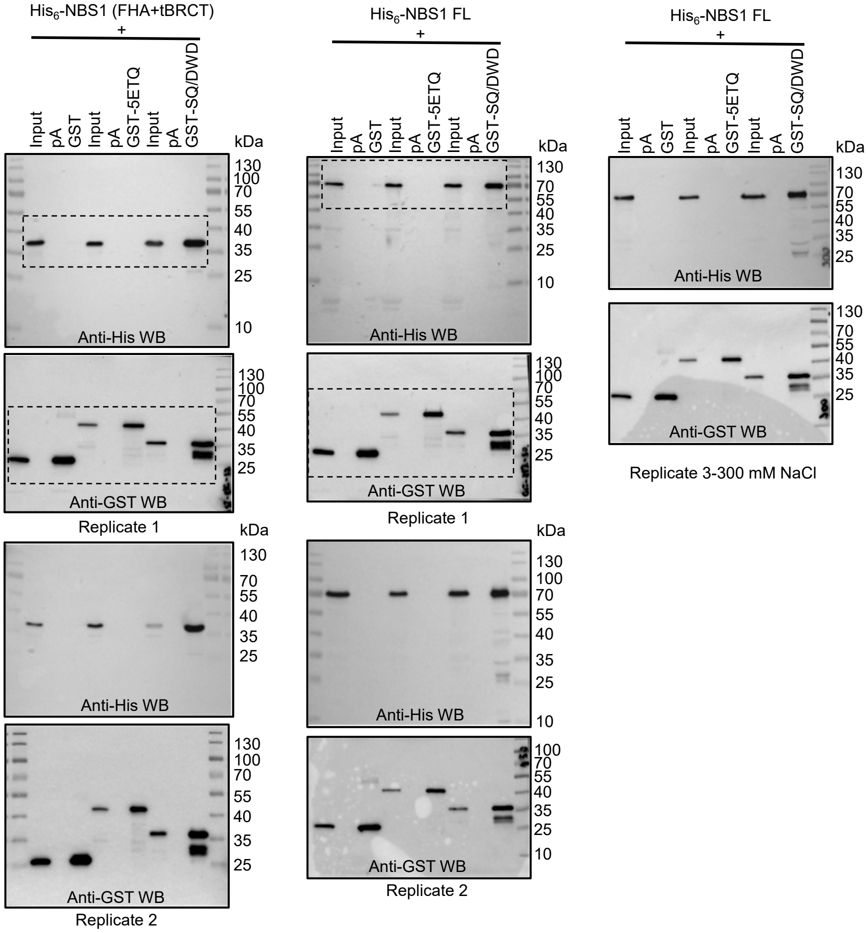

Source data for Figure 6B. Uncropped images of pull-down western blots demonstrating interaction of NBS1 full length (FL) and NBS1 FHA+tBRCT domains with SQ/DWD region of BCP4. Note that the third replicate for full-length NBS1 was done with 300 mM NaCl with the same output as with 150 mM NaCl (all other pull downs). Dashed boxes correspond to images presented in Fig 6B. Shown are chemiluminescence signals overlayed with membranes.
