# Supplementary material for: Identification of plants’ functional counterpart of the metazoan mediator of DNA Damage checkpoint 1
Source: EMBO Rep. 2024 Mar 4;25(4):19. doi: 10.1038/s44319-024-00107-8 (PMC11014961; doi:10.1038/s44319-024-00107-8)
Supplement: Supplementary file 6 — Source Data Fig. 6 [file 44319_2024_107_MOESM6_ESM.zip › Figure 6/6C/EMBOR-2024-58742V1_SourceDataForFigure6c.pdf]

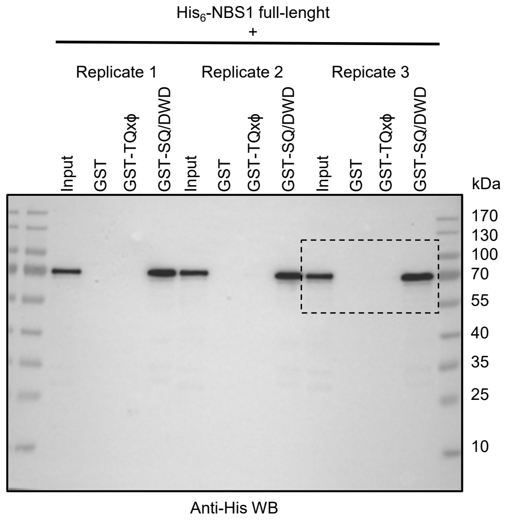

Source data for Figure 6C. Uncropped image of affinity pull-down using GST-tagged domains of BCP4 and His-tagged full-length NBS1. Dashed box correspond to image presented in Fig 6C. Shown is chemiluminescence signal overlayed with the membrane.
