# Supplementary material for: Identification of plants’ functional counterpart of the metazoan mediator of DNA Damage checkpoint 1
Source: EMBO Rep. 2024 Mar 4;25(4):19. doi: 10.1038/s44319-024-00107-8 (PMC11014961; doi:10.1038/s44319-024-00107-8)
Supplement: Supplementary file 6 — Source Data Fig. 6 [file 44319_2024_107_MOESM6_ESM.zip › Figure 6/6H/EMBOR-2024-58742V1_SourceDataForFigure6H.pdf]

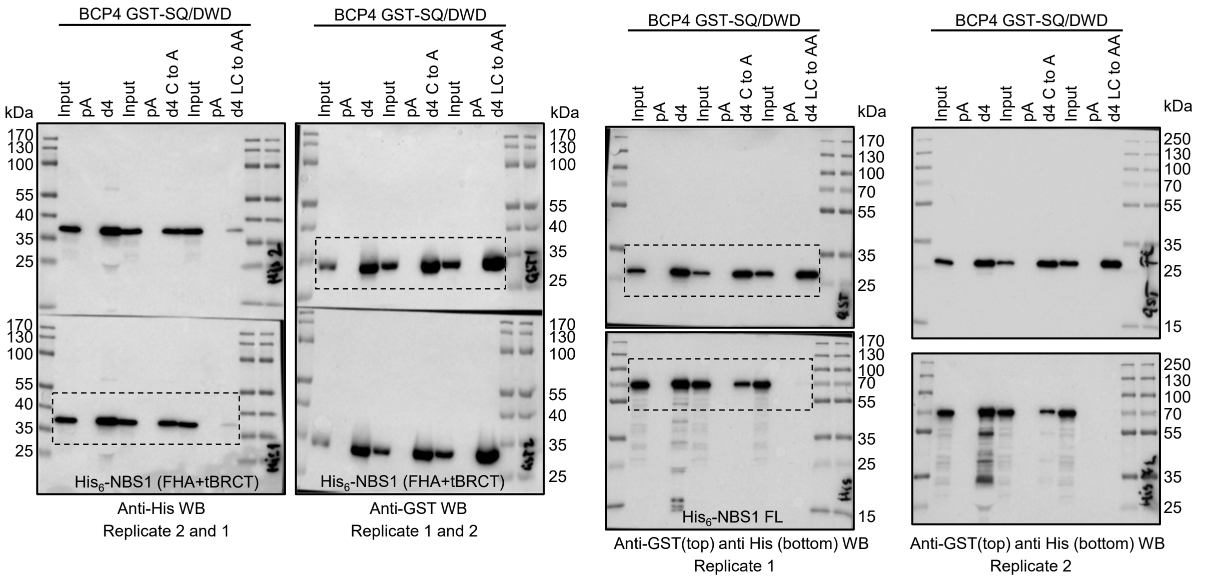

Source data for Figure 6H. Uncropped image of pull-down western blots demonstrating interaction of NBS1 full length (FL) and NBS1 FHA+tBRCT domains with SQ/DWD point mutants of BCP4. Dashed boxes correspond to images presented in Fig 6H. Shown are chemiluminescence signals overlayed with membranes.
