# Supplementary material for: Identification of plants’ functional counterpart of the metazoan mediator of DNA Damage checkpoint 1
Source: EMBO Rep. 2024 Mar 4;25(4):19. doi: 10.1038/s44319-024-00107-8 (PMC11014961; doi:10.1038/s44319-024-00107-8)
Supplement: Supplementary file 8 — Expanded View Figures [file 44319_2024_107_MOESM8_ESM.pdf]

Expanded View Figures

Figure EV1. Phylogeny of BCP1, BCP3, and BCP4.

(**A**) Maximum likelihood tree of BCP1 across Viridiplantae. The schematic presentation of BCP1 is shown at the bottom and a PHD finger present in all BCP sequences except in *Brassicaceae* is indicated. (**B**) Maximum likelihood tree of BCP3 and BCP4 across Viridiplantae. (**A**, **B**) Major clades are indicated by differently colored shading. The non-flowering land plant clade includes sequences from hornworts, mosses, liverworts, lycophytes, and monilophytes. *Thuja plicata* was the only gymnosperm used in the analysis. (**C**) Alignment of BCP3 and BCP4 proteins. Identical and conserved amino acids are indicated in red and blue letters, respectively. The positions of introns are indicated by black arrows. The tBRCT domain and three other conserved regions are shown in colored boxes. (**D**) Schematic representation of *Arabidopsis BCP* genes with exons indicated by gray boxes and introns by black lines. Exons and introns are drawn to scale according to the lengths of DNA sequences. Positions of T-DNA insertions in *bcp* mutant lines used for DNA damage sensitivity assays are indicated above each gene. Data information: A list of protein sequences used for the analysis is available from Source data for Fig. 5A,B.

 © The Author(s)

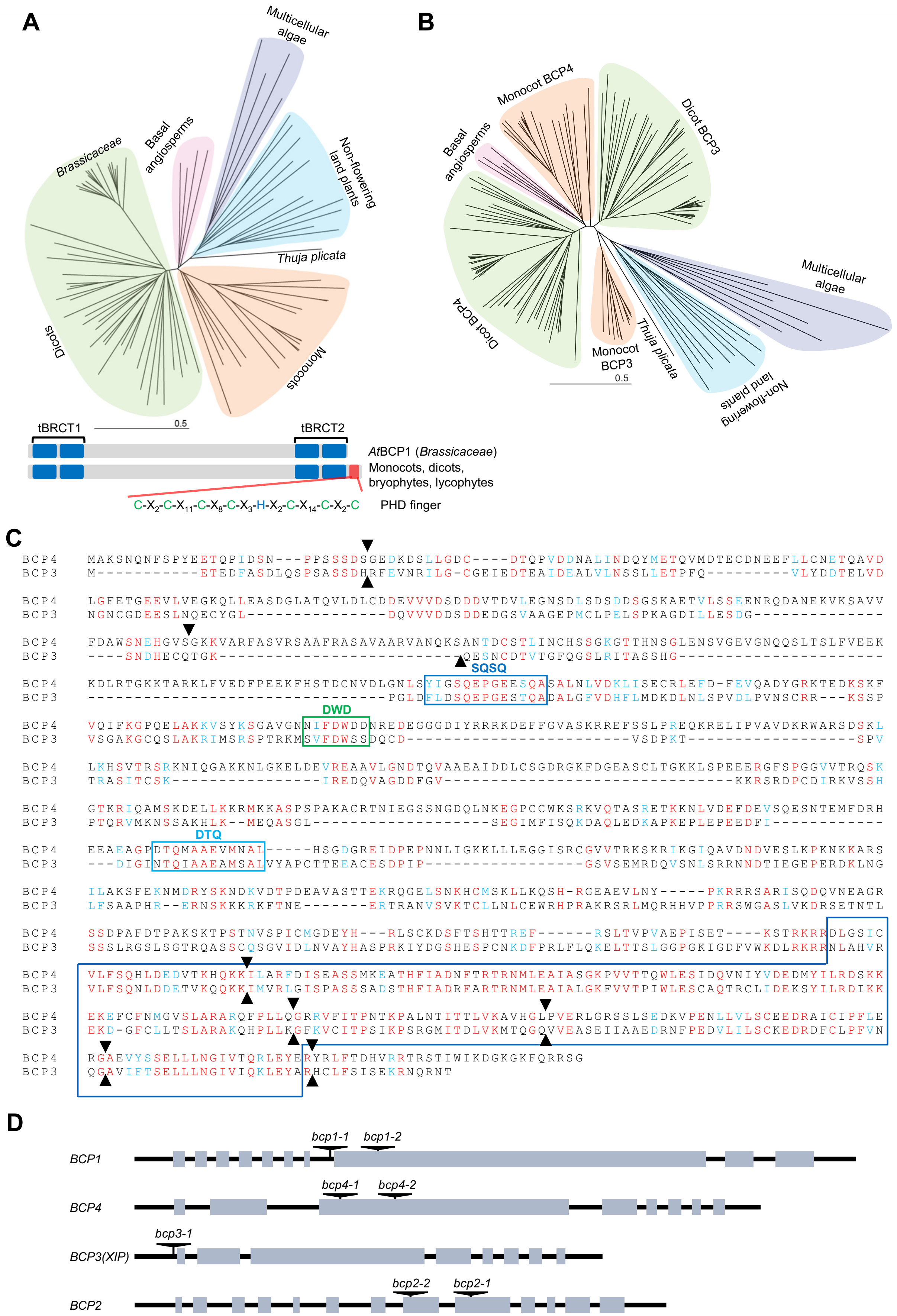
A
Multicellular algae
Basal angiosperms
Brassicaceae
Non-flowering land plants
Thuja plicata
Dicots
Monocots
0.5
tBRCT1
tBRCT2
AtBCP1 (Brassicaceae)
Monocots, dicots, bryophytes, lycophytes
C-X2-C-X11-C-X8-C-X3-H-X2-C-X14-C-X2-C
PHD finger
B
Monocot BCP4
Dicot BCP3
Basal angiosperms
Multicellular algae
Dicot BCP4
Monocot BCP3
Thuja plicata
Non-flowering land plants
0.5
C
SQSQ
DWD
DTQ
D
bcp1-1
bcp1-2
BCP1
bcp4-1
bcp4-2
BCP4
bcp3-1
BCP3(XIP)
bcp2-2
bcp2-1
BCP2

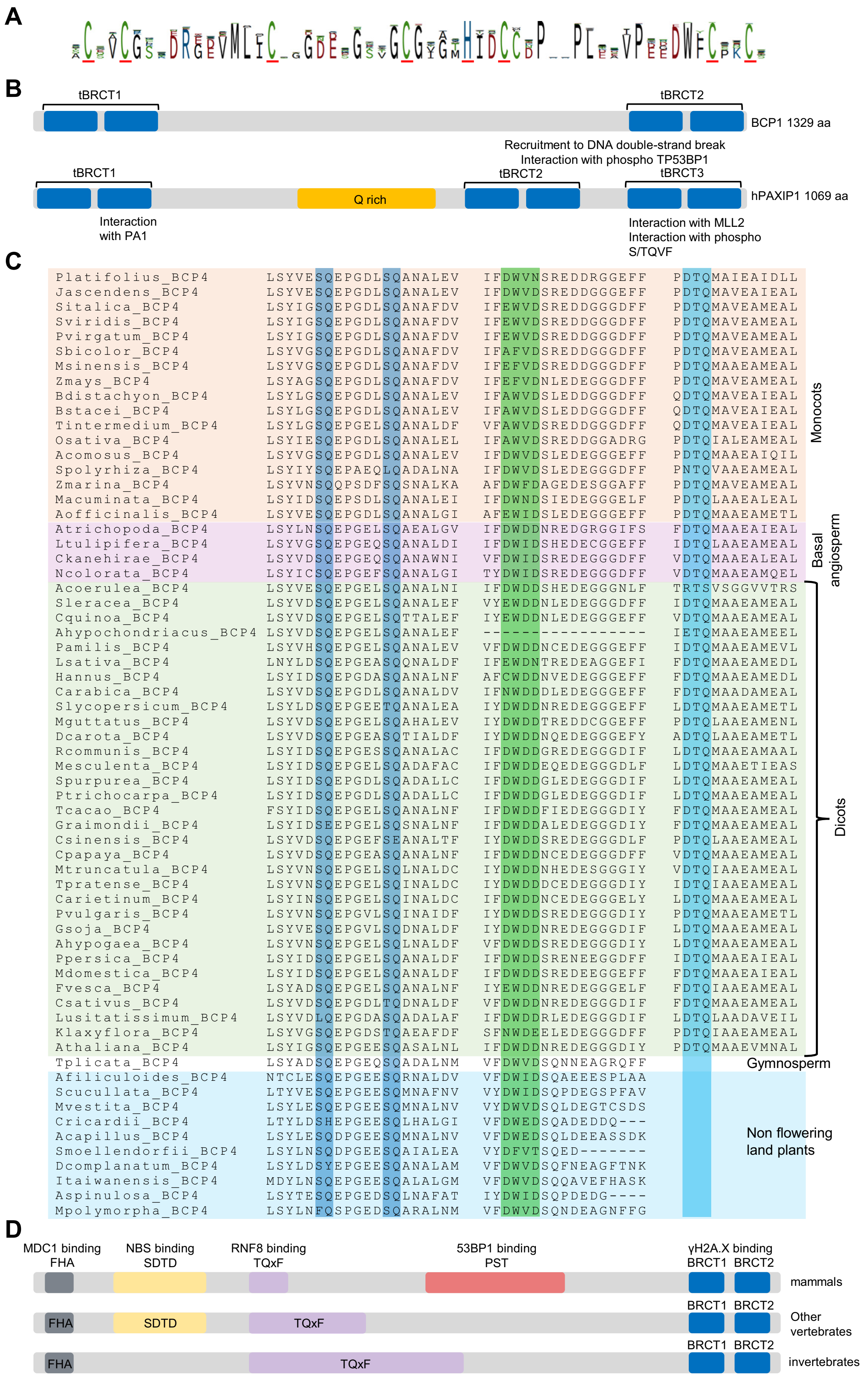
A
B
tBRCT1
tBRCT2
BCP1 1329 aa
Recruitment to DNA double-strand break
Interaction with phospho TP53BP1
tBRCT1
tBRCT2
tBRCT3
Q rich
hPAXIP1 1069 aa
Interaction
with PA1
Interaction with MLL2
Interaction with phospho
S/TQVF
C
Platifolius_BCP4 LSYVESQEPGDLSQANALEV IFDWVNSREDDRGGEFF PDTQMAIEAIDLL
Jascendens_BCP4 LSYVESQEPGDLSQANALEV IFDWVDSREDDGGGEFF PDTQMAVEAIEAL
Sitalica_BCP4 LSYIGSQEPGDLSQANAFDV IFEWVDSREDDGGGDFF PDTQMAVEAMEAL
Sviridis_BCP4 LSYIGSQEPGDLSQANAFDV IFEWVDSREDDGGGDFF PDTQMAVEAMEAL
Pvirgatum_BCP4 LSYIGSQEPGDLSQANAFDV IFEWVDSREDDGGGDFF PDTQMAVEAMEAL
Sbicolor_BCP4 LSYVGSQEPGDLSQANAFDV IFAFVDSREDDGGGDFF PDTQMAVEAMEAL
Msinensis_BCP4 LSYVGSQEPGDLSQANAFDV IFEFVDSREDDGGGDFF PDTQMAAEAMEAL
Zmays_BCP4 LSYAGSQEPGDLSQANAFDV IFEFVDNLEDEGGGDFF PDTQMAVEAMEAL
Bdistachyon_BCP4 LSYLGSQEPGDLSQANALEV IFAWVDSLEDDGGGDFF QDTQMAVEAIEAL
Bstacei_BCP4 LSYLGSQEPGDLSQANALEV IFAWVDSLEDDGGGDFF QDTQMAVEAIEAL
Tintermedium_BCP4 LSYLGSQEPGELSQANALDF VFAWVDSREDDGGGDFF QDTQMAVEAIEAL
Osativa_BCP4 LSYIESQEPGDLSQANALEL IFAWVDSREDDGGADRG PDTQIALEAMEAL
Acomosus_BCP4 LSYVGSQEPGDLSQANALEV IFDWVDSLEDEGGGEFF PDTQMAAEAIQIL
Spolyrhiza_BCP4 LSYIYSQEPAEQLQADALNA IFDWVDSLEDEGGGDFF PNTQVAAEAMEAL
Zmarina_BCP4 LSYVNSQQPSDFSQSNALKA AFDWFDAGEDESGGAFF PDTQMAVEAMEAL
Macuminata_BCP4 LSYIDSQEPSDLSQANALEI IFDWNDSIEDEGGGELF PDTQLAAEALEAL
Aofficinalis_BCP4 LSYVESQEPGDLSQANALGI AFEWIDSLEDEGGGEFF PDTQMAAEAMETL
Atrichopoda_BCP4 LSYLNSQEPGELSQAEALGV IFDWDDNREDGRGGIFS FDTQMAAEAIEAL
Ltulipifera_BCP4 LSYVGSQEPGEQSQANALDI IFDWIDSHEDECGGEFF IDTQLAAEAMEAL
Ckanehirae_BCP4 LSYVDSQEPGEQSQANAWNI VFDWIDSREDEGGGDFF VDTQMAAEALEAL
Ncolorata_BCP4 LSYICSQEPGEFSQANALGI TYDWIDSREDEGGGDFF VDTQMAAEAMQEL
Acoerulea_BCP4 LSYVESQEPGELSQANALNI IFDWDDSHEDEGGGNLF TRTSVSGGVVTRS
Sleracea_BCP4 LSYVDSQEPGELSQANALEF VYEWDDNLEDEGGGDFF IDTQMAAEAMEAL
Cquinoa_BCP4 LSYVDSQEPGELSQTTALEF IYEWDDNLEDEGGGEFF VDTQMAAEAMEAL
Ahypochondriacus_BCP4 LSYVDSQEPGELSQANALEF ----------------- IETQMAAEAMEEL
Pamilis_BCP4 LSYVHSQEPGELSQANALEV VFDWDDNCEDEGGGEFF IDTQMAAEAMEVL
Lsativa_BCP4 LNYLDSQEPGEASQQNALDF IFEWDNTREDEAGGEFI FDTQMAAEAMEDL
Hannus_BCP4 LSYIDSQEPGDASQANALNF AFCWDDNVEDEGGGEFF FDTQMAAEAMEDL
Carabica_BCP4 LSYVDSQEPGDLSQANALDV IFNWDDDLEDEGGGEFF FDTQMAADAMEAL
Slycopersicum_BCP4 LSYLDSQEPGEETQANALEA IYDWDDNREDEGGGEFF LDTQMAAEAMETL
Mguttatus_BCP4 LSYVDSQEPGELSQAHALEV IYDWDDTREDDCGGEFF PDTQLAAEAMENL
Dcarota_BCP4 LSYVDSQEPGEASQTIALDF IYDWDDNQEDEGGGEFY ADTQLAAEAMETL
Rcommunis_BCP4 LSYIDSQEPGESSQANALAC IFDWDDGREDEGGGDIF LDTQMAAEAMAAL
Mesculenta_BCP4 LSYIDSQEPGELSQADAFAC IFDWDDEQEDEGGGDLF LDTQMAAETIEAS
Spurpurea_BCP4 LSYIDSQEPGDLSQADALLC IFDWDDGLEDEGGGDIF LDTQMAAEAMEAL
Ptrichocarpa_BCP4 LSYIDSQEPGDLSQADALLC IFDWDDGLEDEGGGDIF LDTQMAAEAMEAL
Tcacao_BCP4 FSYIDSQEPGELSQANALNF IFDWDDFIEDEGGGDIY FDTQMAAEAMEAL
Graimondii_BCP4 LSYIDSEEPGELSQSNALNF IFDWDDALEDEGGGDIY FDTQMAAEAMEAL
Csinensis_BCP4 LSYVDSQEPGEFSEANALTF IYDWDDSREDEGGGDLF PDTQLAAEAMEAL
Cpapaya_BCP4 LSYVDSQEPGEASQANALNF IFDWDDNCEDEGGGEFF VDTQMAAEAMEAL
Mtruncatula_BCP4 LSYVNSQEPGELSQLNALDC IYDWDDNHEDESGGGIY VDTQIAAEAMEAL
Tpratense_BCP4 LSYVNSQEPGELSQINALDC IYDWDDDCEDEGGGDIY IDTQIAAEAMEAL
Carietinum_BCP4 LSYINSQEPGELSQINALDC IFDWDDNCEDEGGGELY LDTQMAAEAMEAL
Pvulgaris_BCP4 LSYVNSQEPGVLSQINAIDF IYDWDDSREDEGGGDIY PDTQMAAEAMETL
Gsoja_BCP4 LSYVESQEPGVLSQDNALDF IYDWDDNREDEGGGDIF LDTQMAAEAMEAL
Ahypogaea_BCP4 LSYVNSQEPGELSQLNALDF VFDWDDSREDEGGGDIY LDTQMAAEAMEAL
Ppersica_BCP4 LSYIDSQEPGELSQANALDF IFDWDDSRENEGGGDFF IDTQMAAEAIEAL
Mdomestica_BCP4 LSYIDSQEPGELSQANALDF IFDWDDSREDEEGGEFF FDTQMAAEAIEAL
Fvesca_BCP4 LSYADSQEPGELSQANALNF IYEWDDNREDEGGGELF FDTQIAAEAMEAL
Csativus_BCP4 LSYVDSQEPGDLTQDNALDF VFDWDDNREDEGGGDIF FDTQMAAEAMEAL
Lusitatissimum_BCP4 LSYVDLQEPGDASQADALAF IFDWDDRLEDEGGGDIF LDTQLAADAVEIL
Klaxyflora_BCP4 LSYVGSQEPGDSTQAEAFDF SFNWDEELEDEGGGDFF PDTQIAAEAMEAL
Athaliana_BCP4 LSYIGSQEPGEESQASALNL IFDWDDNREDEGGGDIY PDTQMAAEVMNAL
Tplicata_BCP4 LSYADSQEPGEQSQADALNM VFDWVDSQNNEAGRQFF
Afiliculoides_BCP4 NTCLESQEPGEESQRNALDV VFDWIDSQAEEESPLAA
Scucullata_BCP4 LTYVESQEPGEESQMNAFNV VYDWIDSQPDEGSPFAV
Mvestita_BCP4 LSYLESQEPGEESQMNALNV VYDWVDSQLDEGTCSDS
Cricardii_BCP4 LTYLDSHEPGEESQLHALGI VFDWEDSQADEDDQ---
Acapillus_BCP4 LSYLESQDPGEESQMNALNV VFDWEDSQLDEEASSDK
Smoellendorfii_BCP4 LSYLNSQDPGEESQAIALEA VYDFVTSQED-------
Dcomplanatum_BCP4 LSYLDSYEPGEESQANALAM VFDWVDSQFNEAGFTNK
Itaiwanensis_BCP4 MDYLNSQEPGEESQALALGM VFDWVDSQQAVEFHASK
Aspinulosa_BCP4 LSYTESQEPGEDSQLNAFAT IYDWIDSQPDEDG----
Mpolymorpha_BCP4 LSYLNFQSPGEDSQARALNM VFDWVDSQNDEAGNFFG
Monocots
Basal angiosperm
Dicots
Gymnosperm
Non flowering land plants
D
MDC1 binding
FHA
NBS binding
SDTD
RNF8 binding
TQxF
53BP1 binding
PST
γH2A.X binding
BRCT1 BRCT2
mammals
FHA
SDTD
TQxF
BRCT1 BRCT2
Other vertebrates
FHA
TQxF
BRCT1 BRCT2
invertebrates

 © The Author(s)

Figure EV2. Primary sequences analysis of BCP proteins.

(**A**) The consensus sequence of the PHD finger from BCP1 derived from the alignment of plant BCP1 proteins. Cysteine and histidine residues characteristic of PHD fingers are underlined. (**B**) Schematic representation of *Arabidopsis* BCP1 and human PAXIP1 proteins. Conserved domains and motifs of PAXIP1 and their assigned functions are indicated. A list of plant species and the corresponding protein sequences used for the analysis in (**A**, **B**) are available from Source data. (**C**) Alignment of SQSQ, DWD, and DTQ sequence motifs from BCP4. The signature motifs are shaded in blue and green. (**D**) Schematic representation of MDC1 proteins from invertebrate, vertebrate (except mammals), and mammalian species. Conserved domains and motifs and their assigned functions are indicated. Source data are available online for this figure.

© The Author(s)

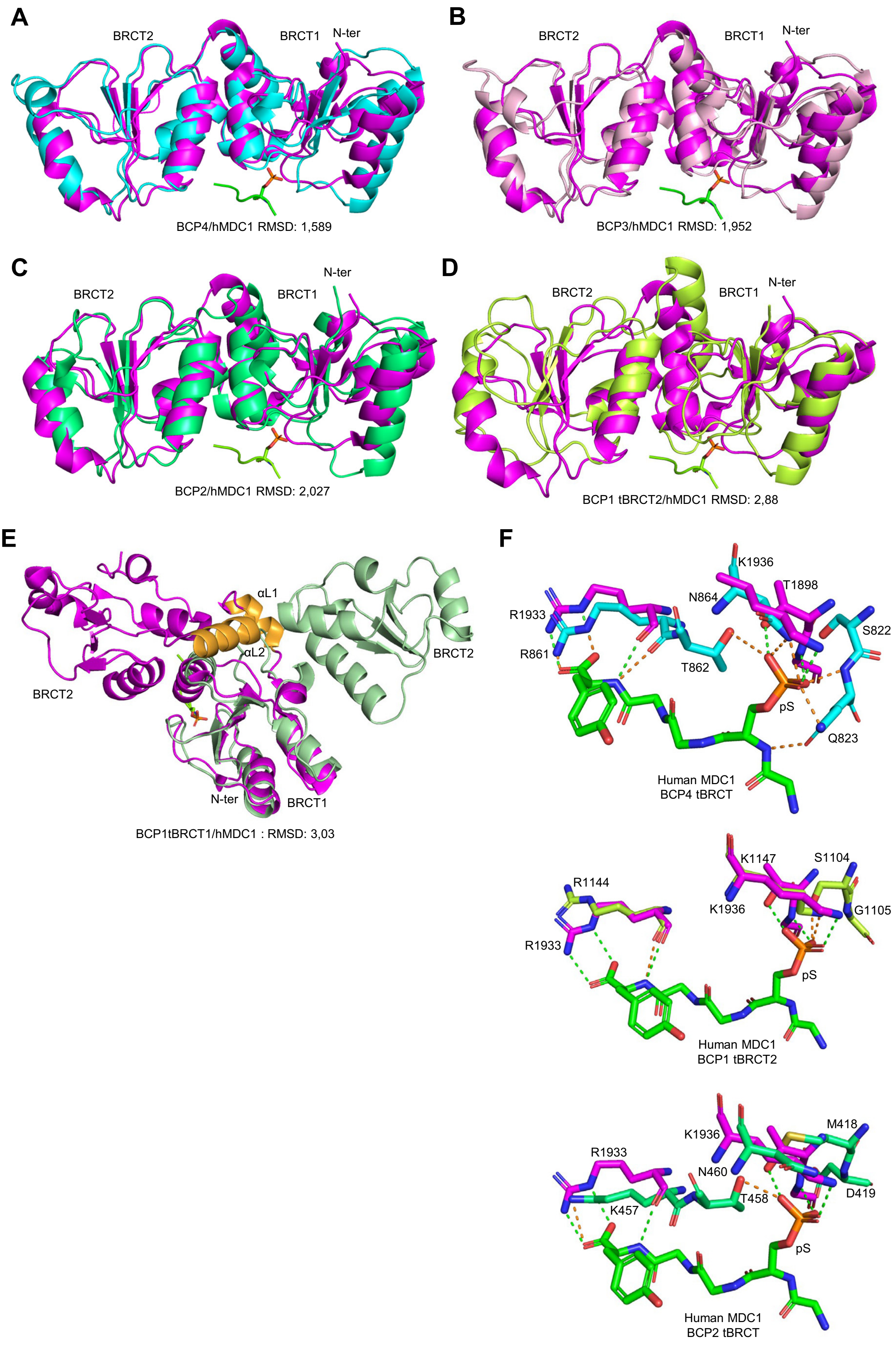
A
BRCT2
BRCT1
N-ter
BCP4/hMDC1 RMSD: 1,589
B
BRCT2
BRCT1
N-ter
BCP3/hMDC1 RMSD: 1,952
C
BRCT2
BRCT1
N-ter
BCP2/hMDC1 RMSD: 2,027
D
BRCT2
BRCT1
N-ter
BCP1 tBRCT2/hMDC1 RMSD: 2,88
E
αL1
αL2
BRCT2
BRCT2
N-ter
BRCT1
BCP1tBRCT1/hMDC1 : RMSD: 3,03
F
K1936
T1898
N864
R1933
S822
R861
T862
pS
Q823
Human MDC1
BCP4 tBRCT
R1144
K1147
S1104
K1936
G1105
R1933
pS
Human MDC1
BCP1 tBRCT2
M418
K1936
R1933
N460
D419
T458
K457
pS
Human MDC1
BCP2 tBRCT

 © The Author(s)

Figure EV3. Predicted structures of BCP proteins.

(**A–E**) AlphaFold2 models of the tBRCT domains of BCP4 (**A**), BCP3 (**B**), BCP2 (**C**), and BCP1 (**D**, **E**) superimposed with a structure of human MDC1 in complex with the phosphorylated C-terminal peptide of H2A.X (Stucki et al, 2005). Note that tBRCT1 of BCP1 overlaps only with MDC1 N-terminal BRCT domain. This is presumably due to the absence of alpha helices (αL1 and αL2, in orange) in tBRCT1 of BCP1 connecting two BRCT domains as indicated in orange. In all panels, human MDC1 tBRCT is purple colored. (**F**) Comparison of interactions of the tBRCT domains of BCP1 and BCP2 with phosphorylated C-terminal peptide of H2A.X. Published (MDC1; Stucki et al, 2005) and AlphaFold2 predicted (BCP1 and BCP2) contacts of amino acids with pSer of H2A.X are indicated respectively with green and orange dotted lines. For a comparison, BCP4 is displayed to indicate reduced abilities of the tBRCT domains of BCP1 and BCP2 to contact pSer of H2A.X. Human MDC1 tBRCT is purple colored.

© The Author(s)

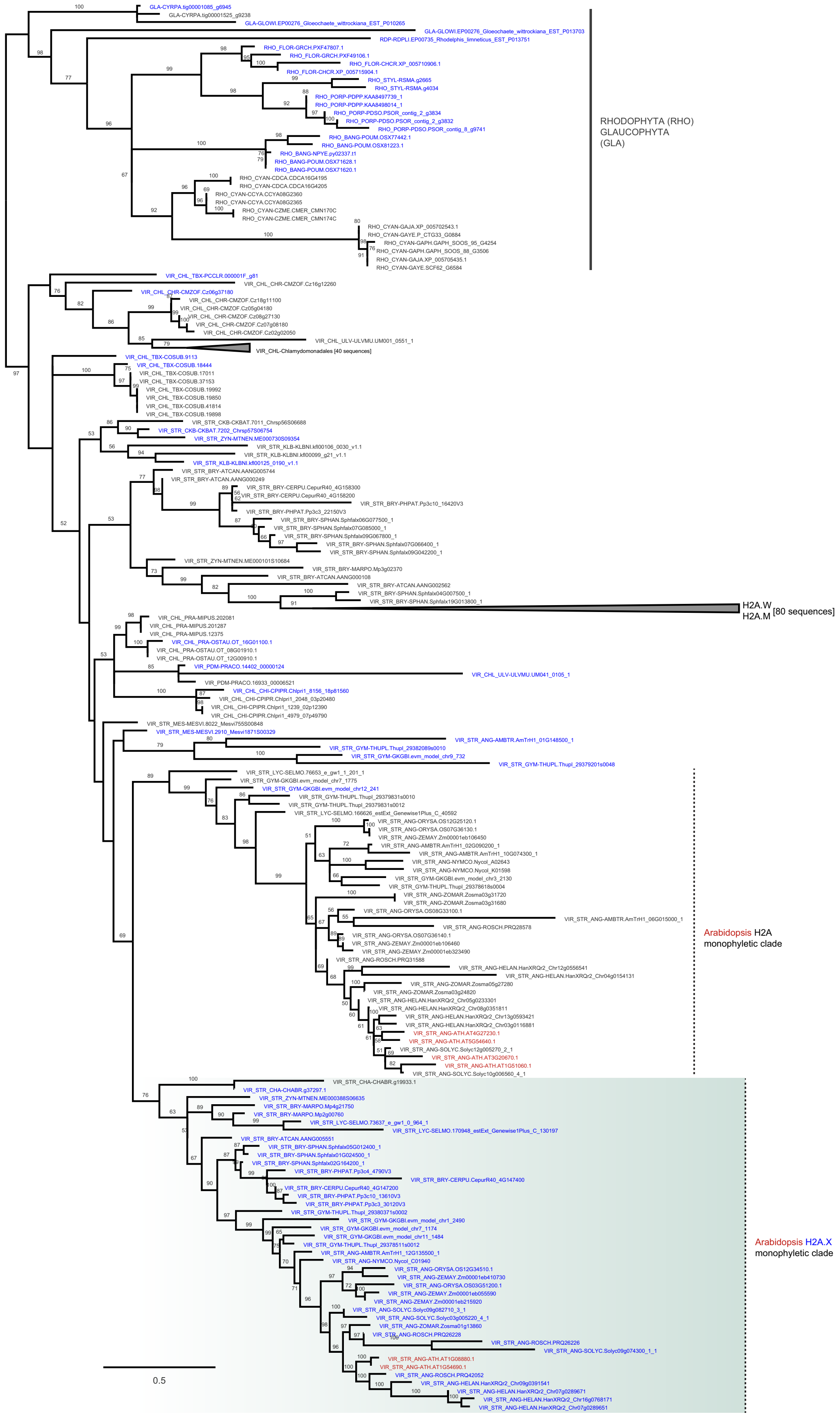
RHODOPHYTA (RHO)
GLAUCOPHYTA (GLA)
VIR_CHL-Chlamydomonadales [40 sequences]
H2A.W
H2A.M [80 sequences]
Arabidopsis H2A
monophyletic clade
Arabidopsis H2A.X
monophyletic clade
0.5

Figure EV4. Phylogeny of H2A variants from Archaeplastida H2A orthogroup.

The phylogenetic positions of *Arabidopsis* H2A variants are marked in red. H2A.X sequences with a SQEF/Y motif or SQEF-like (SQ + E/D + F/I/L/V/Y) motif at the C-terminus are highlighted in blue. Note that in green algae, red algae, and glaucophytes H2A and SQEF/Y motif-containing H2As do not form separate clades.

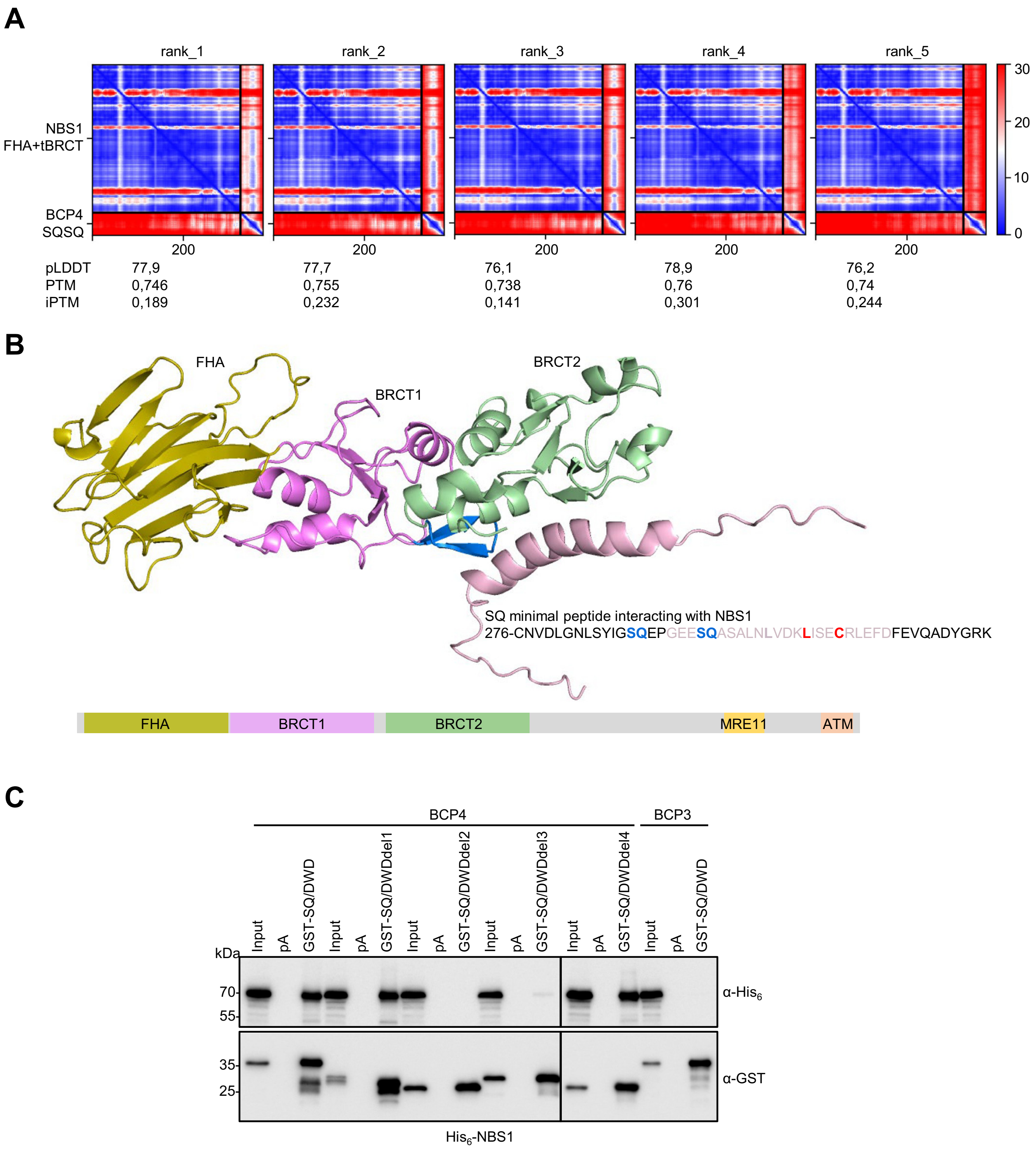

Figure EV5. Predicted structure of NBS1 and its interaction with SQ/DWD region of BCP4.

(**A**) PAE plots of NBS1 (1–324) and BCP4 SQ/DWD (276–325), with calculated predicted local distance difference test (pLDDT), predicted template modeling (PTM), and interface-predicted template modeling (iPTM) scores. (**B**) AlphaFold2 model of *Arabidopsis* NBS1 in complex with SQ/DWD region of BCP4 (top panel). A sequence of BCP4 minimal peptide interacting with NBS1 is indicated with Leu and Cys residues involved in interaction with NBS1 highlighted in red. Schematic presentation of NBS1 with indicated conserved domains (bottom panel). (**C**) Co-purification of NBS1 with GST-tagged deletion 4 of BCP4 SQ/DWD motif and its point mutants. Source data are available online for this figure.

 © The Author(s)
